# Embracing the dropouts in single-cell RNA-seq analysis

**DOI:** 10.1038/s41467-020-14976-9

**Published:** 2020-03-03

**Authors:** Peng Qiu

**Affiliations:** Department of Biomedical Engineering, Georgia Institute of Technology and Emory University, 950 Atlantic Dr. NW, Atlanta, GA 30332 USA

**Keywords:** Biotechnology, Computational biology and bioinformatics

## Abstract

One primary reason that makes single-cell RNA-seq analysis challenging is dropouts, where the data only captures a small fraction of the transcriptome of each cell. Almost all computational algorithms developed for single-cell RNA-seq adopted gene selection, dimension reduction or imputation to address the dropouts. Here, an opposite view is explored. Instead of treating dropouts as a problem to be fixed, we embrace it as a useful signal. We represent the dropout pattern by binarizing single-cell RNA-seq count data, and present a co-occurrence clustering algorithm to cluster cells based on the dropout pattern. We demonstrate in multiple published datasets that the binary dropout pattern is as informative as the quantitative expression of highly variable genes for the purpose of identifying cell types. We expect that recognizing the utility of dropouts provides an alternative direction for developing computational algorithms for single-cell RNA-seq analysis.

## Abstract (web summary)

The analysis of RNA-seq data is complicated by dropouts, and these are usually treated as a problem to be addressed. Here, Peng Qiu uses dropouts as a source of information and presents a co-occurrence clustering algorithm to cluster cells based on the dropout pattern; this could be a complementary approach to existing methods.

## Introduction

Single-cell RNA sequencing (scRNA-seq) is a powerful technology capable of unveiling cellular heterogeneity of the transcriptome at single-cell resolution, producing insights toward subpopulation structures and progression trajectories which would be hidden in bulk cell population RNA sequencing analyses^1–3^. Enabled by scRNA-seq advances including SMART-seq^4^, CEL-seq^5^, Dropseq^6^, InDrop^7^, Chromium 10X^8^, SCI-seq^9^ and SPLiT-seq^10^, scRNA-seq is increasingly used and offers the promise of addressing a variety of biology questions, such as intra-population heterogeneity and subpopulation identification^11^, developmental trajectories^12^, and regulatory mechanisms^13,14^.

scRNA-seq experiments often generate large amounts of data, containing whole-genome gene expression measurements of thousands or more individual cells, which presents challenges in computational analysis and interpretation of the data^15^. There are several reasons why computational analysis of scRNA-seq data is challenging, such as high dimensionality, measurement noise, detection limit, unbalanced size between rare and abundant populations, etc. One important characteristic of scRNA-seq data that feeds into all these challenges is a phenomenon called “dropout”, where a gene is observed at a low or moderate expression level in one cell but is not detected in another cell of the same cell type^16^. These dropout events occur due to the low amounts of mRNA in individual cells and inefficient mRNA capture, as well as the stochasticity of mRNA expression. As a result of the dropouts, the scRNA-seq data is often highly sparse. The excessive zero counts cause the data to be zero-inflated, only capturing a small fraction of the transcriptome of each cell.

Almost all existing computational methods developed for scRNA-seq adopted gene selection and dimension reduction strategies to address the dropouts^17,18^. A common practice for clustering and trajectory finding methods is to preprocess the data by selecting highly variable genes and performing dimension reduction using principal component analysis (PCA)^19^ or t-Distributed Stochastic Neighbor Embedding (tSNE)^20^. Examples include Seurat^6,13,21^, pcaReduce^22^, MNN batch-effect-correction^23^, RaceID3^24^, TSCAN^25^, STREAM^26^, and many others. There are also imputation methods designed to explicitly remove dropouts. A few recent examples include MAGIC^27^, SAVER^28^, scImpute^29^, and RESCUE^30^. These imputation methods typically use highly variable genes and dimension reduction to define gene–gene similarities or cell–cell similarities, which provide the basis for imputing the dropouts with appropriate values. The essential commonality of all these methods is to focus the analysis on highly variable genes that are less affected by dropouts, which has been proven effective because the cell-to-cell heterogeneity of major phenotypes can often be captured by genes exhibiting high variability. However, the selection of highly variable genes can be sensitive to normalization and imputation, which affects the results of the subsequent clustering and trajectory analysis. In addition, genes that are not highly variable may be useful for defining rare cell subpopulations.

The potential for leveraging the dropouts has been hinted in the literature. M3Drop is a gene selection algorithm by modeling the relationship between average detected expression and dropout rate^31^, and showed that gene with higher than expected number of dropouts were useful for mapping scRNA-seq datasets across experiments^32^. In a recent algorithm termed scBFA, it was shown that dimension reduction of the binary zero/non-zero pattern of highly variable genes produced features that accurately classified cell types in multiple scRNA-seq datasets^33^.

Here, in contrast to the majority of existing algorithms that treat the dropouts as a problem that needs to be fixed, we decide to embrace the dropouts as a useful signal. We hypothesize that although the sparsity of scRNA-seq data is primarily caused by dropouts due to noise and stochasticity at single-cell level, genes in the same pathway tend to exhibit similar dropout pattern (i.e., binary zero/non-zero pattern) across various cell types, and can serve as the basis for detecting cell types. In our analysis, we first binarize the scRNA-seq count matrix, turning all the non-zero observations into one. The binarized data is what we refer to as the dropout pattern. We present an iterative co-occurrence clustering algorithm to identify cluster cells based on the binary dropout pattern. Although the quantitative information of detected gene expression levels is removed after the data is binarized, we demonstrate with multiple published datasets that the co-occurrence clustering of the dropout pattern is able to effectively identify cell populations, based on gene pathways beyond the highly variable genes. This suggests the binary dropout pattern in scRNA-seq data is as informative as the quantitative expression of highly variable genes.

## Results

### The co-occurrence clustering algorithm

**Fig. 1:**
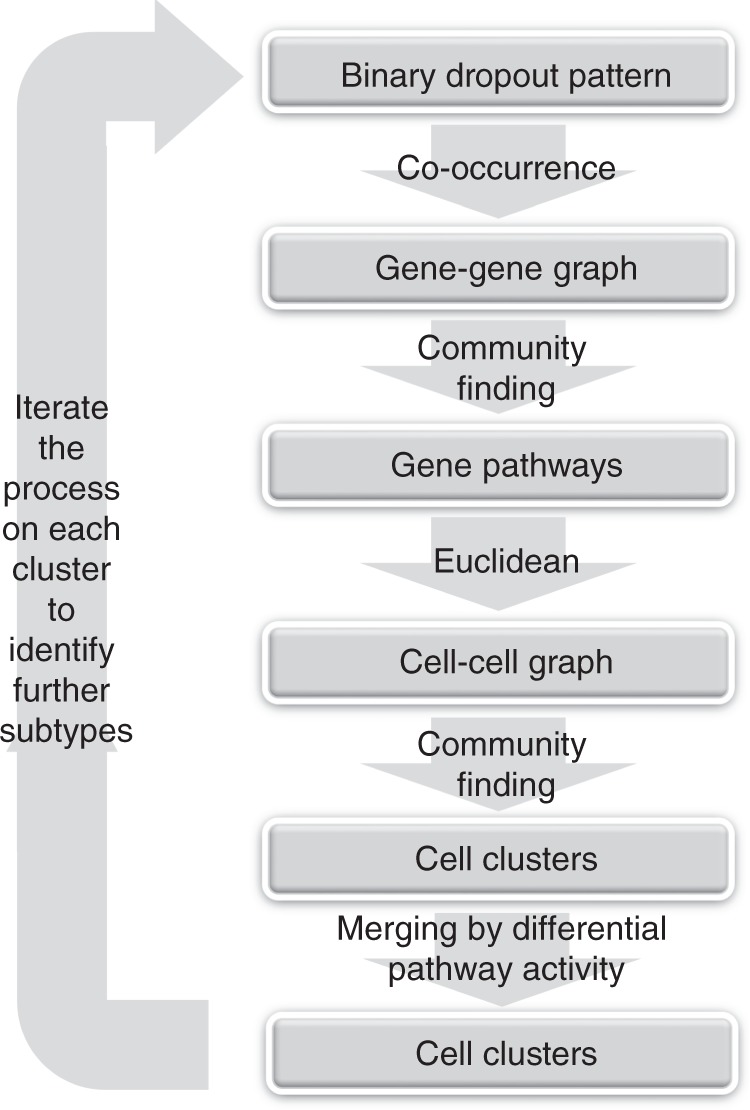
Flowchart of co-occurrence clustering. The co-occurrence clustering algorithm is a divisive hierarchical process that iteratively identifies gene pathways based on binary dropout patterns and cell clusters based on the gene pathways.

To identify cell populations based on the dropout pattern, we developed a co-occurrence clustering algorithm. A flowchart of the algorithm is shown in Fig. 1. The algorithm works in a hierarchically divisive manner, and iteratively performs gene pathway identification and cell type discovery. The starting point of the algorithm is a root node at the top of a hierarchical tree, which contains all the cells in the data. The algorithm first computes a statistical measure for co-occurrence between each pair of genes, which quantifies whether two genes tend to be co-detected in a common subset of cells. The co-occurrence measures are filtered and adjusted by the Jaccard index^34^, which defines a weighted gene–gene graph. The gene–gene graph is partitioned into gene clusters using community detection (e.g., Louvain algorithm^35^). The resulting gene clusters contain genes that share high co-occurrence based on all cells, and can serve as pathway signatures that separate major groups of cell types in the heterogeneous population. For each computationally-derived gene cluster/pathway, the percentage of detected genes is computed for each cell. These percentages form a low-dimensional representation of the cells in the pathway activity space, where each dimension describes the activities of one gene pathway in the cells. The algorithm then builds a cell–cell graph using Euclidean distances based on the low-dimensional pathway activity representation, uses the Jaccard index to filter the cell–cell graph, and applies community detection again to partition the cell–cell graph into cell clusters. For each pair of cell clusters, three metrics (signal-to-noise ratio, mean difference and mean ratio) are used to evaluate whether any of the gene pathways show differential activities. If none of the gene pathways exhibit differential activity between the two cell clusters, these two cell clusters are merged. After merging the cell clusters according to pathway activities, each pair of the resulting cell clusters have at least one gene pathway that shows large difference between the two cell clusters. These cells clusters form children nodes of the root node, and are expected to capture the major groups of cell phenotypes in the data. In subsequent iterations, each resulting cell cluster (children node of the root node) is further divided using the same process, which identifies smaller subtypes in each major group of cell phenotypes. These subtypes form lower-level children nodes further down the hierarchical tree, which are examined in later iterations of the algorithm. A node is not further divided if the community detection step produces only one cell cluster, or all cell clusters produced by the community detection step are merged according to the gene pathway differential activity criteria. Such a node becomes a leaf of the hierarchial clustering process, and is reported as one cell type identified by the algorithm. Therefore, the merging criteria define termination of the iterations and dictate the final number of clusters identified by the algorithm.

### Dropout pattern identifies major cell types in PBMC

**Fig. 2:**
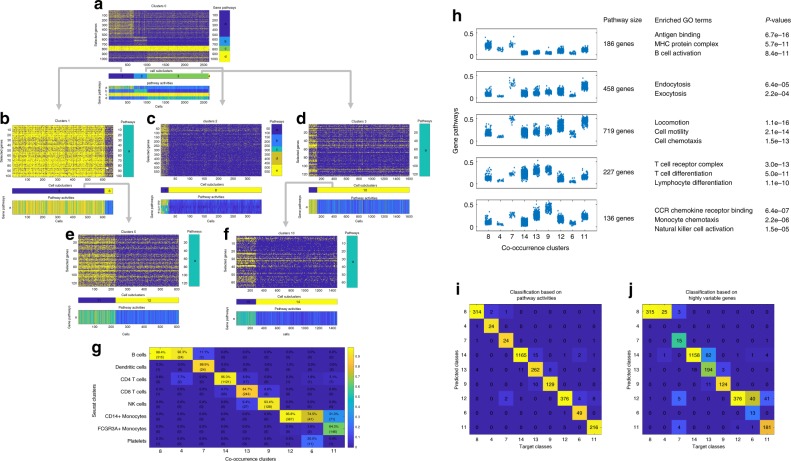
Co-occurrence clustering applied to dropout pattern in PBMC data. **a**–**g** Gene pathways and cell clusters identified in each iteration of the co-occurrence clustering algorithm. **h** Comparison between co-occurrence clusters and Seurat clusters on this dataset. **i** Pathway activities and enriched GO terms. Enrichment is evaluated by one-sided hypergeometric test on the overlap between identified pathways and GO gene sets provided by MSigDB. The reported *p*-values are unadjusted. **j**, **k** Random Forest classification and 5-fold cross-validation of the co-occurrence clusters based on pathway activities and highly variable genes.

To demonstrate the utility of dropouts and co-occurrence clustering, we examined an scRNA-seq dataset of Peripheral Blood Mononuclear Cells (PBMC) freely available from 10X Genomics. This dataset contains scRNA-seq counts data for 32,738 genes in 2700 single cells that were sequenced on the Illumina NextSeq 500. 97.41% of the count matrix were zeros. The first iteration examined the root cluster 0 which contained all 2700 cells. The algorithm constructed a gene–gene graph based on co-occurrence in the binary dropout pattern, and applied community detection to identify four gene pathways, indicated by the upper-right heatmap in Fig. 2a. Each pathway contained genes that were significantly co-detected, as shown in the upper-left heatmap of Fig. 2a. For each gene pathway, the percentage of detected genes was used to represent the detected activity of the pathway in individual cells, which was shown in the bottom heatmap of Fig. 2a. Based on the cell–cell graph constructed by the Euclidean distance of the pathway activity representation, community detection yielded fifteen cell clusters, which were subsequently merged to four-cell clusters according to merging thresholds of 1.5, 0.5, 2 for signal-to-noise ratio, mean difference and mean ratio of pathway activities between each pair of cell clusters (see details in the “Methods” section). These thresholds were chosen to ensure that all resulting cell clusters exhibit distinct dropout patterns, and the same values were used for all datasets examined in this paper. The upper-left heatmap in Fig. 2a showed the binary dropout pattern of genes in the pathways across all the individual cells, where the rows and columns were arranged by the gene pathways and cell clusters identified by the algorithm. It was obvious that distinct cell clusters can be defined by the binary dropout pattern of the identified pathways, both from the heatmap of the binarized data itself and the heatmap of the pathway activity space. In subsequent iterations, the four-cell clusters were separately examined by the same algorithm. Cell clusters 1, 2, and 3 were further divided into smaller clusters, as shown in Fig. 2b–d. Cluster 4 was not divided because the algorithm did not identify any gene pathway with genes that exhibited significant co-occurrence. Moving down the hierarchical process of the iterative algorithm, among cell clusters 5~10, only clusters 5 and 10 were further divided, as shown in Fig. 2e–f. The others were not divided due to lack of gene pathways identified in the gene–gene graph, lack of cell clusters identified in the cell–cell graph, or lack of cell clusters that exhibited differential pathway activities exceeding the thresholds in the merging step. Details of the iterative process were provided in Supplementary Note 1. Overall, co-occurrence clustering of the dropout pattern identified a total of 13 gene pathways that defined 9 cell clusters in this PBMC dataset.

This dataset was previously analyzed with Seurat, which identified 8 cell clusters using community detection based on principle component analysis of the expression data of highly variable genes^6^. The clustering results between co-occurrence clustering and Seurat were highly similar, with a Rand Index of 0.85. A detailed comparison of the two clustering results was visualized in the heatmap in Fig. 2g. Each column of the heatmap was colored by the percentages of overlap between one co-occurrence cluster and the Seurat clusters, showing that most of the co-occurrence clusters was primarily enriched by one Seurat cluster. Six Seurat clusters (Dendritic cells, CD4 T cells, CD8 T cells, NK cells, FCGR3A+ Monocytes) were captured by individual co-occurrence clusters (7, 14, 13, 9, 11). Co-occurrence clustering did not capture the Seurat cluster of Platelets. Two Seurat clusters (B Cells and CD14+ Monocytes) were divided into subtypes. Overall, most of the Seurat clusters were well-separated in the co-occurrence clustering results. In this comparison, the number of highly variable genes defined by Seurat was 1838, and the gene pathways identified by co-occurrence clustering contained a total of 1583 genes. The overlap was only 376. Many highly variable genes were not included in the co-occurrence gene pathways mainly because co-occurrence clustering worked with the binary dropout pattern. Therefore, the co-occurrence clustering and identified gene pathways were primarily driven by genes whose expression profiles were not highly variable. Although the two algorithms were based on different sets of genes and different types of signals, the general agreement between the two clustering results was striking, and suggested that the whole transcriptome binary dropout pattern was as informative as the quantitative expression of highly variable genes for the purpose of defining cell types.

The gene pathways and their activities enabled interpretation of identified cell phenotypes in terms of genes that are not highly variable. Figure 2h showed the activities of five identified gene pathways in individual cells grouped according to the co-occurrence cell clusters, as well as enriched gene ontology (GO) terms for the pathways. The enriched GO terms were consistent with known biology of various cell types. For example, MHC protein complex and antigen binding were enriched in B cells and dendritic cells. Endocytosis and cell motility were high in dendritic cells and monocytes. We applied random forest to classify the co-occurrence clusters based on the activities of co-occurrence gene pathways and expression of highly variable genes. The excellent classification performance in Fig. 2i showed that the co-occurrence clusters showed distinct pathway activities that were easily classified. Figure 2j showed that the two B cell clusters (8 and 4) and the two CD14+ monocyte clusters (12 and 6) were not separable according to the expression of highly variable genes. Random forest analysis based on two imputed versions of the data generated by MAGIC^27^ and scImpute^29^ showed almost the same results (Supplementary Fig. 1a, b), because the imputation algorithms relied on PCA for dimension reduction, where the top principle components were primarily driven by highly variable genes. These results confirmed that approaches based on highly variable genes were unable to separate the two B cell clusters and the two CD14+ monocyte clusters identified by co-occurrence clustering. The two B cell clusters (8 and 4) contained 320 and 26 cells, respectively. The relatively smaller B cell cluster (cluster 4) had lower UMI counts compared to the other B cell cluster (Supplementary Fig. 1c), leading to lower detection rates for genes (Supplementary Fig. 1d) and lower pathway activities in the co-occurrence analysis (Fig. 2h). However, based on the raw expression counts before binarization, the mean expression profiles of the two B cell clusters showed tight correlation (Supplementary Fig. 1e, f), especially after the UMI counts were library-size normalized and log transformed. The observations for the two CD14+ monocyte clusters (12 and 6) were almost the same compared to the two B cell clusters (Supplementary Fig. 1g–i). This was further confirmed by examining the raw expression counts of highly expressed genes across all cell types and highly expressed genes specific to B cells and monocytes (Supplementary Fig. 1j). The most highly expressed genes across all cell types were primarily related to ribosomal proteins, and their detected expression levels were lower in clusters 4 and 6, consistent to the fact that cells in those two clusters had relatively lower UMIs. In terms of top genes specific to B cells and monocytes (such as *CD79A, CD79B, MS4A1, CD14* and *LYZ*), expression levels in clusters 4 and 6 were in par with those in clusters 8 and 12 which contained the majority of B cells and monocytes (Supplementary Fig. 1j). Given the detected expression levels of cell type specific genes, cells in clusters 4 and 6 were likely to be biologically meaningful, rather than poor quality cells. It was interesting that such cell clusters with low UMI but high cell-type-specific gene expression were only observed in B cells and monocytes.

### Dropout pattern clusters cells in human prefrontal cortex

**Fig. 3:**
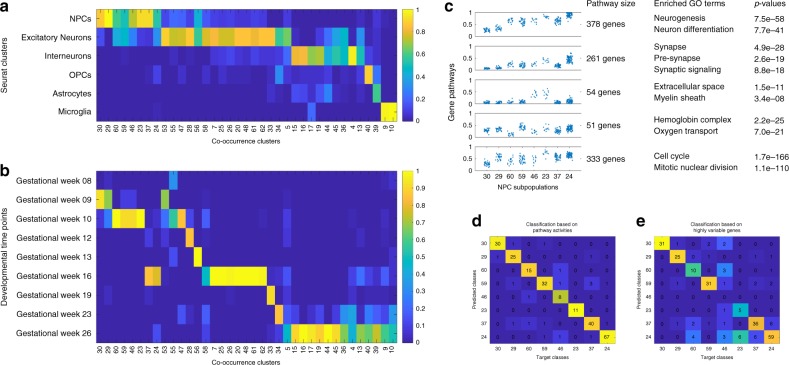
Co-occurrence clustering applied to dropout pattern in human prefrontal cortex data. **a** Comparison between co-occurrence clusters and seurat clusters. Each column was colored by the percentages of cells in one co-occurrence cluster that belonged to each of the experimentally defined cell type. **b** Comparison between co-occurrence clusters and clusters defined by developmental time points when the cells were collected. **c** Pathway activities and enriched GO terms. Enrichment is evaluated by one-sided hypergeometric test on the overlap between identified pathways and GO gene sets provided by MSigDB. The reported *p*-values are unadjusted. **d**, **e** Random Forest classification and 5-fold cross-validation of the co-occurrence clusters based on pathway activities and highly variable genes.

To demonstrate the generality of dropout as a useful signal, we further examined a scRNA-seq dataset of the developing human embryonic prefrontal cortex at gestational weeks 8 to 26^36^. The data were generated with the SMART-seq2 technology, which provided expression measurements for 24,153 genes across 2394 single cells, and the dropout rate was 82%. This dataset was previously analyzed by a combination of tSNE and Seurat, which defined six major clusters: neural progenitor cells (NPCs), excitatory neurons, interneurons, oligodendrocyte progenitor cells (OPCs), astrocytes and microglia^36^. When applied to the binary dropout pattern in this dataset, the co-occurrence clustering algorithm went through 23 iterations that identified meaningful gene pathways and cell clusters, and eventually produced a total of 38 cell clusters. Visualizations of all individual co-occurrence clustering iterations were available in Supplementary Note 2, which showed that all the identified gene pathways and cell clusters exhibited visually striking differences in their dropout patterns. Figure 3a showed that the clustering results of co-occurrence clustering and Seurat were highly consistent, and each Seruat cluster was captured by one or multiple co-occurrence clusters. Figure 3b showed that the co-occurrence clusters were also consistent with the developmental time points when the cells were collected. Clusters of NPCs were present in gestational weeks 9, 10, and 16. Clusters of excitatory neurons existed in all gestational weeks sampled. All clusters of interneurons, OPCs, astrocytes, and microglia emerged later in gestational week 26. This was consistent with known literature that in the normal developmental process, NSCs give rise to neurons first and glial cells later^37,38^.

Co-occurrence clustering identified eight clusters (30, 29, 60, 59, 46, 23, 37, and 24) corresponding to NPCs. As shown in Fig. 3c, these eight co-occurrence clusters exhibited distinct pathway activities according to six of the co-occurrence gene pathways, which were enriched for GO terms including synapse, neurogenesis, neuron differentiation, cell cycle, etc. Based on the pathway activities, random forest with 5-fold cross-validation was able to classify these eight NPC subpopulations with 92.3% accuracy shown in Fig. 3d, confirming that these co-occurrence clusters exhibited distinct dropout patterns. However, Fig. 3e showed that random forest based on the quantitative expression data of highly variable genes only achieved an accuracy of 79.8%, and was unable to classify the two rare NPC subpopulations (clusters 23 and 46) containing a dozen cells each. Random forest based on two imputed versions of the data was able to improve the classification accuracy for cluster 23 to some extent, but unable to classify cluster 46 (Supplementary Fig. 2a, b). As shown in Fig. 3b, c, majority of cells in these two rare NPC clusters belonged to gestational week 10, and showed higher activity level of a co-occurrence gene pathway enriched for extracellular space and myelin sheath. Based on the raw counts (Supplementary Fig. 2c, d), these two rare NPC clusters have similar total UMI counts compared to other cell clusters, and showed elevated expression of *PRG2* (also known as *MBP*, myelin basic protein) and *MAG* (myelin-associated glycoprotein), both associated to myelination and oligodendrocyte differentiation^39^. Although the OPCs did not emerge until gestational week 26 as shown in Fig. 3b, the two rare NPC clusters revealed NPC subpopulations that started to differentiate toward a more oligodendrogenic fate^40^ in earlier gestational week while preserving their tripotency.

### Dropout pattern delineates tissue types in Tabula Muris

To further demonstrate the generality and scalability, co-occurrence clustering was applied to the dropout patterns in a recently published compendium of mouse tissues, the Tabula Muris^41^, which contained scRNA-seq data for about 120,000 cells from 20 organs and tissue types in mouse, including skin, fat, mammary gland, heart, bladder, brain, thymus, spleen, kidney, limb muscle, tongue, marrow, trachea, pancreas, lung, large intestine, and liver. Many of these organs were processed using two technologies, SMART-seq2 on FACS-sorted cells and 10X Genomics on microfluidic droplets. The FACS-sorted SMART-seq2 dataset contained count data for 23,433 genes across 53,760 cells, with an overall dropout rate of 89%. The droplet-based 10X dataset contained count data of 70,118 cells for the same 23,433 genes, with an overall dropout rate of 93%. The Tabula Muris allowed evaluation of dropout patterns and co-occurrence clustering on datasets with similar underlying heterogeneity but profiled by two different scRNA-seq technologies.

**Fig. 4:**
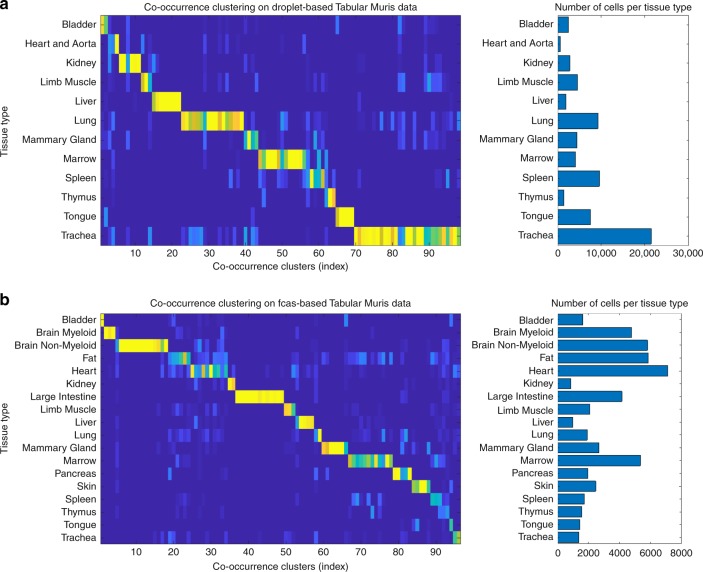
Co-occurrence clustering applied to dropout patterns in the Tabula Muris datasets. **a** Co-occurrence clustering separated tissue types in the droplet-based scRNA-seq data. **b** Co-occurrence clustering separated tissue types in the FACS-based scRNA-seq data.

**Fig. 5:**
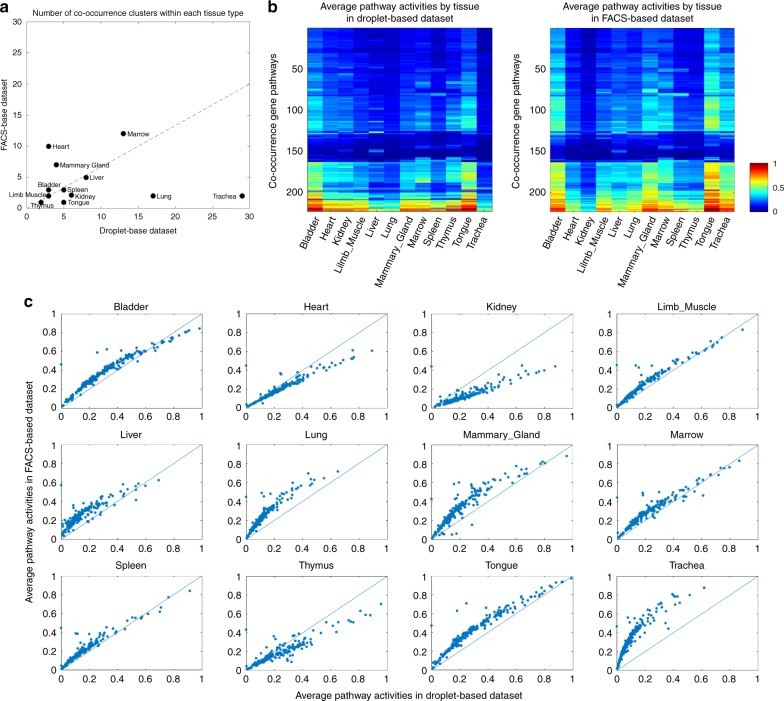
Consistency between the two Tabula Muris datasets in terms of dropout patterns. **a** The numbers of subpopulations within each of the 12 overlapping tissue types identified by co-occurrence clustering were generally in line with each other. **b** Heatmaps of activities of the 261 identified gene pathways across the two datasets, showing high consistency. **c** Scatter plots for individual tissue types, further demonstrating that pathway activities based on dropout patterns were highly correlated between the two datasets.

The dropout patterns of the droplet-based dataset and the FACS-based dataset were analyzed by co-occurrence clustering separately. In both datasets, co-occurrence clustering identified roughly 100 cell clusters. The gene pathways and cell clusters identified in each co-occurrence iteration all exhibited distinct dropout patterns that were visually obvious, as shown in visualization of each iteration of the co-occurrence clustering processes in Supplementary Notes 3 and 4. The Tabula Muris dataset provided tissue type annotations for each individual cell, which was used to evaluate whether the dropout patterns were able to delineate various tissue types. As shown in Fig. 4a, b, co-occurrence clustering of the dropout patterns successfully separated the tissue types in both datasets, and identified further subpopulations within many of the tissue types. This can also be achieved by clustering analysis based on highly variable genes, as indicated in previous literature^41^ and our own analysis (Supplementary Fig. S3a, b). The numbers of subpopulations co-occurrence clustering identified within each of the 12 overlapping tissue types in the two datasets were generally in line with each other as shown in Fig. 5a. The outliers were mainly because the distributions of cells across the tissue types were different between the two datasets. Trachea and lung were two dominant tissue types that accounted for 30% and 13% of the droplet-based dataset, whereas these two together accounted for 6% of the cells in the FACS-based dataset. In contrast, heart was the largest tissue type in the FACS-based dataset, but the smallest in the droplet-based dataset. Co-occurrence clustering identified a total of 261 gene pathways in the analyses of these two datasets. For each gene pathway, we computed its average activity (percentage of detection) for each of the 12 overlapping tissue types in the two datasets separately. The heatmaps in Fig. 5b showed the activities of the gene pathways in various tissue types were highly correlated between the two datasets. The tight correlation was further visualized in Fig. 5c, one scatter plot for each tissue type with the dots corresponding to the 261 gene pathways. For most tissue types (except heart, kidney, and thymus), the pathway activities were higher in the FACS-based dataset, consistent with the fact that the dropout rate in the FACS-based dataset was lower. The comparisons in Fig. 5 demonstrated that the dropout patterns in the two datasets were highly consistent with each other. In contrast, similar analyses based on the expression of highly variable genes showed that the expression levels were less correlated between the two datasets compared to the dropout patterns (Supplementary Fig. S3c–e). This analysis demonstrated the utility and robustness of dropout patterns in large scRNA-seq datasets generated by two different technologies, as well as the scalability of the co-occurrence clustering algorithm, which together identified tissue types and subpopulations based on the binary dropout patterns in the data.

## Discussion

Using multiple scRNA-seq datasets generated by different scRNA-seq technologies in various biology contexts, we have demonstrated that dropout is an extremely useful signal for identifying cell types, and is as informative as the quantitative expression of highly variable genes. This validates our hypothesis that different cell types exhibit distinct binary dropout patterns in scRNA-seq analysis. Since most existing computational methods for scRNA-seq treated dropout as a problem to be fixed, our analyses explore an opposite view and present a unique perspective.

In existing scRNA-seq analysis methods, feature selection is typically performed only once. The two most popular feature selection strategies are using highly variable genes and performing principal component analysis, both of which are primarily driven by genes that exhibit high variation. In the co-occurrence clustering algorithm, feature selection is re-visited in each iteration. Depending on the set of cells under consideration, each iteration builds a gene–gene graph based on co-occurrence, and applies community detection to identify gene pathways that are able to characterize the heterogeneity in the set of cells under consideration. In our analyses, the gene pathways constructed in the initial iteration are often of larger size compared to those in later iterations. This is mainly because the initial set of cells is typically more heterogeneous, containing drastically distinct cell types that can be characterized by the binary on/off expression states of many genes, whereas the sets of cells examined in subsequent iterations contain less heterogeneity manifested in relatively smaller number of genes.

In almost all of the datasets analyzed here, the co-occurrence clustering algorithm generated more clusters than the previous analyses of these datasets, which can be both a blessing and a curse. Although the dropout patterns showed clear differences among the clusters generated in each iteration (Fig. 2 and Supplementary Notes 1–4), the results presented a challenge for interpreting the biological functions and distinctions among the cell clusters. One strategy is to bring back the quantitative gene expression information, and interpret the identified cell clusters using both pathway activities defined by dropouts and expression profiles defined by highly variable genes. Binarizing scRNA-seq data to focus on the dropouts may not be the best strategy for analyzing scRNA-seq data, because the quantitative information of detected gene expression levels is ignored. However, recognizing the utility of dropouts suggests an alternative direction for developing computational algorithms for scRNA-seq. We envision future algorithms that combine quantitative expression of highly variable genes and binary dropout pattern of other genes to fully exploit the richness of scRNA-seq data.

## Methods

### Dropout pattern represented by binarizing scRNA-seq counts

The only data preprocessing required here is converting the count matrix into binary, where all the dropouts are still 0, and all the non-zero counts are turned into 1 regardless of the expression level. No normalization, transformation or imputation is required.

### Filter genes and cells before co-occurrence clustering

In each iteration, the co-occurrence clustering algorithm focuses on the dropout pattern (binarized expression data) of one cell cluster. Based on the binary dropout pattern, the algorithm filters both genes and cells. More specifically, the algorithm removes genes detected in too few cells, and removes cells in which too few genes are detected. The default thresholds for both genes and cells were 10 for all the datasets analyzed in this paper.

### Construct gene–gene graph and gene pathways

Given the set of cells under consideration in the current iteration, the co-occurrence clustering algorithm first evaluates the co-occurrence between each pair of genes using the chi-square statistics. Based on the binarized data of two genes *g*_1_ and *g*_2_, denote *A* as the number of cells in which both genes are detected, *B* as the number of cells in which *g*_1_ is detected but *g*_2_ is undetected, *C* as the numbers of cells where *g*_1_ is undetected but *g*_2_ is detected, and *D* as the number of cells where both genes are undetected. The chi-square score is defined as $\frac{sign(AD \, - \, BC){(AD \, - \, BC)}^{2}}{(A \, + \, B)(A \, + \, C)(B \, + \, D)(C \, + \, D)}$. If the score is large, the two genes exhibit high co-occurrence. Random permutation is used to the define a threshold for what score is considered as large. Ten randomly permutated datasets are obtained by randomly reshuffling each row/gene independently, and the highest chi-square score from the random data is recorded. In the collection of pairwise chi-square scores from the actual data, for the ones smaller than the best random score, the mean and standard deviation are computed. A threshold is defined as the mean plus the standard deviation. An undirected unweighted gene–gene graph is then constructed by applying the threshold to the pair-wise chi-square scores. The Jaccard index^34^ is applied to filter the unweighted graph into a weighted graph, and the Louvain algorithm^35^ is applied to detect communities in the gene–gene graph, which are referred to as gene pathways here. A pre-defined pathway size threshold is used to discard gene pathways that are too small. If all the gene pathways produced by the community dection algorithm are smaller the size threshold, all of them are discarded, and the current iteration ends without dividing the cells under consideration into clusters. The default threshold for the minimum pathway size was 20 for all the datasets analyzed in this paper.

### Construct cell–cell graph and cell clusters

For each gene pathway generated from the gene–gene graph, the percentage of detected genes is computed for each cell. These percentages form a low-dimensional representation of the cells, where the dimensionality is the number of gene pathways, and each dimension describes the activity of one gene pathway in the cells. Using the pairwise Euclidean distance among the cells based on the pathway activity representation, a k-nearest neighbor graph is constructed, which is an undirected unweighted cell–cell graph. The Jaccard index is again applied to filter the unweighted graph into a weighted graph, and the Louvain algorithm is applied to detect communities in the cell–cell graph, which are referred to as cell clusters here. Cell clusters that are smaller than a pre-defined threshold are considered as tiny clusters, and are merged into the nearest non-tiny cluster. Here, “near” is defined by Euclidean distance based on the pathway activity representation. If all cell clusters are tiny, or only one cluster remains after the tiny clusters are merged, the current iteration ends without generating additional clusters. In all the datasets analyzed in this paper, the default k was 5 for the k-nearest neighbor graph, and the default threshold for tiny clusters was 10.

### Merge cell clusters

Although the cell–cell graph is defined based on the pathway percentages of detection, the cell clusters generated by community detection on the cell–cell graph are not necessarily prominently different in terms of the pathway percentages of detection. Therefore, the algorithm further merges the cell clusters according to three metrics of the percentages of detection: mean difference, mean ratio, signal-to-noisy ratio (SNR). For two cell clusters, the mean difference of a gene pathway is defined as the difference in the mean of the pathway’s percentage of detection in the two cell clusters; the mean ratio of the gene pathway is the ratio between the mean of the pathway’s percentage of detection in the two cell clusters; the SNR of the pathway is defined as the mean difference over the sum of the standard deviations of pathway’s the percentage of detection in the two cell clusters. In order for two cluster to be considered as prominently different, the algorithm requires two criteria to be met: (1) the maximum of their SNRs of the gene pathways is larger than 1.5, and (2) either the maximum of their mean differences for the gene pathways is larger than 0.5, or the maximum of their mean ratios is larger than 2. Clusters that do not meet these criteria are merged. After the clusters are merged according to these criteria, any two resulting cell clusters will exhibit prominent difference in at least one gene pathway, where prominent difference means that the SNR is larger than 1.5, and either the mean difference is larger than 0.5 or the mean ratio is larger than 2. If only one cluster remains due to these merging criteria, the current iteration ends without generating additional clusters. If otherwise, subsequent iterations of the algorithm will examine the resulting cell clusters separately, and see whether they can be further divided in to sub-clusters that are prominently different in terms of certain gene pathways. The values 1.5, 0.5 and 2 are chosen to ensure that all resulting cell clusters exhibit distinct dropout patterns, and the same values were used for all the datasets analyzed in this paper.

### Iterative application of co-occurrence clustering

Similar to all divisive hierarchical clustering methods, the co-occurrence clustering algorithm represents a divide-and-concur strategy. When examining the root node that contains many distinct cell types, the gene–gene co-occurrence and the gene pathways are dominated by the differences among the major groups of cell types, enabling the initial iteration to identify the major cell clusters. Each subsequent iteration focuses on a previously identified cell cluster, where the gene–gene co-occurrence leads to different gene pathways driven by the heterogeneity within the cell cluster, which provides the basis for identifying further cell clusters. Typically, the sizes of gene pathways gradually decrease as the iterations proceed, because the cell clusters examined by later iterations are less heterogeneous compared to earlier iterations, and less heterogeneity means less prominent differences among further subtypes that are manifested in fewer genes and smaller pathways. The key strength of this algorithm is that different iterations use different sets of genes and pathways to cluster the cells.

### Reporting summary

Further information on research design is available in the Nature Research Reporting Summary linked to this article.

## Supplementary information

Supplementary Information

Reporting Summary

## Data Availability

The Peripheral Blood Mononuclear Cells (PBMC) dataset was downloaded from 10X Genomics (https://s3-us-west-2.amazonaws.com/10x.files/samples/cell/pbmc3k/pbmc3k_filtered_gene_bc_matrices.tar.gz). The dataset on human prefrontal cortex was downloaded from GEO, with accession number GSE104276. The Tabula Muris was obtained from the easy-data Github repository provided in the collaborative computational tools for the Human Cell Atlas (https://github.com/czi-hca-comp-tools/easy-data/blob/master/datasets/tabula_muris.md).

## Code availability

Source code for the co-occurrence clustering algorithm implementation is available at https://github.com/pqiu/cooccurrence_clustering.
