# Supplementary material for: Embracing the dropouts in single-cell RNA-seq analysis
Source: Nat Commun. 2020 Mar 3;11:1169. doi: 10.1038/s41467-020-14976-9 (PMC7054558; doi:10.1038/s41467-020-14976-9)
Supplement: Supplementary file 1 — Supplementary Information [file 41467_2020_14976_MOESM1_ESM.pdf]

Embracing the dropouts in single-cell RNA-seq analysis

— Supplementary Figures and Notes —

Peng Qiu

Department of Biomedical Engineering

Georgia Institute of Technology and Emory University

Email: peng.qiu@bme.gatech.edu

Supplementary Figure 1: page 2

Supplementary Figure 2: page 3

Supplementary Figure 3: page 4

Supplementary Note 1: pages 5 - 13

Supplementary Note 2: pages 14 - 40

Supplementary Note 3: pages 41 - 130

Supplementary Note 4: pages 131 - 218

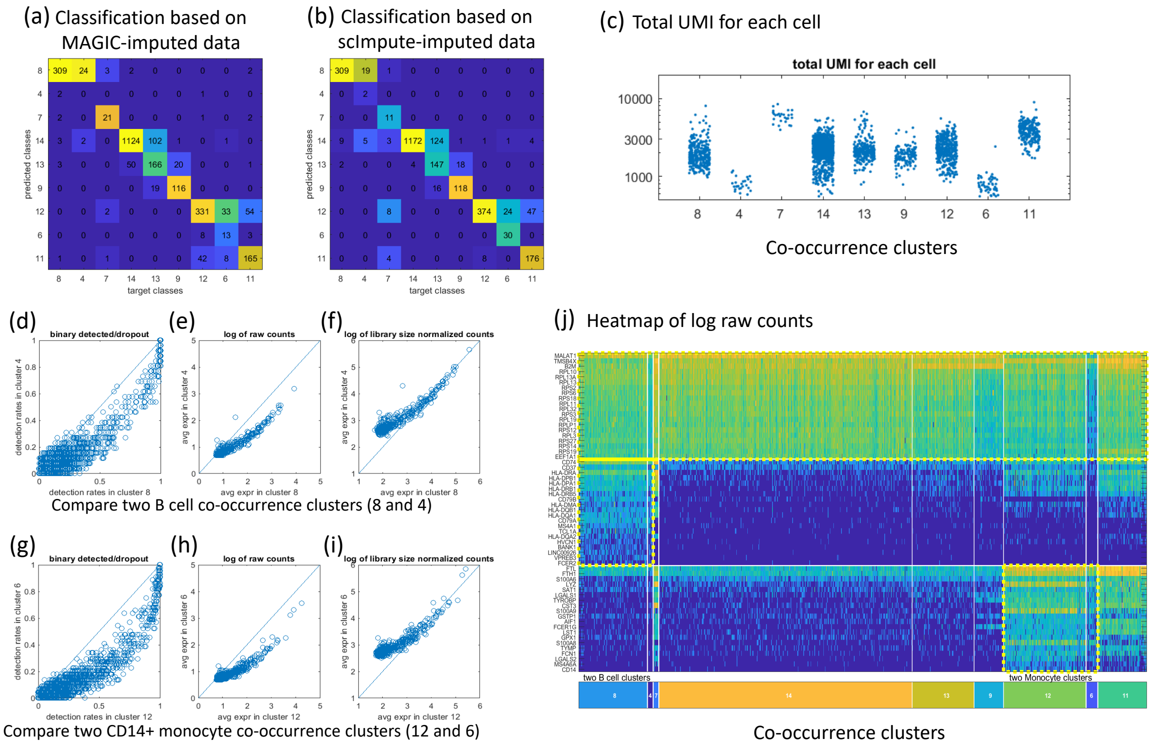

Supplementary Figure 1. Additional analysis of the PBMC dataset. (a-b) Random forest to classify co-occurrence clusters based on imputed PBMC data. We generated two imputed versions of the PBMC dataset using MAGIC (default parameters) and scImpute (default parameters except that number of clusters was set to be 9). Based on both versions of the data, we applied random forest with 5-fold cross-validation to examine whether the data can be used to classify the co-occurrence clusters. The resulting confusion tables shown here were similar to the classification analysis based on expression highly variable genes before imputation (Figure 2j), where clusters 4 and 6 were not accurately classified. This is mainly because both imputation algorithms use PCA as a dimension reduction step in their pipelines, and PCA in scRNA-seq data is often driven by highly variable genes. (c) We computed the total UMI counts for each cell, and showed that clusters 4 and 6 had significantly lower UMIs than their counterparts (clusters 8 and 12) in B cells and monocytes, and also lower than clusters corresponding to other cell types. (d) Comparison of gene detection rate between the two B cell clusters. Each dot is a gene; x-axis is the percentage of cells in cluster 8 that have the gene detected; y-axis the percentage of cells in cluster 4 that have the gene detected. (e-f) Comparison of average detected expression between the two B cell clusters, based on either log of raw UMI counts or log of library-size normalized data. (g-i) Comparisons of gene detection rate and average detected expression between the two CD14+ monocyte clusters. (j) Log of raw expression counts of highly expressed genes. The first 20 genes were highly expressed in all cell types. Most of these 20 genes were about ribosomal proteins. Their expression levels are relatively lower in clusters 4 and 6, consistent to the fact that cells in these two clusters have relatively low UMIs. The subsequent 40 genes were the most highly expressed genes in either B cells or monocytes, which contained gene markers for these two cell types (*CD79A*, *CD79B*, *MS4A1*, *CD14*, *LYZ*, etc). Their expression levels in clusters 4 and 6 were in par with their expression levels in clusters 8 and 12 which contained the majority of B cells and monocytes. Since cells in clusters 4 and 6 showed expression levels of cell-type-specific marker genes similar to their counterpart clusters 8 and 12, although they have lower UMIs, it is likely that they are real clusters, rather than poor quality cells.

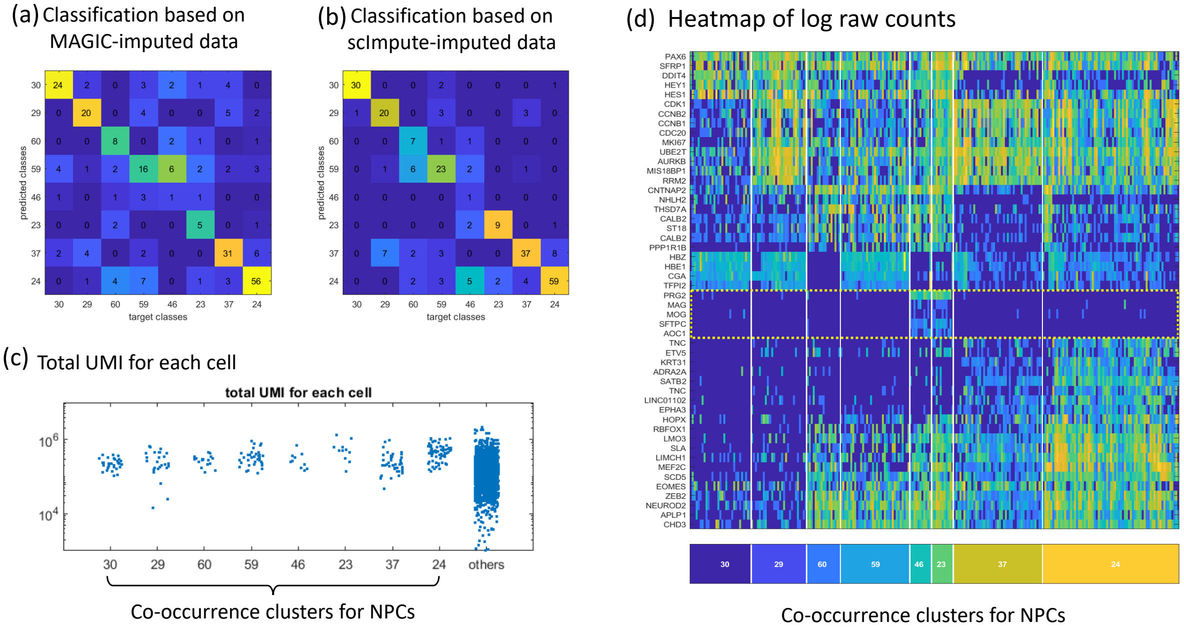

Supplementary Figure 2. Additional analysis of the NPCs in the prefrontal cortex dataset. (a-b) Random forest to classify co-occurrence clusters of NPCs based on imputed versions of the data. We generated two imputed versions of the data using MAGIC (default parameters) and scImpute (default parameters except that number of clusters was set to be 8). Based on both versions of the data, we applied random forest with 5-fold cross-validation to classify the 8 co-occurrence clusters of NPCs. The resulting confusion tables shown here were similar to the classification analysis based on expression highly variable genes before imputation (Figure 3e). The classification accuracy of clusters 23 was improved by scImpute, but cluster 46 was not accurately classified. These results suggested that imputation methods were unable to capture the two rare NPC clusters (23 and 46) identified by co-occurrence clustering. (c) We computed the total UMI counts for each cell, and showed that all the 8 co-occurrence cell clusters of NPCs have similar total UMI counts compared to other cell types. (d) Heatmap of log raw counts for selected highly variable genes. This heatmap visualized the gene expression differences among the co-occurrence cell clusters of NPCs. The highlighted section of the heatmap showed genes specific to the two rare NPC clusters (23 and 46), which exhibited higher expression of *PRG2, MAG, MOG, SFTPC, AOC1* compared to other NPC clusters.

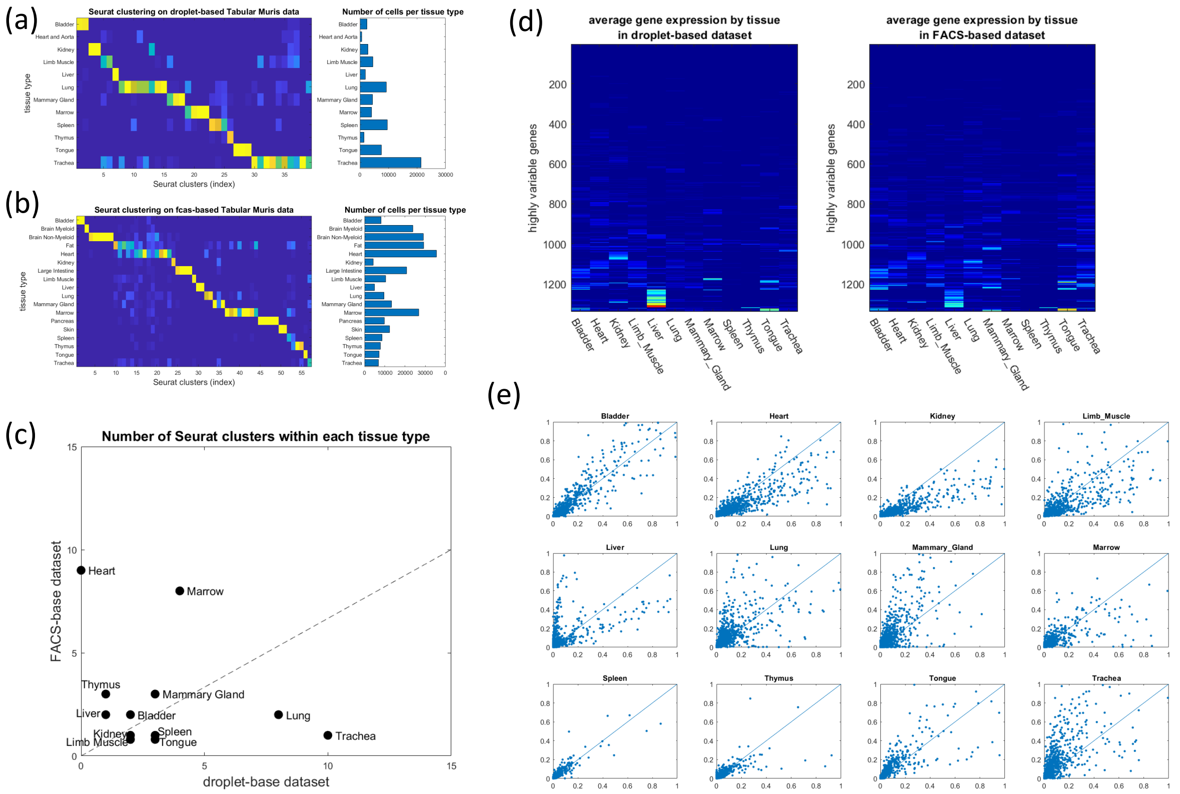

Supplementary Figure 3. Analysis of the two Tabula Muris datasets based on highly variable genes. (a-b) Seurat was applied to perform global clustering of all cells in the droplet-based and facs-based datasets separately. As shown in heatmaps that visualized the agreement between Seurat clusters an known tissue types, the expression of highly variable genes were able to delineate tissue types, similar to the results of co-occurrence clustering of dropouts. (c) For the 12 overlapping tissue types, the numbers of subpopulations identified by Seurat in the two datasets showed less correlation compared to co-occurrence clustering results in Figure 5a. This was in part because Seurat identified fewer cell clusters compared to co-occurrence cluster. (d) Similarity of the two heatmap showed that the average expression profiles of the highly variable genes in the 12 tissue types were highly correlated in the two datasets. (e) However, upon closer inspection of the 12 tissue types separately, the correlation between the two datasets based on highly variable genes is not as high as the correlation based on dropouts and pathway activities shown in Figure 5c. Overall, this analysis showed that the dropout pattern was more consistent than expression of highly variable genes, when comparing the same biology profiled by two scRNA-seq technologies.

— Supplementary Note 1 —

Contents

- addpath to all tools
- initiate one instance of the "cooccurrence_clustering_analysis" class
- read data - 10X format
- filter the data by removing genes and cells (same as Seurat tutorial on this data)
- binarize data
- cooccurrence clustering
- number of cooccurrence clusters

addpath to all tools

```
addpath(genpath('..\tools\'))
```

initiate one instance of the "cooccurrence_clustering_analysis" class

```
pbmc = cooccurrence_clustering_analysis;
```

read data - 10X format

```
pbmc = pbmc.Read10X('Downloads/filtered_gene_bc_matrices/hg19/');
```

```
Loading cell names ... Elapsed time is 0.004469 seconds.
Loading gene names ... Elapsed time is 0.102429 seconds.
Loading raw data ... Elapsed time is 1.091479 seconds.
Converting data of 32738 genes * 2700 cells into matrix ... Elapsed time is 0.176290 seconds.
```

filter the data by removing genes and cells (same as Seurat tutorial on this data)

```
pbmc.initial_filtering_min_num_cells = 3;
pbmc.initial_filtering_min_num_genes = 200;
pbmc.initial_filtering_max_num_genes = 2500;
pbmc.initial_filtering_max_percent_mito = 0.05;
pbmc = pbmc.initial_filtering_of_data(1);
```

```
Data after initial filtering 13714 genes * 2638 cells.
```

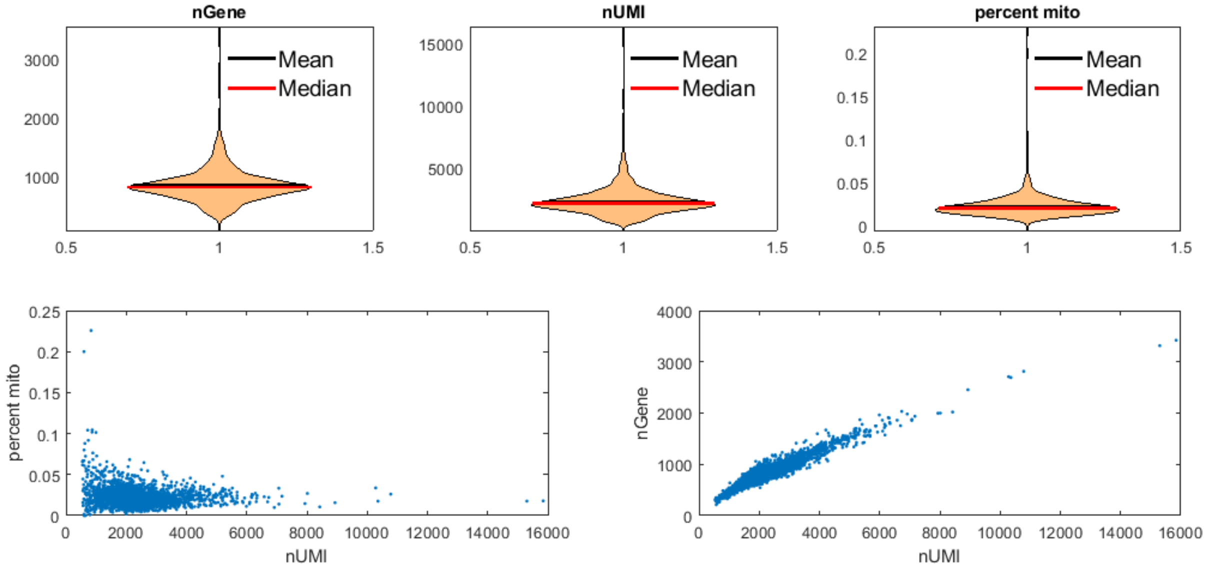

binarize data

```
pbmc.binarization_threshold = 0;
pbmc.binary_data = full(double(pbmc.data>pbmc.binarization_threshold));
```

cooccurrence clustering

```
pbmc.cooccurrence_min_expressed_cells = 10;             % genes will only be considered if detected in >= minimum number of cells, and undetected in >= minimum number of cells
pbmc.cooccurrence_min_pathway_size = 20;                % only considered gene clusters of size >= this threshold
pbmc.cooccurrence_min_population_size = 10;             % only considered gene clusters of size >= this threshold
pbmc.cooccurrence_snr_merge_threshold = 1.5;            % threshold for merging Louvain communities, based on snr of average detection of each gene cluster
pbmc.cooccurrence_mean_diff_merge_threshold = 0.5;      % threshold for merging Louvain communities
pbmc.cooccurrence_mean_ratio_merge_threshold = 2;       % threshold for merging Louvain communities

pbmc = pbmc.iterative_cooccurance_clustering;
```

```
Remaining clusters to partition 1
Processing cluster 0 now ...
Processing data subset with 13714 genes and 2638 cells:
Remove genes detected in <10 cells. Remaining 11085 genes.  Elapsed time is 0.177918 seconds.
Iterate 10 random permutations for gene-gene similarity threshold ...   10 Elapsed time is 132.845130 seconds.
Compute gene-gene similarity ... Elapsed time is 10.005371 seconds.
Create gene-gene graph for clustering genes ...
Writing graph into file ... 100%Elapsed time is 1.011491 seconds.
Running ModularityOptimizer for clustering ...Elapsed time is 1.230488 seconds.
Gene-gene graph contains 7 pathways, 1498 genes in total
Elapsed time is 1.698487 seconds.
Create cell-cell graph for clustering cells ...
Writing graph into file ... 100%Elapsed time is 0.330634 seconds.
Running ModularityOptimizer for clustering ...Elapsed time is 1.673793 seconds.
Cell-cell graph contains 15 cell types by community detection
Elapsed time is 1.733981 seconds.
Cell-cell graph contains 15 cell types after merging tiny cell clusters
Cell-cell graph contains 4 cell types after merging
Number of useful pathways is 4
```

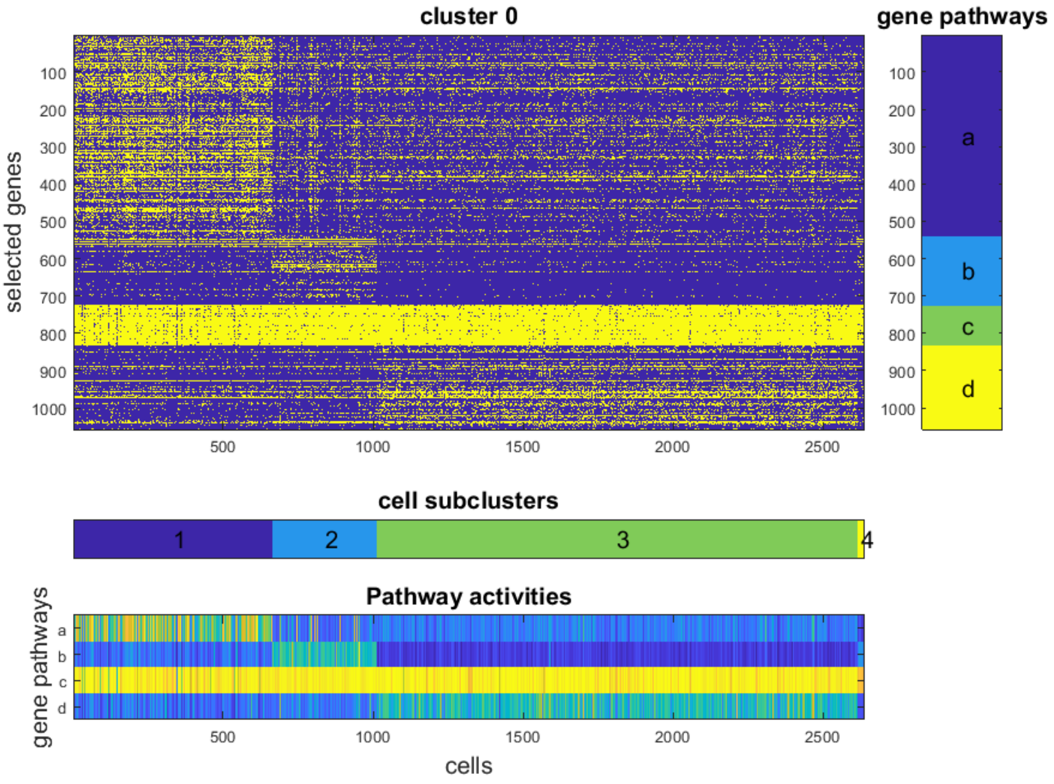


```
Remaining clusters to partition 4
Processing cluster 1 now ...
Processing data subset with 13714 genes and 665 cells:
Remove genes detected in <10 cells. Remaining 7676 genes.  Elapsed time is 0.046734 seconds.
Iterate 10 random permutations for gene-gene similarity threshold ...   10 Elapsed time is 43.234161 seconds.
Compute gene-gene similarity ... Elapsed time is 3.500961 seconds.
Create gene-gene graph for clustering genes ...
Writing graph into file ... 100%Elapsed time is 0.396271 seconds.
Running ModularityOptimizer for clustering ...Elapsed time is 0.643602 seconds.
Gene-gene graph contains 5 pathways, 492 genes in total
Elapsed time is 0.893363 seconds.
Create cell-cell graph for clustering cells ...
Writing graph into file ... 100%Elapsed time is 0.078460 seconds.
Running ModularityOptimizer for clustering ...Elapsed time is 0.498911 seconds.
Cell-cell graph contains 8 cell types by community detection
Elapsed time is 0.519466 seconds.
Cell-cell graph contains 8 cell types after merging tiny cell clusters
Cell-cell graph contains 2 cell types after merging
Number of useful pathways is 1
```

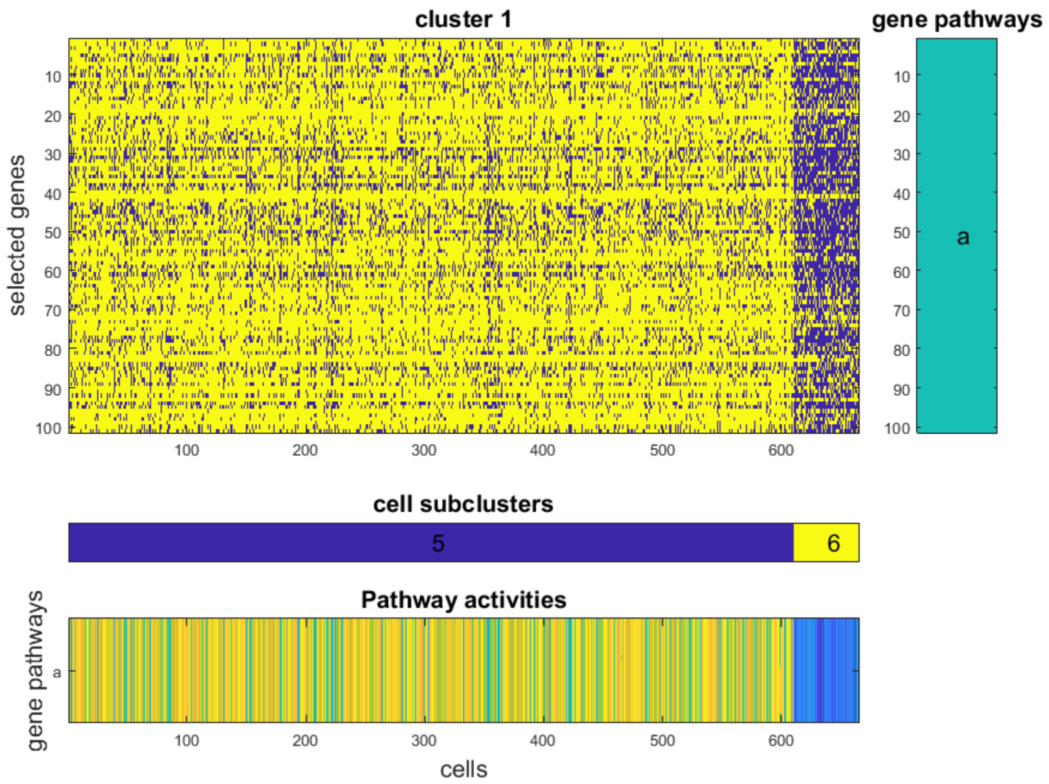


```
Remaining clusters to partition 5
Processing cluster 2 now ...
Processing data subset with 13714 genes and 347 cells:
Remove genes detected in <10 cells. Remaining 5592 genes.  Elapsed time is 0.022152 seconds.
Iterate 10 random permutations for gene-gene similarity threshold ...   10 Elapsed time is 21.886844 seconds.
Compute gene-gene similarity ... Elapsed time is 1.751440 seconds.
Create gene-gene graph for clustering genes ...
Writing graph into file ... 100%Elapsed time is 0.213308 seconds.
Running ModularityOptimizer for clustering ...Elapsed time is 0.660477 seconds.
Gene-gene graph contains 7 pathways, 650 genes in total
Elapsed time is 0.823525 seconds.
Create cell-cell graph for clustering cells ...
Writing graph into file ... 100%Elapsed time is 0.047000 seconds.
Running ModularityOptimizer for clustering ...Elapsed time is 0.345442 seconds.
Cell-cell graph contains 6 cell types by community detection
Elapsed time is 0.355403 seconds.
Cell-cell graph contains 6 cell types after merging tiny cell clusters
Cell-cell graph contains 2 cell types after merging
Number of useful pathways is 5
```

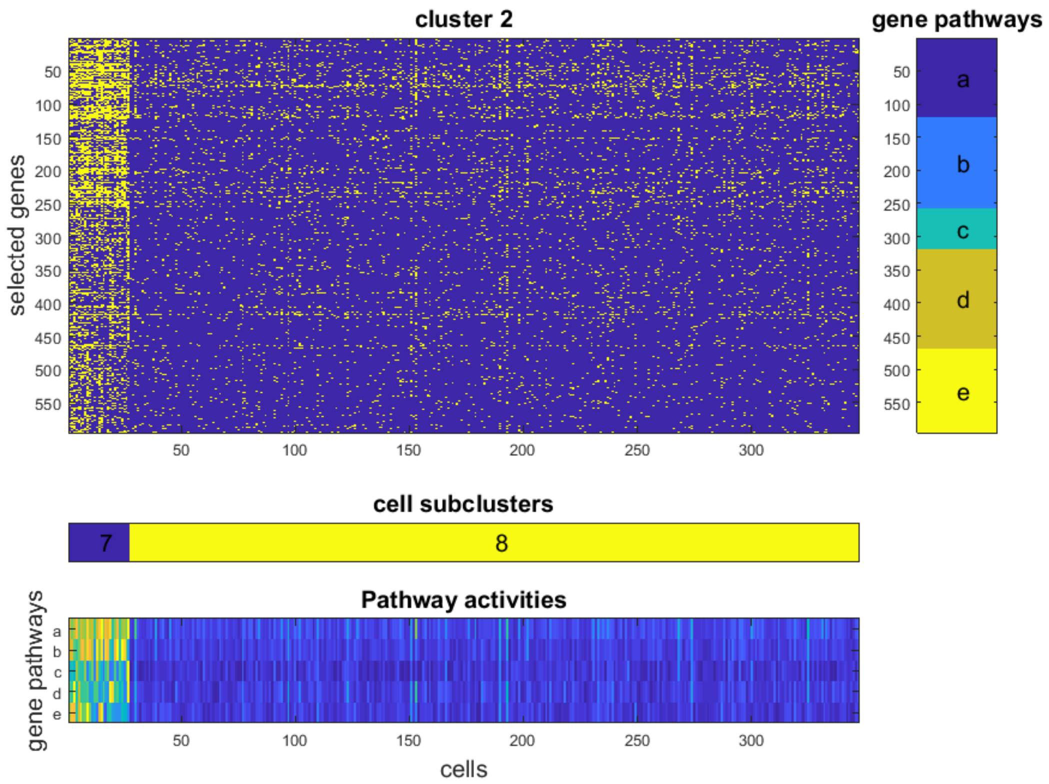


```
Remaining clusters to partition 6
Processing cluster 3 now ...
Processing data subset with 13714 genes and 1600 cells:
Remove genes detected in <10 cells. Remaining 9646 genes.  Elapsed time is 0.110096 seconds.
Iterate 10 random permutations for gene-gene similarity threshold ...   10 Elapsed time is 84.451726 seconds.
Compute gene-gene similarity ... Elapsed time is 6.556662 seconds.
Create gene-gene graph for clustering genes ...
Writing graph into file ... 100%Elapsed time is 0.592362 seconds.
Running ModularityOptimizer for clustering ...Elapsed time is 0.863222 seconds.
Gene-gene graph contains 6 pathways, 374 genes in total
Elapsed time is 1.191017 seconds.
Create cell-cell graph for clustering cells ...
Writing graph into file ... 100%Elapsed time is 0.201610 seconds.
Running ModularityOptimizer for clustering ...Elapsed time is 0.920632 seconds.
Cell-cell graph contains 10 cell types by community detection
Elapsed time is 0.957472 seconds.
Cell-cell graph contains 9 cell types after merging tiny cell clusters
creating a total of 8 edges ...      8
Cell-cell graph contains 2 cell types after merging
Number of useful pathways is 1
```

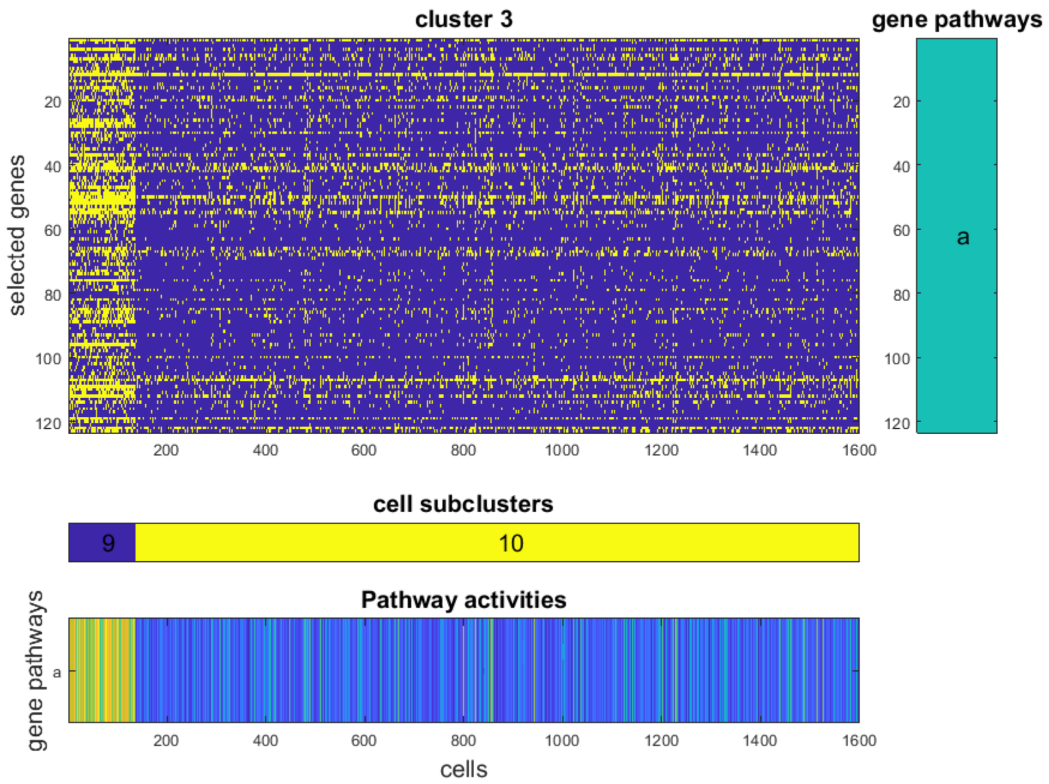


```
Remaining clusters to partition 7
Processing cluster 4 now ...
Processing data subset with 13714 genes and 26 cells:
Remove genes detected in <10 cells. Remaining 74 genes.  Elapsed time is 0.000995 seconds.
Iterate 10 random permutations for gene-gene similarity threshold ...   10 Elapsed time is 0.009967 seconds.
Compute gene-gene similarity ... Elapsed time is 0.000590 seconds.
Create gene-gene graph for clustering genes ...
Writing graph into file ... 111%Elapsed time is 0.001398 seconds.
Running ModularityOptimizer for clustering ...Elapsed time is 0.167751 seconds.
Gene-gene graph contains 0 pathways, 0 genes in total
Elapsed time is 0.172335 seconds.

Remaining clusters to partition 6
Processing cluster 5 now ...
Processing data subset with 13714 genes and 610 cells:
Remove genes detected in <10 cells. Remaining 7544 genes.  Elapsed time is 0.039289 seconds.
Iterate 10 random permutations for gene-gene similarity threshold ...   10 Elapsed time is 41.140392 seconds.
Compute gene-gene similarity ... Elapsed time is 3.329793 seconds.
Create gene-gene graph for clustering genes ...
Writing graph into file ... 100%Elapsed time is 0.358977 seconds.
Running ModularityOptimizer for clustering ...Elapsed time is 0.557954 seconds.
Gene-gene graph contains 3 pathways, 171 genes in total
Elapsed time is 0.806290 seconds.
Create cell-cell graph for clustering cells ...
Writing graph into file ... 100%Elapsed time is 0.070868 seconds.
Running ModularityOptimizer for clustering ...Elapsed time is 0.422437 seconds.
Cell-cell graph contains 10 cell types by community detection
Elapsed time is 0.437139 seconds.
Cell-cell graph contains 8 cell types after merging tiny cell clusters
creating a total of 7 edges ...      7
Cell-cell graph contains 2 cell types after merging
Number of useful pathways is 1
```

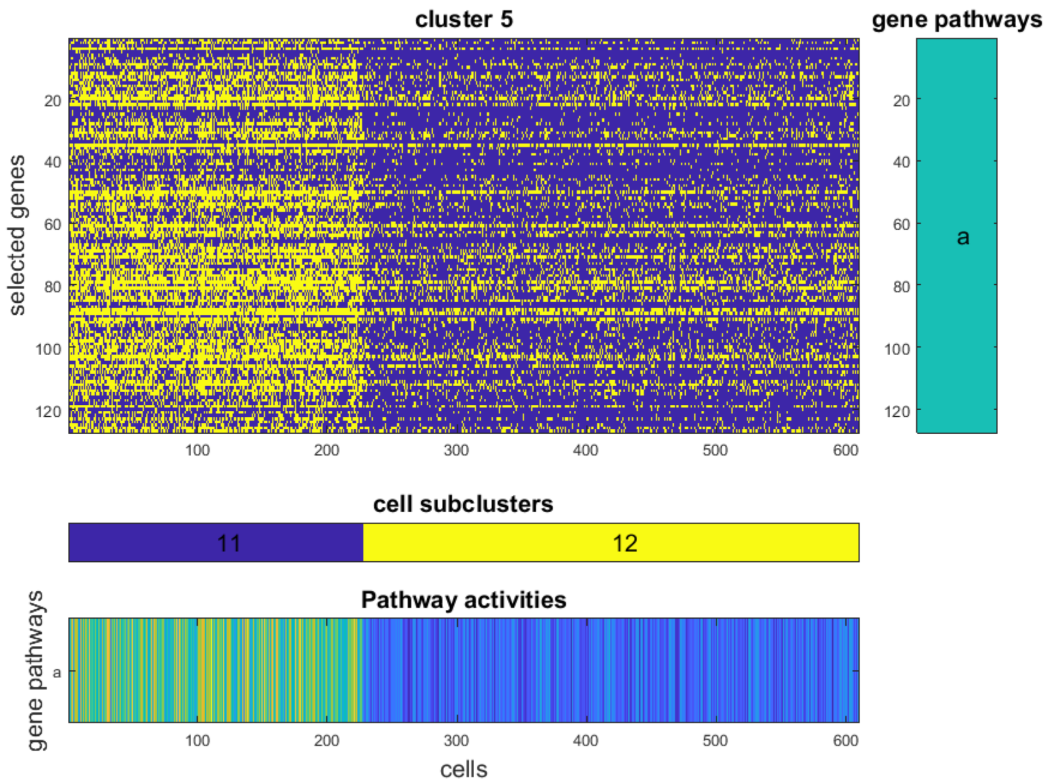


```
Remaining clusters to partition 7
Processing cluster 6 now ...
Processing data subset with 13714 genes and 55 cells:
Remove genes detected in <10 cells. Remaining 428 genes.  Elapsed time is 0.001773 seconds.
Iterate 10 random permutations for gene-gene similarity threshold ...   10 Elapsed time is 0.111292 seconds.
Compute gene-gene similarity ... Elapsed time is 0.009531 seconds.
Create gene-gene graph for clustering genes ...
Writing graph into file ... 100%Elapsed time is 0.009685 seconds.
Running ModularityOptimizer for clustering ...Elapsed time is 0.254108 seconds.
Gene-gene graph contains 2 pathways, 126 genes in total
Elapsed time is 0.266074 seconds.
Create cell-cell graph for clustering cells ...
Writing graph into file ... 100%Elapsed time is 0.007014 seconds.
Running ModularityOptimizer for clustering ...Elapsed time is 0.204163 seconds.
Cell-cell graph contains 3 cell types by community detection
Elapsed time is 0.207982 seconds.
Cell-cell graph contains 2 cell types after merging tiny cell clusters
creating a total of 1 edges ...      1
Cell-cell graph contains 1 cell types after merging

Remaining clusters to partition 6
Processing cluster 7 now ...
Processing data subset with 13714 genes and 27 cells:
Remove genes detected in <10 cells. Remaining 857 genes.  Elapsed time is 0.000847 seconds.
Iterate 10 random permutations for gene-gene similarity threshold ...   10 Elapsed time is 0.439079 seconds.
Compute gene-gene similarity ... Elapsed time is 0.038041 seconds.
Create gene-gene graph for clustering genes ...
Writing graph into file ... 101%Elapsed time is 0.006616 seconds.
Running ModularityOptimizer for clustering ...Elapsed time is 0.241725 seconds.
Gene-gene graph contains 0 pathways, 0 genes in total
Elapsed time is 0.262948 seconds.

Remaining clusters to partition 5
Processing cluster 8 now ...
Processing data subset with 13714 genes and 320 cells:
Remove genes detected in <10 cells. Remaining 4868 genes.  Elapsed time is 0.020069 seconds.
Iterate 10 random permutations for gene-gene similarity threshold ...   10 Elapsed time is 16.246315 seconds.
Compute gene-gene similarity ... Elapsed time is 1.329139 seconds.
Create gene-gene graph for clustering genes ...
Writing graph into file ... 100%Elapsed time is 0.150501 seconds.
Running ModularityOptimizer for clustering ...Elapsed time is 0.458427 seconds.
Gene-gene graph contains 1 pathways, 23 genes in total
Elapsed time is 0.597209 seconds.
Create cell-cell graph for clustering cells ...
Writing graph into file ... 100%Elapsed time is 0.046958 seconds.
Running ModularityOptimizer for clustering ...Elapsed time is 0.314946 seconds.
Cell-cell graph contains 8 cell types by community detection
Elapsed time is 0.324147 seconds.
Cell-cell graph contains 2 cell types after merging tiny cell clusters
creating a total of 1 edges ...      1
Cell-cell graph contains 1 cell types after merging

Remaining clusters to partition 4
Processing cluster 9 now ...
Processing data subset with 13714 genes and 137 cells:
Remove genes detected in <10 cells. Remaining 3058 genes.  Elapsed time is 0.007818 seconds.
```

```
Iterate 10 random permutations for gene-gene similarity threshold ...   10 Elapsed time is 6.022994 seconds.
Compute gene-gene similarity ... Elapsed time is 0.508862 seconds.
Create gene-gene graph for clustering genes ...
Writing graph into file ... 100%Elapsed time is 0.063383 seconds.
Running ModularityOptimizer for clustering ...Elapsed time is 0.378712 seconds.
Gene-gene graph contains 0 pathways, 0 genes in total
Elapsed time is 0.457484 seconds.

Remaining clusters to partition 3
Processing cluster 10 now ...
Processing data subset with 13714 genes and 1463 cells:
Remove genes detected in <10 cells. Remaining 9382 genes.  Elapsed time is 0.092382 seconds.
Iterate 10 random permutations for gene-gene similarity threshold ...   10 Elapsed time is 77.553015 seconds.
Compute gene-gene similarity ... Elapsed time is 6.118575 seconds.
Create gene-gene graph for clustering genes ...
Writing graph into file ... 100%Elapsed time is 0.545185 seconds.
Running ModularityOptimizer for clustering ...Elapsed time is 0.637086 seconds.
Gene-gene graph contains 3 pathways, 129 genes in total
Elapsed time is 0.959851 seconds.
Create cell-cell graph for clustering cells ...
Writing graph into file ... 100%Elapsed time is 0.169767 seconds.
Running ModularityOptimizer for clustering ...Elapsed time is 0.683454 seconds.
Cell-cell graph contains 19 cell types by community detection
Elapsed time is 0.716224 seconds.
Cell-cell graph contains 15 cell types after merging tiny cell clusters
Cell-cell graph contains 2 cell types after merging
Number of useful pathways is 1
```

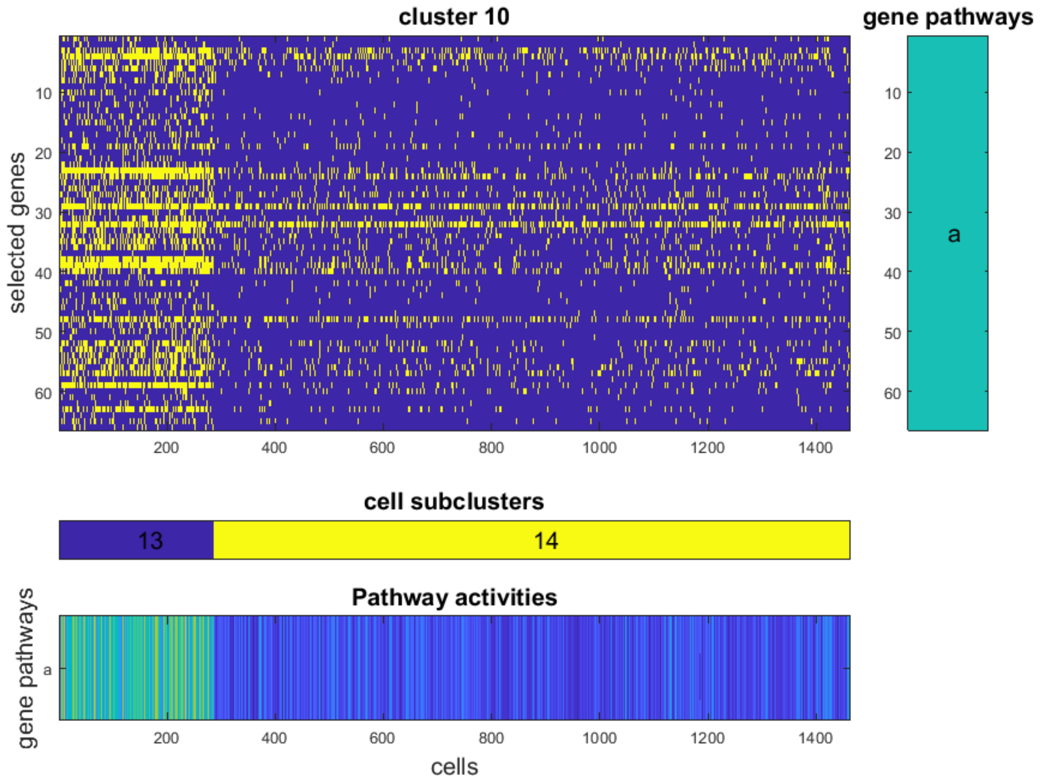


```
Remaining clusters to partition 4
Processing cluster 11 now ...
Processing data subset with 13714 genes and 227 cells:
Remove genes detected in <10 cells. Remaining 5324 genes.  Elapsed time is 0.013087 seconds.
Iterate 10 random permutations for gene-gene similarity threshold ...   10 Elapsed time is 18.750666 seconds.
Compute gene-gene similarity ... Elapsed time is 1.578370 seconds.
Create gene-gene graph for clustering genes ...
Writing graph into file ... 100%Elapsed time is 0.180144 seconds.
Running ModularityOptimizer for clustering ...Elapsed time is 0.457476 seconds.
Gene-gene graph contains 0 pathways, 0 genes in total
Elapsed time is 0.605987 seconds.

Remaining clusters to partition 3
Processing cluster 12 now ...
Processing data subset with 13714 genes and 383 cells:
Remove genes detected in <10 cells. Remaining 5401 genes.  Elapsed time is 0.023151 seconds.
Iterate 10 random permutations for gene-gene similarity threshold ...   10 Elapsed time is 20.759365 seconds.
Compute gene-gene similarity ... Elapsed time is 1.738366 seconds.
Create gene-gene graph for clustering genes ...
Writing graph into file ... 100%Elapsed time is 0.180759 seconds.
Running ModularityOptimizer for clustering ...Elapsed time is 0.469226 seconds.
Gene-gene graph contains 0 pathways, 0 genes in total
Elapsed time is 0.621008 seconds.

Remaining clusters to partition 2
Processing cluster 13 now ...
Processing data subset with 13714 genes and 287 cells:
```

```
Remove genes detected in <10 cells. Remaining 5149 genes.  Elapsed time is 0.018019 seconds.
Iterate 10 random permutations for gene-gene similarity threshold ...   10 Elapsed time is 17.962426 seconds.
Compute gene-gene similarity ... Elapsed time is 1.480242 seconds.
Create gene-gene graph for clustering genes ...
Writing graph into file ... 100%Elapsed time is 0.174401 seconds.
Running ModularityOptimizer for clustering ...Elapsed time is 0.458803 seconds.
Gene-gene graph contains 0 pathways, 0 genes in total
Elapsed time is 0.605164 seconds.

Remaining clusters to partition 1
Processing cluster 14 now ...
Processing data subset with 13714 genes and 1176 cells:
Remove genes detected in <10 cells. Remaining 8838 genes.  Elapsed time is 0.075163 seconds.
Iterate 10 random permutations for gene-gene similarity threshold ...   10 Elapsed time is 64.996407 seconds.
Compute gene-gene similarity ... Elapsed time is 5.196517 seconds.
Create gene-gene graph for clustering genes ...
Writing graph into file ... 100%Elapsed time is 0.485264 seconds.
Running ModularityOptimizer for clustering ...Elapsed time is 0.577508 seconds.
Gene-gene graph contains 1 pathways, 28 genes in total
Elapsed time is 0.867655 seconds.
Create cell-cell graph for clustering cells ...
Writing graph into file ... 100%Elapsed time is 0.187626 seconds.
Running ModularityOptimizer for clustering ...Elapsed time is 0.565569 seconds.
Cell-cell graph contains 15 cell types by community detection
Elapsed time is 0.593367 seconds.
Cell-cell graph contains 2 cell types after merging tiny cell clusters
creating a total of 1 edges ...      1
Cell-cell graph contains 1 cell types after merging
```

number of cooccurrence clusters

```
number_of_cooccurrence_clusters = length(unique(pbmc.cell_labels))
```

```
number_of_cooccurrence_clusters =

     9
```

Published with MATLAB® R2017b

— Supplementary Note 2 —

Contents

- addpath to all tools
- initiate one instance of the "cooccurrence_clustering_analysis" class
- read data prepawed in matlab file
- filter the data by removing genes and cells (same as Seurat tutorial on this data)
- binarize data
- cooccurrence clustering
- number of cooccurrence clusters

addpath to all tools

```
addpath(genpath('..\tools\'))
```

initiate one instance of the "cooccurrence_clustering_analysis" class

```
cooc = cooccurrence_clustering_analysis;
```

read data prepawed in matlab file

```
cooc = cooc.ReadMatlab('GSE104276_all_pfc_2394_UMI_count_NOERCC.mat');
```

filter the data by removing genes and cells (same as Seurat tutorial on this data)

```
cooc.initial_filtering_min_num_cells = 10;
cooc.initial_filtering_min_num_genes = 0;
cooc.initial_filtering_max_num_genes = Inf;
cooc.initial_filtering_max_percent_mito = 1;
cooc = cooc.initial_filtering_of_data(1);
```

```
Data after initial filtering 18021 genes * 2392 cells.
```

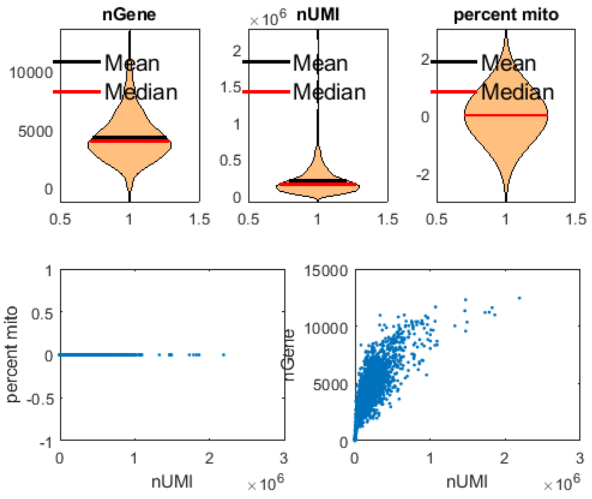

binarize data

```
cooc.binarization_threshold = 0;
cooc.binary_data = full(double(cooc.data>cooc.binarization_threshold));
```

cooccurrence clustering

```
cooc.cooccurrence_min_expressed_cells = 10;            % genes will only be considered if detected in >= minimum number of cells, and undetected in >= minimum number of cells
cooc.cooccurrence_min_pathway_size = 20;               % only considered gene clusters of size >= this threshold
cooc.cooccurrence_min_population_size = 10;            % only considered gene clusters of size >= this threshold
cooc.cooccurrence_snr_merge_threshold = 1.5;           % threshold for merging Louvain communities, based on snr of average detection of each gene cluster
cooc.cooccurrence_mean_diff_merge_threshold = 0.5;     % threshold for merging Louvain communities
cooc.cooccurrence_mean_ratio_merge_threshold = 2;      % threshold for merging Louvain communities


cooc = cooc.iterative_cooccurance_clustering;
```

```
Remaining clusters to partition 1
Processing cluster 0 now ...
Processing data subset with 18021 genes and 2392 cells:
Remove genes detected in <10 cells. Remaining 18015 genes.  Elapsed time is 0.243058 seconds.
Iterate 10 random permutations for gene-gene similarity threshold ...   10 Elapsed time is 321.524395 seconds.
Compute gene-gene similarity ... Elapsed time is 23.854948 seconds.
Create gene-gene graph for clustering genes ...
Writing graph into file ... 100%Elapsed time is 9.169205 seconds.
Running ModularityOptimizer for clustering ...Elapsed time is 24.536095 seconds.
Gene-gene graph contains 8 pathways, 9083 genes in total
Elapsed time is 25.371694 seconds.
```

```
Create cell-cell graph for clustering cells ...
Writing graph into file ... 100%Elapsed time is 0.276242 seconds.
Running ModularityOptimizer for clustering ...Elapsed time is 1.331412 seconds.
Cell-cell graph contains 13 cell types by community detection
Elapsed time is 1.385998 seconds.
Cell-cell graph contains 12 cell types after merging tiny cell clusters
Cell-cell graph contains 6 cell types after merging
Number of useful pathways is 6
```

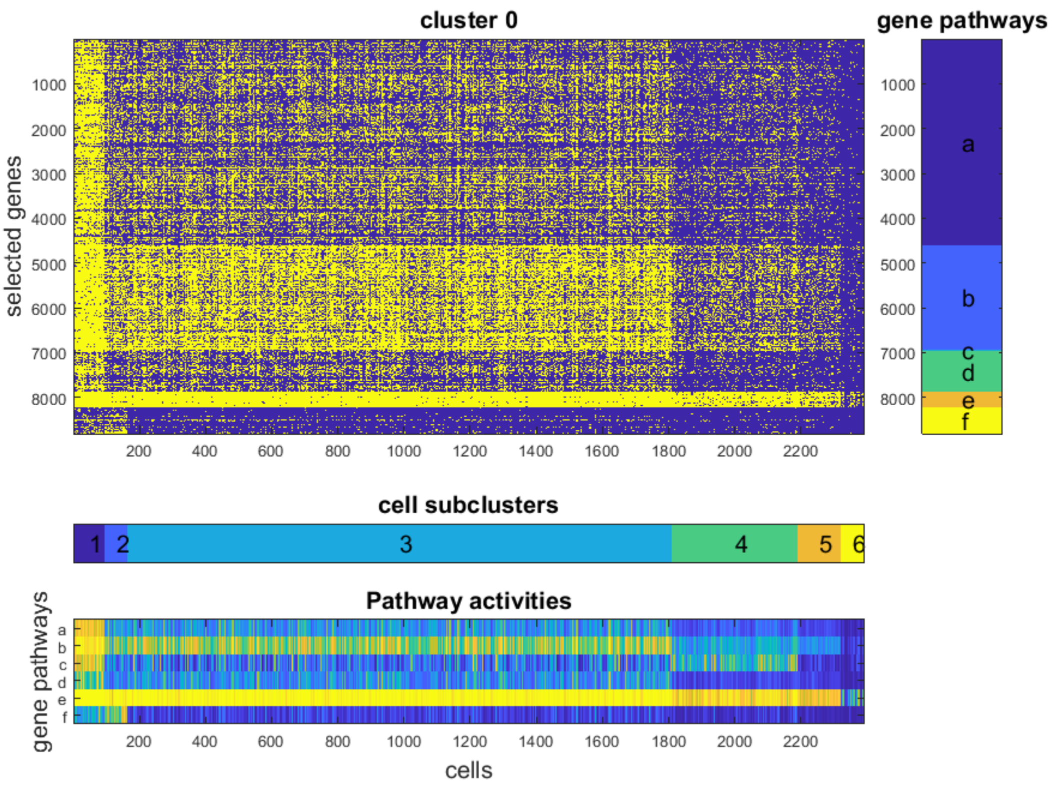


```
Remaining clusters to partition 6
Processing cluster 1 now ...
Processing data subset with 18021 genes and 96 cells:
Remove genes detected in <10 cells. Remaining 10447 genes.  Elapsed time is 0.008502 seconds.
Iterate 10 random permutations for gene-gene similarity threshold ...   10 Elapsed time is 68.697913 seconds.
Compute gene-gene similarity ... Elapsed time is 5.878206 seconds.
Create gene-gene graph for clustering genes ...
Writing graph into file ... 100%Elapsed time is 0.663341 seconds.
Running ModularityOptimizer for clustering ...Elapsed time is 0.776310 seconds.
Gene-gene graph contains 9 pathways, 493 genes in total
Elapsed time is 1.158954 seconds.
Create cell-cell graph for clustering cells ...
Writing graph into file ... 100%Elapsed time is 0.011576 seconds.
Running ModularityOptimizer for clustering ...Elapsed time is 0.242471 seconds.
Cell-cell graph contains 4 cell types by community detection
Elapsed time is 0.248874 seconds.
Cell-cell graph contains 3 cell types after merging tiny cell clusters
Cell-cell graph contains 2 cell types after merging
Number of useful pathways is 2
```

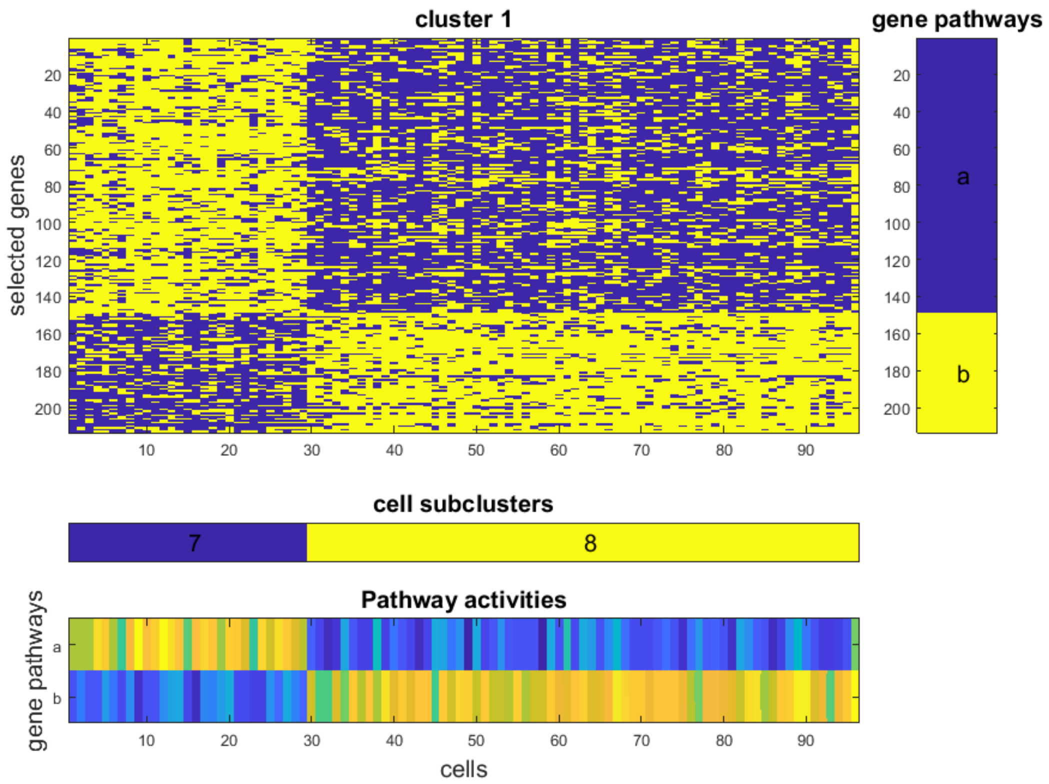


```
Remaining clusters to partition 7
Processing cluster 2 now ...
Processing data subset with 18021 genes and 68 cells:
Remove genes detected in <10 cells. Remaining 9671 genes.  Elapsed time is 0.006584 seconds.
Iterate 10 random permutations for gene-gene similarity threshold ...   10 Elapsed time is 58.053960 seconds.
Compute gene-gene similarity ... Elapsed time is 4.928949 seconds.
Create gene-gene graph for clustering genes ...
Writing graph into file ... 100%Elapsed time is 0.583562 seconds.
Running ModularityOptimizer for clustering ...Elapsed time is 0.742590 seconds.
Gene-gene graph contains 10 pathways, 363 genes in total
Elapsed time is 1.074094 seconds.
Create cell-cell graph for clustering cells ...
Writing graph into file ... 100%Elapsed time is 0.008700 seconds.
Running ModularityOptimizer for clustering ...Elapsed time is 0.224785 seconds.
Cell-cell graph contains 3 cell types by community detection
Elapsed time is 0.228900 seconds.
Cell-cell graph contains 3 cell types after merging tiny cell clusters
creating a total of 2 edges ...      2
Cell-cell graph contains 2 cell types after merging
Number of useful pathways is 1
```

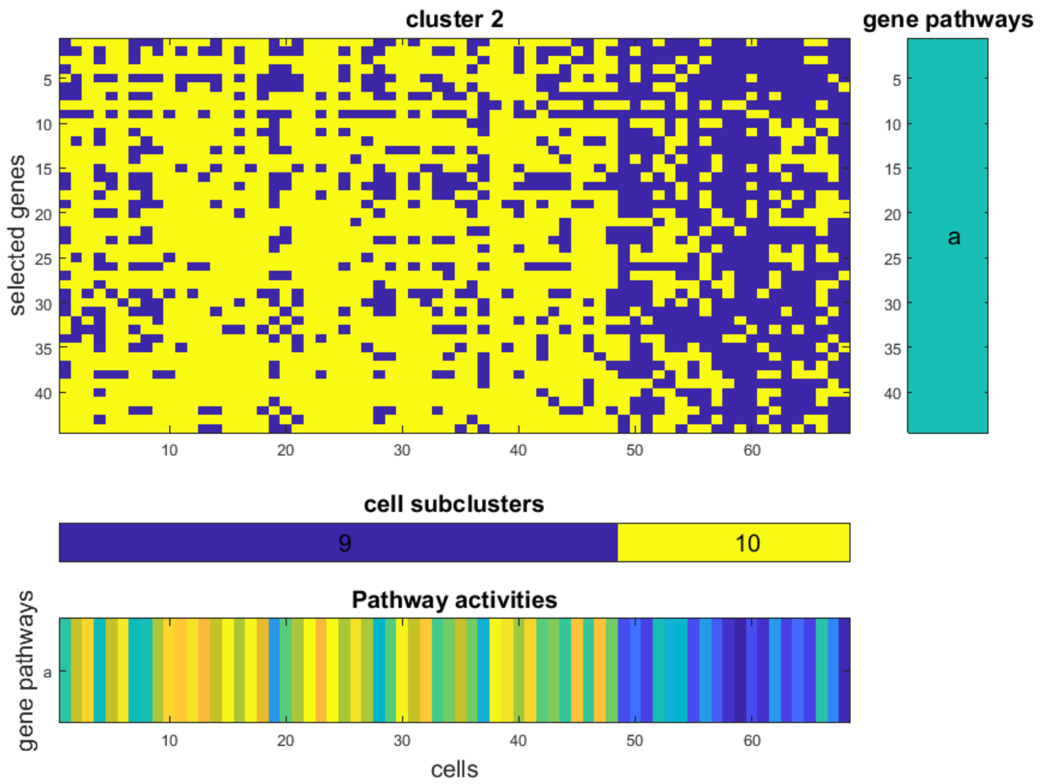


```
Remaining clusters to partition 8
Processing cluster 3 now ...
Processing data subset with 18021 genes and 1646 cells:
Remove genes detected in <10 cells. Remaining 17116 genes.  Elapsed time is 0.159200 seconds.
Iterate 10 random permutations for gene-gene similarity threshold ...   10 Elapsed time is 259.167072 seconds.
Compute gene-gene similarity ... Elapsed time is 19.745708 seconds.
Create gene-gene graph for clustering genes ...
Writing graph into file ... 100%Elapsed time is 2.095177 seconds.
Running ModularityOptimizer for clustering ...Elapsed time is 2.102157 seconds.
Gene-gene graph contains 10 pathways, 3380 genes in total
Elapsed time is 2.868386 seconds.
Create cell-cell graph for clustering cells ...
Writing graph into file ... 100%Elapsed time is 0.196254 seconds.
Running ModularityOptimizer for clustering ...Elapsed time is 1.114177 seconds.
Cell-cell graph contains 9 cell types by community detection
Elapsed time is 1.150228 seconds.
Cell-cell graph contains 8 cell types after merging tiny cell clusters
creating a total of 7 edges ...      7
Cell-cell graph contains 2 cell types after merging
Number of useful pathways is 1
```

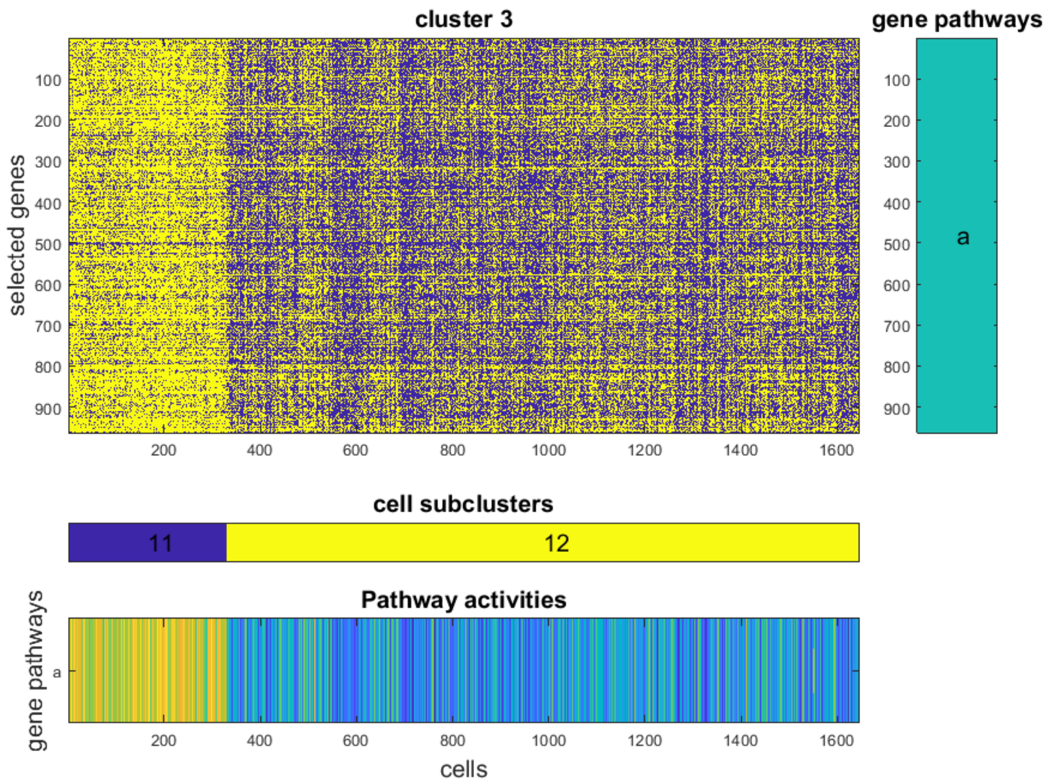


```
Remaining clusters to partition 9
Processing cluster 4 now ...
Processing data subset with 18021 genes and 379 cells:
Remove genes detected in <10 cells. Remaining 12130 genes.  Elapsed time is 0.034684 seconds.
Iterate 10 random permutations for gene-gene similarity threshold ...   10 Elapsed time is 100.348758 seconds.
Compute gene-gene similarity ... Elapsed time is 8.310954 seconds.
Create gene-gene graph for clustering genes ...
Writing graph into file ... 100%Elapsed time is 0.910249 seconds.
Running ModularityOptimizer for clustering ...Elapsed time is 0.802866 seconds.
Gene-gene graph contains 7 pathways, 278 genes in total
Elapsed time is 1.269783 seconds.
Create cell-cell graph for clustering cells ...
Writing graph into file ... 100%Elapsed time is 0.048113 seconds.
Running ModularityOptimizer for clustering ...Elapsed time is 0.389300 seconds.
Cell-cell graph contains 6 cell types by community detection
Elapsed time is 0.399780 seconds.
Cell-cell graph contains 5 cell types after merging tiny cell clusters
creating a total of 4 edges ...       4
Cell-cell graph contains 1 cell types after merging

Remaining clusters to partition 8
Processing cluster 5 now ...
Processing data subset with 18021 genes and 131 cells:
Remove genes detected in <10 cells. Remaining 7412 genes.  Elapsed time is 0.011022 seconds.
Iterate 10 random permutations for gene-gene similarity threshold ...   10 Elapsed time is 34.221664 seconds.
Compute gene-gene similarity ... Elapsed time is 2.970827 seconds.
Create gene-gene graph for clustering genes ...
Writing graph into file ... 100%Elapsed time is 0.346857 seconds.
Running ModularityOptimizer for clustering ...Elapsed time is 0.524786 seconds.
Gene-gene graph contains 2 pathways, 42 genes in total
Elapsed time is 0.759127 seconds.
Create cell-cell graph for clustering cells ...
Writing graph into file ... 100%Elapsed time is 0.017678 seconds.
Running ModularityOptimizer for clustering ...Elapsed time is 0.254560 seconds.
Cell-cell graph contains 5 cell types by community detection
Elapsed time is 0.259812 seconds.
Cell-cell graph contains 2 cell types after merging tiny cell clusters
creating a total of 1 edges ...       1
Cell-cell graph contains 1 cell types after merging

Remaining clusters to partition 7
Processing cluster 6 now ...
Processing data subset with 18021 genes and 72 cells:
Remove genes detected in <10 cells. Remaining 1165 genes.  Elapsed time is 0.004245 seconds.
Iterate 10 random permutations for gene-gene similarity threshold ...   10 Elapsed time is 0.880735 seconds.
Compute gene-gene similarity ... Elapsed time is 0.070913 seconds.
Create gene-gene graph for clustering genes ...
Writing graph into file ... 100%Elapsed time is 0.015342 seconds.
Running ModularityOptimizer for clustering ...Elapsed time is 0.327541 seconds.
Gene-gene graph contains 4 pathways, 161 genes in total
Elapsed time is 0.357526 seconds.
Create cell-cell graph for clustering cells ...
Writing graph into file ... 100%Elapsed time is 0.008715 seconds.
Running ModularityOptimizer for clustering ...Elapsed time is 0.202362 seconds.
Cell-cell graph contains 3 cell types by community detection
Elapsed time is 0.206341 seconds.
```

```
Cell-cell graph contains 3 cell types after merging tiny cell clusters
Cell-cell graph contains 2 cell types after merging
Number of useful pathways is 4
```

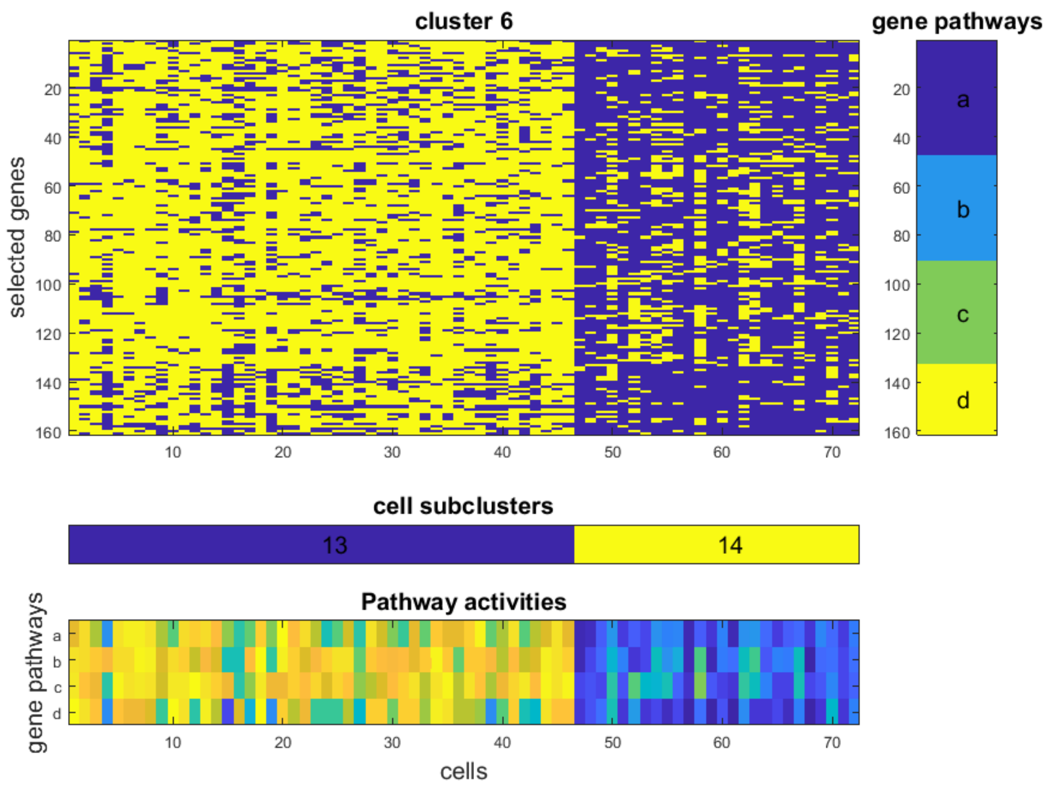


```
Remaining clusters to partition 8
Processing cluster 7 now ...
Processing data subset with 18021 genes and 29 cells:
Remove genes detected in <10 cells. Remaining 2881 genes.  Elapsed time is 0.001487 seconds.
Iterate 10 random permutations for gene-gene similarity threshold ...   10 Elapsed time is 4.943161 seconds.
Compute gene-gene similarity ... Elapsed time is 0.434104 seconds.
Create gene-gene graph for clustering genes ...
Writing graph into file ... 100%Elapsed time is 0.053506 seconds.
Running ModularityOptimizer for clustering ...Elapsed time is 0.341047 seconds.
Gene-gene graph contains 0 pathways, 0 genes in total
Elapsed time is 0.413511 seconds.

Remaining clusters to partition 7
Processing cluster 8 now ...
Processing data subset with 18021 genes and 67 cells:
Remove genes detected in <10 cells. Remaining 9001 genes.  Elapsed time is 0.006536 seconds.
Iterate 10 random permutations for gene-gene similarity threshold ...   10 Elapsed time is 49.871874 seconds.
Compute gene-gene similarity ... Elapsed time is 4.351614 seconds.
Create gene-gene graph for clustering genes ...
Writing graph into file ... 100%Elapsed time is 0.499146 seconds.
Running ModularityOptimizer for clustering ...Elapsed time is 0.590412 seconds.
Gene-gene graph contains 3 pathways, 69 genes in total
Elapsed time is 0.892547 seconds.
Create cell-cell graph for clustering cells ...
Writing graph into file ... 100%Elapsed time is 0.009089 seconds.
Running ModularityOptimizer for clustering ...Elapsed time is 0.218651 seconds.
Cell-cell graph contains 3 cell types by community detection
Elapsed time is 0.222674 seconds.
Cell-cell graph contains 3 cell types after merging tiny cell clusters
Cell-cell graph contains 3 cell types after merging
Number of useful pathways is 2
```

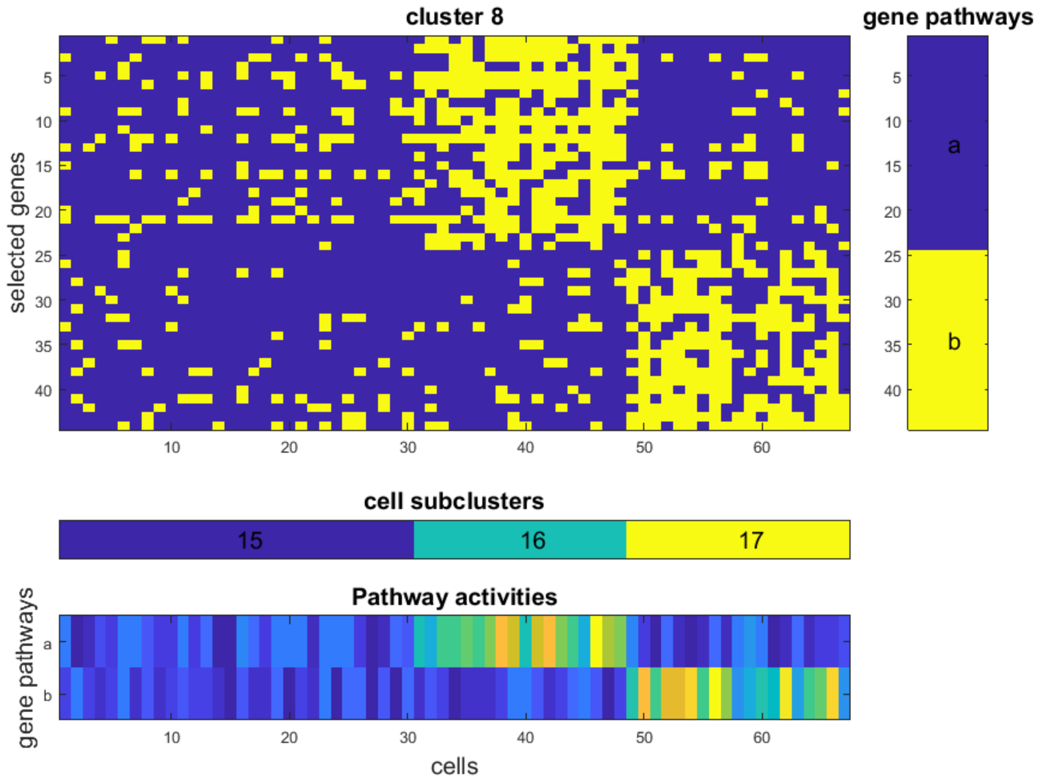


```
Remaining clusters to partition 9
Processing cluster 9 now ...
Processing data subset with 18021 genes and 48 cells:
Remove genes detected in <10 cells. Remaining 8183 genes.  Elapsed time is 0.004898 seconds.
Iterate 10 random permutations for gene-gene similarity threshold ...   10 Elapsed time is 41.028144 seconds.
Compute gene-gene similarity ... Elapsed time is 3.531587 seconds.
Create gene-gene graph for clustering genes ...
Writing graph into file ... 100%Elapsed time is 0.411269 seconds.
Running ModularityOptimizer for clustering ...Elapsed time is 0.574479 seconds.
Gene-gene graph contains 2 pathways, 62 genes in total
Elapsed time is 0.836957 seconds.
Create cell-cell graph for clustering cells ...
Writing graph into file ... 100%Elapsed time is 0.005718 seconds.
Running ModularityOptimizer for clustering ...Elapsed time is 0.201455 seconds.
Cell-cell graph contains 3 cell types by community detection
Elapsed time is 0.205147 seconds.
Cell-cell graph contains 2 cell types after merging tiny cell clusters
creating a total of 1 edges ...      1
Cell-cell graph contains 1 cell types after merging

Remaining clusters to partition 8
Processing cluster 10 now ...
Processing data subset with 18021 genes and 20 cells:
Remove genes detected in <10 cells. Remaining 339 genes.  Elapsed time is 0.000932 seconds.
Iterate 10 random permutations for gene-gene similarity threshold ...   10 Elapsed time is 0.046312 seconds.
Compute gene-gene similarity ... Elapsed time is 0.003663 seconds.
Create gene-gene graph for clustering genes ...
Writing graph into file ... 102%Elapsed time is 0.002390 seconds.
Running ModularityOptimizer for clustering ...Elapsed time is 0.201517 seconds.
Gene-gene graph contains 0 pathways, 0 genes in total
Elapsed time is 0.211181 seconds.

Remaining clusters to partition 7
Processing cluster 11 now ...
Processing data subset with 18021 genes and 331 cells:
Remove genes detected in <10 cells. Remaining 14553 genes.  Elapsed time is 0.030467 seconds.
Iterate 10 random permutations for gene-gene similarity threshold ...   10 Elapsed time is 144.329999 seconds.
Compute gene-gene similarity ... Elapsed time is 11.974912 seconds.
Create gene-gene graph for clustering genes ...
Writing graph into file ... 100%Elapsed time is 1.316260 seconds.
Running ModularityOptimizer for clustering ...Elapsed time is 1.448953 seconds.
Gene-gene graph contains 10 pathways, 1619 genes in total
Elapsed time is 2.048067 seconds.
Create cell-cell graph for clustering cells ...
Writing graph into file ... 100%Elapsed time is 0.039128 seconds.
Running ModularityOptimizer for clustering ...Elapsed time is 0.335600 seconds.
Cell-cell graph contains 6 cell types by community detection
Elapsed time is 0.345122 seconds.
Cell-cell graph contains 6 cell types after merging tiny cell clusters
Cell-cell graph contains 3 cell types after merging
Number of useful pathways is 3
```

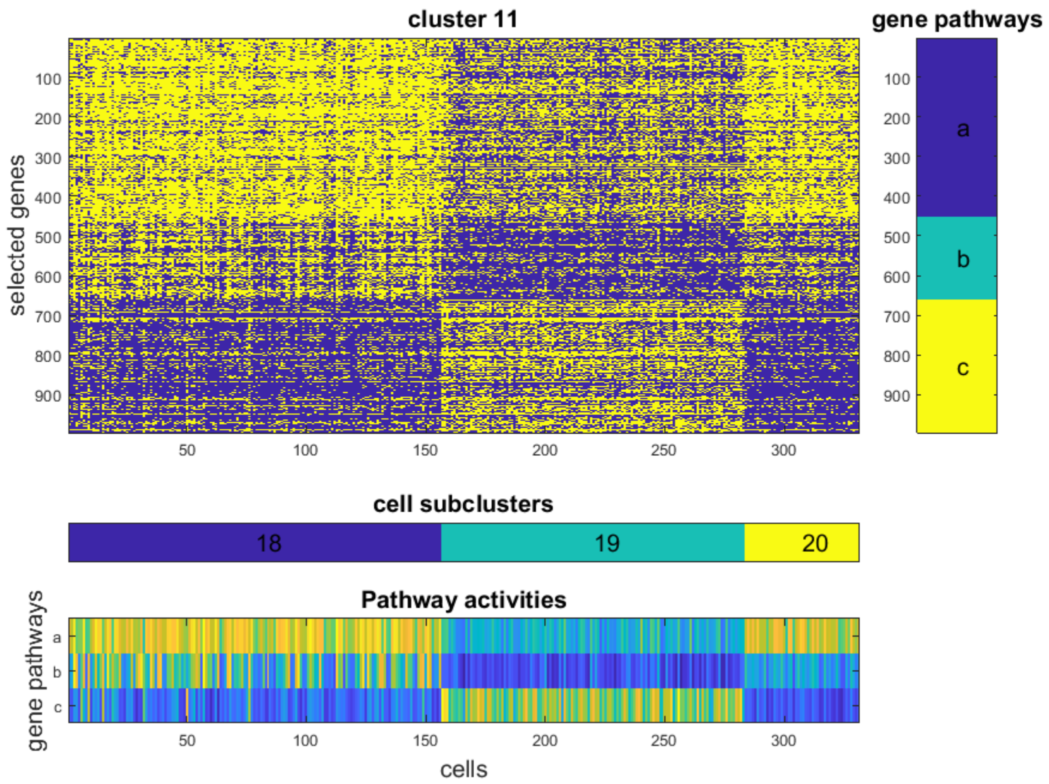


```
Remaining clusters to partition 9
Processing cluster 12 now ...
Processing data subset with 18021 genes and 1315 cells:
Remove genes detected in <10 cells. Remaining 16135 genes.  Elapsed time is 0.137980 seconds.
Iterate 10 random permutations for gene-gene similarity threshold ...   10 Elapsed time is 217.549020 seconds.
Compute gene-gene similarity ... Elapsed time is 17.165441 seconds.
Create gene-gene graph for clustering genes ...
Writing graph into file ... 100%Elapsed time is 1.667105 seconds.
Running ModularityOptimizer for clustering ...Elapsed time is 1.456496 seconds.
Gene-gene graph contains 9 pathways, 2280 genes in total
Elapsed time is 2.159626 seconds.
Create cell-cell graph for clustering cells ...
Writing graph into file ... 100%Elapsed time is 0.159840 seconds.
Running ModularityOptimizer for clustering ...Elapsed time is 0.815144 seconds.
Cell-cell graph contains 13 cell types by community detection
Elapsed time is 0.846197 seconds.
Cell-cell graph contains 12 cell types after merging tiny cell clusters
creating a total of 11 edges ...     11
Cell-cell graph contains 2 cell types after merging
Number of useful pathways is 1
```

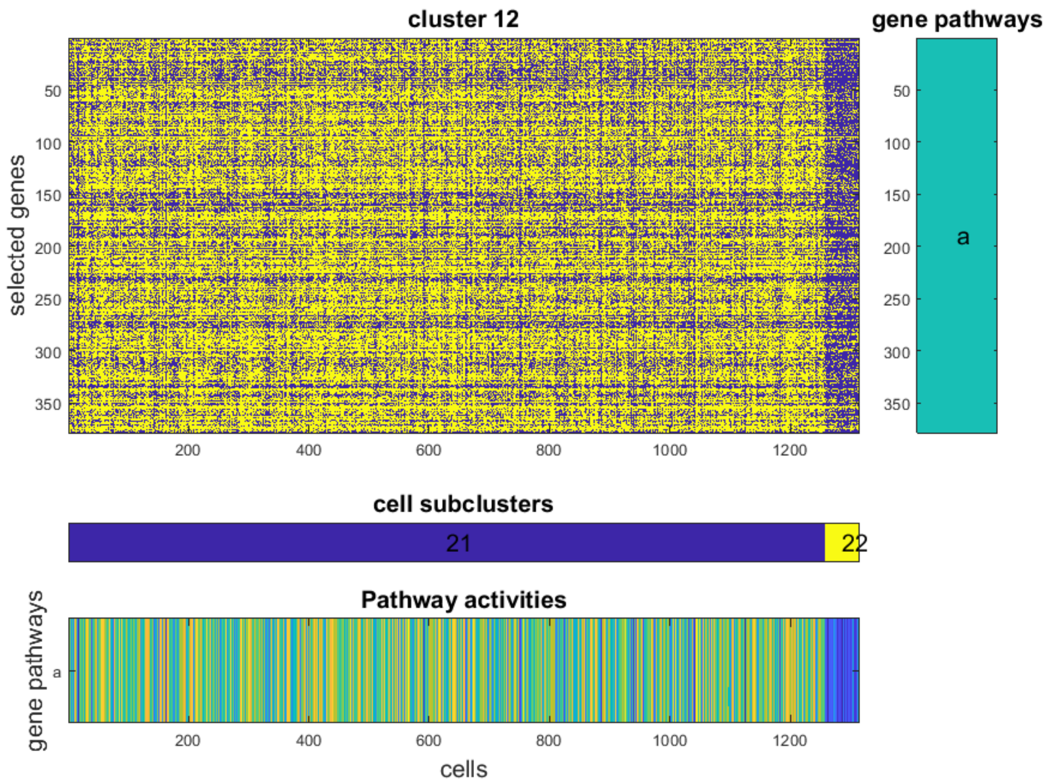


```
Remaining clusters to partition 10
Processing cluster 13 now ...
Processing data subset with 18021 genes and 46 cells:
Remove genes detected in <10 cells. Remaining 934 genes.  Elapsed time is 0.002815 seconds.
Iterate 10 random permutations for gene-gene similarity threshold ...   10 Elapsed time is 0.586661 seconds.
Compute gene-gene similarity ... Elapsed time is 0.051641 seconds.
Create gene-gene graph for clustering genes ...
Writing graph into file ... 101%Elapsed time is 0.007421 seconds.
Running ModularityOptimizer for clustering ...Elapsed time is 0.244001 seconds.
Gene-gene graph contains 0 pathways, 0 genes in total
Elapsed time is 0.266433 seconds.

Remaining clusters to partition 9
Processing cluster 14 now ...
Processing data subset with 18021 genes and 26 cells:
Remove genes detected in <10 cells. Remaining 27 genes.  Elapsed time is 0.001112 seconds.
Iterate 10 random permutations for gene-gene similarity threshold ...   10 Elapsed time is 0.004648 seconds.
Compute gene-gene similarity ... Elapsed time is 0.000283 seconds.
Create gene-gene graph for clustering genes ...
Writing graph into file ... 104%Elapsed time is 0.001498 seconds.
Running ModularityOptimizer for clustering ...Elapsed time is 0.166002 seconds.
Gene-gene graph contains 0 pathways, 0 genes in total
Elapsed time is 0.169277 seconds.

Remaining clusters to partition 8
Processing cluster 15 now ...
Processing data subset with 18021 genes and 30 cells:
Remove genes detected in <10 cells. Remaining 4197 genes.  Elapsed time is 0.001748 seconds.
Iterate 10 random permutations for gene-gene similarity threshold ...   10 Elapsed time is 10.511910 seconds.
Compute gene-gene similarity ... Elapsed time is 0.938416 seconds.
Create gene-gene graph for clustering genes ...
Writing graph into file ... 100%Elapsed time is 0.112736 seconds.
Running ModularityOptimizer for clustering ...Elapsed time is 0.405550 seconds.
Gene-gene graph contains 0 pathways, 0 genes in total
Elapsed time is 0.518831 seconds.

Remaining clusters to partition 7
Processing cluster 16 now ...
Processing data subset with 18021 genes and 18 cells:
Remove genes detected in <10 cells. Remaining 0 genes.  Elapsed time is 0.001028 seconds.

Remaining clusters to partition 6
Processing cluster 17 now ...
Processing data subset with 18021 genes and 19 cells:
Remove genes detected in <10 cells. Remaining 0 genes.  Elapsed time is 0.001148 seconds.

Remaining clusters to partition 5
Processing cluster 18 now ...
Processing data subset with 18021 genes and 156 cells:
Remove genes detected in <10 cells. Remaining 12227 genes.  Elapsed time is 0.015941 seconds.
Iterate 10 random permutations for gene-gene similarity threshold ...   10 Elapsed time is 95.944493 seconds.
Compute gene-gene similarity ... Elapsed time is 8.082140 seconds.
Create gene-gene graph for clustering genes ...
Writing graph into file ... 100%Elapsed time is 0.899405 seconds.
Running ModularityOptimizer for clustering ...Elapsed time is 0.929111 seconds.
Gene-gene graph contains 8 pathways, 488 genes in total
```

```
Elapsed time is 1.396202 seconds.
Create cell-cell graph for clustering cells ...
Writing graph into file ... 100%Elapsed time is 0.020133 seconds.
Running ModularityOptimizer for clustering ...Elapsed time is 0.278614 seconds.
Cell-cell graph contains 4 cell types by community detection
Elapsed time is 0.284513 seconds.
Cell-cell graph contains 4 cell types after merging tiny cell clusters
Cell-cell graph contains 4 cell types after merging
Number of useful pathways is 3
```

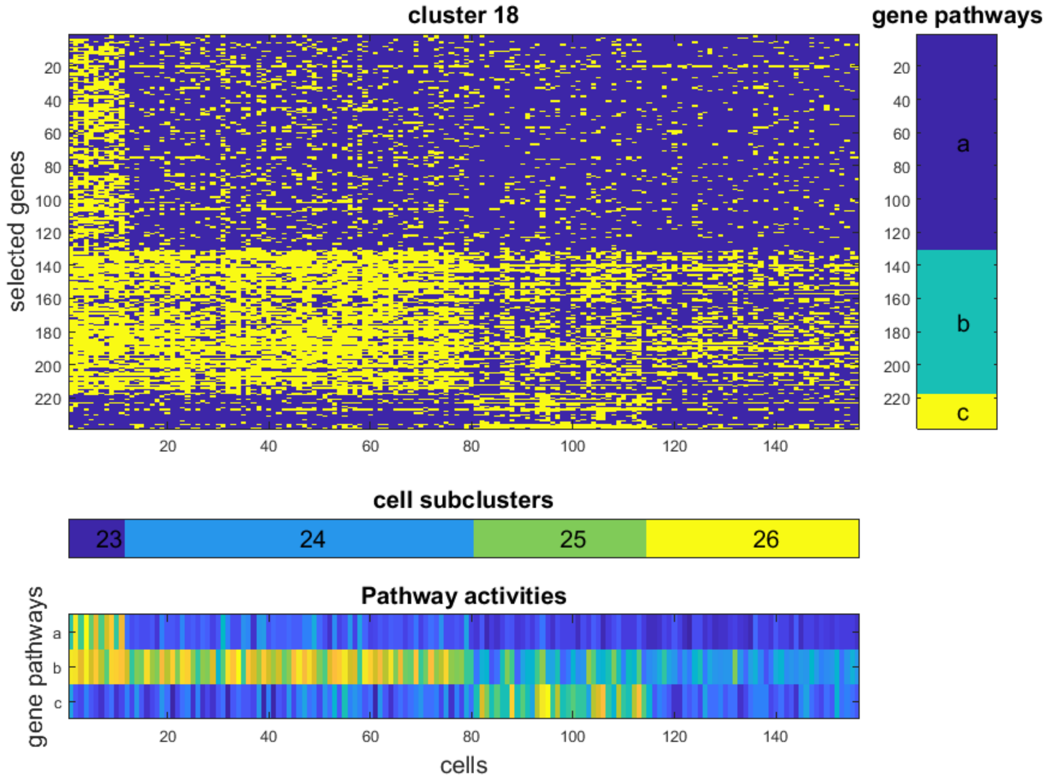


```
Remaining clusters to partition 8
Processing cluster 19 now ...
Processing data subset with 18021 genes and 127 cells:
Remove genes detected in <10 cells. Remaining 12061 genes.  Elapsed time is 0.012538 seconds.
Iterate 10 random permutations for gene-gene similarity threshold ...   10 Elapsed time is 93.609040 seconds.
Compute gene-gene similarity ... Elapsed time is 7.980962 seconds.
Create gene-gene graph for clustering genes ...
Writing graph into file ... 100%Elapsed time is 0.894633 seconds.
Running ModularityOptimizer for clustering ...Elapsed time is 0.780513 seconds.
Gene-gene graph contains 6 pathways, 203 genes in total
Elapsed time is 1.237179 seconds.
Create cell-cell graph for clustering cells ...
Writing graph into file ... 100%Elapsed time is 0.017243 seconds.
Running ModularityOptimizer for clustering ...Elapsed time is 0.264264 seconds.
Cell-cell graph contains 4 cell types by community detection
Elapsed time is 0.269656 seconds.
Cell-cell graph contains 4 cell types after merging tiny cell clusters
creating a total of 3 edges ...      3
Cell-cell graph contains 1 cell types after merging

Remaining clusters to partition 7
Processing cluster 20 now ...
Processing data subset with 18021 genes and 48 cells:
Remove genes detected in <10 cells. Remaining 7403 genes.  Elapsed time is 0.003431 seconds.
Iterate 10 random permutations for gene-gene similarity threshold ...   10 Elapsed time is 33.402398 seconds.
Compute gene-gene similarity ... Elapsed time is 2.903742 seconds.
Create gene-gene graph for clustering genes ...
Writing graph into file ... 100%Elapsed time is 0.344786 seconds.
Running ModularityOptimizer for clustering ...Elapsed time is 0.462271 seconds.
Gene-gene graph contains 0 pathways, 0 genes in total
Elapsed time is 0.692334 seconds.

Remaining clusters to partition 6
Processing cluster 21 now ...
Processing data subset with 18021 genes and 1256 cells:
Remove genes detected in <10 cells. Remaining 16053 genes.  Elapsed time is 0.128699 seconds.
Iterate 10 random permutations for gene-gene similarity threshold ...   10 Elapsed time is 211.936530 seconds.
Compute gene-gene similarity ... Elapsed time is 16.730927 seconds.
Create gene-gene graph for clustering genes ...
Writing graph into file ... 100%Elapsed time is 1.624333 seconds.
Running ModularityOptimizer for clustering ...Elapsed time is 1.671624 seconds.
Gene-gene graph contains 10 pathways, 2118 genes in total
Elapsed time is 2.373977 seconds.
Create cell-cell graph for clustering cells ...
Writing graph into file ... 100%Elapsed time is 0.158627 seconds.
```

```
Running ModularityOptimizer for clustering ...Elapsed time is 0.754566 seconds.
Cell-cell graph contains 13 cell types by community detection
Elapsed time is 0.782870 seconds.
Cell-cell graph contains 13 cell types after merging tiny cell clusters
creating a total of 12 edges ...     12
Cell-cell graph contains 2 cell types after merging
Number of useful pathways is 1
```

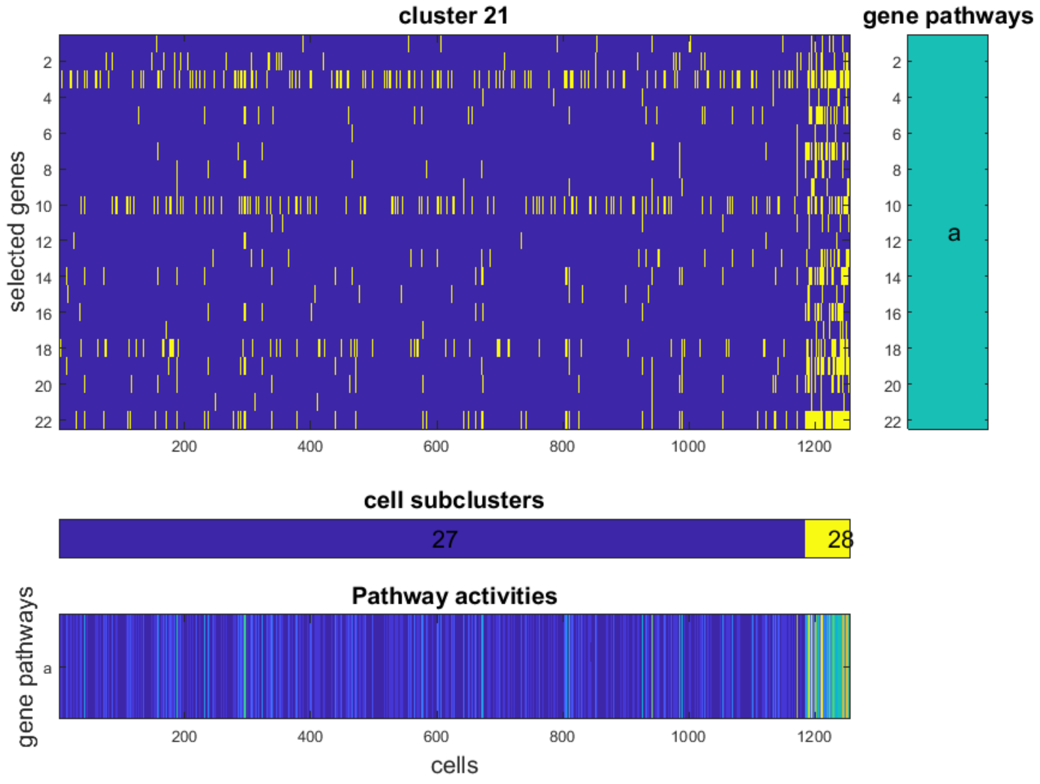


```
Remaining clusters to partition 7
Processing cluster 22 now ...
Processing data subset with 18021 genes and 59 cells:
Remove genes detected in <10 cells. Remaining 6814 genes.  Elapsed time is 0.004634 seconds.
Iterate 10 random permutations for gene-gene similarity threshold ...   10 Elapsed time is 28.960410 seconds.
Compute gene-gene similarity ... Elapsed time is 2.452898 seconds.
Create gene-gene graph for clustering genes ...
Writing graph into file ... 100%Elapsed time is 0.284451 seconds.
Running ModularityOptimizer for clustering ...Elapsed time is 0.580413 seconds.
Gene-gene graph contains 2 pathways, 108 genes in total
Elapsed time is 0.790546 seconds.
Create cell-cell graph for clustering cells ...
Writing graph into file ... 100%Elapsed time is 0.007651 seconds.
Running ModularityOptimizer for clustering ...Elapsed time is 0.201760 seconds.
Cell-cell graph contains 2 cell types by community detection
Elapsed time is 0.205604 seconds.
Cell-cell graph contains 2 cell types after merging tiny cell clusters
Cell-cell graph contains 2 cell types after merging
Number of useful pathways is 1
```

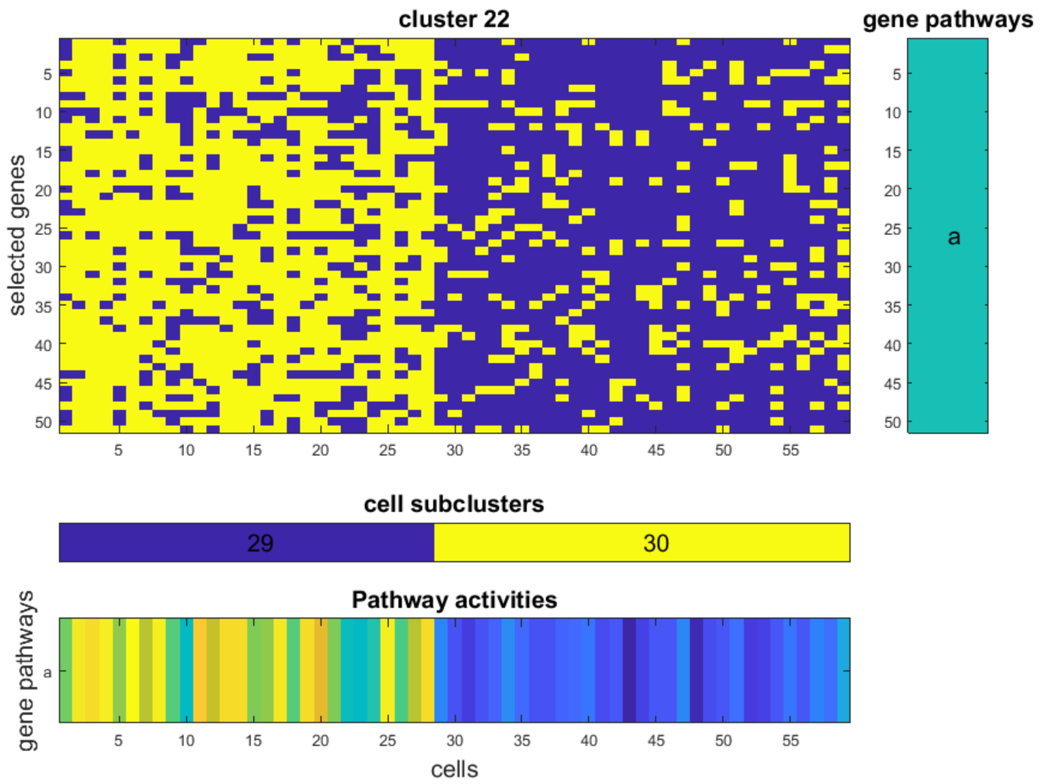


```
Remaining clusters to partition 8
Processing cluster 23 now ...
Processing data subset with 18021 genes and 11 cells:
Remove genes detected in <10 cells. Remaining 0 genes.  Elapsed time is 0.000728 seconds.

Remaining clusters to partition 7
Processing cluster 24 now ...
Processing data subset with 18021 genes and 69 cells:
Remove genes detected in <10 cells. Remaining 9345 genes.  Elapsed time is 0.006862 seconds.
Iterate 10 random permutations for gene-gene similarity threshold ...   10 Elapsed time is 54.841591 seconds.
Compute gene-gene similarity ... Elapsed time is 4.663146 seconds.
Create gene-gene graph for clustering genes ...
Writing graph into file ... 100%Elapsed time is 0.539970 seconds.
Running ModularityOptimizer for clustering ...Elapsed time is 0.634886 seconds.
Gene-gene graph contains 4 pathways, 102 genes in total
Elapsed time is 0.956276 seconds.
Create cell-cell graph for clustering cells ...
Writing graph into file ... 100%Elapsed time is 0.009244 seconds.
Running ModularityOptimizer for clustering ...Elapsed time is 0.216061 seconds.
Cell-cell graph contains 3 cell types by community detection
Elapsed time is 0.220000 seconds.
Cell-cell graph contains 3 cell types after merging tiny cell clusters
creating a total of 2 edges ...      2
Cell-cell graph contains 1 cell types after merging

Remaining clusters to partition 6
Processing cluster 25 now ...
Processing data subset with 18021 genes and 34 cells:
Remove genes detected in <10 cells. Remaining 5162 genes.  Elapsed time is 0.002209 seconds.
Iterate 10 random permutations for gene-gene similarity threshold ...   10 Elapsed time is 15.931459 seconds.
Compute gene-gene similarity ... Elapsed time is 1.392850 seconds.
Create gene-gene graph for clustering genes ...
Writing graph into file ... 100%Elapsed time is 0.164865 seconds.
Running ModularityOptimizer for clustering ...Elapsed time is 0.437610 seconds.
Gene-gene graph contains 0 pathways, 0 genes in total
Elapsed time is 0.590025 seconds.

Remaining clusters to partition 5
Processing cluster 26 now ...
Processing data subset with 18021 genes and 42 cells:
Remove genes detected in <10 cells. Remaining 6646 genes.  Elapsed time is 0.003544 seconds.
Iterate 10 random permutations for gene-gene similarity threshold ...   10 Elapsed time is 26.448302 seconds.
Compute gene-gene similarity ... Elapsed time is 2.356043 seconds.
Create gene-gene graph for clustering genes ...
Writing graph into file ... 100%Elapsed time is 0.276218 seconds.
Running ModularityOptimizer for clustering ...Elapsed time is 0.441062 seconds.
Gene-gene graph contains 0 pathways, 0 genes in total
Elapsed time is 0.646385 seconds.

Remaining clusters to partition 4
Processing cluster 27 now ...
Processing data subset with 18021 genes and 1184 cells:
Remove genes detected in <10 cells. Remaining 15951 genes.  Elapsed time is 0.120847 seconds.
Iterate 10 random permutations for gene-gene similarity threshold ...   10 Elapsed time is 205.895873 seconds.
Compute gene-gene similarity ... Elapsed time is 16.526223 seconds.
Create gene-gene graph for clustering genes ...
```

```
Writing graph into file ... 100%Elapsed time is 1.601474 seconds.
Running ModularityOptimizer for clustering ...Elapsed time is 1.452178 seconds.
Gene-gene graph contains 10 pathways, 2047 genes in total
Elapsed time is 2.155422 seconds.
Create cell-cell graph for clustering cells ...
Writing graph into file ... 100%Elapsed time is 0.145541 seconds.
Running ModularityOptimizer for clustering ...Elapsed time is 0.702176 seconds.
Cell-cell graph contains 13 cell types by community detection
Elapsed time is 0.728977 seconds.
Cell-cell graph contains 11 cell types after merging tiny cell clusters
Cell-cell graph contains 4 cell types after merging
Number of useful pathways is 3
```

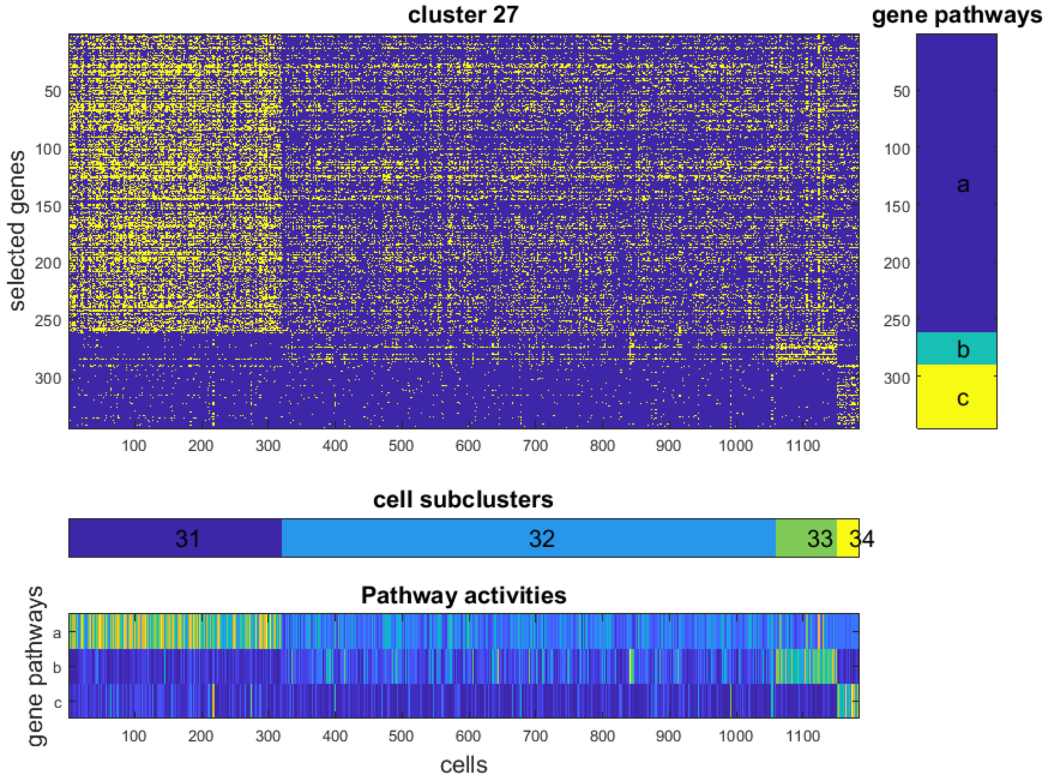


```
Remaining clusters to partition 7
Processing cluster 28 now ...
Processing data subset with 18021 genes and 72 cells:
Remove genes detected in <10 cells. Remaining 7479 genes.  Elapsed time is 0.005811 seconds.
Iterate 10 random permutations for gene-gene similarity threshold ...   10 Elapsed time is 34.504266 seconds.
Compute gene-gene similarity ... Elapsed time is 2.981751 seconds.
Create gene-gene graph for clustering genes ...
Writing graph into file ... 100%Elapsed time is 0.348647 seconds.
Running ModularityOptimizer for clustering ...Elapsed time is 0.521490 seconds.
Gene-gene graph contains 2 pathways, 60 genes in total
Elapsed time is 0.758946 seconds.
Create cell-cell graph for clustering cells ...
Writing graph into file ... 100%Elapsed time is 0.009717 seconds.
Running ModularityOptimizer for clustering ...Elapsed time is 0.220820 seconds.
Cell-cell graph contains 3 cell types by community detection
Elapsed time is 0.224852 seconds.
Cell-cell graph contains 3 cell types after merging tiny cell clusters
Cell-cell graph contains 2 cell types after merging
Number of useful pathways is 1
```

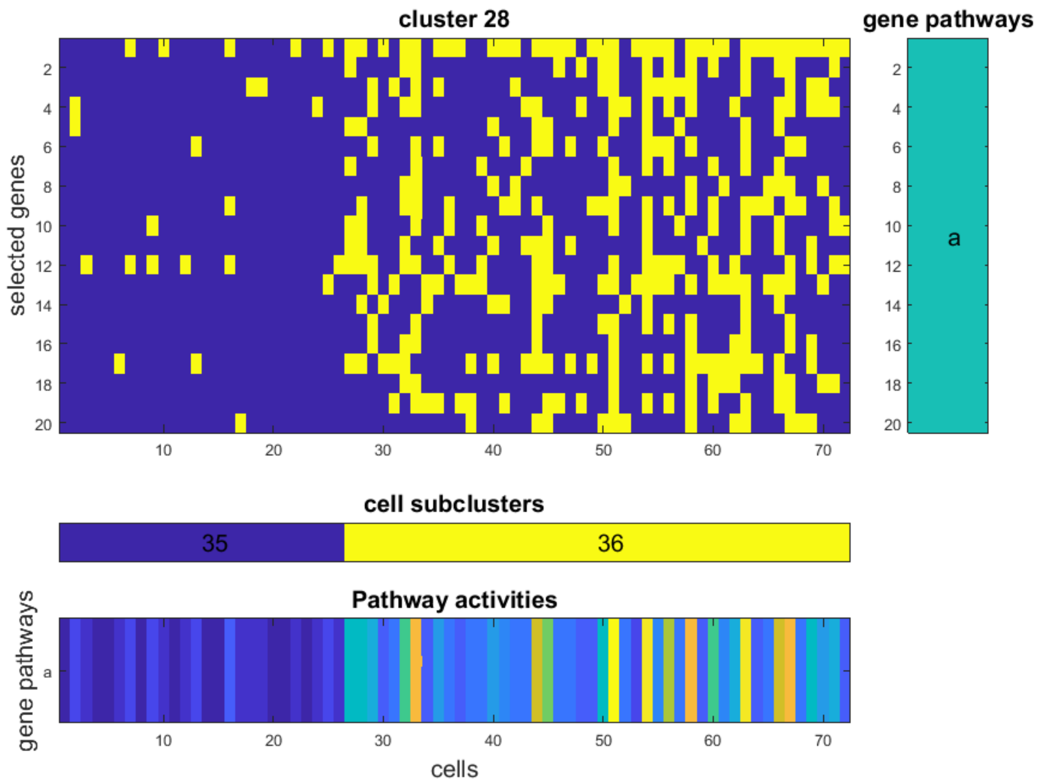


```
Remaining clusters to partition 8
Processing cluster 29 now ...
Processing data subset with 18021 genes and 28 cells:
Remove genes detected in <10 cells. Remaining 3062 genes.  Elapsed time is 0.001418 seconds.
Iterate 10 random permutations for gene-gene similarity threshold ...   10 Elapsed time is 5.564727 seconds.
Compute gene-gene similarity ... Elapsed time is 0.493687 seconds.
Create gene-gene graph for clustering genes ...
Writing graph into file ... 100%Elapsed time is 0.060709 seconds.
Running ModularityOptimizer for clustering ...Elapsed time is 0.352884 seconds.
Gene-gene graph contains 0 pathways, 0 genes in total
Elapsed time is 0.431514 seconds.

Remaining clusters to partition 7
Processing cluster 30 now ...
Processing data subset with 18021 genes and 31 cells:
Remove genes detected in <10 cells. Remaining 2836 genes.  Elapsed time is 0.002101 seconds.
Iterate 10 random permutations for gene-gene similarity threshold ...   10 Elapsed time is 4.750145 seconds.
Compute gene-gene similarity ... Elapsed time is 0.422677 seconds.
Create gene-gene graph for clustering genes ...
Writing graph into file ... 100%Elapsed time is 0.052243 seconds.
Running ModularityOptimizer for clustering ...Elapsed time is 0.345995 seconds.
Gene-gene graph contains 0 pathways, 0 genes in total
Elapsed time is 0.416943 seconds.

Remaining clusters to partition 6
Processing cluster 31 now ...
Processing data subset with 18021 genes and 320 cells:
Remove genes detected in <10 cells. Remaining 13595 genes.  Elapsed time is 0.028394 seconds.
Iterate 10 random permutations for gene-gene similarity threshold ...   10 Elapsed time is 125.723467 seconds.
Compute gene-gene similarity ... Elapsed time is 10.303662 seconds.
Create gene-gene graph for clustering genes ...
Writing graph into file ... 100%Elapsed time is 1.137370 seconds.
Running ModularityOptimizer for clustering ...Elapsed time is 1.014061 seconds.
Gene-gene graph contains 7 pathways, 947 genes in total
Elapsed time is 1.556221 seconds.
Create cell-cell graph for clustering cells ...
Writing graph into file ... 100%Elapsed time is 0.039857 seconds.
Running ModularityOptimizer for clustering ...Elapsed time is 0.356898 seconds.
Cell-cell graph contains 7 cell types by community detection
Elapsed time is 0.366438 seconds.
Cell-cell graph contains 7 cell types after merging tiny cell clusters
Cell-cell graph contains 5 cell types after merging
Number of useful pathways is 4
```

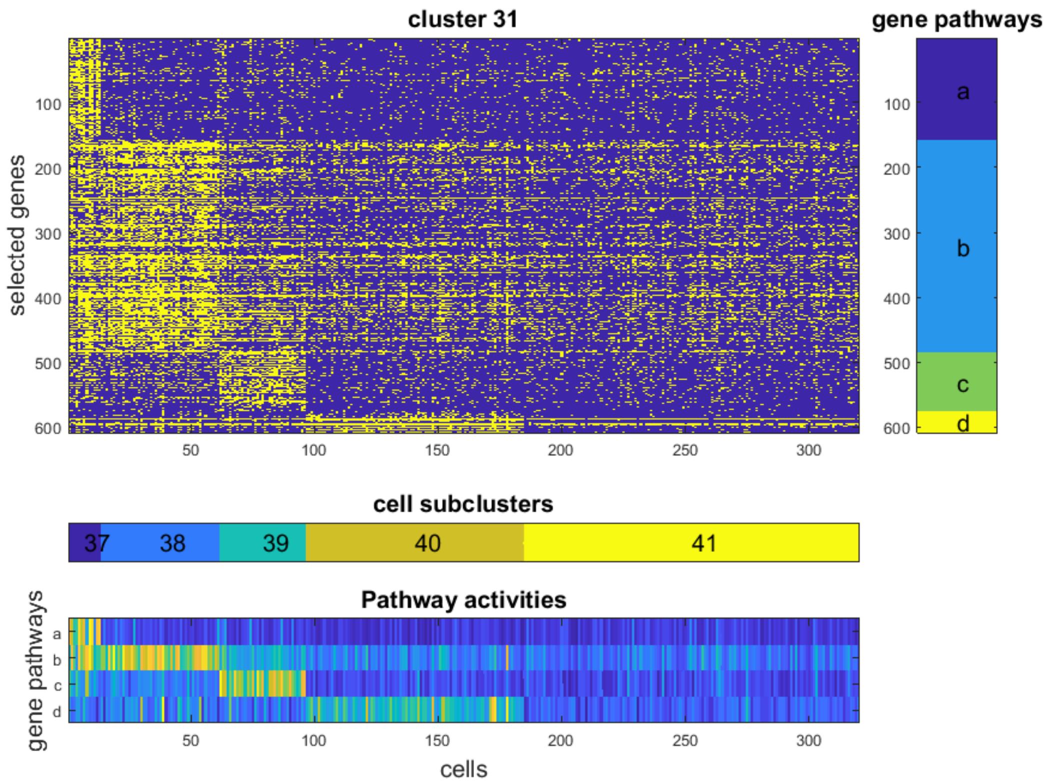


```
Remaining clusters to partition 10
Processing cluster 32 now ...
Processing data subset with 18021 genes and 739 cells:
Remove genes detected in <10 cells. Remaining 14631 genes.  Elapsed time is 0.078535 seconds.
Iterate 10 random permutations for gene-gene similarity threshold ...   10 Elapsed time is 159.070192 seconds.
Compute gene-gene similarity ... Elapsed time is 12.793224 seconds.
Create gene-gene graph for clustering genes ...
Writing graph into file ... 100%Elapsed time is 1.330317 seconds.
Running ModularityOptimizer for clustering ...Elapsed time is 1.234431 seconds.
Gene-gene graph contains 9 pathways, 1347 genes in total
Elapsed time is 1.839960 seconds.
Create cell-cell graph for clustering cells ...
Writing graph into file ... 100%Elapsed time is 0.088687 seconds.
Running ModularityOptimizer for clustering ...Elapsed time is 0.540664 seconds.
Cell-cell graph contains 9 cell types by community detection
Elapsed time is 0.558534 seconds.
Cell-cell graph contains 8 cell types after merging tiny cell clusters
creating a total of 7 edges ...      7
Cell-cell graph contains 2 cell types after merging
Number of useful pathways is 1
```

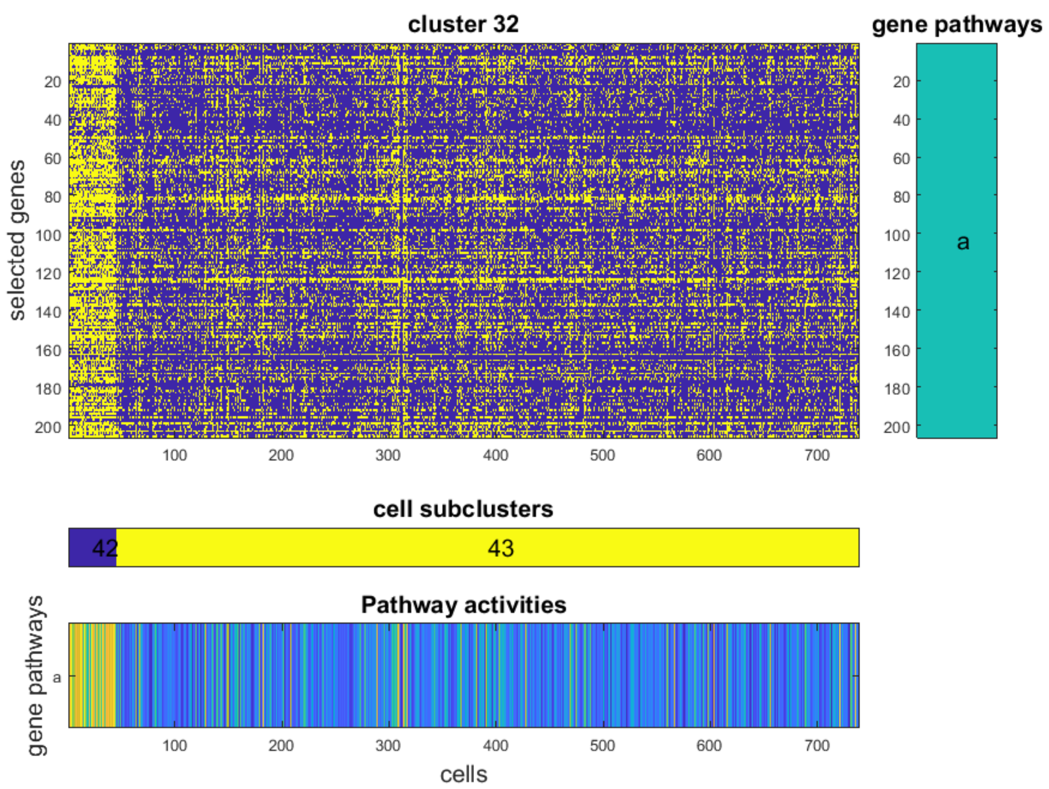


```
Remaining clusters to partition 11
Processing cluster 33 now ...
Processing data subset with 18021 genes and 92 cells:
Remove genes detected in <10 cells. Remaining 8777 genes.  Elapsed time is 0.008034 seconds.
Iterate 10 random permutations for gene-gene similarity threshold ...   10 Elapsed time is 47.970285 seconds.
Compute gene-gene similarity ... Elapsed time is 4.154517 seconds.
Create gene-gene graph for clustering genes ...
Writing graph into file ... 100%Elapsed time is 0.480750 seconds.
Running ModularityOptimizer for clustering ...Elapsed time is 0.669967 seconds.
Gene-gene graph contains 8 pathways, 339 genes in total
Elapsed time is 0.960268 seconds.
Create cell-cell graph for clustering cells ...
Writing graph into file ... 100%Elapsed time is 0.012445 seconds.
Running ModularityOptimizer for clustering ...Elapsed time is 0.240472 seconds.
Cell-cell graph contains 4 cell types by community detection
Elapsed time is 0.245035 seconds.
Cell-cell graph contains 4 cell types after merging tiny cell clusters
creating a total of 3 edges ...       3
Cell-cell graph contains 1 cell types after merging

Remaining clusters to partition 10
Processing cluster 34 now ...
Processing data subset with 18021 genes and 33 cells:
Remove genes detected in <10 cells. Remaining 3328 genes.  Elapsed time is 0.001782 seconds.
Iterate 10 random permutations for gene-gene similarity threshold ...   10 Elapsed time is 6.566239 seconds.
Compute gene-gene similarity ... Elapsed time is 0.605552 seconds.
Create gene-gene graph for clustering genes ...
Writing graph into file ... 100%Elapsed time is 0.071890 seconds.
Running ModularityOptimizer for clustering ...Elapsed time is 0.392100 seconds.
Gene-gene graph contains 0 pathways, 0 genes in total
Elapsed time is 0.477755 seconds.

Remaining clusters to partition 9
Processing cluster 35 now ...
Processing data subset with 18021 genes and 26 cells:
Remove genes detected in <10 cells. Remaining 1740 genes.  Elapsed time is 0.001523 seconds.
Iterate 10 random permutations for gene-gene similarity threshold ...   10 Elapsed time is 1.750528 seconds.
Compute gene-gene similarity ... Elapsed time is 0.158750 seconds.
Create gene-gene graph for clustering genes ...
Writing graph into file ... 101%Elapsed time is 0.022430 seconds.
Running ModularityOptimizer for clustering ...Elapsed time is 0.280544 seconds.
Gene-gene graph contains 0 pathways, 0 genes in total
Elapsed time is 0.323418 seconds.

Remaining clusters to partition 8
Processing cluster 36 now ...
Processing data subset with 18021 genes and 46 cells:
Remove genes detected in <10 cells. Remaining 5766 genes.  Elapsed time is 0.003679 seconds.
Iterate 10 random permutations for gene-gene similarity threshold ...   10 Elapsed time is 20.149431 seconds.
Compute gene-gene similarity ... Elapsed time is 1.752975 seconds.
Create gene-gene graph for clustering genes ...
Writing graph into file ... 100%Elapsed time is 0.209080 seconds.
Running ModularityOptimizer for clustering ...Elapsed time is 0.487278 seconds.
Gene-gene graph contains 0 pathways, 0 genes in total
Elapsed time is 0.654849 seconds.
```

```
Remaining clusters to partition 7
Processing cluster 37 now ...
Processing data subset with 18021 genes and 13 cells:
Remove genes detected in <10 cells. Remaining 0 genes.  Elapsed time is 0.000772 seconds.

Remaining clusters to partition 6
Processing cluster 38 now ...
Processing data subset with 18021 genes and 48 cells:
Remove genes detected in <10 cells. Remaining 7535 genes.  Elapsed time is 0.004027 seconds.
Iterate 10 random permutations for gene-gene similarity threshold ...   10 Elapsed time is 34.432495 seconds.
Compute gene-gene similarity ... Elapsed time is 3.010441 seconds.
Create gene-gene graph for clustering genes ...
Writing graph into file ... 100%Elapsed time is 0.352072 seconds.
Running ModularityOptimizer for clustering ...Elapsed time is 0.470765 seconds.
Gene-gene graph contains 0 pathways, 0 genes in total
Elapsed time is 0.710084 seconds.

Remaining clusters to partition 5
Processing cluster 39 now ...
Processing data subset with 18021 genes and 35 cells:
Remove genes detected in <10 cells. Remaining 5130 genes.  Elapsed time is 0.002847 seconds.
Iterate 10 random permutations for gene-gene similarity threshold ...   10 Elapsed time is 16.119349 seconds.
Compute gene-gene similarity ... Elapsed time is 1.380986 seconds.
Create gene-gene graph for clustering genes ...
Writing graph into file ... 100%Elapsed time is 0.166447 seconds.
Running ModularityOptimizer for clustering ...Elapsed time is 0.406301 seconds.
Gene-gene graph contains 0 pathways, 0 genes in total
Elapsed time is 0.549030 seconds.

Remaining clusters to partition 4
Processing cluster 40 now ...
Processing data subset with 18021 genes and 88 cells:
Remove genes detected in <10 cells. Remaining 9527 genes.  Elapsed time is 0.008785 seconds.
Iterate 10 random permutations for gene-gene similarity threshold ...   10 Elapsed time is 56.582312 seconds.
Compute gene-gene similarity ... Elapsed time is 4.880417 seconds.
Create gene-gene graph for clustering genes ...
Writing graph into file ... 100%Elapsed time is 0.562467 seconds.
Running ModularityOptimizer for clustering ...Elapsed time is 0.603722 seconds.
Gene-gene graph contains 0 pathways, 0 genes in total
Elapsed time is 0.924155 seconds.

Remaining clusters to partition 3
Processing cluster 41 now ...
Processing data subset with 18021 genes and 136 cells:
Remove genes detected in <10 cells. Remaining 11037 genes.  Elapsed time is 0.013204 seconds.
Iterate 10 random permutations for gene-gene similarity threshold ...   10 Elapsed time is 78.657115 seconds.
Compute gene-gene similarity ... Elapsed time is 6.657138 seconds.
Create gene-gene graph for clustering genes ...
Writing graph into file ... 100%Elapsed time is 0.744561 seconds.
Running ModularityOptimizer for clustering ...Elapsed time is 0.758586 seconds.
Gene-gene graph contains 4 pathways, 196 genes in total
Elapsed time is 1.168835 seconds.
Create cell-cell graph for clustering cells ...
Writing graph into file ... 100%Elapsed time is 0.018168 seconds.
Running ModularityOptimizer for clustering ...Elapsed time is 0.273488 seconds.
Cell-cell graph contains 4 cell types by community detection
Elapsed time is 0.280724 seconds.
Cell-cell graph contains 4 cell types after merging tiny cell clusters
creating a total of 3 edges ...      3
Cell-cell graph contains 1 cell types after merging

Remaining clusters to partition 2
Processing cluster 42 now ...
Processing data subset with 18021 genes and 45 cells:
Remove genes detected in <10 cells. Remaining 6568 genes.  Elapsed time is 0.003146 seconds.
Iterate 10 random permutations for gene-gene similarity threshold ...   10 Elapsed time is 26.067422 seconds.
Compute gene-gene similarity ... Elapsed time is 2.334532 seconds.
Create gene-gene graph for clustering genes ...
Writing graph into file ... 100%Elapsed time is 0.273321 seconds.
Running ModularityOptimizer for clustering ...Elapsed time is 0.474225 seconds.
Gene-gene graph contains 0 pathways, 0 genes in total
Elapsed time is 0.678083 seconds.

Remaining clusters to partition 1
Processing cluster 43 now ...
Processing data subset with 18021 genes and 694 cells:
Remove genes detected in <10 cells. Remaining 14466 genes.  Elapsed time is 0.071966 seconds.
Iterate 10 random permutations for gene-gene similarity threshold ...   10 Elapsed time is 155.203278 seconds.
Compute gene-gene similarity ... Elapsed time is 12.381053 seconds.
Create gene-gene graph for clustering genes ...
Writing graph into file ... 100%Elapsed time is 1.311309 seconds.
Running ModularityOptimizer for clustering ...Elapsed time is 1.334342 seconds.
Gene-gene graph contains 9 pathways, 1411 genes in total
Elapsed time is 1.931152 seconds.
Create cell-cell graph for clustering cells ...
Writing graph into file ... 100%Elapsed time is 0.081478 seconds.
Running ModularityOptimizer for clustering ...Elapsed time is 0.515318 seconds.
Cell-cell graph contains 8 cell types by community detection
Elapsed time is 0.534583 seconds.
Cell-cell graph contains 8 cell types after merging tiny cell clusters
creating a total of 7 edges ...      7
Cell-cell graph contains 2 cell types after merging
Number of useful pathways is 1
```

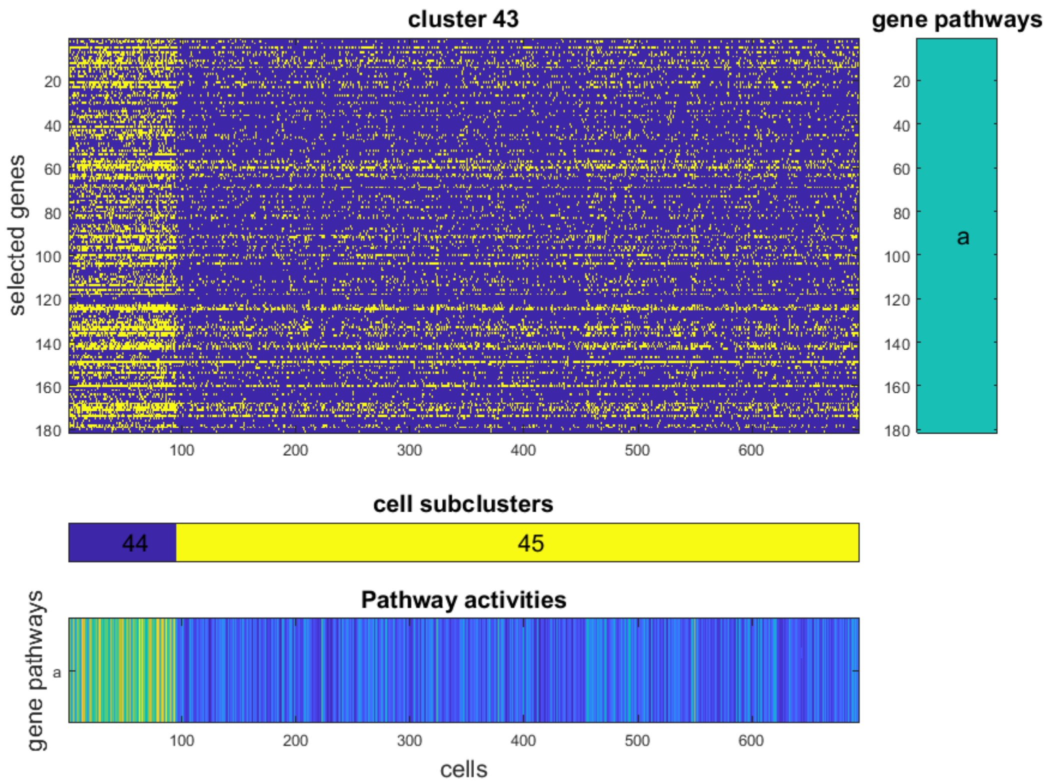


```
Remaining clusters to partition 2
Processing cluster 44 now ...
Processing data subset with 18021 genes and 95 cells:
Remove genes detected in <10 cells. Remaining 10130 genes.  Elapsed time is 0.008275 seconds.
Iterate 10 random permutations for gene-gene similarity threshold ...   10 Elapsed time is 65.750753 seconds.
Compute gene-gene similarity ... Elapsed time is 5.840025 seconds.
Create gene-gene graph for clustering genes ...
Writing graph into file ... 100%Elapsed time is 0.656284 seconds.
Running ModularityOptimizer for clustering ...Elapsed time is 0.730056 seconds.
Gene-gene graph contains 9 pathways, 285 genes in total
Elapsed time is 1.095873 seconds.
Create cell-cell graph for clustering cells ...
Writing graph into file ... 100%Elapsed time is 0.011472 seconds.
Running ModularityOptimizer for clustering ...Elapsed time is 0.257952 seconds.
Cell-cell graph contains 3 cell types by community detection
Elapsed time is 0.262579 seconds.
Cell-cell graph contains 3 cell types after merging tiny cell clusters
Cell-cell graph contains 2 cell types after merging
Number of useful pathways is 1
```

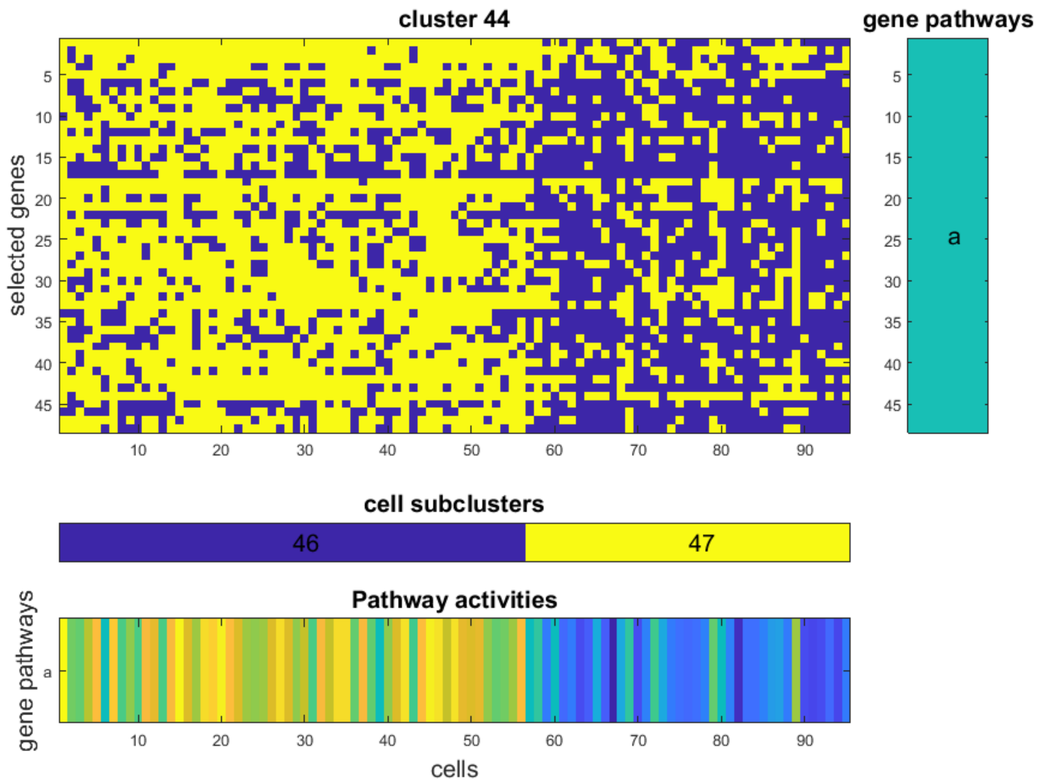


```
Remaining clusters to partition 3
Processing cluster 45 now ...
Processing data subset with 18021 genes and 599 cells:
Remove genes detected in <10 cells. Remaining 13900 genes.  Elapsed time is 0.059052 seconds.
Iterate 10 random permutations for gene-gene similarity threshold ...   10 Elapsed time is 151.390237 seconds.
Compute gene-gene similarity ... Elapsed time is 12.433586 seconds.
Create gene-gene graph for clustering genes ...
Writing graph into file ... 100%Elapsed time is 1.283538 seconds.
Running ModularityOptimizer for clustering ...Elapsed time is 1.122814 seconds.
Gene-gene graph contains 8 pathways, 1028 genes in total
Elapsed time is 1.707018 seconds.
Create cell-cell graph for clustering cells ...
Writing graph into file ... 100%Elapsed time is 0.075075 seconds.
Running ModularityOptimizer for clustering ...Elapsed time is 0.519851 seconds.
Cell-cell graph contains 8 cell types by community detection
Elapsed time is 0.535895 seconds.
Cell-cell graph contains 6 cell types after merging tiny cell clusters
Cell-cell graph contains 3 cell types after merging
Number of useful pathways is 2
```

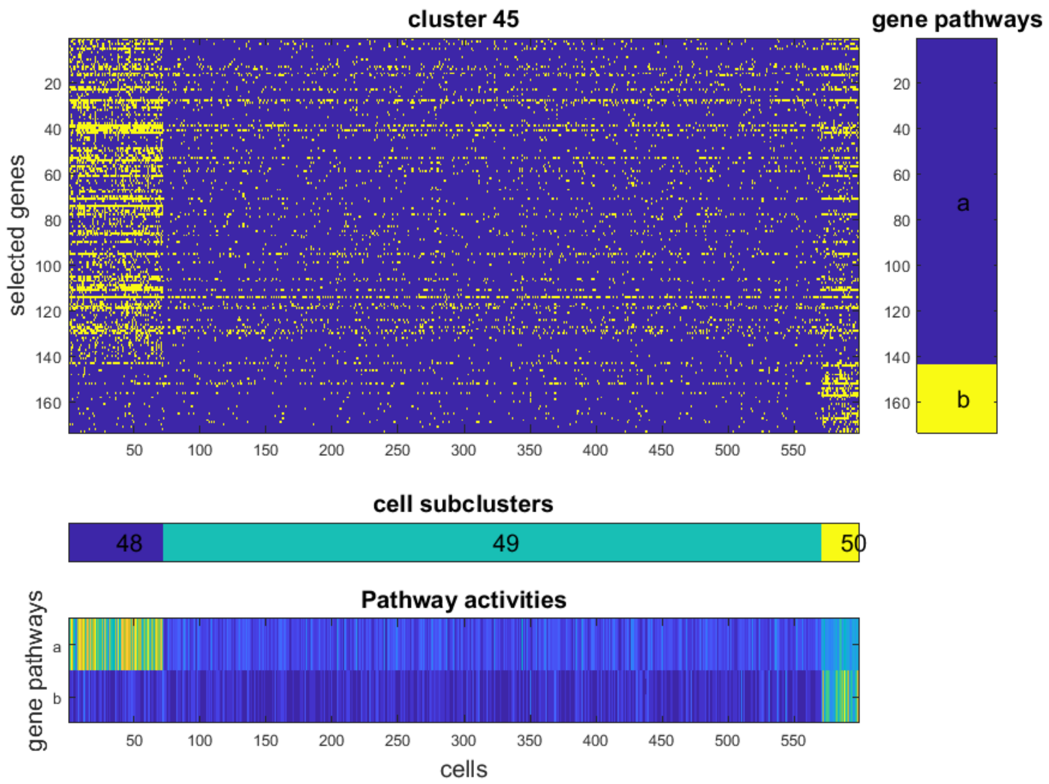


```
Remaining clusters to partition 5
Processing cluster 46 now ...
Processing data subset with 18021 genes and 56 cells:
Remove genes detected in <10 cells. Remaining 8368 genes.  Elapsed time is 0.004789 seconds.
Iterate 10 random permutations for gene-gene similarity threshold ...   10 Elapsed time is 45.690591 seconds.
Compute gene-gene similarity ... Elapsed time is 3.913595 seconds.
Create gene-gene graph for clustering genes ...
Writing graph into file ... 100%Elapsed time is 0.463295 seconds.
Running ModularityOptimizer for clustering ...Elapsed time is 0.589579 seconds.
Gene-gene graph contains 1 pathways, 26 genes in total
Elapsed time is 0.863620 seconds.
Create cell-cell graph for clustering cells ...
Writing graph into file ... 100%Elapsed time is 0.006799 seconds.
Running ModularityOptimizer for clustering ...Elapsed time is 0.214917 seconds.
Cell-cell graph contains 2 cell types by community detection
Elapsed time is 0.218815 seconds.
Cell-cell graph contains 2 cell types after merging tiny cell clusters
Cell-cell graph contains 2 cell types after merging
Number of useful pathways is 1
```

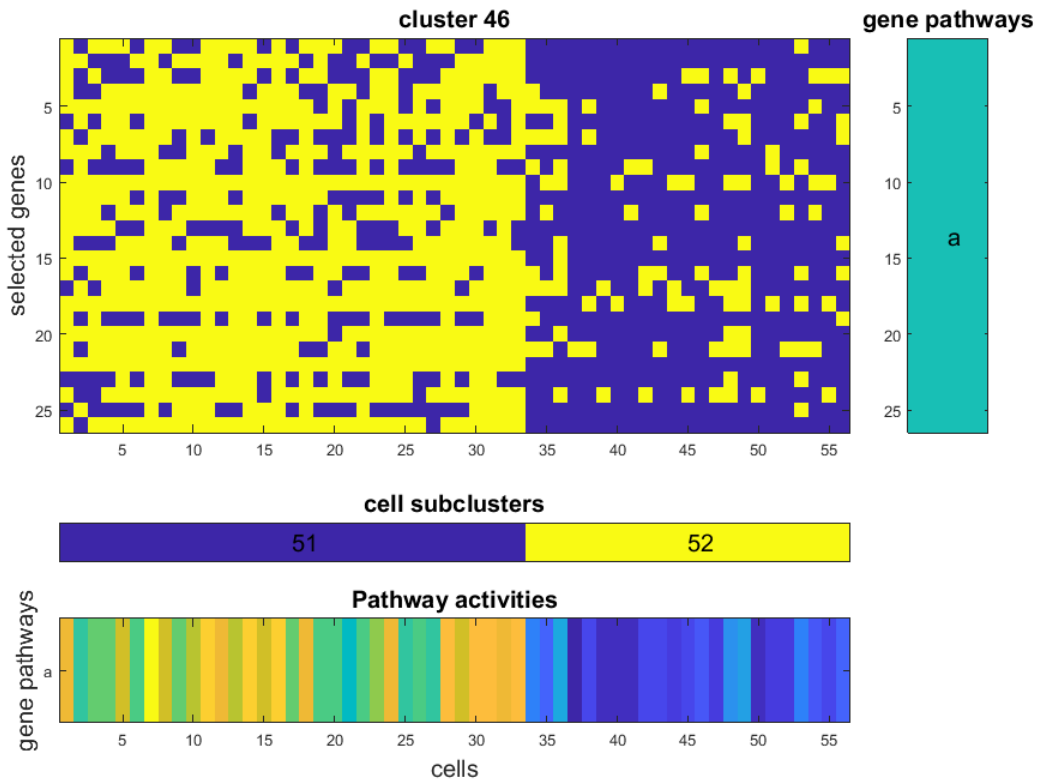


```
Remaining clusters to partition 6
Processing cluster 47 now ...
Processing data subset with 18021 genes and 39 cells:
Remove genes detected in <10 cells. Remaining 5141 genes.  Elapsed time is 0.003080 seconds.
Iterate 10 random permutations for gene-gene similarity threshold ...   10 Elapsed time is 16.987343 seconds.
Compute gene-gene similarity ... Elapsed time is 1.488576 seconds.
Create gene-gene graph for clustering genes ...
Writing graph into file ... 100%Elapsed time is 0.173919 seconds.
Running ModularityOptimizer for clustering ...Elapsed time is 0.519676 seconds.
Gene-gene graph contains 2 pathways, 57 genes in total
Elapsed time is 0.669846 seconds.
Create cell-cell graph for clustering cells ...
Writing graph into file ... 100%Elapsed time is 0.004247 seconds.
Running ModularityOptimizer for clustering ...Elapsed time is 0.198508 seconds.
Cell-cell graph contains 2 cell types by community detection
Elapsed time is 0.201982 seconds.
Cell-cell graph contains 2 cell types after merging tiny cell clusters
creating a total of 1 edges ...       1
Cell-cell graph contains 1 cell types after merging

Remaining clusters to partition 5
Processing cluster 48 now ...
Processing data subset with 18021 genes and 72 cells:
Remove genes detected in <10 cells. Remaining 7603 genes.  Elapsed time is 0.006438 seconds.
Iterate 10 random permutations for gene-gene similarity threshold ...   10 Elapsed time is 37.535381 seconds.
Compute gene-gene similarity ... Elapsed time is 3.266293 seconds.
Create gene-gene graph for clustering genes ...
Writing graph into file ... 100%Elapsed time is 0.377041 seconds.
Running ModularityOptimizer for clustering ...Elapsed time is 0.612522 seconds.
Gene-gene graph contains 4 pathways, 183 genes in total
Elapsed time is 0.859332 seconds.
Create cell-cell graph for clustering cells ...
Writing graph into file ... 100%Elapsed time is 0.009797 seconds.
Running ModularityOptimizer for clustering ...Elapsed time is 0.232432 seconds.
Cell-cell graph contains 3 cell types by community detection
Elapsed time is 0.236589 seconds.
Cell-cell graph contains 3 cell types after merging tiny cell clusters
Cell-cell graph contains 2 cell types after merging
Number of useful pathways is 1
```

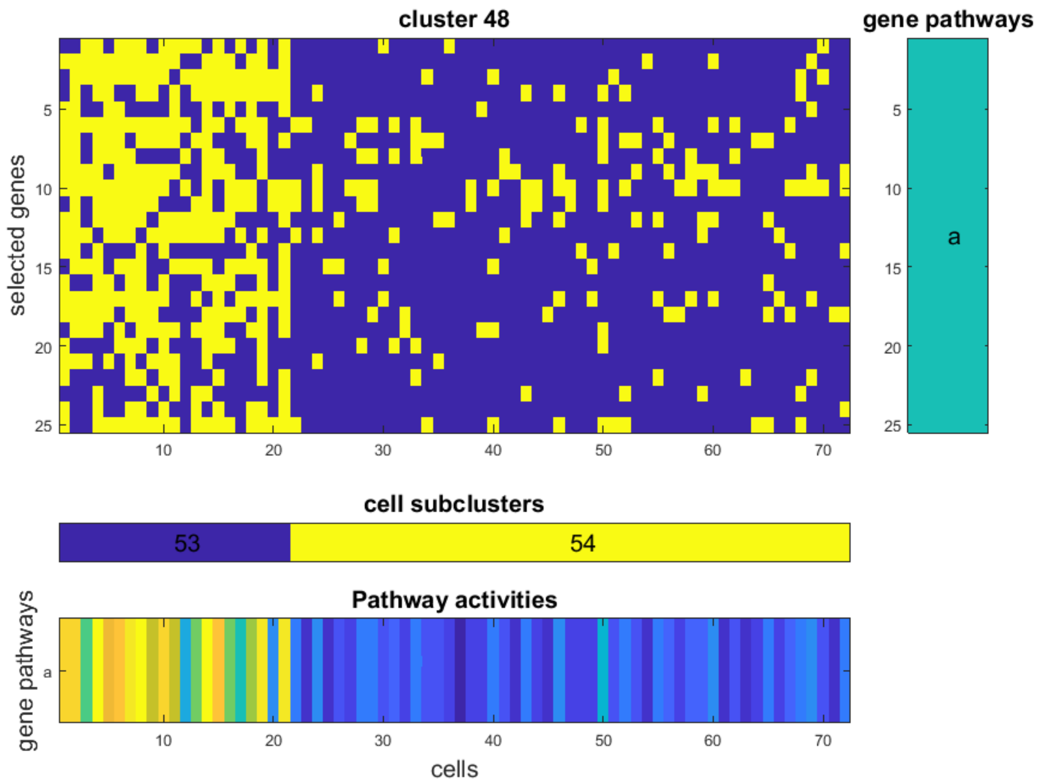


```
Remaining clusters to partition 6
Processing cluster 49 now ...
Processing data subset with 18021 genes and 498 cells:
Remove genes detected in <10 cells. Remaining 13419 genes.  Elapsed time is 0.049783 seconds.
Iterate 10 random permutations for gene-gene similarity threshold ...   10 Elapsed time is 136.879002 seconds.
Compute gene-gene similarity ... Elapsed time is 11.036315 seconds.
Create gene-gene graph for clustering genes ...
Writing graph into file ... 100%Elapsed time is 1.164909 seconds.
Running ModularityOptimizer for clustering ...Elapsed time is 1.122387 seconds.
Gene-gene graph contains 13 pathways, 640 genes in total
Elapsed time is 1.655690 seconds.
Create cell-cell graph for clustering cells ...
Writing graph into file ... 100%Elapsed time is 0.063360 seconds.
Running ModularityOptimizer for clustering ...Elapsed time is 0.474727 seconds.
Cell-cell graph contains 6 cell types by community detection
Elapsed time is 0.487774 seconds.
Cell-cell graph contains 5 cell types after merging tiny cell clusters
creating a total of 4 edges ...      4
Cell-cell graph contains 2 cell types after merging
Number of useful pathways is 1
```

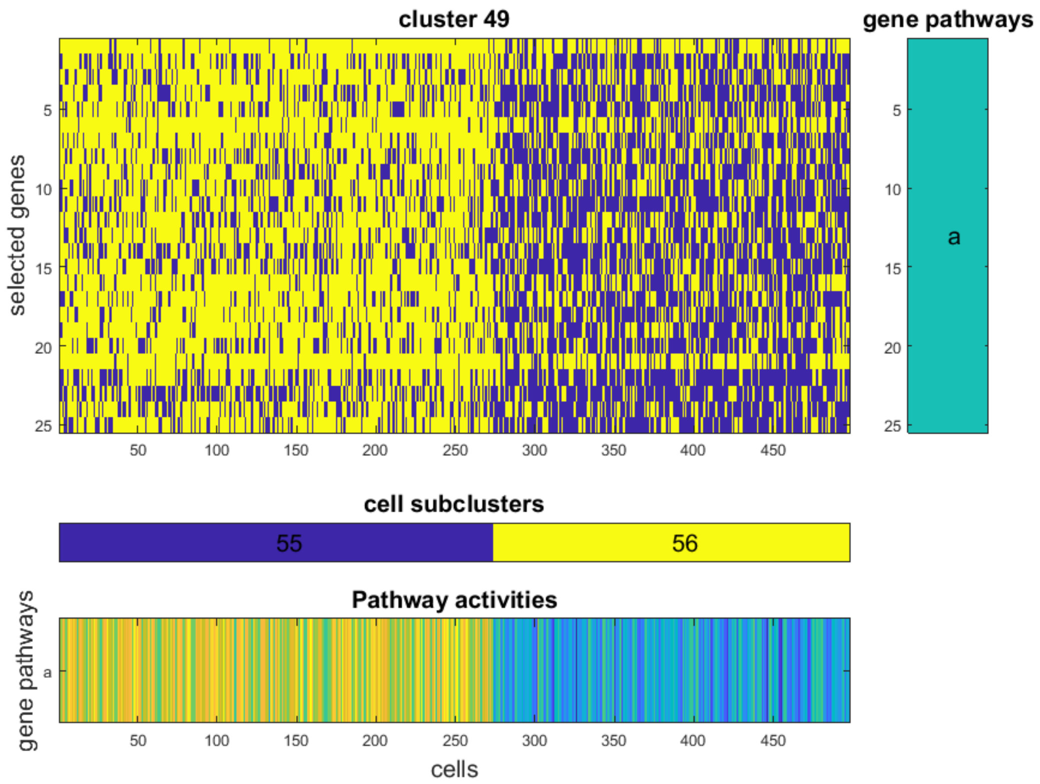


```
Remaining clusters to partition 7
Processing cluster 50 now ...
Processing data subset with 18021 genes and 29 cells:
Remove genes detected in <10 cells. Remaining 2844 genes.  Elapsed time is 0.001843 seconds.
Iterate 10 random permutations for gene-gene similarity threshold ...   10 Elapsed time is 5.142619 seconds.
Compute gene-gene similarity ... Elapsed time is 0.446527 seconds.
Create gene-gene graph for clustering genes ...
Writing graph into file ... 100%Elapsed time is 0.056743 seconds.
Running ModularityOptimizer for clustering ...Elapsed time is 0.415466 seconds.
Gene-gene graph contains 0 pathways, 0 genes in total
Elapsed time is 0.489670 seconds.

Remaining clusters to partition 6
Processing cluster 51 now ...
Processing data subset with 18021 genes and 33 cells:
Remove genes detected in <10 cells. Remaining 5166 genes.  Elapsed time is 0.003297 seconds.
Iterate 10 random permutations for gene-gene similarity threshold ...   10 Elapsed time is 16.970779 seconds.
Compute gene-gene similarity ... Elapsed time is 1.510677 seconds.
Create gene-gene graph for clustering genes ...
Writing graph into file ... 100%Elapsed time is 0.171307 seconds.
Running ModularityOptimizer for clustering ...Elapsed time is 0.472866 seconds.
Gene-gene graph contains 0 pathways, 0 genes in total
Elapsed time is 0.630918 seconds.

Remaining clusters to partition 5
Processing cluster 52 now ...
Processing data subset with 18021 genes and 23 cells:
Remove genes detected in <10 cells. Remaining 2033 genes.  Elapsed time is 0.001748 seconds.
Iterate 10 random permutations for gene-gene similarity threshold ...   10 Elapsed time is 2.566556 seconds.
Compute gene-gene similarity ... Elapsed time is 0.237430 seconds.
Create gene-gene graph for clustering genes ...
Writing graph into file ... 101%Elapsed time is 0.029879 seconds.
Running ModularityOptimizer for clustering ...Elapsed time is 0.306116 seconds.
Gene-gene graph contains 0 pathways, 0 genes in total
Elapsed time is 0.358038 seconds.

Remaining clusters to partition 4
Processing cluster 53 now ...
Processing data subset with 18021 genes and 21 cells:
Remove genes detected in <10 cells. Remaining 716 genes.  Elapsed time is 0.001327 seconds.
Iterate 10 random permutations for gene-gene similarity threshold ...   10 Elapsed time is 0.316691 seconds.
Compute gene-gene similarity ... Elapsed time is 0.028550 seconds.
Create gene-gene graph for clustering genes ...
Writing graph into file ... 101%Elapsed time is 0.005430 seconds.
Running ModularityOptimizer for clustering ...Elapsed time is 0.250787 seconds.
Gene-gene graph contains 0 pathways, 0 genes in total
Elapsed time is 0.274911 seconds.

Remaining clusters to partition 3
Processing cluster 54 now ...
Processing data subset with 18021 genes and 51 cells:
Remove genes detected in <10 cells. Remaining 5843 genes.  Elapsed time is 0.004142 seconds.
Iterate 10 random permutations for gene-gene similarity threshold ...   10 Elapsed time is 22.017076 seconds.
Compute gene-gene similarity ... Elapsed time is 1.912838 seconds.
Create gene-gene graph for clustering genes ...
Writing graph into file ... 100%Elapsed time is 0.221579 seconds.
```

```
Running ModularityOptimizer for clustering ...Elapsed time is 0.522148 seconds.
Gene-gene graph contains 2 pathways, 55 genes in total
Elapsed time is 0.692423 seconds.
Create cell-cell graph for clustering cells ...
Writing graph into file ... 100%Elapsed time is 0.006477 seconds.
Running ModularityOptimizer for clustering ...Elapsed time is 0.231772 seconds.
Cell-cell graph contains 2 cell types by community detection
Elapsed time is 0.236259 seconds.
Cell-cell graph contains 2 cell types after merging tiny cell clusters
creating a total of 1 edges ...      1
Cell-cell graph contains 1 cell types after merging

Remaining clusters to partition 2
Processing cluster 55 now ...
Processing data subset with 18021 genes and 273 cells:
Remove genes detected in <10 cells. Remaining 12288 genes.  Elapsed time is 0.026586 seconds.
Iterate 10 random permutations for gene-gene similarity threshold ...   10 Elapsed time is 108.984990 seconds.
Compute gene-gene similarity ... Elapsed time is 9.073203 seconds.
Create gene-gene graph for clustering genes ...
Writing graph into file ... 100%Elapsed time is 1.005228 seconds.
Running ModularityOptimizer for clustering ...Elapsed time is 0.867557 seconds.
Gene-gene graph contains 8 pathways, 261 genes in total
Elapsed time is 1.342531 seconds.
Create cell-cell graph for clustering cells ...
Writing graph into file ... 100%Elapsed time is 0.035963 seconds.
Running ModularityOptimizer for clustering ...Elapsed time is 0.356333 seconds.
Cell-cell graph contains 7 cell types by community detection
Elapsed time is 0.364630 seconds.
Cell-cell graph contains 7 cell types after merging tiny cell clusters
creating a total of 6 edges ...      6
Cell-cell graph contains 2 cell types after merging
Number of useful pathways is 1
```

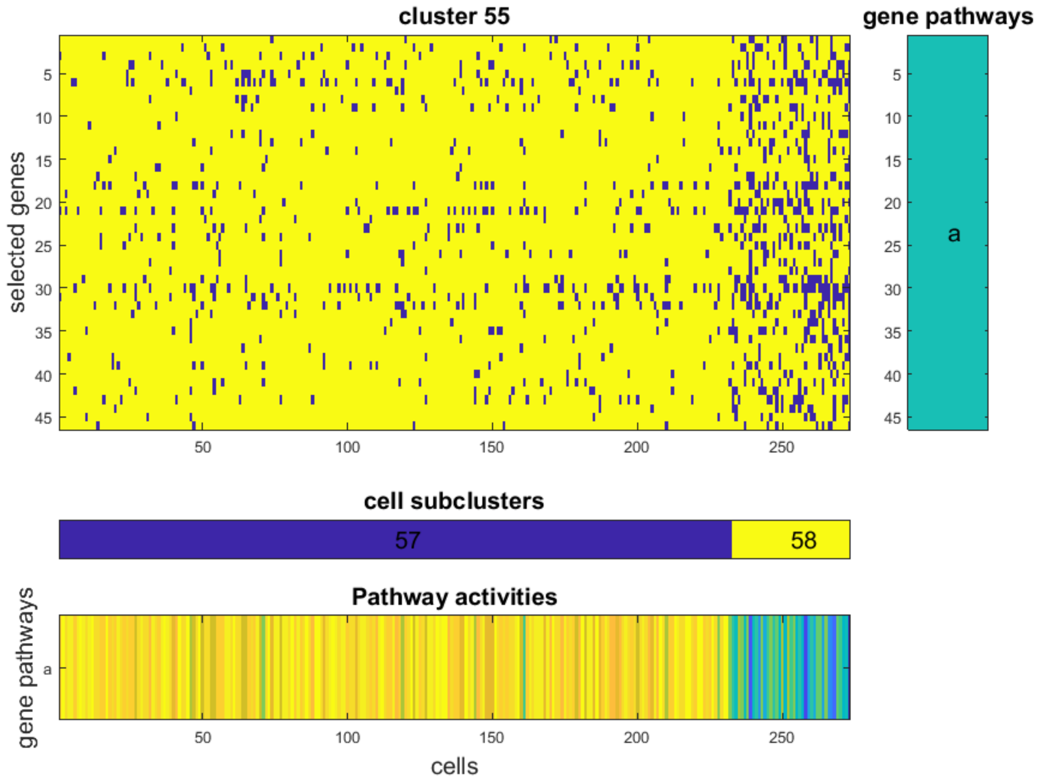


```
Remaining clusters to partition 3
Processing cluster 56 now ...
Processing data subset with 18021 genes and 225 cells:
Remove genes detected in <10 cells. Remaining 11110 genes.  Elapsed time is 0.020888 seconds.
Iterate 10 random permutations for gene-gene similarity threshold ...   10 Elapsed time is 85.342643 seconds.
Compute gene-gene similarity ... Elapsed time is 7.301043 seconds.
Create gene-gene graph for clustering genes ...
Writing graph into file ... 100%Elapsed time is 0.792515 seconds.
Running ModularityOptimizer for clustering ...Elapsed time is 0.762640 seconds.
Gene-gene graph contains 4 pathways, 128 genes in total
Elapsed time is 1.165464 seconds.
Create cell-cell graph for clustering cells ...
Writing graph into file ... 100%Elapsed time is 0.028782 seconds.
Running ModularityOptimizer for clustering ...Elapsed time is 0.312088 seconds.
Cell-cell graph contains 5 cell types by community detection
Elapsed time is 0.319724 seconds.
Cell-cell graph contains 5 cell types after merging tiny cell clusters
creating a total of 4 edges ...      4
Cell-cell graph contains 1 cell types after merging

Remaining clusters to partition 2
Processing cluster 57 now ...
```

```
Processing data subset with 18021 genes and 232 cells:
Remove genes detected in <10 cells. Remaining 11967 genes.  Elapsed time is 0.022463 seconds.
Iterate 10 random permutations for gene-gene similarity threshold ...   10 Elapsed time is 100.025780 seconds.
Compute gene-gene similarity ... Elapsed time is 8.447117 seconds.
Create gene-gene graph for clustering genes ...
Writing graph into file ... 100%Elapsed time is 0.920604 seconds.
Running ModularityOptimizer for clustering ...Elapsed time is 0.788869 seconds.
Gene-gene graph contains 6 pathways, 145 genes in total
Elapsed time is 1.243086 seconds.
Create cell-cell graph for clustering cells ...
Writing graph into file ... 100%Elapsed time is 0.029943 seconds.
Running ModularityOptimizer for clustering ...Elapsed time is 0.347817 seconds.
Cell-cell graph contains 6 cell types by community detection
Elapsed time is 0.355476 seconds.
Cell-cell graph contains 6 cell types after merging tiny cell clusters
creating a total of 5 edges ...      5
Cell-cell graph contains 2 cell types after merging
Number of useful pathways is 1
```

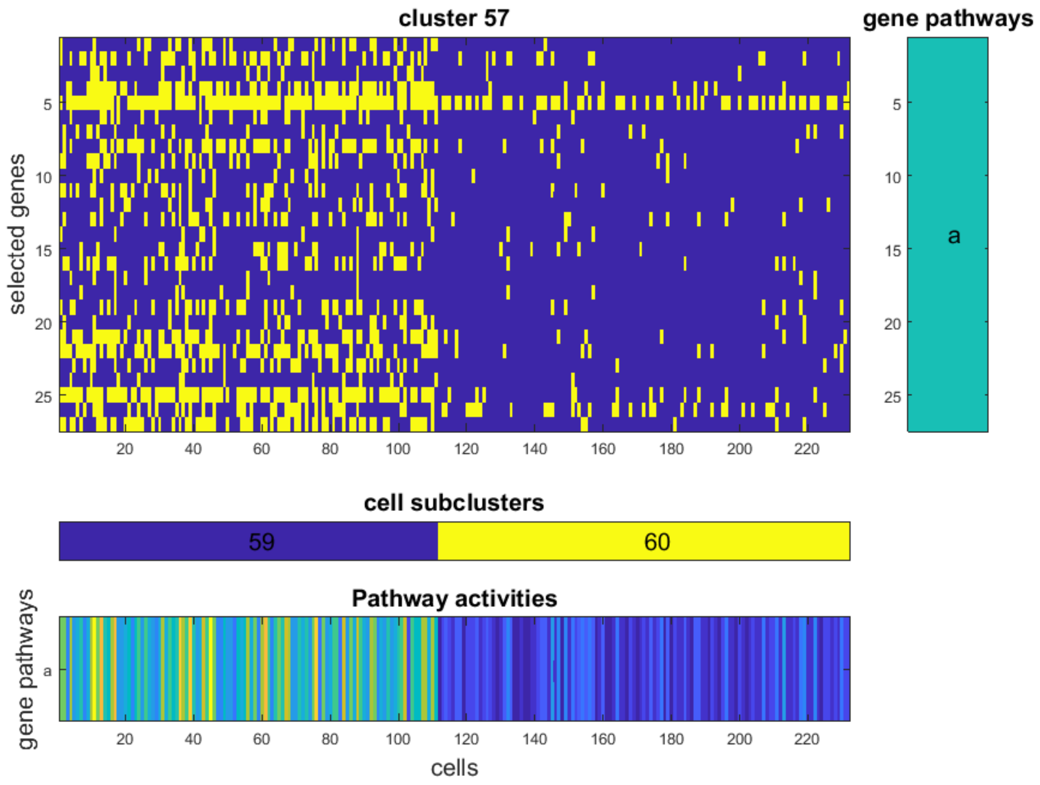


```
Remaining clusters to partition 3
Processing cluster 58 now ...
Processing data subset with 18021 genes and 41 cells:
Remove genes detected in <10 cells. Remaining 4590 genes.  Elapsed time is 0.003287 seconds.
Iterate 10 random permutations for gene-gene similarity threshold ...   10 Elapsed time is 13.455387 seconds.
Compute gene-gene similarity ... Elapsed time is 1.193782 seconds.
Create gene-gene graph for clustering genes ...
Writing graph into file ... 100%Elapsed time is 0.135772 seconds.
Running ModularityOptimizer for clustering ...Elapsed time is 0.425149 seconds.
Gene-gene graph contains 0 pathways, 0 genes in total
Elapsed time is 0.551333 seconds.

Remaining clusters to partition 2
Processing cluster 59 now ...
Processing data subset with 18021 genes and 111 cells:
Remove genes detected in <10 cells. Remaining 10097 genes.  Elapsed time is 0.012863 seconds.
Iterate 10 random permutations for gene-gene similarity threshold ...   10 Elapsed time is 68.019549 seconds.
Compute gene-gene similarity ... Elapsed time is 5.800903 seconds.
Create gene-gene graph for clustering genes ...
Writing graph into file ... 100%Elapsed time is 0.674533 seconds.
Running ModularityOptimizer for clustering ...Elapsed time is 0.667064 seconds.
Gene-gene graph contains 2 pathways, 46 genes in total
Elapsed time is 1.021196 seconds.
Create cell-cell graph for clustering cells ...
Writing graph into file ... 100%Elapsed time is 0.014790 seconds.
Running ModularityOptimizer for clustering ...Elapsed time is 0.291448 seconds.
Cell-cell graph contains 5 cell types by community detection
Elapsed time is 0.296457 seconds.
Cell-cell graph contains 2 cell types after merging tiny cell clusters
creating a total of 1 edges ...      1
Cell-cell graph contains 1 cell types after merging

Remaining clusters to partition 1
Processing cluster 60 now ...
Processing data subset with 18021 genes and 121 cells:
Remove genes detected in <10 cells. Remaining 10069 genes.  Elapsed time is 0.011417 seconds.
```

```
Iterate 10 random permutations for gene-gene similarity threshold ...   10 Elapsed time is 68.004962 seconds.
Compute gene-gene similarity ... Elapsed time is 5.845385 seconds.
Create gene-gene graph for clustering genes ...
Writing graph into file ... 100%Elapsed time is 0.653504 seconds.
Running ModularityOptimizer for clustering ...Elapsed time is 0.611861 seconds.
Gene-gene graph contains 0 pathways, 0 genes in total
Elapsed time is 0.962635 seconds.
```

number of cooccurrence clusters

```
number_of_cooccurrence_clusters = length(unique(cooc.cell_labels))
```

```
number_of_cooccurrence_clusters =

    38
```

Published with MATLAB® R2017b

— Supplementary Note 3 —

Contents

- addpath to all tools
- initiate one instance of the "cooccurrence_clustering_analysis" class
- read data prepawed in matlab file
- filter the data by removing genes and cells (same as Seurat tutorial on this data)
- binarize data
- cooccurrence clustering
- number of cooccurrence clusters

addpath to all tools

```
addpath(genpath('..\tools\'))
```

initiate one instance of the "cooccurrence_clustering_analysis" class

```
cooc = cooccurrence_clustering_analysis;
```

read data prepawed in matlab file

```
cooc = cooc.ReadMatlab('TM_droplet_mat.mat');
```

filter the data by removing genes and cells (same as Seurat tutorial on this data)

```
cooc.initial_filtering_min_num_cells = 10;
cooc.initial_filtering_min_num_genes = 0;
cooc.initial_filtering_max_num_genes = Inf;
cooc.initial_filtering_max_percent_mito = 1;
cooc = cooc.initial_filtering_of_data(1);
```

```
Data after initial filtering 18134 genes * 70118 cells.
```

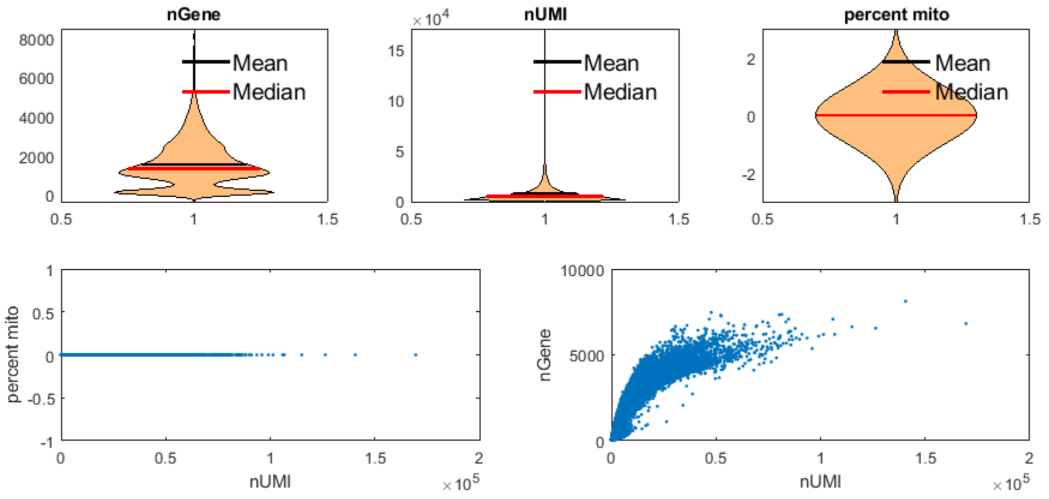

binarize data

```
cooc.binarization_threshold = 0;
cooc.binary_data = full(double(cooc.data>cooc.binarization_threshold));
```

cooccurrence clustering

```
cooc.cooccurrence_min_expressed_cells = 100;            % genes will only be considered if detected in >= minimum number of cells, and undetected in >= minimum number of cells
cooc.cooccurrence_min_pathway_size = 20;                % only considered gene clusters of size >= this threshold
cooc.cooccurrence_min_population_size = 10;             % only considered gene clusters of size >= this threshold
cooc.cooccurrence_snr_merge_threshold = 1.5;            % threshold for merging Louvain communities, based on snr of average detection of each gene cluster
cooc.cooccurrence_mean_diff_merge_threshold = 0.5;      % threshold for merging Louvain communities
cooc.cooccurrence_mean_ratio_merge_threshold = 2;       % threshold for merging Louvain communities


cooc = cooc.iterative_cooccurance_clustering;
```

```
Remaining clusters to partition 1
Processing cluster 0 now ...
Processing data subset with 18134 genes and 70118 cells:
Remove genes detected in <100 cells. Remaining 15864 genes.  Elapsed time is 20.929428 seconds.
Iterate 10 random permutations for gene-gene similarity threshold ...   10 Elapsed time is 5132.383225 seconds.
Compute gene-gene similarity ... Elapsed time is 192.729258 seconds.
Create gene-gene graph for clustering genes ...
Writing graph into file ... 100%Elapsed time is 353.832596 seconds.
Running ModularityOptimizer for clustering ...Elapsed time is 1173.058794 seconds.
Gene-gene graph contains 6 pathways, 15849 genes in total
Elapsed time is 1173.991731 seconds.
```

```
Create cell-cell graph for clustering cells ...
Writing graph into file ... 100%Elapsed time is 7.902195 seconds.
Running ModularityOptimizer for clustering ...Elapsed time is 96.062367 seconds.
Cell-cell graph contains 55 cell types by community detection
Elapsed time is 97.533749 seconds.
Cell-cell graph contains 30 cell types after merging tiny cell clusters
Cell-cell graph contains 2 cell types after merging
Number of useful pathways is 1
```

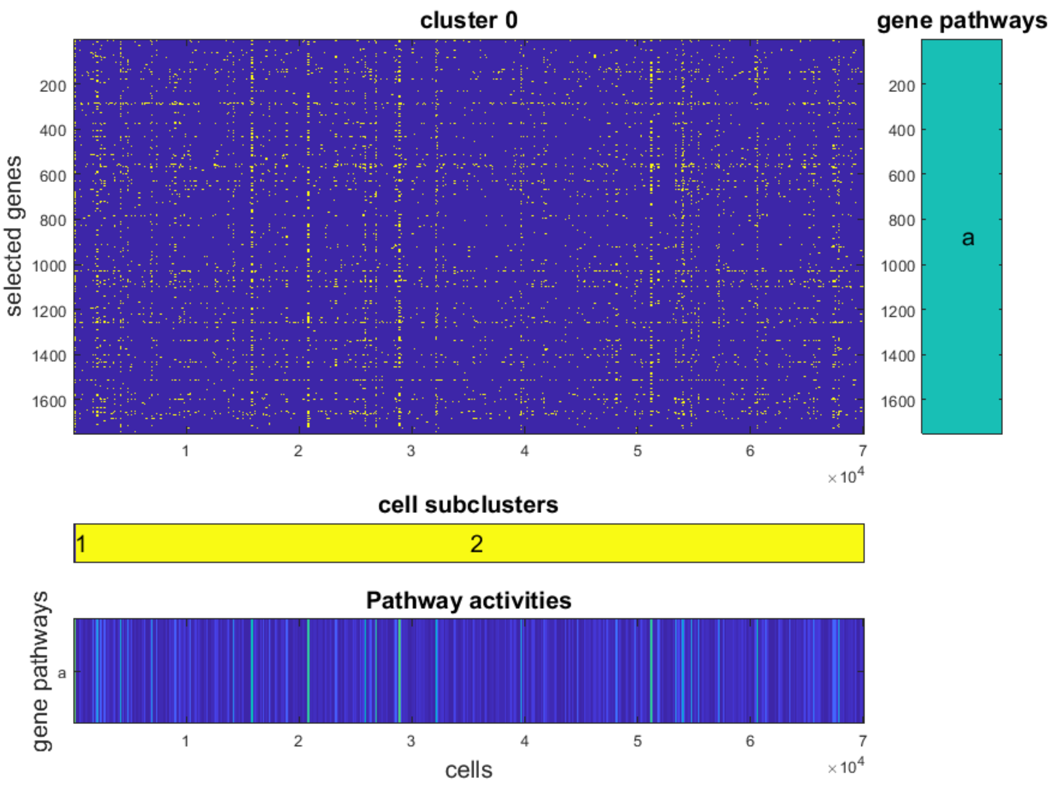


```
Remaining clusters to partition 2
Processing cluster 1 now ...
Processing data subset with 18134 genes and 275 cells:
Remove genes detected in <100 cells. Remaining 1971 genes.  Elapsed time is 0.016036 seconds.
Iterate 10 random permutations for gene-gene similarity threshold ...   10 Elapsed time is 2.898827 seconds.
Compute gene-gene similarity ... Elapsed time is 0.222744 seconds.
Create gene-gene graph for clustering genes ...
Writing graph into file ... 100%Elapsed time is 0.227621 seconds.
Running ModularityOptimizer for clustering ...Elapsed time is 0.631667 seconds.
Gene-gene graph contains 2 pathways, 1175 genes in total
Elapsed time is 0.689631 seconds.
Create cell-cell graph for clustering cells ...
Writing graph into file ... 100%Elapsed time is 0.032860 seconds.
Running ModularityOptimizer for clustering ...Elapsed time is 0.316148 seconds.
Cell-cell graph contains 6 cell types by community detection
Elapsed time is 0.327066 seconds.
Cell-cell graph contains 3 cell types after merging tiny cell clusters
creating a total of 2 edges ...      2
Cell-cell graph contains 1 cell types after merging

Remaining clusters to partition 1
Processing cluster 2 now ...
Processing data subset with 18134 genes and 69843 cells:
Remove genes detected in <100 cells. Remaining 15833 genes.  Elapsed time is 143.669741 seconds.
Iterate 10 random permutations for gene-gene similarity threshold ...   10 Elapsed time is 5996.462466 seconds.
Compute gene-gene similarity ... Elapsed time is 192.457210 seconds.
Create gene-gene graph for clustering genes ...
Writing graph into file ... 100%Elapsed time is 345.998631 seconds.
Running ModularityOptimizer for clustering ...Elapsed time is 1310.088558 seconds.
Gene-gene graph contains 6 pathways, 15814 genes in total
Elapsed time is 1310.999377 seconds.
Create cell-cell graph for clustering cells ...
Writing graph into file ... 100%Elapsed time is 7.944623 seconds.
Running ModularityOptimizer for clustering ...Elapsed time is 92.891636 seconds.
Cell-cell graph contains 54 cell types by community detection
Elapsed time is 94.340668 seconds.
Cell-cell graph contains 28 cell types after merging tiny cell clusters
Cell-cell graph contains 3 cell types after merging
Number of useful pathways is 2
```

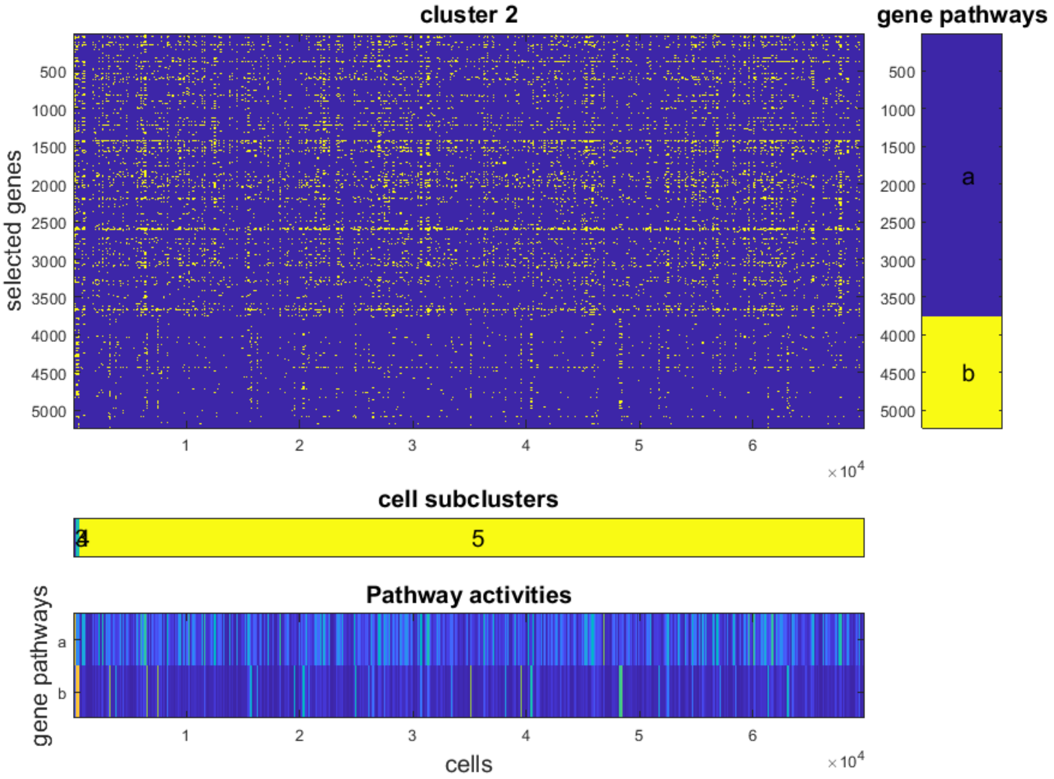


```
Remaining clusters to partition 3
Processing cluster 3 now ...
Processing data subset with 18134 genes and 217 cells:
Remove genes detected in <100 cells. Remaining 833 genes.  Elapsed time is 0.011238 seconds.
Iterate 10 random permutations for gene-gene similarity threshold ...   10 Elapsed time is 0.521736 seconds.
Compute gene-gene similarity ... Elapsed time is 0.037976 seconds.
Create gene-gene graph for clustering genes ...
Writing graph into file ... 100%Elapsed time is 0.030717 seconds.
Running ModularityOptimizer for clustering ...Elapsed time is 0.322663 seconds.
Gene-gene graph contains 3 pathways, 340 genes in total
Elapsed time is 0.347559 seconds.
Create cell-cell graph for clustering cells ...
Writing graph into file ... 100%Elapsed time is 0.025546 seconds.
Running ModularityOptimizer for clustering ...Elapsed time is 0.270046 seconds.
Cell-cell graph contains 5 cell types by community detection
Elapsed time is 0.280298 seconds.
Cell-cell graph contains 5 cell types after merging tiny cell clusters
creating a total of 4 edges ...      4
Cell-cell graph contains 2 cell types after merging
Number of useful pathways is 1
```

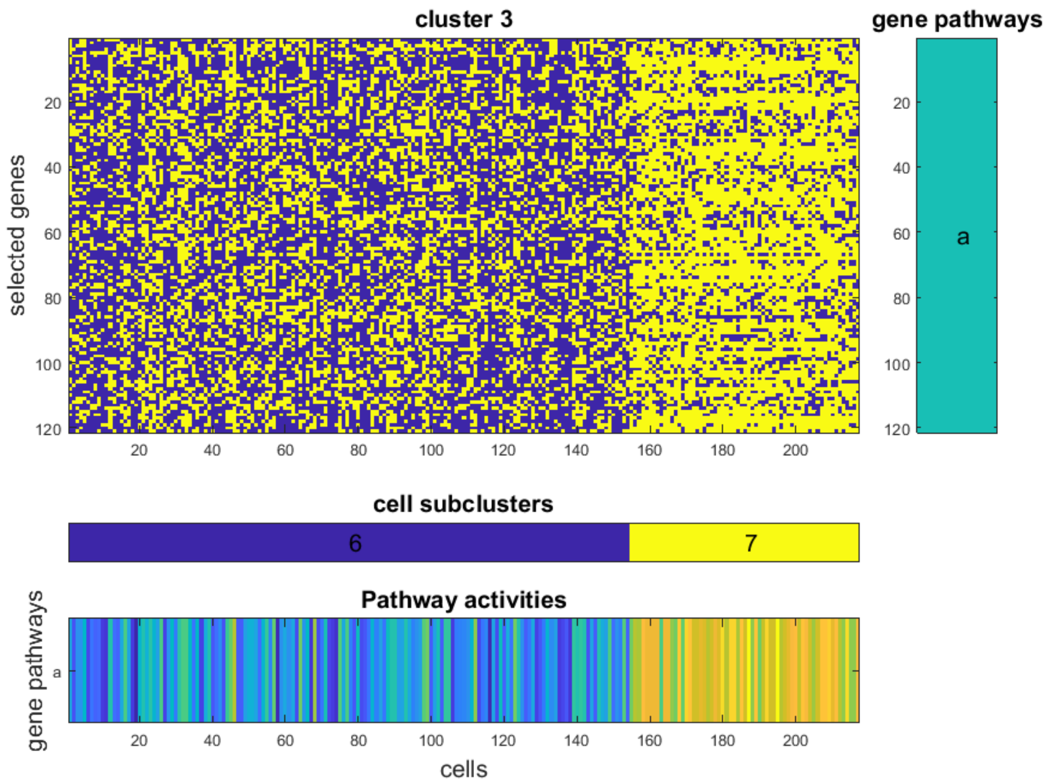


```
Remaining clusters to partition 4
Processing cluster 4 now ...
Processing data subset with 18134 genes and 291 cells:
Remove genes detected in <100 cells. Remaining 2014 genes.  Elapsed time is 0.021224 seconds.
Iterate 10 random permutations for gene-gene similarity threshold ...   10 Elapsed time is 3.027758 seconds.
Compute gene-gene similarity ... Elapsed time is 0.234153 seconds.
Create gene-gene graph for clustering genes ...
Writing graph into file ... 100%Elapsed time is 0.037075 seconds.
Running ModularityOptimizer for clustering ...Elapsed time is 0.331129 seconds.
Gene-gene graph contains 4 pathways, 346 genes in total
Elapsed time is 0.386267 seconds.
Create cell-cell graph for clustering cells ...
Writing graph into file ... 100%Elapsed time is 0.032871 seconds.
Running ModularityOptimizer for clustering ...Elapsed time is 0.313420 seconds.
Cell-cell graph contains 6 cell types by community detection
Elapsed time is 0.324734 seconds.
Cell-cell graph contains 6 cell types after merging tiny cell clusters
Cell-cell graph contains 3 cell types after merging
Number of useful pathways is 4
```

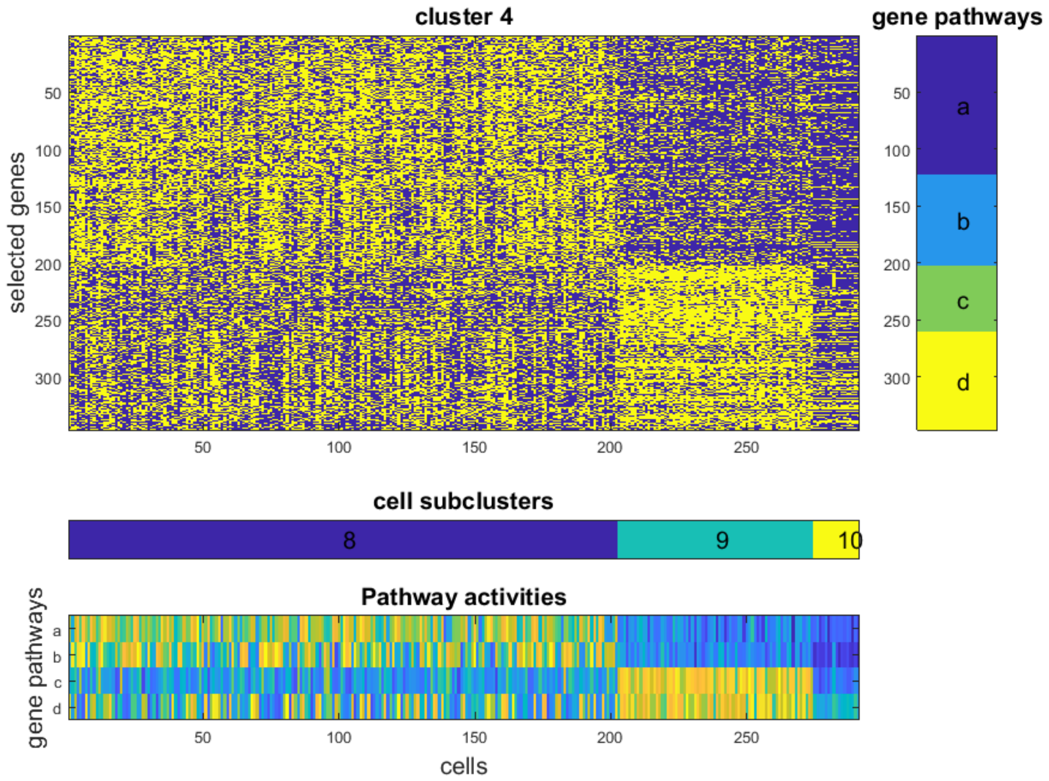


```
Remaining clusters to partition 6
Processing cluster 5 now ...
Processing data subset with 18134 genes and 69335 cells:
Remove genes detected in <100 cells. Remaining 15767 genes.  Elapsed time is 6.396783 seconds.
Iterate 10 random permutations for gene-gene similarity threshold ...   10 Elapsed time is 5540.363153 seconds.
Compute gene-gene similarity ... Elapsed time is 188.858636 seconds.
Create gene-gene graph for clustering genes ...
Writing graph into file ... 100%Elapsed time is 339.087178 seconds.
Running ModularityOptimizer for clustering ...Elapsed time is 1206.291100 seconds.
Gene-gene graph contains 5 pathways, 15749 genes in total
Elapsed time is 1207.196815 seconds.
Create cell-cell graph for clustering cells ...
Writing graph into file ... 100%Elapsed time is 7.756267 seconds.
Running ModularityOptimizer for clustering ...Elapsed time is 89.168565 seconds.
Cell-cell graph contains 36 cell types by community detection
Elapsed time is 90.601580 seconds.
Cell-cell graph contains 18 cell types after merging tiny cell clusters
creating a total of 17 edges ...     17
Cell-cell graph contains 2 cell types after merging
Number of useful pathways is 1
```

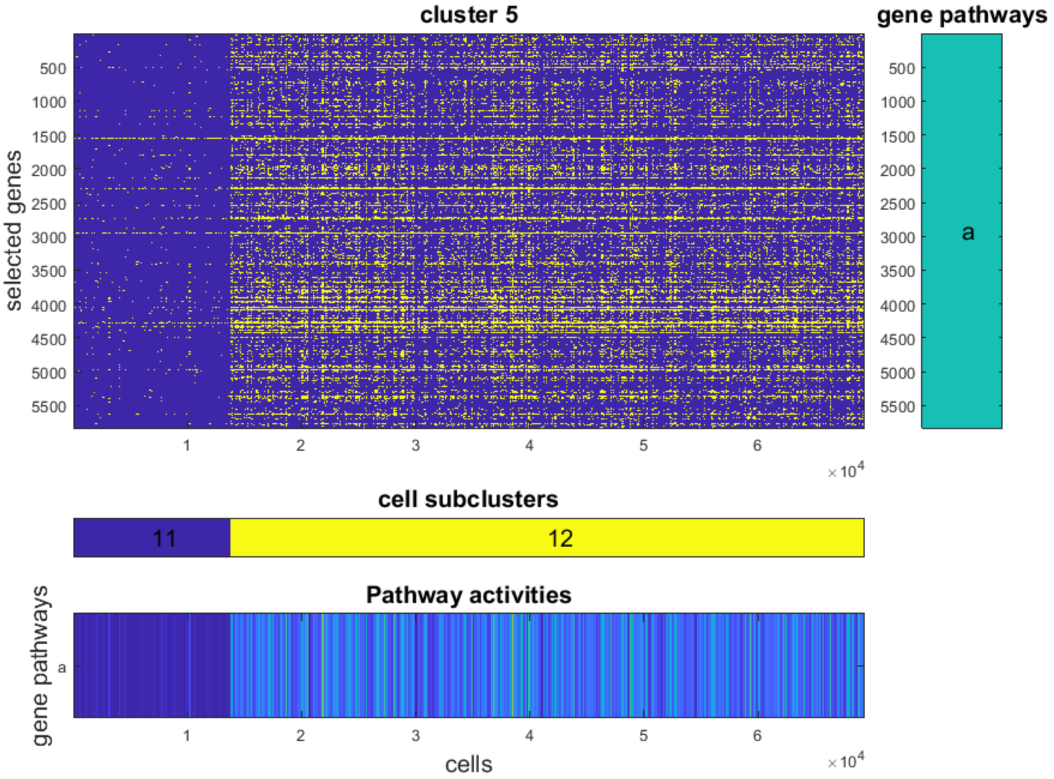


```
Remaining clusters to partition 7
Processing cluster 6 now ...
Processing data subset with 18134 genes and 154 cells:
Remove genes detected in <100 cells. Remaining 0 genes.  Elapsed time is 0.010371 seconds.

Remaining clusters to partition 6
Processing cluster 7 now ...
Processing data subset with 18134 genes and 63 cells:
Remove genes detected in <100 cells. Remaining 0 genes.  Elapsed time is 0.003031 seconds.

Remaining clusters to partition 5
Processing cluster 8 now ...
Processing data subset with 18134 genes and 202 cells:
Remove genes detected in <100 cells. Remaining 86 genes.  Elapsed time is 0.009116 seconds.
Iterate 10 random permutations for gene-gene similarity threshold ...   10 Elapsed time is 0.020521 seconds.
Compute gene-gene similarity ... Elapsed time is 0.000881 seconds.
Create gene-gene graph for clustering genes ...
Writing graph into file ... 107%Elapsed time is 0.001856 seconds.
Running ModularityOptimizer for clustering ...Elapsed time is 0.178965 seconds.
Gene-gene graph contains 0 pathways, 0 genes in total
Elapsed time is 0.214972 seconds.

Remaining clusters to partition 4
Processing cluster 9 now ...
Processing data subset with 18134 genes and 72 cells:
Remove genes detected in <100 cells. Remaining 0 genes.  Elapsed time is 0.003240 seconds.

Remaining clusters to partition 3
Processing cluster 10 now ...
Processing data subset with 18134 genes and 17 cells:
Remove genes detected in <100 cells. Remaining 0 genes.  Elapsed time is 0.000818 seconds.

Remaining clusters to partition 2
Processing cluster 11 now ...
Processing data subset with 18134 genes and 13706 cells:
Remove genes detected in <100 cells. Remaining 3968 genes.  Elapsed time is 0.854254 seconds.
Iterate 10 random permutations for gene-gene similarity threshold ...   10 Elapsed time is 79.718762 seconds.
Compute gene-gene similarity ... Elapsed time is 3.505407 seconds.
Create gene-gene graph for clustering genes ...
Writing graph into file ... 100%Elapsed time is 2.389369 seconds.
Running ModularityOptimizer for clustering ...Elapsed time is 6.583819 seconds.
Gene-gene graph contains 8 pathways, 3909 genes in total
Elapsed time is 6.695332 seconds.
Create cell-cell graph for clustering cells ...
Writing graph into file ... 100%Elapsed time is 1.615264 seconds.
Running ModularityOptimizer for clustering ...Elapsed time is 10.091254 seconds.
Cell-cell graph contains 25 cell types by community detection
Elapsed time is 10.373622 seconds.
Cell-cell graph contains 23 cell types after merging tiny cell clusters
creating a total of 22 edges ...     22
Cell-cell graph contains 2 cell types after merging
Number of useful pathways is 1
```

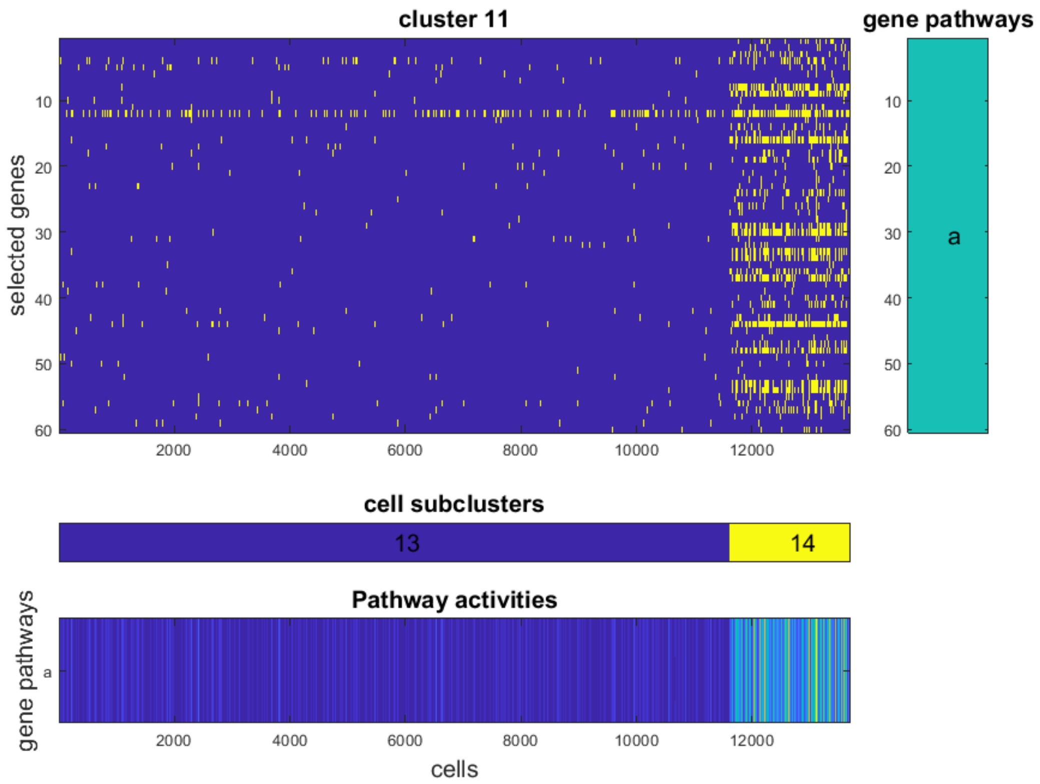


```
Remaining clusters to partition 3
Processing cluster 12 now ...
Processing data subset with 18134 genes and 55629 cells:
Remove genes detected in <100 cells. Remaining 15719 genes.  Elapsed time is 5.104543 seconds.
Iterate 10 random permutations for gene-gene similarity threshold ...   10 Elapsed time is 3437.334976 seconds.
Compute gene-gene similarity ... Elapsed time is 154.070295 seconds.
Create gene-gene graph for clustering genes ...
Writing graph into file ... 100%Elapsed time is 278.195903 seconds.
Running ModularityOptimizer for clustering ...Elapsed time is 768.695779 seconds.
Gene-gene graph contains 5 pathways, 15679 genes in total
Elapsed time is 769.551155 seconds.
Create cell-cell graph for clustering cells ...
Writing graph into file ... 100%Elapsed time is 6.239055 seconds.
Running ModularityOptimizer for clustering ...Elapsed time is 73.960915 seconds.
Cell-cell graph contains 33 cell types by community detection
Elapsed time is 75.106258 seconds.
Cell-cell graph contains 15 cell types after merging tiny cell clusters
Cell-cell graph contains 2 cell types after merging
Number of useful pathways is 1
```

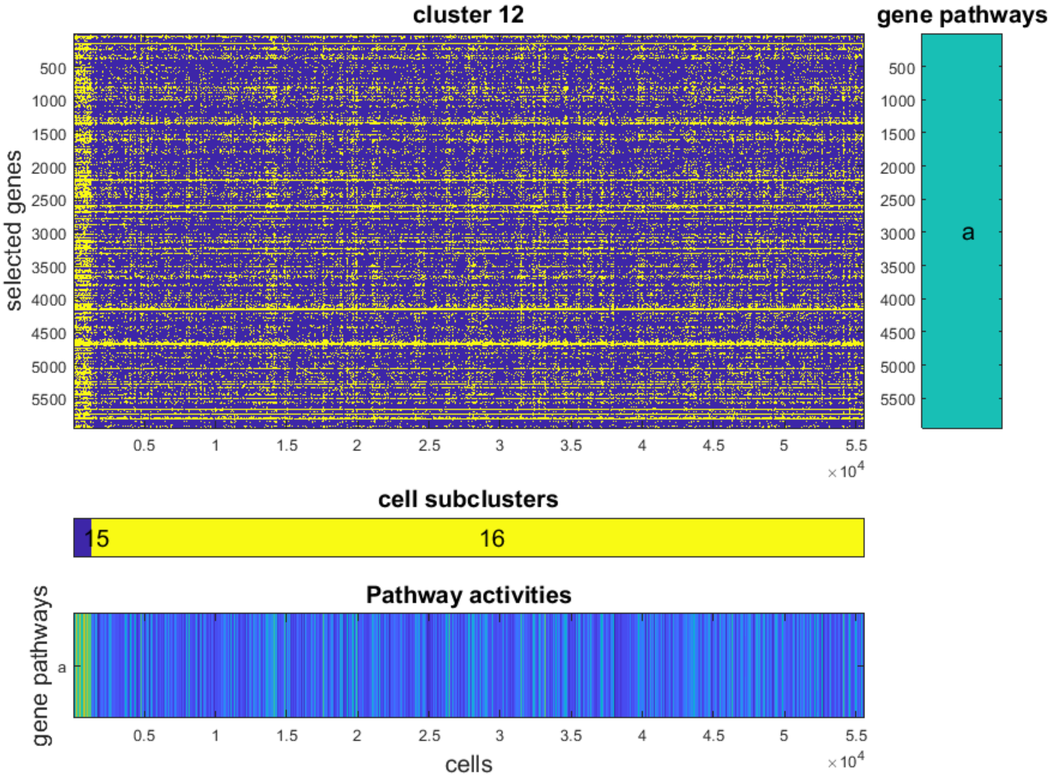


```
Remaining clusters to partition 4
Processing cluster 13 now ...
Processing data subset with 18134 genes and 11598 cells:
Remove genes detected in <100 cells. Remaining 3734 genes.  Elapsed time is 0.782103 seconds.
Iterate 10 random permutations for gene-gene similarity threshold ...   10 Elapsed time is 59.046168 seconds.
Compute gene-gene similarity ... Elapsed time is 2.593122 seconds.
Create gene-gene graph for clustering genes ...
Writing graph into file ... 100%Elapsed time is 2.190421 seconds.
Running ModularityOptimizer for clustering ...Elapsed time is 5.933454 seconds.
Gene-gene graph contains 8 pathways, 3679 genes in total
Elapsed time is 6.045094 seconds.
Create cell-cell graph for clustering cells ...
Writing graph into file ... 100%Elapsed time is 1.364126 seconds.
Running ModularityOptimizer for clustering ...Elapsed time is 8.117596 seconds.
Cell-cell graph contains 23 cell types by community detection
Elapsed time is 8.359558 seconds.
Cell-cell graph contains 17 cell types after merging tiny cell clusters
Cell-cell graph contains 2 cell types after merging
Number of useful pathways is 1
```

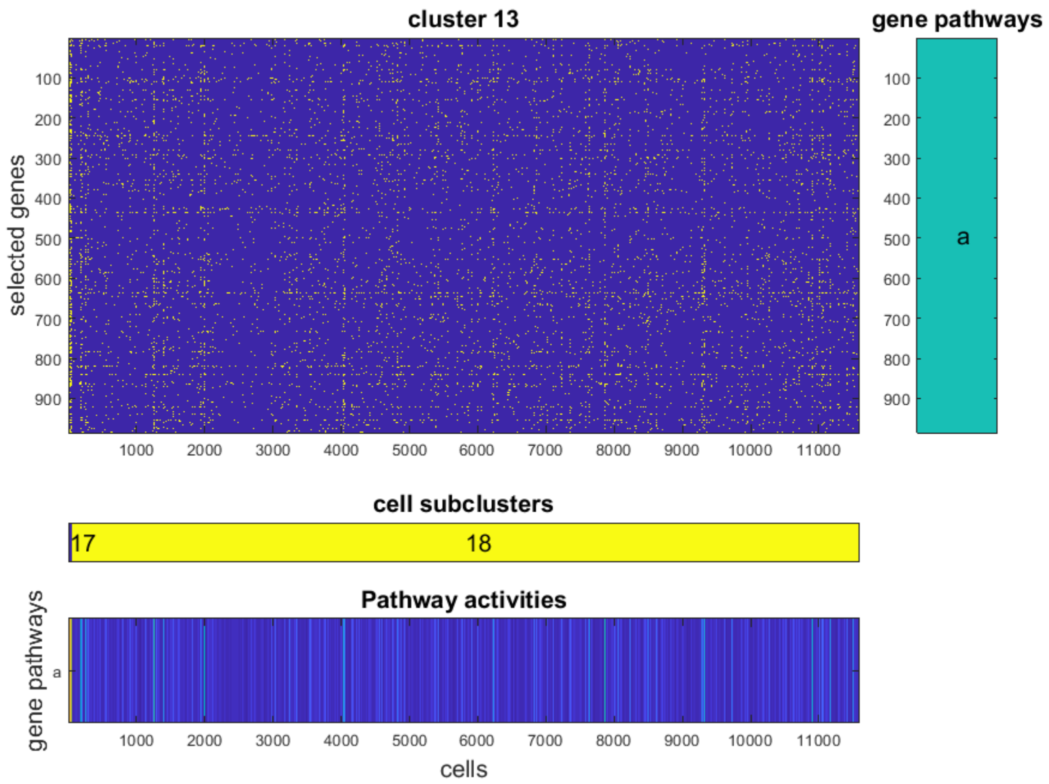


```
Remaining clusters to partition 5
Processing cluster 14 now ...
Processing data subset with 18134 genes and 2108 cells:
Remove genes detected in <100 cells. Remaining 344 genes.  Elapsed time is 0.121338 seconds.
Iterate 10 random permutations for gene-gene similarity threshold ...   10 Elapsed time is 0.579019 seconds.
Compute gene-gene similarity ... Elapsed time is 0.008730 seconds.
Create gene-gene graph for clustering genes ...
Writing graph into file ... 100%Elapsed time is 0.060177 seconds.
Running ModularityOptimizer for clustering ...Elapsed time is 0.366106 seconds.
Gene-gene graph contains 3 pathways, 340 genes in total
Elapsed time is 0.380119 seconds.
Create cell-cell graph for clustering cells ...
Writing graph into file ... 100%Elapsed time is 0.229948 seconds.
Running ModularityOptimizer for clustering ...Elapsed time is 0.974358 seconds.
Cell-cell graph contains 19 cell types by community detection
Elapsed time is 1.023504 seconds.
Cell-cell graph contains 16 cell types after merging tiny cell clusters
Cell-cell graph contains 2 cell types after merging
Number of useful pathways is 1
```

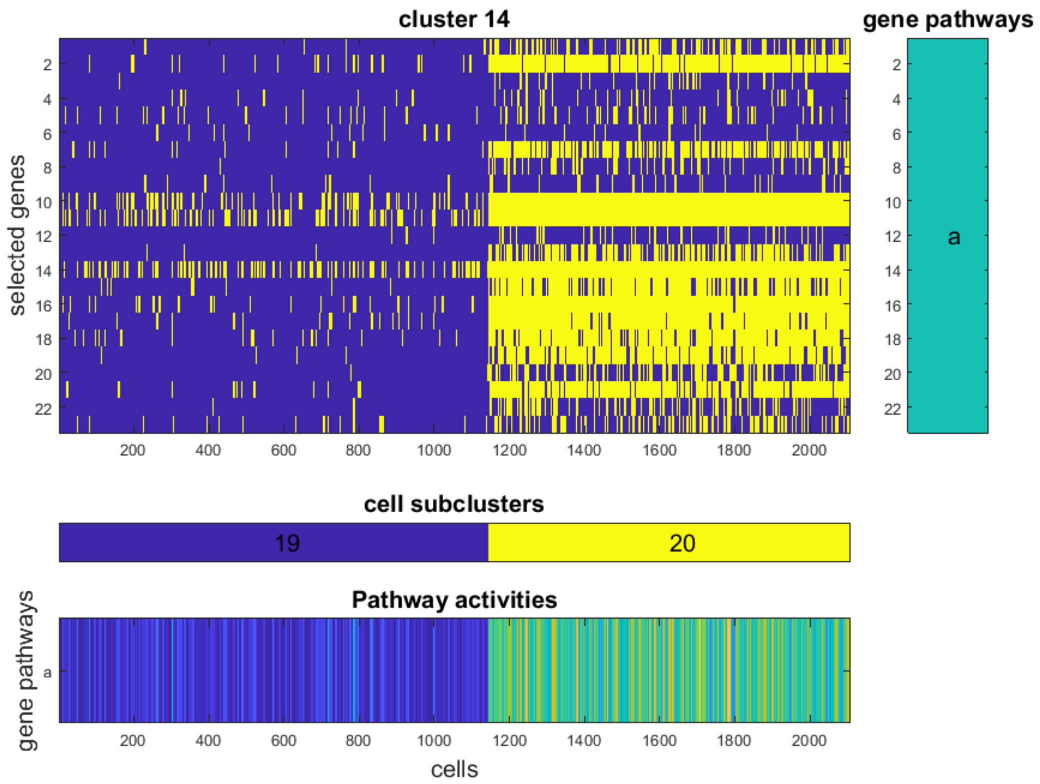


```
Remaining clusters to partition 6
Processing cluster 15 now ...
Processing data subset with 18134 genes and 1287 cells:
Remove genes detected in <100 cells. Remaining 8513 genes.  Elapsed time is 0.096293 seconds.
Iterate 10 random permutations for gene-gene similarity threshold ...   10 Elapsed time is 64.909956 seconds.
Compute gene-gene similarity ... Elapsed time is 4.746057 seconds.
Create gene-gene graph for clustering genes ...
Writing graph into file ... 100%Elapsed time is 4.591258 seconds.
Running ModularityOptimizer for clustering ...Elapsed time is 13.921531 seconds.
Gene-gene graph contains 6 pathways, 7468 genes in total
Elapsed time is 14.222315 seconds.
Create cell-cell graph for clustering cells ...
Writing graph into file ... 100%Elapsed time is 0.140646 seconds.
Running ModularityOptimizer for clustering ...Elapsed time is 0.717089 seconds.
Cell-cell graph contains 14 cell types by community detection
Elapsed time is 0.748220 seconds.
Cell-cell graph contains 14 cell types after merging tiny cell clusters
Cell-cell graph contains 2 cell types after merging
Number of useful pathways is 1
```

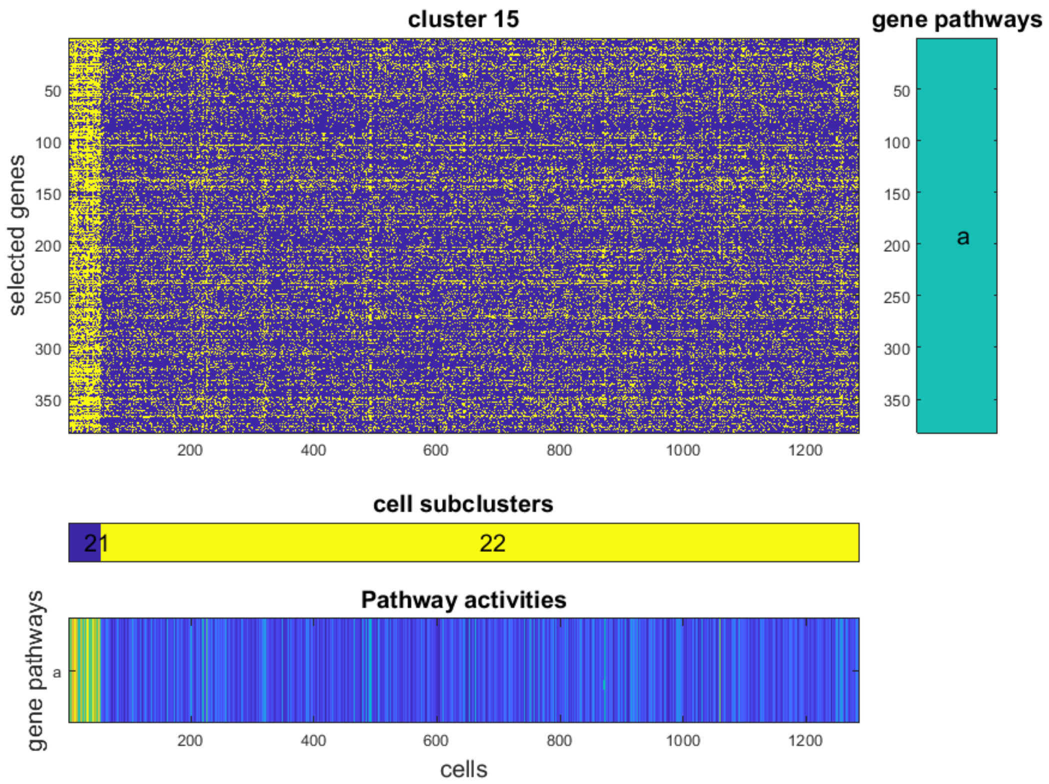


```
Remaining clusters to partition 7
Processing cluster 16 now ...
Processing data subset with 18134 genes and 54342 cells:
Remove genes detected in <100 cells. Remaining 15648 genes.  Elapsed time is 5.026724 seconds.
Iterate 10 random permutations for gene-gene similarity threshold ...   10 Elapsed time is 3005.449154 seconds.
Compute gene-gene similarity ... Elapsed time is 147.262354 seconds.
Create gene-gene graph for clustering genes ...
Writing graph into file ... 100%Elapsed time is 256.926289 seconds.
Running ModularityOptimizer for clustering ...Elapsed time is 772.706233 seconds.
Gene-gene graph contains 5 pathways, 15589 genes in total
Elapsed time is 773.517002 seconds.
Create cell-cell graph for clustering cells ...
Writing graph into file ... 100%Elapsed time is 6.126302 seconds.
Running ModularityOptimizer for clustering ...Elapsed time is 76.154585 seconds.
Cell-cell graph contains 31 cell types by community detection
Elapsed time is 77.298463 seconds.
Cell-cell graph contains 14 cell types after merging tiny cell clusters
creating a total of 13 edges ...     13
Cell-cell graph contains 2 cell types after merging
Number of useful pathways is 1
```

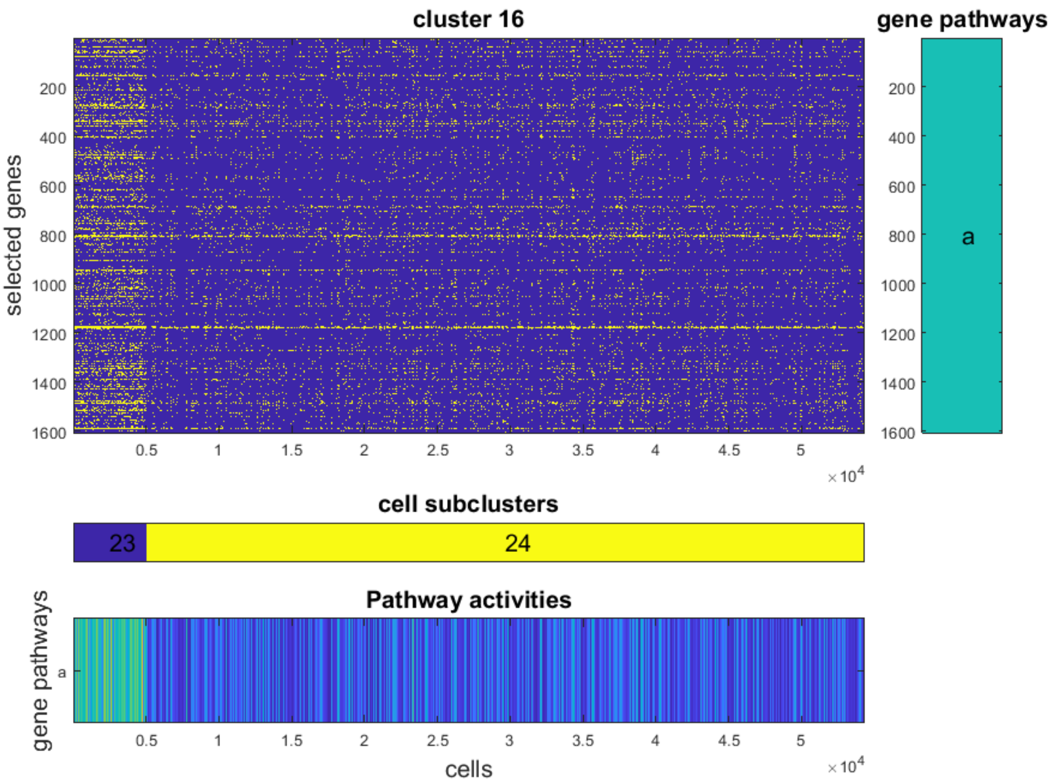


```
Remaining clusters to partition 8
Processing cluster 17 now ...
Processing data subset with 18134 genes and 44 cells:
Remove genes detected in <100 cells. Remaining 0 genes.  Elapsed time is 0.001974 seconds.

Remaining clusters to partition 7
Processing cluster 18 now ...
Processing data subset with 18134 genes and 11554 cells:
Remove genes detected in <100 cells. Remaining 3668 genes.  Elapsed time is 0.750296 seconds.
Iterate 10 random permutations for gene-gene similarity threshold ...   10 Elapsed time is 57.616308 seconds.
Compute gene-gene similarity ... Elapsed time is 2.548790 seconds.
Create gene-gene graph for clustering genes ...
Writing graph into file ... 100%Elapsed time is 1.676554 seconds.
Running ModularityOptimizer for clustering ...Elapsed time is 5.059332 seconds.
Gene-gene graph contains 7 pathways, 3554 genes in total
Elapsed time is 5.163572 seconds.
Create cell-cell graph for clustering cells ...
Writing graph into file ... 100%Elapsed time is 1.348242 seconds.
Running ModularityOptimizer for clustering ...Elapsed time is 7.579616 seconds.
Cell-cell graph contains 21 cell types by community detection
Elapsed time is 7.828465 seconds.
Cell-cell graph contains 15 cell types after merging tiny cell clusters
creating a total of 14 edges ...     14
Cell-cell graph contains 2 cell types after merging
Number of useful pathways is 1
```

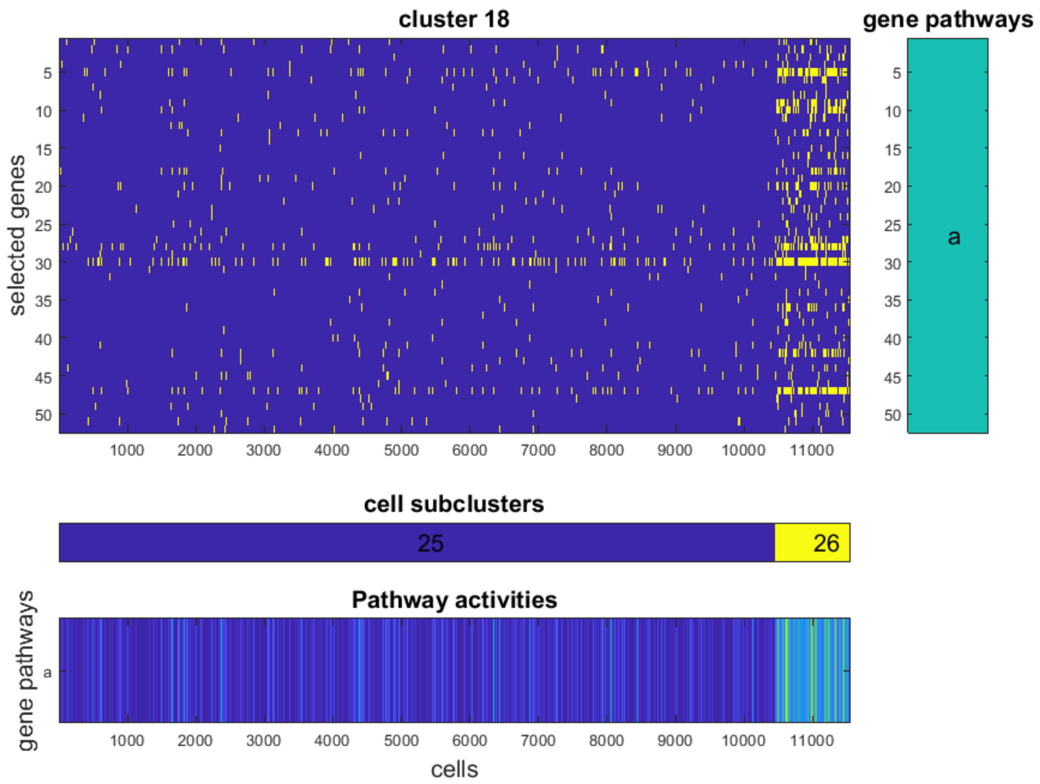


```
Remaining clusters to partition 8
Processing cluster 19 now ...
Processing data subset with 18134 genes and 1146 cells:
Remove genes detected in <100 cells. Remaining 243 genes.  Elapsed time is 0.058154 seconds.
Iterate 10 random permutations for gene-gene similarity threshold ...   10 Elapsed time is 0.191397 seconds.
Compute gene-gene similarity ... Elapsed time is 0.004738 seconds.
Create gene-gene graph for clustering genes ...
Writing graph into file ... 100%Elapsed time is 0.009239 seconds.
Running ModularityOptimizer for clustering ...Elapsed time is 0.264307 seconds.
Gene-gene graph contains 4 pathways, 213 genes in total
Elapsed time is 0.276285 seconds.
Create cell-cell graph for clustering cells ...
Writing graph into file ... 100%Elapsed time is 0.129878 seconds.
Running ModularityOptimizer for clustering ...Elapsed time is 0.766112 seconds.
Cell-cell graph contains 9 cell types by community detection
Elapsed time is 0.795820 seconds.
Cell-cell graph contains 8 cell types after merging tiny cell clusters
Cell-cell graph contains 2 cell types after merging
Number of useful pathways is 1
```

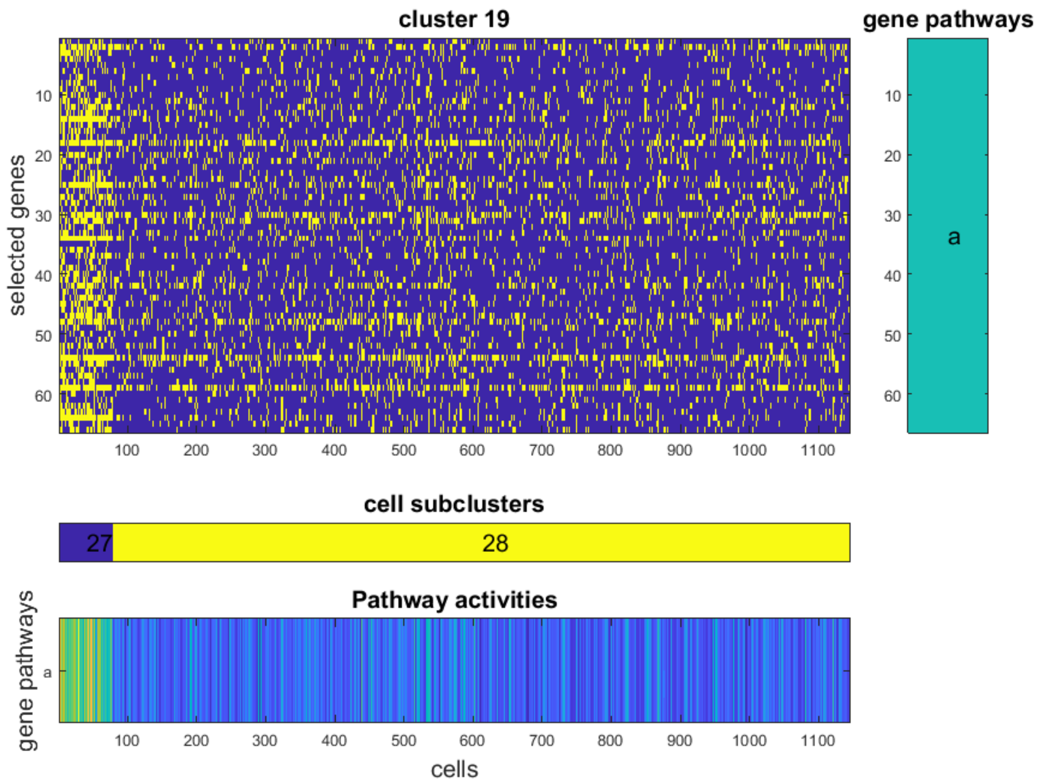


```
Remaining clusters to partition 9
Processing cluster 20 now ...
Processing data subset with 18134 genes and 962 cells:
Remove genes detected in <100 cells. Remaining 118 genes.  Elapsed time is 0.046406 seconds.
Iterate 10 random permutations for gene-gene similarity threshold ...   10 Elapsed time is 0.072420 seconds.
Compute gene-gene similarity ... Elapsed time is 0.001953 seconds.
Create gene-gene graph for clustering genes ...
Writing graph into file ... 100%Elapsed time is 0.005883 seconds.
Running ModularityOptimizer for clustering ...Elapsed time is 0.246861 seconds.
Gene-gene graph contains 3 pathways, 70 genes in total
Elapsed time is 0.254617 seconds.
Create cell-cell graph for clustering cells ...
Writing graph into file ... 100%Elapsed time is 0.105091 seconds.
Running ModularityOptimizer for clustering ...Elapsed time is 0.628520 seconds.
Cell-cell graph contains 10 cell types by community detection
Elapsed time is 0.653147 seconds.
Cell-cell graph contains 9 cell types after merging tiny cell clusters
creating a total of 8 edges ...      8
Cell-cell graph contains 1 cell types after merging

Remaining clusters to partition 8
Processing cluster 21 now ...
Processing data subset with 18134 genes and 52 cells:
Remove genes detected in <100 cells. Remaining 0 genes.  Elapsed time is 0.002106 seconds.

Remaining clusters to partition 7
Processing cluster 22 now ...
Processing data subset with 18134 genes and 1235 cells:
Remove genes detected in <100 cells. Remaining 8361 genes.  Elapsed time is 0.104600 seconds.
Iterate 10 random permutations for gene-gene similarity threshold ...   10 Elapsed time is 60.391135 seconds.
Compute gene-gene similarity ... Elapsed time is 4.512473 seconds.
Create gene-gene graph for clustering genes ...
Writing graph into file ... 100%Elapsed time is 4.142800 seconds.
Running ModularityOptimizer for clustering ...Elapsed time is 10.958474 seconds.
Gene-gene graph contains 5 pathways, 6993 genes in total
Elapsed time is 11.237184 seconds.
Create cell-cell graph for clustering cells ...
Writing graph into file ... 100%Elapsed time is 0.133581 seconds.
Running ModularityOptimizer for clustering ...Elapsed time is 0.659858 seconds.
Cell-cell graph contains 14 cell types by community detection
Elapsed time is 0.691805 seconds.
Cell-cell graph contains 12 cell types after merging tiny cell clusters
creating a total of 11 edges ...     11
Cell-cell graph contains 1 cell types after merging

Remaining clusters to partition 6
Processing cluster 23 now ...
Processing data subset with 18134 genes and 4971 cells:
Remove genes detected in <100 cells. Remaining 10353 genes.  Elapsed time is 0.410028 seconds.
Iterate 10 random permutations for gene-gene similarity threshold ...   10 Elapsed time is 164.176214 seconds.
Compute gene-gene similarity ... Elapsed time is 11.468494 seconds.
Create gene-gene graph for clustering genes ...
Writing graph into file ... 100%Elapsed time is 8.911699 seconds.
Running ModularityOptimizer for clustering ...Elapsed time is 24.631540 seconds.
Gene-gene graph contains 5 pathways, 8837 genes in total
Elapsed time is 25.016785 seconds.
```

```
Create cell-cell graph for clustering cells ...
Writing graph into file ... 100%Elapsed time is 0.566970 seconds.
Running ModularityOptimizer for clustering ...Elapsed time is 2.867408 seconds.
Cell-cell graph contains 19 cell types by community detection
Elapsed time is 2.973019 seconds.
Cell-cell graph contains 16 cell types after merging tiny cell clusters
creating a total of 15 edges ...     15
Cell-cell graph contains 2 cell types after merging
Number of useful pathways is 1
```

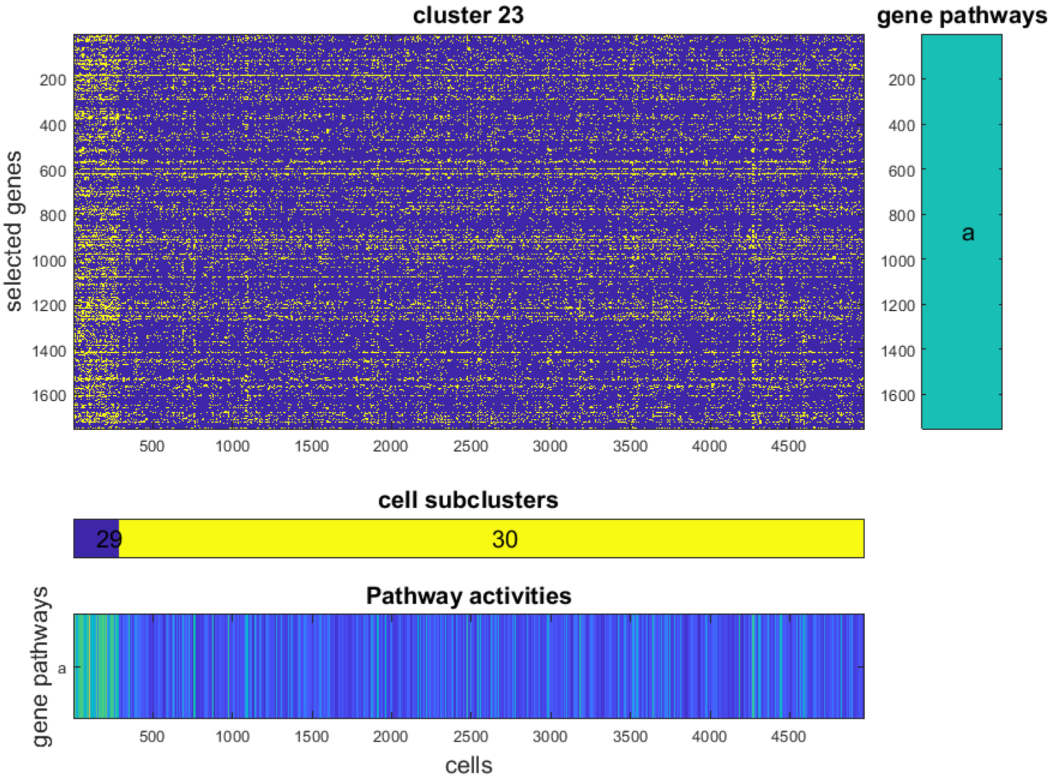


```
Remaining clusters to partition 7
Processing cluster 24 now ...
Processing data subset with 18134 genes and 49371 cells:
Remove genes detected in <100 cells. Remaining 15461 genes.  Elapsed time is 4.499605 seconds.
Iterate 10 random permutations for gene-gene similarity threshold ...   10 Elapsed time is 2796.048185 seconds.
Compute gene-gene similarity ... Elapsed time is 133.876595 seconds.
Create gene-gene graph for clustering genes ...
Writing graph into file ... 100%Elapsed time is 254.897441 seconds.
Running ModularityOptimizer for clustering ...Elapsed time is 978.772277 seconds.
Gene-gene graph contains 5 pathways, 15395 genes in total
Elapsed time is 979.580316 seconds.
Create cell-cell graph for clustering cells ...
Writing graph into file ... 100%Elapsed time is 5.555722 seconds.
Running ModularityOptimizer for clustering ...Elapsed time is 60.505001 seconds.
Cell-cell graph contains 26 cell types by community detection
Elapsed time is 61.561163 seconds.
Cell-cell graph contains 19 cell types after merging tiny cell clusters
creating a total of 18 edges ...     18
Cell-cell graph contains 2 cell types after merging
Number of useful pathways is 1
```

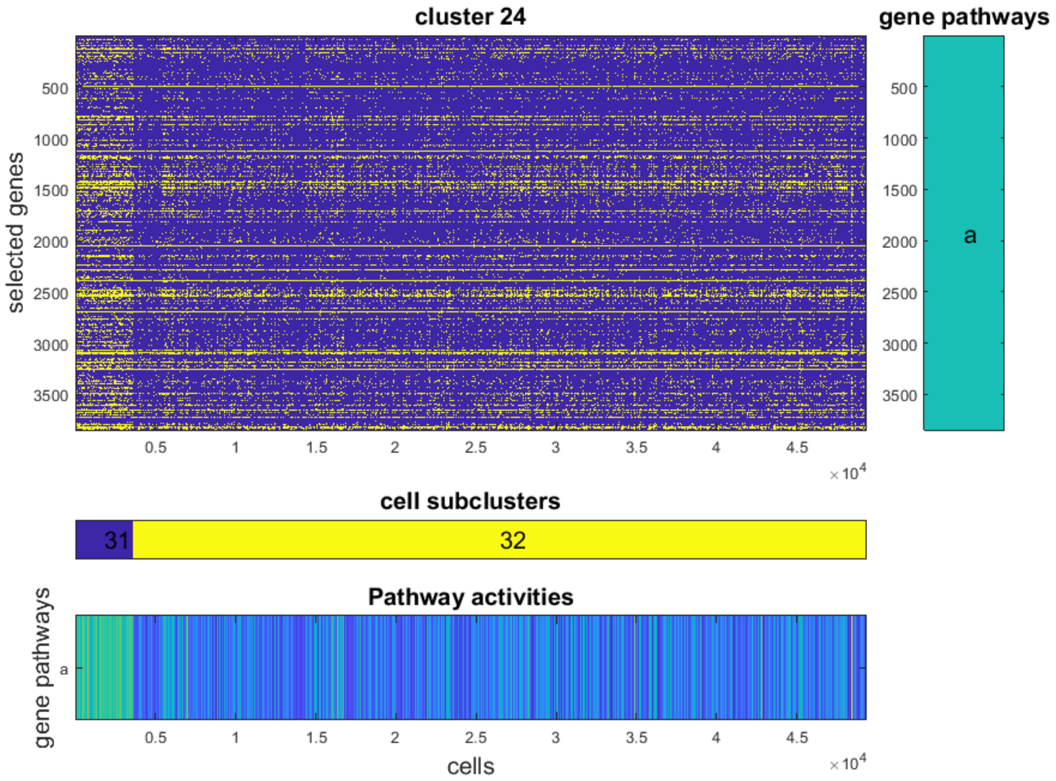


```
Remaining clusters to partition 8
Processing cluster 25 now ...
Processing data subset with 18134 genes and 10454 cells:
Remove genes detected in <100 cells. Remaining 3482 genes.  Elapsed time is 0.757205 seconds.
Iterate 10 random permutations for gene-gene similarity threshold ...   10 Elapsed time is 48.706415 seconds.
Compute gene-gene similarity ... Elapsed time is 2.120067 seconds.
Create gene-gene graph for clustering genes ...
Writing graph into file ... 100%Elapsed time is 1.840834 seconds.
Running ModularityOptimizer for clustering ...Elapsed time is 5.594687 seconds.
Gene-gene graph contains 6 pathways, 3402 genes in total
Elapsed time is 5.696186 seconds.
Create cell-cell graph for clustering cells ...
Writing graph into file ... 100%Elapsed time is 1.216855 seconds.
Running ModularityOptimizer for clustering ...Elapsed time is 8.120211 seconds.
Cell-cell graph contains 23 cell types by community detection
Elapsed time is 8.343805 seconds.
Cell-cell graph contains 18 cell types after merging tiny cell clusters
creating a total of 17 edges ...     17
Cell-cell graph contains 2 cell types after merging
Number of useful pathways is 1
```

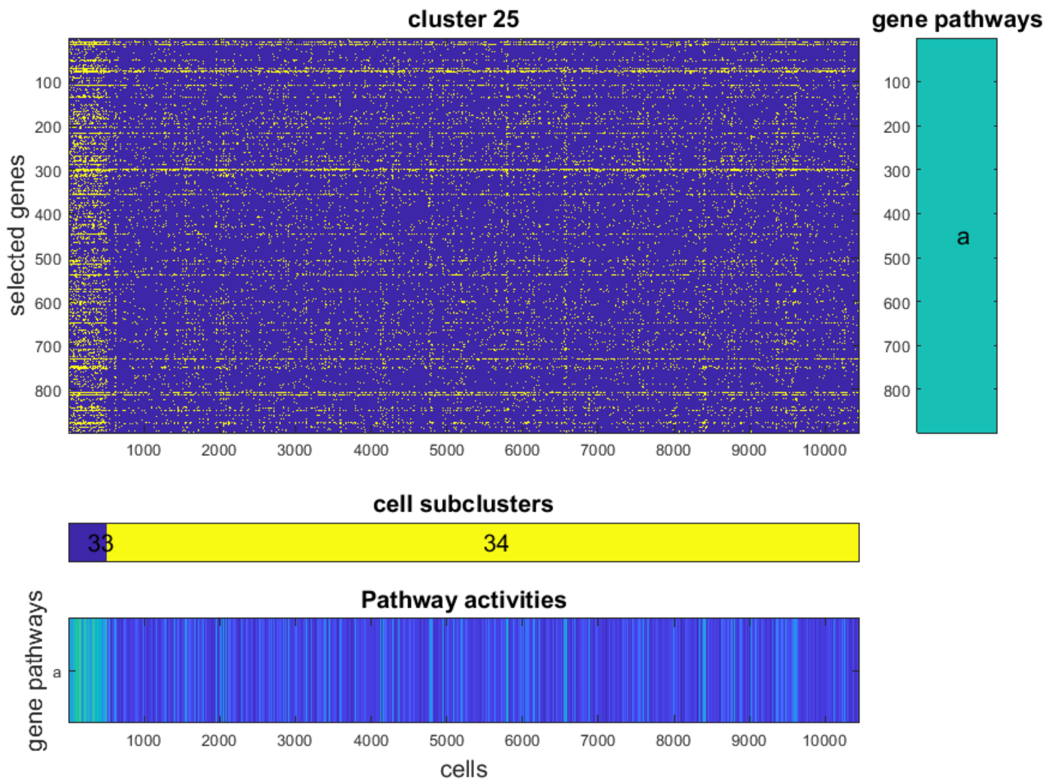


```
Remaining clusters to partition 9
Processing cluster 26 now ...
Processing data subset with 18134 genes and 1100 cells:
Remove genes detected in <100 cells. Remaining 267 genes.  Elapsed time is 0.057992 seconds.
Iterate 10 random permutations for gene-gene similarity threshold ...   10 Elapsed time is 0.206391 seconds.
Compute gene-gene similarity ... Elapsed time is 0.003976 seconds.
Create gene-gene graph for clustering genes ...
Writing graph into file ... 100%Elapsed time is 0.016836 seconds.
Running ModularityOptimizer for clustering ...Elapsed time is 0.261499 seconds.
Gene-gene graph contains 3 pathways, 219 genes in total
Elapsed time is 0.273755 seconds.
Create cell-cell graph for clustering cells ...
Writing graph into file ... 100%Elapsed time is 0.120656 seconds.
Running ModularityOptimizer for clustering ...Elapsed time is 0.629047 seconds.
Cell-cell graph contains 10 cell types by community detection
Elapsed time is 0.657107 seconds.
Cell-cell graph contains 10 cell types after merging tiny cell clusters
creating a total of 9 edges ...      9
Cell-cell graph contains 1 cell types after merging

Remaining clusters to partition 8
Processing cluster 27 now ...
Processing data subset with 18134 genes and 79 cells:
Remove genes detected in <100 cells. Remaining 0 genes.  Elapsed time is 0.004393 seconds.

Remaining clusters to partition 7
Processing cluster 28 now ...
Processing data subset with 18134 genes and 1067 cells:
Remove genes detected in <100 cells. Remaining 208 genes.  Elapsed time is 0.055785 seconds.
Iterate 10 random permutations for gene-gene similarity threshold ...   10 Elapsed time is 0.149241 seconds.
Compute gene-gene similarity ... Elapsed time is 0.003559 seconds.
Create gene-gene graph for clustering genes ...
Writing graph into file ... 100%Elapsed time is 0.006719 seconds.
Running ModularityOptimizer for clustering ...Elapsed time is 0.223277 seconds.
Gene-gene graph contains 2 pathways, 96 genes in total
Elapsed time is 0.233342 seconds.
Create cell-cell graph for clustering cells ...
Writing graph into file ... 100%Elapsed time is 0.121558 seconds.
Running ModularityOptimizer for clustering ...Elapsed time is 0.679101 seconds.
Cell-cell graph contains 10 cell types by community detection
Elapsed time is 0.706533 seconds.
Cell-cell graph contains 2 cell types after merging tiny cell clusters
creating a total of 1 edges ...      1
Cell-cell graph contains 1 cell types after merging

Remaining clusters to partition 6
Processing cluster 29 now ...
Processing data subset with 18134 genes and 290 cells:
Remove genes detected in <100 cells. Remaining 2230 genes.  Elapsed time is 0.020240 seconds.
Iterate 10 random permutations for gene-gene similarity threshold ...   10 Elapsed time is 3.641899 seconds.
Compute gene-gene similarity ... Elapsed time is 0.282134 seconds.
Create gene-gene graph for clustering genes ...
Writing graph into file ... 100%Elapsed time is 0.078483 seconds.
Running ModularityOptimizer for clustering ...Elapsed time is 0.483440 seconds.
Gene-gene graph contains 4 pathways, 781 genes in total
Elapsed time is 0.544681 seconds.
```

```
Create cell-cell graph for clustering cells ...
Writing graph into file ... 100%Elapsed time is 0.033195 seconds.
Running ModularityOptimizer for clustering ...Elapsed time is 0.306888 seconds.
Cell-cell graph contains 6 cell types by community detection
Elapsed time is 0.319583 seconds.
Cell-cell graph contains 5 cell types after merging tiny cell clusters
Cell-cell graph contains 2 cell types after merging
Number of useful pathways is 2
```

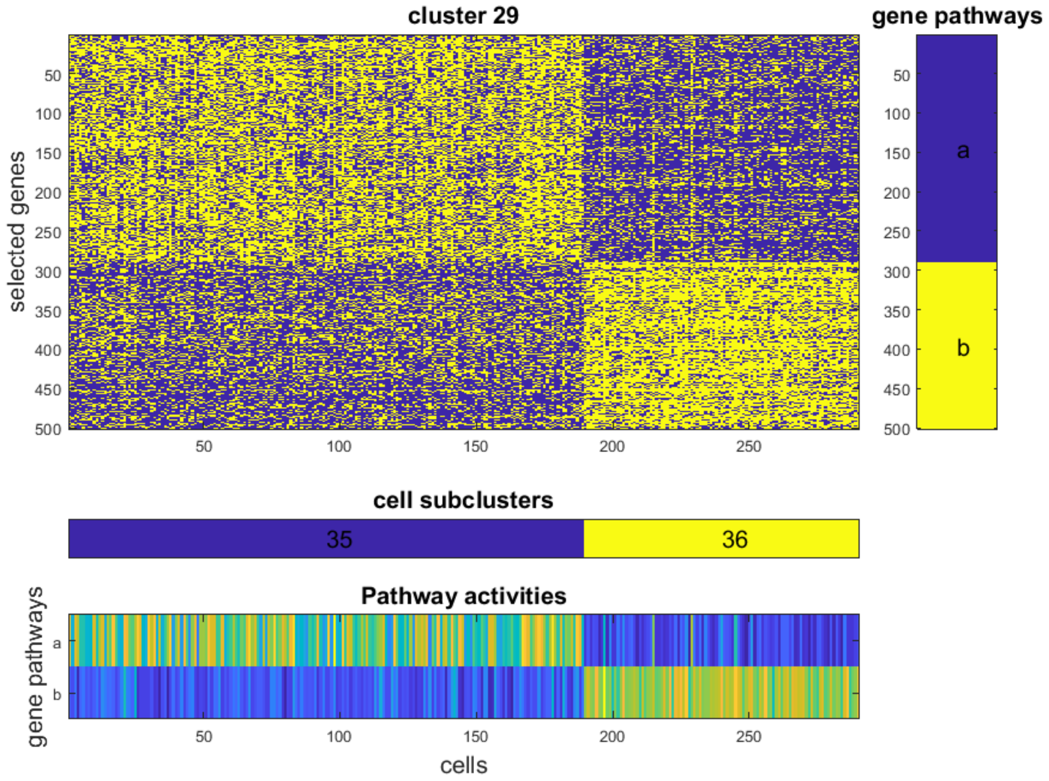


```
Remaining clusters to partition 7
Processing cluster 30 now ...
Processing data subset with 18134 genes and 4681 cells:
Remove genes detected in <100 cells. Remaining 10109 genes.  Elapsed time is 0.407129 seconds.
Iterate 10 random permutations for gene-gene similarity threshold ...   10 Elapsed time is 151.378239 seconds.
Compute gene-gene similarity ... Elapsed time is 10.666570 seconds.
Create gene-gene graph for clustering genes ...
Writing graph into file ... 100%Elapsed time is 7.271046 seconds.
Running ModularityOptimizer for clustering ...Elapsed time is 23.212090 seconds.
Gene-gene graph contains 4 pathways, 8322 genes in total
Elapsed time is 23.591864 seconds.
Create cell-cell graph for clustering cells ...
Writing graph into file ... 100%Elapsed time is 0.520213 seconds.
Running ModularityOptimizer for clustering ...Elapsed time is 2.755017 seconds.
Cell-cell graph contains 18 cell types by community detection
Elapsed time is 2.855063 seconds.
Cell-cell graph contains 14 cell types after merging tiny cell clusters
creating a total of 13 edges ...     13
Cell-cell graph contains 2 cell types after merging
Number of useful pathways is 1
```

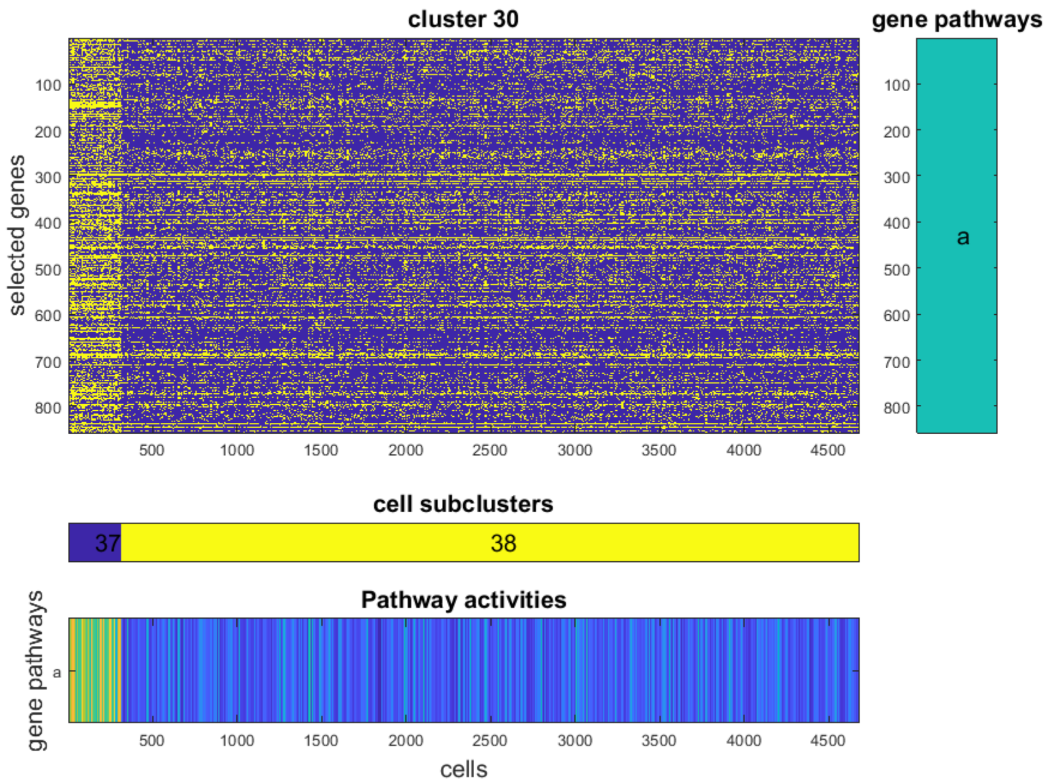


```
Remaining clusters to partition 8
Processing cluster 31 now ...
Processing data subset with 18134 genes and 3602 cells:
Remove genes detected in <100 cells. Remaining 10699 genes.  Elapsed time is 0.286150 seconds.
Iterate 10 random permutations for gene-gene similarity threshold ...   10 Elapsed time is 148.561742 seconds.
Compute gene-gene similarity ... Elapsed time is 10.577848 seconds.
Create gene-gene graph for clustering genes ...
Writing graph into file ... 100%Elapsed time is 4.615611 seconds.
Running ModularityOptimizer for clustering ...Elapsed time is 13.689132 seconds.
Gene-gene graph contains 5 pathways, 7626 genes in total
Elapsed time is 14.088136 seconds.
Create cell-cell graph for clustering cells ...
Writing graph into file ... 100%Elapsed time is 0.412923 seconds.
Running ModularityOptimizer for clustering ...Elapsed time is 1.876546 seconds.
Cell-cell graph contains 17 cell types by community detection
Elapsed time is 1.958236 seconds.
Cell-cell graph contains 17 cell types after merging tiny cell clusters
creating a total of 16 edges ...     16
Cell-cell graph contains 2 cell types after merging
Number of useful pathways is 1
```

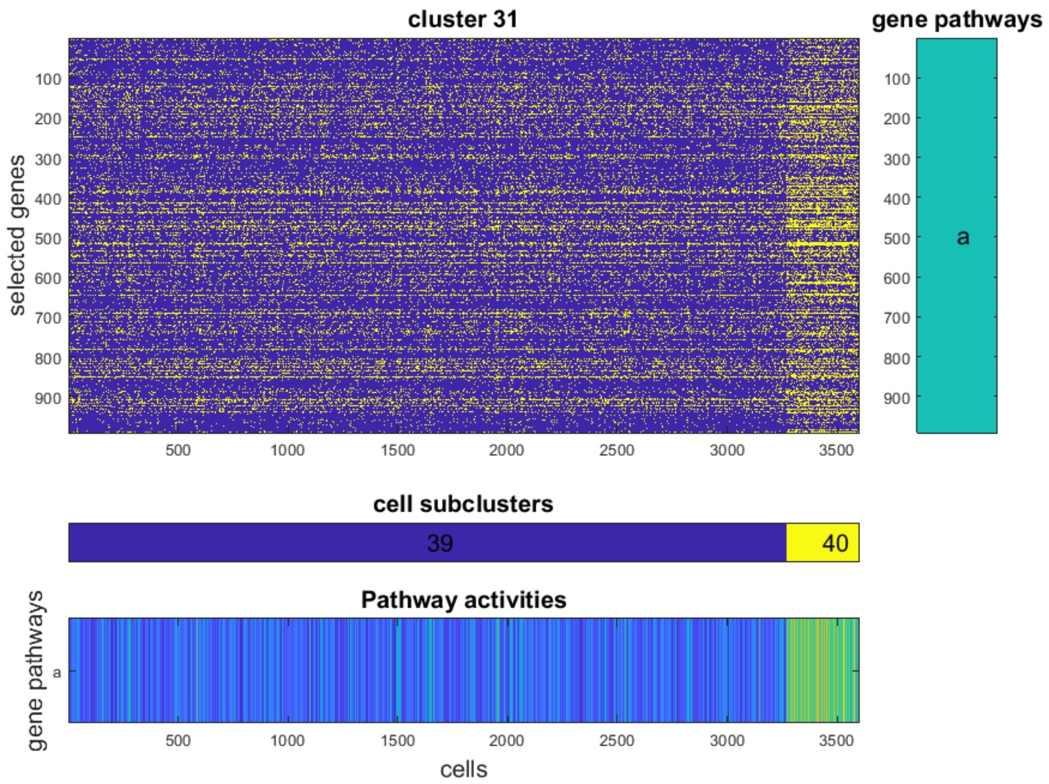


```
Remaining clusters to partition 9
Processing cluster 32 now ...
Processing data subset with 18134 genes and 45769 cells:
Remove genes detected in <100 cells. Remaining 15254 genes.  Elapsed time is 4.156302 seconds.
Iterate 10 random permutations for gene-gene similarity threshold ...   10 Elapsed time is 2455.119949 seconds.
Compute gene-gene similarity ... Elapsed time is 121.551239 seconds.
Create gene-gene graph for clustering genes ...
Writing graph into file ... 100%Elapsed time is 253.328710 seconds.
Running ModularityOptimizer for clustering ...Elapsed time is 727.721803 seconds.
Gene-gene graph contains 6 pathways, 15197 genes in total
Elapsed time is 728.483141 seconds.
Create cell-cell graph for clustering cells ...
Writing graph into file ... 100%Elapsed time is 5.232440 seconds.
Running ModularityOptimizer for clustering ...Elapsed time is 69.368112 seconds.
Cell-cell graph contains 35 cell types by community detection
Elapsed time is 70.295436 seconds.
Cell-cell graph contains 12 cell types after merging tiny cell clusters
creating a total of 11 edges ...     11
Cell-cell graph contains 2 cell types after merging
Number of useful pathways is 1
```

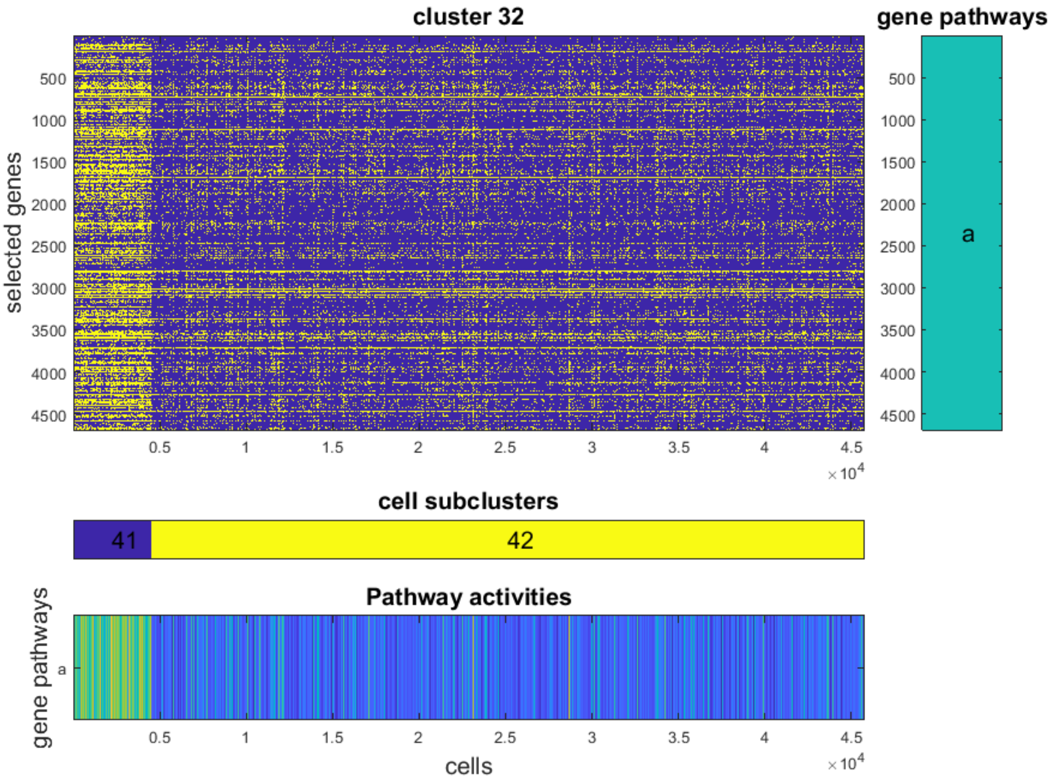


```
Remaining clusters to partition 10
Processing cluster 33 now ...
Processing data subset with 18134 genes and 509 cells:
Remove genes detected in <100 cells. Remaining 556 genes.  Elapsed time is 0.024116 seconds.
Iterate 10 random permutations for gene-gene similarity threshold ...   10 Elapsed time is 0.367013 seconds.
Compute gene-gene similarity ... Elapsed time is 0.016971 seconds.
Create gene-gene graph for clustering genes ...
Writing graph into file ... 100%Elapsed time is 0.016950 seconds.
Running ModularityOptimizer for clustering ...Elapsed time is 0.272046 seconds.
Gene-gene graph contains 5 pathways, 433 genes in total
Elapsed time is 0.289551 seconds.
Create cell-cell graph for clustering cells ...
Writing graph into file ... 100%Elapsed time is 0.057556 seconds.
Running ModularityOptimizer for clustering ...Elapsed time is 0.402396 seconds.
Cell-cell graph contains 8 cell types by community detection
Elapsed time is 0.417977 seconds.
Cell-cell graph contains 8 cell types after merging tiny cell clusters
Cell-cell graph contains 3 cell types after merging
Number of useful pathways is 2
```

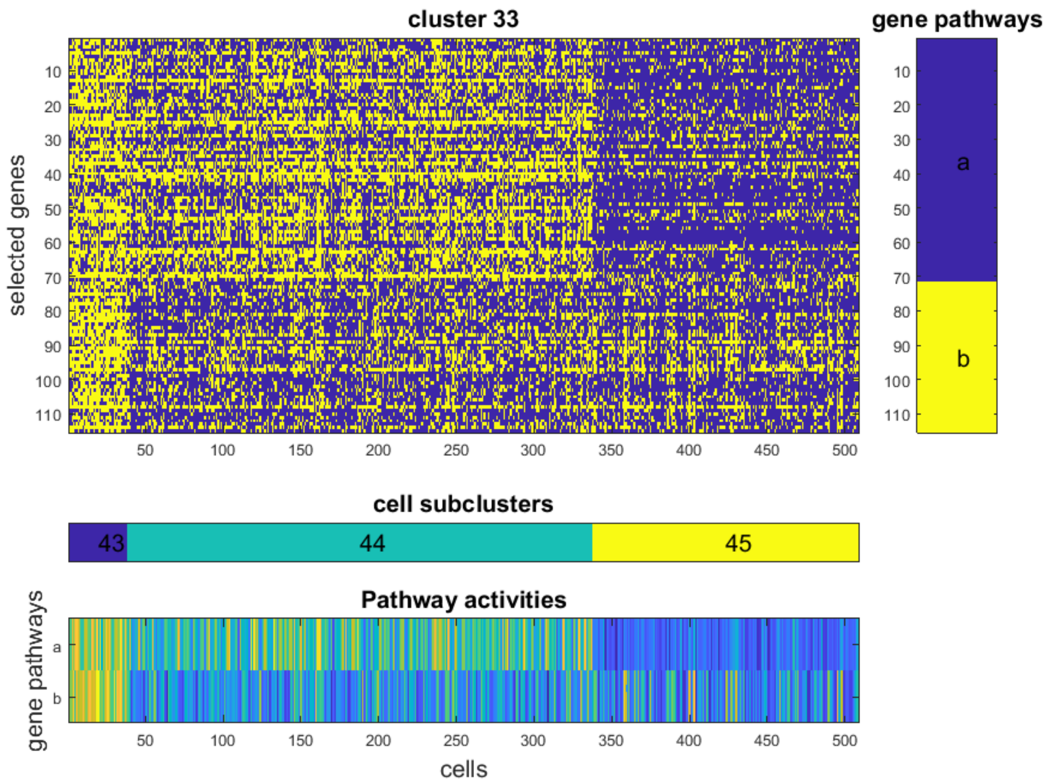


```
Remaining clusters to partition 12
Processing cluster 34 now ...
Processing data subset with 18134 genes and 9945 cells:
Remove genes detected in <100 cells. Remaining 2798 genes.  Elapsed time is 0.654831 seconds.
Iterate 10 random permutations for gene-gene similarity threshold ...   10 Elapsed time is 34.089562 seconds.
Compute gene-gene similarity ... Elapsed time is 1.407046 seconds.
Create gene-gene graph for clustering genes ...
Writing graph into file ... 100%Elapsed time is 0.501310 seconds.
Running ModularityOptimizer for clustering ...Elapsed time is 1.559566 seconds.
Gene-gene graph contains 6 pathways, 2328 genes in total
Elapsed time is 1.635645 seconds.
Create cell-cell graph for clustering cells ...
Writing graph into file ... 100%Elapsed time is 1.164076 seconds.
Running ModularityOptimizer for clustering ...Elapsed time is 7.357852 seconds.
Cell-cell graph contains 18 cell types by community detection
Elapsed time is 7.568526 seconds.
Cell-cell graph contains 14 cell types after merging tiny cell clusters
creating a total of 13 edges ...     13
Cell-cell graph contains 2 cell types after merging
Number of useful pathways is 1
```

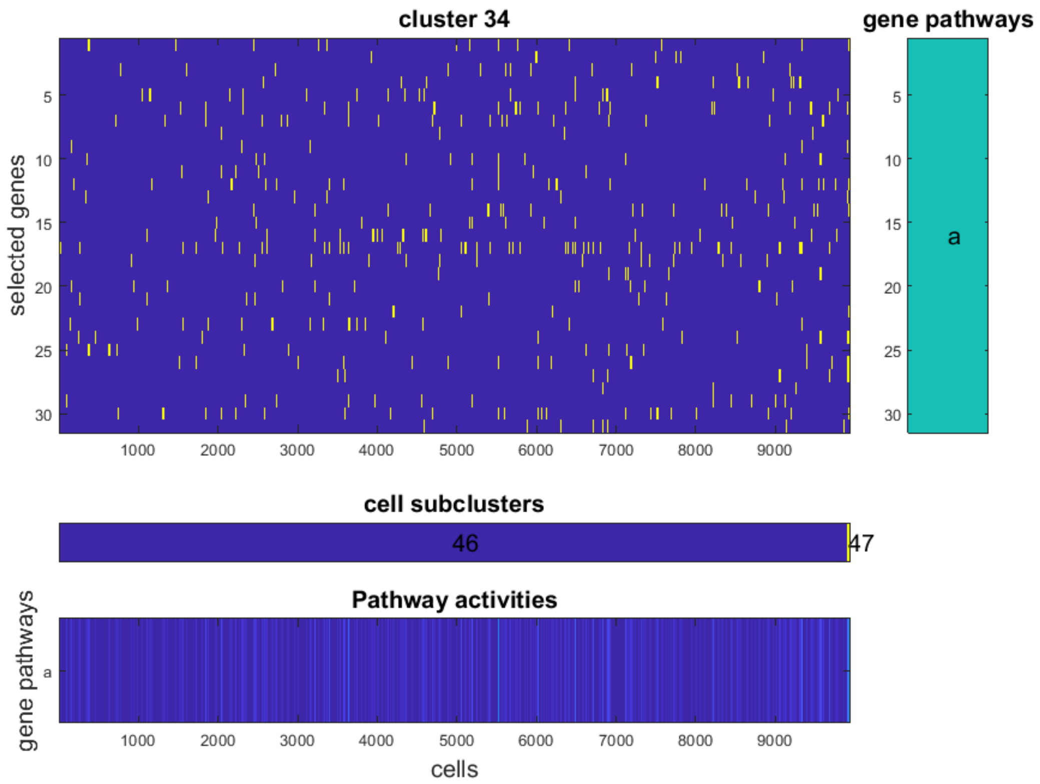


```
Remaining clusters to partition 13
Processing cluster 35 now ...
Processing data subset with 18134 genes and 189 cells:
Remove genes detected in <100 cells. Remaining 0 genes.  Elapsed time is 0.008704 seconds.

Remaining clusters to partition 12
Processing cluster 36 now ...
Processing data subset with 18134 genes and 101 cells:
Remove genes detected in <100 cells. Remaining 0 genes.  Elapsed time is 0.005396 seconds.

Remaining clusters to partition 11
Processing cluster 37 now ...
Processing data subset with 18134 genes and 314 cells:
Remove genes detected in <100 cells. Remaining 1726 genes.  Elapsed time is 0.019665 seconds.
Iterate 10 random permutations for gene-gene similarity threshold ...   10 Elapsed time is 2.316723 seconds.
Compute gene-gene similarity ... Elapsed time is 0.183789 seconds.
Create gene-gene graph for clustering genes ...
Writing graph into file ... 100%Elapsed time is 0.052778 seconds.
Running ModularityOptimizer for clustering ...Elapsed time is 0.422785 seconds.
Gene-gene graph contains 4 pathways, 548 genes in total
Elapsed time is 0.470061 seconds.
Create cell-cell graph for clustering cells ...
Writing graph into file ... 100%Elapsed time is 0.034203 seconds.
Running ModularityOptimizer for clustering ...Elapsed time is 0.318267 seconds.
Cell-cell graph contains 6 cell types by community detection
Elapsed time is 0.330468 seconds.
Cell-cell graph contains 6 cell types after merging tiny cell clusters
Cell-cell graph contains 2 cell types after merging
Number of useful pathways is 2
```

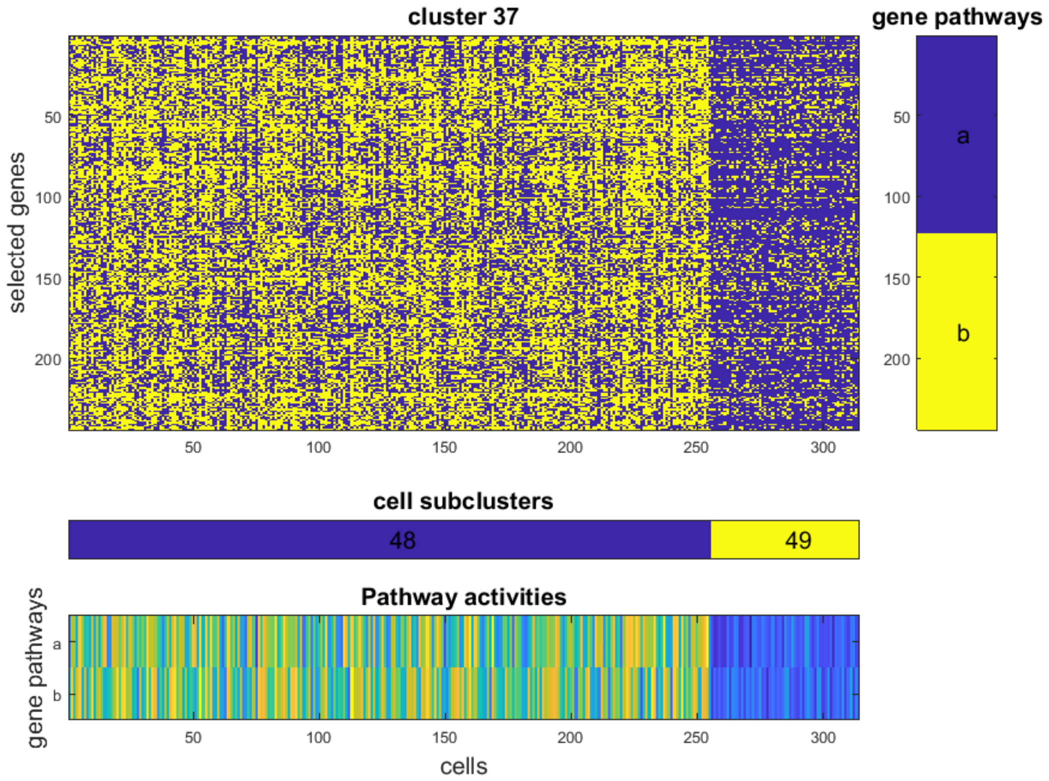


```
Remaining clusters to partition 12
Processing cluster 38 now ...
Processing data subset with 18134 genes and 4367 cells:
Remove genes detected in <100 cells. Remaining 9947 genes.  Elapsed time is 0.374551 seconds.
Iterate 10 random permutations for gene-gene similarity threshold ...   10 Elapsed time is 141.209698 seconds.
Compute gene-gene similarity ... Elapsed time is 9.915393 seconds.
Create gene-gene graph for clustering genes ...
Writing graph into file ... 100%Elapsed time is 6.122351 seconds.
Running ModularityOptimizer for clustering ...Elapsed time is 15.548015 seconds.
Gene-gene graph contains 5 pathways, 7900 genes in total
Elapsed time is 15.908004 seconds.
Create cell-cell graph for clustering cells ...
Writing graph into file ... 100%Elapsed time is 0.508195 seconds.
Running ModularityOptimizer for clustering ...Elapsed time is 2.650876 seconds.
Cell-cell graph contains 15 cell types by community detection
Elapsed time is 2.745793 seconds.
Cell-cell graph contains 12 cell types after merging tiny cell clusters
Cell-cell graph contains 2 cell types after merging
Number of useful pathways is 1
```

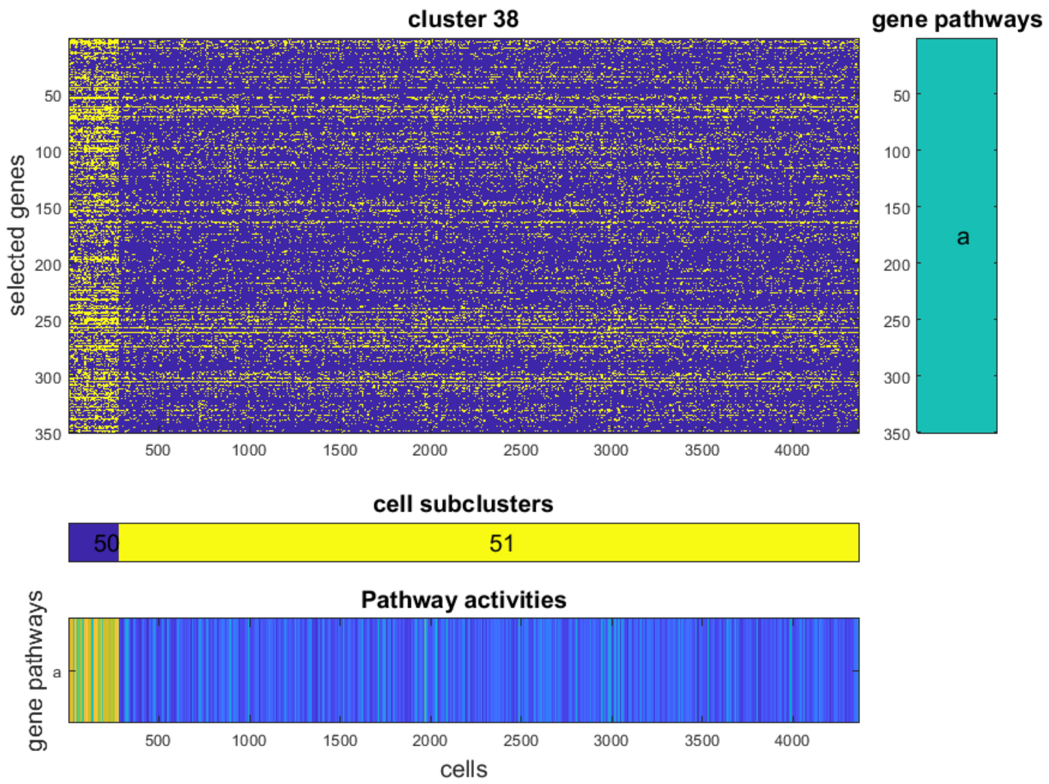


```
Remaining clusters to partition 13
Processing cluster 39 now ...
Processing data subset with 18134 genes and 3267 cells:
Remove genes detected in <100 cells. Remaining 10450 genes.  Elapsed time is 0.295430 seconds.
Iterate 10 random permutations for gene-gene similarity threshold ...   10 Elapsed time is 132.488025 seconds.
Compute gene-gene similarity ... Elapsed time is 9.693341 seconds.
Create gene-gene graph for clustering genes ...
Writing graph into file ... 100%Elapsed time is 4.269834 seconds.
Running ModularityOptimizer for clustering ...Elapsed time is 14.797010 seconds.
Gene-gene graph contains 6 pathways, 7246 genes in total
Elapsed time is 15.182151 seconds.
Create cell-cell graph for clustering cells ...
Writing graph into file ... 100%Elapsed time is 0.370005 seconds.
Running ModularityOptimizer for clustering ...Elapsed time is 1.768895 seconds.
Cell-cell graph contains 14 cell types by community detection
Elapsed time is 1.842662 seconds.
Cell-cell graph contains 13 cell types after merging tiny cell clusters
Cell-cell graph contains 2 cell types after merging
Number of useful pathways is 1
```

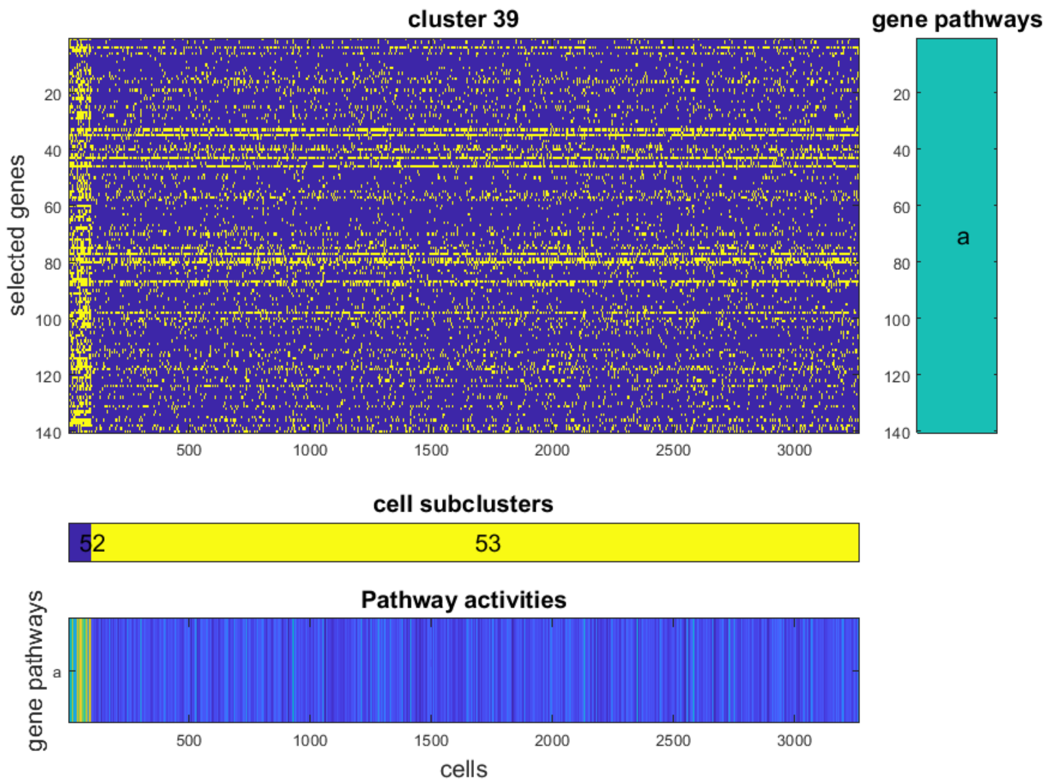


```
Remaining clusters to partition 14
Processing cluster 40 now ...
Processing data subset with 18134 genes and 335 cells:
Remove genes detected in <100 cells. Remaining 2140 genes.  Elapsed time is 0.022650 seconds.
Iterate 10 random permutations for gene-gene similarity threshold ...   10 Elapsed time is 3.462753 seconds.
Compute gene-gene similarity ... Elapsed time is 0.272574 seconds.
Create gene-gene graph for clustering genes ...
Writing graph into file ... 100%Elapsed time is 0.037808 seconds.
Running ModularityOptimizer for clustering ...Elapsed time is 0.314917 seconds.
Gene-gene graph contains 2 pathways, 69 genes in total
Elapsed time is 0.371355 seconds.
Create cell-cell graph for clustering cells ...
Writing graph into file ... 100%Elapsed time is 0.036703 seconds.
Running ModularityOptimizer for clustering ...Elapsed time is 0.340256 seconds.
Cell-cell graph contains 5 cell types by community detection
Elapsed time is 0.353074 seconds.
Cell-cell graph contains 2 cell types after merging tiny cell clusters
creating a total of 1 edges ...      1
Cell-cell graph contains 1 cell types after merging

Remaining clusters to partition 13
Processing cluster 41 now ...
Processing data subset with 18134 genes and 4501 cells:
Remove genes detected in <100 cells. Remaining 10973 genes.  Elapsed time is 0.366584 seconds.
Iterate 10 random permutations for gene-gene similarity threshold ...   10 Elapsed time is 170.461659 seconds.
Compute gene-gene similarity ... Elapsed time is 11.993361 seconds.
Create gene-gene graph for clustering genes ...
Writing graph into file ... 100%Elapsed time is 27.516088 seconds.
Running ModularityOptimizer for clustering ...Elapsed time is 77.354188 seconds.
Gene-gene graph contains 3 pathways, 10582 genes in total
Elapsed time is 77.775991 seconds.
Create cell-cell graph for clustering cells ...
Writing graph into file ... 100%Elapsed time is 0.479469 seconds.
Running ModularityOptimizer for clustering ...Elapsed time is 2.100371 seconds.
Cell-cell graph contains 16 cell types by community detection
Elapsed time is 2.193664 seconds.
Cell-cell graph contains 13 cell types after merging tiny cell clusters
creating a total of 12 edges ...     12
Cell-cell graph contains 1 cell types after merging

Remaining clusters to partition 12
Processing cluster 42 now ...
Processing data subset with 18134 genes and 41268 cells:
Remove genes detected in <100 cells. Remaining 14863 genes.  Elapsed time is 3.724059 seconds.
Iterate 10 random permutations for gene-gene similarity threshold ...   10 Elapsed time is 2053.127522 seconds.
Compute gene-gene similarity ... Elapsed time is 106.984402 seconds.
Create gene-gene graph for clustering genes ...
Writing graph into file ... 100%Elapsed time is 159.775255 seconds.
Running ModularityOptimizer for clustering ...Elapsed time is 500.769086 seconds.
Gene-gene graph contains 5 pathways, 14756 genes in total
Elapsed time is 501.465295 seconds.
Create cell-cell graph for clustering cells ...
Writing graph into file ... 100%Elapsed time is 4.707293 seconds.
Running ModularityOptimizer for clustering ...Elapsed time is 52.640319 seconds.
Cell-cell graph contains 22 cell types by community detection
Elapsed time is 53.449553 seconds.
```

```
Cell-cell graph contains 15 cell types after merging tiny cell clusters
creating a total of 14 edges ...     14
Cell-cell graph contains 2 cell types after merging
Number of useful pathways is 1
```

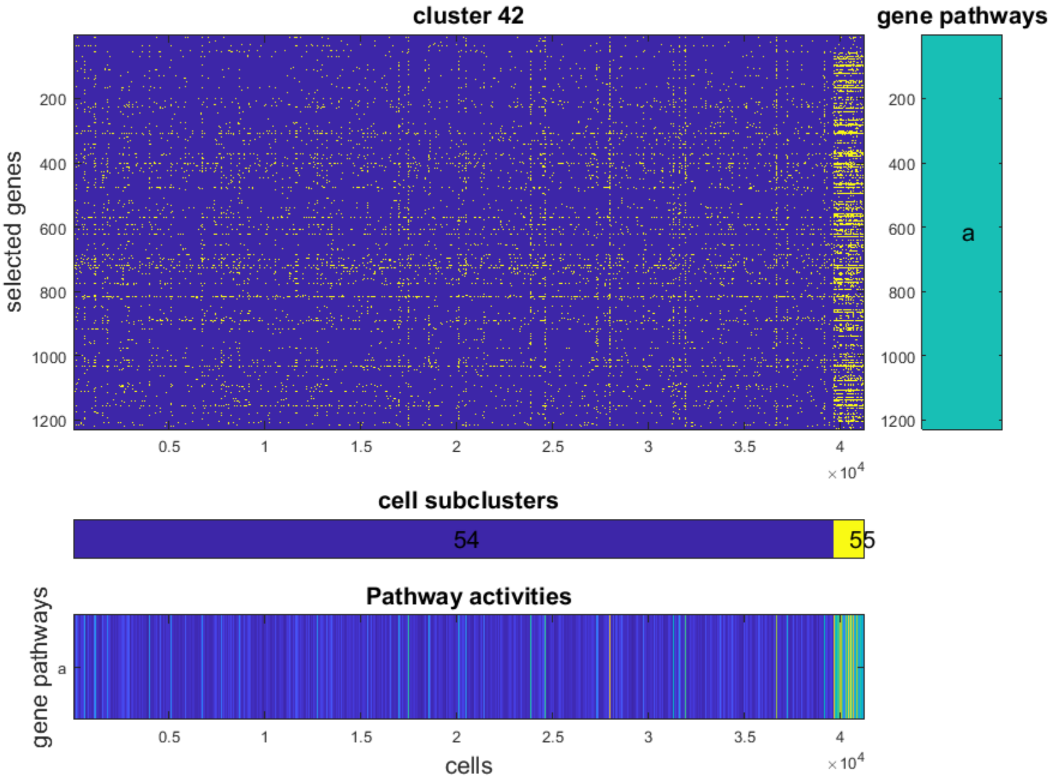


```
Remaining clusters to partition 13
Processing cluster 43 now ...
Processing data subset with 18134 genes and 38 cells:
Remove genes detected in <100 cells. Remaining 0 genes.  Elapsed time is 0.001449 seconds.

Remaining clusters to partition 12
Processing cluster 44 now ...
Processing data subset with 18134 genes and 299 cells:
Remove genes detected in <100 cells. Remaining 226 genes.  Elapsed time is 0.015708 seconds.
Iterate 10 random permutations for gene-gene similarity threshold ...   10 Elapsed time is 0.056574 seconds.
Compute gene-gene similarity ... Elapsed time is 0.002308 seconds.
Create gene-gene graph for clustering genes ...
Writing graph into file ... 100%Elapsed time is 0.004938 seconds.
Running ModularityOptimizer for clustering ...Elapsed time is 0.225046 seconds.
Gene-gene graph contains 4 pathways, 144 genes in total
Elapsed time is 0.235087 seconds.
Create cell-cell graph for clustering cells ...
Writing graph into file ... 100%Elapsed time is 0.034570 seconds.
Running ModularityOptimizer for clustering ...Elapsed time is 0.326775 seconds.
Cell-cell graph contains 7 cell types by community detection
Elapsed time is 0.337673 seconds.
Cell-cell graph contains 6 cell types after merging tiny cell clusters
creating a total of 5 edges ...     5
Cell-cell graph contains 1 cell types after merging

Remaining clusters to partition 11
Processing cluster 45 now ...
Processing data subset with 18134 genes and 172 cells:
Remove genes detected in <100 cells. Remaining 0 genes.  Elapsed time is 0.008336 seconds.

Remaining clusters to partition 10
Processing cluster 46 now ...
Processing data subset with 18134 genes and 9887 cells:
Remove genes detected in <100 cells. Remaining 2789 genes.  Elapsed time is 0.588517 seconds.
Iterate 10 random permutations for gene-gene similarity threshold ...   10 Elapsed time is 33.991074 seconds.
Compute gene-gene similarity ... Elapsed time is 1.446977 seconds.
Create gene-gene graph for clustering genes ...
Writing graph into file ... 100%Elapsed time is 0.558543 seconds.
Running ModularityOptimizer for clustering ...Elapsed time is 1.882922 seconds.
Gene-gene graph contains 5 pathways, 2422 genes in total
Elapsed time is 1.960136 seconds.
Create cell-cell graph for clustering cells ...
Writing graph into file ... 100%Elapsed time is 1.120876 seconds.
Running ModularityOptimizer for clustering ...Elapsed time is 7.567667 seconds.
Cell-cell graph contains 16 cell types by community detection
Elapsed time is 7.770966 seconds.
Cell-cell graph contains 14 cell types after merging tiny cell clusters
creating a total of 13 edges ...     13
Cell-cell graph contains 2 cell types after merging
Number of useful pathways is 1
```

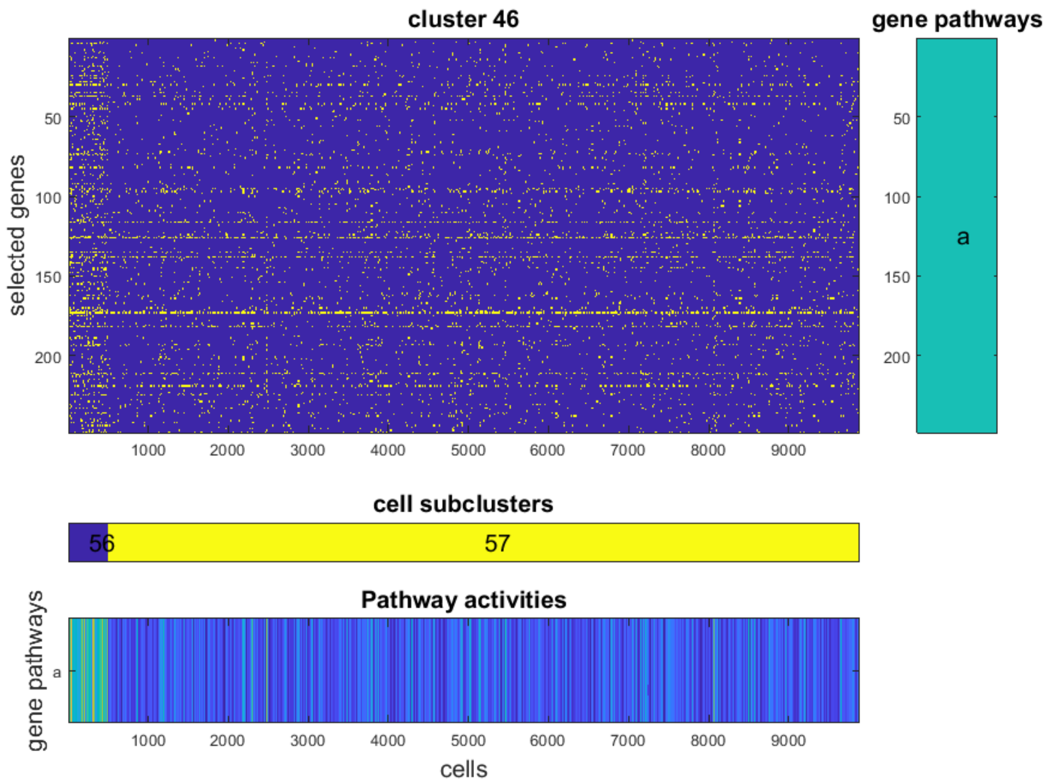


```
Remaining clusters to partition 11
Processing cluster 47 now ...
Processing data subset with 18134 genes and 58 cells:
Remove genes detected in <100 cells. Remaining 0 genes.  Elapsed time is 0.003638 seconds.

Remaining clusters to partition 10
Processing cluster 48 now ...
Processing data subset with 18134 genes and 255 cells:
Remove genes detected in <100 cells. Remaining 1013 genes.  Elapsed time is 0.014572 seconds.
Iterate 10 random permutations for gene-gene similarity threshold ...   10 Elapsed time is 0.790485 seconds.
Compute gene-gene similarity ... Elapsed time is 0.056169 seconds.
Create gene-gene graph for clustering genes ...
Writing graph into file ... 100%Elapsed time is 0.010678 seconds.
Running ModularityOptimizer for clustering ...Elapsed time is 0.331687 seconds.
Gene-gene graph contains 4 pathways, 155 genes in total
Elapsed time is 0.359629 seconds.
Create cell-cell graph for clustering cells ...
Writing graph into file ... 100%Elapsed time is 0.028803 seconds.
Running ModularityOptimizer for clustering ...Elapsed time is 0.310847 seconds.
Cell-cell graph contains 6 cell types by community detection
Elapsed time is 0.320964 seconds.
Cell-cell graph contains 5 cell types after merging tiny cell clusters
creating a total of 4 edges ...      4
Cell-cell graph contains 2 cell types after merging
Number of useful pathways is 1
```

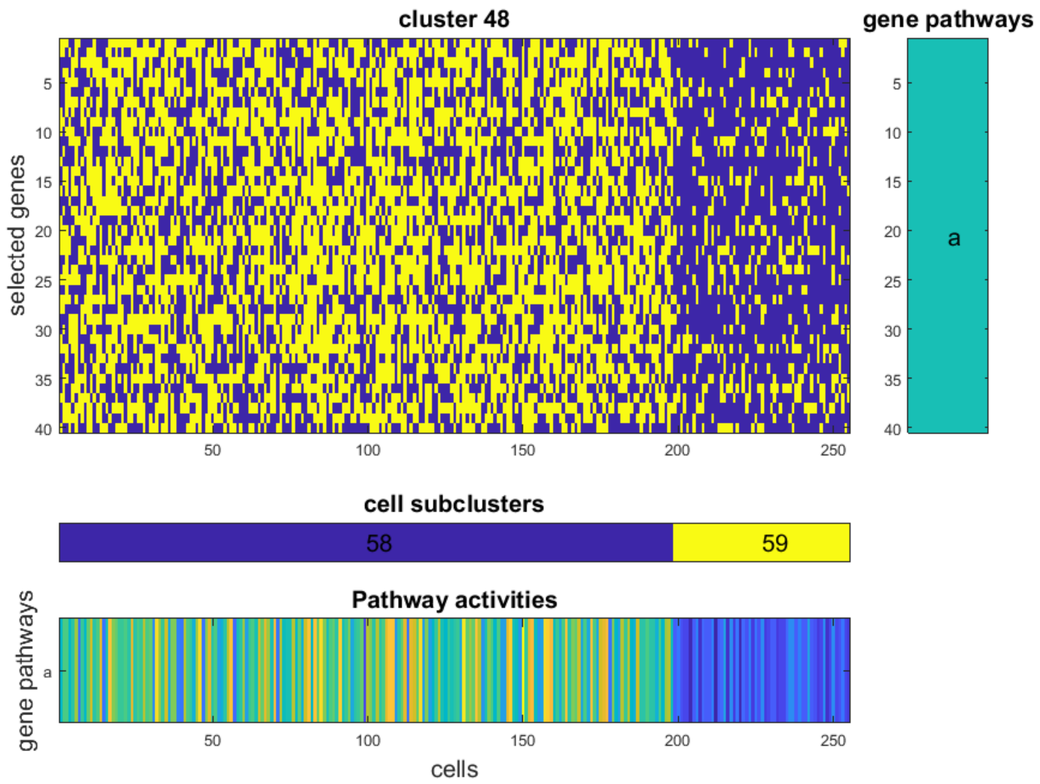


```
Remaining clusters to partition 11
Processing cluster 49 now ...
Processing data subset with 18134 genes and 59 cells:
Remove genes detected in <100 cells. Remaining 0 genes.  Elapsed time is 0.003003 seconds.

Remaining clusters to partition 10
Processing cluster 50 now ...
Processing data subset with 18134 genes and 280 cells:
Remove genes detected in <100 cells. Remaining 1151 genes.  Elapsed time is 0.017112 seconds.
Iterate 10 random permutations for gene-gene similarity threshold ...   10 Elapsed time is 1.124372 seconds.
Compute gene-gene similarity ... Elapsed time is 0.084499 seconds.
Create gene-gene graph for clustering genes ...
Writing graph into file ... 101%Elapsed time is 0.011061 seconds.
Running ModularityOptimizer for clustering ...Elapsed time is 0.291058 seconds.
Gene-gene graph contains 0 pathways, 0 genes in total
Elapsed time is 0.325736 seconds.

Remaining clusters to partition 9
Processing cluster 51 now ...
Processing data subset with 18134 genes and 4087 cells:
Remove genes detected in <100 cells. Remaining 9808 genes.  Elapsed time is 0.361681 seconds.
Iterate 10 random permutations for gene-gene similarity threshold ...   10 Elapsed time is 132.937528 seconds.
Compute gene-gene similarity ... Elapsed time is 9.272352 seconds.
Create gene-gene graph for clustering genes ...
Writing graph into file ... 100%Elapsed time is 6.328236 seconds.
Running ModularityOptimizer for clustering ...Elapsed time is 17.966634 seconds.
Gene-gene graph contains 4 pathways, 7913 genes in total
Elapsed time is 18.344820 seconds.
Create cell-cell graph for clustering cells ...
Writing graph into file ... 100%Elapsed time is 0.451890 seconds.
Running ModularityOptimizer for clustering ...Elapsed time is 2.102223 seconds.
Cell-cell graph contains 15 cell types by community detection
Elapsed time is 2.193156 seconds.
Cell-cell graph contains 11 cell types after merging tiny cell clusters
creating a total of 10 edges ...     10
Cell-cell graph contains 1 cell types after merging

Remaining clusters to partition 8
Processing cluster 52 now ...
Processing data subset with 18134 genes and 92 cells:
Remove genes detected in <100 cells. Remaining 0 genes.  Elapsed time is 0.005448 seconds.

Remaining clusters to partition 7
Processing cluster 53 now ...
Processing data subset with 18134 genes and 3175 cells:
Remove genes detected in <100 cells. Remaining 10363 genes.  Elapsed time is 0.280272 seconds.
Iterate 10 random permutations for gene-gene similarity threshold ...   10 Elapsed time is 128.795680 seconds.
Compute gene-gene similarity ... Elapsed time is 9.375802 seconds.
Create gene-gene graph for clustering genes ...
Writing graph into file ... 100%Elapsed time is 4.176550 seconds.
Running ModularityOptimizer for clustering ...Elapsed time is 11.617290 seconds.
Gene-gene graph contains 5 pathways, 7102 genes in total
Elapsed time is 11.999402 seconds.
Create cell-cell graph for clustering cells ...
Writing graph into file ... 100%Elapsed time is 0.346751 seconds.
Running ModularityOptimizer for clustering ...Elapsed time is 1.557762 seconds.
```

```
Cell-cell graph contains 14 cell types by community detection
Elapsed time is 1.629167 seconds.
Cell-cell graph contains 10 cell types after merging tiny cell clusters
creating a total of 9 edges ...      9
Cell-cell graph contains 1 cell types after merging

Remaining clusters to partition 6
Processing cluster 54 now ...
Processing data subset with 18134 genes and 39643 cells:
Remove genes detected in <100 cells. Remaining 14634 genes.  Elapsed time is 3.523141 seconds.
Iterate 10 random permutations for gene-gene similarity threshold ...   10 Elapsed time is 1953.066768 seconds.
Compute gene-gene similarity ... Elapsed time is 97.583571 seconds.
Create gene-gene graph for clustering genes ...
Writing graph into file ... 100%Elapsed time is 162.512050 seconds.
Running ModularityOptimizer for clustering ...Elapsed time is 492.256192 seconds.
Gene-gene graph contains 4 pathways, 14542 genes in total
Elapsed time is 492.966575 seconds.
Create cell-cell graph for clustering cells ...
Writing graph into file ... 100%Elapsed time is 4.374372 seconds.
Running ModularityOptimizer for clustering ...Elapsed time is 40.165683 seconds.
Cell-cell graph contains 27 cell types by community detection
Elapsed time is 40.944324 seconds.
Cell-cell graph contains 15 cell types after merging tiny cell clusters
creating a total of 14 edges ...     14
Cell-cell graph contains 2 cell types after merging
Number of useful pathways is 1
```

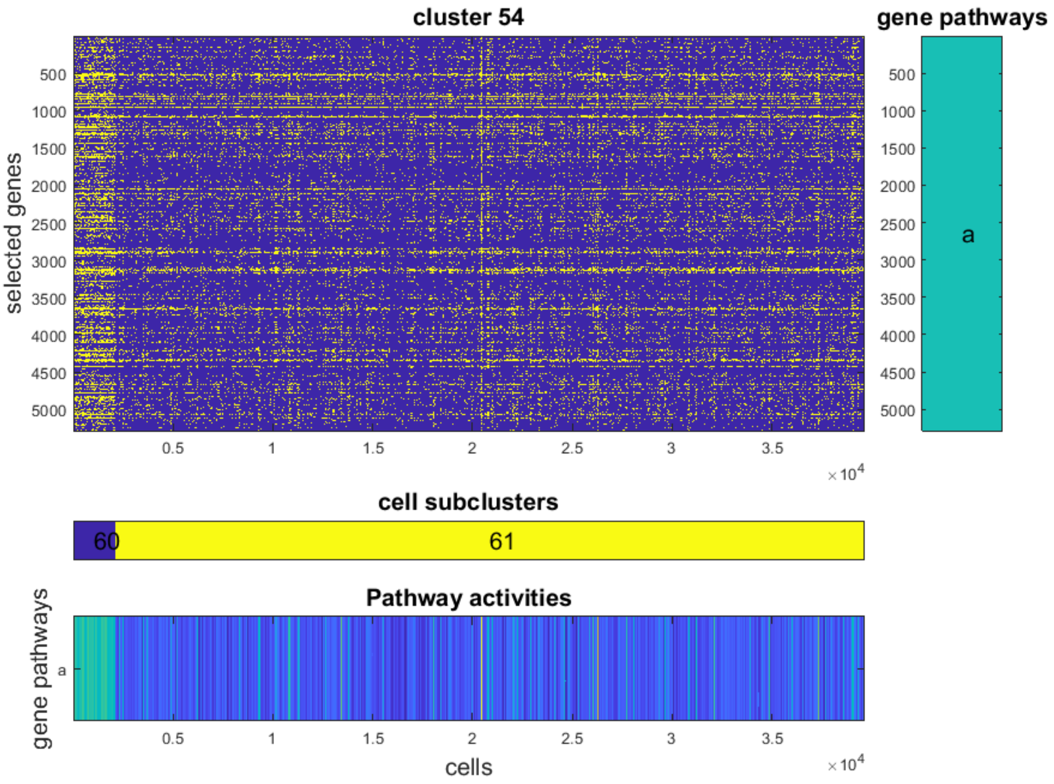


```
Remaining clusters to partition 7
Processing cluster 55 now ...
Processing data subset with 18134 genes and 1625 cells:
Remove genes detected in <100 cells. Remaining 4204 genes.  Elapsed time is 0.110586 seconds.
Iterate 10 random permutations for gene-gene similarity threshold ...   10 Elapsed time is 18.894974 seconds.
Compute gene-gene similarity ... Elapsed time is 1.189501 seconds.
Create gene-gene graph for clustering genes ...
Writing graph into file ... 100%Elapsed time is 4.037343 seconds.
Running ModularityOptimizer for clustering ...Elapsed time is 9.107775 seconds.
Gene-gene graph contains 4 pathways, 4141 genes in total
Elapsed time is 9.224970 seconds.
Create cell-cell graph for clustering cells ...
Writing graph into file ... 100%Elapsed time is 0.176368 seconds.
Running ModularityOptimizer for clustering ...Elapsed time is 0.761953 seconds.
Cell-cell graph contains 13 cell types by community detection
Elapsed time is 0.797704 seconds.
Cell-cell graph contains 9 cell types after merging tiny cell clusters
creating a total of 8 edges ...      8
Cell-cell graph contains 2 cell types after merging
Number of useful pathways is 1
```

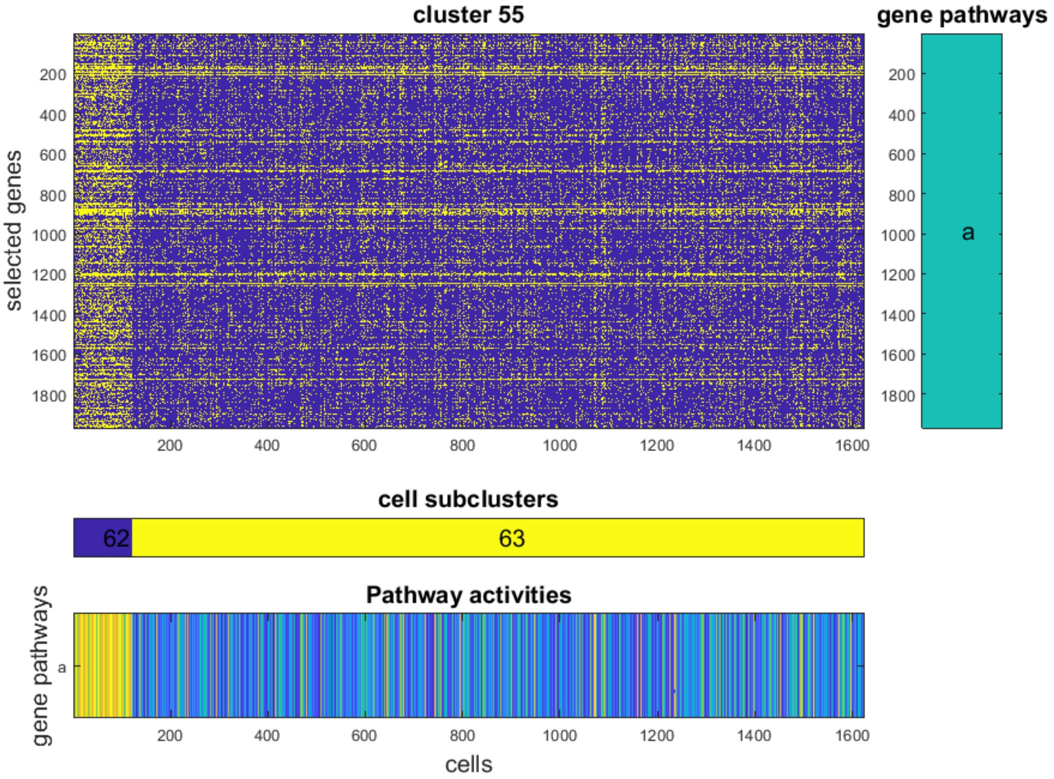


```
Remaining clusters to partition 8
Processing cluster 56 now ...
Processing data subset with 18134 genes and 496 cells:
Remove genes detected in <100 cells. Remaining 248 genes.  Elapsed time is 0.024166 seconds.
Iterate 10 random permutations for gene-gene similarity threshold ...   10 Elapsed time is 0.080587 seconds.
Compute gene-gene similarity ... Elapsed time is 0.003127 seconds.
Create gene-gene graph for clustering genes ...
Writing graph into file ... 100%Elapsed time is 0.004894 seconds.
Running ModularityOptimizer for clustering ...Elapsed time is 0.217763 seconds.
Gene-gene graph contains 3 pathways, 109 genes in total
Elapsed time is 0.226398 seconds.
Create cell-cell graph for clustering cells ...
Writing graph into file ... 100%Elapsed time is 0.063972 seconds.
Running ModularityOptimizer for clustering ...Elapsed time is 0.412261 seconds.
Cell-cell graph contains 8 cell types by community detection
Elapsed time is 0.424677 seconds.
Cell-cell graph contains 8 cell types after merging tiny cell clusters
Cell-cell graph contains 2 cell types after merging
Number of useful pathways is 1
```

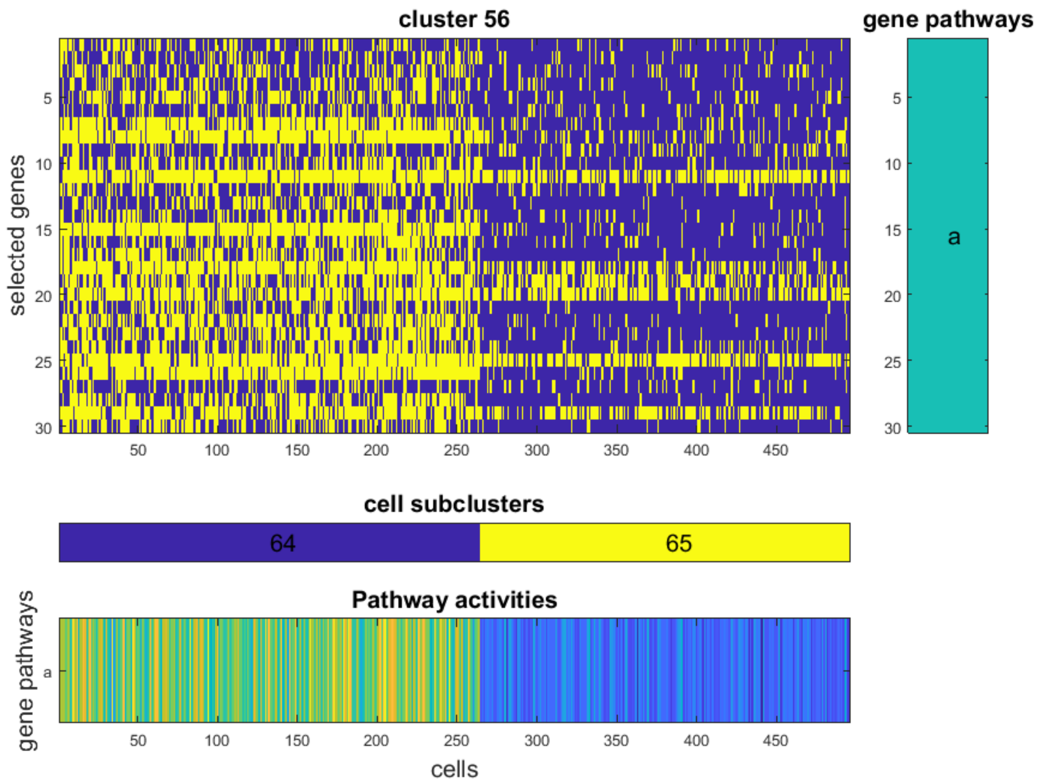


```
Remaining clusters to partition 9
Processing cluster 57 now ...
Processing data subset with 18134 genes and 9391 cells:
Remove genes detected in <100 cells. Remaining 2568 genes.  Elapsed time is 0.620743 seconds.
Iterate 10 random permutations for gene-gene similarity threshold ...   10 Elapsed time is 29.112353 seconds.
Compute gene-gene similarity ... Elapsed time is 1.178174 seconds.
Create gene-gene graph for clustering genes ...
Writing graph into file ... 100%Elapsed time is 0.524092 seconds.
Running ModularityOptimizer for clustering ...Elapsed time is 1.672927 seconds.
Gene-gene graph contains 5 pathways, 2277 genes in total
Elapsed time is 1.739609 seconds.
Create cell-cell graph for clustering cells ...
Writing graph into file ... 100%Elapsed time is 1.065876 seconds.
Running ModularityOptimizer for clustering ...Elapsed time is 6.701706 seconds.
Cell-cell graph contains 16 cell types by community detection
Elapsed time is 6.891683 seconds.
Cell-cell graph contains 15 cell types after merging tiny cell clusters
creating a total of 14 edges ...     14
Cell-cell graph contains 2 cell types after merging
Number of useful pathways is 1
```

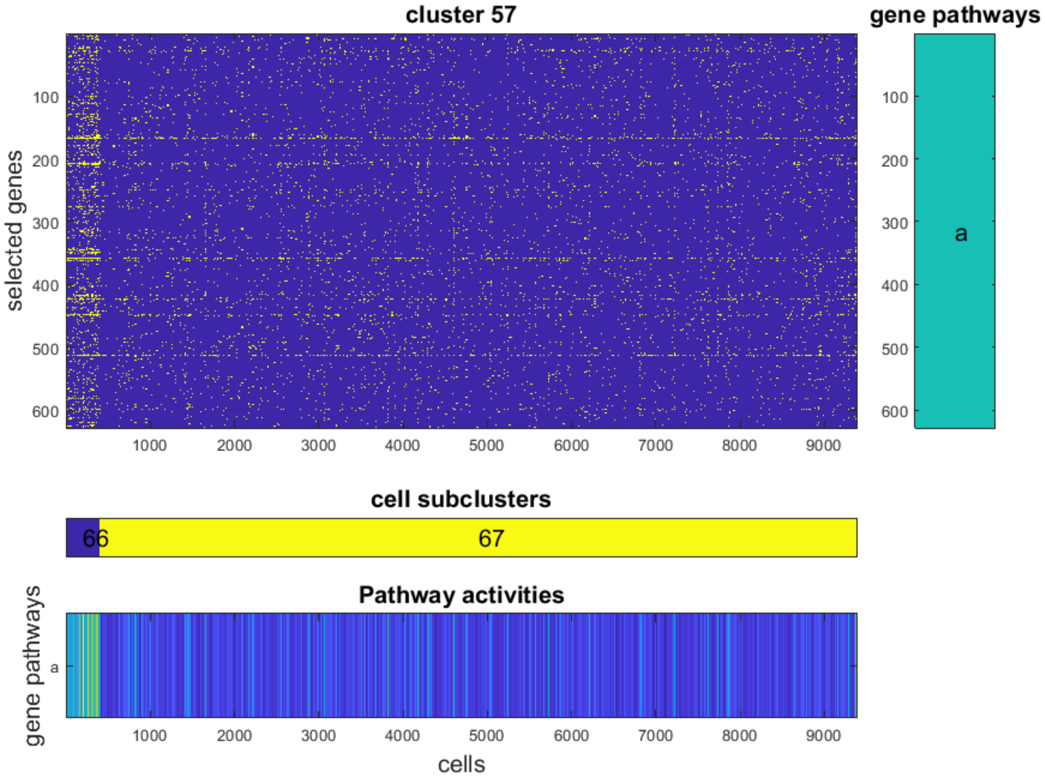


```
Remaining clusters to partition 10
Processing cluster 58 now ...
Processing data subset with 18134 genes and 198 cells:
Remove genes detected in <100 cells. Remaining 0 genes.  Elapsed time is 0.012335 seconds.

Remaining clusters to partition 9
Processing cluster 59 now ...
Processing data subset with 18134 genes and 57 cells:
Remove genes detected in <100 cells. Remaining 0 genes.  Elapsed time is 0.003065 seconds.

Remaining clusters to partition 8
Processing cluster 60 now ...
Processing data subset with 18134 genes and 2094 cells:
Remove genes detected in <100 cells. Remaining 8278 genes.  Elapsed time is 0.170348 seconds.
Iterate 10 random permutations for gene-gene similarity threshold ...   10 Elapsed time is 72.012214 seconds.
Compute gene-gene similarity ... Elapsed time is 5.189914 seconds.
Create gene-gene graph for clustering genes ...
Writing graph into file ... 100%Elapsed time is 0.606670 seconds.
Running ModularityOptimizer for clustering ...Elapsed time is 1.123849 seconds.
Gene-gene graph contains 6 pathways, 2644 genes in total
Elapsed time is 1.407794 seconds.
Create cell-cell graph for clustering cells ...
Writing graph into file ... 100%Elapsed time is 0.241965 seconds.
Running ModularityOptimizer for clustering ...Elapsed time is 1.442766 seconds.
Cell-cell graph contains 11 cell types by community detection
Elapsed time is 1.489268 seconds.
Cell-cell graph contains 11 cell types after merging tiny cell clusters
creating a total of 10 edges ...     10
Cell-cell graph contains 2 cell types after merging
Number of useful pathways is 1
```

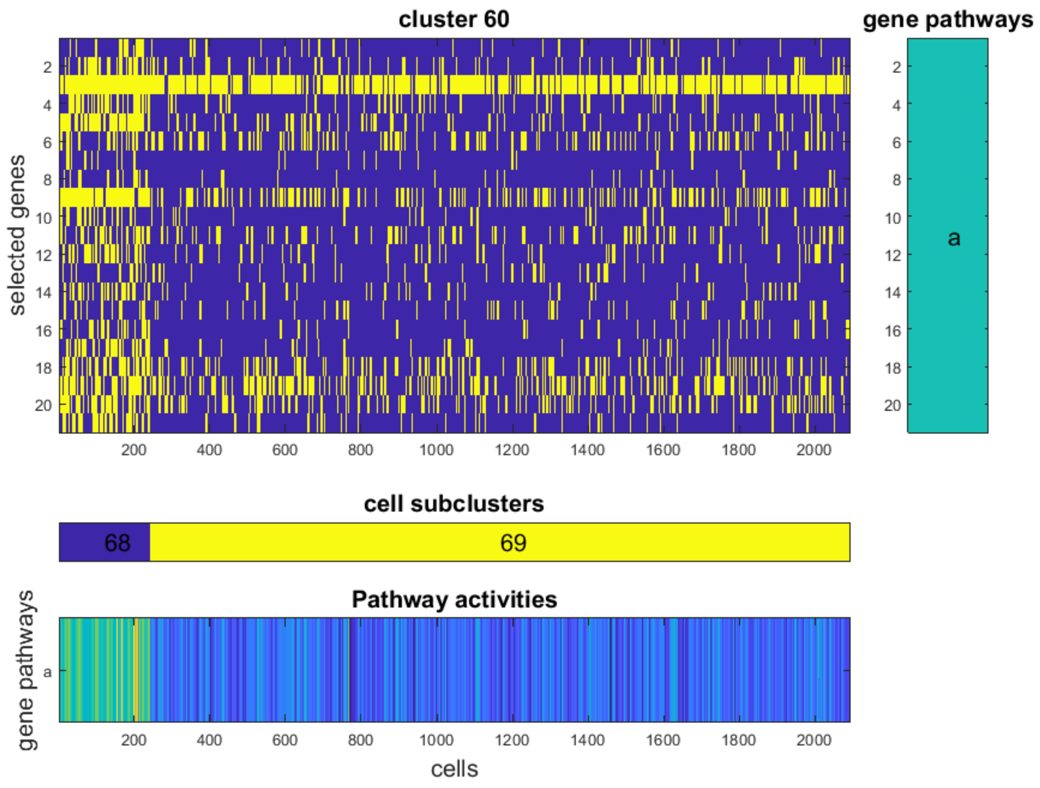


```
Remaining clusters to partition 9
Processing cluster 61 now ...
Processing data subset with 18134 genes and 37549 cells:
Remove genes detected in <100 cells. Remaining 14511 genes.  Elapsed time is 3.544331 seconds.
Iterate 10 random permutations for gene-gene similarity threshold ...   10 Elapsed time is 1773.832889 seconds.
Compute gene-gene similarity ... Elapsed time is 92.392481 seconds.
Create gene-gene graph for clustering genes ...
Writing graph into file ... 100%Elapsed time is 150.355566 seconds.
Running ModularityOptimizer for clustering ...Elapsed time is 494.741203 seconds.
Gene-gene graph contains 5 pathways, 14422 genes in total
Elapsed time is 495.415288 seconds.
Create cell-cell graph for clustering cells ...
Writing graph into file ... 100%Elapsed time is 4.276043 seconds.
Running ModularityOptimizer for clustering ...Elapsed time is 53.725266 seconds.
Cell-cell graph contains 26 cell types by community detection
Elapsed time is 54.510763 seconds.
Cell-cell graph contains 15 cell types after merging tiny cell clusters
creating a total of 14 edges ...     14
Cell-cell graph contains 2 cell types after merging
Number of useful pathways is 1
```

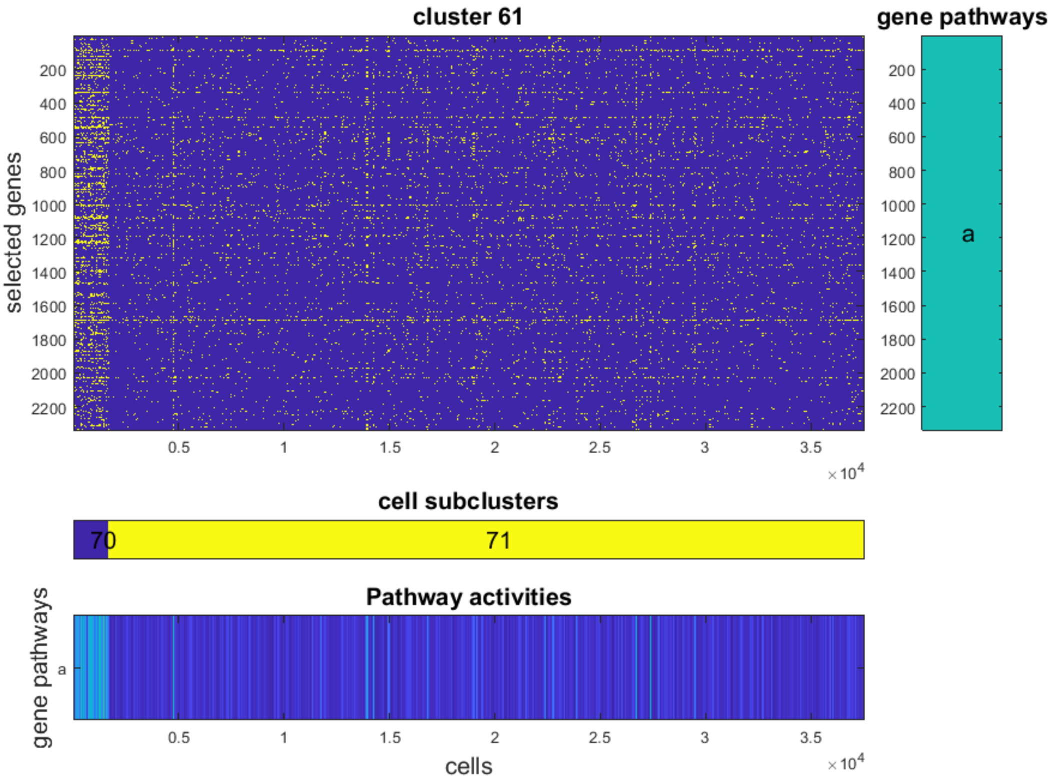


```
Remaining clusters to partition 10
Processing cluster 62 now ...
Processing data subset with 18134 genes and 121 cells:
Remove genes detected in <100 cells. Remaining 0 genes.  Elapsed time is 0.006858 seconds.

Remaining clusters to partition 9
Processing cluster 63 now ...
Processing data subset with 18134 genes and 1504 cells:
Remove genes detected in <100 cells. Remaining 3677 genes.  Elapsed time is 0.098222 seconds.
Iterate 10 random permutations for gene-gene similarity threshold ...   10 Elapsed time is 14.532965 seconds.
Compute gene-gene similarity ... Elapsed time is 0.997479 seconds.
Create gene-gene graph for clustering genes ...
Writing graph into file ... 100%Elapsed time is 2.648314 seconds.
Running ModularityOptimizer for clustering ...Elapsed time is 6.266544 seconds.
Gene-gene graph contains 4 pathways, 3625 genes in total
Elapsed time is 6.374525 seconds.
Create cell-cell graph for clustering cells ...
Writing graph into file ... 100%Elapsed time is 0.165824 seconds.
Running ModularityOptimizer for clustering ...Elapsed time is 0.824832 seconds.
Cell-cell graph contains 14 cell types by community detection
Elapsed time is 0.861105 seconds.
Cell-cell graph contains 13 cell types after merging tiny cell clusters
creating a total of 12 edges ...     12
Cell-cell graph contains 2 cell types after merging
Number of useful pathways is 1
```

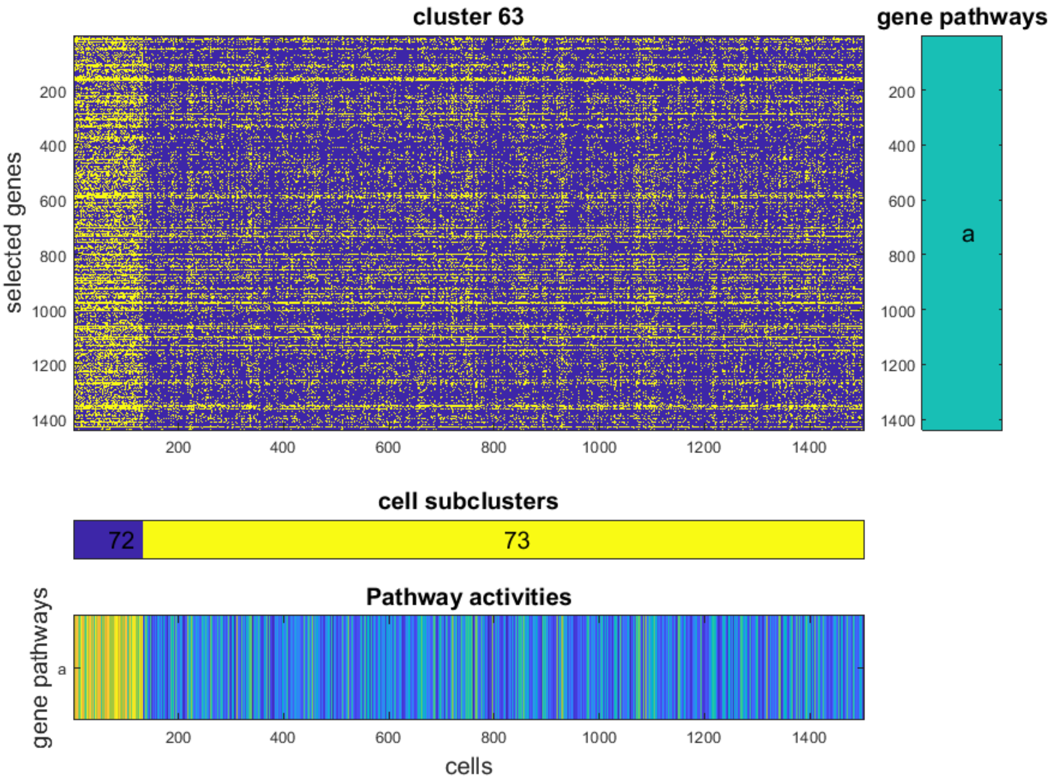


```
Remaining clusters to partition 10
Processing cluster 64 now ...
Processing data subset with 18134 genes and 264 cells:
Remove genes detected in <100 cells. Remaining 59 genes.  Elapsed time is 0.013613 seconds.
Iterate 10 random permutations for gene-gene similarity threshold ...   10 Elapsed time is 0.017864 seconds.
Compute gene-gene similarity ... Elapsed time is 0.000616 seconds.
Create gene-gene graph for clustering genes ...
Writing graph into file ... 103%Elapsed time is 0.001506 seconds.
Running ModularityOptimizer for clustering ...Elapsed time is 0.177338 seconds.
Gene-gene graph contains 0 pathways, 0 genes in total
Elapsed time is 0.182267 seconds.

Remaining clusters to partition 9
Processing cluster 65 now ...
Processing data subset with 18134 genes and 232 cells:
Remove genes detected in <100 cells. Remaining 43 genes.  Elapsed time is 0.012447 seconds.
Iterate 10 random permutations for gene-gene similarity threshold ...   10 Elapsed time is 0.014059 seconds.
Compute gene-gene similarity ... Elapsed time is 0.000540 seconds.
Create gene-gene graph for clustering genes ...
Writing graph into file ... 120%Elapsed time is 0.002647 seconds.
Running ModularityOptimizer for clustering ...Elapsed time is 0.176062 seconds.
Gene-gene graph contains 0 pathways, 0 genes in total
Elapsed time is 0.179919 seconds.

Remaining clusters to partition 8
Processing cluster 66 now ...
Processing data subset with 18134 genes and 398 cells:
Remove genes detected in <100 cells. Remaining 204 genes.  Elapsed time is 0.021603 seconds.
Iterate 10 random permutations for gene-gene similarity threshold ...   10 Elapsed time is 0.063339 seconds.
Compute gene-gene similarity ... Elapsed time is 0.002330 seconds.
Create gene-gene graph for clustering genes ...
Writing graph into file ... 100%Elapsed time is 0.004573 seconds.
Running ModularityOptimizer for clustering ...Elapsed time is 0.243901 seconds.
Gene-gene graph contains 3 pathways, 90 genes in total
Elapsed time is 0.252390 seconds.
Create cell-cell graph for clustering cells ...
Writing graph into file ... 100%Elapsed time is 0.046282 seconds.
Running ModularityOptimizer for clustering ...Elapsed time is 0.397872 seconds.
Cell-cell graph contains 7 cell types by community detection
Elapsed time is 0.408606 seconds.
Cell-cell graph contains 7 cell types after merging tiny cell clusters
creating a total of 6 edges ...      6
Cell-cell graph contains 1 cell types after merging

Remaining clusters to partition 7
Processing cluster 67 now ...
Processing data subset with 18134 genes and 8993 cells:
Remove genes detected in <100 cells. Remaining 2320 genes.  Elapsed time is 0.599781 seconds.
Iterate 10 random permutations for gene-gene similarity threshold ...   10 Elapsed time is 26.935142 seconds.
Compute gene-gene similarity ... Elapsed time is 0.953900 seconds.
Create gene-gene graph for clustering genes ...
Writing graph into file ... 100%Elapsed time is 0.347989 seconds.
Running ModularityOptimizer for clustering ...Elapsed time is 1.234301 seconds.
Gene-gene graph contains 6 pathways, 1847 genes in total
Elapsed time is 1.296095 seconds.
Create cell-cell graph for clustering cells ...
```

```
Writing graph into file ... 100%Elapsed time is 1.044793 seconds.
Running ModularityOptimizer for clustering ...Elapsed time is 7.683152 seconds.
Cell-cell graph contains 17 cell types by community detection
Elapsed time is 7.875871 seconds.
Cell-cell graph contains 15 cell types after merging tiny cell clusters
creating a total of 14 edges ...     14
Cell-cell graph contains 2 cell types after merging
Number of useful pathways is 2
```

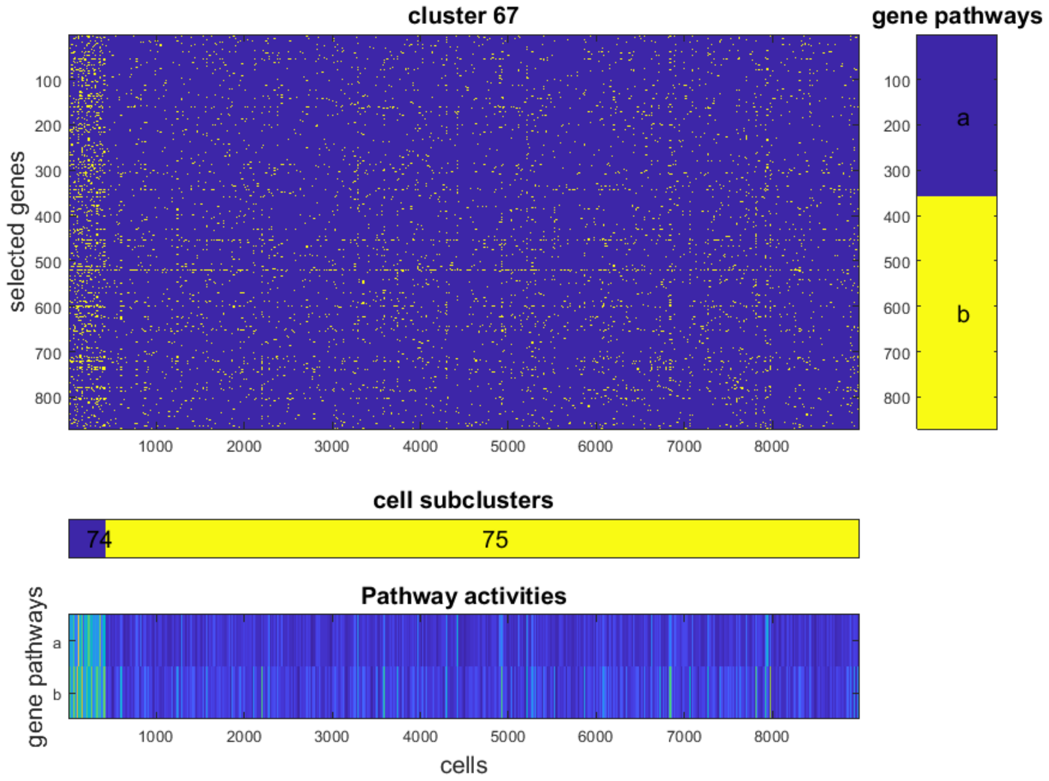


```
Remaining clusters to partition 8
Processing cluster 68 now ...
Processing data subset with 18134 genes and 240 cells:
Remove genes detected in <100 cells. Remaining 890 genes.  Elapsed time is 0.012480 seconds.
Iterate 10 random permutations for gene-gene similarity threshold ...   10 Elapsed time is 0.616841 seconds.
Compute gene-gene similarity ... Elapsed time is 0.045362 seconds.
Create gene-gene graph for clustering genes ...
Writing graph into file ... 101%Elapsed time is 0.007638 seconds.
Running ModularityOptimizer for clustering ...Elapsed time is 0.252878 seconds.
Gene-gene graph contains 1 pathways, 20 genes in total
Elapsed time is 0.356101 seconds.
Create cell-cell graph for clustering cells ...
Writing graph into file ... 100%Elapsed time is 0.027054 seconds.
Running ModularityOptimizer for clustering ...Elapsed time is 0.284304 seconds.
Cell-cell graph contains 7 cell types by community detection
Elapsed time is 0.291878 seconds.
Cell-cell graph contains 2 cell types after merging tiny cell clusters
creating a total of 1 edges ...      1
Cell-cell graph contains 1 cell types after merging

Remaining clusters to partition 7
Processing cluster 69 now ...
Processing data subset with 18134 genes and 1854 cells:
Remove genes detected in <100 cells. Remaining 7947 genes.  Elapsed time is 0.146061 seconds.
Iterate 10 random permutations for gene-gene similarity threshold ...   10 Elapsed time is 64.284224 seconds.
Compute gene-gene similarity ... Elapsed time is 4.648133 seconds.
Create gene-gene graph for clustering genes ...
Writing graph into file ... 100%Elapsed time is 0.541966 seconds.
Running ModularityOptimizer for clustering ...Elapsed time is 0.965377 seconds.
Gene-gene graph contains 5 pathways, 2383 genes in total
Elapsed time is 1.231865 seconds.
Create cell-cell graph for clustering cells ...
Writing graph into file ... 100%Elapsed time is 0.213524 seconds.
Running ModularityOptimizer for clustering ...Elapsed time is 1.128954 seconds.
Cell-cell graph contains 13 cell types by community detection
Elapsed time is 1.174348 seconds.
Cell-cell graph contains 13 cell types after merging tiny cell clusters
creating a total of 12 edges ...     12
Cell-cell graph contains 2 cell types after merging
Number of useful pathways is 1
```

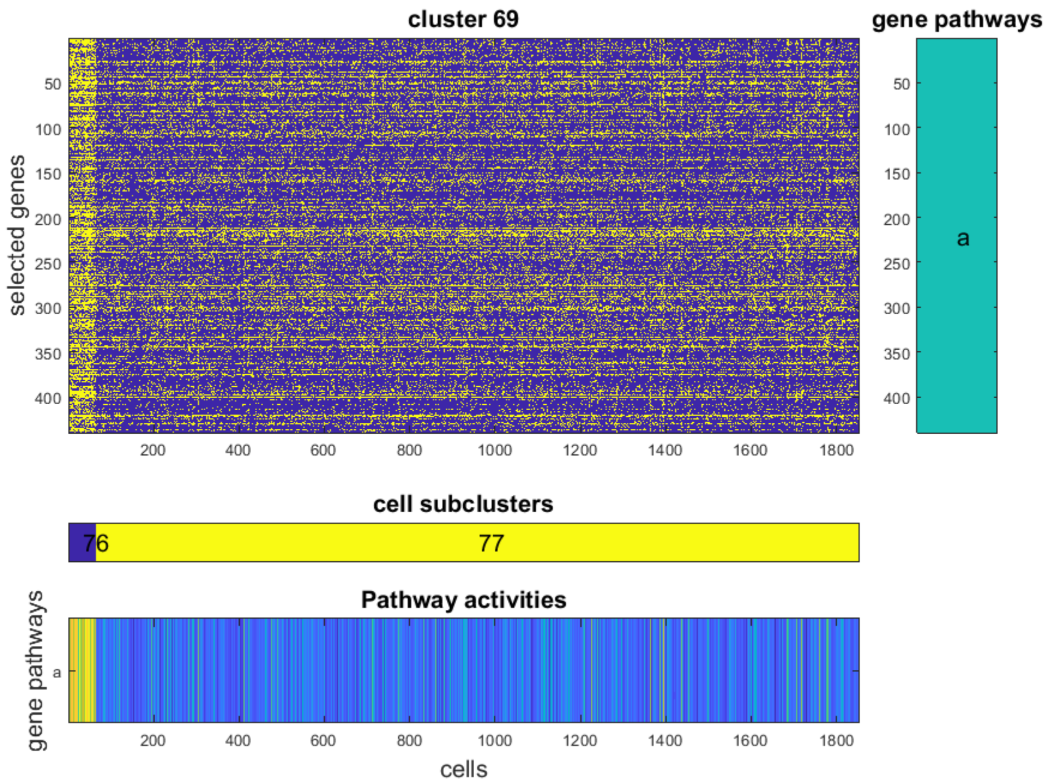


```
Remaining clusters to partition 8
Processing cluster 70 now ...
Processing data subset with 18134 genes and 1646 cells:
Remove genes detected in <100 cells. Remaining 7017 genes.  Elapsed time is 0.115664 seconds.
Iterate 10 random permutations for gene-gene similarity threshold ...   10 Elapsed time is 47.527128 seconds.
Compute gene-gene similarity ... Elapsed time is 3.368670 seconds.
Create gene-gene graph for clustering genes ...
Writing graph into file ... 100%Elapsed time is 3.278767 seconds.
Running ModularityOptimizer for clustering ...Elapsed time is 9.758954 seconds.
Gene-gene graph contains 3 pathways, 6076 genes in total
Elapsed time is 9.975464 seconds.
Create cell-cell graph for clustering cells ...
Writing graph into file ... 100%Elapsed time is 0.177744 seconds.
Running ModularityOptimizer for clustering ...Elapsed time is 0.783334 seconds.
Cell-cell graph contains 15 cell types by community detection
Elapsed time is 0.818278 seconds.
Cell-cell graph contains 15 cell types after merging tiny cell clusters
creating a total of 14 edges ...     14
Cell-cell graph contains 2 cell types after merging
Number of useful pathways is 1
```

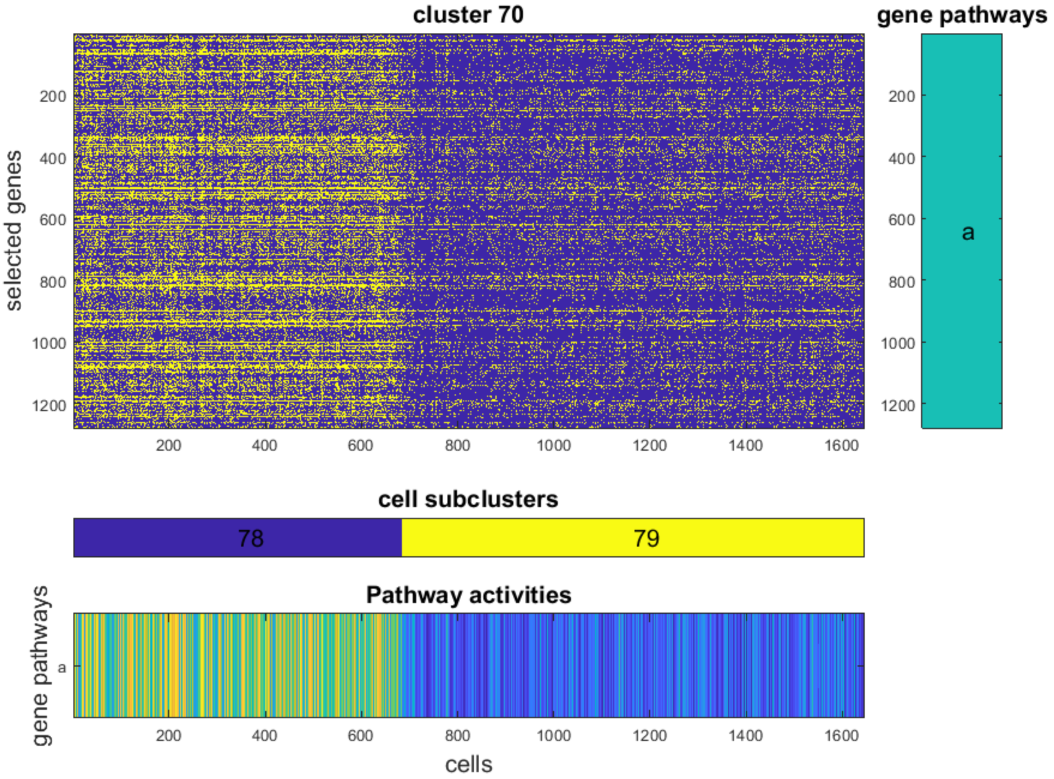


```
Remaining clusters to partition 9
Processing cluster 71 now ...
Processing data subset with 18134 genes and 35903 cells:
Remove genes detected in <100 cells. Remaining 14186 genes.  Elapsed time is 3.120725 seconds.
Iterate 10 random permutations for gene-gene similarity threshold ...   10 Elapsed time is 1536.011112 seconds.
Compute gene-gene similarity ... Elapsed time is 84.771704 seconds.
Create gene-gene graph for clustering genes ...
Writing graph into file ... 100%Elapsed time is 143.111801 seconds.
Running ModularityOptimizer for clustering ...Elapsed time is 369.377032 seconds.
Gene-gene graph contains 4 pathways, 14100 genes in total
Elapsed time is 370.009640 seconds.
Create cell-cell graph for clustering cells ...
Writing graph into file ... 100%Elapsed time is 3.957678 seconds.
Running ModularityOptimizer for clustering ...Elapsed time is 35.353796 seconds.
Cell-cell graph contains 20 cell types by community detection
Elapsed time is 36.061127 seconds.
Cell-cell graph contains 12 cell types after merging tiny cell clusters
creating a total of 11 edges ...     11
Cell-cell graph contains 2 cell types after merging
Number of useful pathways is 1
```

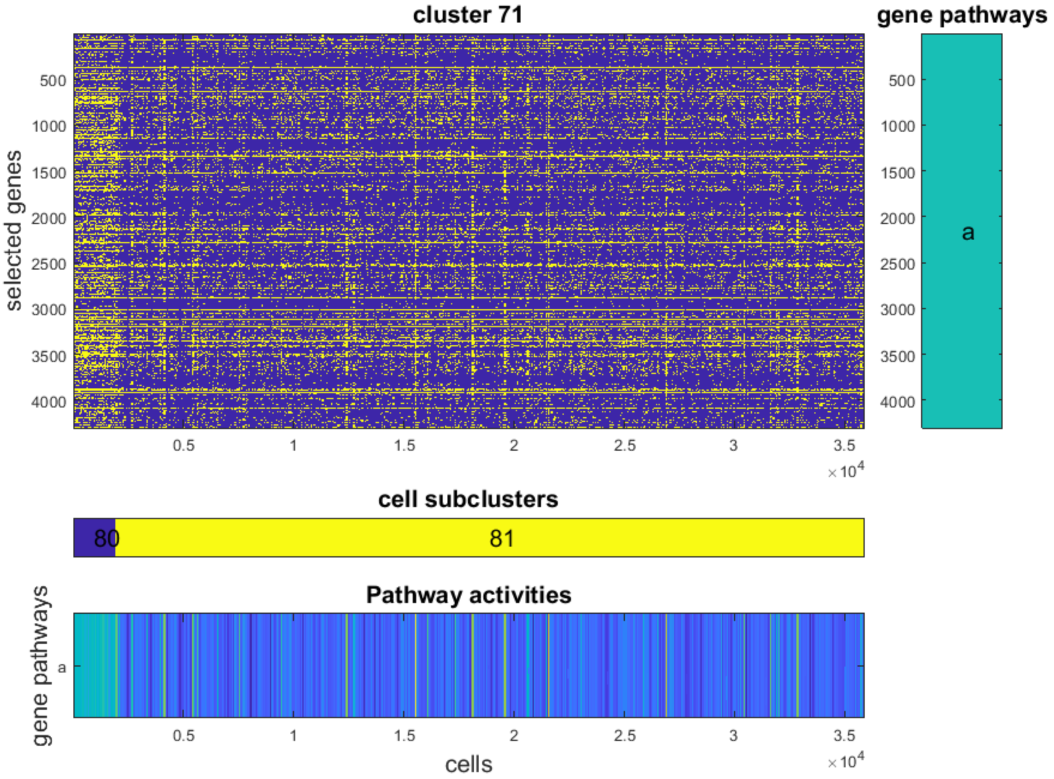


```
Remaining clusters to partition 10
Processing cluster 72 now ...
Processing data subset with 18134 genes and 133 cells:
Remove genes detected in <100 cells. Remaining 0 genes.  Elapsed time is 0.006536 seconds.

Remaining clusters to partition 9
Processing cluster 73 now ...
Processing data subset with 18134 genes and 1371 cells:
Remove genes detected in <100 cells. Remaining 3114 genes.  Elapsed time is 0.086283 seconds.
Iterate 10 random permutations for gene-gene similarity threshold ...   10 Elapsed time is 10.503336 seconds.
Compute gene-gene similarity ... Elapsed time is 0.645415 seconds.
Create gene-gene graph for clustering genes ...
Writing graph into file ... 100%Elapsed time is 1.595118 seconds.
Running ModularityOptimizer for clustering ...Elapsed time is 3.969225 seconds.
Gene-gene graph contains 4 pathways, 3049 genes in total
Elapsed time is 4.051001 seconds.
Create cell-cell graph for clustering cells ...
Writing graph into file ... 100%Elapsed time is 0.151565 seconds.
Running ModularityOptimizer for clustering ...Elapsed time is 0.777526 seconds.
Cell-cell graph contains 12 cell types by community detection
Elapsed time is 0.814302 seconds.
Cell-cell graph contains 10 cell types after merging tiny cell clusters
creating a total of 9 edges ...      9
Cell-cell graph contains 2 cell types after merging
Number of useful pathways is 3
```

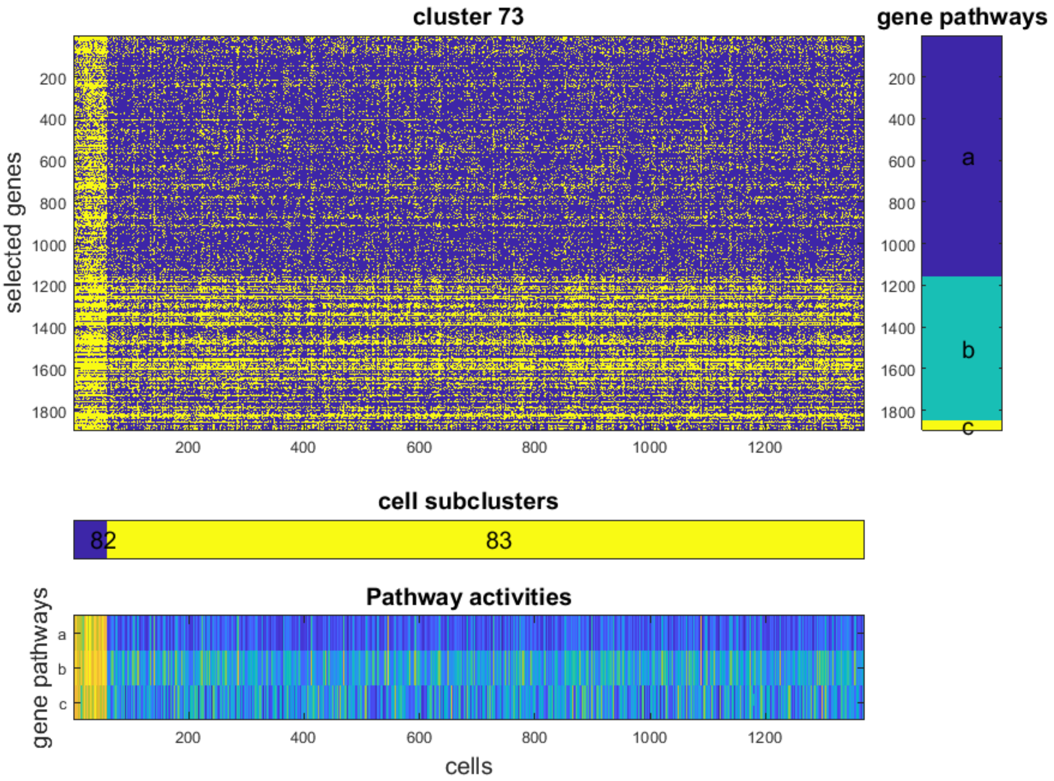


```
Remaining clusters to partition 10
Processing cluster 74 now ...
Processing data subset with 18134 genes and 416 cells:
Remove genes detected in <100 cells. Remaining 240 genes.  Elapsed time is 0.022024 seconds.
Iterate 10 random permutations for gene-gene similarity threshold ...   10 Elapsed time is 0.076172 seconds.
Compute gene-gene similarity ... Elapsed time is 0.002939 seconds.
Create gene-gene graph for clustering genes ...
Writing graph into file ... 100%Elapsed time is 0.003998 seconds.
Running ModularityOptimizer for clustering ...Elapsed time is 0.217035 seconds.
Gene-gene graph contains 4 pathways, 116 genes in total
Elapsed time is 0.226343 seconds.
Create cell-cell graph for clustering cells ...
Writing graph into file ... 100%Elapsed time is 0.046395 seconds.
Running ModularityOptimizer for clustering ...Elapsed time is 0.432631 seconds.
Cell-cell graph contains 8 cell types by community detection
Elapsed time is 0.443848 seconds.
Cell-cell graph contains 8 cell types after merging tiny cell clusters
creating a total of 7 edges ...      7
Cell-cell graph contains 1 cell types after merging

Remaining clusters to partition 9
Processing cluster 75 now ...
Processing data subset with 18134 genes and 8577 cells:
Remove genes detected in <100 cells. Remaining 1941 genes.  Elapsed time is 0.522428 seconds.
Iterate 10 random permutations for gene-gene similarity threshold ...   10 Elapsed time is 18.857246 seconds.
Compute gene-gene similarity ... Elapsed time is 0.636448 seconds.
Create gene-gene graph for clustering genes ...
Writing graph into file ... 100%Elapsed time is 0.177772 seconds.
Running ModularityOptimizer for clustering ...Elapsed time is 0.716480 seconds.
Gene-gene graph contains 5 pathways, 1326 genes in total
Elapsed time is 0.764885 seconds.
Create cell-cell graph for clustering cells ...
Writing graph into file ... 100%Elapsed time is 0.974994 seconds.
Running ModularityOptimizer for clustering ...Elapsed time is 6.372695 seconds.
Cell-cell graph contains 17 cell types by community detection
Elapsed time is 6.547887 seconds.
Cell-cell graph contains 16 cell types after merging tiny cell clusters
creating a total of 15 edges ...     15
Cell-cell graph contains 2 cell types after merging
Number of useful pathways is 1
```

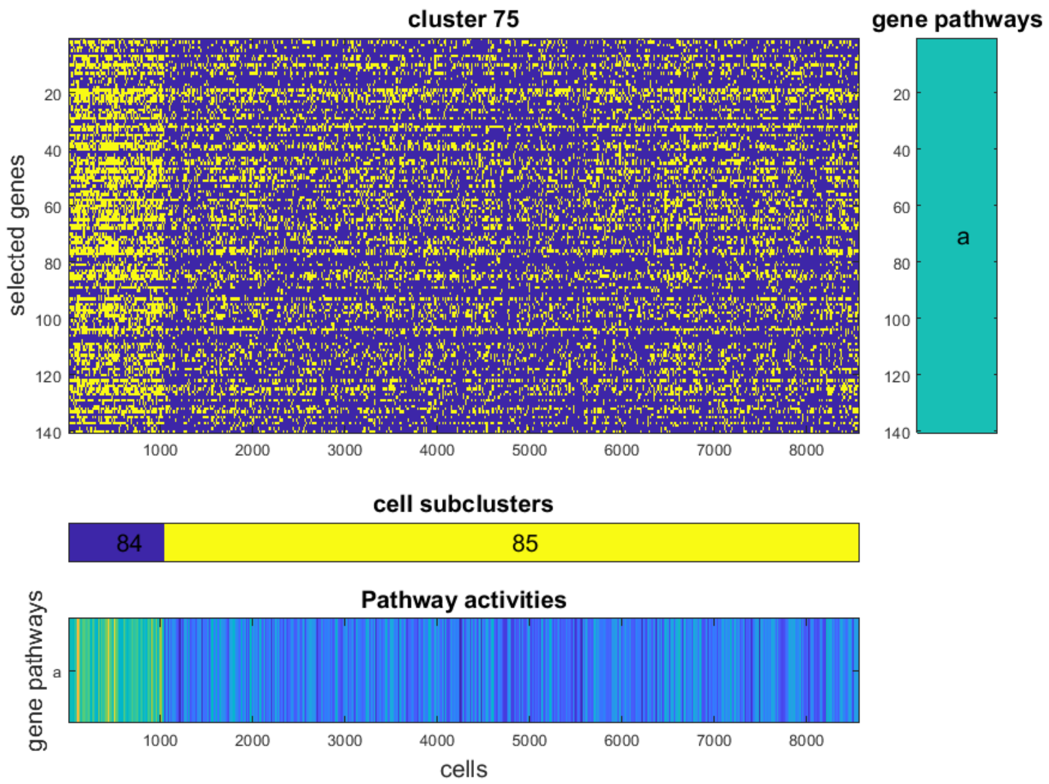


```
Remaining clusters to partition 10
Processing cluster 76 now ...
Processing data subset with 18134 genes and 65 cells:
Remove genes detected in <100 cells. Remaining 0 genes.  Elapsed time is 0.003605 seconds.

Remaining clusters to partition 9
Processing cluster 77 now ...
Processing data subset with 18134 genes and 1789 cells:
Remove genes detected in <100 cells. Remaining 7826 genes.  Elapsed time is 0.130658 seconds.
Iterate 10 random permutations for gene-gene similarity threshold ...   10 Elapsed time is 60.524515 seconds.
Compute gene-gene similarity ... Elapsed time is 4.438145 seconds.
Create gene-gene graph for clustering genes ...
Writing graph into file ... 100%Elapsed time is 0.498416 seconds.
Running ModularityOptimizer for clustering ...Elapsed time is 0.872067 seconds.
Gene-gene graph contains 8 pathways, 2151 genes in total
Elapsed time is 1.121342 seconds.
Create cell-cell graph for clustering cells ...
Writing graph into file ... 100%Elapsed time is 0.211981 seconds.
Running ModularityOptimizer for clustering ...Elapsed time is 1.230014 seconds.
Cell-cell graph contains 12 cell types by community detection
Elapsed time is 1.268824 seconds.
Cell-cell graph contains 11 cell types after merging tiny cell clusters
creating a total of 10 edges ...     10
Cell-cell graph contains 1 cell types after merging

Remaining clusters to partition 8
Processing cluster 78 now ...
Processing data subset with 18134 genes and 685 cells:
Remove genes detected in <100 cells. Remaining 3494 genes.  Elapsed time is 0.041291 seconds.
Iterate 10 random permutations for gene-gene similarity threshold ...   10 Elapsed time is 10.139603 seconds.
Compute gene-gene similarity ... Elapsed time is 0.716358 seconds.
Create gene-gene graph for clustering genes ...
Writing graph into file ... 100%Elapsed time is 0.145500 seconds.
Running ModularityOptimizer for clustering ...Elapsed time is 0.700154 seconds.
Gene-gene graph contains 5 pathways, 1824 genes in total
Elapsed time is 0.792372 seconds.
Create cell-cell graph for clustering cells ...
Writing graph into file ... 100%Elapsed time is 0.073005 seconds.
Running ModularityOptimizer for clustering ...Elapsed time is 0.480843 seconds.
Cell-cell graph contains 10 cell types by community detection
Elapsed time is 0.498225 seconds.
Cell-cell graph contains 10 cell types after merging tiny cell clusters
creating a total of 9 edges ...      9
Cell-cell graph contains 1 cell types after merging

Remaining clusters to partition 7
Processing cluster 79 now ...
Processing data subset with 18134 genes and 961 cells:
Remove genes detected in <100 cells. Remaining 4870 genes.  Elapsed time is 0.066634 seconds.
Iterate 10 random permutations for gene-gene similarity threshold ...   10 Elapsed time is 20.669387 seconds.
Compute gene-gene similarity ... Elapsed time is 1.435308 seconds.
Create gene-gene graph for clustering genes ...
Writing graph into file ... 100%Elapsed time is 1.564358 seconds.
Running ModularityOptimizer for clustering ...Elapsed time is 5.061562 seconds.
Gene-gene graph contains 5 pathways, 4472 genes in total
Elapsed time is 5.199389 seconds.
```

```
Create cell-cell graph for clustering cells ...
Writing graph into file ... 100%Elapsed time is 0.108055 seconds.
Running ModularityOptimizer for clustering ...Elapsed time is 0.654824 seconds.
Cell-cell graph contains 10 cell types by community detection
Elapsed time is 0.676390 seconds.
Cell-cell graph contains 9 cell types after merging tiny cell clusters
Cell-cell graph contains 2 cell types after merging
Number of useful pathways is 2
```

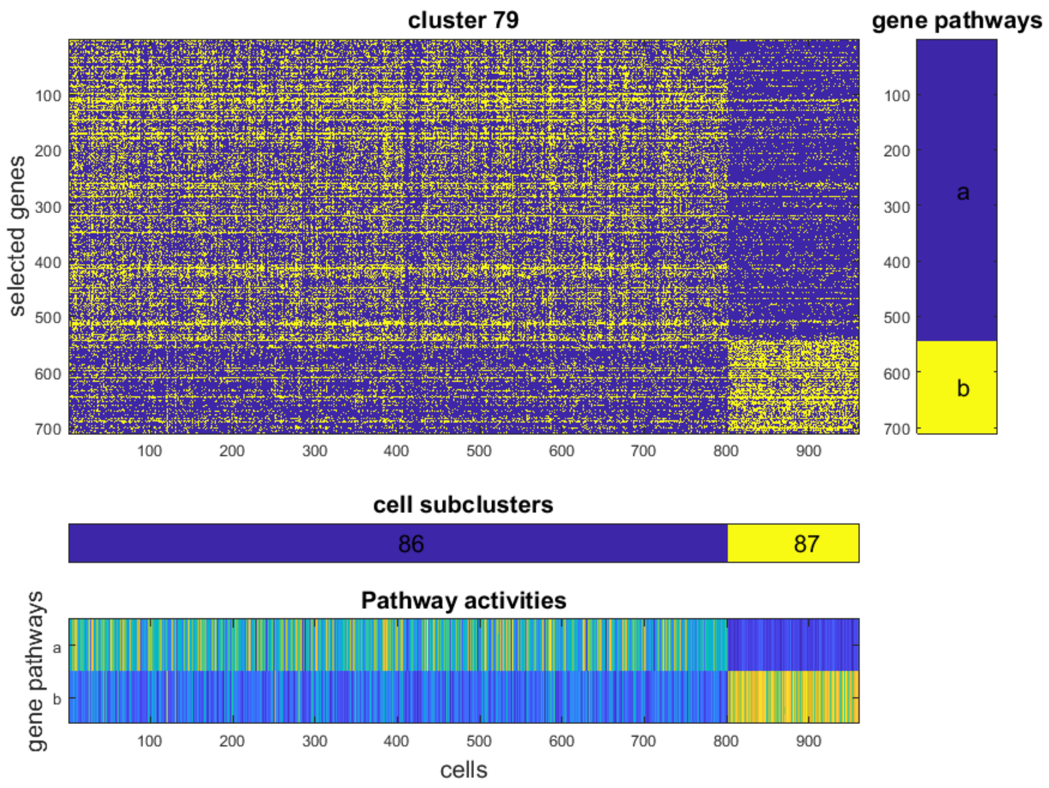


```
Remaining clusters to partition 8
Processing cluster 80 now ...
Processing data subset with 18134 genes and 1902 cells:
Remove genes detected in <100 cells. Remaining 7250 genes.  Elapsed time is 0.140355 seconds.
Iterate 10 random permutations for gene-gene similarity threshold ...   10 Elapsed time is 53.765691 seconds.
Compute gene-gene similarity ... Elapsed time is 4.015277 seconds.
Create gene-gene graph for clustering genes ...
Writing graph into file ... 100%Elapsed time is 0.473661 seconds.
Running ModularityOptimizer for clustering ...Elapsed time is 0.958236 seconds.
Gene-gene graph contains 6 pathways, 2219 genes in total
Elapsed time is 1.188495 seconds.
Create cell-cell graph for clustering cells ...
Writing graph into file ... 100%Elapsed time is 0.227794 seconds.
Running ModularityOptimizer for clustering ...Elapsed time is 1.338885 seconds.
Cell-cell graph contains 11 cell types by community detection
Elapsed time is 1.380472 seconds.
Cell-cell graph contains 11 cell types after merging tiny cell clusters
creating a total of 10 edges ...     10
Cell-cell graph contains 1 cell types after merging

Remaining clusters to partition 7
Processing cluster 81 now ...
Processing data subset with 18134 genes and 34001 cells:
Remove genes detected in <100 cells. Remaining 14011 genes.  Elapsed time is 2.945192 seconds.
Iterate 10 random permutations for gene-gene similarity threshold ...   10 Elapsed time is 1416.219406 seconds.
Compute gene-gene similarity ... Elapsed time is 78.784238 seconds.
Create gene-gene graph for clustering genes ...
Writing graph into file ... 100%Elapsed time is 143.004000 seconds.
Running ModularityOptimizer for clustering ...Elapsed time is 390.497650 seconds.
Gene-gene graph contains 6 pathways, 13951 genes in total
Elapsed time is 391.113064 seconds.
Create cell-cell graph for clustering cells ...
Writing graph into file ... 100%Elapsed time is 3.890311 seconds.
Running ModularityOptimizer for clustering ...Elapsed time is 36.655687 seconds.
Cell-cell graph contains 25 cell types by community detection
Elapsed time is 37.333246 seconds.
Cell-cell graph contains 11 cell types after merging tiny cell clusters
Cell-cell graph contains 2 cell types after merging
Number of useful pathways is 1
```

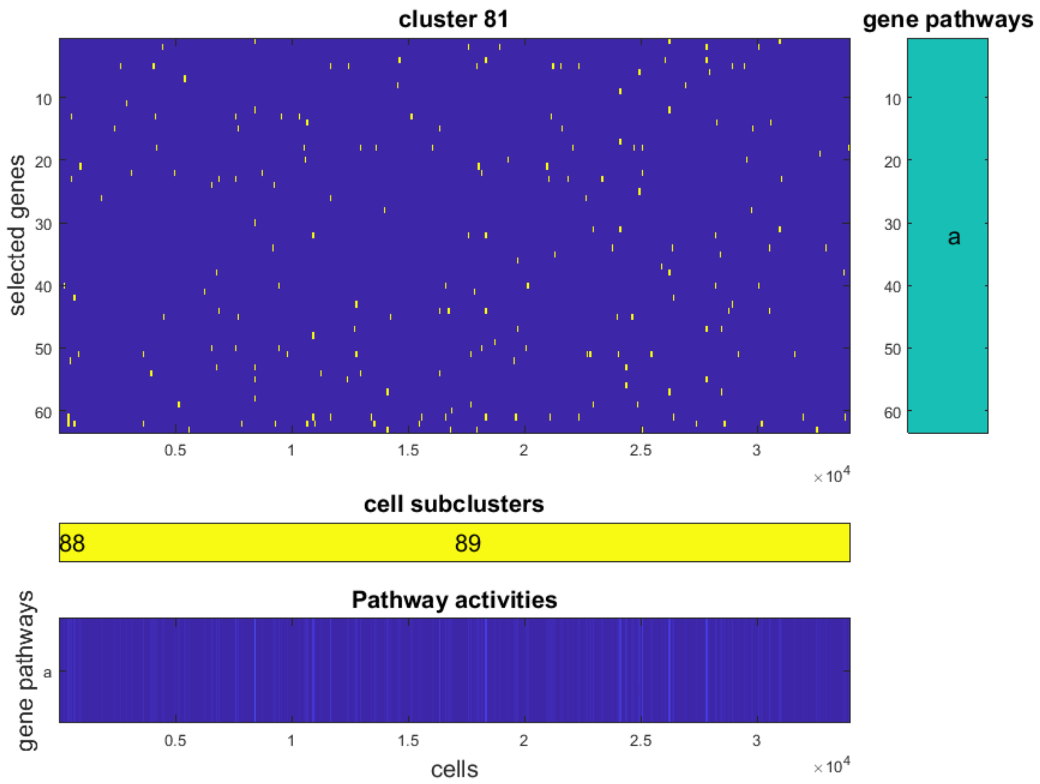


```
Remaining clusters to partition 8
Processing cluster 82 now ...
Processing data subset with 18134 genes and 59 cells:
Remove genes detected in <100 cells. Remaining 0 genes.  Elapsed time is 0.003454 seconds.

Remaining clusters to partition 7
Processing cluster 83 now ...
Processing data subset with 18134 genes and 1312 cells:
Remove genes detected in <100 cells. Remaining 2804 genes.  Elapsed time is 0.091450 seconds.
Iterate 10 random permutations for gene-gene similarity threshold ...   10 Elapsed time is 8.550889 seconds.
Compute gene-gene similarity ... Elapsed time is 0.530921 seconds.
Create gene-gene graph for clustering genes ...
Writing graph into file ... 100%Elapsed time is 0.481047 seconds.
Running ModularityOptimizer for clustering ...Elapsed time is 1.667814 seconds.
Gene-gene graph contains 4 pathways, 2475 genes in total
Elapsed time is 1.740980 seconds.
Create cell-cell graph for clustering cells ...
Writing graph into file ... 100%Elapsed time is 0.143386 seconds.
Running ModularityOptimizer for clustering ...Elapsed time is 0.726320 seconds.
Cell-cell graph contains 12 cell types by community detection
Elapsed time is 0.755129 seconds.
Cell-cell graph contains 12 cell types after merging tiny cell clusters
creating a total of 11 edges ...     11
Cell-cell graph contains 2 cell types after merging
Number of useful pathways is 1
```

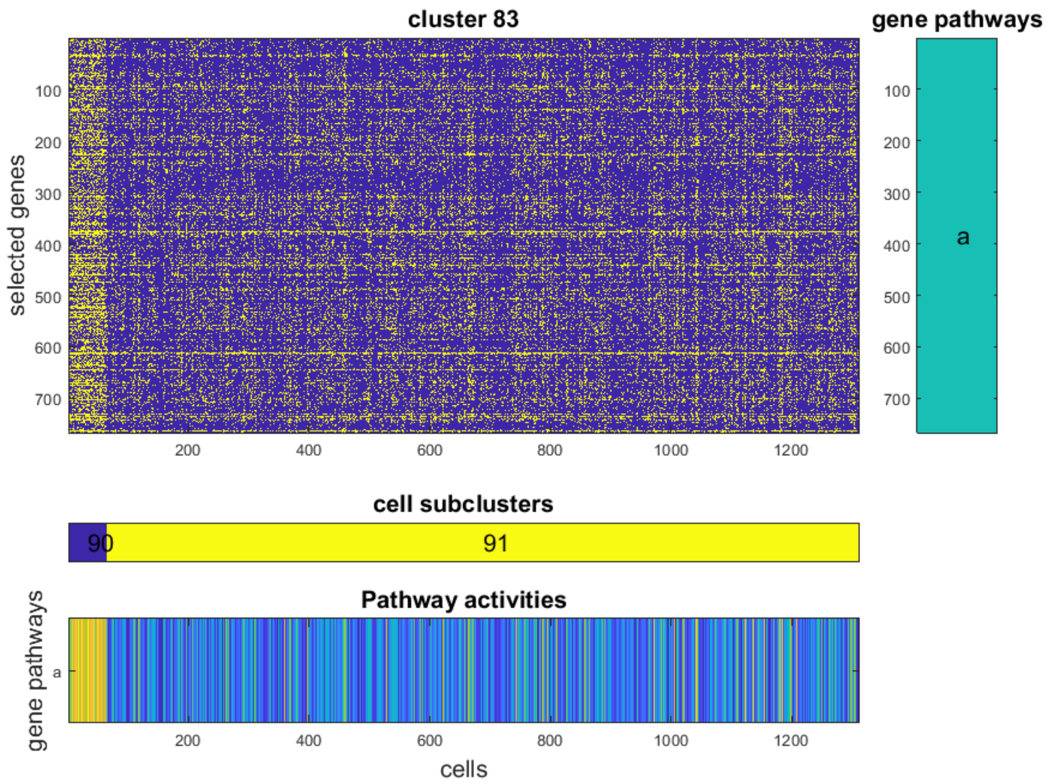


```
Remaining clusters to partition 8
Processing cluster 84 now ...
Processing data subset with 18134 genes and 1036 cells:
Remove genes detected in <100 cells. Remaining 384 genes.  Elapsed time is 0.052057 seconds.
Iterate 10 random permutations for gene-gene similarity threshold ...   10 Elapsed time is 0.332684 seconds.
Compute gene-gene similarity ... Elapsed time is 0.010629 seconds.
Create gene-gene graph for clustering genes ...
Writing graph into file ... 100%Elapsed time is 0.023494 seconds.
Running ModularityOptimizer for clustering ...Elapsed time is 0.309258 seconds.
Gene-gene graph contains 4 pathways, 251 genes in total
Elapsed time is 0.320568 seconds.
Create cell-cell graph for clustering cells ...
Writing graph into file ... 100%Elapsed time is 0.119680 seconds.
Running ModularityOptimizer for clustering ...Elapsed time is 0.641378 seconds.
Cell-cell graph contains 10 cell types by community detection
Elapsed time is 0.665115 seconds.
Cell-cell graph contains 10 cell types after merging tiny cell clusters
creating a total of 9 edges ...      9
Cell-cell graph contains 2 cell types after merging
Number of useful pathways is 1
```

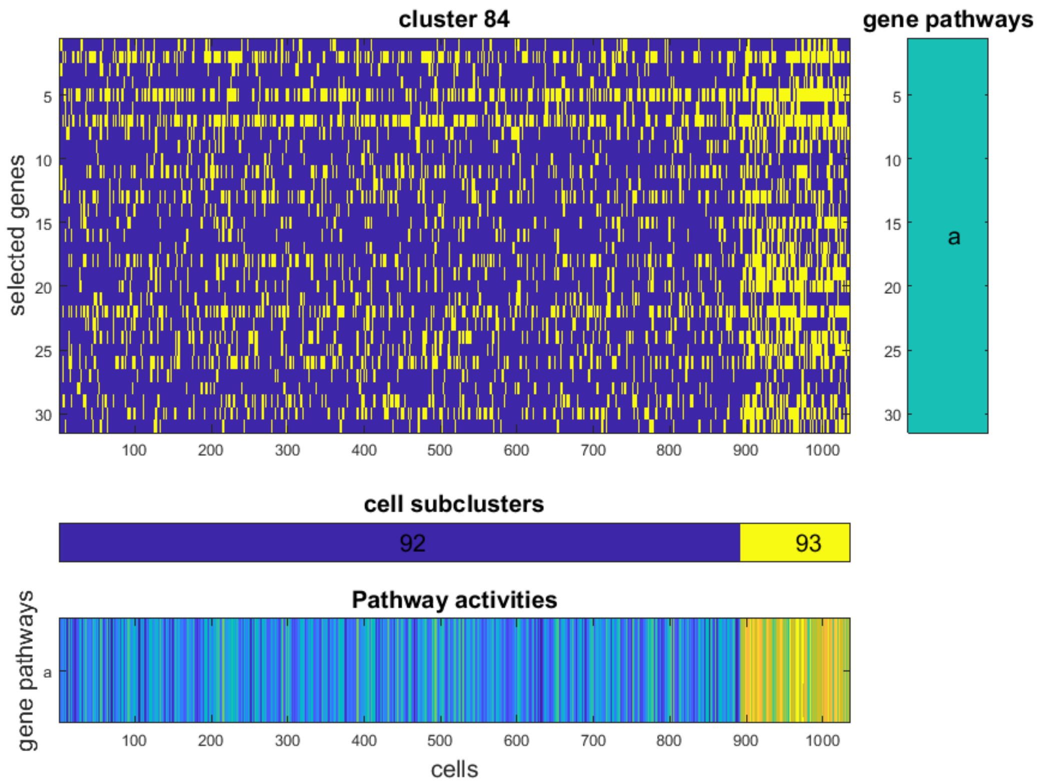


```
Remaining clusters to partition 9
Processing cluster 85 now ...
Processing data subset with 18134 genes and 7541 cells:
Remove genes detected in <100 cells. Remaining 1520 genes.  Elapsed time is 0.392369 seconds.
Iterate 10 random permutations for gene-gene similarity threshold ...   10 Elapsed time is 11.688405 seconds.
Compute gene-gene similarity ... Elapsed time is 0.394027 seconds.
Create gene-gene graph for clustering genes ...
Writing graph into file ... 100%Elapsed time is 0.058344 seconds.
Running ModularityOptimizer for clustering ...Elapsed time is 0.431513 seconds.
Gene-gene graph contains 7 pathways, 829 genes in total
Elapsed time is 0.469098 seconds.
Create cell-cell graph for clustering cells ...
Writing graph into file ... 100%Elapsed time is 0.891727 seconds.
Running ModularityOptimizer for clustering ...Elapsed time is 6.266348 seconds.
Cell-cell graph contains 17 cell types by community detection
Elapsed time is 6.416574 seconds.
Cell-cell graph contains 17 cell types after merging tiny cell clusters
Cell-cell graph contains 2 cell types after merging
Number of useful pathways is 1
```

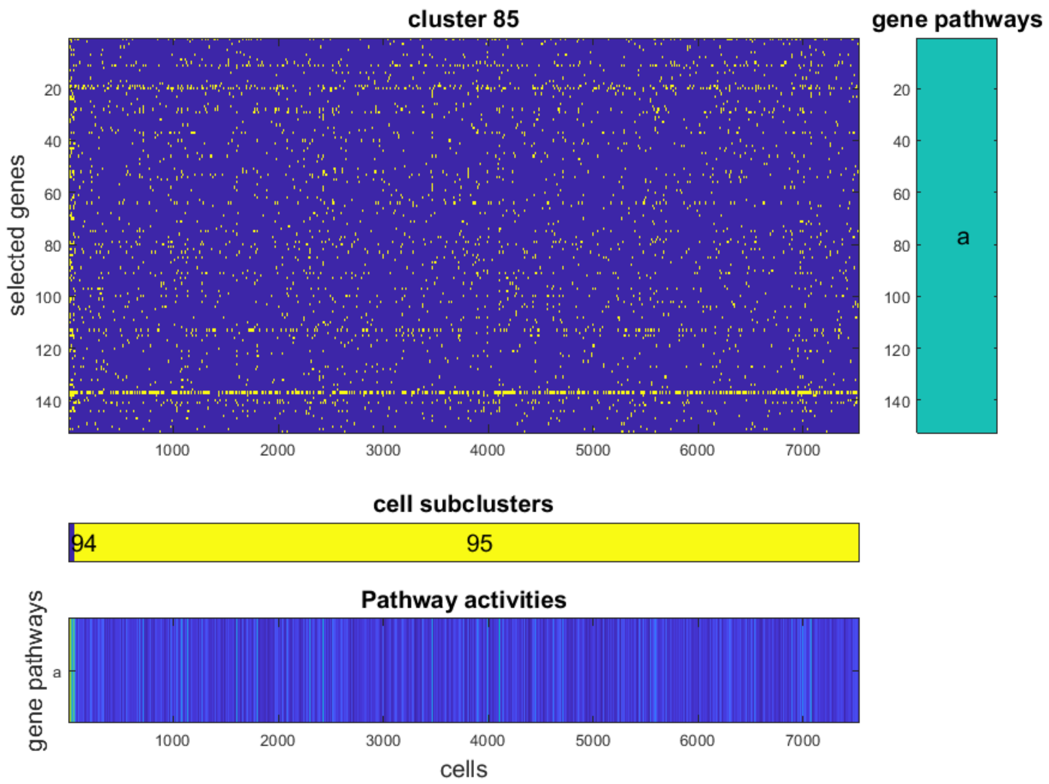


```
Remaining clusters to partition 10
Processing cluster 86 now ...
Processing data subset with 18134 genes and 801 cells:
Remove genes detected in <100 cells. Remaining 4407 genes.  Elapsed time is 0.056840 seconds.
Iterate 10 random permutations for gene-gene similarity threshold ...   10 Elapsed time is 16.210388 seconds.
Compute gene-gene similarity ... Elapsed time is 1.200057 seconds.
Create gene-gene graph for clustering genes ...
Writing graph into file ... 100%Elapsed time is 0.284638 seconds.
Running ModularityOptimizer for clustering ...Elapsed time is 1.456567 seconds.
Gene-gene graph contains 6 pathways, 3238 genes in total
Elapsed time is 1.580484 seconds.
Create cell-cell graph for clustering cells ...
Writing graph into file ... 100%Elapsed time is 0.091335 seconds.
Running ModularityOptimizer for clustering ...Elapsed time is 0.520618 seconds.
Cell-cell graph contains 9 cell types by community detection
Elapsed time is 0.538525 seconds.
Cell-cell graph contains 9 cell types after merging tiny cell clusters
creating a total of 8 edges ...      8
Cell-cell graph contains 1 cell types after merging

Remaining clusters to partition 9
Processing cluster 87 now ...
Processing data subset with 18134 genes and 160 cells:
Remove genes detected in <100 cells. Remaining 0 genes.  Elapsed time is 0.007806 seconds.

Remaining clusters to partition 8
Processing cluster 88 now ...
Processing data subset with 18134 genes and 49 cells:
Remove genes detected in <100 cells. Remaining 0 genes.  Elapsed time is 0.001989 seconds.

Remaining clusters to partition 7
Processing cluster 89 now ...
Processing data subset with 18134 genes and 33952 cells:
Remove genes detected in <100 cells. Remaining 13990 genes.  Elapsed time is 2.965241 seconds.
Iterate 10 random permutations for gene-gene similarity threshold ...   10 Elapsed time is 1412.799758 seconds.
Compute gene-gene similarity ... Elapsed time is 78.138680 seconds.
Create gene-gene graph for clustering genes ...
Writing graph into file ... 100%Elapsed time is 135.815645 seconds.
Running ModularityOptimizer for clustering ...Elapsed time is 381.425202 seconds.
Gene-gene graph contains 5 pathways, 13906 genes in total
Elapsed time is 382.044727 seconds.
Create cell-cell graph for clustering cells ...
Writing graph into file ... 100%Elapsed time is 3.855180 seconds.
Running ModularityOptimizer for clustering ...Elapsed time is 36.881925 seconds.
Cell-cell graph contains 23 cell types by community detection
Elapsed time is 37.539407 seconds.
Cell-cell graph contains 14 cell types after merging tiny cell clusters
Cell-cell graph contains 2 cell types after merging
Number of useful pathways is 1
```

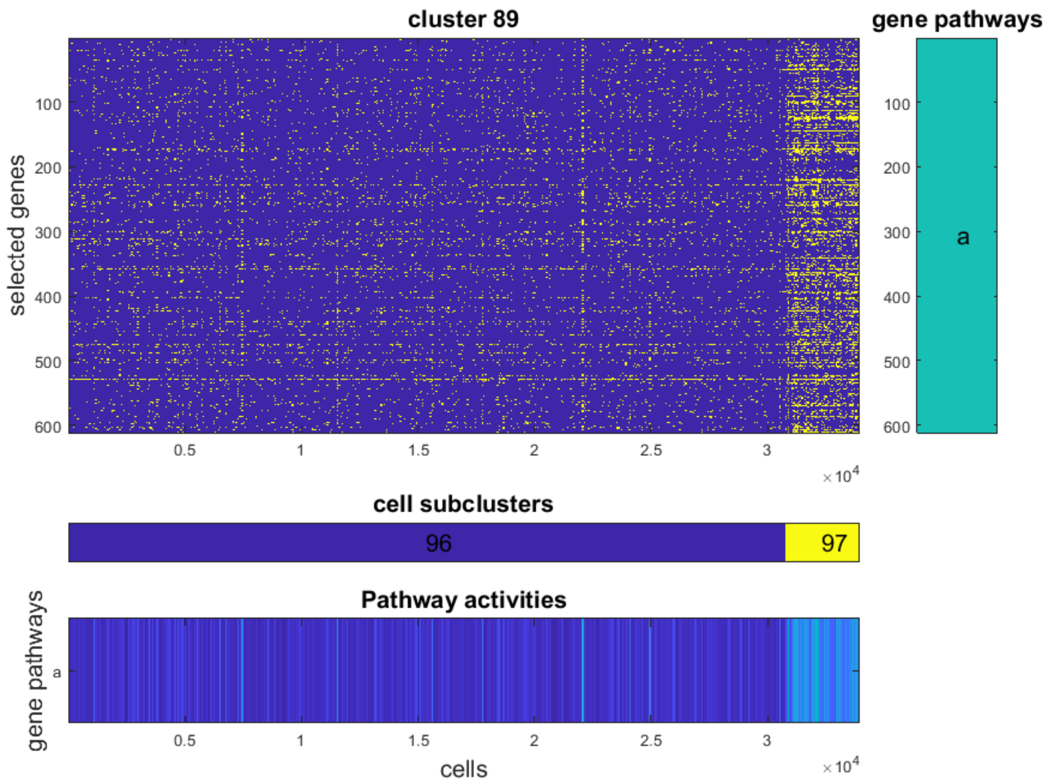


```
Remaining clusters to partition 8
Processing cluster 90 now ...
Processing data subset with 18134 genes and 64 cells:
Remove genes detected in <100 cells. Remaining 0 genes.  Elapsed time is 0.003631 seconds.

Remaining clusters to partition 7
Processing cluster 91 now ...
Processing data subset with 18134 genes and 1248 cells:
Remove genes detected in <100 cells. Remaining 2596 genes.  Elapsed time is 0.079411 seconds.
Iterate 10 random permutations for gene-gene similarity threshold ...   10 Elapsed time is 7.249316 seconds.
Compute gene-gene similarity ... Elapsed time is 0.454642 seconds.
Create gene-gene graph for clustering genes ...
Writing graph into file ... 100%Elapsed time is 0.331846 seconds.
Running ModularityOptimizer for clustering ...Elapsed time is 1.226581 seconds.
Gene-gene graph contains 4 pathways, 2268 genes in total
Elapsed time is 1.293562 seconds.
Create cell-cell graph for clustering cells ...
Writing graph into file ... 100%Elapsed time is 0.141117 seconds.
Running ModularityOptimizer for clustering ...Elapsed time is 0.656387 seconds.
Cell-cell graph contains 10 cell types by community detection
Elapsed time is 0.687159 seconds.
Cell-cell graph contains 9 cell types after merging tiny cell clusters
creating a total of 8 edges ...      8
Cell-cell graph contains 2 cell types after merging
Number of useful pathways is 1
```

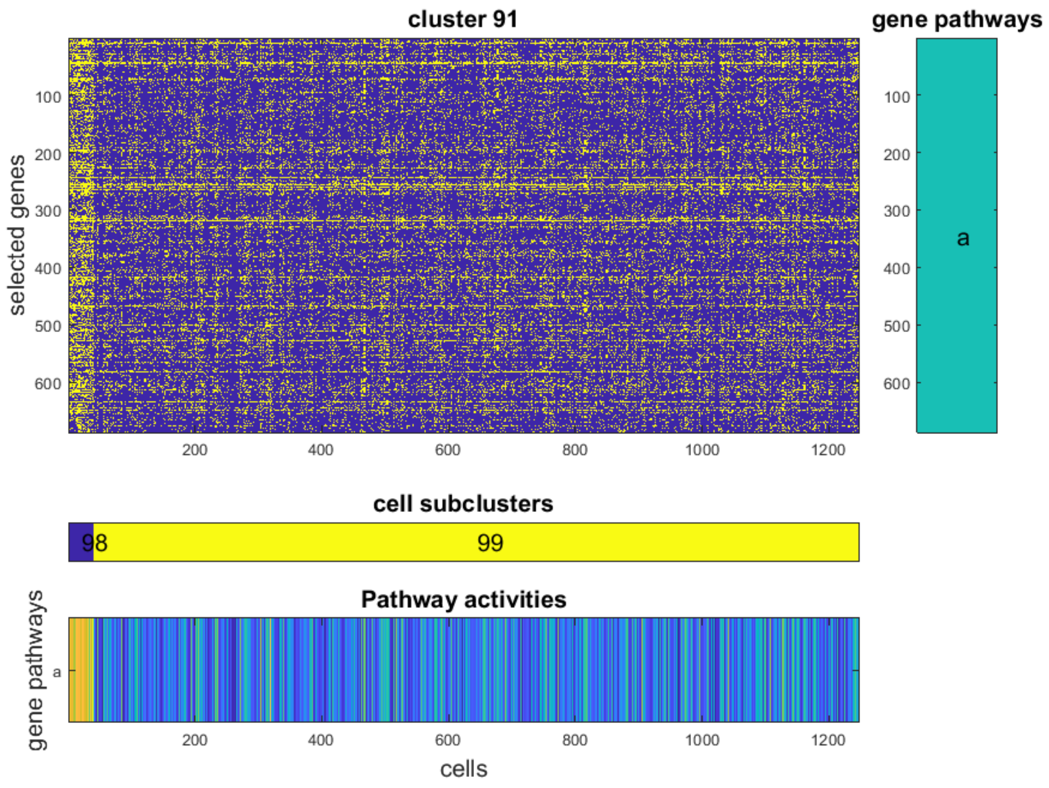


```
Remaining clusters to partition 8
Processing cluster 92 now ...
Processing data subset with 18134 genes and 892 cells:
Remove genes detected in <100 cells. Remaining 327 genes.  Elapsed time is 0.046488 seconds.
Iterate 10 random permutations for gene-gene similarity threshold ...   10 Elapsed time is 0.239650 seconds.
Compute gene-gene similarity ... Elapsed time is 0.006691 seconds.
Create gene-gene graph for clustering genes ...
Writing graph into file ... 100%Elapsed time is 0.019688 seconds.
Running ModularityOptimizer for clustering ...Elapsed time is 0.296084 seconds.
Gene-gene graph contains 5 pathways, 218 genes in total
Elapsed time is 0.306361 seconds.
Create cell-cell graph for clustering cells ...
Writing graph into file ... 100%Elapsed time is 0.102951 seconds.
Running ModularityOptimizer for clustering ...Elapsed time is 0.706782 seconds.
Cell-cell graph contains 9 cell types by community detection
Elapsed time is 0.728050 seconds.
Cell-cell graph contains 9 cell types after merging tiny cell clusters
creating a total of 8 edges ...      8
Cell-cell graph contains 2 cell types after merging
Number of useful pathways is 1
```

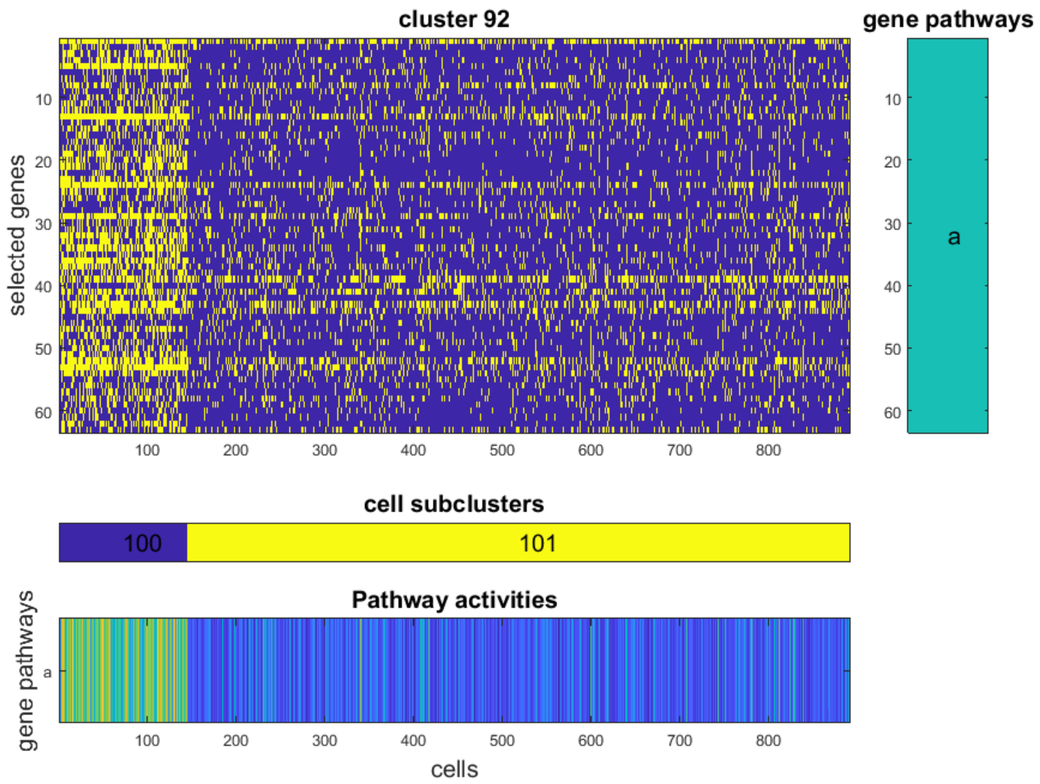


```
Remaining clusters to partition 9
Processing cluster 93 now ...
Processing data subset with 18134 genes and 144 cells:
Remove genes detected in <100 cells. Remaining 0 genes.  Elapsed time is 0.008393 seconds.

Remaining clusters to partition 8
Processing cluster 94 now ...
Processing data subset with 18134 genes and 56 cells:
Remove genes detected in <100 cells. Remaining 0 genes.  Elapsed time is 0.002725 seconds.

Remaining clusters to partition 7
Processing cluster 95 now ...
Processing data subset with 18134 genes and 7485 cells:
Remove genes detected in <100 cells. Remaining 1481 genes.  Elapsed time is 0.437032 seconds.
Iterate 10 random permutations for gene-gene similarity threshold ...   10 Elapsed time is 11.805629 seconds.
Compute gene-gene similarity ... Elapsed time is 0.372801 seconds.
Create gene-gene graph for clustering genes ...
Writing graph into file ... 100%Elapsed time is 0.055382 seconds.
Running ModularityOptimizer for clustering ...Elapsed time is 0.412157 seconds.
Gene-gene graph contains 7 pathways, 816 genes in total
Elapsed time is 0.448242 seconds.
Create cell-cell graph for clustering cells ...
Writing graph into file ... 100%Elapsed time is 0.873097 seconds.
Running ModularityOptimizer for clustering ...Elapsed time is 5.707494 seconds.
Cell-cell graph contains 18 cell types by community detection
Elapsed time is 5.859207 seconds.
Cell-cell graph contains 18 cell types after merging tiny cell clusters
creating a total of 17 edges ...     17
Cell-cell graph contains 2 cell types after merging
Number of useful pathways is 1
```

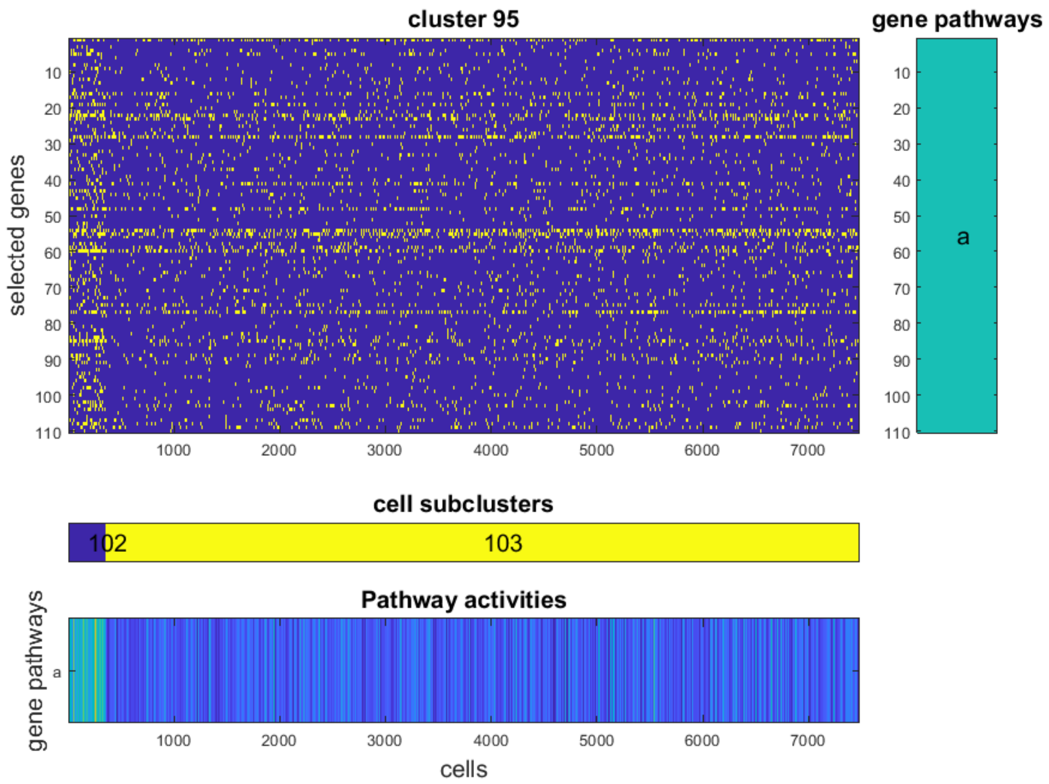


```
Remaining clusters to partition 8
Processing cluster 96 now ...
Processing data subset with 18134 genes and 30690 cells:
Remove genes detected in <100 cells. Remaining 13811 genes.  Elapsed time is 2.673791 seconds.
Iterate 10 random permutations for gene-gene similarity threshold ...   10 Elapsed time is 1260.600236 seconds.
Compute gene-gene similarity ... Elapsed time is 76.949664 seconds.
Create gene-gene graph for clustering genes ...
Writing graph into file ... 100%Elapsed time is 142.155903 seconds.
Running ModularityOptimizer for clustering ...Elapsed time is 426.494504 seconds.
Gene-gene graph contains 4 pathways, 13736 genes in total
Elapsed time is 427.114747 seconds.
Create cell-cell graph for clustering cells ...
Writing graph into file ... 100%Elapsed time is 3.401981 seconds.
Running ModularityOptimizer for clustering ...Elapsed time is 30.863247 seconds.
Cell-cell graph contains 27 cell types by community detection
Elapsed time is 31.499615 seconds.
Cell-cell graph contains 12 cell types after merging tiny cell clusters
creating a total of 11 edges ...     11
Cell-cell graph contains 2 cell types after merging
Number of useful pathways is 1
```

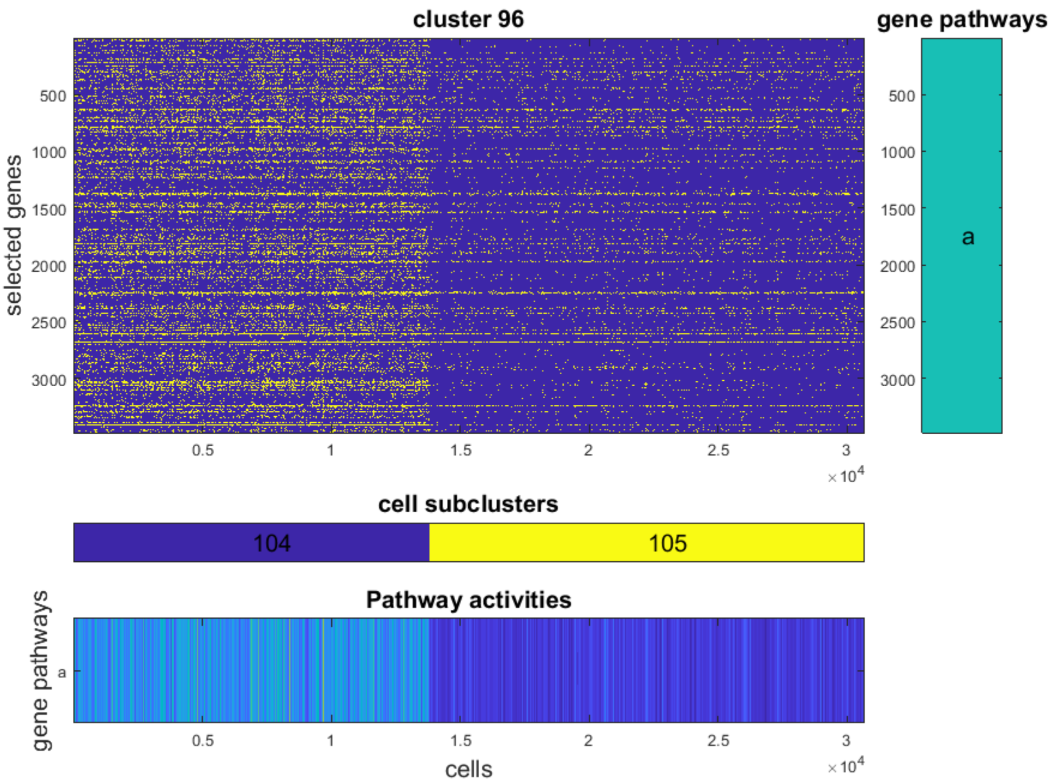


```
Remaining clusters to partition 9
Processing cluster 97 now ...
Processing data subset with 18134 genes and 3262 cells:
Remove genes detected in <100 cells. Remaining 7546 genes.  Elapsed time is 0.246086 seconds.
Iterate 10 random permutations for gene-gene similarity threshold ...   10 Elapsed time is 75.272458 seconds.
Compute gene-gene similarity ... Elapsed time is 5.149352 seconds.
Create gene-gene graph for clustering genes ...
Writing graph into file ... 100%Elapsed time is 1.516969 seconds.
Running ModularityOptimizer for clustering ...Elapsed time is 4.511173 seconds.
Gene-gene graph contains 6 pathways, 5422 genes in total
Elapsed time is 4.758456 seconds.
Create cell-cell graph for clustering cells ...
Writing graph into file ... 100%Elapsed time is 0.376771 seconds.
Running ModularityOptimizer for clustering ...Elapsed time is 1.770079 seconds.
Cell-cell graph contains 16 cell types by community detection
Elapsed time is 1.846600 seconds.
Cell-cell graph contains 16 cell types after merging tiny cell clusters
creating a total of 15 edges ...     15
Cell-cell graph contains 2 cell types after merging
Number of useful pathways is 1
```

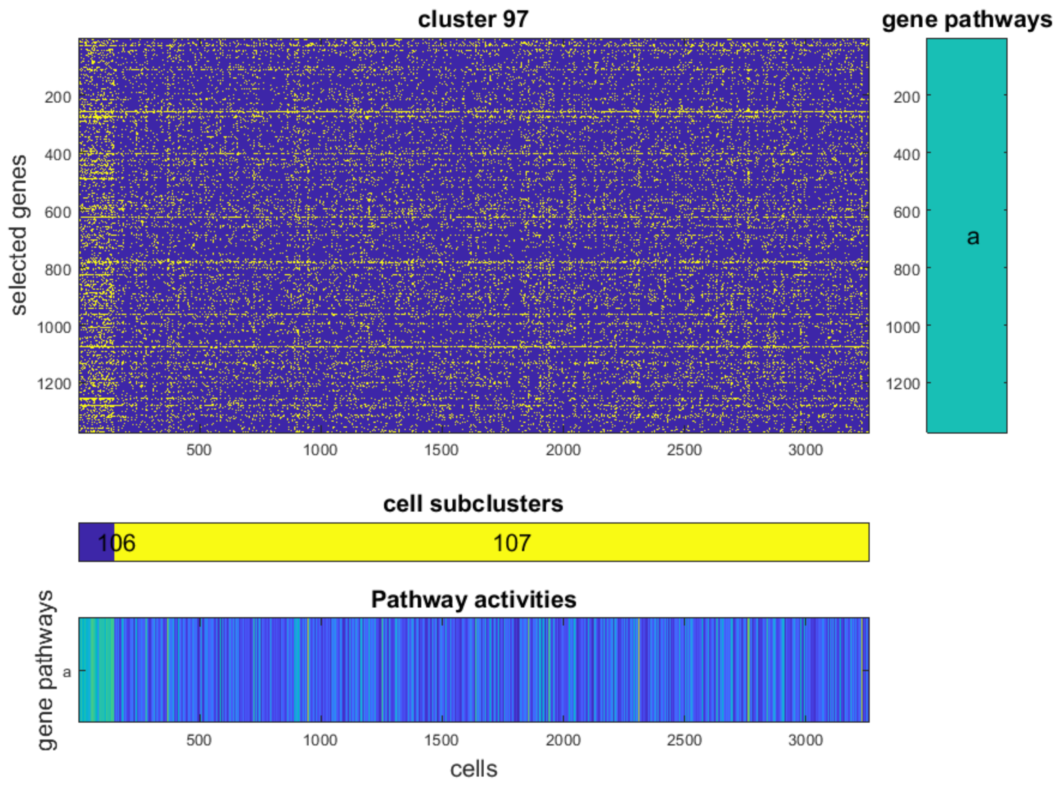


```
Remaining clusters to partition 10
Processing cluster 98 now ...
Processing data subset with 18134 genes and 40 cells:
Remove genes detected in <100 cells. Remaining 0 genes.  Elapsed time is 0.001667 seconds.

Remaining clusters to partition 9
Processing cluster 99 now ...
Processing data subset with 18134 genes and 1208 cells:
Remove genes detected in <100 cells. Remaining 2456 genes.  Elapsed time is 0.077457 seconds.
Iterate 10 random permutations for gene-gene similarity threshold ...   10 Elapsed time is 6.756030 seconds.
Compute gene-gene similarity ... Elapsed time is 0.404484 seconds.
Create gene-gene graph for clustering genes ...
Writing graph into file ... 100%Elapsed time is 0.240937 seconds.
Running ModularityOptimizer for clustering ...Elapsed time is 0.942011 seconds.
Gene-gene graph contains 4 pathways, 2019 genes in total
Elapsed time is 1.008668 seconds.
Create cell-cell graph for clustering cells ...
Writing graph into file ... 100%Elapsed time is 0.160823 seconds.
Running ModularityOptimizer for clustering ...Elapsed time is 0.738535 seconds.
Cell-cell graph contains 13 cell types by community detection
Elapsed time is 0.769122 seconds.
Cell-cell graph contains 11 cell types after merging tiny cell clusters
creating a total of 10 edges ...     10
Cell-cell graph contains 1 cell types after merging

Remaining clusters to partition 8
Processing cluster 100 now ...
Processing data subset with 18134 genes and 144 cells:
Remove genes detected in <100 cells. Remaining 0 genes.  Elapsed time is 0.007293 seconds.

Remaining clusters to partition 7
Processing cluster 101 now ...
Processing data subset with 18134 genes and 748 cells:
Remove genes detected in <100 cells. Remaining 211 genes.  Elapsed time is 0.034069 seconds.
Iterate 10 random permutations for gene-gene similarity threshold ...   10 Elapsed time is 0.112300 seconds.
Compute gene-gene similarity ... Elapsed time is 0.003031 seconds.
Create gene-gene graph for clustering genes ...
Writing graph into file ... 100%Elapsed time is 0.003013 seconds.
Running ModularityOptimizer for clustering ...Elapsed time is 0.206633 seconds.
Gene-gene graph contains 2 pathways, 51 genes in total
Elapsed time is 0.217610 seconds.
Create cell-cell graph for clustering cells ...
Writing graph into file ... 100%Elapsed time is 0.083356 seconds.
Running ModularityOptimizer for clustering ...Elapsed time is 0.563093 seconds.
Cell-cell graph contains 9 cell types by community detection
Elapsed time is 0.584608 seconds.
Cell-cell graph contains 2 cell types after merging tiny cell clusters
creating a total of 1 edges ...      1
Cell-cell graph contains 1 cell types after merging

Remaining clusters to partition 6
Processing cluster 102 now ...
Processing data subset with 18134 genes and 355 cells:
Remove genes detected in <100 cells. Remaining 97 genes.  Elapsed time is 0.016885 seconds.
Iterate 10 random permutations for gene-gene similarity threshold ...   10 Elapsed time is 0.026647 seconds.
Compute gene-gene similarity ... Elapsed time is 0.000958 seconds.
```

```
Create gene-gene graph for clustering genes ...
Writing graph into file ... 107%Elapsed time is 0.001402 seconds.
Running ModularityOptimizer for clustering ...Elapsed time is 0.171890 seconds.
Gene-gene graph contains 0 pathways, 0 genes in total
Elapsed time is 0.180043 seconds.

Remaining clusters to partition 5
Processing cluster 103 now ...
Processing data subset with 18134 genes and 7130 cells:
Remove genes detected in <100 cells. Remaining 1386 genes.  Elapsed time is 0.380902 seconds.
Iterate 10 random permutations for gene-gene similarity threshold ...   10 Elapsed time is 10.026251 seconds.
Compute gene-gene similarity ... Elapsed time is 0.353399 seconds.
Create gene-gene graph for clustering genes ...
Writing graph into file ... 100%Elapsed time is 0.048119 seconds.
Running ModularityOptimizer for clustering ...Elapsed time is 0.390690 seconds.
Gene-gene graph contains 5 pathways, 662 genes in total
Elapsed time is 0.428912 seconds.
Create cell-cell graph for clustering cells ...
Writing graph into file ... 100%Elapsed time is 0.850421 seconds.
Running ModularityOptimizer for clustering ...Elapsed time is 5.056712 seconds.
Cell-cell graph contains 16 cell types by community detection
Elapsed time is 5.226379 seconds.
Cell-cell graph contains 15 cell types after merging tiny cell clusters
creating a total of 14 edges ...     14
Cell-cell graph contains 2 cell types after merging
Number of useful pathways is 1
```

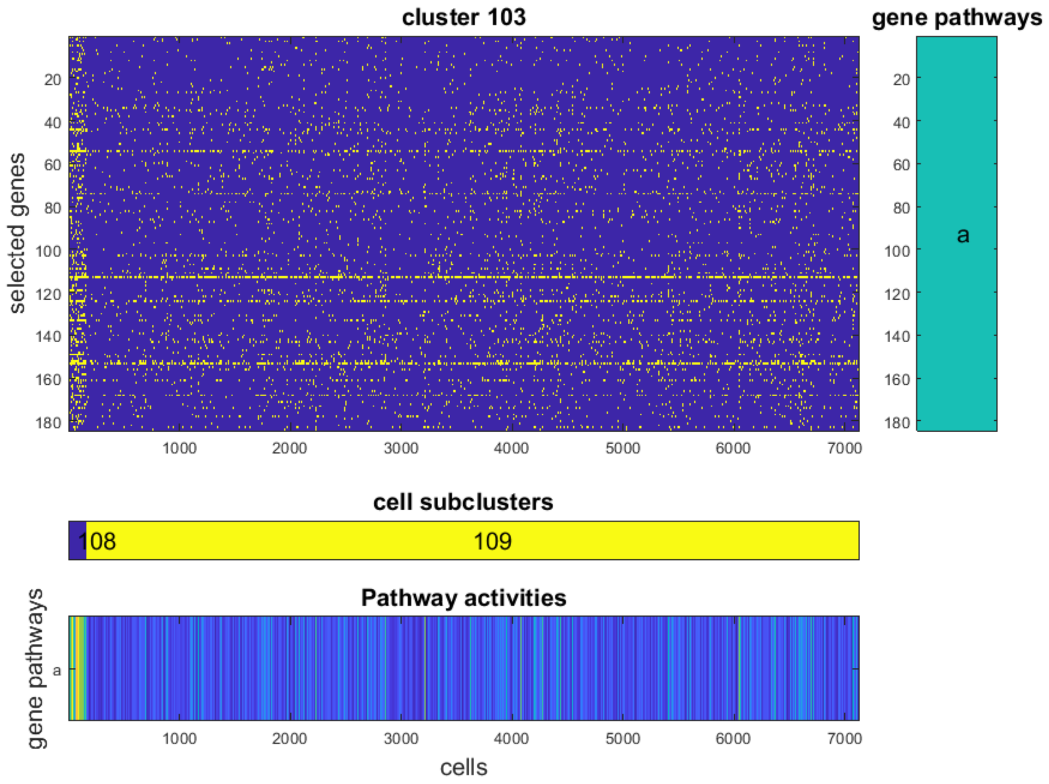


```
Remaining clusters to partition 6
Processing cluster 104 now ...
Processing data subset with 18134 genes and 13820 cells:
Remove genes detected in <100 cells. Remaining 12618 genes.  Elapsed time is 1.213747 seconds.
Iterate 10 random permutations for gene-gene similarity threshold ...   10 Elapsed time is 506.844349 seconds.
Compute gene-gene similarity ... Elapsed time is 32.709640 seconds.
Create gene-gene graph for clustering genes ...
Writing graph into file ... 100%Elapsed time is 143.598590 seconds.
Running ModularityOptimizer for clustering ...Elapsed time is 527.533535 seconds.
Gene-gene graph contains 4 pathways, 12532 genes in total
Elapsed time is 528.083662 seconds.
Create cell-cell graph for clustering cells ...
Writing graph into file ... 100%Elapsed time is 1.526255 seconds.
Running ModularityOptimizer for clustering ...Elapsed time is 8.882741 seconds.
Cell-cell graph contains 24 cell types by community detection
Elapsed time is 9.168928 seconds.
Cell-cell graph contains 21 cell types after merging tiny cell clusters
Cell-cell graph contains 2 cell types after merging
Number of useful pathways is 3
```

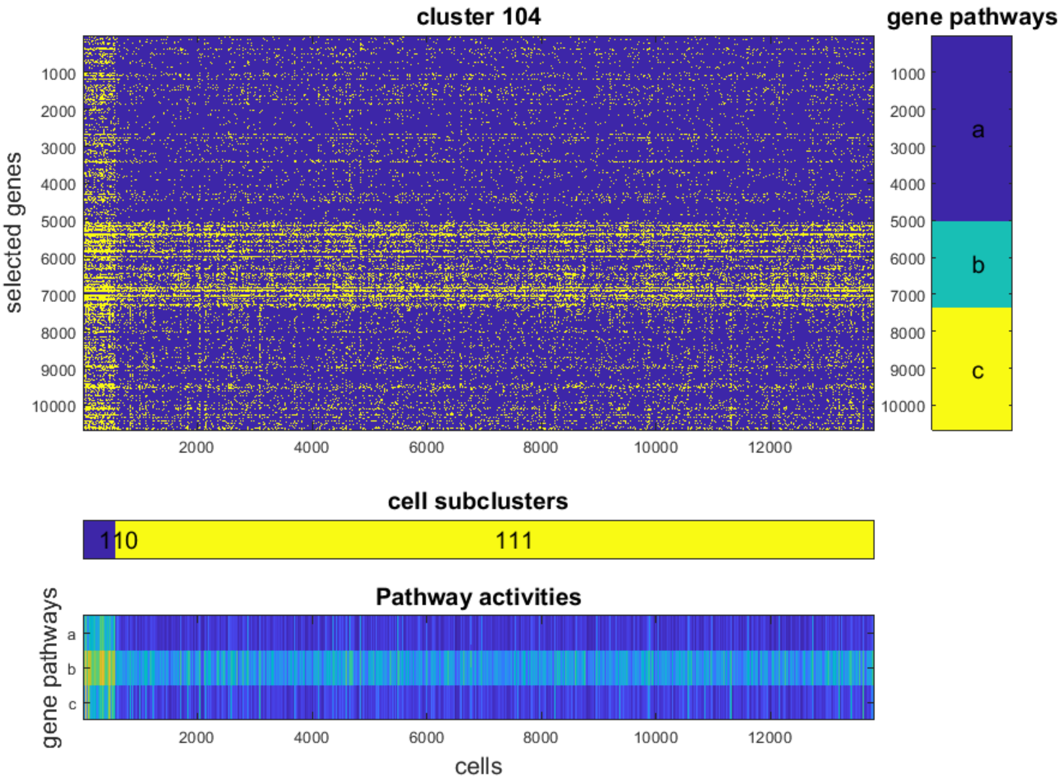


```
Remaining clusters to partition 7
Processing cluster 105 now ...
Processing data subset with 18134 genes and 16870 cells:
Remove genes detected in <100 cells. Remaining 10566 genes.  Elapsed time is 1.368929 seconds.
Iterate 10 random permutations for gene-gene similarity threshold ...   10 Elapsed time is 447.856720 seconds.
Compute gene-gene similarity ... Elapsed time is 27.485016 seconds.
Create gene-gene graph for clustering genes ...
Writing graph into file ... 100%Elapsed time is 11.429370 seconds.
Running ModularityOptimizer for clustering ...Elapsed time is 24.085711 seconds.
Gene-gene graph contains 6 pathways, 9059 genes in total
Elapsed time is 24.473459 seconds.
Create cell-cell graph for clustering cells ...
Writing graph into file ... 100%Elapsed time is 1.989913 seconds.
Running ModularityOptimizer for clustering ...Elapsed time is 21.898242 seconds.
Cell-cell graph contains 19 cell types by community detection
Elapsed time is 22.231473 seconds.
Cell-cell graph contains 16 cell types after merging tiny cell clusters
Cell-cell graph contains 3 cell types after merging
Number of useful pathways is 2
```

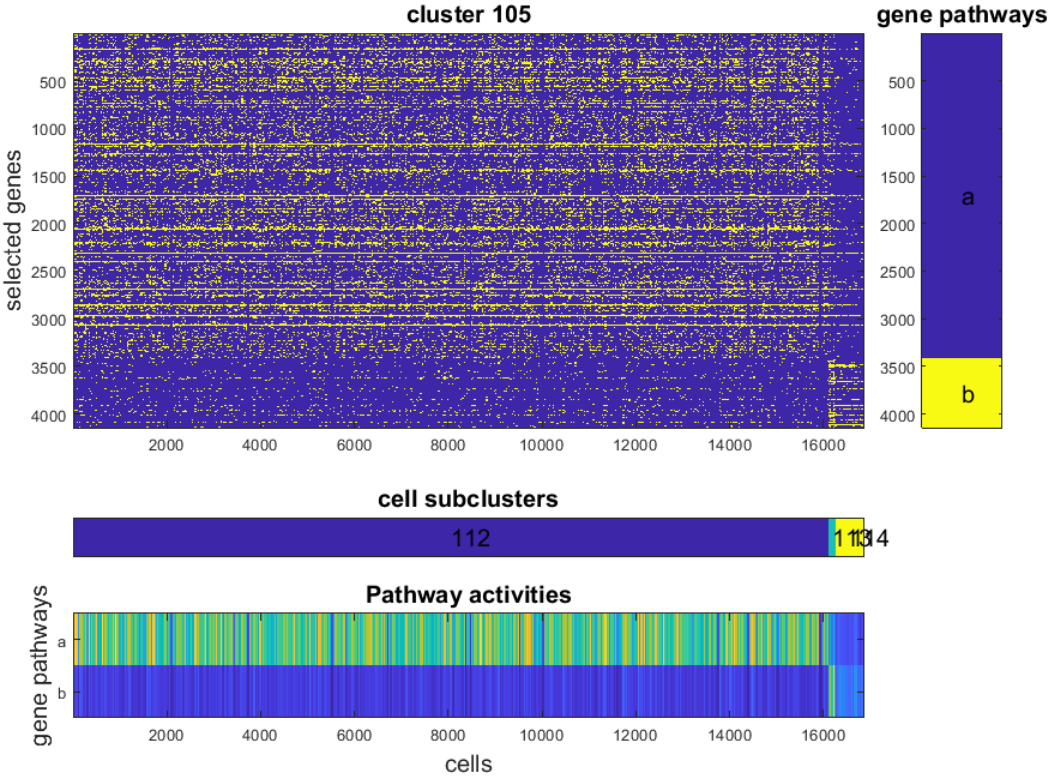


```
Remaining clusters to partition 9
Processing cluster 106 now ...
Processing data subset with 18134 genes and 148 cells:
Remove genes detected in <100 cells. Remaining 0 genes.  Elapsed time is 0.008161 seconds.

Remaining clusters to partition 8
Processing cluster 107 now ...
Processing data subset with 18134 genes and 3114 cells:
Remove genes detected in <100 cells. Remaining 7334 genes.  Elapsed time is 0.218558 seconds.
Iterate 10 random permutations for gene-gene similarity threshold ...   10 Elapsed time is 70.116542 seconds.
Compute gene-gene similarity ... Elapsed time is 4.588137 seconds.
Create gene-gene graph for clustering genes ...
Writing graph into file ... 100%Elapsed time is 1.362950 seconds.
Running ModularityOptimizer for clustering ...Elapsed time is 3.641762 seconds.
Gene-gene graph contains 5 pathways, 5220 genes in total
Elapsed time is 3.876035 seconds.
Create cell-cell graph for clustering cells ...
Writing graph into file ... 100%Elapsed time is 0.349451 seconds.
Running ModularityOptimizer for clustering ...Elapsed time is 1.773209 seconds.
Cell-cell graph contains 13 cell types by community detection
Elapsed time is 1.845773 seconds.
Cell-cell graph contains 10 cell types after merging tiny cell clusters
creating a total of 9 edges ...      9
Cell-cell graph contains 1 cell types after merging

Remaining clusters to partition 7
Processing cluster 108 now ...
Processing data subset with 18134 genes and 162 cells:
Remove genes detected in <100 cells. Remaining 0 genes.  Elapsed time is 0.010428 seconds.

Remaining clusters to partition 6
Processing cluster 109 now ...
Processing data subset with 18134 genes and 6968 cells:
Remove genes detected in <100 cells. Remaining 1324 genes.  Elapsed time is 0.389823 seconds.
Iterate 10 random permutations for gene-gene similarity threshold ...   10 Elapsed time is 9.962618 seconds.
Compute gene-gene similarity ... Elapsed time is 0.359310 seconds.
Create gene-gene graph for clustering genes ...
Writing graph into file ... 100%Elapsed time is 0.044251 seconds.
Running ModularityOptimizer for clustering ...Elapsed time is 0.396307 seconds.
Gene-gene graph contains 6 pathways, 628 genes in total
Elapsed time is 0.432654 seconds.
Create cell-cell graph for clustering cells ...
Writing graph into file ... 100%Elapsed time is 0.818561 seconds.
Running ModularityOptimizer for clustering ...Elapsed time is 5.607819 seconds.
Cell-cell graph contains 12 cell types by community detection
Elapsed time is 5.756755 seconds.
Cell-cell graph contains 11 cell types after merging tiny cell clusters
creating a total of 10 edges ...     10
Cell-cell graph contains 1 cell types after merging

Remaining clusters to partition 5
Processing cluster 110 now ...
Processing data subset with 18134 genes and 560 cells:
Remove genes detected in <100 cells. Remaining 7059 genes.  Elapsed time is 0.041714 seconds.
Iterate 10 random permutations for gene-gene similarity threshold ...   10 Elapsed time is 37.277679 seconds.
Compute gene-gene similarity ... Elapsed time is 2.871091 seconds.
```

```
Create gene-gene graph for clustering genes ...
Writing graph into file ... 100%Elapsed time is 1.042791 seconds.
Running ModularityOptimizer for clustering ...Elapsed time is 2.536745 seconds.
Gene-gene graph contains 5 pathways, 4872 genes in total
Elapsed time is 2.759608 seconds.
Create cell-cell graph for clustering cells ...
Writing graph into file ... 100%Elapsed time is 0.059109 seconds.
Running ModularityOptimizer for clustering ...Elapsed time is 0.372808 seconds.
Cell-cell graph contains 10 cell types by community detection
Elapsed time is 0.390246 seconds.
Cell-cell graph contains 10 cell types after merging tiny cell clusters
creating a total of 9 edges ...      9
Cell-cell graph contains 1 cell types after merging

Remaining clusters to partition 4
Processing cluster 111 now ...
Processing data subset with 18134 genes and 13260 cells:
Remove genes detected in <100 cells. Remaining 12107 genes.  Elapsed time is 1.103489 seconds.
Iterate 10 random permutations for gene-gene similarity threshold ...   10 Elapsed time is 451.541912 seconds.
Compute gene-gene similarity ... Elapsed time is 28.777945 seconds.
Create gene-gene graph for clustering genes ...
Writing graph into file ... 100%Elapsed time is 31.915783 seconds.
Running ModularityOptimizer for clustering ...Elapsed time is 86.649877 seconds.
Gene-gene graph contains 6 pathways, 11526 genes in total
Elapsed time is 87.123643 seconds.
Create cell-cell graph for clustering cells ...
Writing graph into file ... 100%Elapsed time is 1.540307 seconds.
Running ModularityOptimizer for clustering ...Elapsed time is 10.193538 seconds.
Cell-cell graph contains 16 cell types by community detection
Elapsed time is 10.466995 seconds.
Cell-cell graph contains 14 cell types after merging tiny cell clusters
Cell-cell graph contains 2 cell types after merging
Number of useful pathways is 1
```

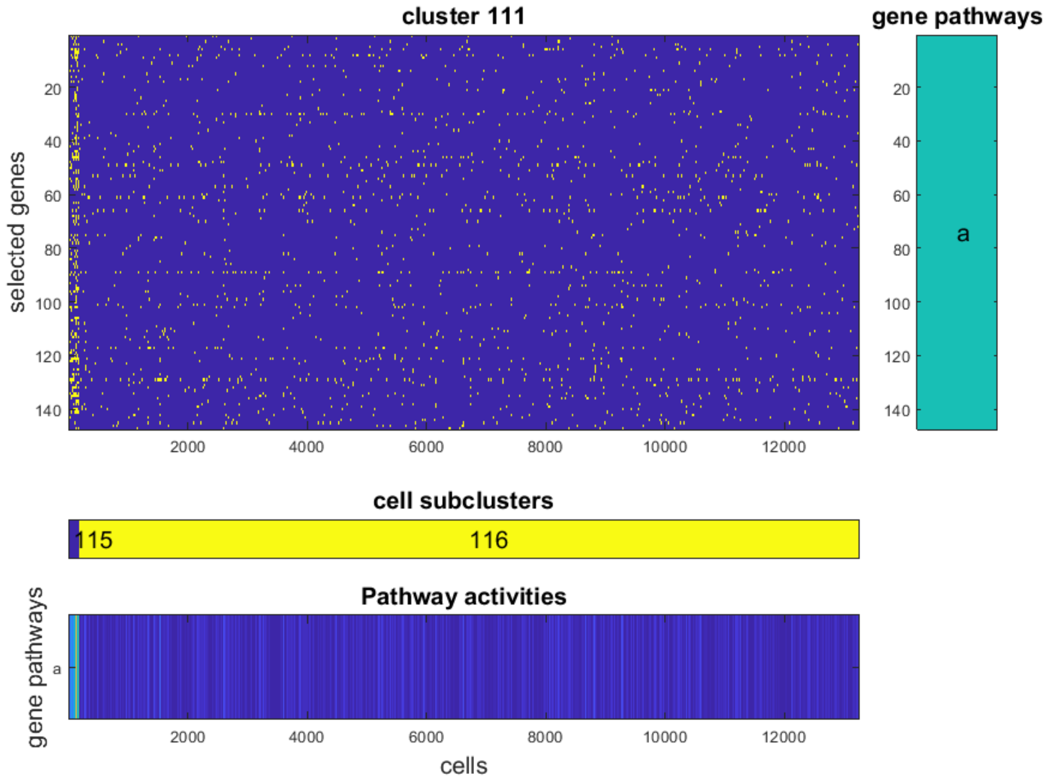


```
Remaining clusters to partition 5
Processing cluster 112 now ...
Processing data subset with 18134 genes and 16090 cells:
Remove genes detected in <100 cells. Remaining 10455 genes.  Elapsed time is 1.319445 seconds.
Iterate 10 random permutations for gene-gene similarity threshold ...   10 Elapsed time is 419.560458 seconds.
Compute gene-gene similarity ... Elapsed time is 25.158353 seconds.
Create gene-gene graph for clustering genes ...
Writing graph into file ... 100%Elapsed time is 8.268879 seconds.
Running ModularityOptimizer for clustering ...Elapsed time is 19.169554 seconds.
Gene-gene graph contains 5 pathways, 8385 genes in total
Elapsed time is 19.553252 seconds.
Create cell-cell graph for clustering cells ...
Writing graph into file ... 100%Elapsed time is 1.876710 seconds.
Running ModularityOptimizer for clustering ...Elapsed time is 14.457196 seconds.
Cell-cell graph contains 17 cell types by community detection
Elapsed time is 14.791687 seconds.
Cell-cell graph contains 15 cell types after merging tiny cell clusters
creating a total of 14 edges ...      14
Cell-cell graph contains 2 cell types after merging
Number of useful pathways is 1
```

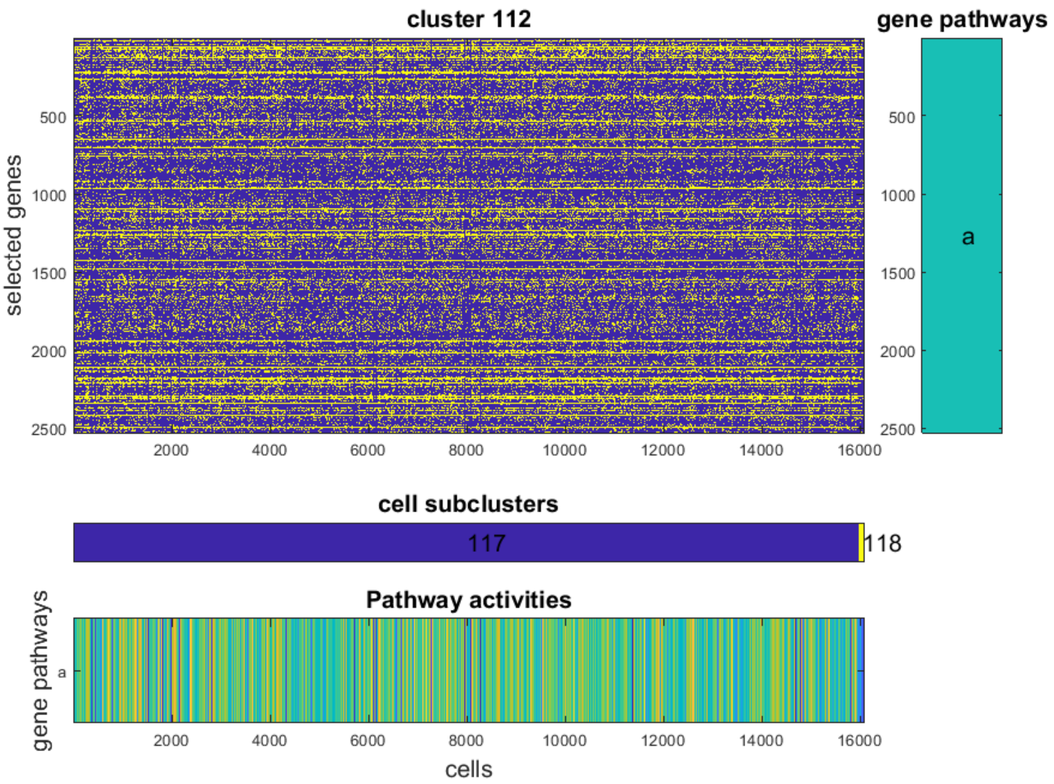


```
Remaining clusters to partition 6
Processing cluster 113 now ...
Processing data subset with 18134 genes and 180 cells:
Remove genes detected in <100 cells. Remaining 0 genes.  Elapsed time is 0.008983 seconds.

Remaining clusters to partition 5
Processing cluster 114 now ...
Processing data subset with 18134 genes and 600 cells:
Remove genes detected in <100 cells. Remaining 476 genes.  Elapsed time is 0.027775 seconds.
Iterate 10 random permutations for gene-gene similarity threshold ...   10 Elapsed time is 0.299505 seconds.
Compute gene-gene similarity ... Elapsed time is 0.012876 seconds.
Create gene-gene graph for clustering genes ...
Writing graph into file ... 100%Elapsed time is 0.016720 seconds.
Running ModularityOptimizer for clustering ...Elapsed time is 0.303273 seconds.
Gene-gene graph contains 4 pathways, 350 genes in total
Elapsed time is 0.319059 seconds.
Create cell-cell graph for clustering cells ...
Writing graph into file ... 100%Elapsed time is 0.066849 seconds.
Running ModularityOptimizer for clustering ...Elapsed time is 0.482058 seconds.
Cell-cell graph contains 7 cell types by community detection
Elapsed time is 0.500286 seconds.
Cell-cell graph contains 7 cell types after merging tiny cell clusters
creating a total of 6 edges ...      6
Cell-cell graph contains 2 cell types after merging
Number of useful pathways is 1
```

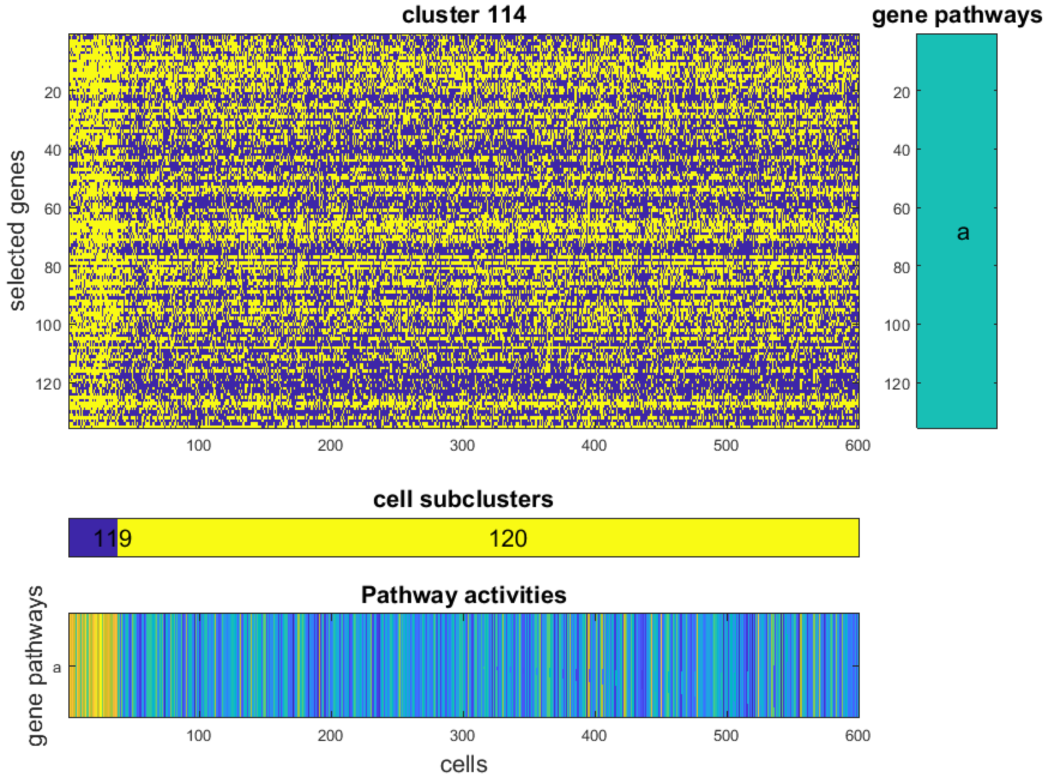


```
Remaining clusters to partition 6
Processing cluster 115 now ...
Processing data subset with 18134 genes and 176 cells:
Remove genes detected in <100 cells. Remaining 0 genes.  Elapsed time is 0.008580 seconds.

Remaining clusters to partition 5
Processing cluster 116 now ...
Processing data subset with 18134 genes and 13084 cells:
Remove genes detected in <100 cells. Remaining 12009 genes.  Elapsed time is 1.116539 seconds.
Iterate 10 random permutations for gene-gene similarity threshold ...   10 Elapsed time is 438.760789 seconds.
Compute gene-gene similarity ... Elapsed time is 28.372900 seconds.
Create gene-gene graph for clustering genes ...
Writing graph into file ... 100%Elapsed time is 30.612782 seconds.
Running ModularityOptimizer for clustering ...Elapsed time is 81.727967 seconds.
Gene-gene graph contains 5 pathways, 11404 genes in total
Elapsed time is 82.194338 seconds.
Create cell-cell graph for clustering cells ...
Writing graph into file ... 100%Elapsed time is 1.482222 seconds.
Running ModularityOptimizer for clustering ...Elapsed time is 9.219338 seconds.
Cell-cell graph contains 17 cell types by community detection
Elapsed time is 9.488919 seconds.
Cell-cell graph contains 13 cell types after merging tiny cell clusters
creating a total of 12 edges ...     12
Cell-cell graph contains 2 cell types after merging
Number of useful pathways is 1
```

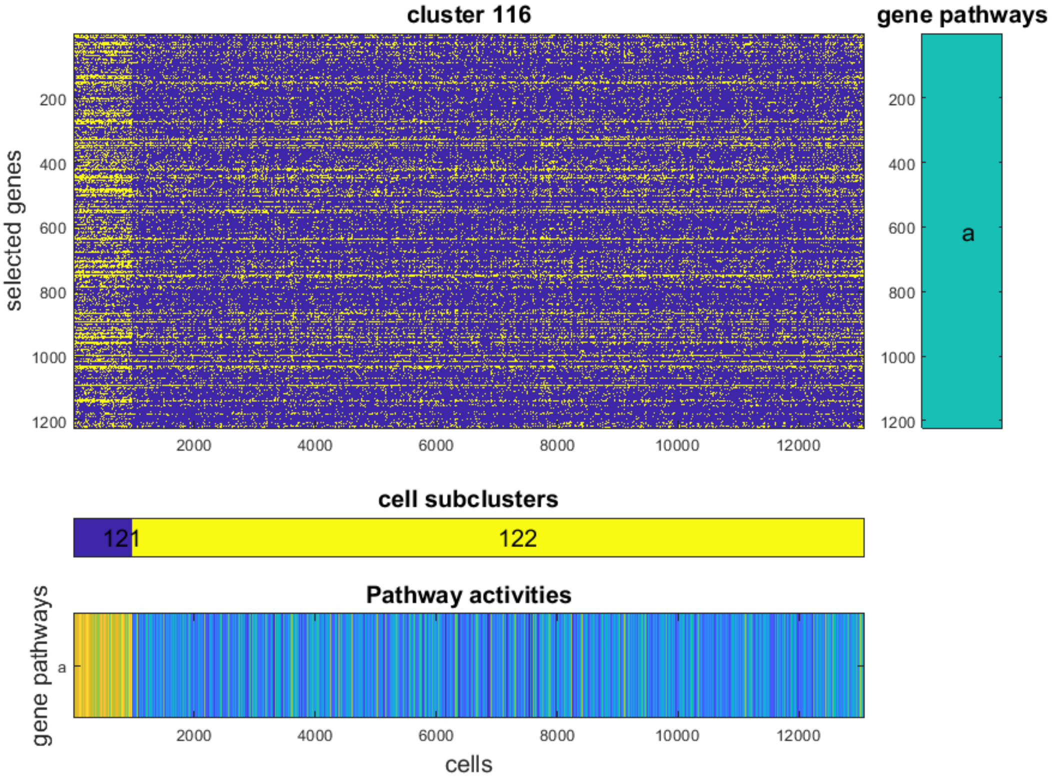


```
Remaining clusters to partition 6
Processing cluster 117 now ...
Processing data subset with 18134 genes and 15969 cells:
Remove genes detected in <100 cells. Remaining 10442 genes.  Elapsed time is 1.272948 seconds.
Iterate 10 random permutations for gene-gene similarity threshold ...   10 Elapsed time is 414.538769 seconds.
Compute gene-gene similarity ... Elapsed time is 25.272356 seconds.
Create gene-gene graph for clustering genes ...
Writing graph into file ... 100%Elapsed time is 8.179287 seconds.
Running ModularityOptimizer for clustering ...Elapsed time is 18.517813 seconds.
Gene-gene graph contains 5 pathways, 8347 genes in total
Elapsed time is 18.896257 seconds.
Create cell-cell graph for clustering cells ...
Writing graph into file ... 100%Elapsed time is 1.841046 seconds.
Running ModularityOptimizer for clustering ...Elapsed time is 14.358298 seconds.
Cell-cell graph contains 22 cell types by community detection
Elapsed time is 14.694007 seconds.
Cell-cell graph contains 21 cell types after merging tiny cell clusters
creating a total of 20 edges ...     20
Cell-cell graph contains 2 cell types after merging
Number of useful pathways is 1
```

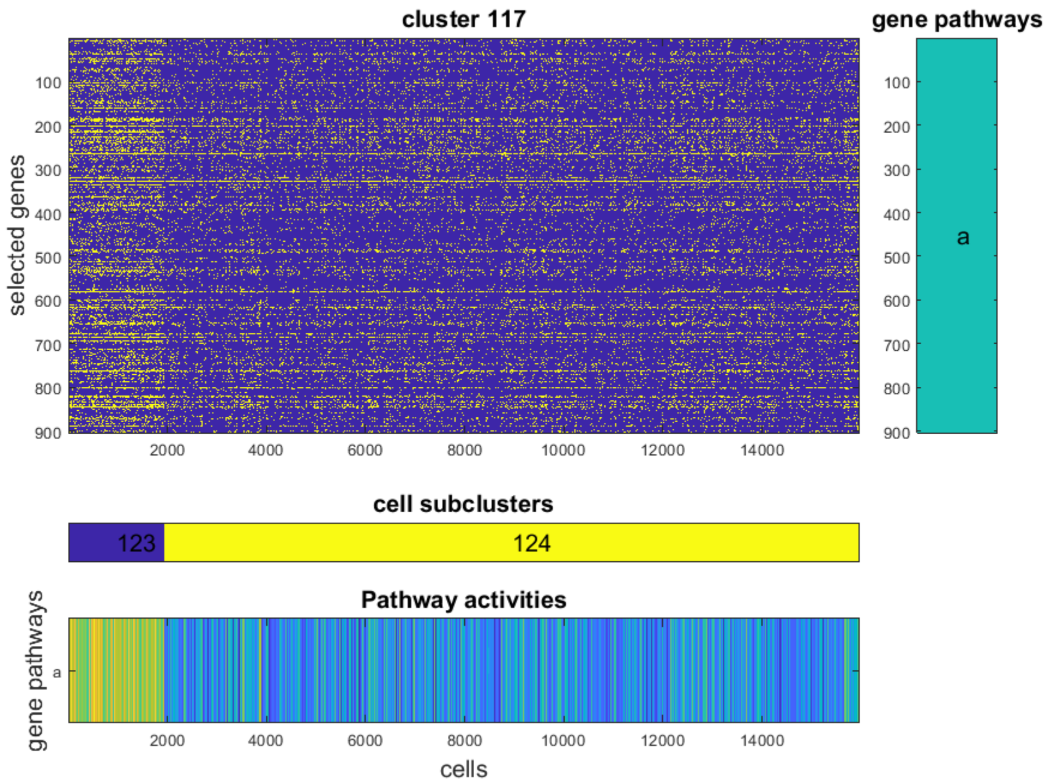


```
Remaining clusters to partition 7
Processing cluster 118 now ...
Processing data subset with 18134 genes and 121 cells:
Remove genes detected in <100 cells. Remaining 0 genes.  Elapsed time is 0.005641 seconds.

Remaining clusters to partition 6
Processing cluster 119 now ...
Processing data subset with 18134 genes and 37 cells:
Remove genes detected in <100 cells. Remaining 0 genes.  Elapsed time is 0.001363 seconds.

Remaining clusters to partition 5
Processing cluster 120 now ...
Processing data subset with 18134 genes and 563 cells:
Remove genes detected in <100 cells. Remaining 422 genes.  Elapsed time is 0.027797 seconds.
Iterate 10 random permutations for gene-gene similarity threshold ...   10 Elapsed time is 0.244043 seconds.
Compute gene-gene similarity ... Elapsed time is 0.009869 seconds.
Create gene-gene graph for clustering genes ...
Writing graph into file ... 100%Elapsed time is 0.009217 seconds.
Running ModularityOptimizer for clustering ...Elapsed time is 0.293524 seconds.
Gene-gene graph contains 4 pathways, 213 genes in total
Elapsed time is 0.305643 seconds.
Create cell-cell graph for clustering cells ...
Writing graph into file ... 100%Elapsed time is 0.061948 seconds.
Running ModularityOptimizer for clustering ...Elapsed time is 0.443368 seconds.
Cell-cell graph contains 8 cell types by community detection
Elapsed time is 0.457213 seconds.
Cell-cell graph contains 7 cell types after merging tiny cell clusters
creating a total of 6 edges ...      6
Cell-cell graph contains 2 cell types after merging
Number of useful pathways is 1
```

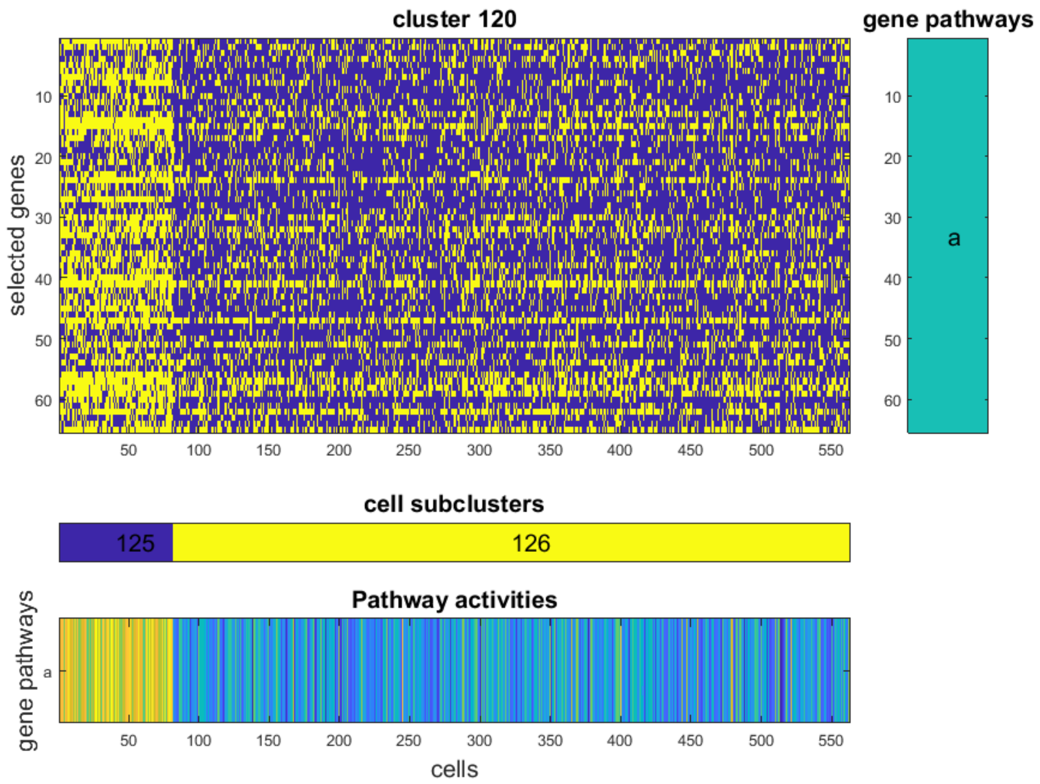


```
Remaining clusters to partition 6
Processing cluster 121 now ...
Processing data subset with 18134 genes and 969 cells:
Remove genes detected in <100 cells. Remaining 4487 genes.  Elapsed time is 0.063486 seconds.
Iterate 10 random permutations for gene-gene similarity threshold ...   10 Elapsed time is 17.905712 seconds.
Compute gene-gene similarity ... Elapsed time is 1.329954 seconds.
Create gene-gene graph for clustering genes ...
Writing graph into file ... 100%Elapsed time is 0.165637 seconds.
Running ModularityOptimizer for clustering ...Elapsed time is 0.570074 seconds.
Gene-gene graph contains 5 pathways, 941 genes in total
Elapsed time is 0.701096 seconds.
Create cell-cell graph for clustering cells ...
Writing graph into file ... 100%Elapsed time is 0.106403 seconds.
Running ModularityOptimizer for clustering ...Elapsed time is 0.689331 seconds.
Cell-cell graph contains 9 cell types by community detection
Elapsed time is 0.713464 seconds.
Cell-cell graph contains 9 cell types after merging tiny cell clusters
creating a total of 8 edges ...      8
Cell-cell graph contains 1 cell types after merging

Remaining clusters to partition 5
Processing cluster 122 now ...
Processing data subset with 18134 genes and 12115 cells:
Remove genes detected in <100 cells. Remaining 11879 genes.  Elapsed time is 1.009915 seconds.
Iterate 10 random permutations for gene-gene similarity threshold ...   10 Elapsed time is 402.335862 seconds.
Compute gene-gene similarity ... Elapsed time is 25.850900 seconds.
Create gene-gene graph for clustering genes ...
Writing graph into file ... 100%Elapsed time is 30.105488 seconds.
Running ModularityOptimizer for clustering ...Elapsed time is 89.269227 seconds.
Gene-gene graph contains 5 pathways, 11261 genes in total
Elapsed time is 89.739798 seconds.
Create cell-cell graph for clustering cells ...
Writing graph into file ... 100%Elapsed time is 1.358940 seconds.
Running ModularityOptimizer for clustering ...Elapsed time is 8.669083 seconds.
Cell-cell graph contains 19 cell types by community detection
Elapsed time is 8.936044 seconds.
Cell-cell graph contains 14 cell types after merging tiny cell clusters
creating a total of 13 edges ...     13
Cell-cell graph contains 2 cell types after merging
Number of useful pathways is 1
```

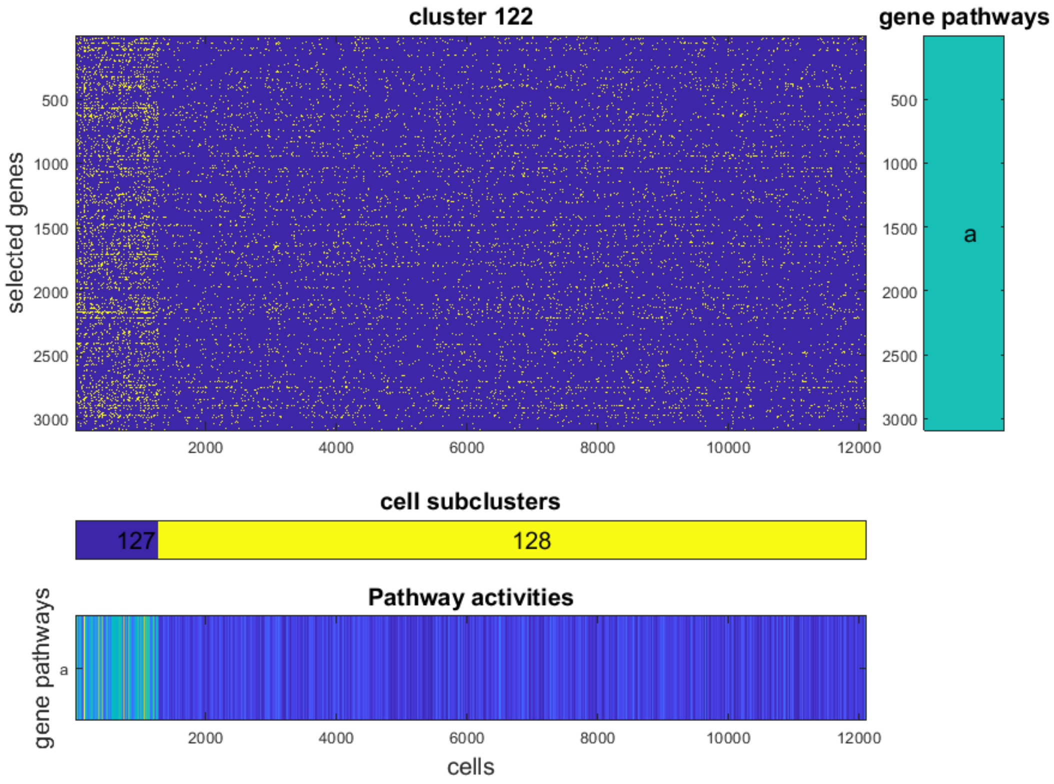


```
Remaining clusters to partition 6
Processing cluster 123 now ...
Processing data subset with 18134 genes and 1929 cells:
Remove genes detected in <100 cells. Remaining 5783 genes.  Elapsed time is 0.144052 seconds.
Iterate 10 random permutations for gene-gene similarity threshold ...   10 Elapsed time is 37.015856 seconds.
Compute gene-gene similarity ... Elapsed time is 2.515217 seconds.
Create gene-gene graph for clustering genes ...
Writing graph into file ... 100%Elapsed time is 0.222783 seconds.
Running ModularityOptimizer for clustering ...Elapsed time is 0.527694 seconds.
Gene-gene graph contains 7 pathways, 772 genes in total
Elapsed time is 0.695065 seconds.
Create cell-cell graph for clustering cells ...
Writing graph into file ... 100%Elapsed time is 0.232154 seconds.
Running ModularityOptimizer for clustering ...Elapsed time is 1.334681 seconds.
Cell-cell graph contains 11 cell types by community detection
Elapsed time is 1.376483 seconds.
Cell-cell graph contains 11 cell types after merging tiny cell clusters
creating a total of 10 edges ...     10
Cell-cell graph contains 2 cell types after merging
Number of useful pathways is 1
```

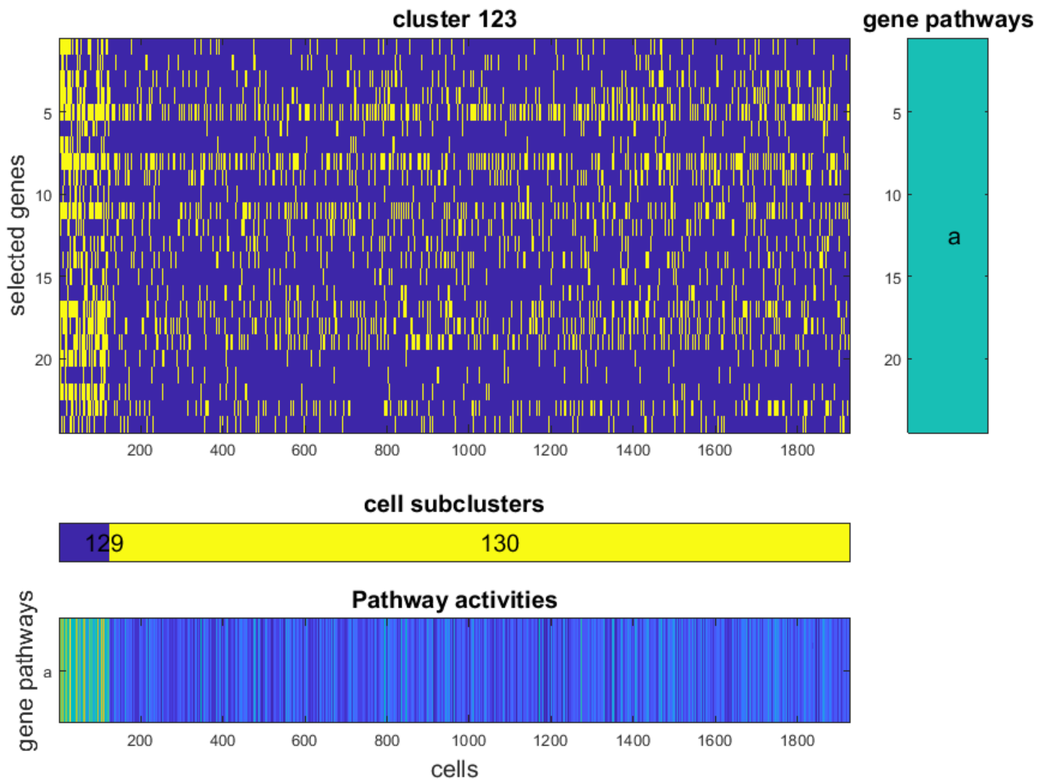


```
Remaining clusters to partition 7
Processing cluster 124 now ...
Processing data subset with 18134 genes and 14040 cells:
Remove genes detected in <100 cells. Remaining 10262 genes.  Elapsed time is 1.108090 seconds.
Iterate 10 random permutations for gene-gene similarity threshold ...   10 Elapsed time is 360.374761 seconds.
Compute gene-gene similarity ... Elapsed time is 22.194016 seconds.
Create gene-gene graph for clustering genes ...
Writing graph into file ... 100%Elapsed time is 8.226159 seconds.
Running ModularityOptimizer for clustering ...Elapsed time is 19.054350 seconds.
Gene-gene graph contains 6 pathways, 8275 genes in total
Elapsed time is 19.420317 seconds.
Create cell-cell graph for clustering cells ...
Writing graph into file ... 100%Elapsed time is 1.655645 seconds.
Running ModularityOptimizer for clustering ...Elapsed time is 12.816048 seconds.
Cell-cell graph contains 15 cell types by community detection
Elapsed time is 13.088766 seconds.
Cell-cell graph contains 13 cell types after merging tiny cell clusters
Cell-cell graph contains 3 cell types after merging
Number of useful pathways is 2
```

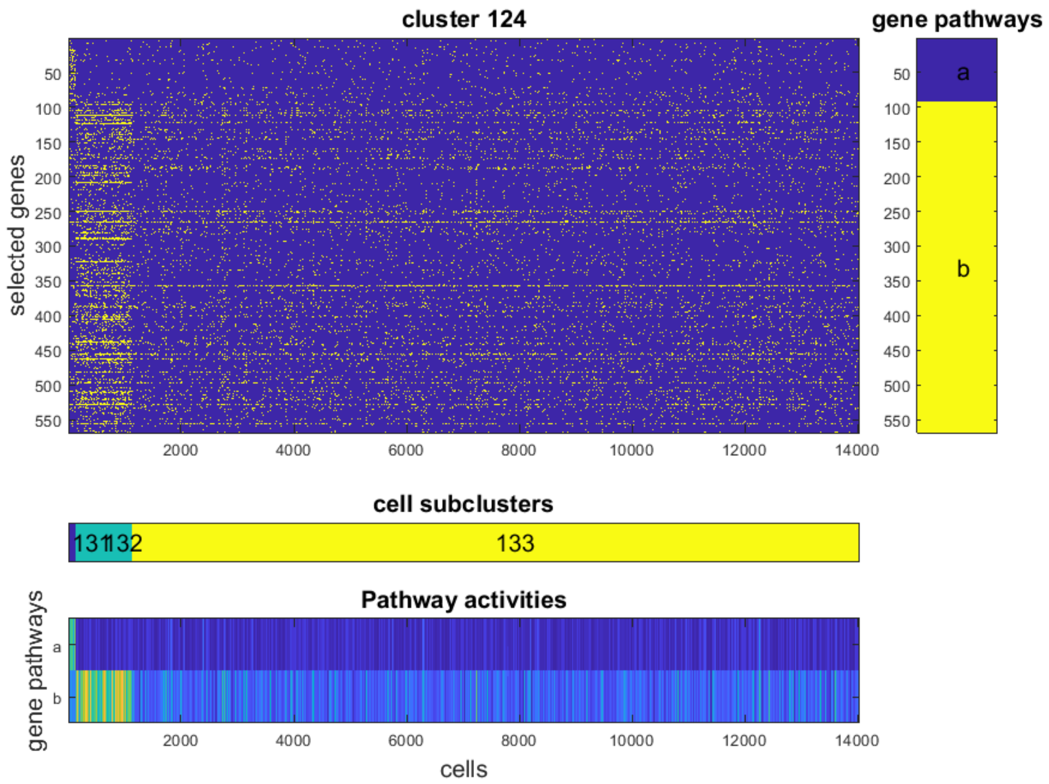


```
Remaining clusters to partition 9
Processing cluster 125 now ...
Processing data subset with 18134 genes and 81 cells:
Remove genes detected in <100 cells. Remaining 0 genes.  Elapsed time is 0.004343 seconds.

Remaining clusters to partition 8
Processing cluster 126 now ...
Processing data subset with 18134 genes and 482 cells:
Remove genes detected in <100 cells. Remaining 324 genes.  Elapsed time is 0.023720 seconds.
Iterate 10 random permutations for gene-gene similarity threshold ...   10 Elapsed time is 0.131765 seconds.
Compute gene-gene similarity ... Elapsed time is 0.003899 seconds.
Create gene-gene graph for clustering genes ...
Writing graph into file ... 100%Elapsed time is 0.004610 seconds.
Running ModularityOptimizer for clustering ...Elapsed time is 0.242198 seconds.
Gene-gene graph contains 4 pathways, 110 genes in total
Elapsed time is 0.251969 seconds.
Create cell-cell graph for clustering cells ...
Writing graph into file ... 100%Elapsed time is 0.054266 seconds.
Running ModularityOptimizer for clustering ...Elapsed time is 0.454652 seconds.
Cell-cell graph contains 7 cell types by community detection
Elapsed time is 0.467075 seconds.
Cell-cell graph contains 6 cell types after merging tiny cell clusters
creating a total of 5 edges ...      5
Cell-cell graph contains 2 cell types after merging
Number of useful pathways is 1
```

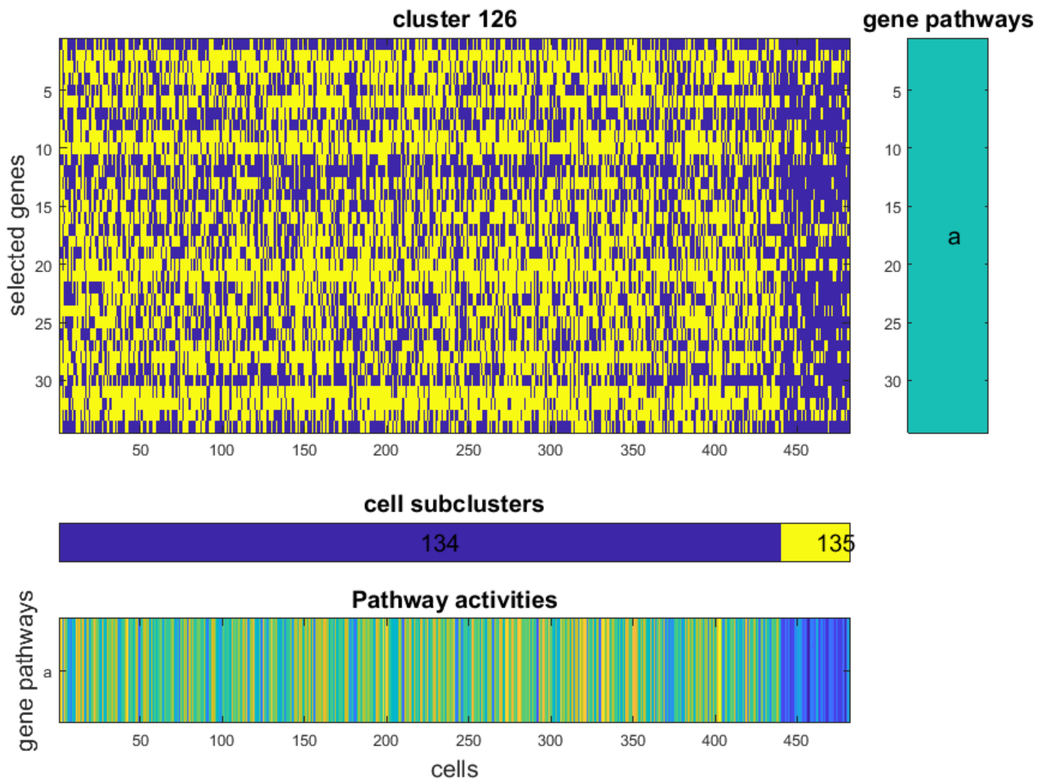


```
Remaining clusters to partition 9
Processing cluster 127 now ...
Processing data subset with 18134 genes and 1261 cells:
Remove genes detected in <100 cells. Remaining 7530 genes.  Elapsed time is 0.089401 seconds.
Iterate 10 random permutations for gene-gene similarity threshold ...   10 Elapsed time is 50.658287 seconds.
Compute gene-gene similarity ... Elapsed time is 3.738236 seconds.
Create gene-gene graph for clustering genes ...
Writing graph into file ... 100%Elapsed time is 3.770108 seconds.
Running ModularityOptimizer for clustering ...Elapsed time is 14.029144 seconds.
Gene-gene graph contains 3 pathways, 5992 genes in total
Elapsed time is 14.267262 seconds.
Create cell-cell graph for clustering cells ...
Writing graph into file ... 100%Elapsed time is 0.137232 seconds.
Running ModularityOptimizer for clustering ...Elapsed time is 0.689805 seconds.
Cell-cell graph contains 13 cell types by community detection
Elapsed time is 0.717594 seconds.
Cell-cell graph contains 12 cell types after merging tiny cell clusters
creating a total of 11 edges ...     11
Cell-cell graph contains 2 cell types after merging
Number of useful pathways is 1
```

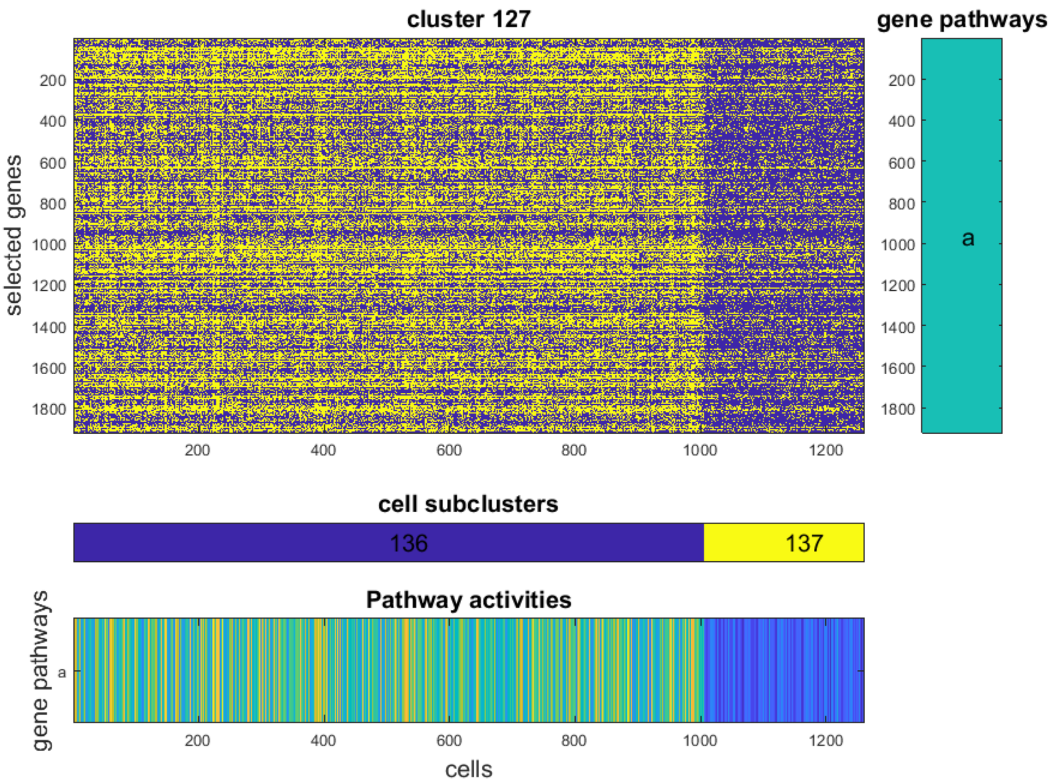


```
Remaining clusters to partition 10
Processing cluster 128 now ...
Processing data subset with 18134 genes and 10854 cells:
Remove genes detected in <100 cells. Remaining 11030 genes.  Elapsed time is 0.867825 seconds.
Iterate 10 random permutations for gene-gene similarity threshold ...   10 Elapsed time is 324.936045 seconds.
Compute gene-gene similarity ... Elapsed time is 21.268414 seconds.
Create gene-gene graph for clustering genes ...
Writing graph into file ... 100%Elapsed time is 7.975663 seconds.
Running ModularityOptimizer for clustering ...Elapsed time is 27.049345 seconds.
Gene-gene graph contains 6 pathways, 8857 genes in total
Elapsed time is 27.463063 seconds.
Create cell-cell graph for clustering cells ...
Writing graph into file ... 100%Elapsed time is 1.189074 seconds.
Running ModularityOptimizer for clustering ...Elapsed time is 5.603694 seconds.
Cell-cell graph contains 29 cell types by community detection
Elapsed time is 5.841671 seconds.
Cell-cell graph contains 24 cell types after merging tiny cell clusters
creating a total of 23 edges ...     23
Cell-cell graph contains 2 cell types after merging
Number of useful pathways is 1
```

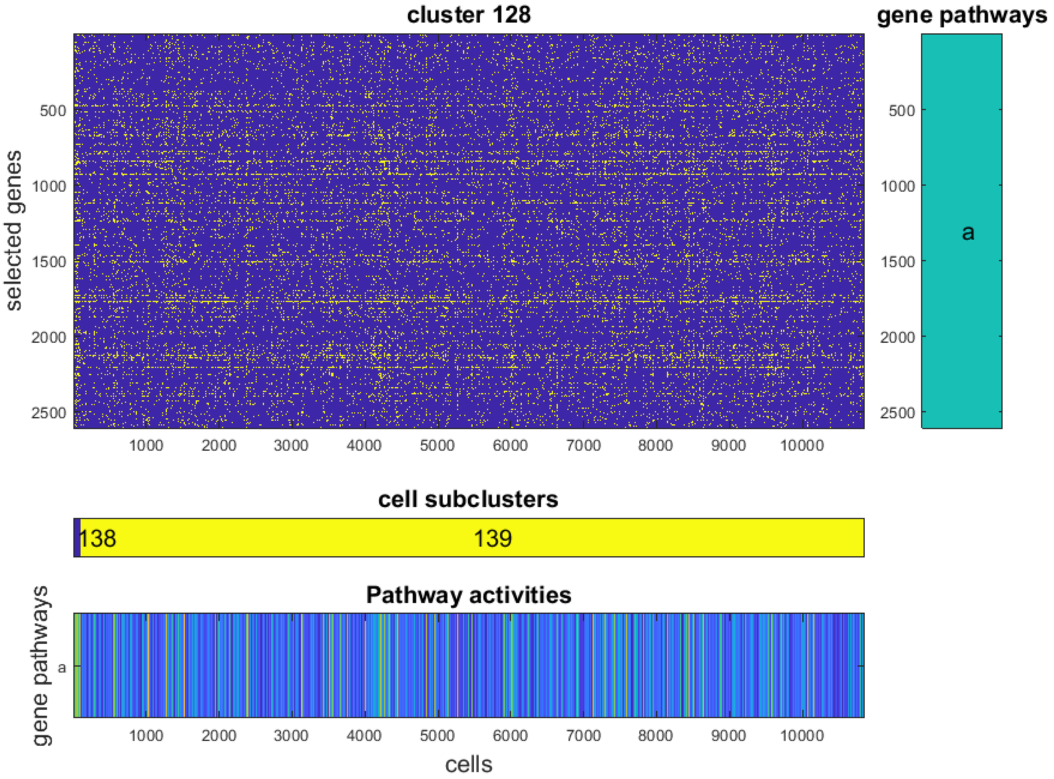


```
Remaining clusters to partition 11
Processing cluster 129 now ...
Processing data subset with 18134 genes and 124 cells:
Remove genes detected in <100 cells. Remaining 0 genes.  Elapsed time is 0.005829 seconds.

Remaining clusters to partition 10
Processing cluster 130 now ...
Processing data subset with 18134 genes and 1805 cells:
Remove genes detected in <100 cells. Remaining 5578 genes.  Elapsed time is 0.117329 seconds.
Iterate 10 random permutations for gene-gene similarity threshold ...   10 Elapsed time is 33.162871 seconds.
Compute gene-gene similarity ... Elapsed time is 2.326893 seconds.
Create gene-gene graph for clustering genes ...
Writing graph into file ... 100%Elapsed time is 0.201051 seconds.
Running ModularityOptimizer for clustering ...Elapsed time is 0.502740 seconds.
Gene-gene graph contains 5 pathways, 713 genes in total
Elapsed time is 0.659470 seconds.
Create cell-cell graph for clustering cells ...
Writing graph into file ... 100%Elapsed time is 0.207298 seconds.
Running ModularityOptimizer for clustering ...Elapsed time is 1.222668 seconds.
Cell-cell graph contains 11 cell types by community detection
Elapsed time is 1.262277 seconds.
Cell-cell graph contains 11 cell types after merging tiny cell clusters
creating a total of 10 edges ...     10
Cell-cell graph contains 1 cell types after merging

Remaining clusters to partition 9
Processing cluster 131 now ...
Processing data subset with 18134 genes and 136 cells:
Remove genes detected in <100 cells. Remaining 0 genes.  Elapsed time is 0.007515 seconds.

Remaining clusters to partition 8
Processing cluster 132 now ...
Processing data subset with 18134 genes and 977 cells:
Remove genes detected in <100 cells. Remaining 3036 genes.  Elapsed time is 0.060593 seconds.
Iterate 10 random permutations for gene-gene similarity threshold ...   10 Elapsed time is 8.886584 seconds.
Compute gene-gene similarity ... Elapsed time is 0.587403 seconds.
Create gene-gene graph for clustering genes ...
Writing graph into file ... 100%Elapsed time is 0.066972 seconds.
Running ModularityOptimizer for clustering ...Elapsed time is 0.427711 seconds.
Gene-gene graph contains 8 pathways, 534 genes in total
Elapsed time is 0.506624 seconds.
Create cell-cell graph for clustering cells ...
Writing graph into file ... 100%Elapsed time is 0.112273 seconds.
Running ModularityOptimizer for clustering ...Elapsed time is 0.767545 seconds.
Cell-cell graph contains 9 cell types by community detection
Elapsed time is 0.789618 seconds.
Cell-cell graph contains 9 cell types after merging tiny cell clusters
creating a total of 8 edges ...     8
Cell-cell graph contains 2 cell types after merging
Number of useful pathways is 2
```

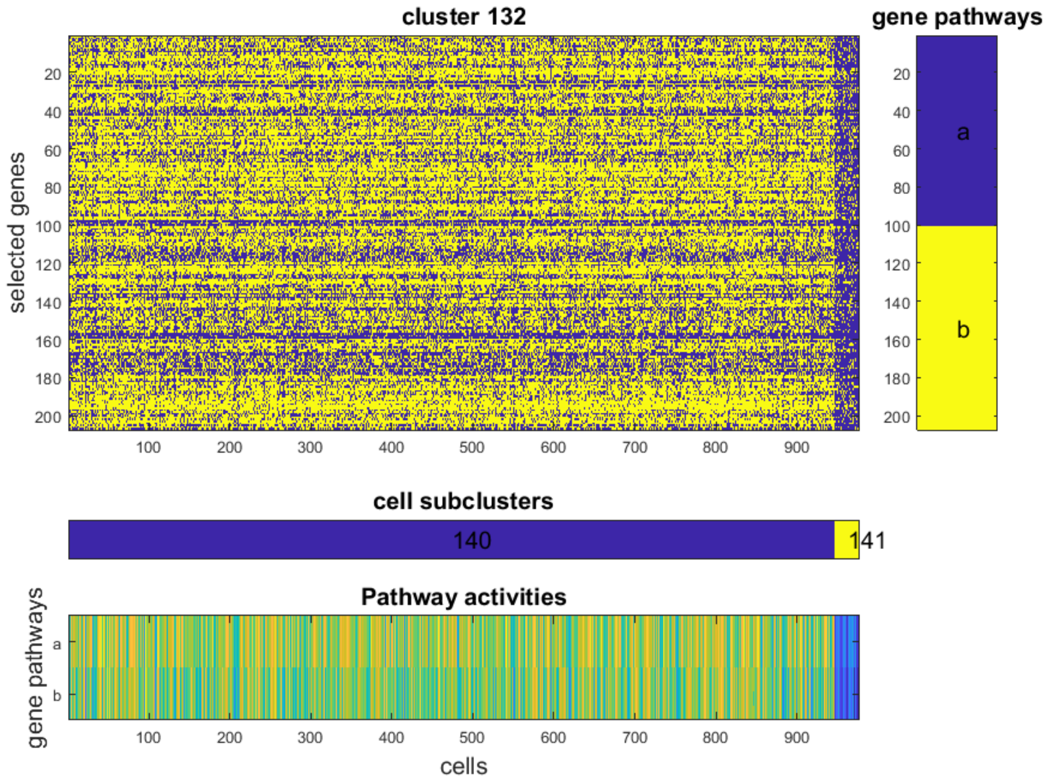


```
Remaining clusters to partition 9
Processing cluster 133 now ...
Processing data subset with 18134 genes and 12927 cells:
Remove genes detected in <100 cells. Remaining 10050 genes.  Elapsed time is 1.033634 seconds.
Iterate 10 random permutations for gene-gene similarity threshold ...   10 Elapsed time is 322.522389 seconds.
Compute gene-gene similarity ... Elapsed time is 19.753496 seconds.
Create gene-gene graph for clustering genes ...
Writing graph into file ... 100%Elapsed time is 8.039622 seconds.
Running ModularityOptimizer for clustering ...Elapsed time is 17.967275 seconds.
Gene-gene graph contains 4 pathways, 7998 genes in total
Elapsed time is 18.335983 seconds.
Create cell-cell graph for clustering cells ...
Writing graph into file ... 100%Elapsed time is 1.463962 seconds.
Running ModularityOptimizer for clustering ...Elapsed time is 9.205633 seconds.
Cell-cell graph contains 15 cell types by community detection
Elapsed time is 9.485721 seconds.
Cell-cell graph contains 12 cell types after merging tiny cell clusters
creating a total of 11 edges ...     11
Cell-cell graph contains 2 cell types after merging
Number of useful pathways is 1
```

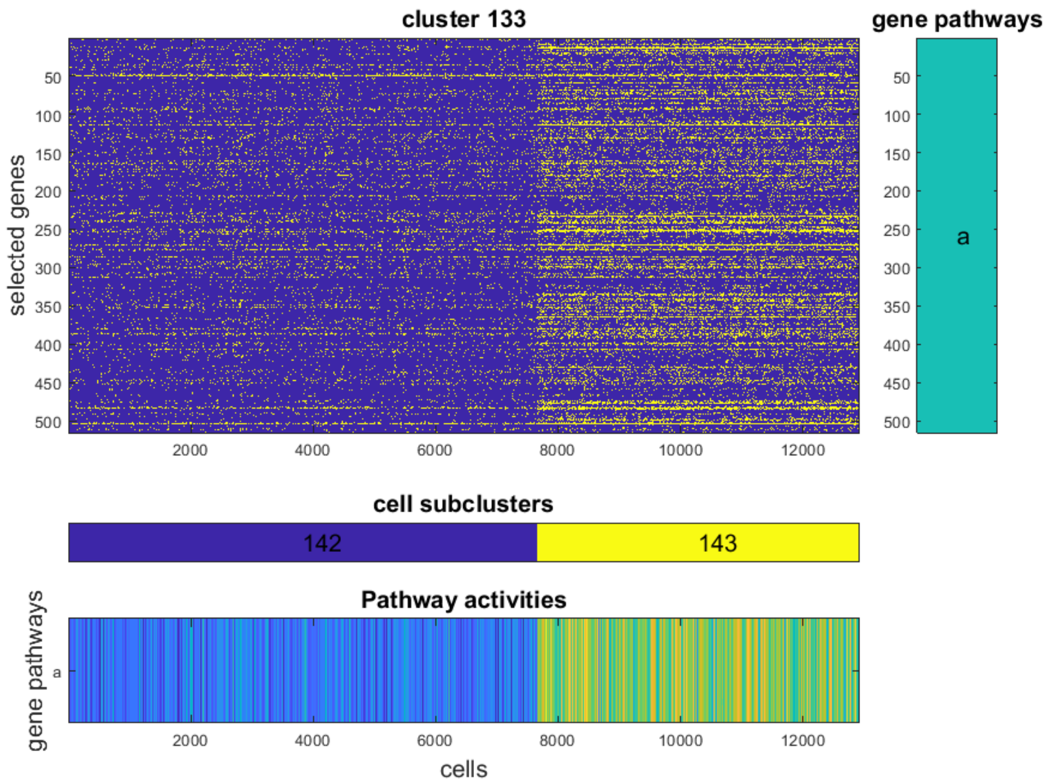


```
Remaining clusters to partition 10
Processing cluster 134 now ...
Processing data subset with 18134 genes and 440 cells:
Remove genes detected in <100 cells. Remaining 298 genes.  Elapsed time is 0.023265 seconds.
Iterate 10 random permutations for gene-gene similarity threshold ...   10 Elapsed time is 0.118463 seconds.
Compute gene-gene similarity ... Elapsed time is 0.003426 seconds.
Create gene-gene graph for clustering genes ...
Writing graph into file ... 101%Elapsed time is 0.002638 seconds.
Running ModularityOptimizer for clustering ...Elapsed time is 0.203720 seconds.
Gene-gene graph contains 1 pathways, 20 genes in total
Elapsed time is 0.213219 seconds.
Create cell-cell graph for clustering cells ...
Writing graph into file ... 100%Elapsed time is 0.048518 seconds.
Running ModularityOptimizer for clustering ...Elapsed time is 0.394372 seconds.
Cell-cell graph contains 9 cell types by community detection
Elapsed time is 0.405854 seconds.
Cell-cell graph contains 2 cell types after merging tiny cell clusters
creating a total of 1 edges ...      1
Cell-cell graph contains 1 cell types after merging

Remaining clusters to partition 9
Processing cluster 135 now ...
Processing data subset with 18134 genes and 42 cells:
Remove genes detected in <100 cells. Remaining 0 genes.  Elapsed time is 0.001627 seconds.

Remaining clusters to partition 8
Processing cluster 136 now ...
Processing data subset with 18134 genes and 1005 cells:
Remove genes detected in <100 cells. Remaining 6902 genes.  Elapsed time is 0.073390 seconds.
Iterate 10 random permutations for gene-gene similarity threshold ...   10 Elapsed time is 40.299549 seconds.
Compute gene-gene similarity ... Elapsed time is 3.032444 seconds.
Create gene-gene graph for clustering genes ...
Writing graph into file ... 100%Elapsed time is 1.821796 seconds.
Running ModularityOptimizer for clustering ...Elapsed time is 4.611453 seconds.
Gene-gene graph contains 4 pathways, 4653 genes in total
Elapsed time is 4.826414 seconds.
Create cell-cell graph for clustering cells ...
Writing graph into file ... 100%Elapsed time is 0.112557 seconds.
Running ModularityOptimizer for clustering ...Elapsed time is 0.581323 seconds.
Cell-cell graph contains 11 cell types by community detection
Elapsed time is 0.603991 seconds.
Cell-cell graph contains 11 cell types after merging tiny cell clusters
creating a total of 10 edges ...     10
Cell-cell graph contains 2 cell types after merging
Number of useful pathways is 1
```

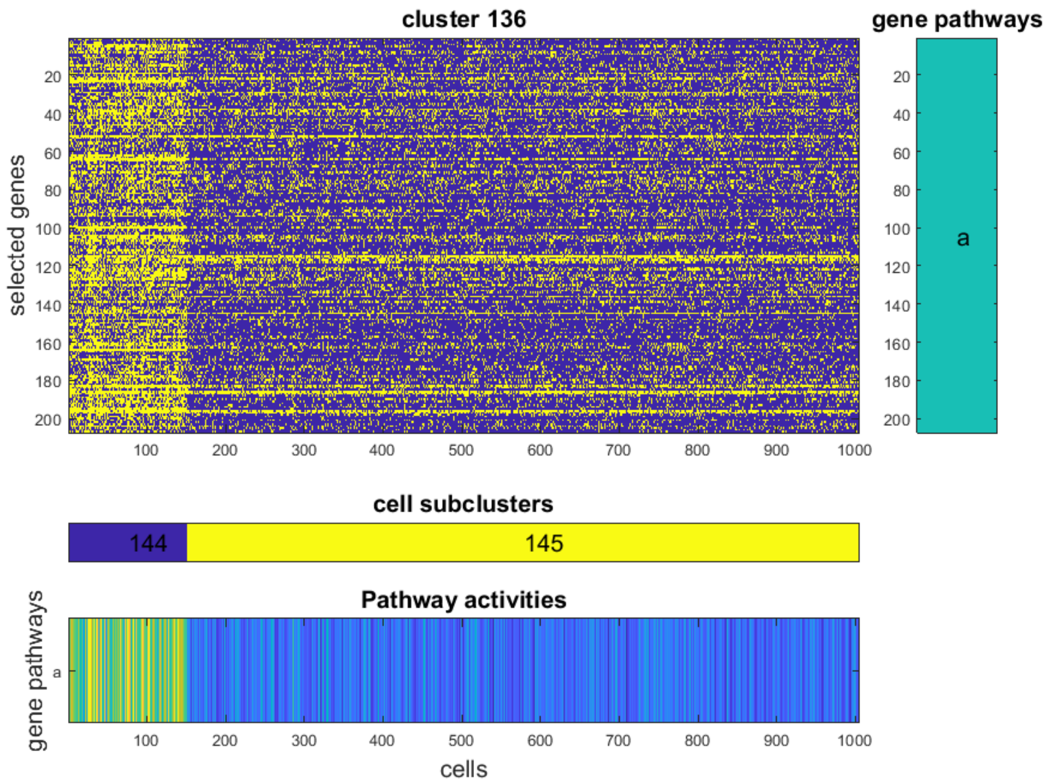


```
Remaining clusters to partition 9
Processing cluster 137 now ...
Processing data subset with 18134 genes and 256 cells:
Remove genes detected in <100 cells. Remaining 487 genes.  Elapsed time is 0.012339 seconds.
Iterate 10 random permutations for gene-gene similarity threshold ...   10 Elapsed time is 0.191916 seconds.
Compute gene-gene similarity ... Elapsed time is 0.013888 seconds.
Create gene-gene graph for clustering genes ...
Writing graph into file ... 100%Elapsed time is 0.007170 seconds.
Running ModularityOptimizer for clustering ...Elapsed time is 0.271196 seconds.
Gene-gene graph contains 4 pathways, 169 genes in total
Elapsed time is 0.285081 seconds.
Create cell-cell graph for clustering cells ...
Writing graph into file ... 100%Elapsed time is 0.028686 seconds.
Running ModularityOptimizer for clustering ...Elapsed time is 0.292631 seconds.
Cell-cell graph contains 6 cell types by community detection
Elapsed time is 0.300892 seconds.
Cell-cell graph contains 6 cell types after merging tiny cell clusters
Cell-cell graph contains 3 cell types after merging
Number of useful pathways is 2
```

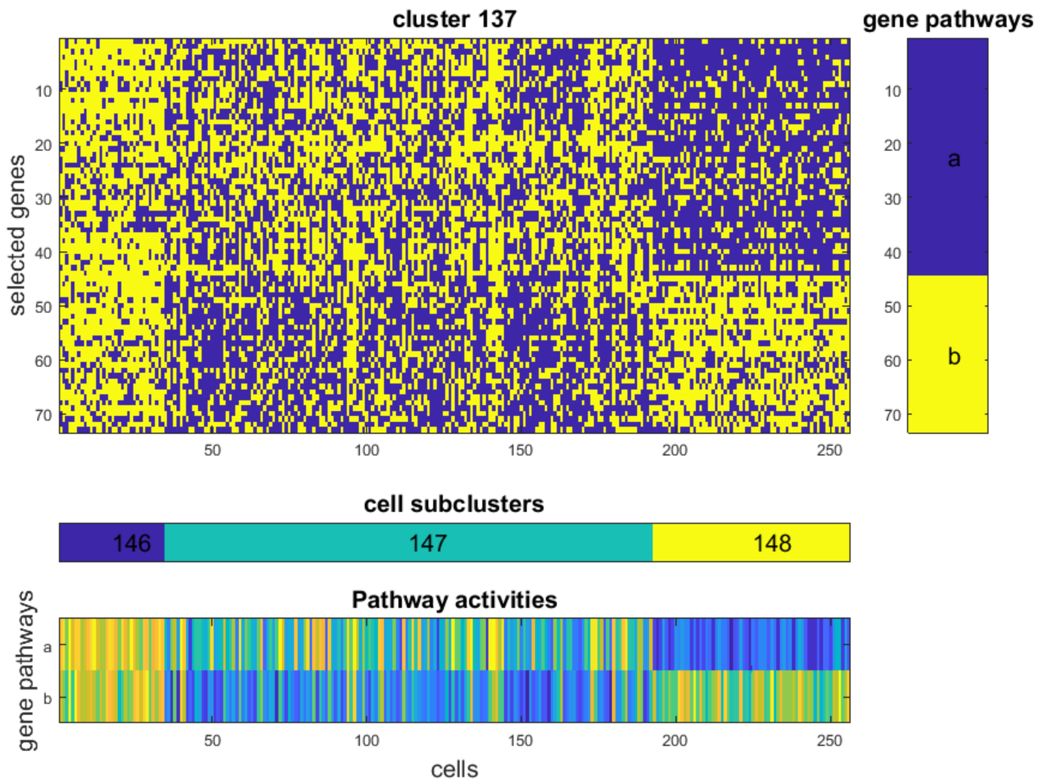


```
Remaining clusters to partition 11
Processing cluster 138 now ...
Processing data subset with 18134 genes and 89 cells:
Remove genes detected in <100 cells. Remaining 0 genes.  Elapsed time is 0.004686 seconds.

Remaining clusters to partition 10
Processing cluster 139 now ...
Processing data subset with 18134 genes and 10765 cells:
Remove genes detected in <100 cells. Remaining 10998 genes.  Elapsed time is 0.853764 seconds.
Iterate 10 random permutations for gene-gene similarity threshold ...   10 Elapsed time is 319.673733 seconds.
Compute gene-gene similarity ... Elapsed time is 21.011782 seconds.
Create gene-gene graph for clustering genes ...
Writing graph into file ... 100%Elapsed time is 8.234430 seconds.
Running ModularityOptimizer for clustering ...Elapsed time is 26.494098 seconds.
Gene-gene graph contains 6 pathways, 8971 genes in total
Elapsed time is 26.891545 seconds.
Create cell-cell graph for clustering cells ...
Writing graph into file ... 100%Elapsed time is 1.178960 seconds.
Running ModularityOptimizer for clustering ...Elapsed time is 5.928985 seconds.
Cell-cell graph contains 28 cell types by community detection
Elapsed time is 6.145676 seconds.
Cell-cell graph contains 20 cell types after merging tiny cell clusters
creating a total of 19 edges ...     19
Cell-cell graph contains 2 cell types after merging
Number of useful pathways is 1
```

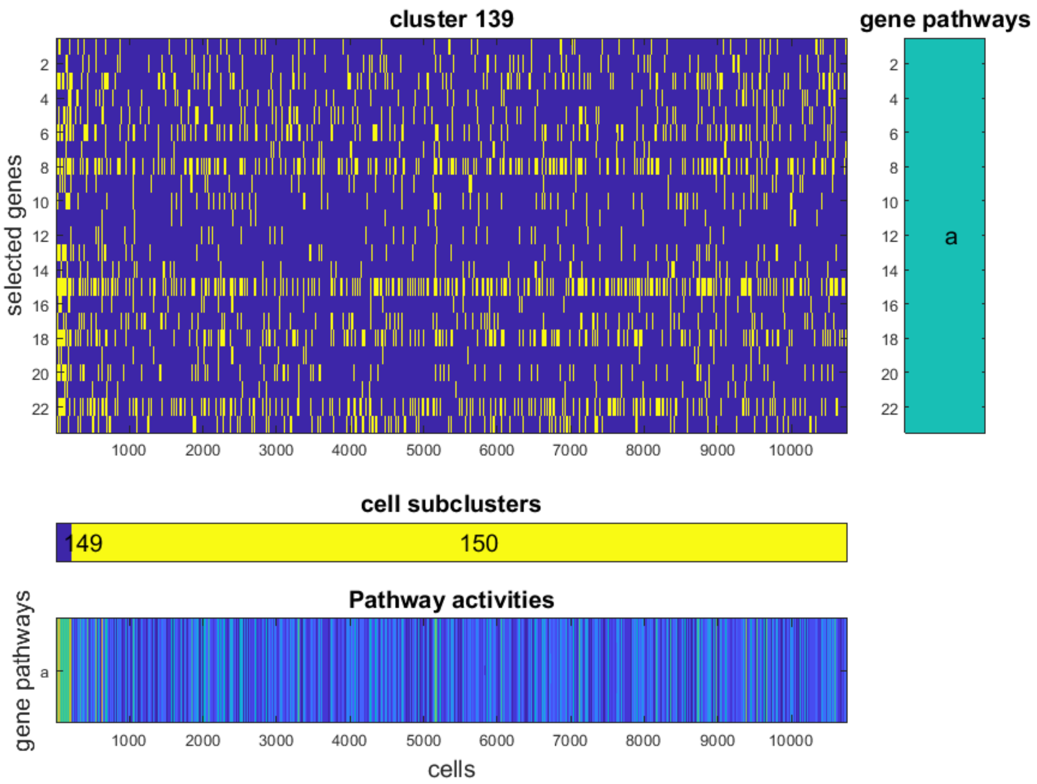


```
Remaining clusters to partition 11
Processing cluster 140 now ...
Processing data subset with 18134 genes and 946 cells:
Remove genes detected in <100 cells. Remaining 3000 genes.  Elapsed time is 0.058056 seconds.
Iterate 10 random permutations for gene-gene similarity threshold ...   10 Elapsed time is 9.106246 seconds.
Compute gene-gene similarity ... Elapsed time is 0.645225 seconds.
Create gene-gene graph for clustering genes ...
Writing graph into file ... 100%Elapsed time is 0.065510 seconds.
Running ModularityOptimizer for clustering ...Elapsed time is 0.442197 seconds.
Gene-gene graph contains 5 pathways, 245 genes in total
Elapsed time is 0.519681 seconds.
Create cell-cell graph for clustering cells ...
Writing graph into file ... 100%Elapsed time is 0.109160 seconds.
Running ModularityOptimizer for clustering ...Elapsed time is 0.779664 seconds.
Cell-cell graph contains 9 cell types by community detection
Elapsed time is 0.801166 seconds.
Cell-cell graph contains 9 cell types after merging tiny cell clusters
creating a total of 8 edges ...      8
Cell-cell graph contains 1 cell types after merging

Remaining clusters to partition 10
Processing cluster 141 now ...
Processing data subset with 18134 genes and 31 cells:
Remove genes detected in <100 cells. Remaining 0 genes.  Elapsed time is 0.001866 seconds.

Remaining clusters to partition 9
Processing cluster 142 now ...
Processing data subset with 18134 genes and 7648 cells:
Remove genes detected in <100 cells. Remaining 9315 genes.  Elapsed time is 0.623208 seconds.
Iterate 10 random permutations for gene-gene similarity threshold ...   10 Elapsed time is 184.975545 seconds.
Compute gene-gene similarity ... Elapsed time is 12.028878 seconds.
Create gene-gene graph for clustering genes ...
Writing graph into file ... 100%Elapsed time is 7.390442 seconds.
Running ModularityOptimizer for clustering ...Elapsed time is 18.617804 seconds.
Gene-gene graph contains 4 pathways, 7708 genes in total
Elapsed time is 18.931818 seconds.
Create cell-cell graph for clustering cells ...
Writing graph into file ... 100%Elapsed time is 0.852174 seconds.
Running ModularityOptimizer for clustering ...Elapsed time is 4.407404 seconds.
Cell-cell graph contains 20 cell types by community detection
Elapsed time is 4.568781 seconds.
Cell-cell graph contains 14 cell types after merging tiny cell clusters
creating a total of 13 edges ...     13
Cell-cell graph contains 2 cell types after merging
Number of useful pathways is 1
```

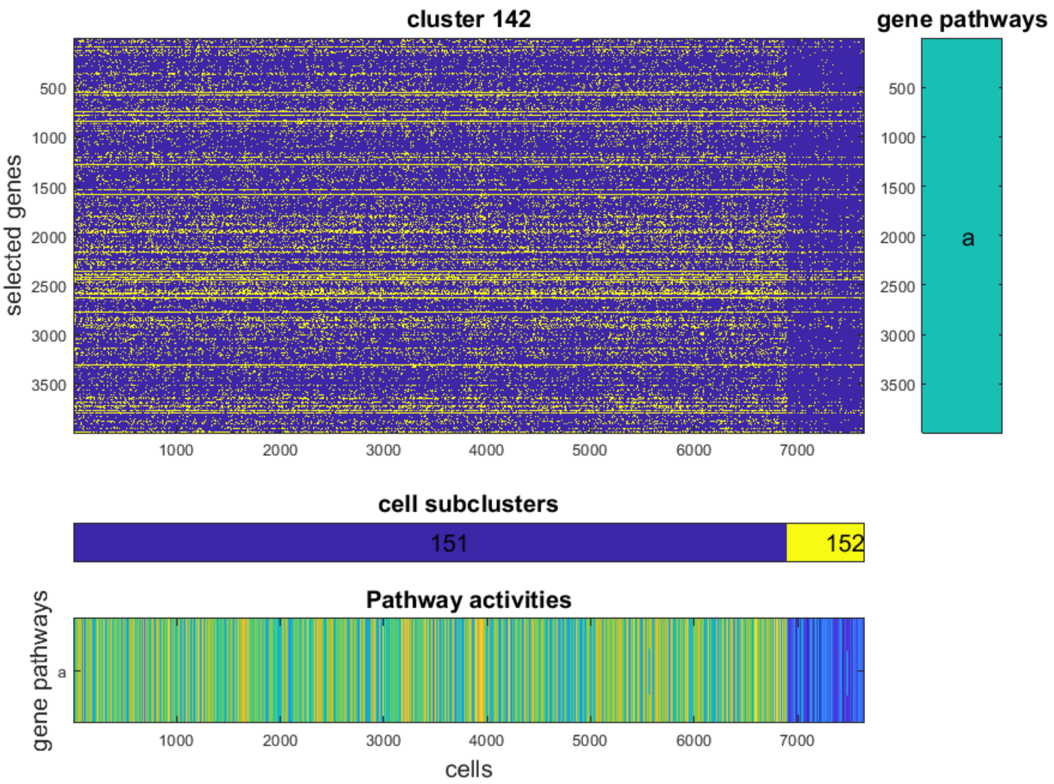


```
Remaining clusters to partition 10
Processing cluster 143 now ...
Processing data subset with 18134 genes and 5279 cells:
Remove genes detected in <100 cells. Remaining 7313 genes.  Elapsed time is 0.376191 seconds.
Iterate 10 random permutations for gene-gene similarity threshold ...   10 Elapsed time is 93.084626 seconds.
Compute gene-gene similarity ... Elapsed time is 6.142255 seconds.
Create gene-gene graph for clustering genes ...
Writing graph into file ... 100%Elapsed time is 0.468509 seconds.
Running ModularityOptimizer for clustering ...Elapsed time is 1.115324 seconds.
Gene-gene graph contains 7 pathways, 1892 genes in total
Elapsed time is 1.339734 seconds.
Create cell-cell graph for clustering cells ...
Writing graph into file ... 100%Elapsed time is 0.628315 seconds.
Running ModularityOptimizer for clustering ...Elapsed time is 4.172369 seconds.
Cell-cell graph contains 15 cell types by community detection
Elapsed time is 4.286683 seconds.
Cell-cell graph contains 14 cell types after merging tiny cell clusters
Cell-cell graph contains 2 cell types after merging
Number of useful pathways is 1
```

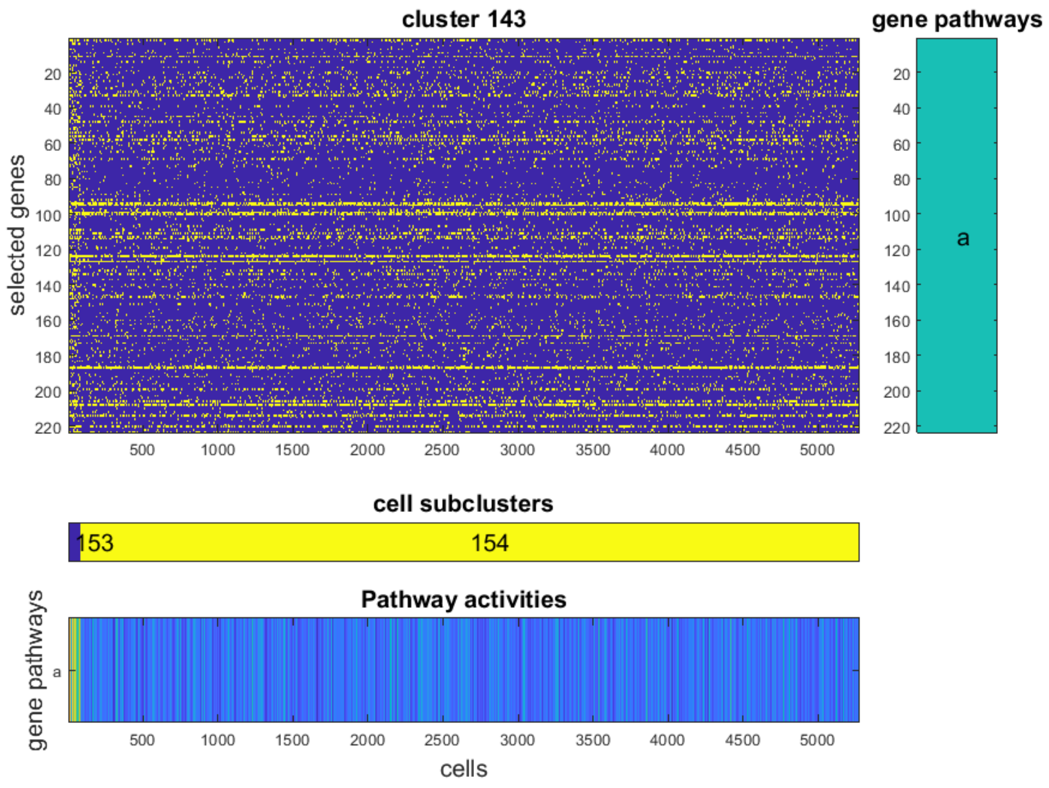


```
Remaining clusters to partition 11
Processing cluster 144 now ...
Processing data subset with 18134 genes and 151 cells:
Remove genes detected in <100 cells. Remaining 0 genes.  Elapsed time is 0.008448 seconds.

Remaining clusters to partition 10
Processing cluster 145 now ...
Processing data subset with 18134 genes and 854 cells:
Remove genes detected in <100 cells. Remaining 6350 genes.  Elapsed time is 0.060541 seconds.
Iterate 10 random permutations for gene-gene similarity threshold ...   10 Elapsed time is 32.967135 seconds.
Compute gene-gene similarity ... Elapsed time is 2.523511 seconds.
Create gene-gene graph for clustering genes ...
Writing graph into file ... 100%Elapsed time is 1.900427 seconds.
Running ModularityOptimizer for clustering ...Elapsed time is 4.724309 seconds.
Gene-gene graph contains 3 pathways, 4274 genes in total
Elapsed time is 4.912704 seconds.
Create cell-cell graph for clustering cells ...
Writing graph into file ... 100%Elapsed time is 0.090629 seconds.
Running ModularityOptimizer for clustering ...Elapsed time is 0.473255 seconds.
Cell-cell graph contains 10 cell types by community detection
Elapsed time is 0.493465 seconds.
Cell-cell graph contains 10 cell types after merging tiny cell clusters
creating a total of 9 edges ...      9
Cell-cell graph contains 1 cell types after merging

Remaining clusters to partition 9
Processing cluster 146 now ...
Processing data subset with 18134 genes and 34 cells:
Remove genes detected in <100 cells. Remaining 0 genes.  Elapsed time is 0.001610 seconds.

Remaining clusters to partition 8
Processing cluster 147 now ...
Processing data subset with 18134 genes and 158 cells:
Remove genes detected in <100 cells. Remaining 0 genes.  Elapsed time is 0.007665 seconds.

Remaining clusters to partition 7
Processing cluster 148 now ...
Processing data subset with 18134 genes and 64 cells:
Remove genes detected in <100 cells. Remaining 0 genes.  Elapsed time is 0.003323 seconds.

Remaining clusters to partition 6
Processing cluster 149 now ...
Processing data subset with 18134 genes and 205 cells:
Remove genes detected in <100 cells. Remaining 86 genes.  Elapsed time is 0.010366 seconds.
Iterate 10 random permutations for gene-gene similarity threshold ...   10 Elapsed time is 0.018025 seconds.
Compute gene-gene similarity ... Elapsed time is 0.000845 seconds.
Create gene-gene graph for clustering genes ...
Writing graph into file ... 105%Elapsed time is 0.001671 seconds.
Running ModularityOptimizer for clustering ...Elapsed time is 0.170656 seconds.
Gene-gene graph contains 0 pathways, 0 genes in total
Elapsed time is 0.175536 seconds.

Remaining clusters to partition 5
Processing cluster 150 now ...
Processing data subset with 18134 genes and 10560 cells:
Remove genes detected in <100 cells. Remaining 10934 genes.  Elapsed time is 0.859697 seconds.
```

```
Iterate 10 random permutations for gene-gene similarity threshold ...   10 Elapsed time is 318.266109 seconds.
Compute gene-gene similarity ... Elapsed time is 20.784080 seconds.
Create gene-gene graph for clustering genes ...
Writing graph into file ... 100%Elapsed time is 7.742363 seconds.
Running ModularityOptimizer for clustering ...Elapsed time is 24.851279 seconds.
Gene-gene graph contains 5 pathways, 8780 genes in total
Elapsed time is 25.246429 seconds.
Create cell-cell graph for clustering cells ...
Writing graph into file ... 100%Elapsed time is 1.195140 seconds.
Running ModularityOptimizer for clustering ...Elapsed time is 6.583968 seconds.
Cell-cell graph contains 19 cell types by community detection
Elapsed time is 6.794240 seconds.
Cell-cell graph contains 15 cell types after merging tiny cell clusters
creating a total of 14 edges ...     14
Cell-cell graph contains 2 cell types after merging
Number of useful pathways is 1
```

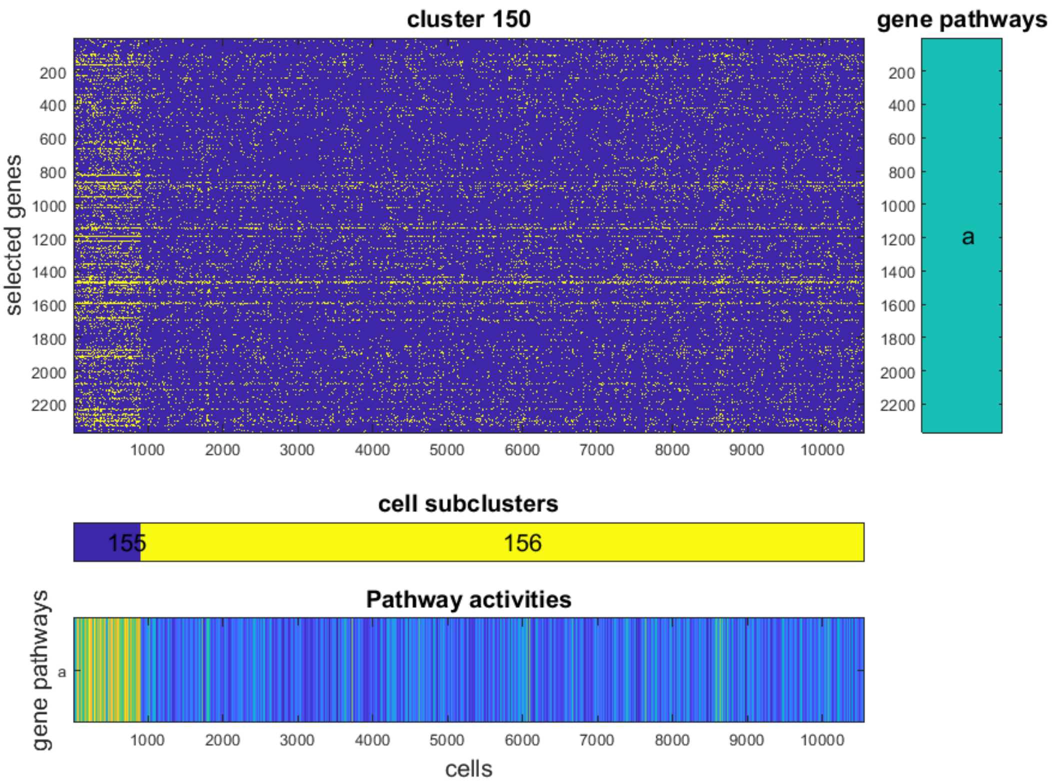


```
Remaining clusters to partition 6
Processing cluster 151 now ...
Processing data subset with 18134 genes and 6894 cells:
Remove genes detected in <100 cells. Remaining 9175 genes.  Elapsed time is 0.537645 seconds.
Iterate 10 random permutations for gene-gene similarity threshold ...   10 Elapsed time is 168.934730 seconds.
Compute gene-gene similarity ... Elapsed time is 11.225783 seconds.
Create gene-gene graph for clustering genes ...
Writing graph into file ... 100%Elapsed time is 4.829962 seconds.
Running ModularityOptimizer for clustering ...Elapsed time is 9.658356 seconds.
Gene-gene graph contains 3 pathways, 6766 genes in total
Elapsed time is 9.979930 seconds.
Create cell-cell graph for clustering cells ...
Writing graph into file ... 100%Elapsed time is 0.735073 seconds.
Running ModularityOptimizer for clustering ...Elapsed time is 3.520021 seconds.
Cell-cell graph contains 20 cell types by community detection
Elapsed time is 3.661937 seconds.
Cell-cell graph contains 17 cell types after merging tiny cell clusters
creating a total of 16 edges ...     16
Cell-cell graph contains 2 cell types after merging
Number of useful pathways is 1
```

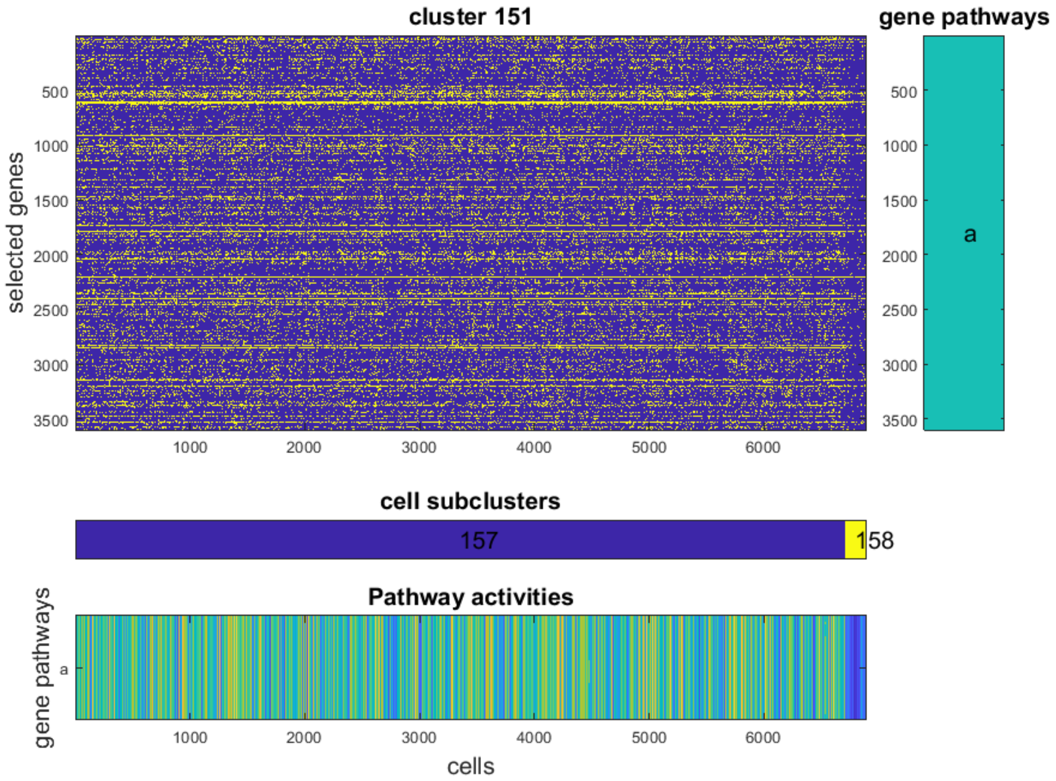


```
Remaining clusters to partition 7
Processing cluster 152 now ...
Processing data subset with 18134 genes and 754 cells:
Remove genes detected in <100 cells. Remaining 751 genes.  Elapsed time is 0.040730 seconds.
Iterate 10 random permutations for gene-gene similarity threshold ...   10 Elapsed time is 0.731353 seconds.
Compute gene-gene similarity ... Elapsed time is 0.033781 seconds.
Create gene-gene graph for clustering genes ...
Writing graph into file ... 100%Elapsed time is 0.281637 seconds.
Running ModularityOptimizer for clustering ...Elapsed time is 0.904441 seconds.
Gene-gene graph contains 4 pathways, 728 genes in total
Elapsed time is 0.924283 seconds.
Create cell-cell graph for clustering cells ...
Writing graph into file ... 100%Elapsed time is 0.082673 seconds.
Running ModularityOptimizer for clustering ...Elapsed time is 0.553250 seconds.
Cell-cell graph contains 9 cell types by community detection
Elapsed time is 0.570503 seconds.
Cell-cell graph contains 9 cell types after merging tiny cell clusters
Cell-cell graph contains 4 cell types after merging
Number of useful pathways is 4
```

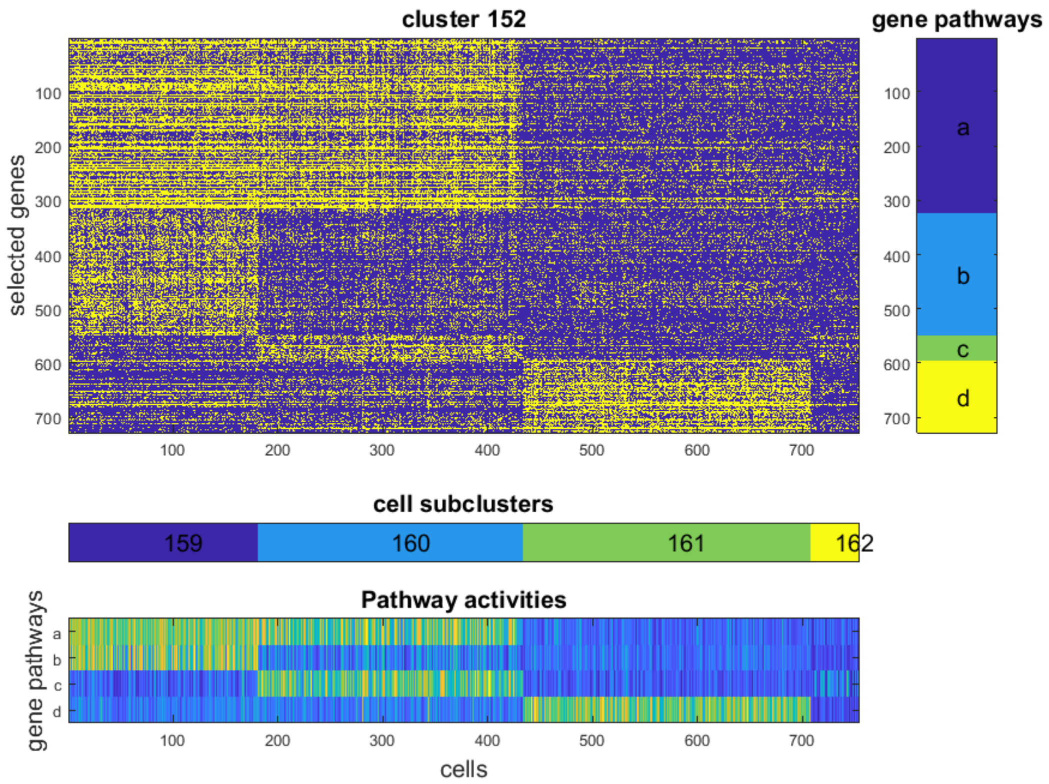


```
Remaining clusters to partition 10
Processing cluster 153 now ...
Processing data subset with 18134 genes and 81 cells:
Remove genes detected in <100 cells. Remaining 0 genes.  Elapsed time is 0.004257 seconds.

Remaining clusters to partition 9
Processing cluster 154 now ...
Processing data subset with 18134 genes and 5198 cells:
Remove genes detected in <100 cells. Remaining 7224 genes.  Elapsed time is 0.366246 seconds.
Iterate 10 random permutations for gene-gene similarity threshold ...   10 Elapsed time is 91.238634 seconds.
Compute gene-gene similarity ... Elapsed time is 5.818404 seconds.
Create gene-gene graph for clustering genes ...
Writing graph into file ... 100%Elapsed time is 0.472861 seconds.
Running ModularityOptimizer for clustering ...Elapsed time is 1.237306 seconds.
Gene-gene graph contains 5 pathways, 1748 genes in total
Elapsed time is 1.460025 seconds.
Create cell-cell graph for clustering cells ...
Writing graph into file ... 100%Elapsed time is 0.606269 seconds.
Running ModularityOptimizer for clustering ...Elapsed time is 3.863073 seconds.
Cell-cell graph contains 12 cell types by community detection
Elapsed time is 3.969413 seconds.
Cell-cell graph contains 11 cell types after merging tiny cell clusters
creating a total of 10 edges ...     10
Cell-cell graph contains 1 cell types after merging

Remaining clusters to partition 8
Processing cluster 155 now ...
Processing data subset with 18134 genes and 902 cells:
Remove genes detected in <100 cells. Remaining 3988 genes.  Elapsed time is 0.063503 seconds.
Iterate 10 random permutations for gene-gene similarity threshold ...   10 Elapsed time is 14.753776 seconds.
Compute gene-gene similarity ... Elapsed time is 1.012120 seconds.
Create gene-gene graph for clustering genes ...
Writing graph into file ... 100%Elapsed time is 0.117104 seconds.
Running ModularityOptimizer for clustering ...Elapsed time is 0.550862 seconds.
Gene-gene graph contains 7 pathways, 431 genes in total
Elapsed time is 0.657068 seconds.
Create cell-cell graph for clustering cells ...
Writing graph into file ... 100%Elapsed time is 0.103673 seconds.
Running ModularityOptimizer for clustering ...Elapsed time is 0.713396 seconds.
Cell-cell graph contains 8 cell types by community detection
Elapsed time is 0.735217 seconds.
Cell-cell graph contains 8 cell types after merging tiny cell clusters
creating a total of 7 edges ...      7
Cell-cell graph contains 1 cell types after merging

Remaining clusters to partition 7
Processing cluster 156 now ...
Processing data subset with 18134 genes and 9658 cells:
Remove genes detected in <100 cells. Remaining 10621 genes.  Elapsed time is 0.756133 seconds.
Iterate 10 random permutations for gene-gene similarity threshold ...   10 Elapsed time is 277.290800 seconds.
Compute gene-gene similarity ... Elapsed time is 18.016005 seconds.
Create gene-gene graph for clustering genes ...
Writing graph into file ... 100%Elapsed time is 6.803573 seconds.
Running ModularityOptimizer for clustering ...Elapsed time is 20.810987 seconds.
Gene-gene graph contains 7 pathways, 8052 genes in total
Elapsed time is 21.184838 seconds.
```

```
Create cell-cell graph for clustering cells ...
Writing graph into file ... 100%Elapsed time is 1.093163 seconds.
Running ModularityOptimizer for clustering ...Elapsed time is 5.930423 seconds.
Cell-cell graph contains 21 cell types by community detection
Elapsed time is 6.125667 seconds.
Cell-cell graph contains 17 cell types after merging tiny cell clusters
Cell-cell graph contains 2 cell types after merging
Number of useful pathways is 1
```

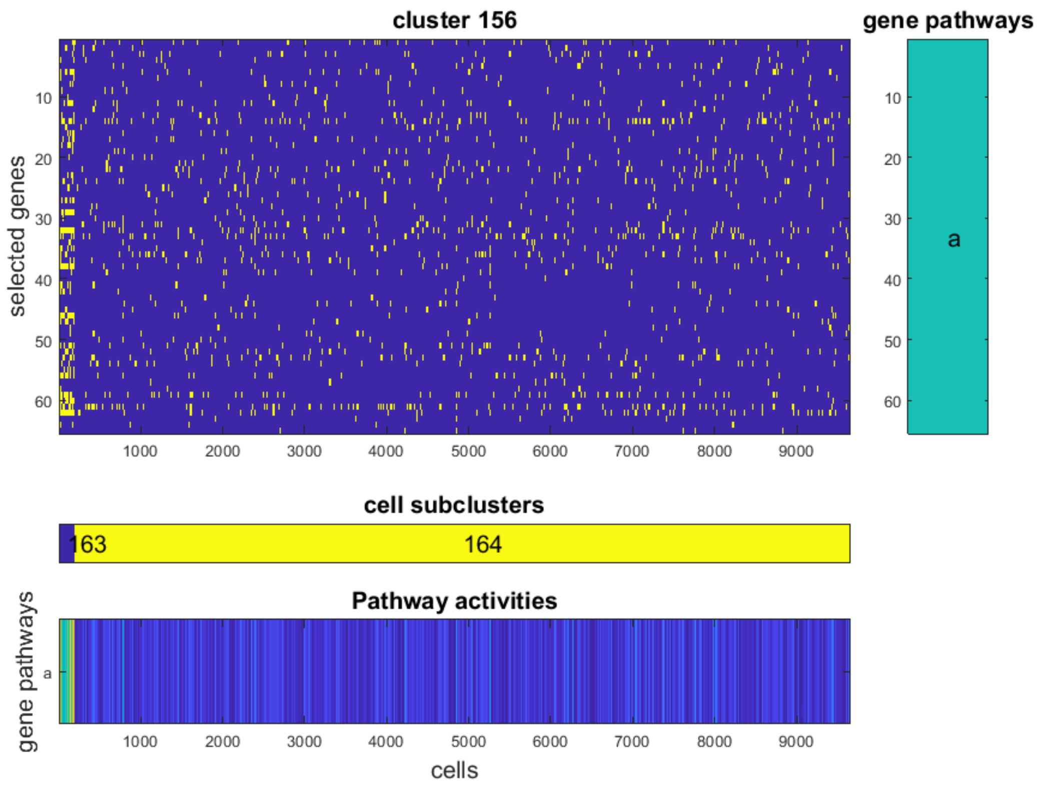


```
Remaining clusters to partition 8
Processing cluster 157 now ...
Processing data subset with 18134 genes and 6695 cells:
Remove genes detected in <100 cells. Remaining 9137 genes.  Elapsed time is 0.507091 seconds.
Iterate 10 random permutations for gene-gene similarity threshold ...   10 Elapsed time is 161.383745 seconds.
Compute gene-gene similarity ... Elapsed time is 10.837195 seconds.
Create gene-gene graph for clustering genes ...
Writing graph into file ... 100%Elapsed time is 4.835268 seconds.
Running ModularityOptimizer for clustering ...Elapsed time is 10.091653 seconds.
Gene-gene graph contains 3 pathways, 6683 genes in total
Elapsed time is 10.418954 seconds.
Create cell-cell graph for clustering cells ...
Writing graph into file ... 100%Elapsed time is 0.714650 seconds.
Running ModularityOptimizer for clustering ...Elapsed time is 3.631778 seconds.
Cell-cell graph contains 17 cell types by community detection
Elapsed time is 3.765843 seconds.
Cell-cell graph contains 14 cell types after merging tiny cell clusters
creating a total of 13 edges ...     13
Cell-cell graph contains 2 cell types after merging
Number of useful pathways is 1
```

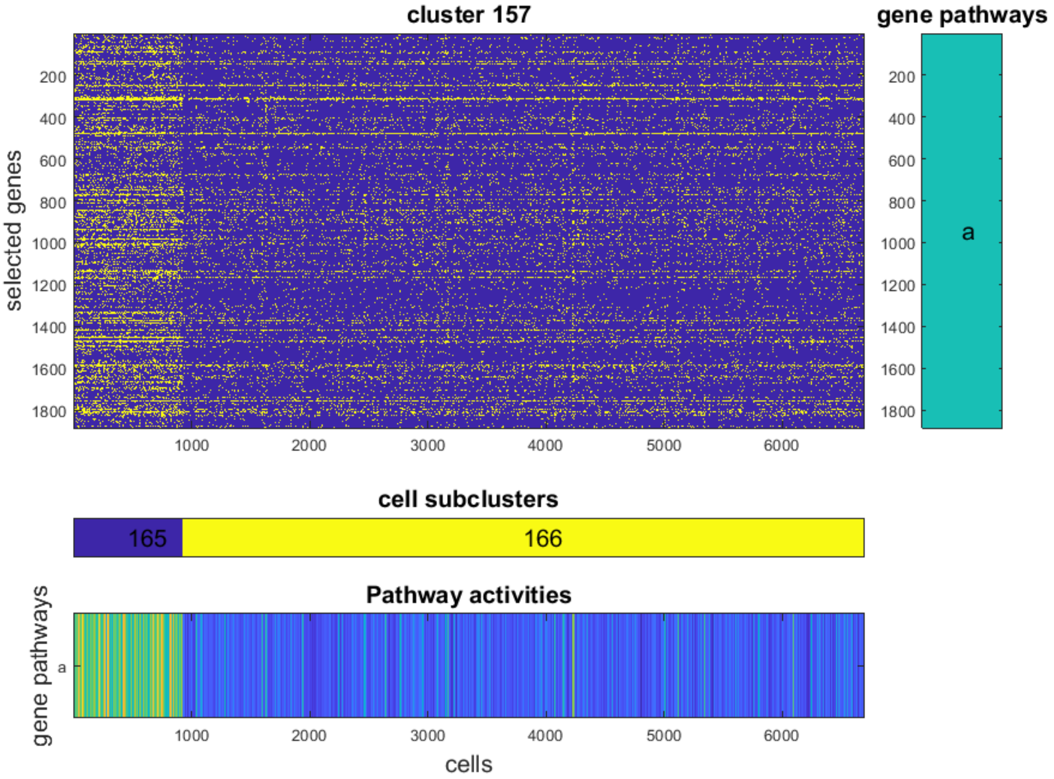


```
Remaining clusters to partition 9
Processing cluster 158 now ...
Processing data subset with 18134 genes and 199 cells:
Remove genes detected in <100 cells. Remaining 0 genes.  Elapsed time is 0.009764 seconds.

Remaining clusters to partition 8
Processing cluster 159 now ...
Processing data subset with 18134 genes and 181 cells:
Remove genes detected in <100 cells. Remaining 0 genes.  Elapsed time is 0.008310 seconds.

Remaining clusters to partition 7
Processing cluster 160 now ...
Processing data subset with 18134 genes and 252 cells:
Remove genes detected in <100 cells. Remaining 113 genes.  Elapsed time is 0.011994 seconds.
Iterate 10 random permutations for gene-gene similarity threshold ...   10 Elapsed time is 0.027243 seconds.
Compute gene-gene similarity ... Elapsed time is 0.001111 seconds.
Create gene-gene graph for clustering genes ...
Writing graph into file ... 100%Elapsed time is 0.005428 seconds.
Running ModularityOptimizer for clustering ...Elapsed time is 0.234950 seconds.
Gene-gene graph contains 3 pathways, 69 genes in total
Elapsed time is 0.240592 seconds.
Create cell-cell graph for clustering cells ...
Writing graph into file ... 100%Elapsed time is 0.029004 seconds.
Running ModularityOptimizer for clustering ...Elapsed time is 0.337002 seconds.
Cell-cell graph contains 7 cell types by community detection
Elapsed time is 0.344610 seconds.
Cell-cell graph contains 5 cell types after merging tiny cell clusters
Cell-cell graph contains 2 cell types after merging
Number of useful pathways is 1
```

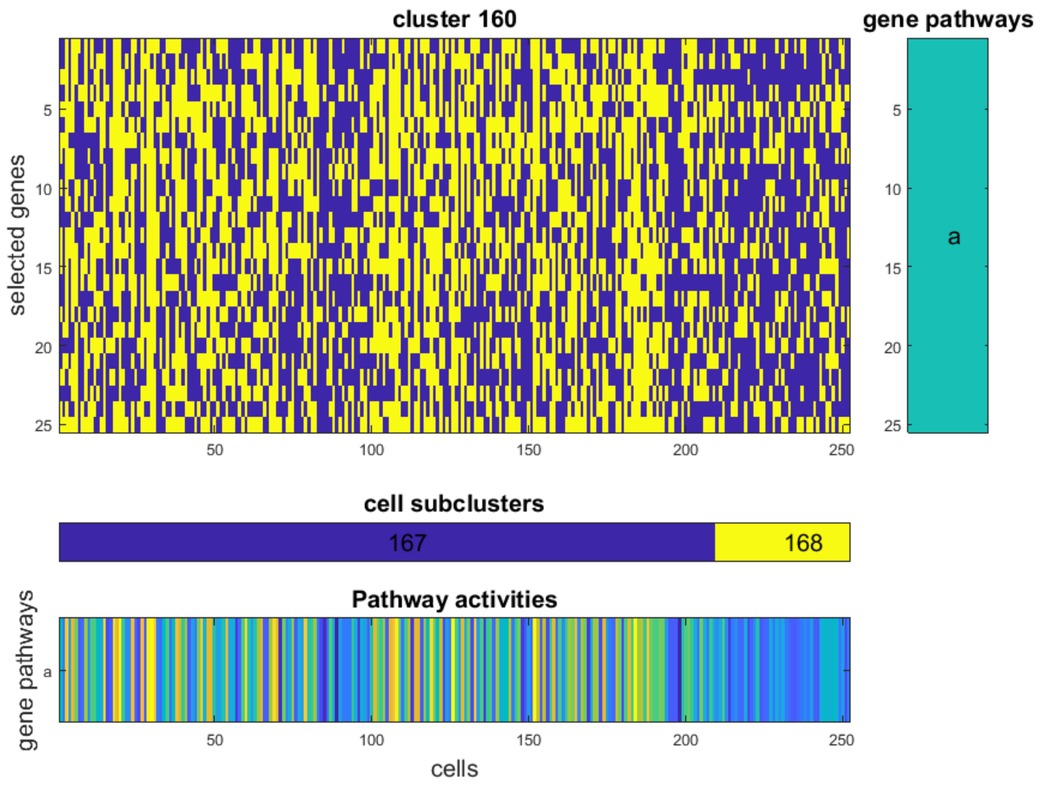


```
Remaining clusters to partition 8
Processing cluster 161 now ...
Processing data subset with 18134 genes and 274 cells:
Remove genes detected in <100 cells. Remaining 135 genes.  Elapsed time is 0.013522 seconds.
Iterate 10 random permutations for gene-gene similarity threshold ...   10 Elapsed time is 0.031945 seconds.
Compute gene-gene similarity ... Elapsed time is 0.001431 seconds.
Create gene-gene graph for clustering genes ...
Writing graph into file ... 101%Elapsed time is 0.001869 seconds.
Running ModularityOptimizer for clustering ...Elapsed time is 0.186681 seconds.
Gene-gene graph contains 0 pathways, 0 genes in total
Elapsed time is 0.192296 seconds.

Remaining clusters to partition 7
Processing cluster 162 now ...
Processing data subset with 18134 genes and 47 cells:
Remove genes detected in <100 cells. Remaining 0 genes.  Elapsed time is 0.001874 seconds.

Remaining clusters to partition 6
Processing cluster 163 now ...
Processing data subset with 18134 genes and 200 cells:
Remove genes detected in <100 cells. Remaining 9 genes.  Elapsed time is 0.009849 seconds.
Iterate 10 random permutations for gene-gene similarity threshold ...   10 Elapsed time is 0.006622 seconds.
Compute gene-gene similarity ... Elapsed time is 0.000249 seconds.
Create gene-gene graph for clustering genes ...
Writing graph into file ... 114%Elapsed time is 0.001321 seconds.
Running ModularityOptimizer for clustering ...Elapsed time is 0.149829 seconds.
Gene-gene graph contains 0 pathways, 0 genes in total
Elapsed time is 0.152925 seconds.

Remaining clusters to partition 5
Processing cluster 164 now ...
Processing data subset with 18134 genes and 9458 cells:
Remove genes detected in <100 cells. Remaining 10567 genes.  Elapsed time is 0.774704 seconds.
Iterate 10 random permutations for gene-gene similarity threshold ...   10 Elapsed time is 272.372075 seconds.
Compute gene-gene similarity ... Elapsed time is 17.873109 seconds.
Create gene-gene graph for clustering genes ...
Writing graph into file ... 100%Elapsed time is 6.476881 seconds.
Running ModularityOptimizer for clustering ...Elapsed time is 22.446400 seconds.
Gene-gene graph contains 5 pathways, 7931 genes in total
Elapsed time is 22.826820 seconds.
Create cell-cell graph for clustering cells ...
Writing graph into file ... 100%Elapsed time is 1.086514 seconds.
Running ModularityOptimizer for clustering ...Elapsed time is 6.151306 seconds.
Cell-cell graph contains 19 cell types by community detection
Elapsed time is 6.342317 seconds.
Cell-cell graph contains 15 cell types after merging tiny cell clusters
creating a total of 14 edges ...      14
Cell-cell graph contains 2 cell types after merging
Number of useful pathways is 1
```

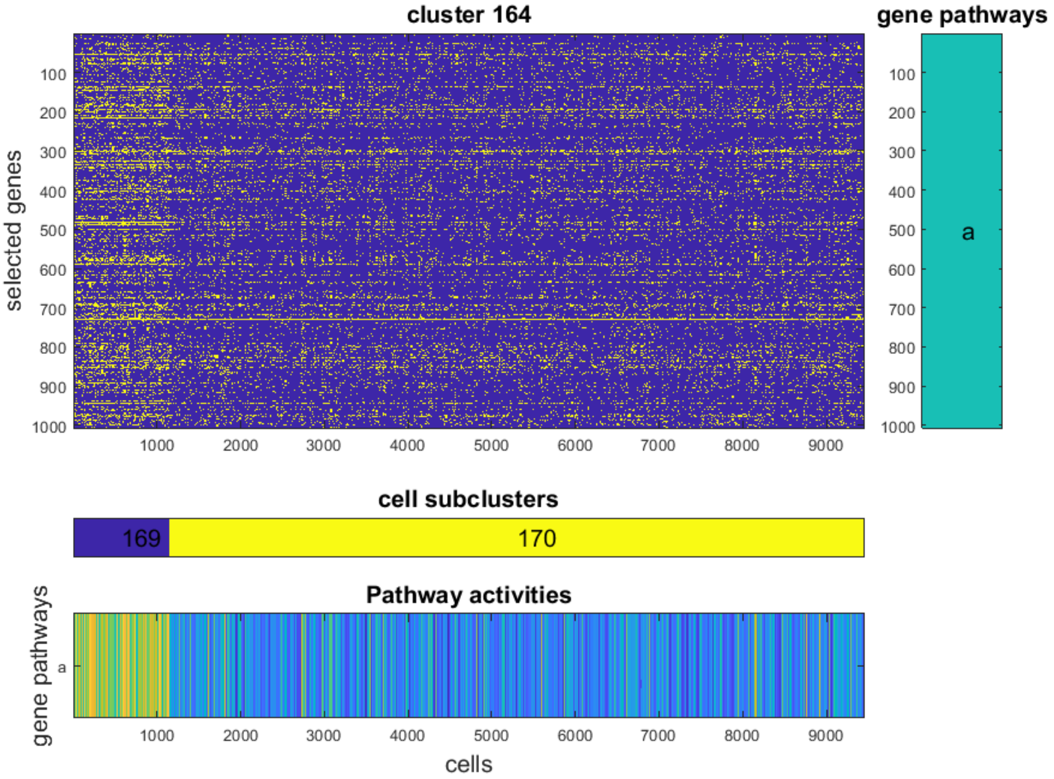


```
Remaining clusters to partition 6
Processing cluster 165 now ...
Processing data subset with 18134 genes and 921 cells:
Remove genes detected in <100 cells. Remaining 3489 genes.  Elapsed time is 0.061040 seconds.
Iterate 10 random permutations for gene-gene similarity threshold ...   10 Elapsed time is 11.369109 seconds.
Compute gene-gene similarity ... Elapsed time is 0.801662 seconds.
Create gene-gene graph for clustering genes ...
Writing graph into file ... 100%Elapsed time is 0.184993 seconds.
Running ModularityOptimizer for clustering ...Elapsed time is 0.874588 seconds.
Gene-gene graph contains 4 pathways, 1541 genes in total
Elapsed time is 0.970532 seconds.
Create cell-cell graph for clustering cells ...
Writing graph into file ... 100%Elapsed time is 0.098642 seconds.
Running ModularityOptimizer for clustering ...Elapsed time is 0.583970 seconds.
Cell-cell graph contains 10 cell types by community detection
Elapsed time is 0.604859 seconds.
Cell-cell graph contains 9 cell types after merging tiny cell clusters
creating a total of 8 edges ...      8
Cell-cell graph contains 1 cell types after merging

Remaining clusters to partition 5
Processing cluster 166 now ...
Processing data subset with 18134 genes and 5774 cells:
Remove genes detected in <100 cells. Remaining 8575 genes.  Elapsed time is 0.455721 seconds.
Iterate 10 random permutations for gene-gene similarity threshold ...   10 Elapsed time is 135.008777 seconds.
Compute gene-gene similarity ... Elapsed time is 9.197431 seconds.
Create gene-gene graph for clustering genes ...
Writing graph into file ... 100%Elapsed time is 3.200778 seconds.
Running ModularityOptimizer for clustering ...Elapsed time is 7.364894 seconds.
Gene-gene graph contains 4 pathways, 5706 genes in total
Elapsed time is 7.652552 seconds.
Create cell-cell graph for clustering cells ...
Writing graph into file ... 100%Elapsed time is 0.655060 seconds.
Running ModularityOptimizer for clustering ...Elapsed time is 3.409603 seconds.
Cell-cell graph contains 16 cell types by community detection
Elapsed time is 3.528488 seconds.
Cell-cell graph contains 14 cell types after merging tiny cell clusters
creating a total of 13 edges ...     13
Cell-cell graph contains 2 cell types after merging
Number of useful pathways is 1
```

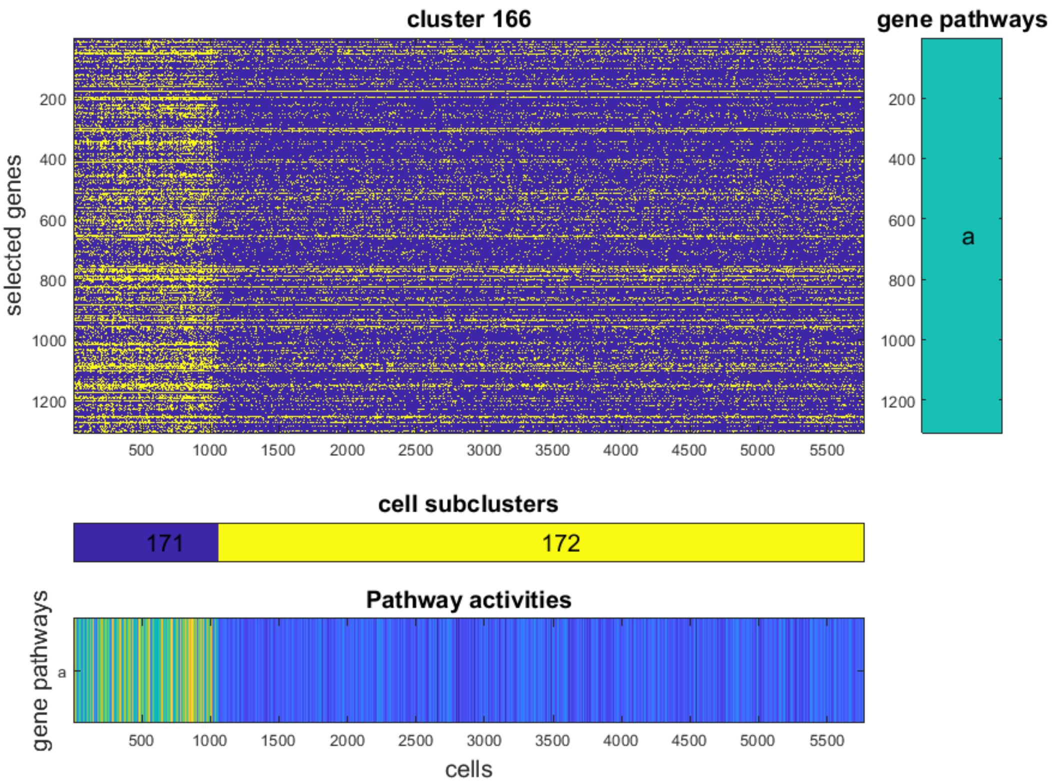


```
Remaining clusters to partition 6
Processing cluster 167 now ...
Processing data subset with 18134 genes and 209 cells:
Remove genes detected in <100 cells. Remaining 19 genes.  Elapsed time is 0.010931 seconds.
Iterate 10 random permutations for gene-gene similarity threshold ...   10 Elapsed time is 0.006777 seconds.
Compute gene-gene similarity ... Elapsed time is 0.000299 seconds.
Create gene-gene graph for clustering genes ...
Writing graph into file ... 102%Elapsed time is 0.001718 seconds.
Running ModularityOptimizer for clustering ...Elapsed time is 0.169064 seconds.
Gene-gene graph contains 0 pathways, 0 genes in total
Elapsed time is 0.172108 seconds.

Remaining clusters to partition 5
Processing cluster 168 now ...
Processing data subset with 18134 genes and 43 cells:
Remove genes detected in <100 cells. Remaining 0 genes.  Elapsed time is 0.001600 seconds.

Remaining clusters to partition 4
Processing cluster 169 now ...
Processing data subset with 18134 genes and 1149 cells:
Remove genes detected in <100 cells. Remaining 4216 genes.  Elapsed time is 0.073840 seconds.
Iterate 10 random permutations for gene-gene similarity threshold ...   10 Elapsed time is 16.968074 seconds.
Compute gene-gene similarity ... Elapsed time is 1.299026 seconds.
Create gene-gene graph for clustering genes ...
Writing graph into file ... 100%Elapsed time is 0.148953 seconds.
Running ModularityOptimizer for clustering ...Elapsed time is 0.579198 seconds.
Gene-gene graph contains 6 pathways, 918 genes in total
Elapsed time is 0.691933 seconds.
Create cell-cell graph for clustering cells ...
Writing graph into file ... 100%Elapsed time is 0.134786 seconds.
Running ModularityOptimizer for clustering ...Elapsed time is 0.772390 seconds.
Cell-cell graph contains 8 cell types by community detection
Elapsed time is 0.797324 seconds.
Cell-cell graph contains 8 cell types after merging tiny cell clusters
creating a total of 7 edges ...      7
Cell-cell graph contains 2 cell types after merging
Number of useful pathways is 1
```

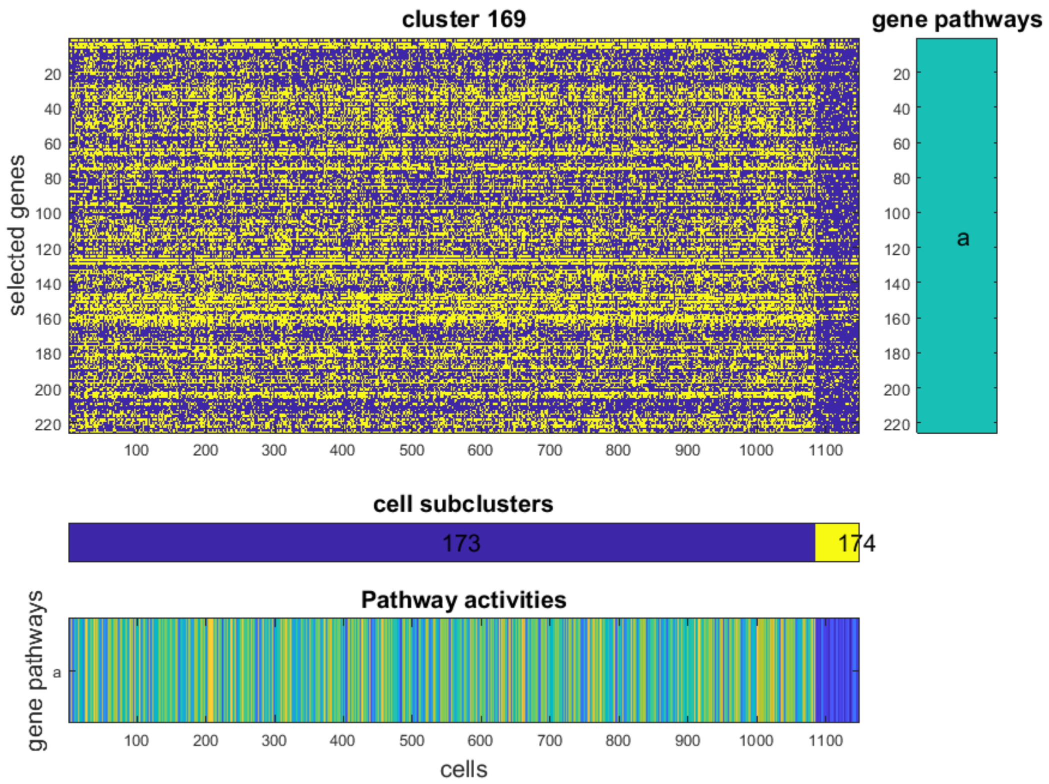


```
Remaining clusters to partition 5
Processing cluster 170 now ...
Processing data subset with 18134 genes and 8309 cells:
Remove genes detected in <100 cells. Remaining 10268 genes.  Elapsed time is 0.654850 seconds.
Iterate 10 random permutations for gene-gene similarity threshold ...   10 Elapsed time is 236.280117 seconds.
Compute gene-gene similarity ... Elapsed time is 15.417891 seconds.
Create gene-gene graph for clustering genes ...
Writing graph into file ... 100%Elapsed time is 7.107484 seconds.
Running ModularityOptimizer for clustering ...Elapsed time is 21.903907 seconds.
Gene-gene graph contains 5 pathways, 8069 genes in total
Elapsed time is 22.265433 seconds.
Create cell-cell graph for clustering cells ...
Writing graph into file ... 100%Elapsed time is 0.928877 seconds.
Running ModularityOptimizer for clustering ...Elapsed time is 5.278058 seconds.
Cell-cell graph contains 19 cell types by community detection
Elapsed time is 5.447989 seconds.
Cell-cell graph contains 17 cell types after merging tiny cell clusters
creating a total of 16 edges ...     16
Cell-cell graph contains 2 cell types after merging
Number of useful pathways is 1
```

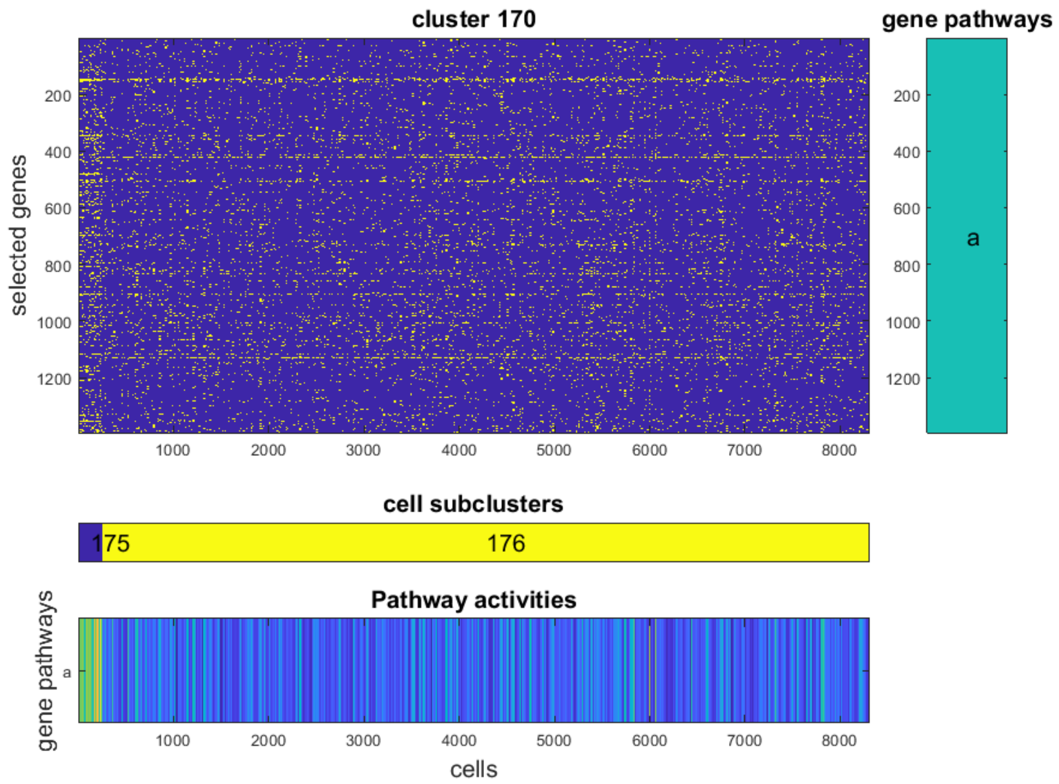


```
Remaining clusters to partition 6
Processing cluster 171 now ...
Processing data subset with 18134 genes and 1057 cells:
Remove genes detected in <100 cells. Remaining 3396 genes.  Elapsed time is 0.067059 seconds.
Iterate 10 random permutations for gene-gene similarity threshold ...   10 Elapsed time is 11.175153 seconds.
Compute gene-gene similarity ... Elapsed time is 0.763835 seconds.
Create gene-gene graph for clustering genes ...
Writing graph into file ... 100%Elapsed time is 0.560926 seconds.
Running ModularityOptimizer for clustering ...Elapsed time is 1.235313 seconds.
Gene-gene graph contains 2 pathways, 2016 genes in total
Elapsed time is 1.328506 seconds.
Create cell-cell graph for clustering cells ...
Writing graph into file ... 100%Elapsed time is 0.117860 seconds.
Running ModularityOptimizer for clustering ...Elapsed time is 0.659155 seconds.
Cell-cell graph contains 10 cell types by community detection
Elapsed time is 0.685549 seconds.
Cell-cell graph contains 2 cell types after merging tiny cell clusters
creating a total of 1 edges ...      1
Cell-cell graph contains 1 cell types after merging

Remaining clusters to partition 5
Processing cluster 172 now ...
Processing data subset with 18134 genes and 4717 cells:
Remove genes detected in <100 cells. Remaining 7767 genes.  Elapsed time is 0.357142 seconds.
Iterate 10 random permutations for gene-gene similarity threshold ...   10 Elapsed time is 98.279015 seconds.
Compute gene-gene similarity ... Elapsed time is 6.815692 seconds.
Create gene-gene graph for clustering genes ...
Writing graph into file ... 100%Elapsed time is 0.796752 seconds.
Running ModularityOptimizer for clustering ...Elapsed time is 1.780244 seconds.
Gene-gene graph contains 4 pathways, 3605 genes in total
Elapsed time is 2.036363 seconds.
Create cell-cell graph for clustering cells ...
Writing graph into file ... 100%Elapsed time is 0.638218 seconds.
Running ModularityOptimizer for clustering ...Elapsed time is 2.761274 seconds.
Cell-cell graph contains 16 cell types by community detection
Elapsed time is 2.857802 seconds.
Cell-cell graph contains 10 cell types after merging tiny cell clusters
creating a total of 9 edges ...      9
Cell-cell graph contains 2 cell types after merging
Number of useful pathways is 1
```

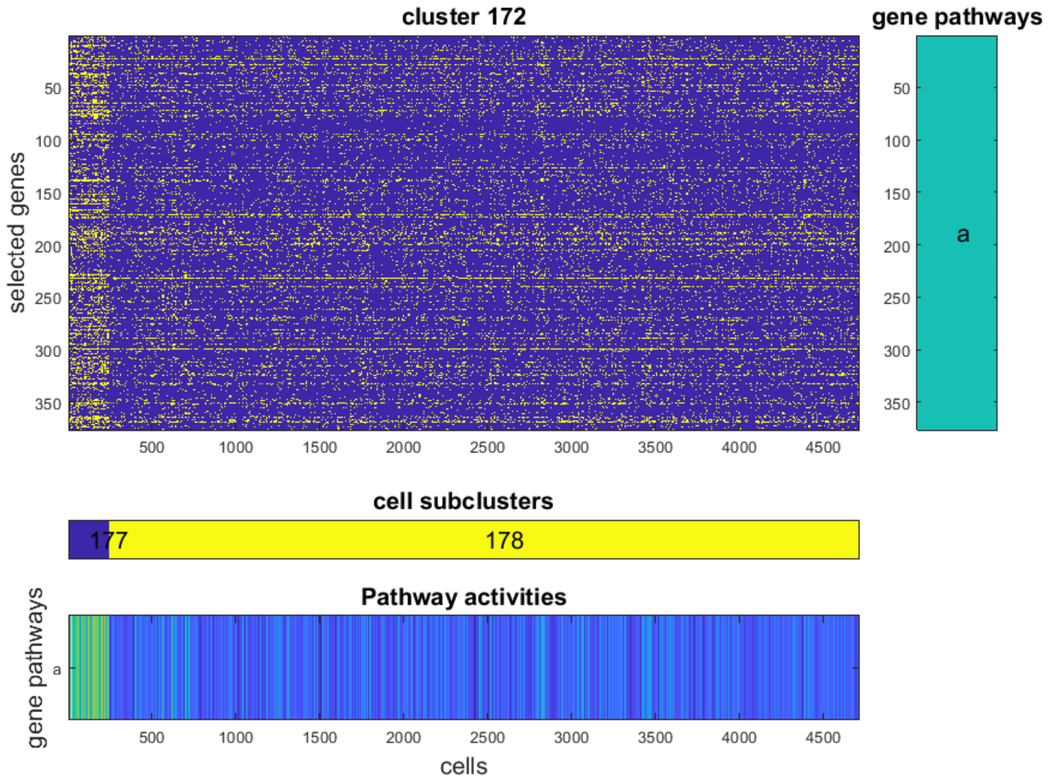


```
Remaining clusters to partition 6
Processing cluster 173 now ...
Processing data subset with 18134 genes and 1084 cells:
Remove genes detected in <100 cells. Remaining 4013 genes.  Elapsed time is 0.069533 seconds.
Iterate 10 random permutations for gene-gene similarity threshold ...   10 Elapsed time is 15.764975 seconds.
Compute gene-gene similarity ... Elapsed time is 1.099115 seconds.
Create gene-gene graph for clustering genes ...
Writing graph into file ... 100%Elapsed time is 0.140088 seconds.
Running ModularityOptimizer for clustering ...Elapsed time is 0.676244 seconds.
Gene-gene graph contains 5 pathways, 881 genes in total
Elapsed time is 0.784086 seconds.
Create cell-cell graph for clustering cells ...
Writing graph into file ... 100%Elapsed time is 0.125711 seconds.
Running ModularityOptimizer for clustering ...Elapsed time is 0.754332 seconds.
Cell-cell graph contains 8 cell types by community detection
Elapsed time is 0.780531 seconds.
Cell-cell graph contains 8 cell types after merging tiny cell clusters
creating a total of 7 edges ...       7
Cell-cell graph contains 1 cell types after merging

Remaining clusters to partition 5
Processing cluster 174 now ...
Processing data subset with 18134 genes and 65 cells:
Remove genes detected in <100 cells. Remaining 0 genes.  Elapsed time is 0.003988 seconds.

Remaining clusters to partition 4
Processing cluster 175 now ...
Processing data subset with 18134 genes and 253 cells:
Remove genes detected in <100 cells. Remaining 507 genes.  Elapsed time is 0.014569 seconds.
Iterate 10 random permutations for gene-gene similarity threshold ...   10 Elapsed time is 0.224124 seconds.
Compute gene-gene similarity ... Elapsed time is 0.014229 seconds.
Create gene-gene graph for clustering genes ...
Writing graph into file ... 100%Elapsed time is 0.008037 seconds.
Running ModularityOptimizer for clustering ...Elapsed time is 0.276122 seconds.
Gene-gene graph contains 4 pathways, 176 genes in total
Elapsed time is 0.290410 seconds.
Create cell-cell graph for clustering cells ...
Writing graph into file ... 100%Elapsed time is 0.027162 seconds.
Running ModularityOptimizer for clustering ...Elapsed time is 0.312916 seconds.
Cell-cell graph contains 5 cell types by community detection
Elapsed time is 0.320784 seconds.
Cell-cell graph contains 5 cell types after merging tiny cell clusters
Cell-cell graph contains 3 cell types after merging
Number of useful pathways is 3
```

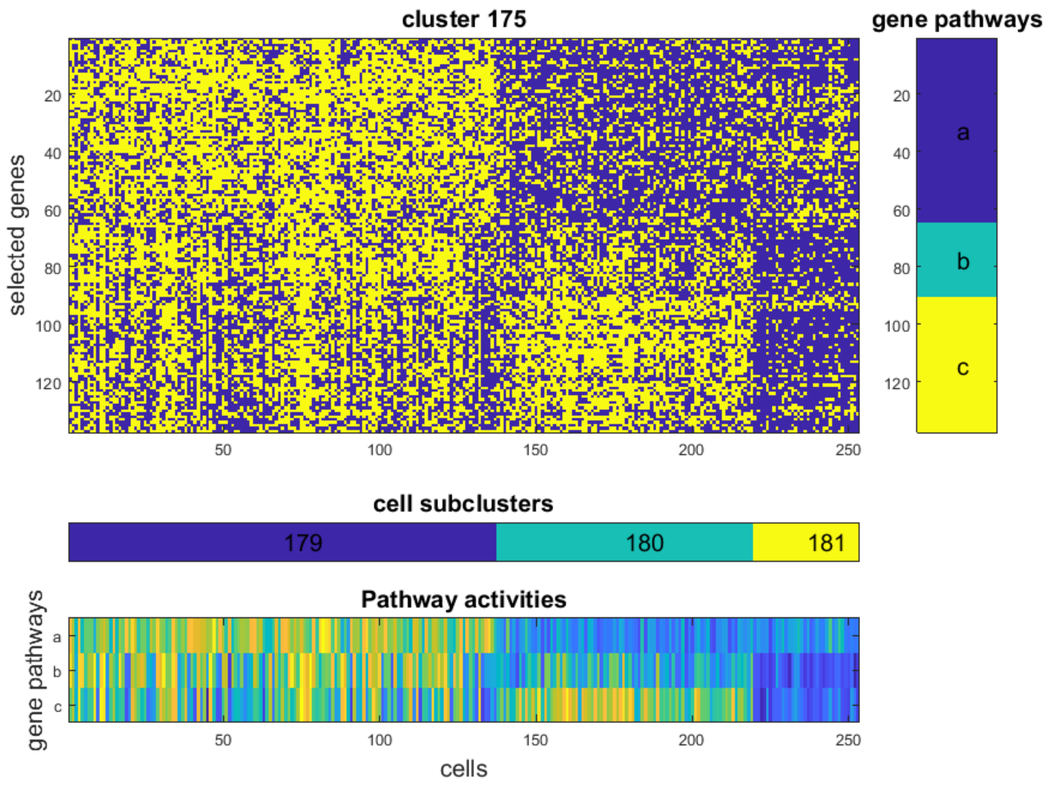


```
Remaining clusters to partition 6
Processing cluster 176 now ...
Processing data subset with 18134 genes and 8056 cells:
Remove genes detected in <100 cells. Remaining 10141 genes.  Elapsed time is 0.742815 seconds.
Iterate 10 random permutations for gene-gene similarity threshold ...   10 Elapsed time is 223.390112 seconds.
Compute gene-gene similarity ... Elapsed time is 14.487439 seconds.
Create gene-gene graph for clustering genes ...
Writing graph into file ... 100%Elapsed time is 6.528531 seconds.
Running ModularityOptimizer for clustering ...Elapsed time is 23.661887 seconds.
Gene-gene graph contains 5 pathways, 7648 genes in total
Elapsed time is 24.017988 seconds.
Create cell-cell graph for clustering cells ...
Writing graph into file ... 100%Elapsed time is 0.898089 seconds.
Running ModularityOptimizer for clustering ...Elapsed time is 5.053481 seconds.
Cell-cell graph contains 17 cell types by community detection
Elapsed time is 5.214253 seconds.
Cell-cell graph contains 13 cell types after merging tiny cell clusters
creating a total of 12 edges ...      12
Cell-cell graph contains 1 cell types after merging

Remaining clusters to partition 5
Processing cluster 177 now ...
Processing data subset with 18134 genes and 237 cells:
Remove genes detected in <100 cells. Remaining 314 genes.  Elapsed time is 0.013627 seconds.
Iterate 10 random permutations for gene-gene similarity threshold ...   10 Elapsed time is 0.073714 seconds.
Compute gene-gene similarity ... Elapsed time is 0.003545 seconds.
Create gene-gene graph for clustering genes ...
Writing graph into file ... 101%Elapsed time is 0.002658 seconds.
Running ModularityOptimizer for clustering ...Elapsed time is 0.200858 seconds.
Gene-gene graph contains 0 pathways, 0 genes in total
Elapsed time is 0.209923 seconds.

Remaining clusters to partition 4
Processing cluster 178 now ...
Processing data subset with 18134 genes and 4480 cells:
Remove genes detected in <100 cells. Remaining 7610 genes.  Elapsed time is 0.314591 seconds.
Iterate 10 random permutations for gene-gene similarity threshold ...   10 Elapsed time is 90.589652 seconds.
Compute gene-gene similarity ... Elapsed time is 6.082141 seconds.
Create gene-gene graph for clustering genes ...
Writing graph into file ... 100%Elapsed time is 0.708723 seconds.
Running ModularityOptimizer for clustering ...Elapsed time is 1.560169 seconds.
Gene-gene graph contains 4 pathways, 3432 genes in total
Elapsed time is 1.804451 seconds.
Create cell-cell graph for clustering cells ...
Writing graph into file ... 100%Elapsed time is 0.503802 seconds.
Running ModularityOptimizer for clustering ...Elapsed time is 2.543808 seconds.
Cell-cell graph contains 12 cell types by community detection
Elapsed time is 2.635003 seconds.
Cell-cell graph contains 10 cell types after merging tiny cell clusters
creating a total of 9 edges ...      9
Cell-cell graph contains 2 cell types after merging
Number of useful pathways is 1
```

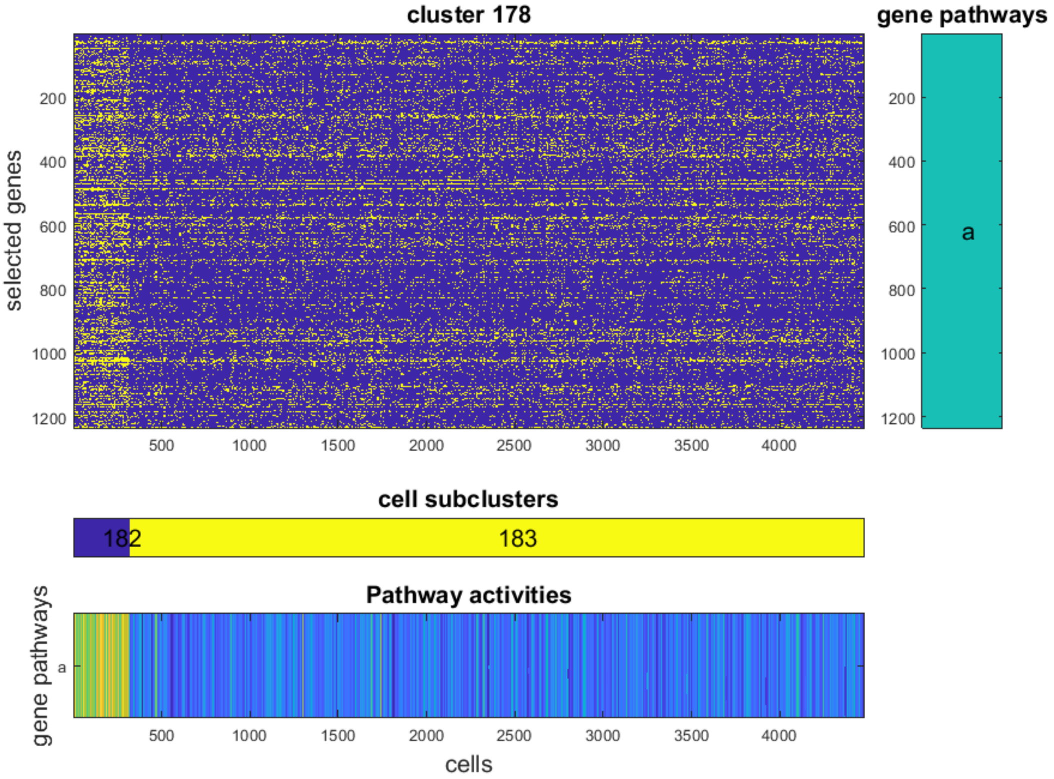


```
Remaining clusters to partition 5
Processing cluster 179 now ...
Processing data subset with 18134 genes and 137 cells:
Remove genes detected in <100 cells. Remaining 0 genes.  Elapsed time is 0.006750 seconds.

Remaining clusters to partition 4
Processing cluster 180 now ...
Processing data subset with 18134 genes and 82 cells:
Remove genes detected in <100 cells. Remaining 0 genes.  Elapsed time is 0.004254 seconds.

Remaining clusters to partition 3
Processing cluster 181 now ...
Processing data subset with 18134 genes and 34 cells:
Remove genes detected in <100 cells. Remaining 0 genes.  Elapsed time is 0.001194 seconds.

Remaining clusters to partition 2
Processing cluster 182 now ...
Processing data subset with 18134 genes and 324 cells:
Remove genes detected in <100 cells. Remaining 957 genes.  Elapsed time is 0.016668 seconds.
Iterate 10 random permutations for gene-gene similarity threshold ...   10 Elapsed time is 0.771902 seconds.
Compute gene-gene similarity ... Elapsed time is 0.051737 seconds.
Create gene-gene graph for clustering genes ...
Writing graph into file ... 101%Elapsed time is 0.007879 seconds.
Running ModularityOptimizer for clustering ...Elapsed time is 0.249918 seconds.
Gene-gene graph contains 0 pathways, 0 genes in total
Elapsed time is 0.272490 seconds.

Remaining clusters to partition 1
Processing cluster 183 now ...
Processing data subset with 18134 genes and 4156 cells:
Remove genes detected in <100 cells. Remaining 7381 genes.  Elapsed time is 0.313276 seconds.
Iterate 10 random permutations for gene-gene similarity threshold ...   10 Elapsed time is 82.068572 seconds.
Compute gene-gene similarity ... Elapsed time is 5.402817 seconds.
Create gene-gene graph for clustering genes ...
Writing graph into file ... 100%Elapsed time is 0.504492 seconds.
Running ModularityOptimizer for clustering ...Elapsed time is 1.218574 seconds.
Gene-gene graph contains 6 pathways, 2491 genes in total
Elapsed time is 1.449950 seconds.
Create cell-cell graph for clustering cells ...
Writing graph into file ... 100%Elapsed time is 0.485185 seconds.
Running ModularityOptimizer for clustering ...Elapsed time is 2.875712 seconds.
Cell-cell graph contains 10 cell types by community detection
Elapsed time is 2.961616 seconds.
Cell-cell graph contains 9 cell types after merging tiny cell clusters
creating a total of 8 edges ...      8
Cell-cell graph contains 2 cell types after merging
Number of useful pathways is 1
```

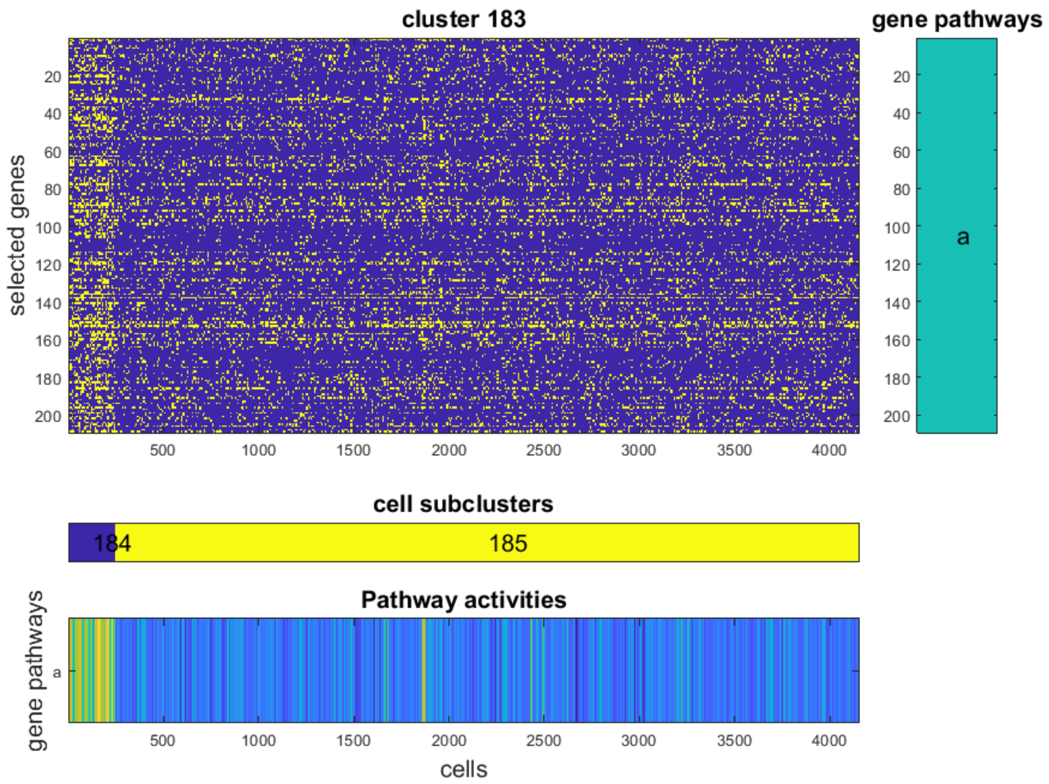


```
Remaining clusters to partition 2
Processing cluster 184 now ...
Processing data subset with 18134 genes and 247 cells:
Remove genes detected in <100 cells. Remaining 448 genes.  Elapsed time is 0.013434 seconds.
Iterate 10 random permutations for gene-gene similarity threshold ...   10 Elapsed time is 0.161619 seconds.
Compute gene-gene similarity ... Elapsed time is 0.010453 seconds.
Create gene-gene graph for clustering genes ...
Writing graph into file ... 101%Elapsed time is 0.003309 seconds.
Running ModularityOptimizer for clustering ...Elapsed time is 0.237055 seconds.
Gene-gene graph contains 0 pathways, 0 genes in total
Elapsed time is 0.249063 seconds.

Remaining clusters to partition 1
Processing cluster 185 now ...
Processing data subset with 18134 genes and 3909 cells:
Remove genes detected in <100 cells. Remaining 7196 genes.  Elapsed time is 0.279122 seconds.
Iterate 10 random permutations for gene-gene similarity threshold ...   10 Elapsed time is 75.821408 seconds.
Compute gene-gene similarity ... Elapsed time is 5.253818 seconds.
Create gene-gene graph for clustering genes ...
Writing graph into file ... 100%Elapsed time is 0.478176 seconds.
Running ModularityOptimizer for clustering ...Elapsed time is 1.223466 seconds.
Gene-gene graph contains 6 pathways, 2383 genes in total
Elapsed time is 1.444004 seconds.
Create cell-cell graph for clustering cells ...
Writing graph into file ... 100%Elapsed time is 0.452233 seconds.
Running ModularityOptimizer for clustering ...Elapsed time is 2.655086 seconds.
Cell-cell graph contains 13 cell types by community detection
Elapsed time is 2.734023 seconds.
Cell-cell graph contains 13 cell types after merging tiny cell clusters
creating a total of 12 edges ...     12
Cell-cell graph contains 1 cell types after merging
```

number of cooccurrence clusters

```
number_of_cooccurrence_clusters = length(unique(cooc.cell_labels))
```

```
number_of_cooccurrence_clusters =

    98
```

Published with MATLAB® R2017b

— Supplementary Note 4 —

Contents

- addpath to all tools
- initiate one instance of the "cooccurrence_clustering_analysis" class
- read data prepawed in matlab file
- filter the data by removing genes and cells (same as Seurat tutorial on this data)
- binarize data
- cooccurrence clustering
- number of cooccurrence clusters

addpath to all tools

```
addpath(genpath('..\tools\'))
```

initiate one instance of the "cooccurrence_clustering_analysis" class

```
cooc = cooccurrence_clustering_analysis;
```

read data prepawed in matlab file

```
cooc = cooc.ReadMatlab('TM_facs_mat.mat');
```

filter the data by removing genes and cells (same as Seurat tutorial on this data)

```
cooc.initial_filtering_min_num_cells = 10;
cooc.initial_filtering_min_num_genes = 0;
cooc.initial_filtering_max_num_genes = Inf;
cooc.initial_filtering_max_percent_mito = 1;
cooc = cooc.initial_filtering_of_data(1);
```

```
Data after initial filtering 22327 genes * 53760 cells.
```

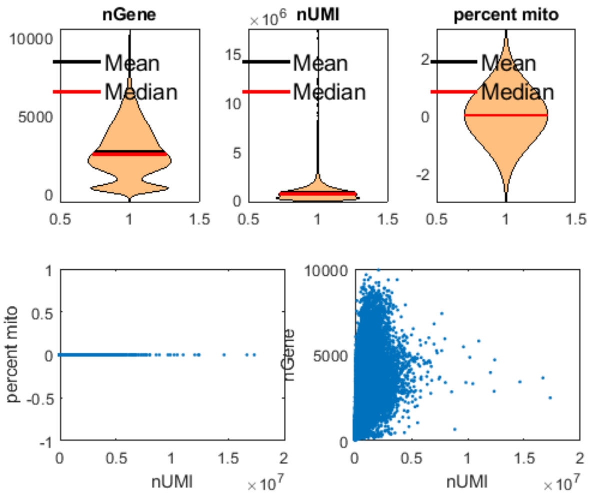

binarize data

```
cooc.binarization_threshold = 0;
cooc.binary_data = full(double(cooc.data>cooc.binarization_threshold));
```

cooccurrence clustering

```
cooc.cooccurrence_min_expressed_cells = 100;            % genes will only be considered if detected in >= minimum number of cells, and undetected in >= minimum number of cells
cooc.cooccurrence_min_pathway_size = 20;                % only considered gene clusters of size >= this threshold
cooc.cooccurrence_min_population_size = 10;             % only considered gene clusters of size >= this threshold
cooc.cooccurrence_snr_merge_threshold = 1.5;            % threshold for merging Louvain communities, based on snr of average detection of each gene cluster
cooc.cooccurrence_mean_diff_merge_threshold = 0.5;      % threshold for merging Louvain communities
cooc.cooccurrence_mean_ratio_merge_threshold = 2;       % threshold for merging Louvain communities


cooc = cooc.iterative_cooccurance_clustering;
```

```
Remaining clusters to partition 1
Processing cluster 0 now ...
Processing data subset with 22327 genes and 53760 cells:
Remove genes detected in <100 cells. Remaining 18360 genes.  Elapsed time is 16.754922 seconds.
Iterate 10 random permutations for gene-gene similarity threshold ...   10 Elapsed time is 4513.105316 seconds.
Compute gene-gene similarity ... Elapsed time is 211.325458 seconds.
Create gene-gene graph for clustering genes ...
Writing graph into file ... 100%Elapsed time is 503.556410 seconds.
Running ModularityOptimizer for clustering ...Elapsed time is 1368.983184 seconds.
Gene-gene graph contains 6 pathways, 18333 genes in total
Elapsed time is 1370.169865 seconds.
```

```
Create cell-cell graph for clustering cells ...
Writing graph into file ... 100%Elapsed time is 6.329618 seconds.
Running ModularityOptimizer for clustering ...Elapsed time is 73.850265 seconds.
Cell-cell graph contains 31 cell types by community detection
Elapsed time is 74.949272 seconds.
Cell-cell graph contains 15 cell types after merging tiny cell clusters
Cell-cell graph contains 2 cell types after merging
Number of useful pathways is 1
```

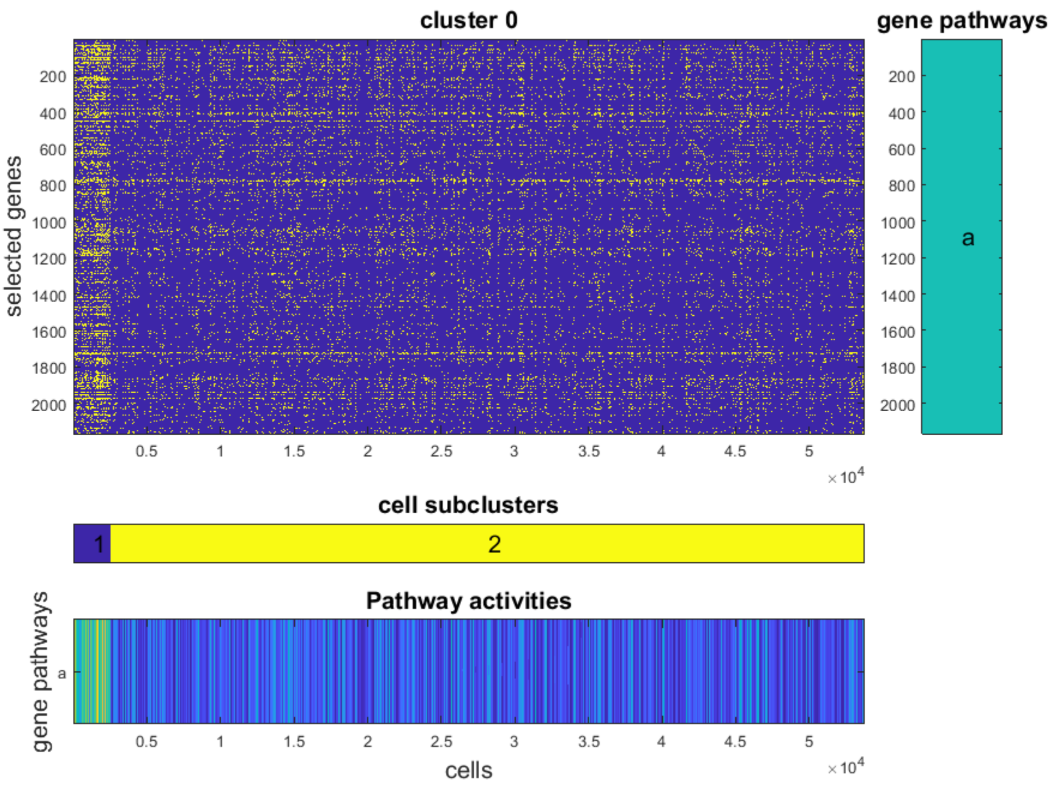


```
Remaining clusters to partition 2
Processing cluster 1 now ...
Processing data subset with 22327 genes and 2576 cells:
Remove genes detected in <100 cells. Remaining 11519 genes.  Elapsed time is 0.256535 seconds.
Iterate 10 random permutations for gene-gene similarity threshold ...   10 Elapsed time is 146.487524 seconds.
Compute gene-gene similarity ... Elapsed time is 10.819913 seconds.
Create gene-gene graph for clustering genes ...
Writing graph into file ... 100%Elapsed time is 20.936430 seconds.
Running ModularityOptimizer for clustering ...Elapsed time is 61.821088 seconds.
Gene-gene graph contains 5 pathways, 10981 genes in total
Elapsed time is 62.263156 seconds.
Create cell-cell graph for clustering cells ...
Writing graph into file ... 100%Elapsed time is 0.321605 seconds.
Running ModularityOptimizer for clustering ...Elapsed time is 1.439442 seconds.
Cell-cell graph contains 15 cell types by community detection
Elapsed time is 1.494934 seconds.
Cell-cell graph contains 14 cell types after merging tiny cell clusters
creating a total of 13 edges ...     13
Cell-cell graph contains 1 cell types after merging

Remaining clusters to partition 1
Processing cluster 2 now ...
Processing data subset with 22327 genes and 51184 cells:
Remove genes detected in <100 cells. Remaining 18280 genes.  Elapsed time is 6.176651 seconds.
Iterate 10 random permutations for gene-gene similarity threshold ...   10 Elapsed time is 3926.600551 seconds.
Compute gene-gene similarity ... Elapsed time is 241.822365 seconds.
Create gene-gene graph for clustering genes ...
Writing graph into file ... 100%Elapsed time is 506.559452 seconds.
Running ModularityOptimizer for clustering ...Elapsed time is 1374.835127 seconds.
Gene-gene graph contains 7 pathways, 18246 genes in total
Elapsed time is 1376.196595 seconds.
Create cell-cell graph for clustering cells ...
Writing graph into file ... 100%Elapsed time is 6.355144 seconds.
Running ModularityOptimizer for clustering ...Elapsed time is 80.966220 seconds.
Cell-cell graph contains 34 cell types by community detection
Elapsed time is 82.076697 seconds.
Cell-cell graph contains 25 cell types after merging tiny cell clusters
Cell-cell graph contains 3 cell types after merging
Number of useful pathways is 2
```

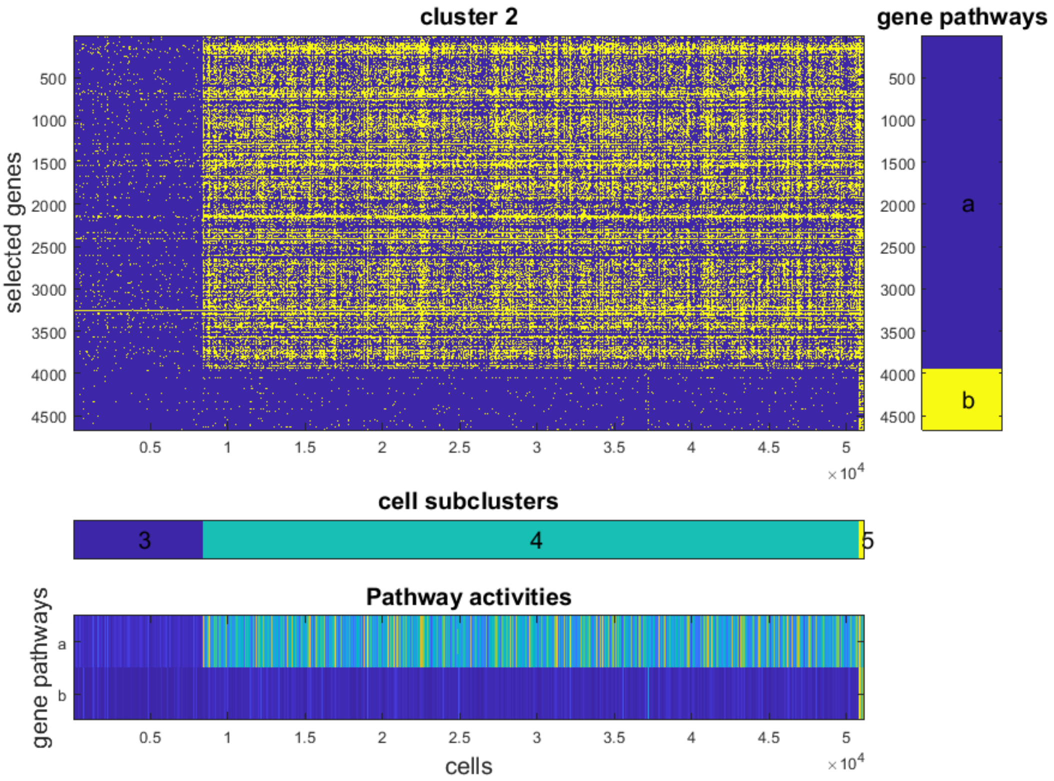


```
Remaining clusters to partition 3
Processing cluster 3 now ...
Processing data subset with 22327 genes and 8408 cells:
Remove genes detected in <100 cells. Remaining 6357 genes.  Elapsed time is 0.702884 seconds.
Iterate 10 random permutations for gene-gene similarity threshold ...   10 Elapsed time is 116.724373 seconds.
Compute gene-gene similarity ... Elapsed time is 6.837961 seconds.
Create gene-gene graph for clustering genes ...
Writing graph into file ... 100%Elapsed time is 0.908771 seconds.
Running ModularityOptimizer for clustering ...Elapsed time is 2.212496 seconds.
Gene-gene graph contains 8 pathways, 3655 genes in total
Elapsed time is 2.413786 seconds.
Create cell-cell graph for clustering cells ...
Writing graph into file ... 100%Elapsed time is 1.068214 seconds.
Running ModularityOptimizer for clustering ...Elapsed time is 6.472900 seconds.
Cell-cell graph contains 20 cell types by community detection
Elapsed time is 6.653487 seconds.
Cell-cell graph contains 20 cell types after merging tiny cell clusters
Cell-cell graph contains 2 cell types after merging
Number of useful pathways is 1
```

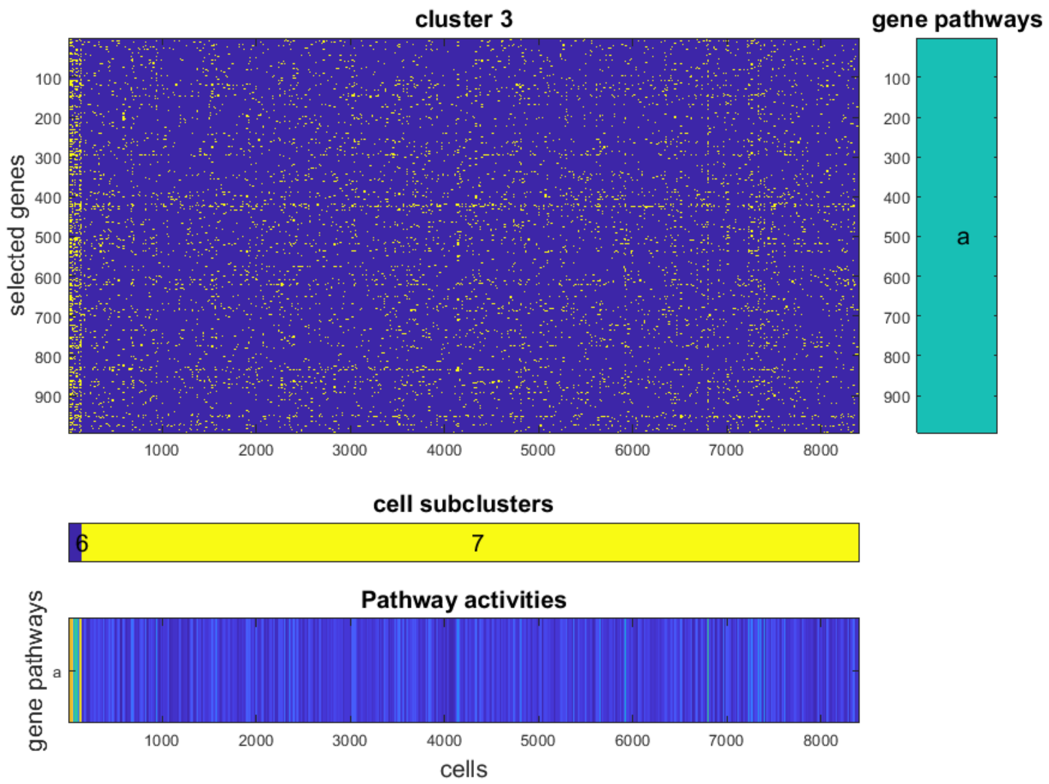


```
Remaining clusters to partition 4
Processing cluster 4 now ...
Processing data subset with 22327 genes and 42350 cells:
Remove genes detected in <100 cells. Remaining 18065 genes.  Elapsed time is 4.958958 seconds.
Iterate 10 random permutations for gene-gene similarity threshold ...   10 Elapsed time is 3603.244607 seconds.
Compute gene-gene similarity ... Elapsed time is 198.303449 seconds.
Create gene-gene graph for clustering genes ...
Writing graph into file ... 100%Elapsed time is 407.155793 seconds.
Running ModularityOptimizer for clustering ...Elapsed time is 1294.333687 seconds.
Gene-gene graph contains 6 pathways, 17989 genes in total
Elapsed time is 1295.435055 seconds.
Create cell-cell graph for clustering cells ...
Writing graph into file ... 100%Elapsed time is 5.139699 seconds.
Running ModularityOptimizer for clustering ...Elapsed time is 60.181225 seconds.
Cell-cell graph contains 28 cell types by community detection
Elapsed time is 61.095585 seconds.
Cell-cell graph contains 14 cell types after merging tiny cell clusters
Cell-cell graph contains 2 cell types after merging
Number of useful pathways is 1
```

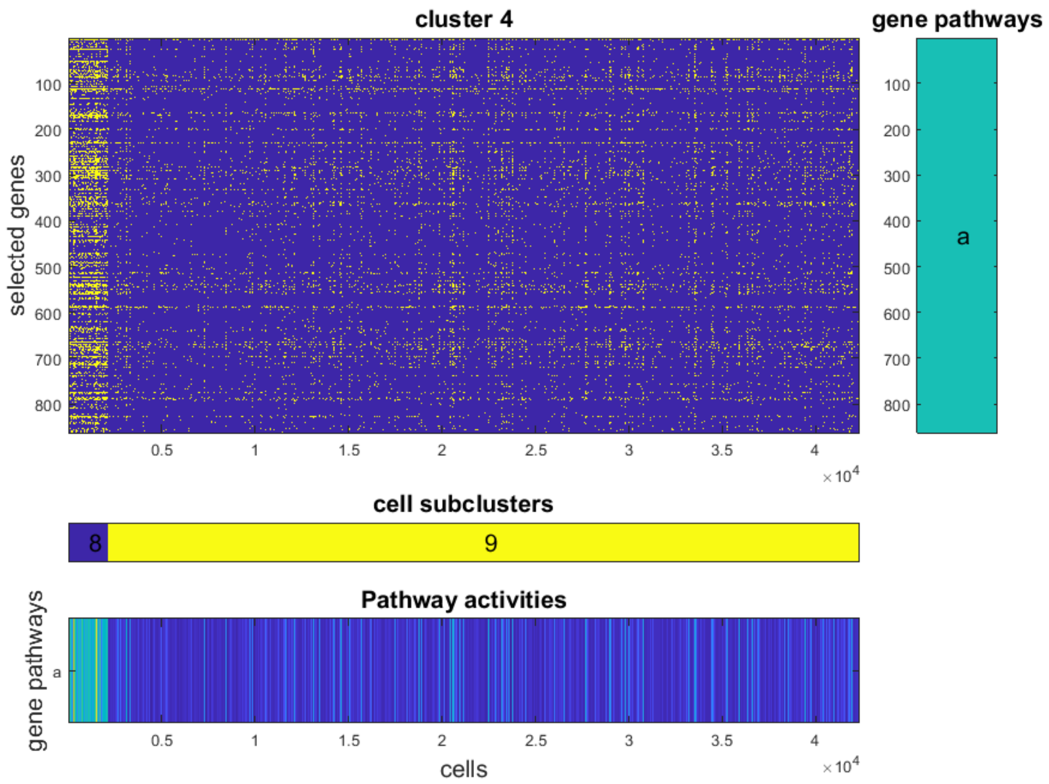


```
Remaining clusters to partition 5
Processing cluster 5 now ...
Processing data subset with 22327 genes and 426 cells:
Remove genes detected in <100 cells. Remaining 7146 genes.  Elapsed time is 0.036417 seconds.
Iterate 10 random permutations for gene-gene similarity threshold ...   10 Elapsed time is 38.627849 seconds.
Compute gene-gene similarity ... Elapsed time is 2.862895 seconds.
Create gene-gene graph for clustering genes ...
Writing graph into file ... 100%Elapsed time is 142.440052 seconds.
Running ModularityOptimizer for clustering ...Elapsed time is 218.005664 seconds.
Gene-gene graph contains 2 pathways, 7122 genes in total
Elapsed time is 218.298295 seconds.
Create cell-cell graph for clustering cells ...
Writing graph into file ... 100%Elapsed time is 0.047828 seconds.
Running ModularityOptimizer for clustering ...Elapsed time is 0.392261 seconds.
Cell-cell graph contains 9 cell types by community detection
Elapsed time is 0.404605 seconds.
Cell-cell graph contains 2 cell types after merging tiny cell clusters
Cell-cell graph contains 2 cell types after merging
Number of useful pathways is 2
```

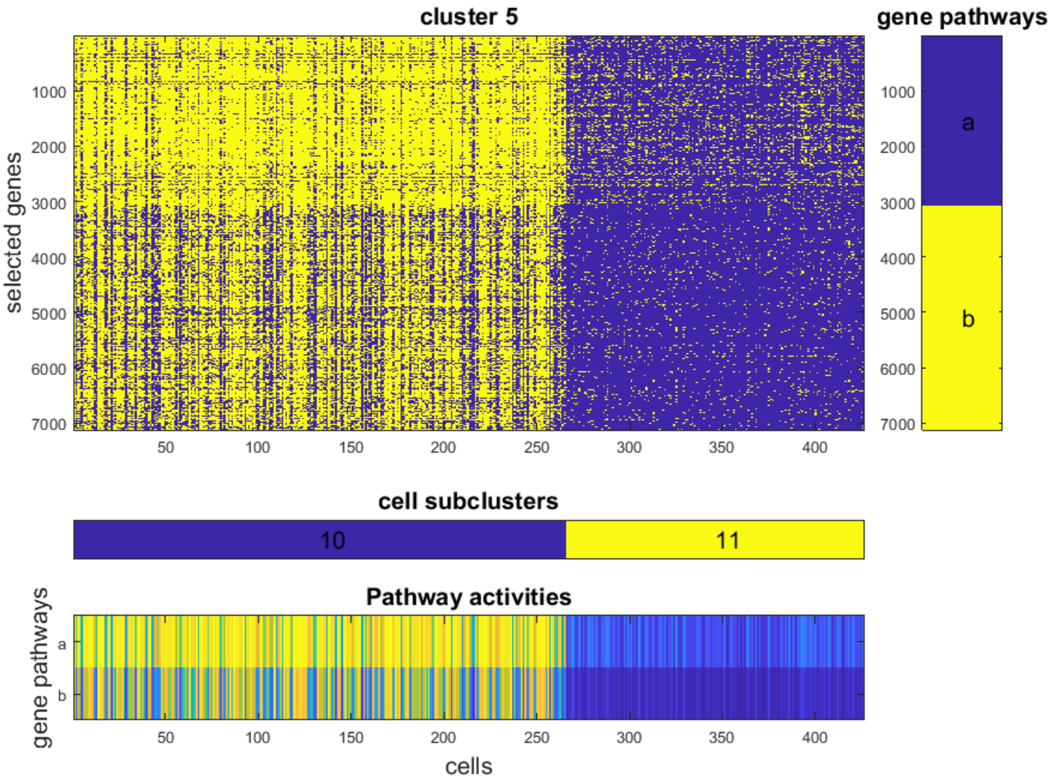


```
Remaining clusters to partition 6
Processing cluster 6 now ...
Processing data subset with 22327 genes and 142 cells:
Remove genes detected in <100 cells. Remaining 0 genes.  Elapsed time is 0.008157 seconds.

Remaining clusters to partition 5
Processing cluster 7 now ...
Processing data subset with 22327 genes and 8266 cells:
Remove genes detected in <100 cells. Remaining 6090 genes.  Elapsed time is 0.744495 seconds.
Iterate 10 random permutations for gene-gene similarity threshold ...   10 Elapsed time is 104.832373 seconds.
Compute gene-gene similarity ... Elapsed time is 6.327348 seconds.
Create gene-gene graph for clustering genes ...
Writing graph into file ... 100%Elapsed time is 0.696901 seconds.
Running ModularityOptimizer for clustering ...Elapsed time is 1.670505 seconds.
Gene-gene graph contains 9 pathways, 3218 genes in total
Elapsed time is 1.855794 seconds.
Create cell-cell graph for clustering cells ...
Writing graph into file ... 100%Elapsed time is 1.041817 seconds.
Running ModularityOptimizer for clustering ...Elapsed time is 6.914371 seconds.
Cell-cell graph contains 17 cell types by community detection
Elapsed time is 7.085561 seconds.
Cell-cell graph contains 17 cell types after merging tiny cell clusters
Cell-cell graph contains 2 cell types after merging
Number of useful pathways is 1
```

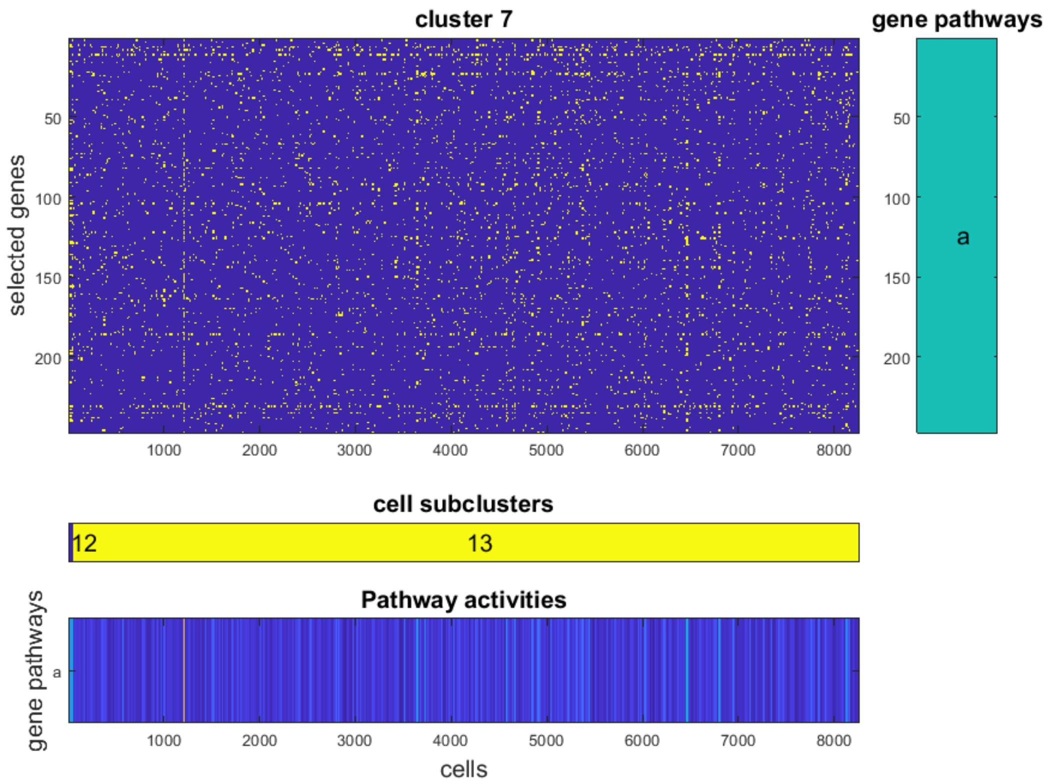


```
Remaining clusters to partition 6
Processing cluster 8 now ...
Processing data subset with 22327 genes and 2120 cells:
Remove genes detected in <100 cells. Remaining 11267 genes.  Elapsed time is 0.221084 seconds.
Iterate 10 random permutations for gene-gene similarity threshold ...   10 Elapsed time is 139.705758 seconds.
Compute gene-gene similarity ... Elapsed time is 10.194171 seconds.
Create gene-gene graph for clustering genes ...
Writing graph into file ... 100%Elapsed time is 11.172821 seconds.
Running ModularityOptimizer for clustering ...Elapsed time is 29.996206 seconds.
Gene-gene graph contains 5 pathways, 9919 genes in total
Elapsed time is 30.449675 seconds.
Create cell-cell graph for clustering cells ...
Writing graph into file ... 100%Elapsed time is 0.250161 seconds.
Running ModularityOptimizer for clustering ...Elapsed time is 1.231023 seconds.
Cell-cell graph contains 14 cell types by community detection
Elapsed time is 1.280583 seconds.
Cell-cell graph contains 14 cell types after merging tiny cell clusters
creating a total of 13 edges ...     13
Cell-cell graph contains 2 cell types after merging
Number of useful pathways is 1
```

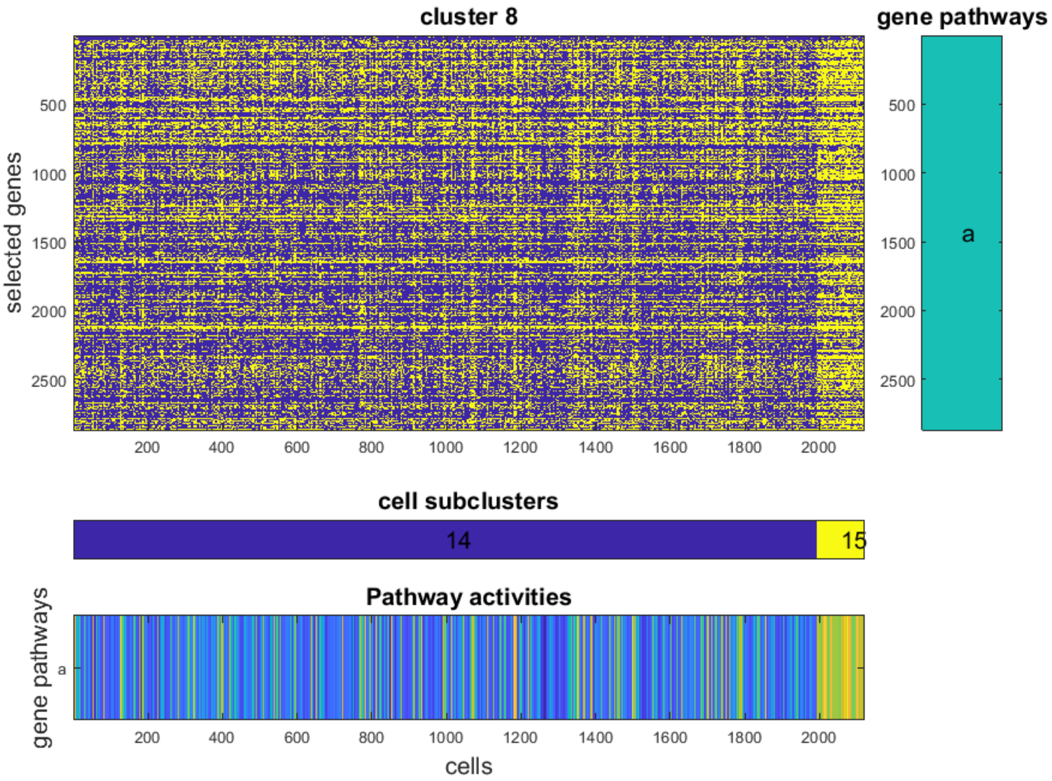


```
Remaining clusters to partition 7
Processing cluster 9 now ...
Processing data subset with 22327 genes and 40230 cells:
Remove genes detected in <100 cells. Remaining 17919 genes.  Elapsed time is 4.830486 seconds.
Iterate 10 random permutations for gene-gene similarity threshold ...   10 Elapsed time is 3113.513049 seconds.
Compute gene-gene similarity ... Elapsed time is 162.923699 seconds.
Create gene-gene graph for clustering genes ...
Writing graph into file ... 100%Elapsed time is 390.871630 seconds.
Running ModularityOptimizer for clustering ...Elapsed time is 1261.404732 seconds.
Gene-gene graph contains 5 pathways, 17843 genes in total
Elapsed time is 1262.478106 seconds.
Create cell-cell graph for clustering cells ...
Writing graph into file ... 100%Elapsed time is 4.671637 seconds.
Running ModularityOptimizer for clustering ...Elapsed time is 47.278873 seconds.
Cell-cell graph contains 28 cell types by community detection
Elapsed time is 48.099991 seconds.
Cell-cell graph contains 12 cell types after merging tiny cell clusters
Cell-cell graph contains 2 cell types after merging
Number of useful pathways is 1
```

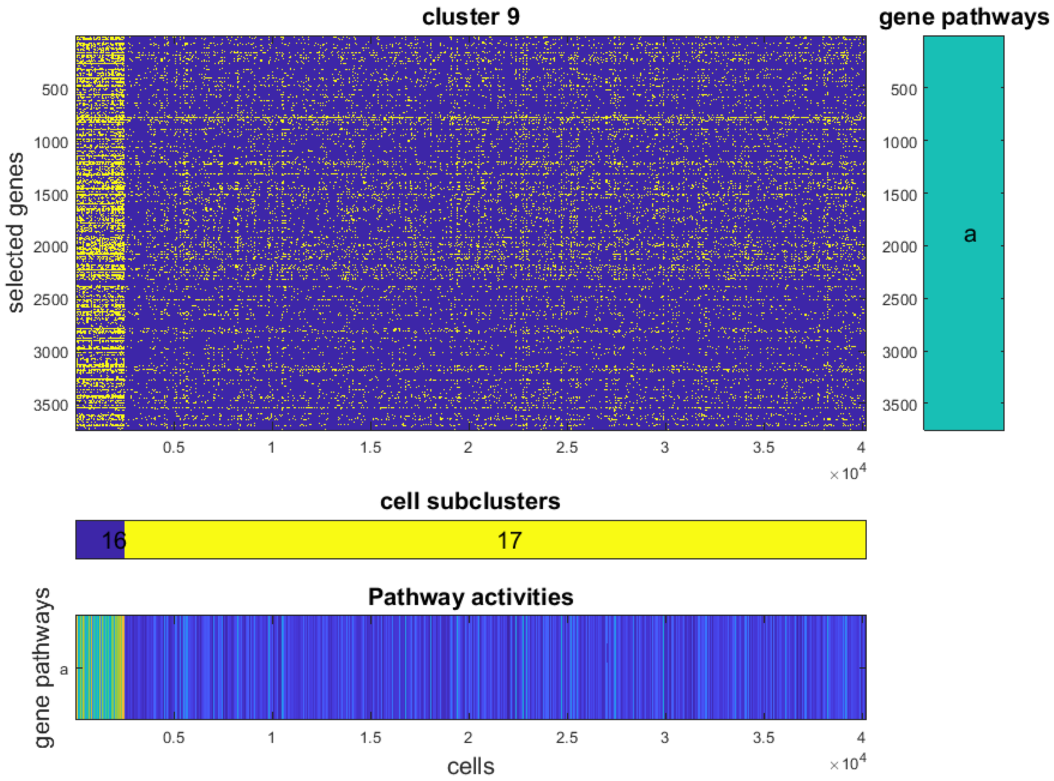


```
Remaining clusters to partition 8
Processing cluster 10 now ...
Processing data subset with 22327 genes and 265 cells:
Remove genes detected in <100 cells. Remaining 2494 genes.  Elapsed time is 0.019721 seconds.
Iterate 10 random permutations for gene-gene similarity threshold ...   10 Elapsed time is 4.434775 seconds.
Compute gene-gene similarity ... Elapsed time is 0.334278 seconds.
Create gene-gene graph for clustering genes ...
Writing graph into file ... 100%Elapsed time is 5.207173 seconds.
Running ModularityOptimizer for clustering ...Elapsed time is 22.670712 seconds.
Gene-gene graph contains 3 pathways, 2459 genes in total
Elapsed time is 22.738318 seconds.
Create cell-cell graph for clustering cells ...
Writing graph into file ... 100%Elapsed time is 0.036560 seconds.
Running ModularityOptimizer for clustering ...Elapsed time is 0.322739 seconds.
Cell-cell graph contains 6 cell types by community detection
Elapsed time is 0.331575 seconds.
Cell-cell graph contains 5 cell types after merging tiny cell clusters
Cell-cell graph contains 2 cell types after merging
Number of useful pathways is 3
```

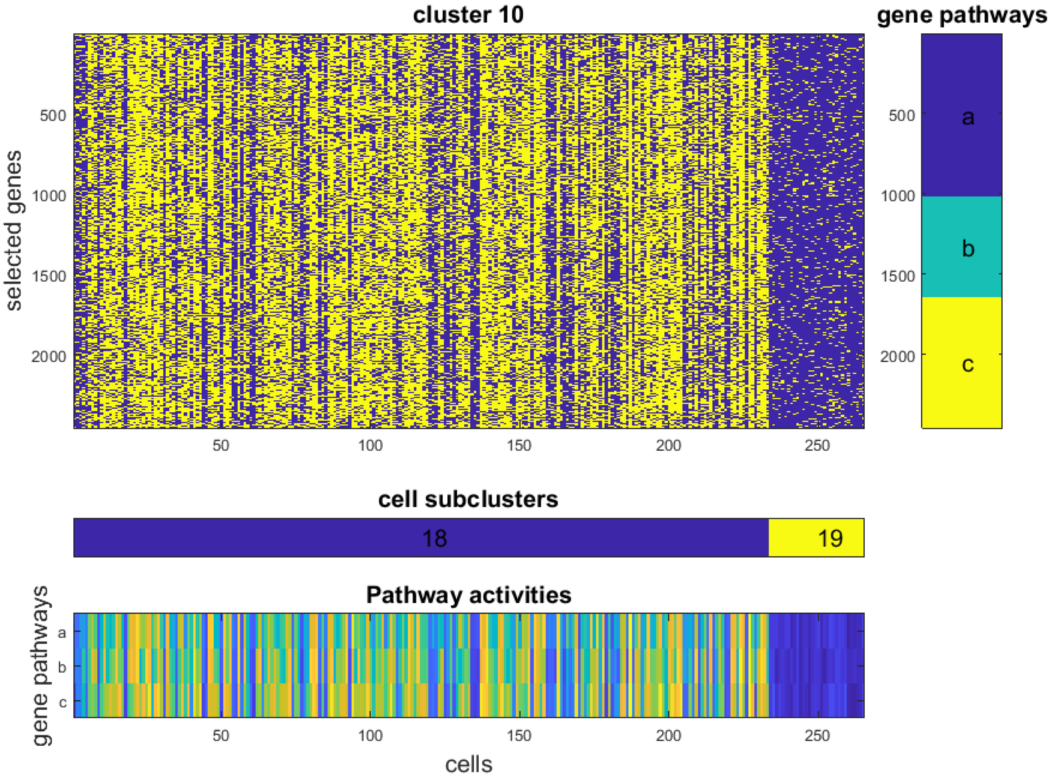


```
Remaining clusters to partition 9
Processing cluster 11 now ...
Processing data subset with 22327 genes and 161 cells:
Remove genes detected in <100 cells. Remaining 0 genes.  Elapsed time is 0.011722 seconds.

Remaining clusters to partition 8
Processing cluster 12 now ...
Processing data subset with 22327 genes and 53 cells:
Remove genes detected in <100 cells. Remaining 0 genes.  Elapsed time is 0.004152 seconds.

Remaining clusters to partition 7
Processing cluster 13 now ...
Processing data subset with 22327 genes and 8213 cells:
Remove genes detected in <100 cells. Remaining 6021 genes.  Elapsed time is 0.724796 seconds.
Iterate 10 random permutations for gene-gene similarity threshold ...   10 Elapsed time is 94.891433 seconds.
Compute gene-gene similarity ... Elapsed time is 5.490349 seconds.
Create gene-gene graph for clustering genes ...
Writing graph into file ... 100%Elapsed time is 0.584768 seconds.
Running ModularityOptimizer for clustering ...Elapsed time is 1.770902 seconds.
Gene-gene graph contains 8 pathways, 3015 genes in total
Elapsed time is 1.957822 seconds.
Create cell-cell graph for clustering cells ...
Writing graph into file ... 100%Elapsed time is 0.995114 seconds.
Running ModularityOptimizer for clustering ...Elapsed time is 6.264500 seconds.
Cell-cell graph contains 16 cell types by community detection
Elapsed time is 6.432353 seconds.
Cell-cell graph contains 14 cell types after merging tiny cell clusters
creating a total of 13 edges ...     13
Cell-cell graph contains 2 cell types after merging
Number of useful pathways is 1
```

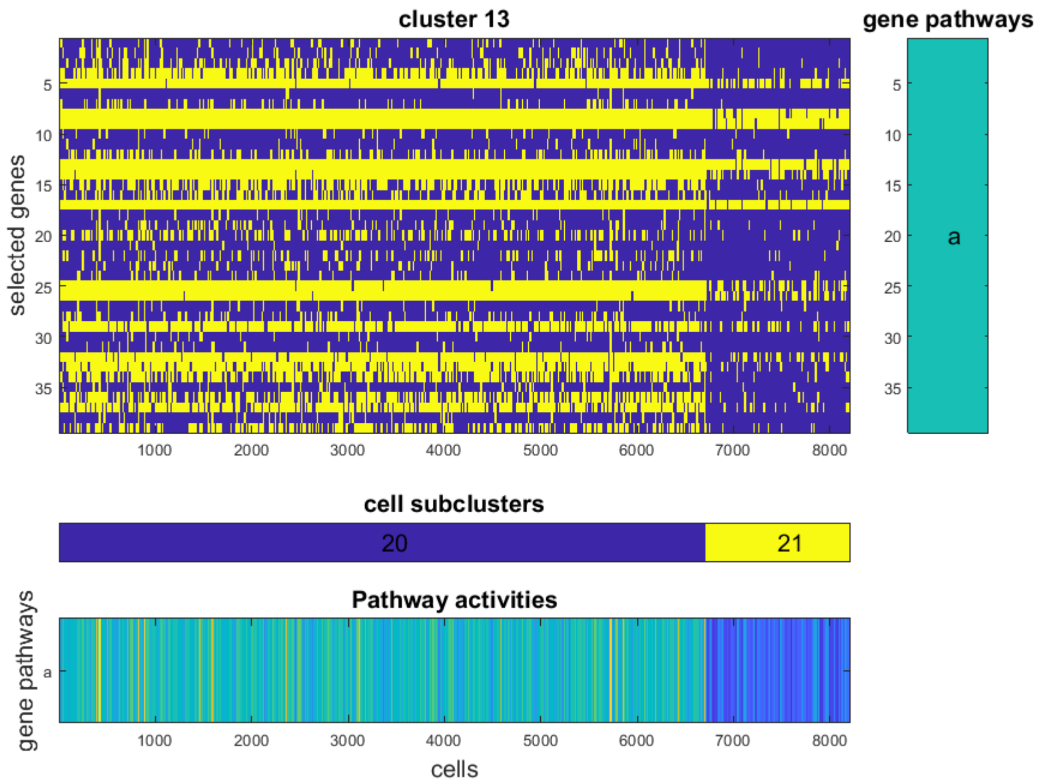


```
Remaining clusters to partition 8
Processing cluster 14 now ...
Processing data subset with 22327 genes and 1993 cells:
Remove genes detected in <100 cells. Remaining 11137 genes.  Elapsed time is 0.208682 seconds.
Iterate 10 random permutations for gene-gene similarity threshold ...   10 Elapsed time is 126.063440 seconds.
Compute gene-gene similarity ... Elapsed time is 9.414428 seconds.
Create gene-gene graph for clustering genes ...
Writing graph into file ... 100%Elapsed time is 8.699888 seconds.
Running ModularityOptimizer for clustering ...Elapsed time is 25.947468 seconds.
Gene-gene graph contains 5 pathways, 9799 genes in total
Elapsed time is 26.372335 seconds.
Create cell-cell graph for clustering cells ...
Writing graph into file ... 100%Elapsed time is 0.238028 seconds.
Running ModularityOptimizer for clustering ...Elapsed time is 1.128222 seconds.
Cell-cell graph contains 13 cell types by community detection
Elapsed time is 1.172460 seconds.
Cell-cell graph contains 13 cell types after merging tiny cell clusters
creating a total of 12 edges ...     12
Cell-cell graph contains 1 cell types after merging

Remaining clusters to partition 7
Processing cluster 15 now ...
Processing data subset with 22327 genes and 127 cells:
Remove genes detected in <100 cells. Remaining 0 genes.  Elapsed time is 0.008776 seconds.

Remaining clusters to partition 6
Processing cluster 16 now ...
Processing data subset with 22327 genes and 2521 cells:
Remove genes detected in <100 cells. Remaining 11706 genes.  Elapsed time is 0.242025 seconds.
Iterate 10 random permutations for gene-gene similarity threshold ...   10 Elapsed time is 154.192012 seconds.
Compute gene-gene similarity ... Elapsed time is 10.936685 seconds.
Create gene-gene graph for clustering genes ...
Writing graph into file ... 100%Elapsed time is 79.473229 seconds.
Running ModularityOptimizer for clustering ...Elapsed time is 238.572325 seconds.
Gene-gene graph contains 5 pathways, 11397 genes in total
Elapsed time is 239.056205 seconds.
Create cell-cell graph for clustering cells ...
Writing graph into file ... 100%Elapsed time is 0.296260 seconds.
Running ModularityOptimizer for clustering ...Elapsed time is 1.342989 seconds.
Cell-cell graph contains 13 cell types by community detection
Elapsed time is 1.396278 seconds.
Cell-cell graph contains 10 cell types after merging tiny cell clusters
creating a total of 9 edges ...      9
Cell-cell graph contains 2 cell types after merging
Number of useful pathways is 1
```

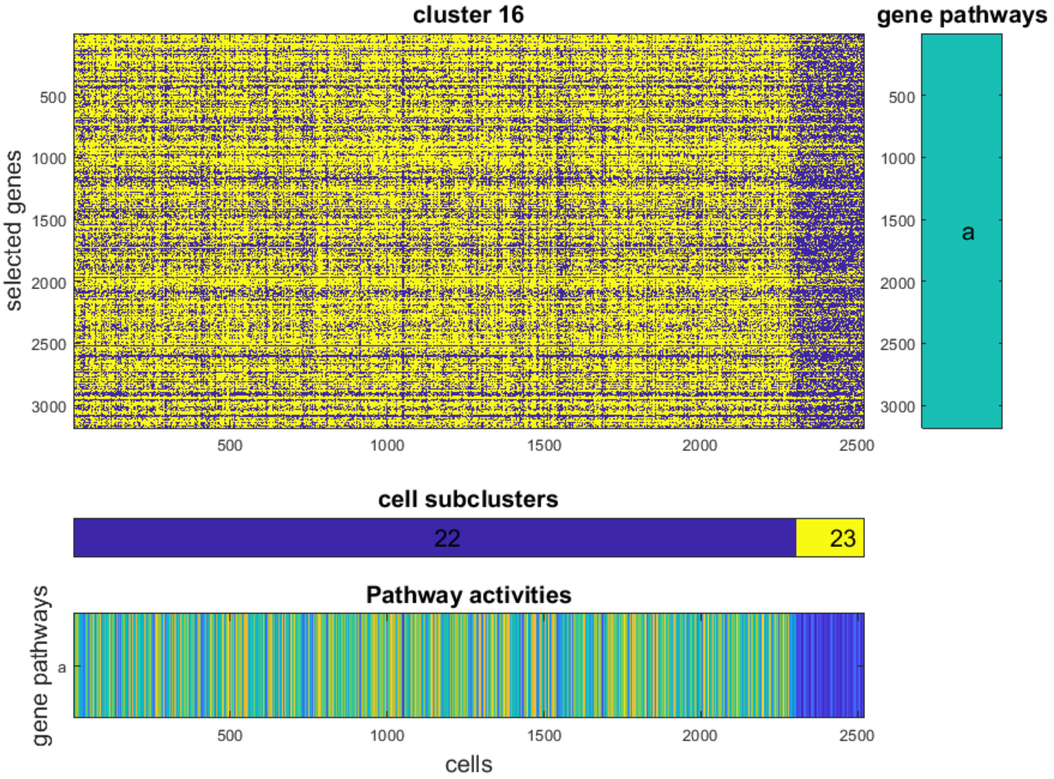


```
Remaining clusters to partition 7
Processing cluster 17 now ...
Processing data subset with 22327 genes and 37709 cells:
Remove genes detected in <100 cells. Remaining 17694 genes.  Elapsed time is 4.168171 seconds.
Iterate 10 random permutations for gene-gene similarity threshold ...   10 Elapsed time is 2579.136187 seconds.
Compute gene-gene similarity ... Elapsed time is 144.253655 seconds.
Create gene-gene graph for clustering genes ...
Writing graph into file ... 100%Elapsed time is 323.050478 seconds.
Running ModularityOptimizer for clustering ...Elapsed time is 1015.165593 seconds.
Gene-gene graph contains 5 pathways, 17614 genes in total
Elapsed time is 1016.152828 seconds.
Create cell-cell graph for clustering cells ...
Writing graph into file ... 100%Elapsed time is 4.396675 seconds.
Running ModularityOptimizer for clustering ...Elapsed time is 45.740877 seconds.
Cell-cell graph contains 26 cell types by community detection
Elapsed time is 46.508122 seconds.
Cell-cell graph contains 15 cell types after merging tiny cell clusters
creating a total of 14 edges ...     14
Cell-cell graph contains 2 cell types after merging
Number of useful pathways is 1
```

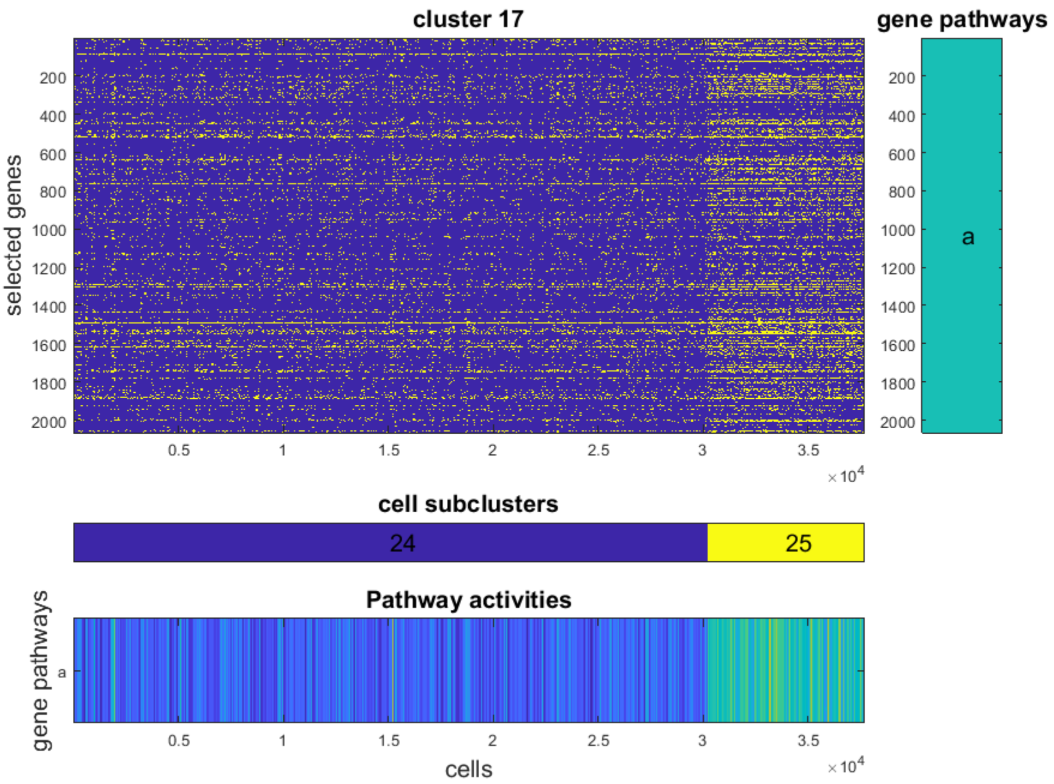


```
Remaining clusters to partition 8
Processing cluster 18 now ...
Processing data subset with 22327 genes and 233 cells:
Remove genes detected in <100 cells. Remaining 1252 genes.  Elapsed time is 0.015854 seconds.
Iterate 10 random permutations for gene-gene similarity threshold ...   10 Elapsed time is 1.175376 seconds.
Compute gene-gene similarity ... Elapsed time is 0.087432 seconds.
Create gene-gene graph for clustering genes ...
Writing graph into file ... 100%Elapsed time is 0.147192 seconds.
Running ModularityOptimizer for clustering ...Elapsed time is 1.121497 seconds.
Gene-gene graph contains 7 pathways, 1168 genes in total
Elapsed time is 1.155886 seconds.
Create cell-cell graph for clustering cells ...
Writing graph into file ... 100%Elapsed time is 0.028643 seconds.
Running ModularityOptimizer for clustering ...Elapsed time is 0.293153 seconds.
Cell-cell graph contains 5 cell types by community detection
Elapsed time is 0.300937 seconds.
Cell-cell graph contains 5 cell types after merging tiny cell clusters
Cell-cell graph contains 2 cell types after merging
Number of useful pathways is 5
```

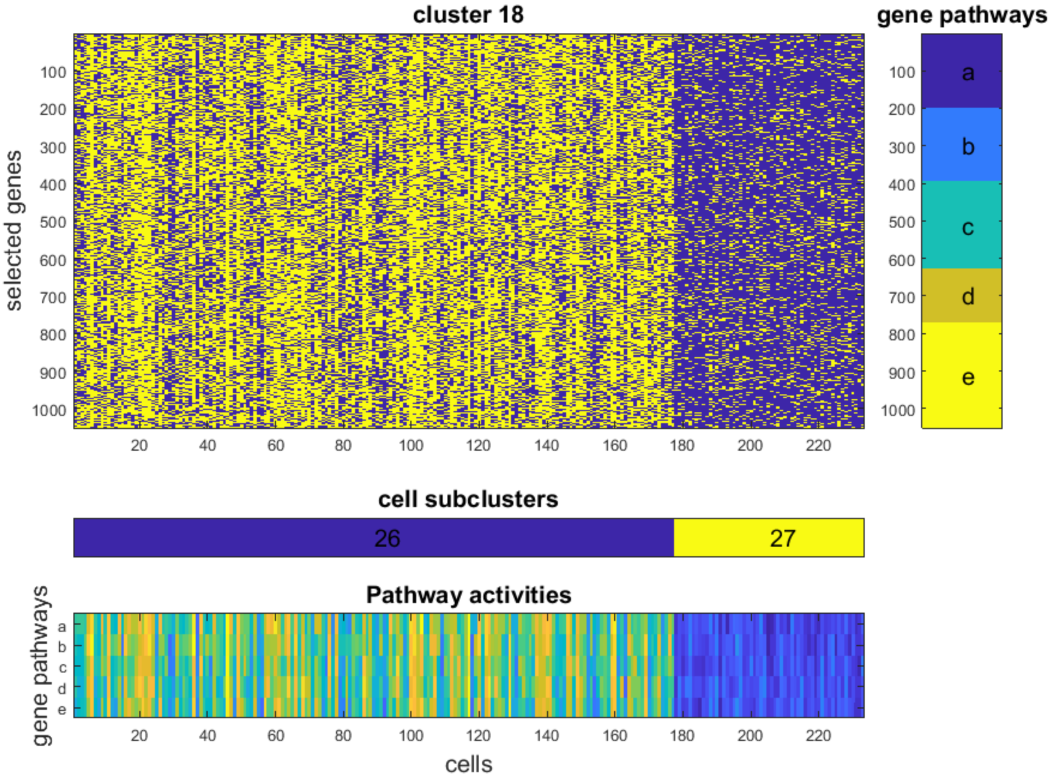


```
Remaining clusters to partition 9
Processing cluster 19 now ...
Processing data subset with 22327 genes and 32 cells:
Remove genes detected in <100 cells. Remaining 0 genes.  Elapsed time is 0.002421 seconds.

Remaining clusters to partition 8
Processing cluster 20 now ...
Processing data subset with 22327 genes and 6698 cells:
Remove genes detected in <100 cells. Remaining 5193 genes.  Elapsed time is 0.577324 seconds.
Iterate 10 random permutations for gene-gene similarity threshold ...   10 Elapsed time is 64.516148 seconds.
Compute gene-gene similarity ... Elapsed time is 3.654834 seconds.
Create gene-gene graph for clustering genes ...
Writing graph into file ... 100%Elapsed time is 0.490005 seconds.
Running ModularityOptimizer for clustering ...Elapsed time is 1.550704 seconds.
Gene-gene graph contains 7 pathways, 2709 genes in total
Elapsed time is 1.702953 seconds.
Create cell-cell graph for clustering cells ...
Writing graph into file ... 100%Elapsed time is 0.827408 seconds.
Running ModularityOptimizer for clustering ...Elapsed time is 5.056950 seconds.
Cell-cell graph contains 14 cell types by community detection
Elapsed time is 5.197317 seconds.
Cell-cell graph contains 10 cell types after merging tiny cell clusters
creating a total of 9 edges ...      9
Cell-cell graph contains 2 cell types after merging
Number of useful pathways is 1
```

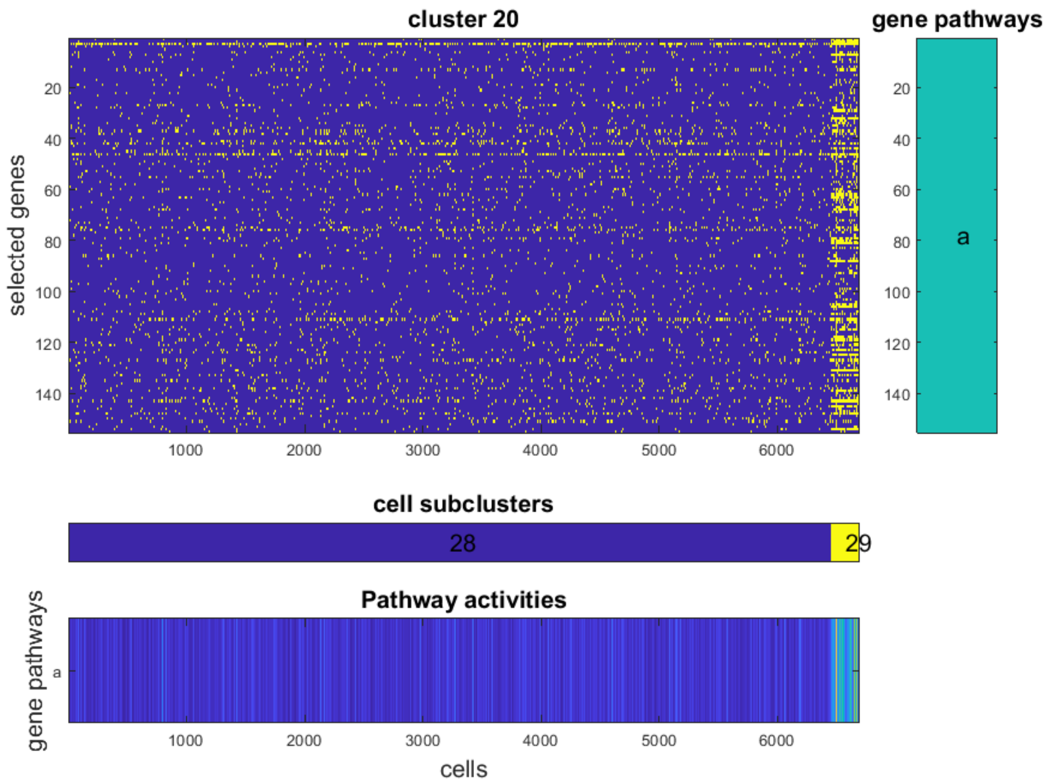


```
Remaining clusters to partition 9
Processing cluster 21 now ...
Processing data subset with 22327 genes and 1515 cells:
Remove genes detected in <100 cells. Remaining 667 genes.  Elapsed time is 0.109761 seconds.
Iterate 10 random permutations for gene-gene similarity threshold ...   10 Elapsed time is 1.142926 seconds.
Compute gene-gene similarity ... Elapsed time is 0.043068 seconds.
Create gene-gene graph for clustering genes ...
Writing graph into file ... 100%Elapsed time is 0.013745 seconds.
Running ModularityOptimizer for clustering ...Elapsed time is 0.316513 seconds.
Gene-gene graph contains 5 pathways, 288 genes in total
Elapsed time is 0.334536 seconds.
Create cell-cell graph for clustering cells ...
Writing graph into file ... 100%Elapsed time is 0.188811 seconds.
Running ModularityOptimizer for clustering ...Elapsed time is 1.117013 seconds.
Cell-cell graph contains 9 cell types by community detection
Elapsed time is 1.163917 seconds.
Cell-cell graph contains 9 cell types after merging tiny cell clusters
creating a total of 8 edges ...      8
Cell-cell graph contains 1 cell types after merging

Remaining clusters to partition 8
Processing cluster 22 now ...
Processing data subset with 22327 genes and 2303 cells:
Remove genes detected in <100 cells. Remaining 11468 genes.  Elapsed time is 0.258714 seconds.
Iterate 10 random permutations for gene-gene similarity threshold ...   10 Elapsed time is 141.526589 seconds.
Compute gene-gene similarity ... Elapsed time is 10.345258 seconds.
Create gene-gene graph for clustering genes ...
Writing graph into file ... 100%Elapsed time is 61.849998 seconds.
Running ModularityOptimizer for clustering ...Elapsed time is 177.983452 seconds.
Gene-gene graph contains 5 pathways, 11148 genes in total
Elapsed time is 178.456067 seconds.
Create cell-cell graph for clustering cells ...
Writing graph into file ... 100%Elapsed time is 0.259931 seconds.
Running ModularityOptimizer for clustering ...Elapsed time is 1.230795 seconds.
Cell-cell graph contains 14 cell types by community detection
Elapsed time is 1.282077 seconds.
Cell-cell graph contains 12 cell types after merging tiny cell clusters
creating a total of 11 edges ...     11
Cell-cell graph contains 2 cell types after merging
Number of useful pathways is 1
```

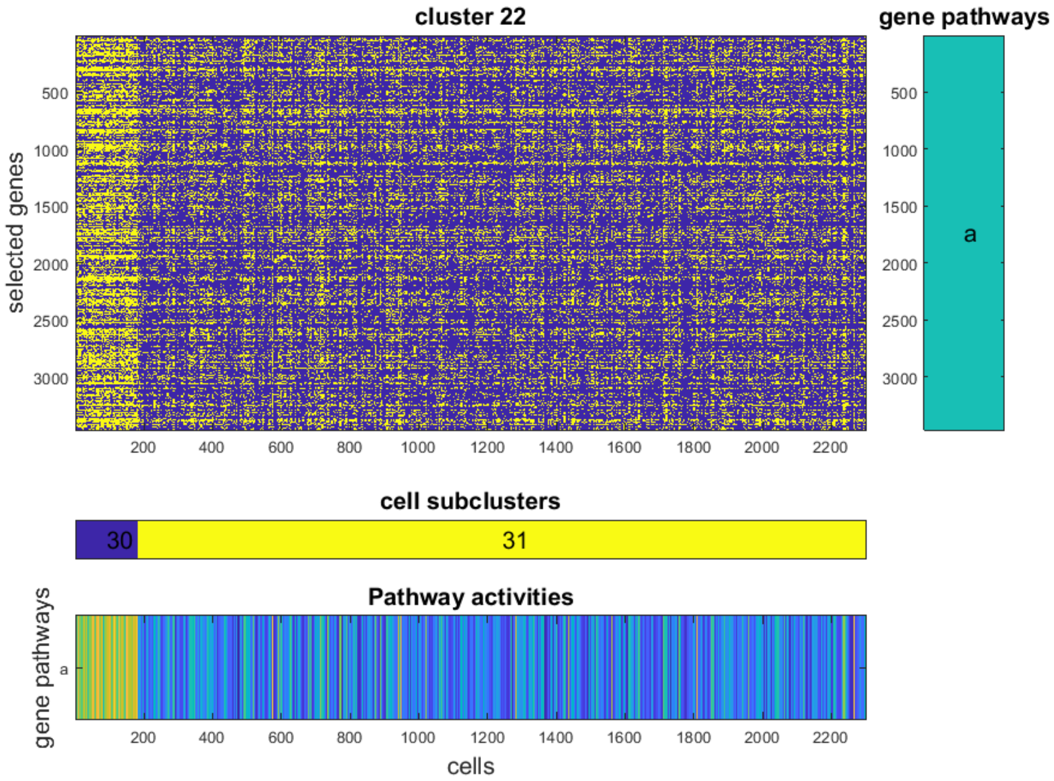


```
Remaining clusters to partition 9
Processing cluster 23 now ...
Processing data subset with 22327 genes and 218 cells:
Remove genes detected in <100 cells. Remaining 607 genes.  Elapsed time is 0.014167 seconds.
Iterate 10 random permutations for gene-gene similarity threshold ...   10 Elapsed time is 0.305427 seconds.
Compute gene-gene similarity ... Elapsed time is 0.020977 seconds.
Create gene-gene graph for clustering genes ...
Writing graph into file ... 101%Elapsed time is 0.004741 seconds.
Running ModularityOptimizer for clustering ...Elapsed time is 0.239324 seconds.
Gene-gene graph contains 0 pathways, 0 genes in total
Elapsed time is 0.327220 seconds.

Remaining clusters to partition 8
Processing cluster 24 now ...
Processing data subset with 22327 genes and 30195 cells:
Remove genes detected in <100 cells. Remaining 17411 genes.  Elapsed time is 3.424119 seconds.
Iterate 10 random permutations for gene-gene similarity threshold ...   10 Elapsed time is 2036.258670 seconds.
Compute gene-gene similarity ... Elapsed time is 118.012221 seconds.
Create gene-gene graph for clustering genes ...
Writing graph into file ... 100%Elapsed time is 337.536549 seconds.
Running ModularityOptimizer for clustering ...Elapsed time is 880.206026 seconds.
Gene-gene graph contains 4 pathways, 17344 genes in total
Elapsed time is 881.185693 seconds.
Create cell-cell graph for clustering cells ...
Writing graph into file ... 100%Elapsed time is 3.459113 seconds.
Running ModularityOptimizer for clustering ...Elapsed time is 28.575325 seconds.
Cell-cell graph contains 31 cell types by community detection
Elapsed time is 29.223923 seconds.
Cell-cell graph contains 20 cell types after merging tiny cell clusters
creating a total of 19 edges ...     19
Cell-cell graph contains 2 cell types after merging
Number of useful pathways is 1
```

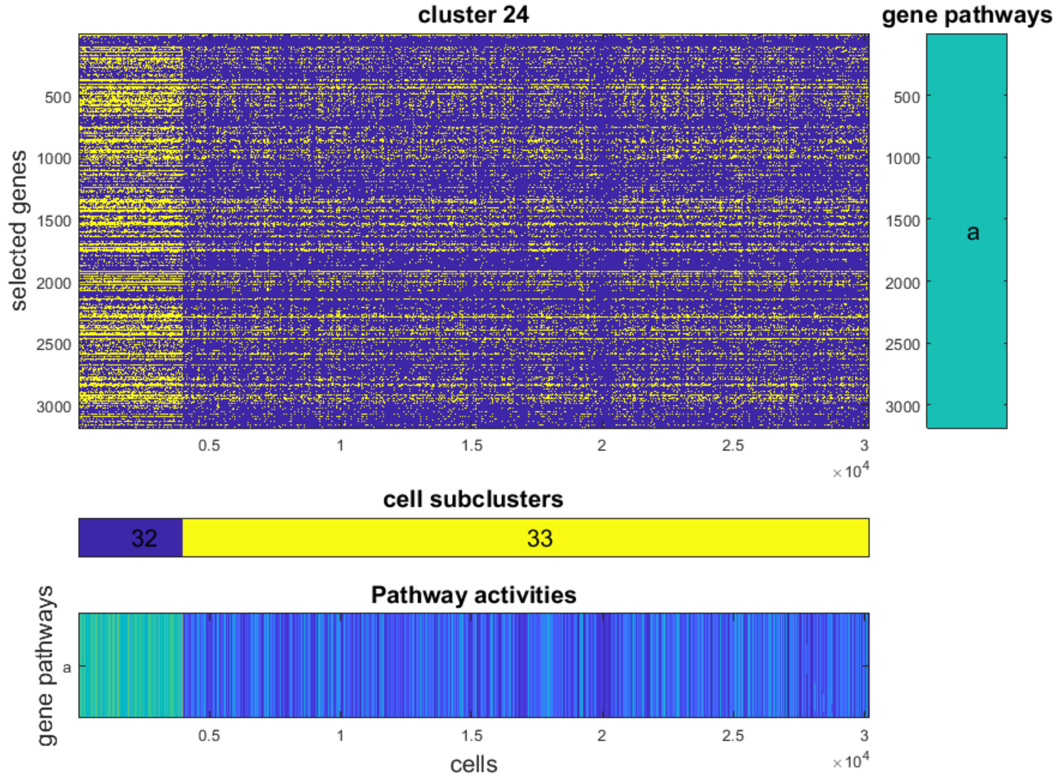


```
Remaining clusters to partition 9
Processing cluster 25 now ...
Processing data subset with 22327 genes and 7514 cells:
Remove genes detected in <100 cells. Remaining 12511 genes.  Elapsed time is 0.854637 seconds.
Iterate 10 random permutations for gene-gene similarity threshold ...   10 Elapsed time is 316.481694 seconds.
Compute gene-gene similarity ... Elapsed time is 22.646260 seconds.
Create gene-gene graph for clustering genes ...
Writing graph into file ... 100%Elapsed time is 20.092826 seconds.
Running ModularityOptimizer for clustering ...Elapsed time is 62.588693 seconds.
Gene-gene graph contains 6 pathways, 11040 genes in total
Elapsed time is 63.101439 seconds.
Create cell-cell graph for clustering cells ...
Writing graph into file ... 100%Elapsed time is 0.892336 seconds.
Running ModularityOptimizer for clustering ...Elapsed time is 5.831501 seconds.
Cell-cell graph contains 19 cell types by community detection
Elapsed time is 5.997231 seconds.
Cell-cell graph contains 16 cell types after merging tiny cell clusters
Cell-cell graph contains 2 cell types after merging
Number of useful pathways is 1
```

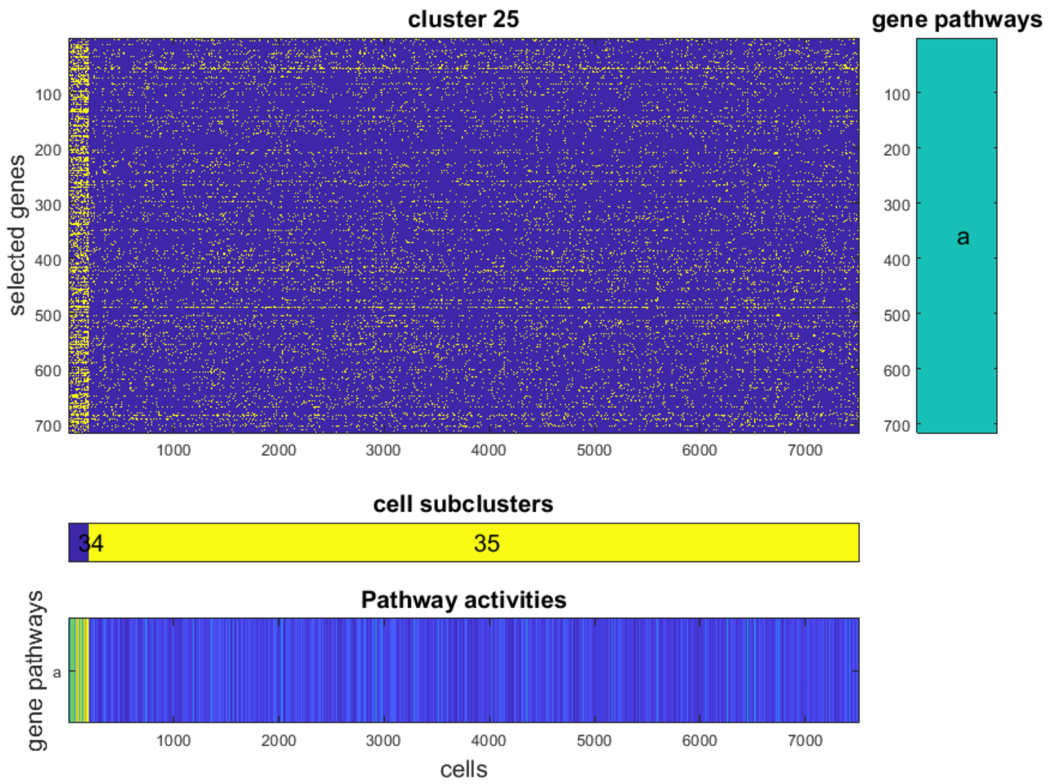


```
Remaining clusters to partition 10
Processing cluster 26 now ...
Processing data subset with 22327 genes and 177 cells:
Remove genes detected in <100 cells. Remaining 0 genes.  Elapsed time is 0.012277 seconds.

Remaining clusters to partition 9
Processing cluster 27 now ...
Processing data subset with 22327 genes and 56 cells:
Remove genes detected in <100 cells. Remaining 0 genes.  Elapsed time is 0.003513 seconds.

Remaining clusters to partition 8
Processing cluster 28 now ...
Processing data subset with 22327 genes and 6446 cells:
Remove genes detected in <100 cells. Remaining 4991 genes.  Elapsed time is 0.523225 seconds.
Iterate 10 random permutations for gene-gene similarity threshold ...   10 Elapsed time is 58.997871 seconds.
Compute gene-gene similarity ... Elapsed time is 3.293949 seconds.
Create gene-gene graph for clustering genes ...
Writing graph into file ... 100%Elapsed time is 0.450493 seconds.
Running ModularityOptimizer for clustering ...Elapsed time is 1.335523 seconds.
Gene-gene graph contains 6 pathways, 2426 genes in total
Elapsed time is 1.480578 seconds.
Create cell-cell graph for clustering cells ...
Writing graph into file ... 100%Elapsed time is 0.755376 seconds.
Running ModularityOptimizer for clustering ...Elapsed time is 4.402231 seconds.
Cell-cell graph contains 16 cell types by community detection
Elapsed time is 4.548639 seconds.
Cell-cell graph contains 15 cell types after merging tiny cell clusters
creating a total of 14 edges ...     14
Cell-cell graph contains 2 cell types after merging
Number of useful pathways is 1
```

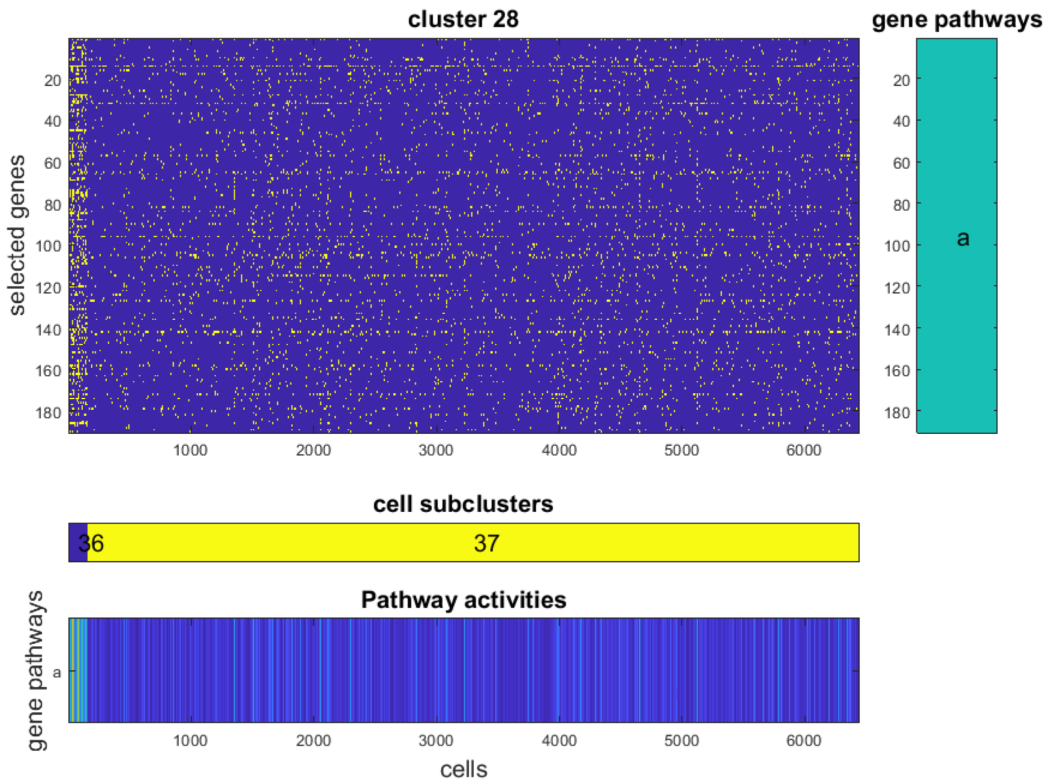


```
Remaining clusters to partition 9
Processing cluster 29 now ...
Processing data subset with 22327 genes and 252 cells:
Remove genes detected in <100 cells. Remaining 43 genes.  Elapsed time is 0.013783 seconds.
Iterate 10 random permutations for gene-gene similarity threshold ...   10 Elapsed time is 0.015943 seconds.
Compute gene-gene similarity ... Elapsed time is 0.000566 seconds.
Create gene-gene graph for clustering genes ...
Writing graph into file ... 101%Elapsed time is 0.002407 seconds.
Running ModularityOptimizer for clustering ...Elapsed time is 0.184739 seconds.
Gene-gene graph contains 0 pathways, 0 genes in total
Elapsed time is 0.188823 seconds.

Remaining clusters to partition 8
Processing cluster 30 now ...
Processing data subset with 22327 genes and 182 cells:
Remove genes detected in <100 cells. Remaining 0 genes.  Elapsed time is 0.011907 seconds.

Remaining clusters to partition 7
Processing cluster 31 now ...
Processing data subset with 22327 genes and 2121 cells:
Remove genes detected in <100 cells. Remaining 11232 genes.  Elapsed time is 0.218865 seconds.
Iterate 10 random permutations for gene-gene similarity threshold ...   10 Elapsed time is 132.408722 seconds.
Compute gene-gene similarity ... Elapsed time is 9.529734 seconds.
Create gene-gene graph for clustering genes ...
Writing graph into file ... 100%Elapsed time is 39.770442 seconds.
Running ModularityOptimizer for clustering ...Elapsed time is 125.908694 seconds.
Gene-gene graph contains 5 pathways, 10803 genes in total
Elapsed time is 126.356419 seconds.
Create cell-cell graph for clustering cells ...
Writing graph into file ... 100%Elapsed time is 0.260394 seconds.
Running ModularityOptimizer for clustering ...Elapsed time is 1.122628 seconds.
Cell-cell graph contains 11 cell types by community detection
Elapsed time is 1.169694 seconds.
Cell-cell graph contains 10 cell types after merging tiny cell clusters
creating a total of 9 edges ...       9
Cell-cell graph contains 2 cell types after merging
Number of useful pathways is 1
```

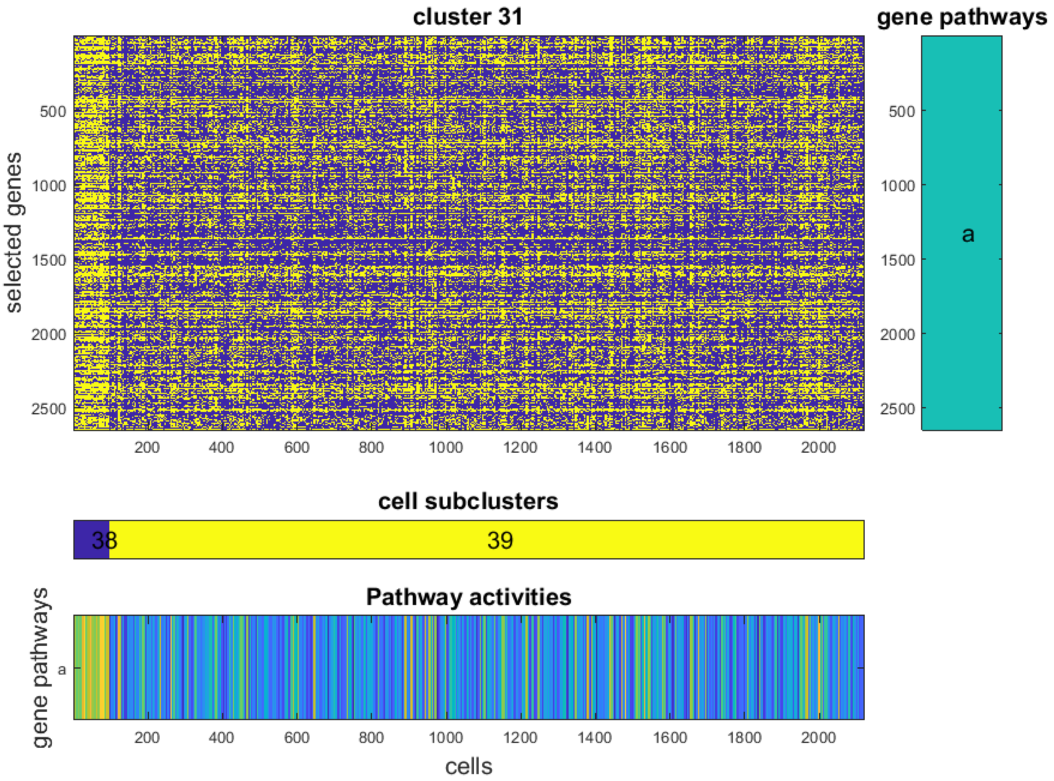


```
Remaining clusters to partition 8
Processing cluster 32 now ...
Processing data subset with 22327 genes and 3996 cells:
Remove genes detected in <100 cells. Remaining 12623 genes.  Elapsed time is 0.385423 seconds.
Iterate 10 random permutations for gene-gene similarity threshold ...   10 Elapsed time is 216.764075 seconds.
Compute gene-gene similarity ... Elapsed time is 15.754979 seconds.
Create gene-gene graph for clustering genes ...
Writing graph into file ... 100%Elapsed time is 4.692392 seconds.
Running ModularityOptimizer for clustering ...Elapsed time is 17.193897 seconds.
Gene-gene graph contains 8 pathways, 10795 genes in total
Elapsed time is 17.691063 seconds.
Create cell-cell graph for clustering cells ...
Writing graph into file ... 100%Elapsed time is 0.466931 seconds.
Running ModularityOptimizer for clustering ...Elapsed time is 2.544209 seconds.
Cell-cell graph contains 18 cell types by community detection
Elapsed time is 2.629720 seconds.
Cell-cell graph contains 17 cell types after merging tiny cell clusters
creating a total of 16 edges ...     16
Cell-cell graph contains 2 cell types after merging
Number of useful pathways is 1
```

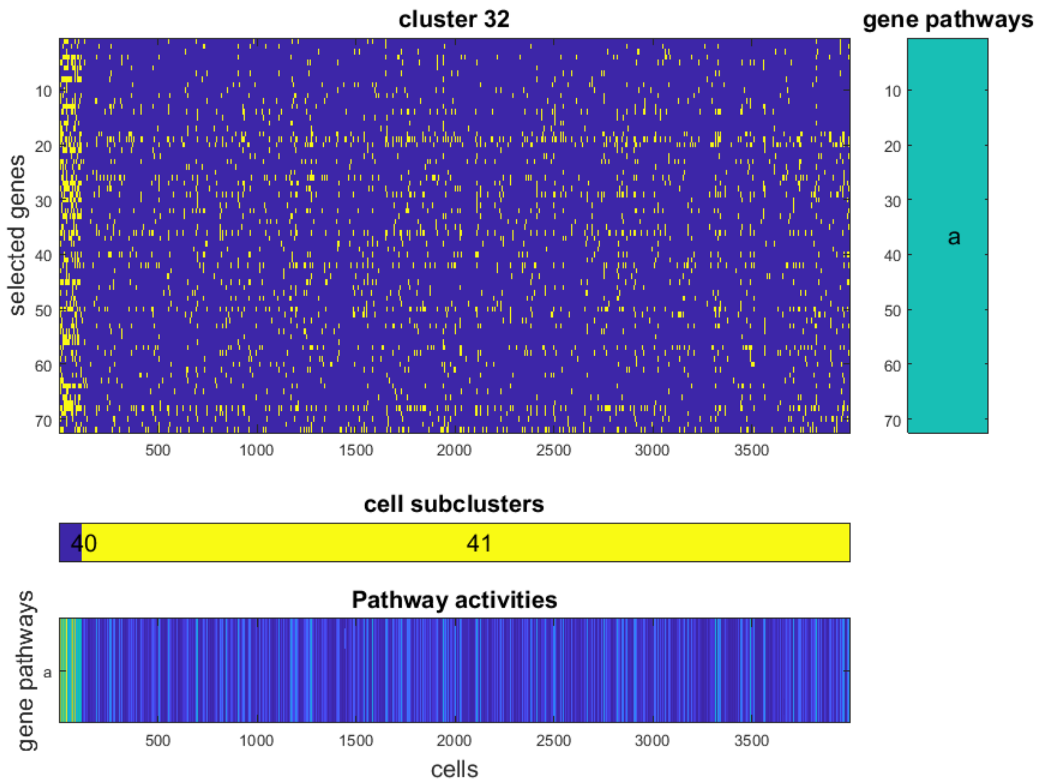


```
Remaining clusters to partition 9
Processing cluster 33 now ...
Processing data subset with 22327 genes and 26199 cells:
Remove genes detected in <100 cells. Remaining 17123 genes.  Elapsed time is 3.038860 seconds.
Iterate 10 random permutations for gene-gene similarity threshold ...   10 Elapsed time is 1629.059107 seconds.
Compute gene-gene similarity ... Elapsed time is 101.229385 seconds.
Create gene-gene graph for clustering genes ...
Writing graph into file ... 100%Elapsed time is 308.057172 seconds.
Running ModularityOptimizer for clustering ...Elapsed time is 783.834431 seconds.
Gene-gene graph contains 5 pathways, 17069 genes in total
Elapsed time is 784.768554 seconds.
Create cell-cell graph for clustering cells ...
Writing graph into file ... 100%Elapsed time is 3.040818 seconds.
Running ModularityOptimizer for clustering ...Elapsed time is 26.489763 seconds.
Cell-cell graph contains 22 cell types by community detection
Elapsed time is 27.013081 seconds.
Cell-cell graph contains 14 cell types after merging tiny cell clusters
creating a total of 13 edges ...     13
Cell-cell graph contains 2 cell types after merging
Number of useful pathways is 1
```

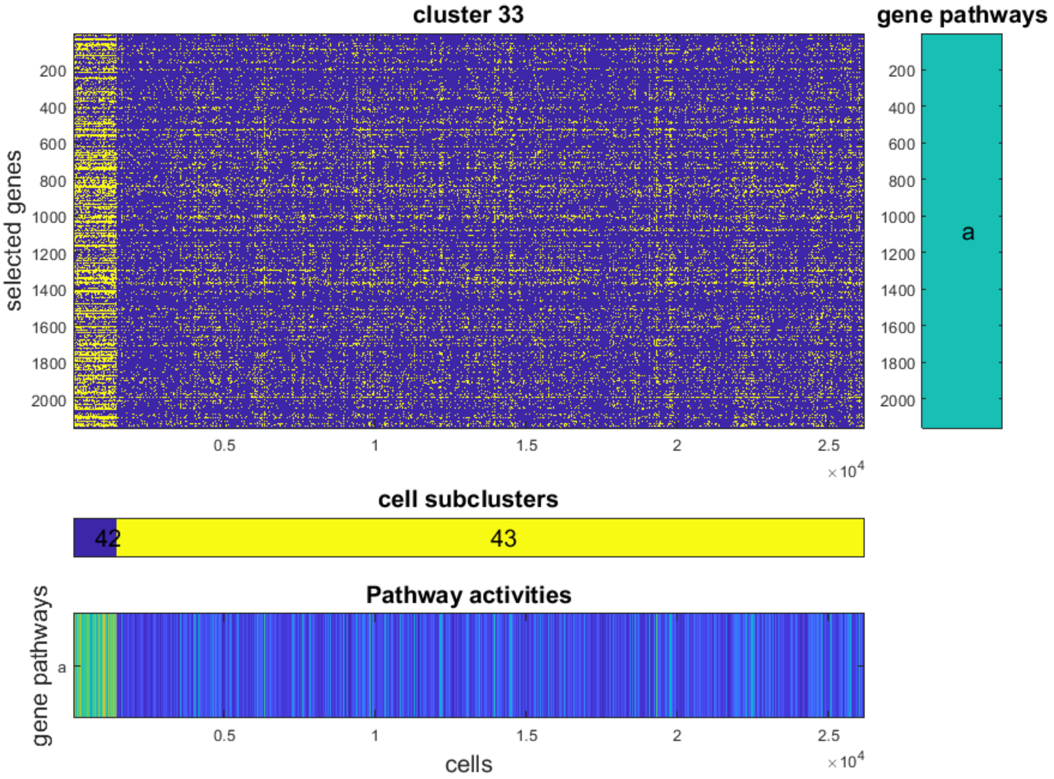


```
Remaining clusters to partition 10
Processing cluster 34 now ...
Processing data subset with 22327 genes and 193 cells:
Remove genes detected in <100 cells. Remaining 0 genes.  Elapsed time is 0.010587 seconds.

Remaining clusters to partition 9
Processing cluster 35 now ...
Processing data subset with 22327 genes and 7321 cells:
Remove genes detected in <100 cells. Remaining 12355 genes.  Elapsed time is 0.785462 seconds.
Iterate 10 random permutations for gene-gene similarity threshold ...   10 Elapsed time is 307.505480 seconds.
Compute gene-gene similarity ... Elapsed time is 20.912015 seconds.
Create gene-gene graph for clustering genes ...
Writing graph into file ... 100%Elapsed time is 18.199276 seconds.
Running ModularityOptimizer for clustering ...Elapsed time is 55.473716 seconds.
Gene-gene graph contains 5 pathways, 10638 genes in total
Elapsed time is 55.968522 seconds.
Create cell-cell graph for clustering cells ...
Writing graph into file ... 100%Elapsed time is 0.851441 seconds.
Running ModularityOptimizer for clustering ...Elapsed time is 4.945482 seconds.
Cell-cell graph contains 15 cell types by community detection
Elapsed time is 5.097863 seconds.
Cell-cell graph contains 13 cell types after merging tiny cell clusters
creating a total of 12 edges ...     12
Cell-cell graph contains 2 cell types after merging
Number of useful pathways is 1
```

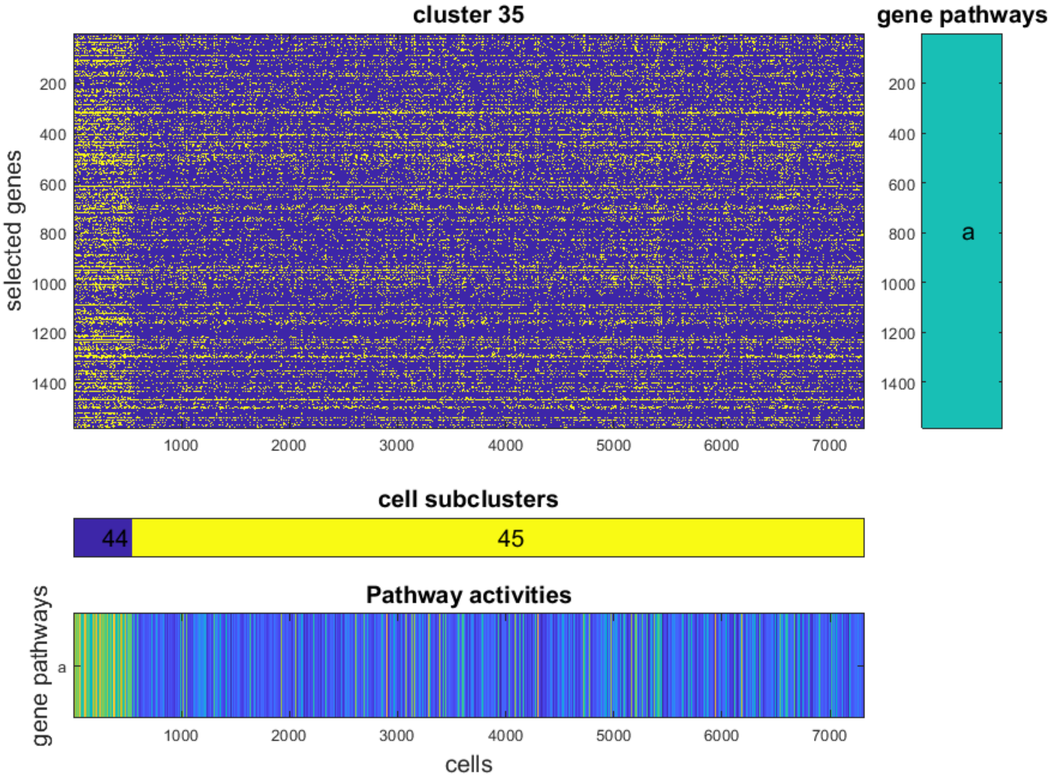


```
Remaining clusters to partition 10
Processing cluster 36 now ...
Processing data subset with 22327 genes and 158 cells:
Remove genes detected in <100 cells. Remaining 0 genes.  Elapsed time is 0.010593 seconds.

Remaining clusters to partition 9
Processing cluster 37 now ...
Processing data subset with 22327 genes and 6288 cells:
Remove genes detected in <100 cells. Remaining 4740 genes.  Elapsed time is 0.478677 seconds.
Iterate 10 random permutations for gene-gene similarity threshold ...   10 Elapsed time is 53.207431 seconds.
Compute gene-gene similarity ... Elapsed time is 2.983529 seconds.
Create gene-gene graph for clustering genes ...
Writing graph into file ... 100%Elapsed time is 0.416702 seconds.
Running ModularityOptimizer for clustering ...Elapsed time is 1.237163 seconds.
Gene-gene graph contains 6 pathways, 2354 genes in total
Elapsed time is 1.372387 seconds.
Create cell-cell graph for clustering cells ...
Writing graph into file ... 100%Elapsed time is 0.742703 seconds.
Running ModularityOptimizer for clustering ...Elapsed time is 4.512014 seconds.
Cell-cell graph contains 18 cell types by community detection
Elapsed time is 4.642009 seconds.
Cell-cell graph contains 18 cell types after merging tiny cell clusters
Cell-cell graph contains 2 cell types after merging
Number of useful pathways is 1
```

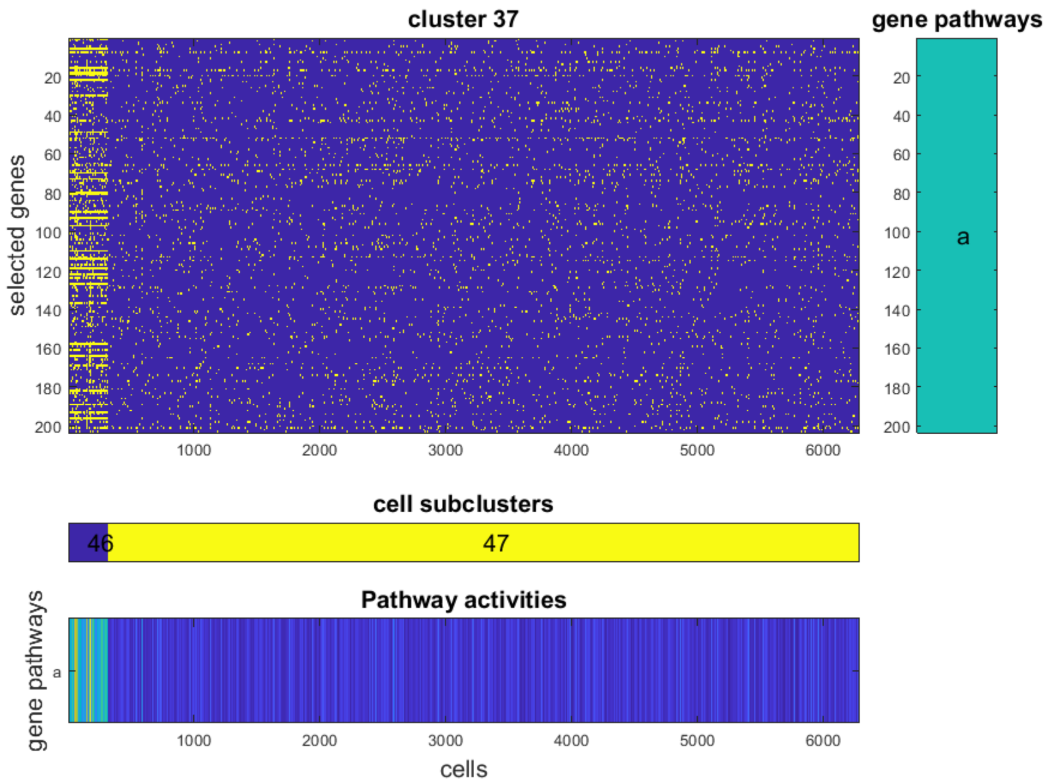


```
Remaining clusters to partition 10
Processing cluster 38 now ...
Processing data subset with 22327 genes and 96 cells:
Remove genes detected in <100 cells. Remaining 0 genes.  Elapsed time is 0.005935 seconds.

Remaining clusters to partition 9
Processing cluster 39 now ...
Processing data subset with 22327 genes and 2025 cells:
Remove genes detected in <100 cells. Remaining 11048 genes.  Elapsed time is 0.194876 seconds.
Iterate 10 random permutations for gene-gene similarity threshold ...   10 Elapsed time is 124.480943 seconds.
Compute gene-gene similarity ... Elapsed time is 9.098562 seconds.
Create gene-gene graph for clustering genes ...
Writing graph into file ... 100%Elapsed time is 26.985410 seconds.
Running ModularityOptimizer for clustering ...Elapsed time is 107.541965 seconds.
Gene-gene graph contains 5 pathways, 10361 genes in total
Elapsed time is 107.967476 seconds.
Create cell-cell graph for clustering cells ...
Writing graph into file ... 100%Elapsed time is 0.230544 seconds.
Running ModularityOptimizer for clustering ...Elapsed time is 1.116603 seconds.
Cell-cell graph contains 11 cell types by community detection
Elapsed time is 1.161233 seconds.
Cell-cell graph contains 11 cell types after merging tiny cell clusters
creating a total of 10 edges ...     10
Cell-cell graph contains 1 cell types after merging

Remaining clusters to partition 8
Processing cluster 40 now ...
Processing data subset with 22327 genes and 117 cells:
Remove genes detected in <100 cells. Remaining 0 genes.  Elapsed time is 0.008730 seconds.

Remaining clusters to partition 7
Processing cluster 41 now ...
Processing data subset with 22327 genes and 3879 cells:
Remove genes detected in <100 cells. Remaining 12530 genes.  Elapsed time is 0.412762 seconds.
Iterate 10 random permutations for gene-gene similarity threshold ...   10 Elapsed time is 209.327199 seconds.
Compute gene-gene similarity ... Elapsed time is 15.173182 seconds.
Create gene-gene graph for clustering genes ...
Writing graph into file ... 100%Elapsed time is 4.886239 seconds.
Running ModularityOptimizer for clustering ...Elapsed time is 17.528804 seconds.
Gene-gene graph contains 7 pathways, 10874 genes in total
Elapsed time is 18.020491 seconds.
Create cell-cell graph for clustering cells ...
Writing graph into file ... 100%Elapsed time is 0.466341 seconds.
Running ModularityOptimizer for clustering ...Elapsed time is 2.215319 seconds.
Cell-cell graph contains 14 cell types by community detection
Elapsed time is 2.295098 seconds.
Cell-cell graph contains 10 cell types after merging tiny cell clusters
creating a total of 9 edges ...      9
Cell-cell graph contains 1 cell types after merging

Remaining clusters to partition 6
Processing cluster 42 now ...
Processing data subset with 22327 genes and 1414 cells:
Remove genes detected in <100 cells. Remaining 8578 genes.  Elapsed time is 0.122912 seconds.
Iterate 10 random permutations for gene-gene similarity threshold ...   10 Elapsed time is 68.800997 seconds.
Compute gene-gene similarity ... Elapsed time is 5.026678 seconds.
```

```
Create gene-gene graph for clustering genes ...
Writing graph into file ... 100%Elapsed time is 0.629100 seconds.
Running ModularityOptimizer for clustering ...Elapsed time is 2.003827 seconds.
Gene-gene graph contains 9 pathways, 5199 genes in total
Elapsed time is 2.290500 seconds.
Create cell-cell graph for clustering cells ...
Writing graph into file ... 100%Elapsed time is 0.174687 seconds.
Running ModularityOptimizer for clustering ...Elapsed time is 1.118866 seconds.
Cell-cell graph contains 8 cell types by community detection
Elapsed time is 1.151334 seconds.
Cell-cell graph contains 8 cell types after merging tiny cell clusters
creating a total of 7 edges ...      7
Cell-cell graph contains 2 cell types after merging
Number of useful pathways is 1
```

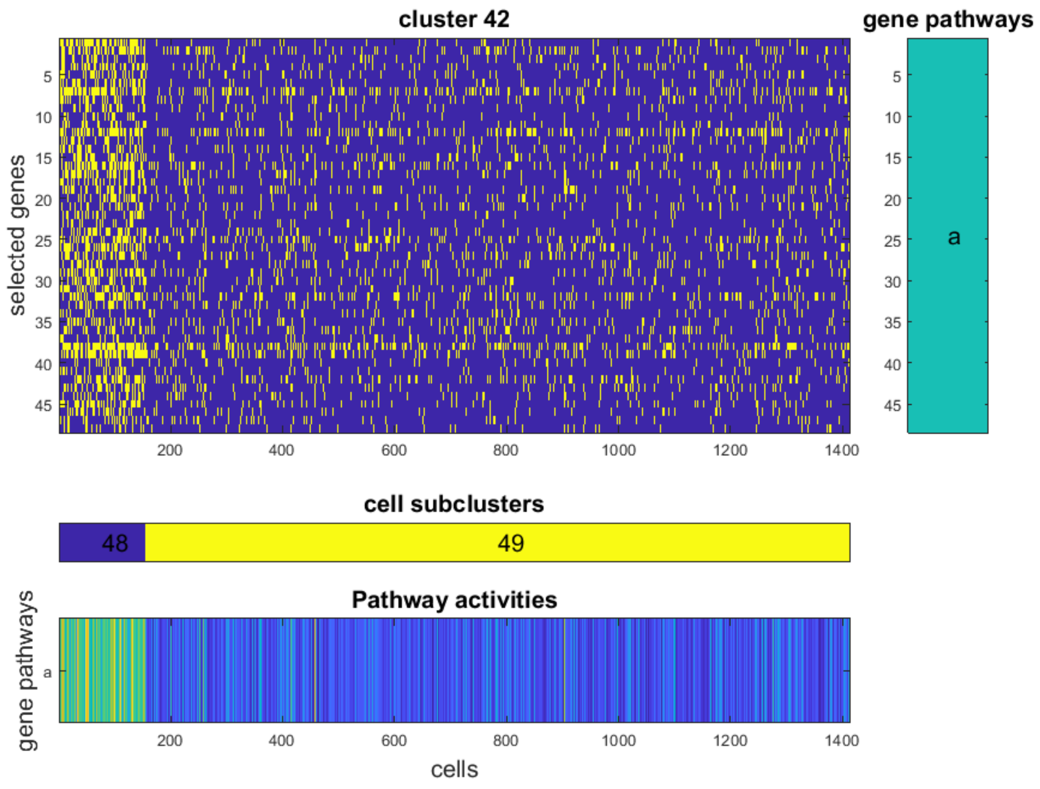


```
Remaining clusters to partition 7
Processing cluster 43 now ...
Processing data subset with 22327 genes and 24785 cells:
Remove genes detected in <100 cells. Remaining 17010 genes.  Elapsed time is 2.786318 seconds.
Iterate 10 random permutations for gene-gene similarity threshold ...   10 Elapsed time is 1505.625981 seconds.
Compute gene-gene similarity ... Elapsed time is 95.459021 seconds.
Create gene-gene graph for clustering genes ...
Writing graph into file ... 100%Elapsed time is 304.648738 seconds.
Running ModularityOptimizer for clustering ...Elapsed time is 739.320306 seconds.
Gene-gene graph contains 5 pathways, 16956 genes in total
Elapsed time is 740.224395 seconds.
Create cell-cell graph for clustering cells ...
Writing graph into file ... 100%Elapsed time is 2.911838 seconds.
Running ModularityOptimizer for clustering ...Elapsed time is 23.979031 seconds.
Cell-cell graph contains 31 cell types by community detection
Elapsed time is 24.480463 seconds.
Cell-cell graph contains 25 cell types after merging tiny cell clusters
creating a total of 24 edges ...     24
Cell-cell graph contains 2 cell types after merging
Number of useful pathways is 1
```

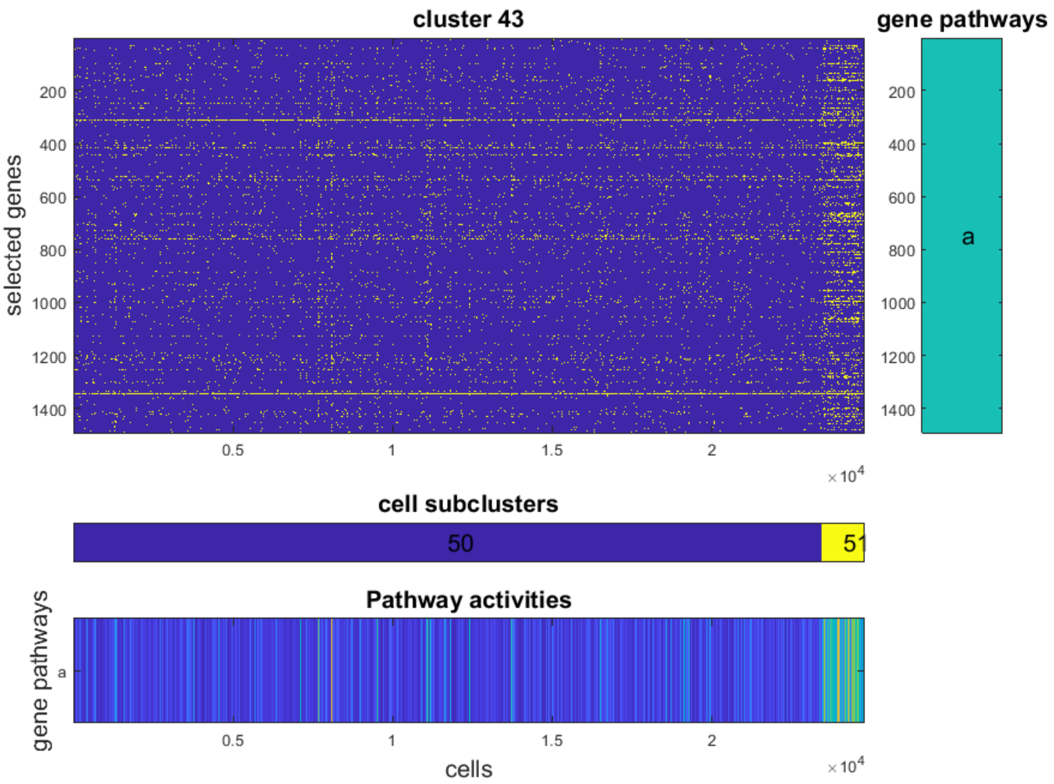


```
Remaining clusters to partition 8
Processing cluster 44 now ...
Processing data subset with 22327 genes and 541 cells:
Remove genes detected in <100 cells. Remaining 5115 genes.  Elapsed time is 0.042011 seconds.
Iterate 10 random permutations for gene-gene similarity threshold ...   10 Elapsed time is 20.313403 seconds.
Compute gene-gene similarity ... Elapsed time is 1.577131 seconds.
Create gene-gene graph for clustering genes ...
Writing graph into file ... 100%Elapsed time is 0.215100 seconds.
Running ModularityOptimizer for clustering ...Elapsed time is 0.621250 seconds.
Gene-gene graph contains 5 pathways, 1027 genes in total
Elapsed time is 0.770002 seconds.
Create cell-cell graph for clustering cells ...
Writing graph into file ... 100%Elapsed time is 0.061265 seconds.
Running ModularityOptimizer for clustering ...Elapsed time is 0.460052 seconds.
Cell-cell graph contains 8 cell types by community detection
Elapsed time is 0.473222 seconds.
Cell-cell graph contains 8 cell types after merging tiny cell clusters
creating a total of 7 edges ...       7
Cell-cell graph contains 1 cell types after merging

Remaining clusters to partition 7
Processing cluster 45 now ...
Processing data subset with 22327 genes and 6780 cells:
Remove genes detected in <100 cells. Remaining 12152 genes.  Elapsed time is 0.644280 seconds.
Iterate 10 random permutations for gene-gene similarity threshold ...   10 Elapsed time is 277.413371 seconds.
Compute gene-gene similarity ... Elapsed time is 20.007719 seconds.
Create gene-gene graph for clustering genes ...
Writing graph into file ... 100%Elapsed time is 17.469181 seconds.
Running ModularityOptimizer for clustering ...Elapsed time is 43.346541 seconds.
Gene-gene graph contains 5 pathways, 10493 genes in total
Elapsed time is 43.837411 seconds.
Create cell-cell graph for clustering cells ...
Writing graph into file ... 100%Elapsed time is 0.814190 seconds.
Running ModularityOptimizer for clustering ...Elapsed time is 4.514413 seconds.
Cell-cell graph contains 17 cell types by community detection
Elapsed time is 4.661386 seconds.
Cell-cell graph contains 15 cell types after merging tiny cell clusters
creating a total of 14 edges ...      14
Cell-cell graph contains 2 cell types after merging
Number of useful pathways is 1
```

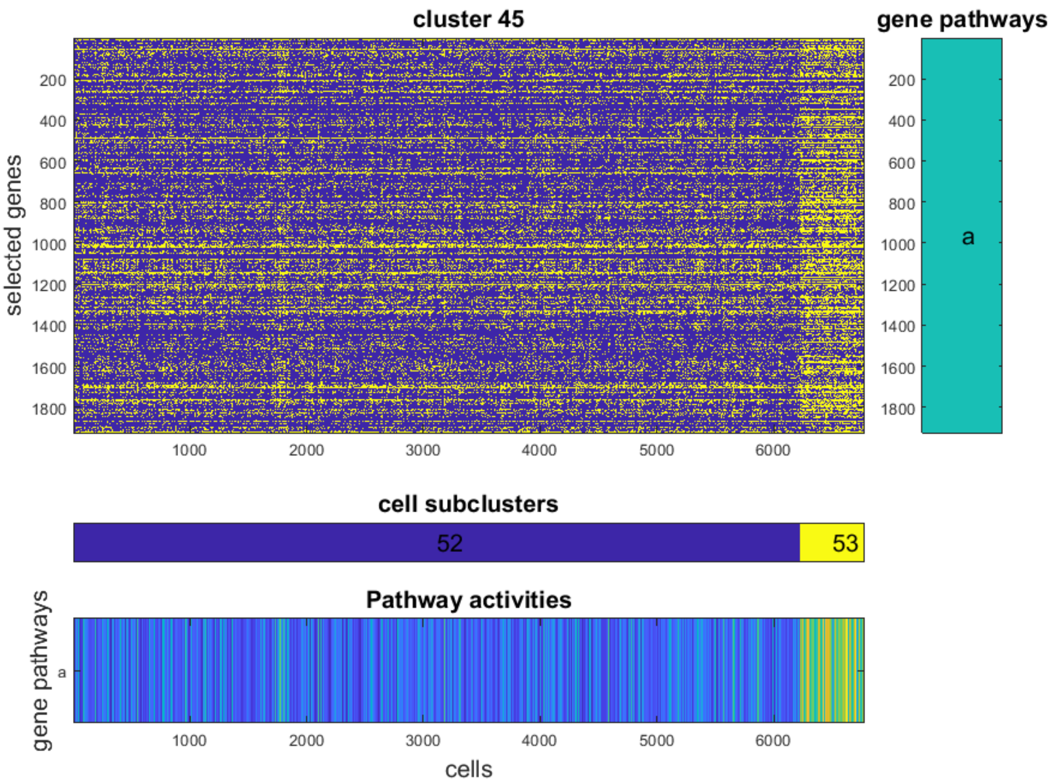


```
Remaining clusters to partition 8
Processing cluster 46 now ...
Processing data subset with 22327 genes and 311 cells:
Remove genes detected in <100 cells. Remaining 95 genes.  Elapsed time is 0.017410 seconds.
Iterate 10 random permutations for gene-gene similarity threshold ...   10 Elapsed time is 0.026063 seconds.
Compute gene-gene similarity ... Elapsed time is 0.001025 seconds.
Create gene-gene graph for clustering genes ...
Writing graph into file ... 100%Elapsed time is 0.006670 seconds.
Running ModularityOptimizer for clustering ...Elapsed time is 0.222924 seconds.
Gene-gene graph contains 1 pathways, 31 genes in total
Elapsed time is 0.243243 seconds.
Create cell-cell graph for clustering cells ...
Writing graph into file ... 100%Elapsed time is 0.035724 seconds.
Running ModularityOptimizer for clustering ...Elapsed time is 0.440774 seconds.
Cell-cell graph contains 8 cell types by community detection
Elapsed time is 0.449656 seconds.
Cell-cell graph contains 2 cell types after merging tiny cell clusters
creating a total of 1 edges ...      1
Cell-cell graph contains 1 cell types after merging

Remaining clusters to partition 7
Processing cluster 47 now ...
Processing data subset with 22327 genes and 5977 cells:
Remove genes detected in <100 cells. Remaining 4334 genes.  Elapsed time is 0.492173 seconds.
Iterate 10 random permutations for gene-gene similarity threshold ...   10 Elapsed time is 46.800565 seconds.
Compute gene-gene similarity ... Elapsed time is 2.503870 seconds.
Create gene-gene graph for clustering genes ...
Writing graph into file ... 100%Elapsed time is 0.295247 seconds.
Running ModularityOptimizer for clustering ...Elapsed time is 0.854614 seconds.
Gene-gene graph contains 9 pathways, 1768 genes in total
Elapsed time is 0.980155 seconds.
Create cell-cell graph for clustering cells ...
Writing graph into file ... 100%Elapsed time is 0.763143 seconds.
Running ModularityOptimizer for clustering ...Elapsed time is 4.941838 seconds.
Cell-cell graph contains 13 cell types by community detection
Elapsed time is 5.071214 seconds.
Cell-cell graph contains 11 cell types after merging tiny cell clusters
creating a total of 10 edges ...     10
Cell-cell graph contains 1 cell types after merging

Remaining clusters to partition 6
Processing cluster 48 now ...
Processing data subset with 22327 genes and 155 cells:
Remove genes detected in <100 cells. Remaining 0 genes.  Elapsed time is 0.011824 seconds.

Remaining clusters to partition 5
Processing cluster 49 now ...
Processing data subset with 22327 genes and 1259 cells:
Remove genes detected in <100 cells. Remaining 8282 genes.  Elapsed time is 0.123332 seconds.
Iterate 10 random permutations for gene-gene similarity threshold ...   10 Elapsed time is 62.234635 seconds.
Compute gene-gene similarity ... Elapsed time is 4.548841 seconds.
Create gene-gene graph for clustering genes ...
Writing graph into file ... 100%Elapsed time is 0.588929 seconds.
Running ModularityOptimizer for clustering ...Elapsed time is 2.217929 seconds.
Gene-gene graph contains 9 pathways, 5150 genes in total
Elapsed time is 2.490019 seconds.
```

```
Create cell-cell graph for clustering cells ...
Writing graph into file ... 100%Elapsed time is 0.152076 seconds.
Running ModularityOptimizer for clustering ...Elapsed time is 0.834455 seconds.
Cell-cell graph contains 8 cell types by community detection
Elapsed time is 0.861701 seconds.
Cell-cell graph contains 8 cell types after merging tiny cell clusters
creating a total of 7 edges ...      7
Cell-cell graph contains 2 cell types after merging
Number of useful pathways is 3
```

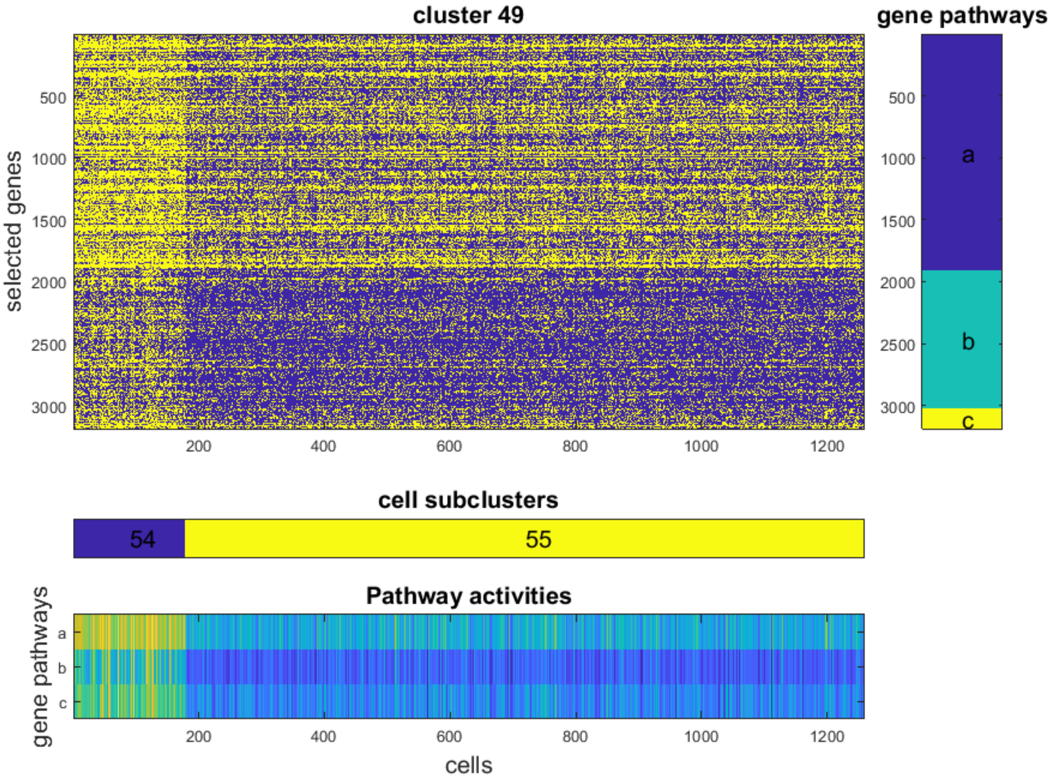


```
Remaining clusters to partition 6
Processing cluster 50 now ...
Processing data subset with 22327 genes and 23437 cells:
Remove genes detected in <100 cells. Remaining 16729 genes.  Elapsed time is 2.597409 seconds.
Iterate 10 random permutations for gene-gene similarity threshold ...   10 Elapsed time is 1378.030039 seconds.
Compute gene-gene similarity ... Elapsed time is 89.245081 seconds.
Create gene-gene graph for clustering genes ...
Writing graph into file ... 100%Elapsed time is 320.312852 seconds.
Running ModularityOptimizer for clustering ...Elapsed time is 1002.663509 seconds.
Gene-gene graph contains 5 pathways, 16679 genes in total
Elapsed time is 1003.536429 seconds.
Create cell-cell graph for clustering cells ...
Writing graph into file ... 100%Elapsed time is 2.711232 seconds.
Running ModularityOptimizer for clustering ...Elapsed time is 22.878445 seconds.
Cell-cell graph contains 23 cell types by community detection
Elapsed time is 23.354246 seconds.
Cell-cell graph contains 16 cell types after merging tiny cell clusters
creating a total of 15 edges ...     15
Cell-cell graph contains 2 cell types after merging
Number of useful pathways is 1
```

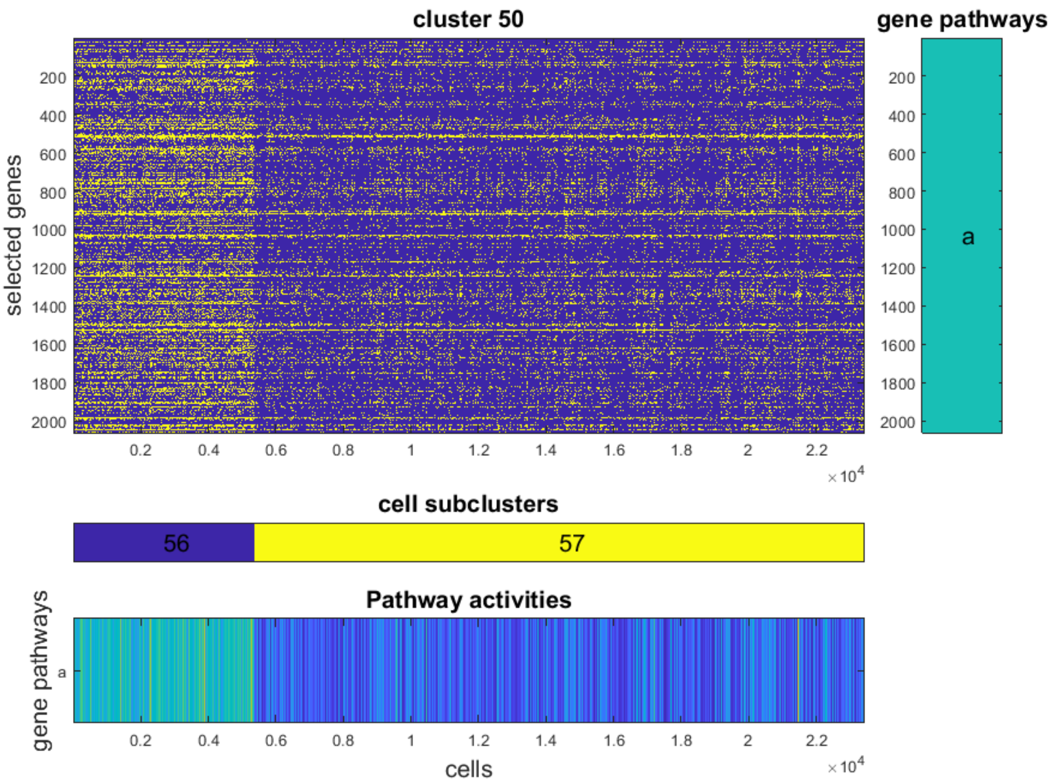


```
Remaining clusters to partition 7
Processing cluster 51 now ...
Processing data subset with 22327 genes and 1348 cells:
Remove genes detected in <100 cells. Remaining 7978 genes.  Elapsed time is 0.119169 seconds.
Iterate 10 random permutations for gene-gene similarity threshold ...   10 Elapsed time is 58.915852 seconds.
Compute gene-gene similarity ... Elapsed time is 4.394704 seconds.
Create gene-gene graph for clustering genes ...
Writing graph into file ... 100%Elapsed time is 3.067458 seconds.
Running ModularityOptimizer for clustering ...Elapsed time is 8.451485 seconds.
Gene-gene graph contains 4 pathways, 5447 genes in total
Elapsed time is 8.713588 seconds.
Create cell-cell graph for clustering cells ...
Writing graph into file ... 100%Elapsed time is 0.150027 seconds.
Running ModularityOptimizer for clustering ...Elapsed time is 0.799765 seconds.
Cell-cell graph contains 13 cell types by community detection
Elapsed time is 0.828937 seconds.
Cell-cell graph contains 13 cell types after merging tiny cell clusters
creating a total of 12 edges ...     12
Cell-cell graph contains 2 cell types after merging
Number of useful pathways is 1
```

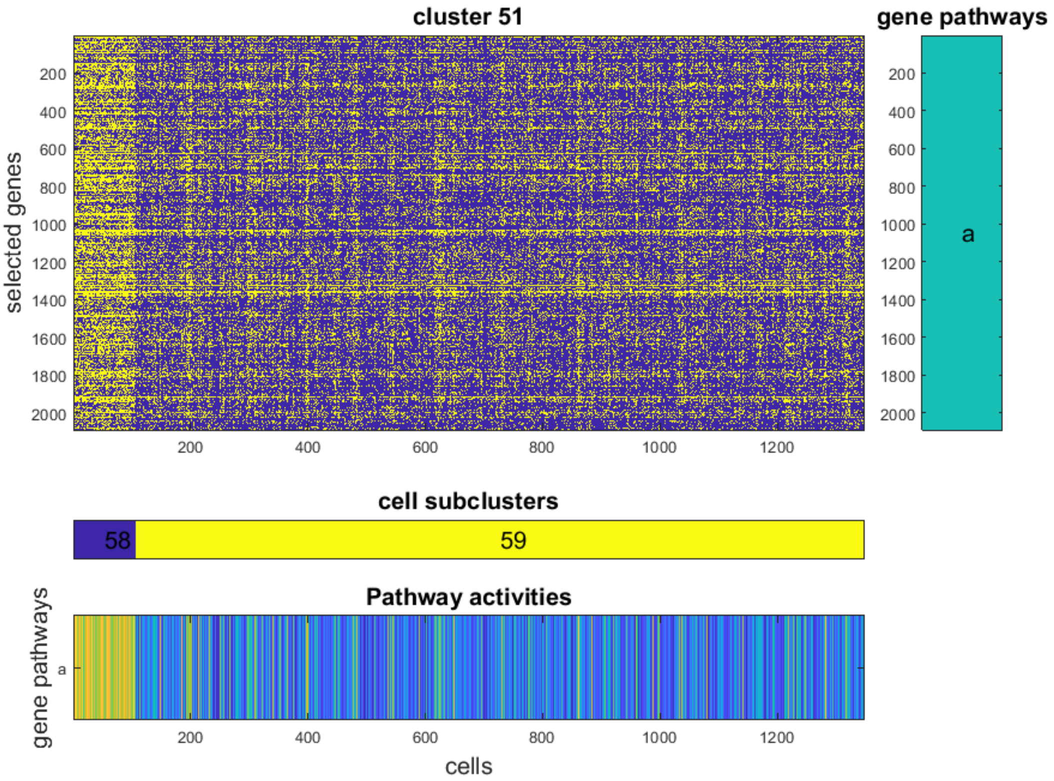


```
Remaining clusters to partition 8
Processing cluster 52 now ...
Processing data subset with 22327 genes and 6224 cells:
Remove genes detected in <100 cells. Remaining 11967 genes.  Elapsed time is 0.610693 seconds.
Iterate 10 random permutations for gene-gene similarity threshold ...   10 Elapsed time is 256.613273 seconds.
Compute gene-gene similarity ... Elapsed time is 17.871065 seconds.
Create gene-gene graph for clustering genes ...
Writing graph into file ... 100%Elapsed time is 15.895380 seconds.
Running ModularityOptimizer for clustering ...Elapsed time is 53.175047 seconds.
Gene-gene graph contains 5 pathways, 9857 genes in total
Elapsed time is 53.636495 seconds.
Create cell-cell graph for clustering cells ...
Writing graph into file ... 100%Elapsed time is 0.736458 seconds.
Running ModularityOptimizer for clustering ...Elapsed time is 3.744024 seconds.
Cell-cell graph contains 15 cell types by community detection
Elapsed time is 3.870929 seconds.
Cell-cell graph contains 12 cell types after merging tiny cell clusters
creating a total of 11 edges ...     11
Cell-cell graph contains 2 cell types after merging
Number of useful pathways is 1
```

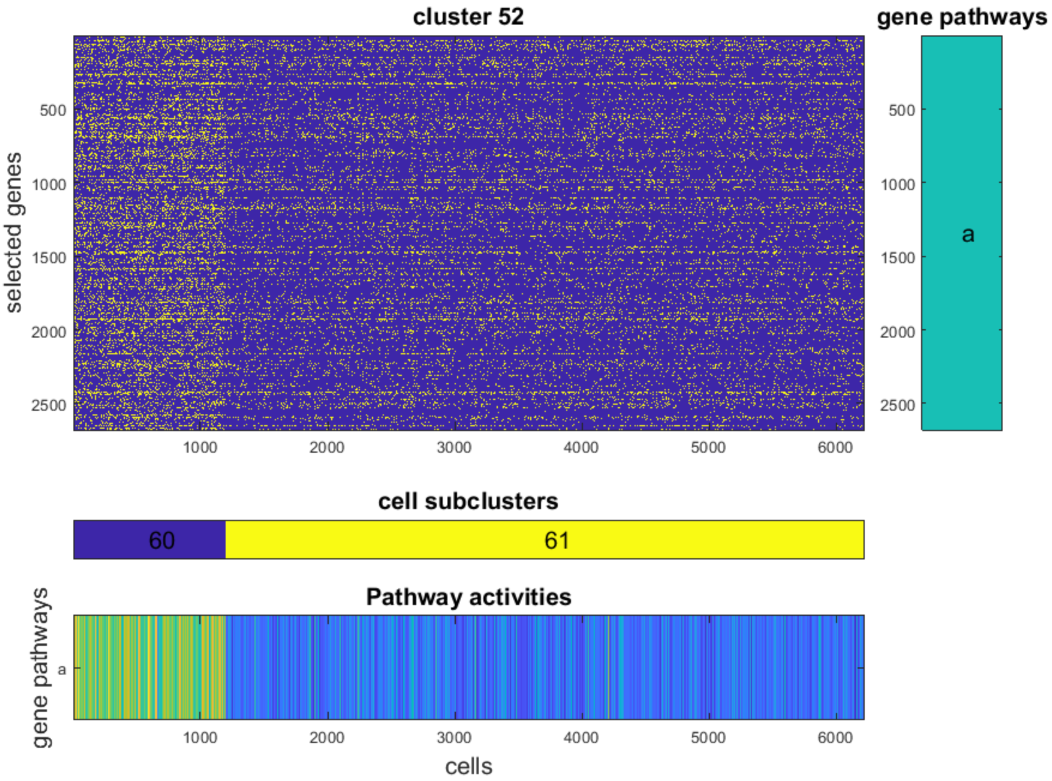


```
Remaining clusters to partition 9
Processing cluster 53 now ...
Processing data subset with 22327 genes and 556 cells:
Remove genes detected in <100 cells. Remaining 3463 genes.  Elapsed time is 0.039915 seconds.
Iterate 10 random permutations for gene-gene similarity threshold ...   10 Elapsed time is 9.778037 seconds.
Compute gene-gene similarity ... Elapsed time is 0.726241 seconds.
Create gene-gene graph for clustering genes ...
Writing graph into file ... 100%Elapsed time is 0.306426 seconds.
Running ModularityOptimizer for clustering ...Elapsed time is 0.930340 seconds.
Gene-gene graph contains 3 pathways, 1793 genes in total
Elapsed time is 1.024529 seconds.
Create cell-cell graph for clustering cells ...
Writing graph into file ... 100%Elapsed time is 0.058448 seconds.
Running ModularityOptimizer for clustering ...Elapsed time is 0.393198 seconds.
Cell-cell graph contains 10 cell types by community detection
Elapsed time is 0.407396 seconds.
Cell-cell graph contains 10 cell types after merging tiny cell clusters
creating a total of 9 edges ...      9
Cell-cell graph contains 2 cell types after merging
Number of useful pathways is 1
```

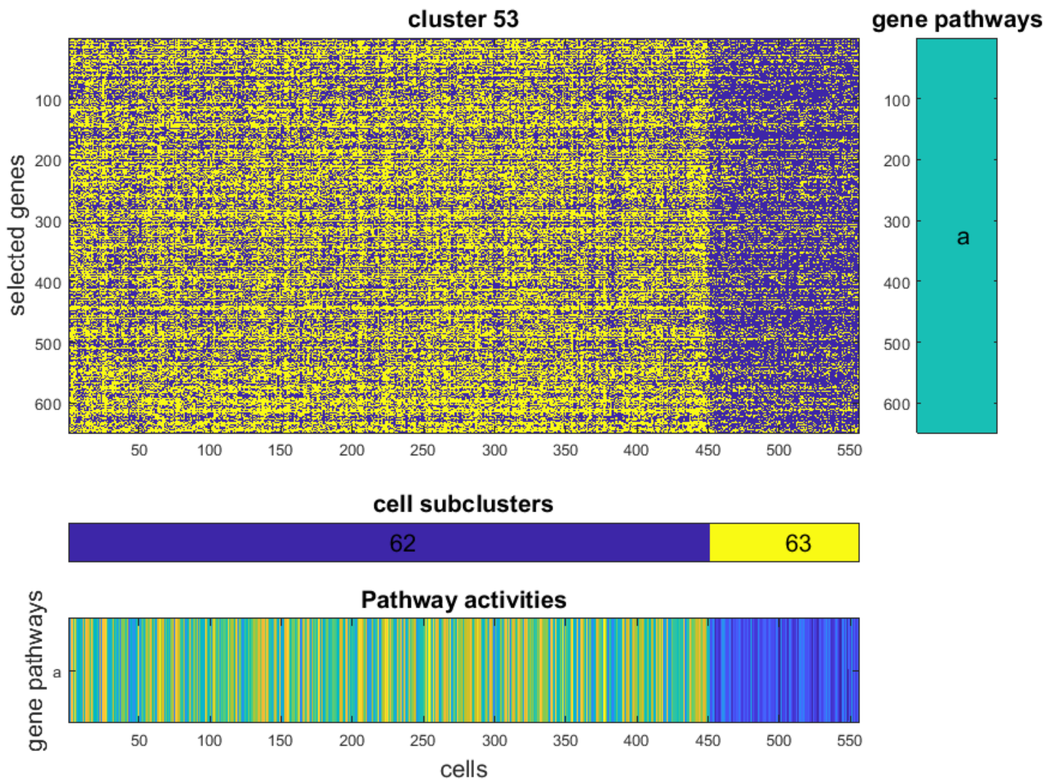


```
Remaining clusters to partition 10
Processing cluster 54 now ...
Processing data subset with 22327 genes and 179 cells:
Remove genes detected in <100 cells. Remaining 0 genes.  Elapsed time is 0.009770 seconds.

Remaining clusters to partition 9
Processing cluster 55 now ...
Processing data subset with 22327 genes and 1080 cells:
Remove genes detected in <100 cells. Remaining 7424 genes.  Elapsed time is 0.089707 seconds.
Iterate 10 random permutations for gene-gene similarity threshold ...   10 Elapsed time is 48.578084 seconds.
Compute gene-gene similarity ... Elapsed time is 3.637481 seconds.
Create gene-gene graph for clustering genes ...
Writing graph into file ... 100%Elapsed time is 0.373261 seconds.
Running ModularityOptimizer for clustering ...Elapsed time is 0.783850 seconds.
Gene-gene graph contains 9 pathways, 1083 genes in total
Elapsed time is 1.030272 seconds.
Create cell-cell graph for clustering cells ...
Writing graph into file ... 100%Elapsed time is 0.140544 seconds.
Running ModularityOptimizer for clustering ...Elapsed time is 0.892846 seconds.
Cell-cell graph contains 8 cell types by community detection
Elapsed time is 0.923371 seconds.
Cell-cell graph contains 8 cell types after merging tiny cell clusters
creating a total of 7 edges ...      7
Cell-cell graph contains 2 cell types after merging
Number of useful pathways is 1
```

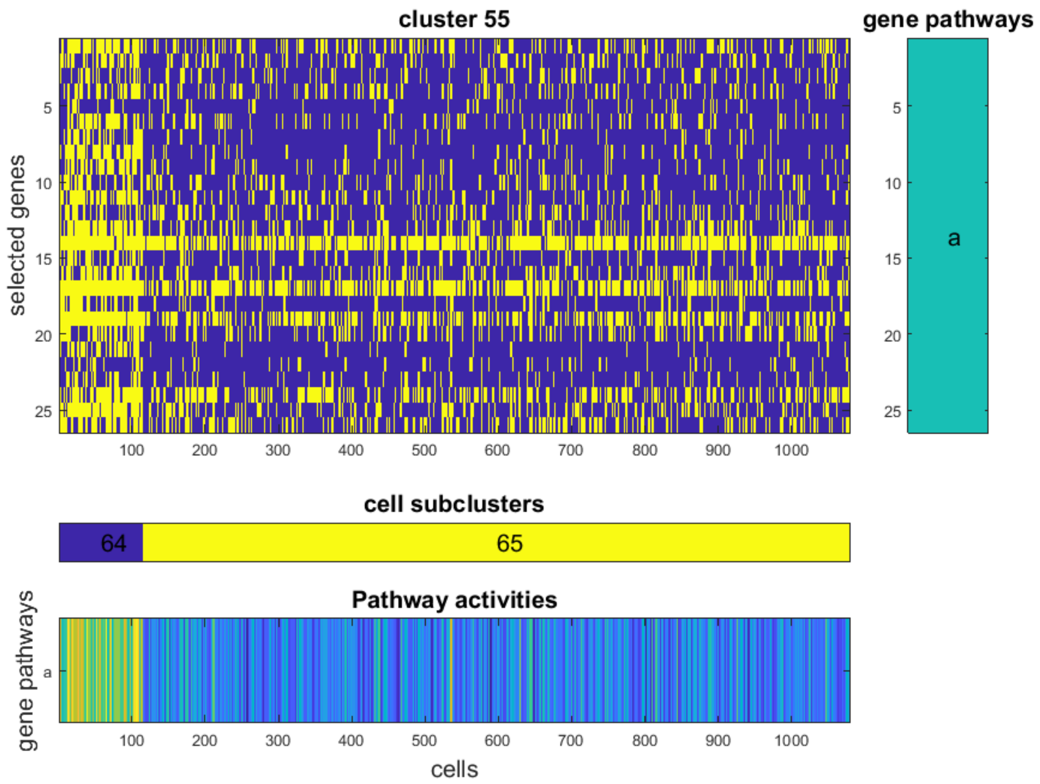


```
Remaining clusters to partition 10
Processing cluster 56 now ...
Processing data subset with 22327 genes and 5359 cells:
Remove genes detected in <100 cells. Remaining 12538 genes.  Elapsed time is 0.538715 seconds.
Iterate 10 random permutations for gene-gene similarity threshold ...   10 Elapsed time is 252.608931 seconds.
Compute gene-gene similarity ... Elapsed time is 17.982264 seconds.
Create gene-gene graph for clustering genes ...
Writing graph into file ... 100%Elapsed time is 9.179135 seconds.
Running ModularityOptimizer for clustering ...Elapsed time is 25.288498 seconds.
Gene-gene graph contains 5 pathways, 9592 genes in total
Elapsed time is 25.780314 seconds.
Create cell-cell graph for clustering cells ...
Writing graph into file ... 100%Elapsed time is 0.614102 seconds.
Running ModularityOptimizer for clustering ...Elapsed time is 3.414320 seconds.
Cell-cell graph contains 18 cell types by community detection
Elapsed time is 3.525785 seconds.
Cell-cell graph contains 16 cell types after merging tiny cell clusters
creating a total of 15 edges ...     15
Cell-cell graph contains 1 cell types after merging

Remaining clusters to partition 9
Processing cluster 57 now ...
Processing data subset with 22327 genes and 18078 cells:
Remove genes detected in <100 cells. Remaining 16342 genes.  Elapsed time is 1.954671 seconds.
Iterate 10 random permutations for gene-gene similarity threshold ...   10 Elapsed time is 1014.428624 seconds.
Compute gene-gene similarity ... Elapsed time is 68.093820 seconds.
Create gene-gene graph for clustering genes ...
Writing graph into file ... 100%Elapsed time is 326.013878 seconds.
Running ModularityOptimizer for clustering ...Elapsed time is 788.180899 seconds.
Gene-gene graph contains 4 pathways, 16299 genes in total
Elapsed time is 789.045209 seconds.
Create cell-cell graph for clustering cells ...
Writing graph into file ... 100%Elapsed time is 2.060823 seconds.
Running ModularityOptimizer for clustering ...Elapsed time is 13.591390 seconds.
Cell-cell graph contains 27 cell types by community detection
Elapsed time is 13.953503 seconds.
Cell-cell graph contains 23 cell types after merging tiny cell clusters
creating a total of 22 edges ...     22
Cell-cell graph contains 2 cell types after merging
Number of useful pathways is 1
```

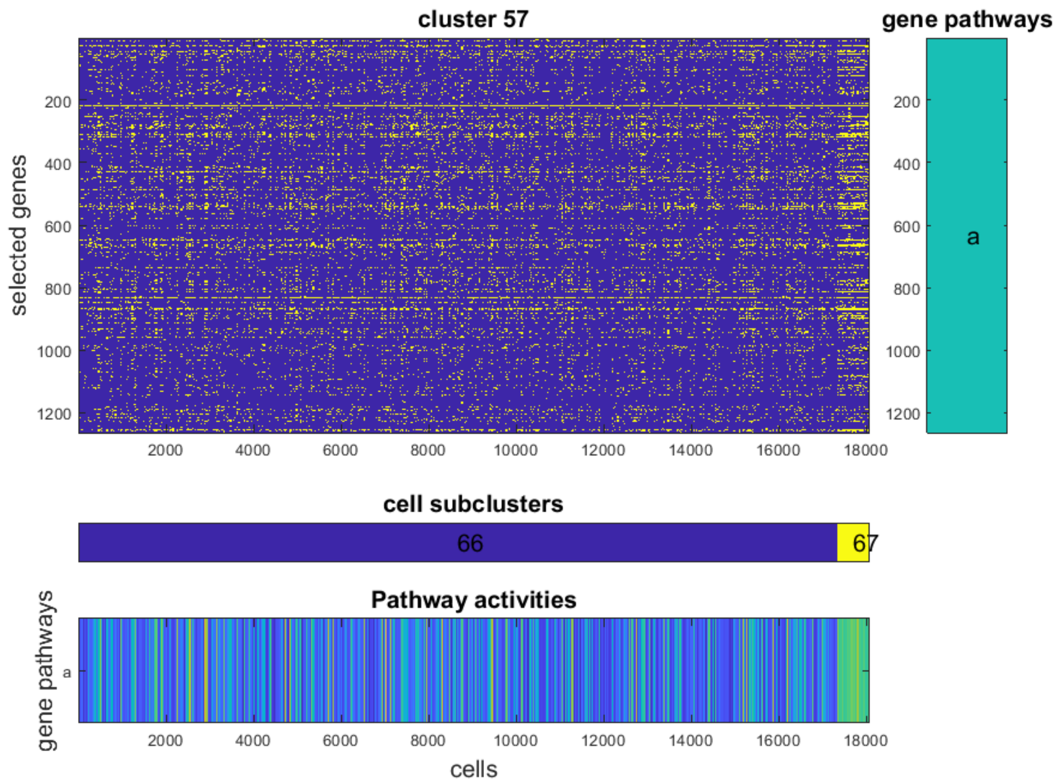


```
Remaining clusters to partition 10
Processing cluster 58 now ...
Processing data subset with 22327 genes and 107 cells:
Remove genes detected in <100 cells. Remaining 0 genes.  Elapsed time is 0.006419 seconds.

Remaining clusters to partition 9
Processing cluster 59 now ...
Processing data subset with 22327 genes and 1241 cells:
Remove genes detected in <100 cells. Remaining 7519 genes.  Elapsed time is 0.102714 seconds.
Iterate 10 random permutations for gene-gene similarity threshold ...   10 Elapsed time is 51.396094 seconds.
Compute gene-gene similarity ... Elapsed time is 3.823508 seconds.
Create gene-gene graph for clustering genes ...
Writing graph into file ... 100%Elapsed time is 2.014998 seconds.
Running ModularityOptimizer for clustering ...Elapsed time is 4.839392 seconds.
Gene-gene graph contains 5 pathways, 4821 genes in total
Elapsed time is 5.078799 seconds.
Create cell-cell graph for clustering cells ...
Writing graph into file ... 100%Elapsed time is 0.141691 seconds.
Running ModularityOptimizer for clustering ...Elapsed time is 0.768341 seconds.
Cell-cell graph contains 11 cell types by community detection
Elapsed time is 0.797969 seconds.
Cell-cell graph contains 10 cell types after merging tiny cell clusters
Cell-cell graph contains 2 cell types after merging
Number of useful pathways is 1
```

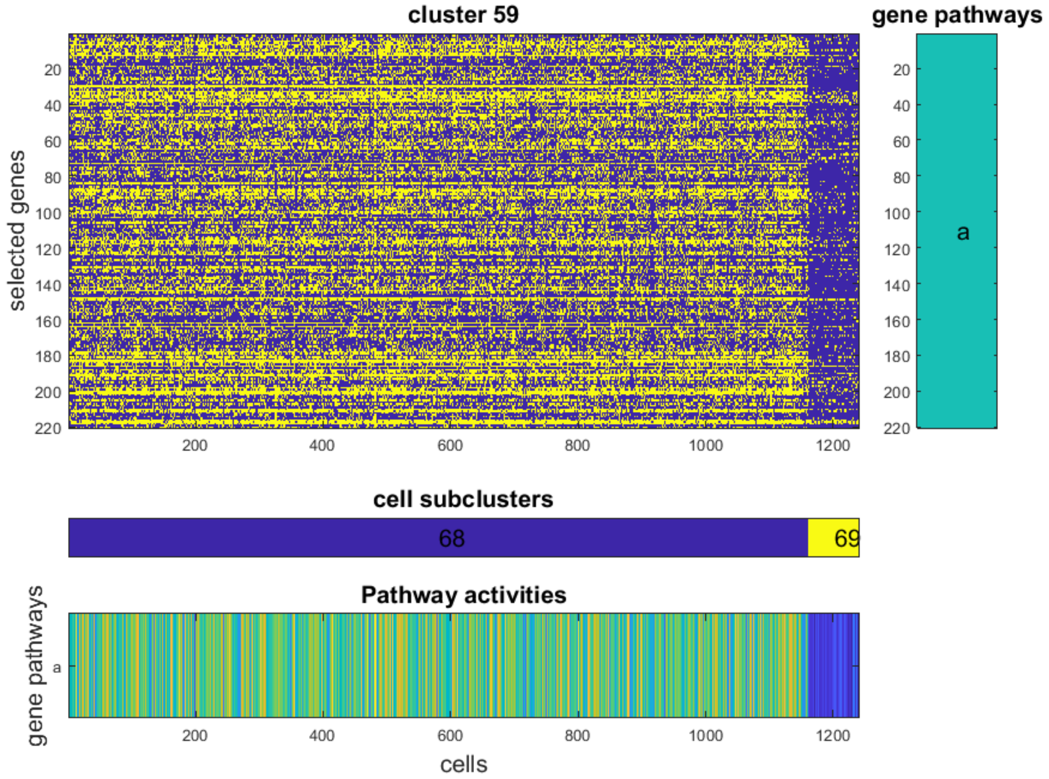


```
Remaining clusters to partition 10
Processing cluster 60 now ...
Processing data subset with 22327 genes and 1197 cells:
Remove genes detected in <100 cells. Remaining 8118 genes.  Elapsed time is 0.102779 seconds.
Iterate 10 random permutations for gene-gene similarity threshold ...   10 Elapsed time is 58.218438 seconds.
Compute gene-gene similarity ... Elapsed time is 4.595729 seconds.
Create gene-gene graph for clustering genes ...
Writing graph into file ... 100%Elapsed time is 1.114873 seconds.
Running ModularityOptimizer for clustering ...Elapsed time is 2.210514 seconds.
Gene-gene graph contains 6 pathways, 3866 genes in total
Elapsed time is 2.502300 seconds.
Create cell-cell graph for clustering cells ...
Writing graph into file ... 100%Elapsed time is 0.142527 seconds.
Running ModularityOptimizer for clustering ...Elapsed time is 0.693626 seconds.
Cell-cell graph contains 10 cell types by community detection
Elapsed time is 0.724657 seconds.
Cell-cell graph contains 9 cell types after merging tiny cell clusters
creating a total of 8 edges ...      8
Cell-cell graph contains 2 cell types after merging
Number of useful pathways is 1
```

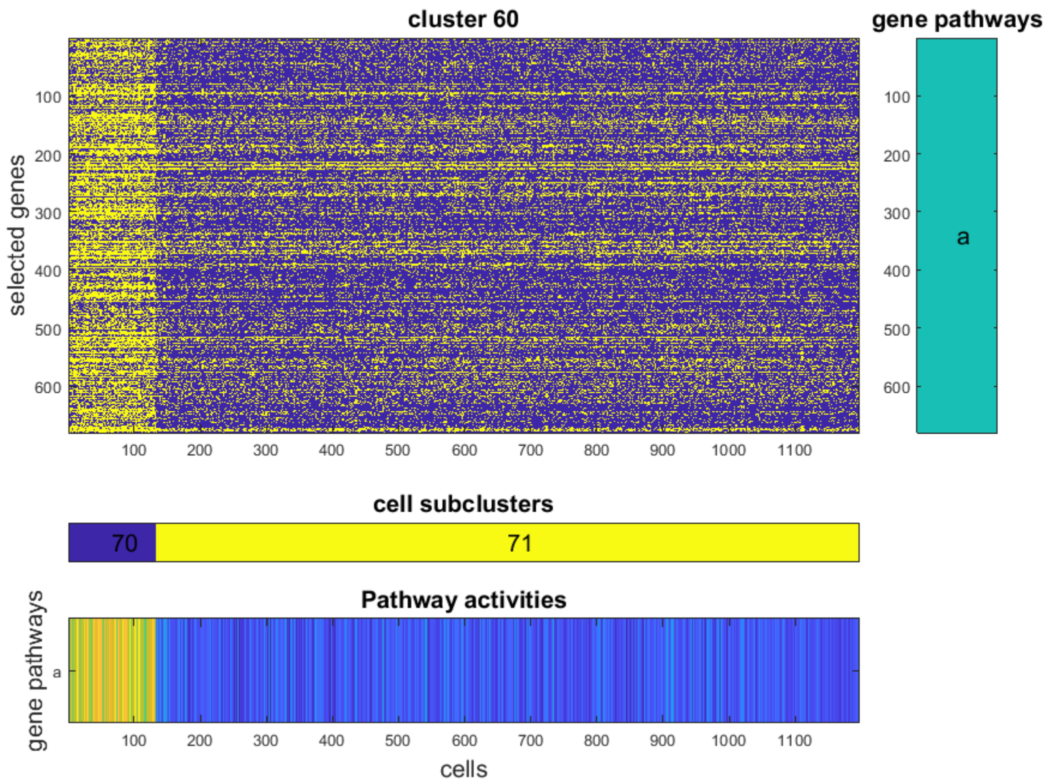


```
Remaining clusters to partition 11
Processing cluster 61 now ...
Processing data subset with 22327 genes and 5027 cells:
Remove genes detected in <100 cells. Remaining 11172 genes.  Elapsed time is 0.469781 seconds.
Iterate 10 random permutations for gene-gene similarity threshold ...   10 Elapsed time is 193.926183 seconds.
Compute gene-gene similarity ... Elapsed time is 13.914852 seconds.
Create gene-gene graph for clustering genes ...
Writing graph into file ... 100%Elapsed time is 9.973151 seconds.
Running ModularityOptimizer for clustering ...Elapsed time is 30.980495 seconds.
Gene-gene graph contains 5 pathways, 7707 genes in total
Elapsed time is 31.398471 seconds.
Create cell-cell graph for clustering cells ...
Writing graph into file ... 100%Elapsed time is 0.583699 seconds.
Running ModularityOptimizer for clustering ...Elapsed time is 3.082299 seconds.
Cell-cell graph contains 16 cell types by community detection
Elapsed time is 3.185650 seconds.
Cell-cell graph contains 16 cell types after merging tiny cell clusters
creating a total of 15 edges ...     15
Cell-cell graph contains 2 cell types after merging
Number of useful pathways is 1
```

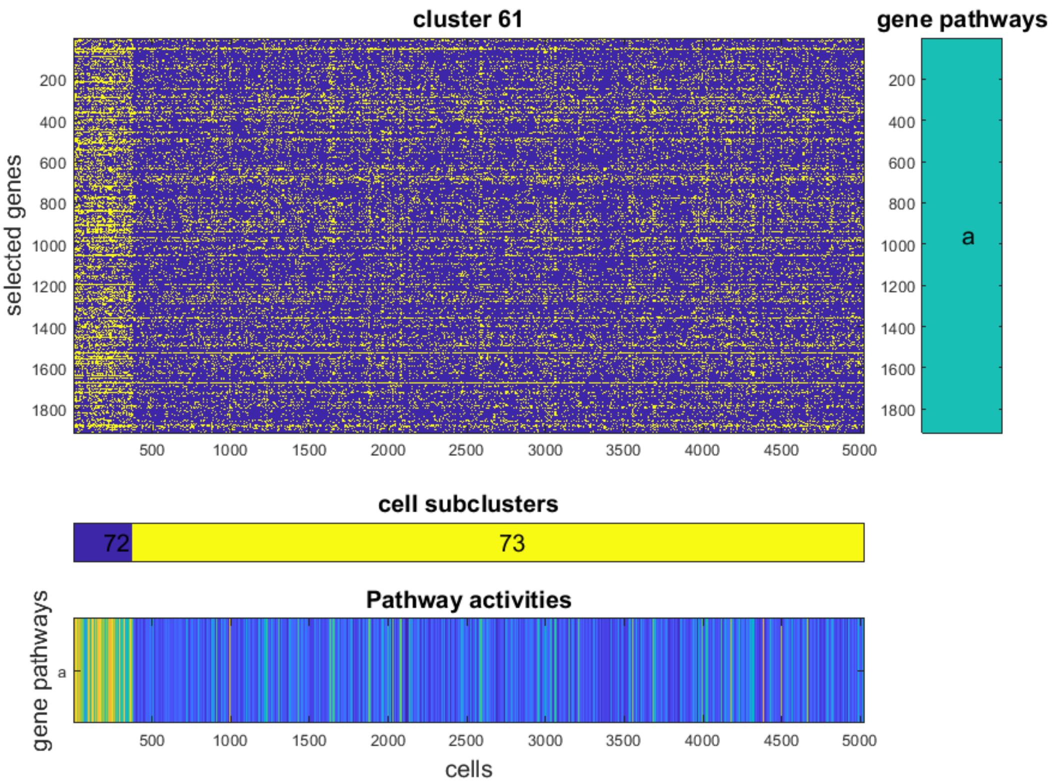


```
Remaining clusters to partition 12
Processing cluster 62 now ...
Processing data subset with 22327 genes and 451 cells:
Remove genes detected in <100 cells. Remaining 2938 genes.  Elapsed time is 0.031918 seconds.
Iterate 10 random permutations for gene-gene similarity threshold ...   10 Elapsed time is 6.867489 seconds.
Compute gene-gene similarity ... Elapsed time is 0.520576 seconds.
Create gene-gene graph for clustering genes ...
Writing graph into file ... 100%Elapsed time is 0.088726 seconds.
Running ModularityOptimizer for clustering ...Elapsed time is 0.537118 seconds.
Gene-gene graph contains 5 pathways, 859 genes in total
Elapsed time is 0.613433 seconds.
Create cell-cell graph for clustering cells ...
Writing graph into file ... 100%Elapsed time is 0.049464 seconds.
Running ModularityOptimizer for clustering ...Elapsed time is 0.405712 seconds.
Cell-cell graph contains 7 cell types by community detection
Elapsed time is 0.417163 seconds.
Cell-cell graph contains 7 cell types after merging tiny cell clusters
creating a total of 6 edges ...      6
Cell-cell graph contains 1 cell types after merging

Remaining clusters to partition 11
Processing cluster 63 now ...
Processing data subset with 22327 genes and 105 cells:
Remove genes detected in <100 cells. Remaining 0 genes.  Elapsed time is 0.006225 seconds.

Remaining clusters to partition 10
Processing cluster 64 now ...
Processing data subset with 22327 genes and 115 cells:
Remove genes detected in <100 cells. Remaining 0 genes.  Elapsed time is 0.006574 seconds.

Remaining clusters to partition 9
Processing cluster 65 now ...
Processing data subset with 22327 genes and 965 cells:
Remove genes detected in <100 cells. Remaining 7050 genes.  Elapsed time is 0.082927 seconds.
Iterate 10 random permutations for gene-gene similarity threshold ...   10 Elapsed time is 42.663919 seconds.
Compute gene-gene similarity ... Elapsed time is 3.176384 seconds.
Create gene-gene graph for clustering genes ...
Writing graph into file ... 100%Elapsed time is 0.336078 seconds.
Running ModularityOptimizer for clustering ...Elapsed time is 0.815570 seconds.
Gene-gene graph contains 10 pathways, 940 genes in total
Elapsed time is 1.033038 seconds.
Create cell-cell graph for clustering cells ...
Writing graph into file ... 100%Elapsed time is 0.126301 seconds.
Running ModularityOptimizer for clustering ...Elapsed time is 0.767598 seconds.
Cell-cell graph contains 8 cell types by community detection
Elapsed time is 0.789166 seconds.
Cell-cell graph contains 8 cell types after merging tiny cell clusters
creating a total of 7 edges ...      7
Cell-cell graph contains 1 cell types after merging

Remaining clusters to partition 8
Processing cluster 66 now ...
Processing data subset with 22327 genes and 17312 cells:
Remove genes detected in <100 cells. Remaining 16313 genes.  Elapsed time is 1.861746 seconds.
Iterate 10 random permutations for gene-gene similarity threshold ...   10 Elapsed time is 969.664690 seconds.
Compute gene-gene similarity ... Elapsed time is 66.271402 seconds.
```

```
Create gene-gene graph for clustering genes ...
Writing graph into file ... 100%Elapsed time is 325.214555 seconds.
Running ModularityOptimizer for clustering ...Elapsed time is 758.764328 seconds.
Gene-gene graph contains 4 pathways, 16271 genes in total
Elapsed time is 759.621374 seconds.
Create cell-cell graph for clustering cells ...
Writing graph into file ... 100%Elapsed time is 1.976525 seconds.
Running ModularityOptimizer for clustering ...Elapsed time is 12.605683 seconds.
Cell-cell graph contains 22 cell types by community detection
Elapsed time is 12.952475 seconds.
Cell-cell graph contains 13 cell types after merging tiny cell clusters
creating a total of 12 edges ...     12
Cell-cell graph contains 2 cell types after merging
Number of useful pathways is 1
```

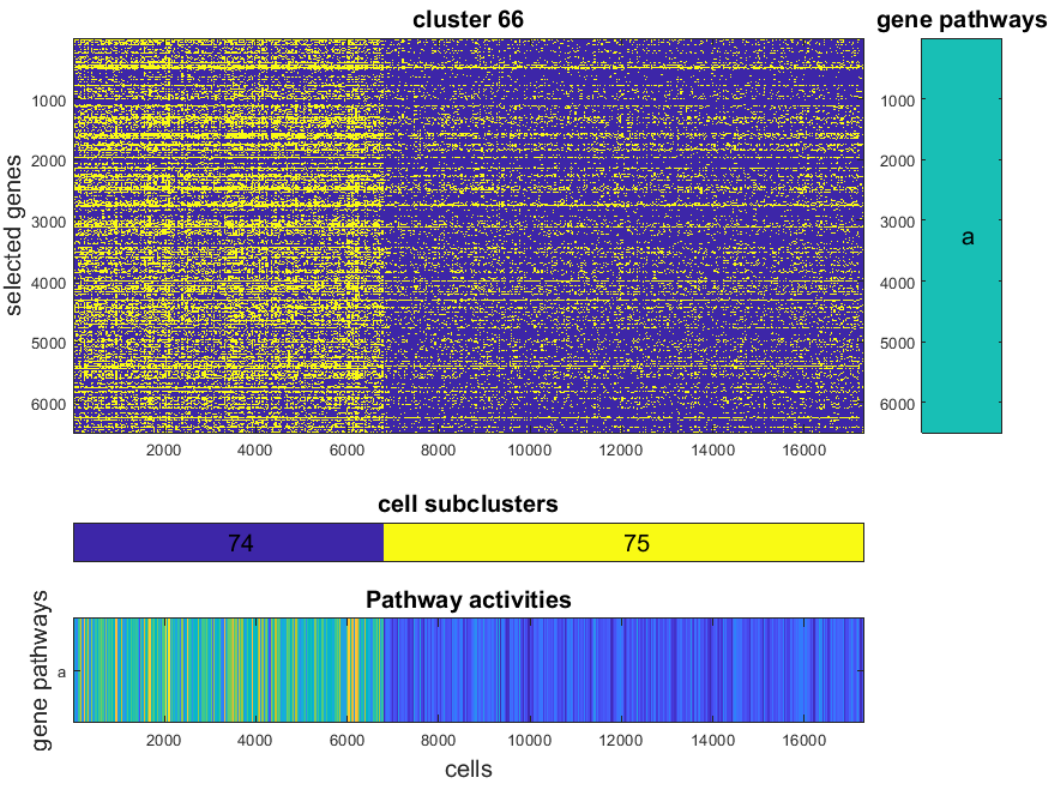


```
Remaining clusters to partition 9
Processing cluster 67 now ...
Processing data subset with 22327 genes and 766 cells:
Remove genes detected in <100 cells. Remaining 4243 genes.  Elapsed time is 0.056175 seconds.
Iterate 10 random permutations for gene-gene similarity threshold ...   10 Elapsed time is 15.457843 seconds.
Compute gene-gene similarity ... Elapsed time is 1.147582 seconds.
Create gene-gene graph for clustering genes ...
Writing graph into file ... 100%Elapsed time is 0.150503 seconds.
Running ModularityOptimizer for clustering ...Elapsed time is 0.552482 seconds.
Gene-gene graph contains 6 pathways, 721 genes in total
Elapsed time is 0.667206 seconds.
Create cell-cell graph for clustering cells ...
Writing graph into file ... 100%Elapsed time is 0.088396 seconds.
Running ModularityOptimizer for clustering ...Elapsed time is 0.566843 seconds.
Cell-cell graph contains 9 cell types by community detection
Elapsed time is 0.584441 seconds.
Cell-cell graph contains 9 cell types after merging tiny cell clusters
Cell-cell graph contains 3 cell types after merging
Number of useful pathways is 4
```

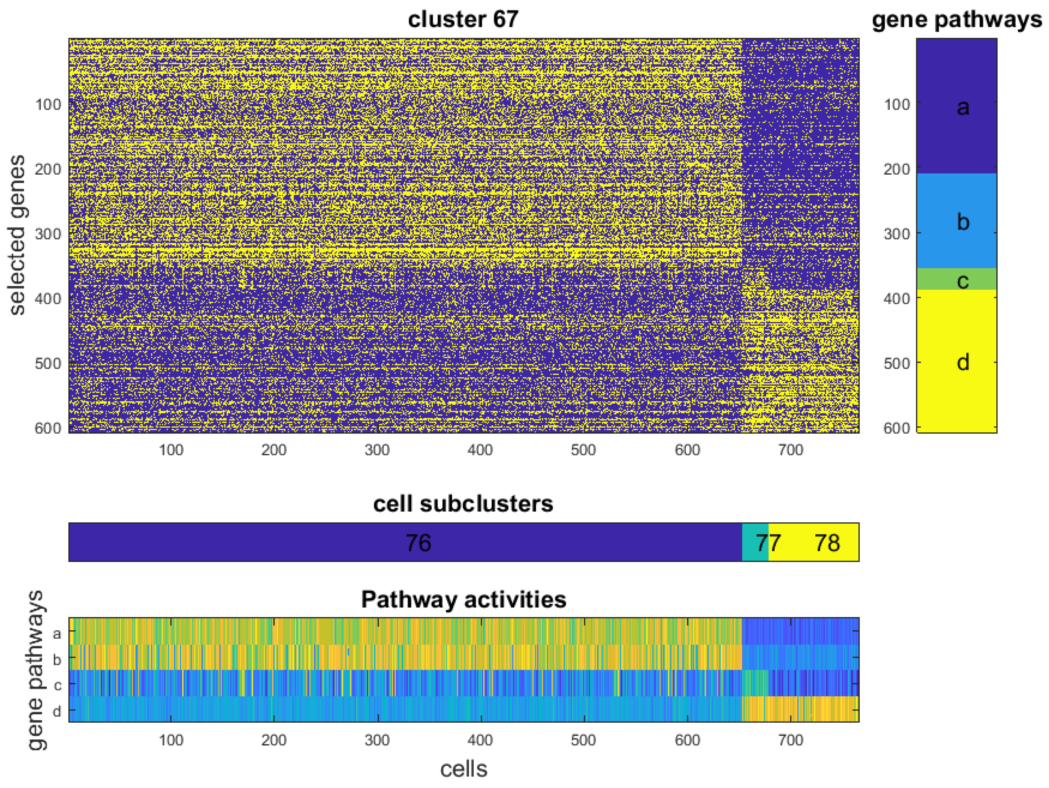


```
Remaining clusters to partition 11
Processing cluster 68 now ...
Processing data subset with 22327 genes and 1161 cells:
Remove genes detected in <100 cells. Remaining 7178 genes.  Elapsed time is 0.097656 seconds.
Iterate 10 random permutations for gene-gene similarity threshold ...   10 Elapsed time is 46.454966 seconds.
Compute gene-gene similarity ... Elapsed time is 3.414841 seconds.
Create gene-gene graph for clustering genes ...
Writing graph into file ... 100%Elapsed time is 1.960640 seconds.
Running ModularityOptimizer for clustering ...Elapsed time is 4.945472 seconds.
Gene-gene graph contains 5 pathways, 4517 genes in total
Elapsed time is 5.167496 seconds.
Create cell-cell graph for clustering cells ...
Writing graph into file ... 100%Elapsed time is 0.133163 seconds.
Running ModularityOptimizer for clustering ...Elapsed time is 0.688532 seconds.
Cell-cell graph contains 11 cell types by community detection
Elapsed time is 0.716152 seconds.
Cell-cell graph contains 10 cell types after merging tiny cell clusters
creating a total of 9 edges ...      9
Cell-cell graph contains 2 cell types after merging
Number of useful pathways is 1
```

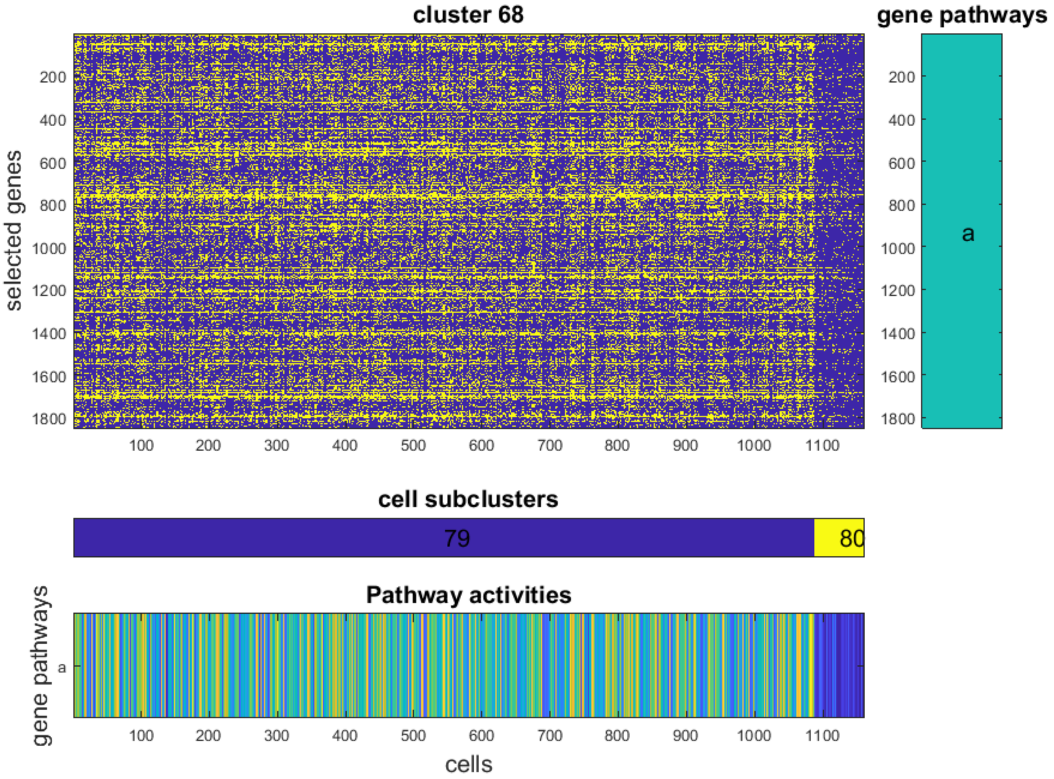


```
Remaining clusters to partition 12
Processing cluster 69 now ...
Processing data subset with 22327 genes and 80 cells:
Remove genes detected in <100 cells. Remaining 0 genes.  Elapsed time is 0.005031 seconds.

Remaining clusters to partition 11
Processing cluster 70 now ...
Processing data subset with 22327 genes and 132 cells:
Remove genes detected in <100 cells. Remaining 0 genes.  Elapsed time is 0.007606 seconds.

Remaining clusters to partition 10
Processing cluster 71 now ...
Processing data subset with 22327 genes and 1065 cells:
Remove genes detected in <100 cells. Remaining 7684 genes.  Elapsed time is 0.089345 seconds.
Iterate 10 random permutations for gene-gene similarity threshold ...   10 Elapsed time is 51.294989 seconds.
Compute gene-gene similarity ... Elapsed time is 3.967318 seconds.
Create gene-gene graph for clustering genes ...
Writing graph into file ... 100%Elapsed time is 0.854963 seconds.
Running ModularityOptimizer for clustering ...Elapsed time is 1.884033 seconds.
Gene-gene graph contains 6 pathways, 3194 genes in total
Elapsed time is 2.131011 seconds.
Create cell-cell graph for clustering cells ...
Writing graph into file ... 100%Elapsed time is 0.138492 seconds.
Running ModularityOptimizer for clustering ...Elapsed time is 0.764900 seconds.
Cell-cell graph contains 8 cell types by community detection
Elapsed time is 0.794737 seconds.
Cell-cell graph contains 7 cell types after merging tiny cell clusters
creating a total of 6 edges ...      6
Cell-cell graph contains 1 cell types after merging

Remaining clusters to partition 9
Processing cluster 72 now ...
Processing data subset with 22327 genes and 377 cells:
Remove genes detected in <100 cells. Remaining 3080 genes.  Elapsed time is 0.032230 seconds.
Iterate 10 random permutations for gene-gene similarity threshold ...   10 Elapsed time is 7.190278 seconds.
Compute gene-gene similarity ... Elapsed time is 0.547824 seconds.
Create gene-gene graph for clustering genes ...
Writing graph into file ... 100%Elapsed time is 0.074355 seconds.
Running ModularityOptimizer for clustering ...Elapsed time is 0.536492 seconds.
Gene-gene graph contains 7 pathways, 644 genes in total
Elapsed time is 0.617257 seconds.
Create cell-cell graph for clustering cells ...
Writing graph into file ... 100%Elapsed time is 0.042610 seconds.
Running ModularityOptimizer for clustering ...Elapsed time is 0.378832 seconds.
Cell-cell graph contains 6 cell types by community detection
Elapsed time is 0.389147 seconds.
Cell-cell graph contains 6 cell types after merging tiny cell clusters
creating a total of 5 edges ...      5
Cell-cell graph contains 1 cell types after merging

Remaining clusters to partition 8
Processing cluster 73 now ...
Processing data subset with 22327 genes and 4650 cells:
Remove genes detected in <100 cells. Remaining 10963 genes.  Elapsed time is 0.458542 seconds.
Iterate 10 random permutations for gene-gene similarity threshold ...   10 Elapsed time is 182.746487 seconds.
Compute gene-gene similarity ... Elapsed time is 12.895754 seconds.
```

```
Create gene-gene graph for clustering genes ...
Writing graph into file ... 100%Elapsed time is 6.115369 seconds.
Running ModularityOptimizer for clustering ...Elapsed time is 19.711548 seconds.
Gene-gene graph contains 4 pathways, 6699 genes in total
Elapsed time is 20.120184 seconds.
Create cell-cell graph for clustering cells ...
Writing graph into file ... 100%Elapsed time is 0.548891 seconds.
Running ModularityOptimizer for clustering ...Elapsed time is 2.749373 seconds.
Cell-cell graph contains 13 cell types by community detection
Elapsed time is 2.844993 seconds.
Cell-cell graph contains 12 cell types after merging tiny cell clusters
creating a total of 11 edges ...     11
Cell-cell graph contains 2 cell types after merging
Number of useful pathways is 1
```

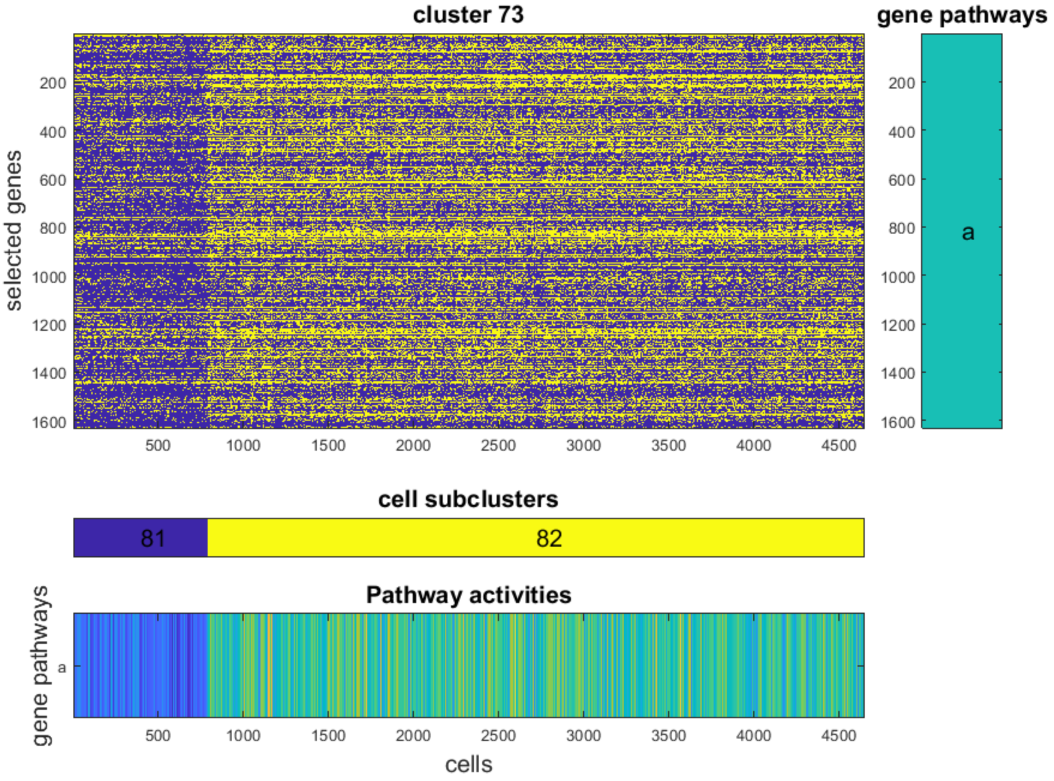


```
Remaining clusters to partition 9
Processing cluster 74 now ...
Processing data subset with 22327 genes and 6771 cells:
Remove genes detected in <100 cells. Remaining 15141 genes.  Elapsed time is 0.754330 seconds.
Iterate 10 random permutations for gene-gene similarity threshold ...   10 Elapsed time is 411.395470 seconds.
Compute gene-gene similarity ... Elapsed time is 30.082309 seconds.
Create gene-gene graph for clustering genes ...
Writing graph into file ... 100%Elapsed time is 123.107408 seconds.
Running ModularityOptimizer for clustering ...Elapsed time is 379.668992 seconds.
Gene-gene graph contains 5 pathways, 15078 genes in total
Elapsed time is 380.423262 seconds.
Create cell-cell graph for clustering cells ...
Writing graph into file ... 100%Elapsed time is 0.782117 seconds.
Running ModularityOptimizer for clustering ...Elapsed time is 3.962169 seconds.
Cell-cell graph contains 19 cell types by community detection
Elapsed time is 4.100355 seconds.
Cell-cell graph contains 16 cell types after merging tiny cell clusters
Cell-cell graph contains 2 cell types after merging
Number of useful pathways is 1
```

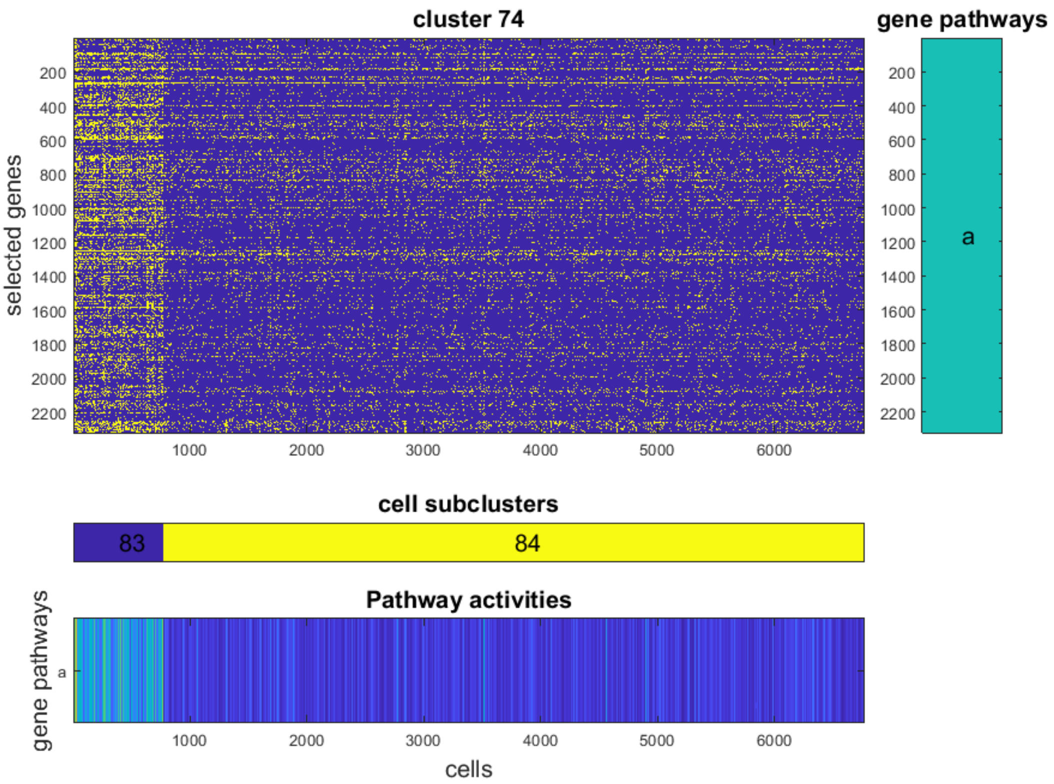


```
Remaining clusters to partition 10
Processing cluster 75 now ...
Processing data subset with 22327 genes and 10541 cells:
Remove genes detected in <100 cells. Remaining 13305 genes.  Elapsed time is 1.078854 seconds.
Iterate 10 random permutations for gene-gene similarity threshold ...   10 Elapsed time is 450.836370 seconds.
Compute gene-gene similarity ... Elapsed time is 30.960217 seconds.
Create gene-gene graph for clustering genes ...
Writing graph into file ... 100%Elapsed time is 15.674938 seconds.
Running ModularityOptimizer for clustering ...Elapsed time is 47.939891 seconds.
Gene-gene graph contains 8 pathways, 11788 genes in total
Elapsed time is 48.489128 seconds.
Create cell-cell graph for clustering cells ...
Writing graph into file ... 100%Elapsed time is 1.291516 seconds.
Running ModularityOptimizer for clustering ...Elapsed time is 7.348498 seconds.
Cell-cell graph contains 20 cell types by community detection
Elapsed time is 7.563129 seconds.
Cell-cell graph contains 19 cell types after merging tiny cell clusters
Cell-cell graph contains 3 cell types after merging
Number of useful pathways is 2
```

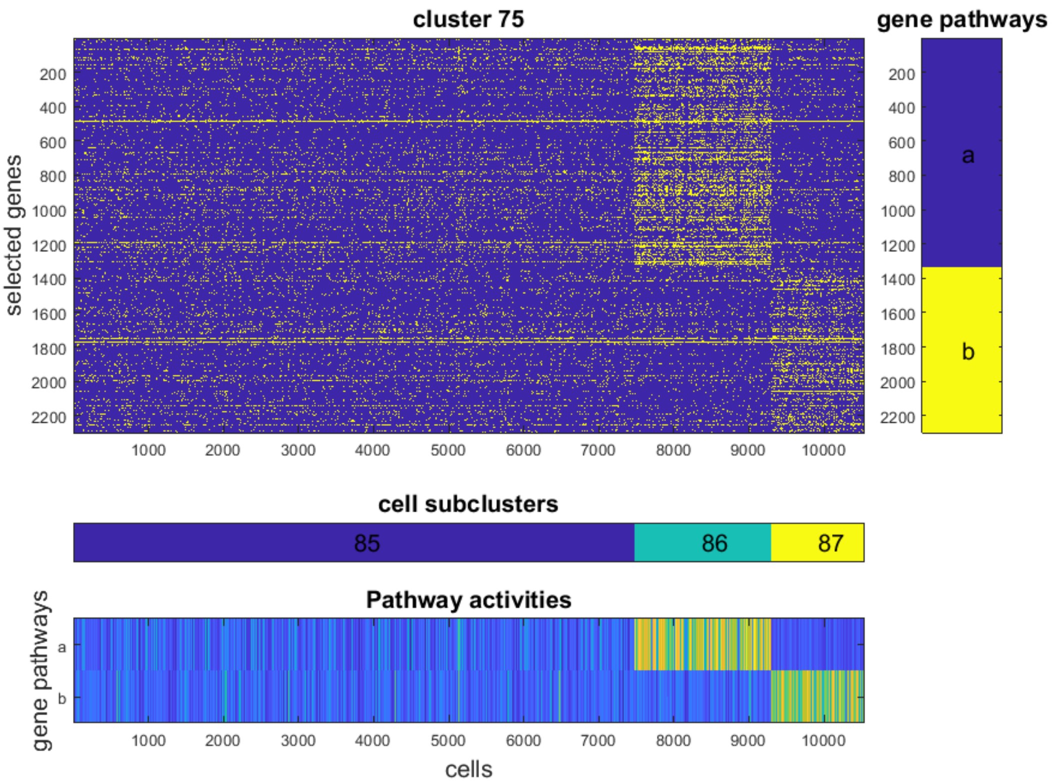


```
Remaining clusters to partition 12
Processing cluster 76 now ...
Processing data subset with 22327 genes and 652 cells:
Remove genes detected in <100 cells. Remaining 3553 genes.  Elapsed time is 0.047041 seconds.
Iterate 10 random permutations for gene-gene similarity threshold ...   10 Elapsed time is 10.656905 seconds.
Compute gene-gene similarity ... Elapsed time is 0.827372 seconds.
Create gene-gene graph for clustering genes ...
Writing graph into file ... 100%Elapsed time is 0.087992 seconds.
Running ModularityOptimizer for clustering ...Elapsed time is 0.399544 seconds.
Gene-gene graph contains 4 pathways, 167 genes in total
Elapsed time is 0.494935 seconds.
Create cell-cell graph for clustering cells ...
Writing graph into file ... 100%Elapsed time is 0.077936 seconds.
Running ModularityOptimizer for clustering ...Elapsed time is 0.547896 seconds.
Cell-cell graph contains 7 cell types by community detection
Elapsed time is 0.563477 seconds.
Cell-cell graph contains 7 cell types after merging tiny cell clusters
Cell-cell graph contains 2 cell types after merging
Number of useful pathways is 1
```

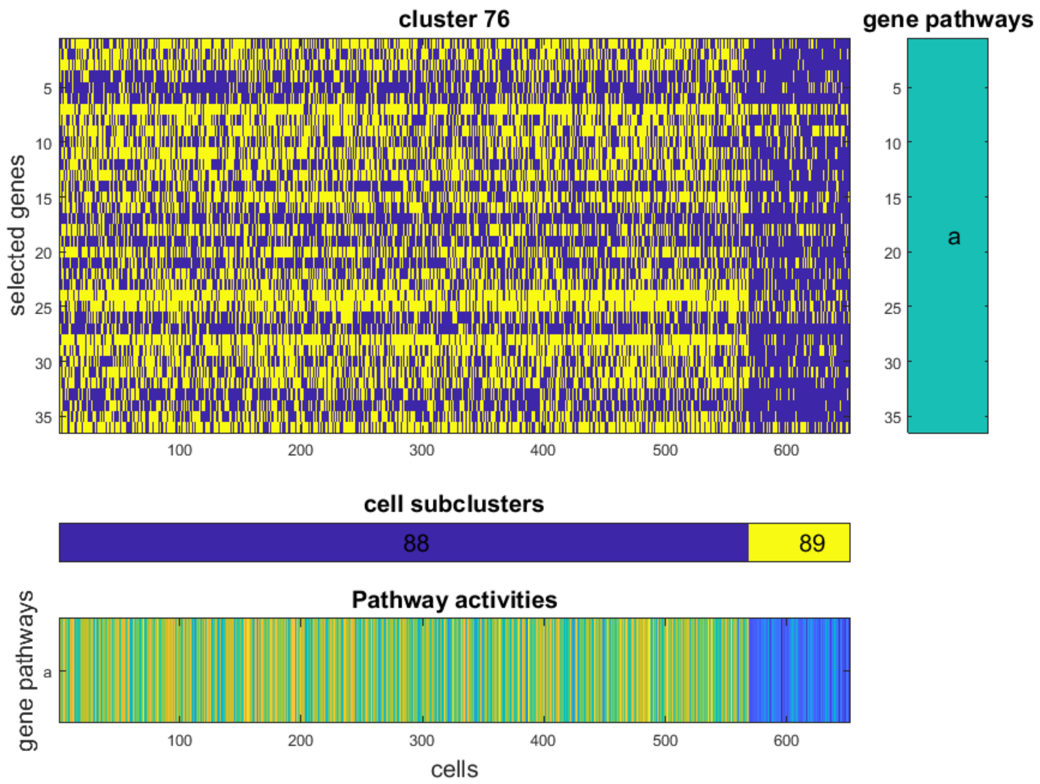


```
Remaining clusters to partition 13
Processing cluster 77 now ...
Processing data subset with 22327 genes and 26 cells:
Remove genes detected in <100 cells. Remaining 0 genes.  Elapsed time is 0.001438 seconds.

Remaining clusters to partition 12
Processing cluster 78 now ...
Processing data subset with 22327 genes and 88 cells:
Remove genes detected in <100 cells. Remaining 0 genes.  Elapsed time is 0.005190 seconds.

Remaining clusters to partition 11
Processing cluster 79 now ...
Processing data subset with 22327 genes and 1087 cells:
Remove genes detected in <100 cells. Remaining 6985 genes.  Elapsed time is 0.088207 seconds.
Iterate 10 random permutations for gene-gene similarity threshold ...   10 Elapsed time is 43.628832 seconds.
Compute gene-gene similarity ... Elapsed time is 3.298624 seconds.
Create gene-gene graph for clustering genes ...
Writing graph into file ... 100%Elapsed time is 1.687226 seconds.
Running ModularityOptimizer for clustering ...Elapsed time is 4.613976 seconds.
Gene-gene graph contains 4 pathways, 4167 genes in total
Elapsed time is 4.831142 seconds.
Create cell-cell graph for clustering cells ...
Writing graph into file ... 100%Elapsed time is 0.121312 seconds.
Running ModularityOptimizer for clustering ...Elapsed time is 0.663548 seconds.
Cell-cell graph contains 12 cell types by community detection
Elapsed time is 0.689719 seconds.
Cell-cell graph contains 12 cell types after merging tiny cell clusters
creating a total of 11 edges ...     11
Cell-cell graph contains 2 cell types after merging
Number of useful pathways is 1
```

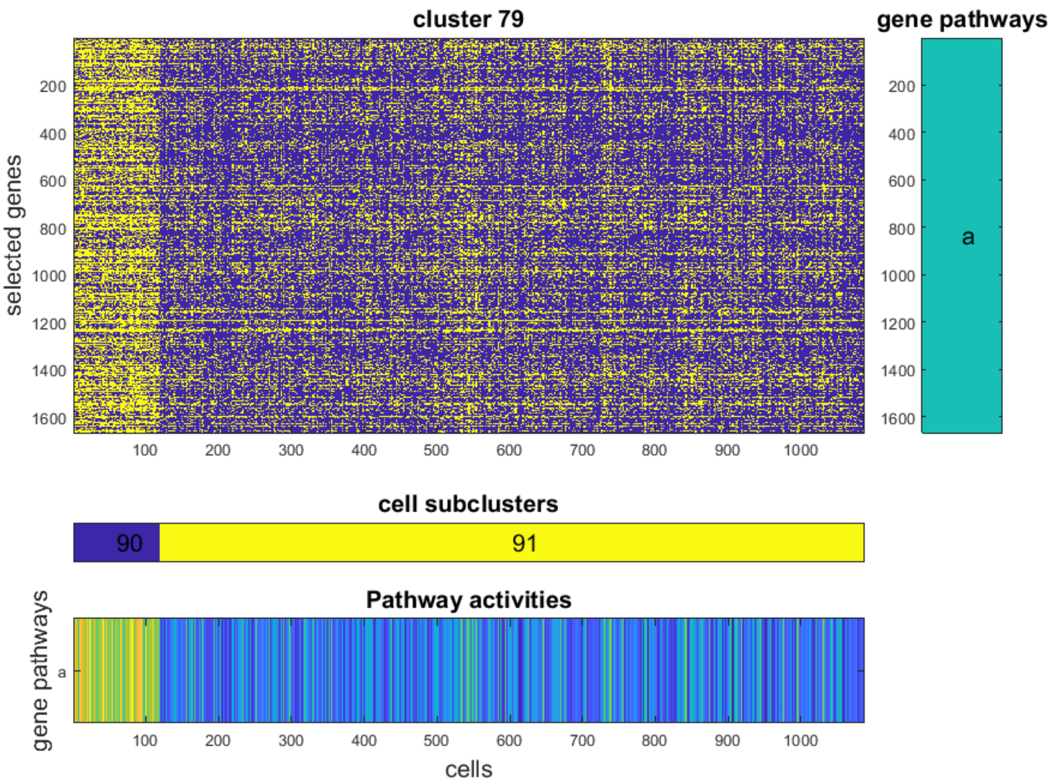


```
Remaining clusters to partition 12
Processing cluster 80 now ...
Processing data subset with 22327 genes and 74 cells:
Remove genes detected in <100 cells. Remaining 0 genes.  Elapsed time is 0.005002 seconds.

Remaining clusters to partition 11
Processing cluster 81 now ...
Processing data subset with 22327 genes and 789 cells:
Remove genes detected in <100 cells. Remaining 6045 genes.  Elapsed time is 0.062372 seconds.
Iterate 10 random permutations for gene-gene similarity threshold ...   10 Elapsed time is 30.548950 seconds.
Compute gene-gene similarity ... Elapsed time is 2.384769 seconds.
Create gene-gene graph for clustering genes ...
Writing graph into file ... 100%Elapsed time is 0.623003 seconds.
Running ModularityOptimizer for clustering ...Elapsed time is 1.772666 seconds.
Gene-gene graph contains 5 pathways, 3504 genes in total
Elapsed time is 1.958889 seconds.
Create cell-cell graph for clustering cells ...
Writing graph into file ... 100%Elapsed time is 0.087024 seconds.
Running ModularityOptimizer for clustering ...Elapsed time is 0.545718 seconds.
Cell-cell graph contains 10 cell types by community detection
Elapsed time is 0.564262 seconds.
Cell-cell graph contains 10 cell types after merging tiny cell clusters
creating a total of 9 edges ...      9
Cell-cell graph contains 1 cell types after merging

Remaining clusters to partition 10
Processing cluster 82 now ...
Processing data subset with 22327 genes and 3861 cells:
Remove genes detected in <100 cells. Remaining 10190 genes.  Elapsed time is 0.344778 seconds.
Iterate 10 random permutations for gene-gene similarity threshold ...   10 Elapsed time is 143.209745 seconds.
Compute gene-gene similarity ... Elapsed time is 10.030342 seconds.
Create gene-gene graph for clustering genes ...
Writing graph into file ... 100%Elapsed time is 0.752600 seconds.
Running ModularityOptimizer for clustering ...Elapsed time is 1.112103 seconds.
Gene-gene graph contains 9 pathways, 2721 genes in total
Elapsed time is 1.469949 seconds.
Create cell-cell graph for clustering cells ...
Writing graph into file ... 100%Elapsed time is 0.490509 seconds.
Running ModularityOptimizer for clustering ...Elapsed time is 3.090679 seconds.
Cell-cell graph contains 10 cell types by community detection
Elapsed time is 3.170728 seconds.
Cell-cell graph contains 9 cell types after merging tiny cell clusters
Cell-cell graph contains 2 cell types after merging
Number of useful pathways is 1
```

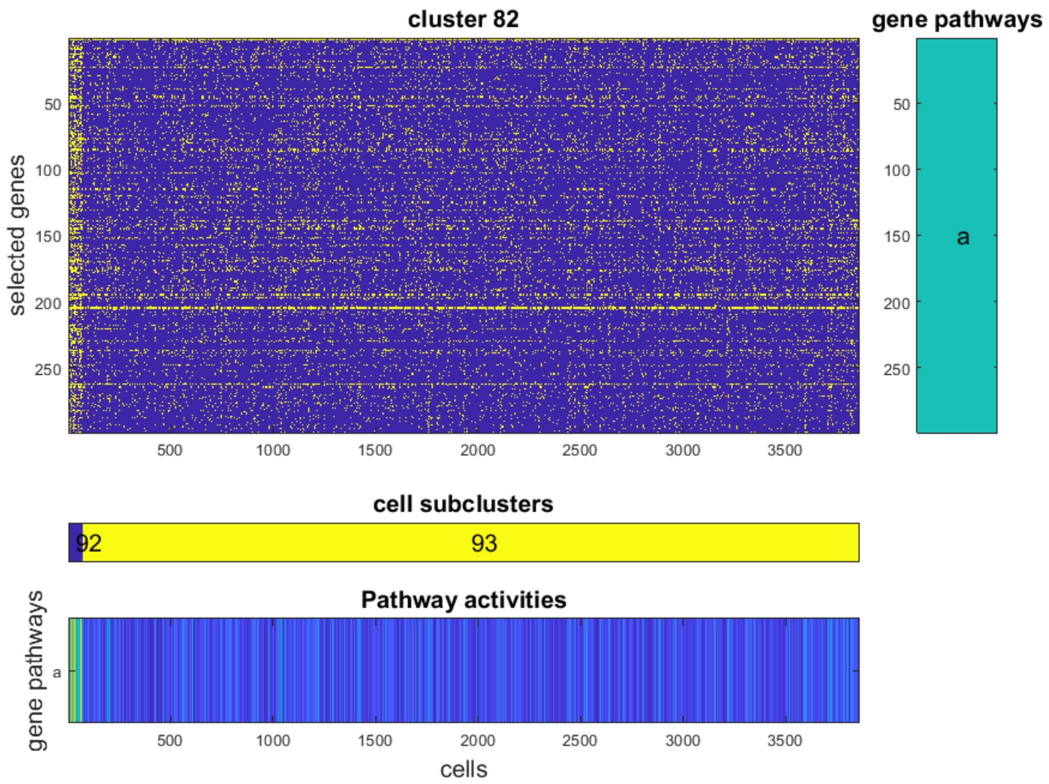


```
Remaining clusters to partition 11
Processing cluster 83 now ...
Processing data subset with 22327 genes and 771 cells:
Remove genes detected in <100 cells. Remaining 9372 genes.  Elapsed time is 0.078165 seconds.
Iterate 10 random permutations for gene-gene similarity threshold ...   10 Elapsed time is 70.130816 seconds.
Compute gene-gene similarity ... Elapsed time is 5.278919 seconds.
Create gene-gene graph for clustering genes ...
Writing graph into file ... 100%Elapsed time is 23.605842 seconds.
Running ModularityOptimizer for clustering ...Elapsed time is 62.253160 seconds.
Gene-gene graph contains 4 pathways, 9030 genes in total
Elapsed time is 62.581843 seconds.
Create cell-cell graph for clustering cells ...
Writing graph into file ... 100%Elapsed time is 0.099124 seconds.
Running ModularityOptimizer for clustering ...Elapsed time is 0.501639 seconds.
Cell-cell graph contains 11 cell types by community detection
Elapsed time is 0.519166 seconds.
Cell-cell graph contains 10 cell types after merging tiny cell clusters
Cell-cell graph contains 2 cell types after merging
Number of useful pathways is 1
```

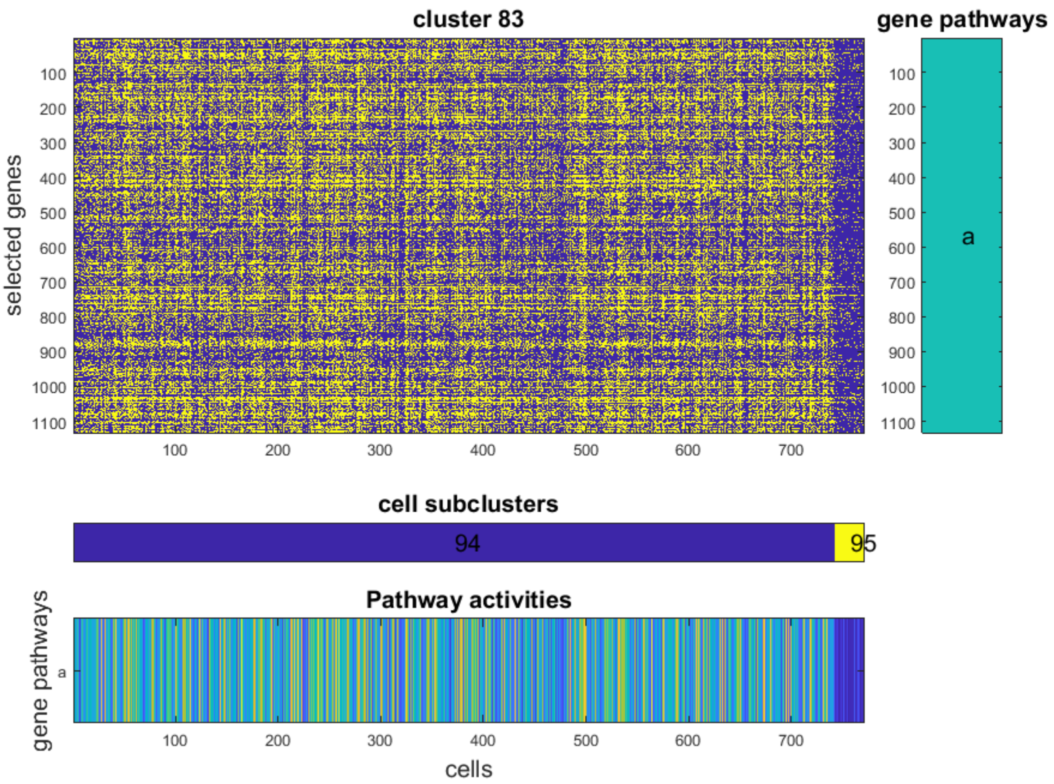


```
Remaining clusters to partition 12
Processing cluster 84 now ...
Processing data subset with 22327 genes and 6000 cells:
Remove genes detected in <100 cells. Remaining 14442 genes.  Elapsed time is 0.606149 seconds.
Iterate 10 random permutations for gene-gene similarity threshold ...   10 Elapsed time is 348.908935 seconds.
Compute gene-gene similarity ... Elapsed time is 25.224502 seconds.
Create gene-gene graph for clustering genes ...
Writing graph into file ... 100%Elapsed time is 96.471950 seconds.
Running ModularityOptimizer for clustering ...Elapsed time is 513.646296 seconds.
Gene-gene graph contains 4 pathways, 14344 genes in total
Elapsed time is 514.287117 seconds.
Create cell-cell graph for clustering cells ...
Writing graph into file ... 100%Elapsed time is 0.679062 seconds.
Running ModularityOptimizer for clustering ...Elapsed time is 3.198216 seconds.
Cell-cell graph contains 21 cell types by community detection
Elapsed time is 3.325209 seconds.
Cell-cell graph contains 19 cell types after merging tiny cell clusters
creating a total of 18 edges ...     18
Cell-cell graph contains 2 cell types after merging
Number of useful pathways is 1
```

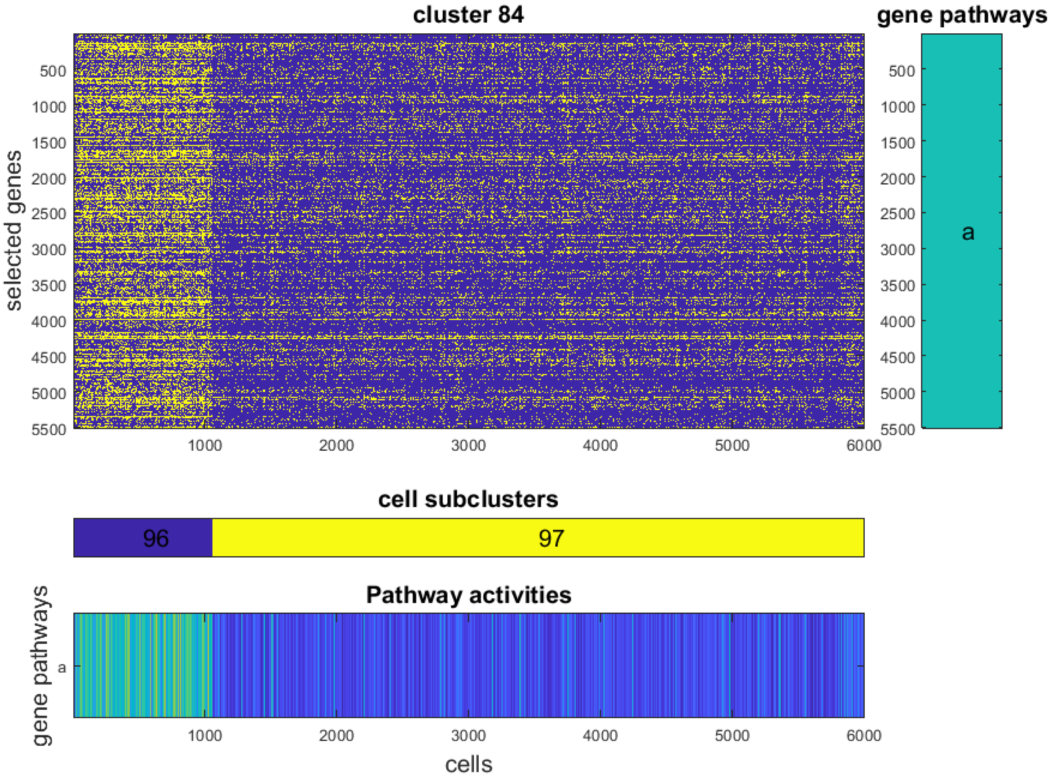


```
Remaining clusters to partition 13
Processing cluster 85 now ...
Processing data subset with 22327 genes and 7467 cells:
Remove genes detected in <100 cells. Remaining 12466 genes.  Elapsed time is 0.797083 seconds.
Iterate 10 random permutations for gene-gene similarity threshold ...   10 Elapsed time is 311.166380 seconds.
Compute gene-gene similarity ... Elapsed time is 21.749844 seconds.
Create gene-gene graph for clustering genes ...
Writing graph into file ... 100%Elapsed time is 12.164625 seconds.
Running ModularityOptimizer for clustering ...Elapsed time is 37.976194 seconds.
Gene-gene graph contains 7 pathways, 10615 genes in total
Elapsed time is 38.482102 seconds.
Create cell-cell graph for clustering cells ...
Writing graph into file ... 100%Elapsed time is 0.879956 seconds.
Running ModularityOptimizer for clustering ...Elapsed time is 4.505577 seconds.
Cell-cell graph contains 21 cell types by community detection
Elapsed time is 4.659485 seconds.
Cell-cell graph contains 18 cell types after merging tiny cell clusters
Cell-cell graph contains 4 cell types after merging
Number of useful pathways is 3
```

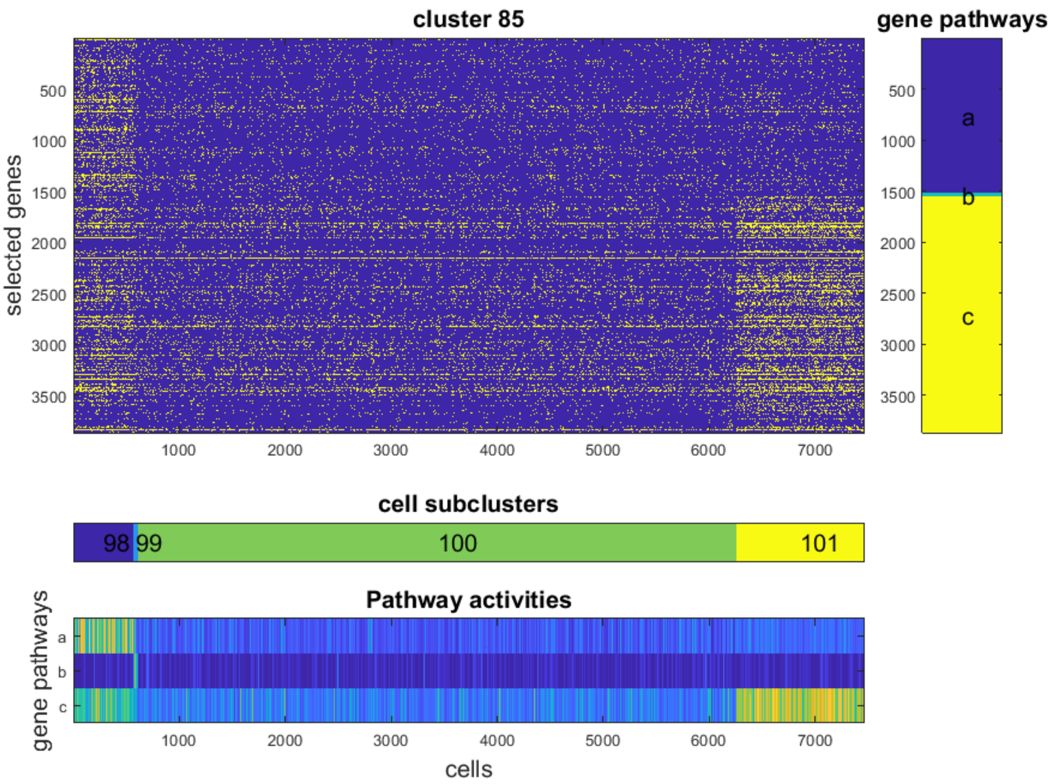


```
Remaining clusters to partition 16
Processing cluster 86 now ...
Processing data subset with 22327 genes and 1819 cells:
Remove genes detected in <100 cells. Remaining 6986 genes.  Elapsed time is 0.147824 seconds.
Iterate 10 random permutations for gene-gene similarity threshold ...   10 Elapsed time is 51.102417 seconds.
Compute gene-gene similarity ... Elapsed time is 3.609223 seconds.
Create gene-gene graph for clustering genes ...
Writing graph into file ... 100%Elapsed time is 0.583066 seconds.
Running ModularityOptimizer for clustering ...Elapsed time is 1.342002 seconds.
Gene-gene graph contains 9 pathways, 3059 genes in total
Elapsed time is 1.562338 seconds.
Create cell-cell graph for clustering cells ...
Writing graph into file ... 100%Elapsed time is 0.218846 seconds.
Running ModularityOptimizer for clustering ...Elapsed time is 1.235313 seconds.
Cell-cell graph contains 10 cell types by community detection
Elapsed time is 1.275848 seconds.
Cell-cell graph contains 10 cell types after merging tiny cell clusters
Cell-cell graph contains 2 cell types after merging
Number of useful pathways is 1
```

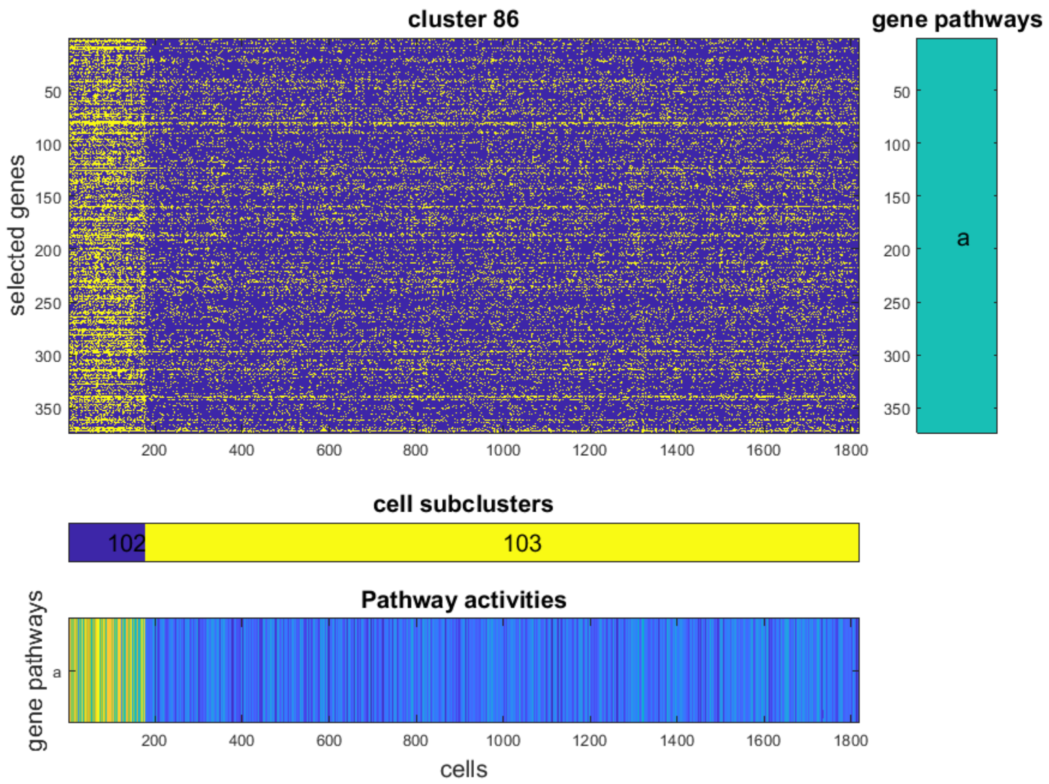


```
Remaining clusters to partition 17
Processing cluster 87 now ...
Processing data subset with 22327 genes and 1255 cells:
Remove genes detected in <100 cells. Remaining 4636 genes.  Elapsed time is 0.096117 seconds.
Iterate 10 random permutations for gene-gene similarity threshold ...   10 Elapsed time is 21.124820 seconds.
Compute gene-gene similarity ... Elapsed time is 1.516505 seconds.
Create gene-gene graph for clustering genes ...
Writing graph into file ... 100%Elapsed time is 1.527429 seconds.
Running ModularityOptimizer for clustering ...Elapsed time is 2.643498 seconds.
Gene-gene graph contains 3 pathways, 3041 genes in total
Elapsed time is 2.783174 seconds.
Create cell-cell graph for clustering cells ...
Writing graph into file ... 100%Elapsed time is 0.143486 seconds.
Running ModularityOptimizer for clustering ...Elapsed time is 0.783608 seconds.
Cell-cell graph contains 11 cell types by community detection
Elapsed time is 0.811030 seconds.
Cell-cell graph contains 10 cell types after merging tiny cell clusters
creating a total of 9 edges ...       9
Cell-cell graph contains 2 cell types after merging
Number of useful pathways is 1
```

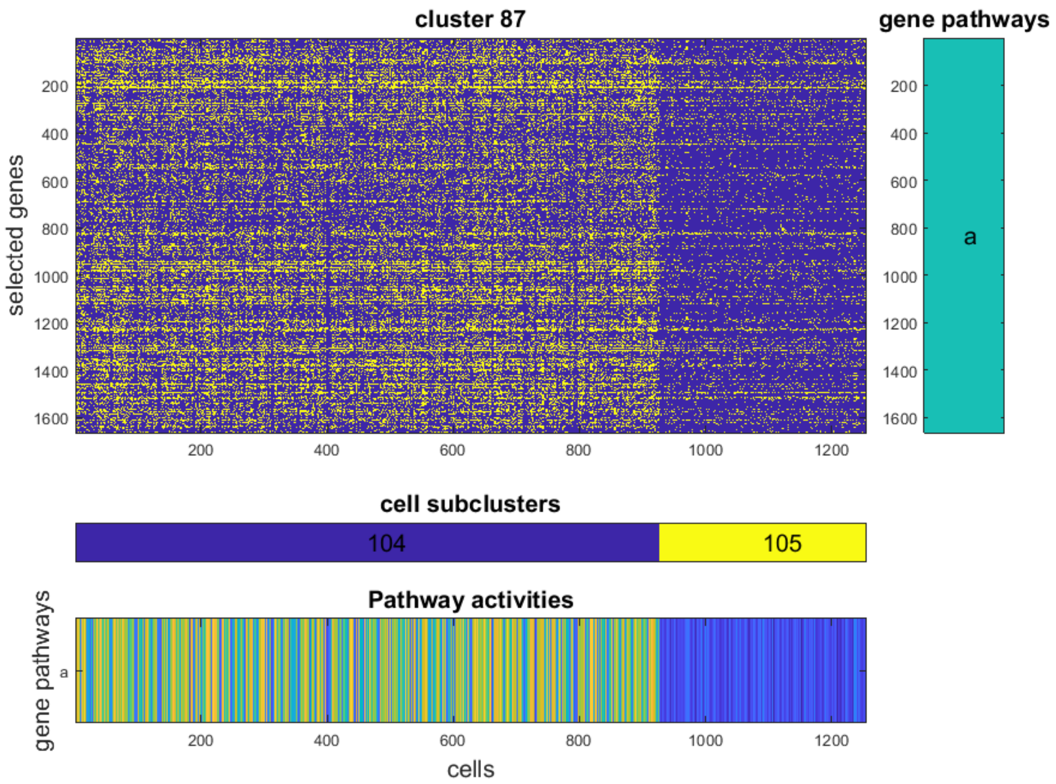


```
Remaining clusters to partition 18
Processing cluster 88 now ...
Processing data subset with 22327 genes and 568 cells:
Remove genes detected in <100 cells. Remaining 2985 genes.  Elapsed time is 0.039115 seconds.
Iterate 10 random permutations for gene-gene similarity threshold ...   10 Elapsed time is 7.491297 seconds.
Compute gene-gene similarity ... Elapsed time is 0.546401 seconds.
Create gene-gene graph for clustering genes ...
Writing graph into file ... 100%Elapsed time is 0.060188 seconds.
Running ModularityOptimizer for clustering ...Elapsed time is 0.408640 seconds.
Gene-gene graph contains 2 pathways, 48 genes in total
Elapsed time is 0.484742 seconds.
Create cell-cell graph for clustering cells ...
Writing graph into file ... 100%Elapsed time is 0.065758 seconds.
Running ModularityOptimizer for clustering ...Elapsed time is 0.476602 seconds.
Cell-cell graph contains 7 cell types by community detection
Elapsed time is 0.491439 seconds.
Cell-cell graph contains 2 cell types after merging tiny cell clusters
creating a total of 1 edges ...      1
Cell-cell graph contains 1 cell types after merging

Remaining clusters to partition 17
Processing cluster 89 now ...
Processing data subset with 22327 genes and 84 cells:
Remove genes detected in <100 cells. Remaining 0 genes.  Elapsed time is 0.005771 seconds.

Remaining clusters to partition 16
Processing cluster 90 now ...
Processing data subset with 22327 genes and 119 cells:
Remove genes detected in <100 cells. Remaining 0 genes.  Elapsed time is 0.006778 seconds.

Remaining clusters to partition 15
Processing cluster 91 now ...
Processing data subset with 22327 genes and 968 cells:
Remove genes detected in <100 cells. Remaining 6337 genes.  Elapsed time is 0.076380 seconds.
Iterate 10 random permutations for gene-gene similarity threshold ...   10 Elapsed time is 34.761320 seconds.
Compute gene-gene similarity ... Elapsed time is 2.741098 seconds.
Create gene-gene graph for clustering genes ...
Writing graph into file ... 100%Elapsed time is 0.862740 seconds.
Running ModularityOptimizer for clustering ...Elapsed time is 2.327216 seconds.
Gene-gene graph contains 5 pathways, 3395 genes in total
Elapsed time is 2.518814 seconds.
Create cell-cell graph for clustering cells ...
Writing graph into file ... 100%Elapsed time is 0.109772 seconds.
Running ModularityOptimizer for clustering ...Elapsed time is 0.680932 seconds.
Cell-cell graph contains 10 cell types by community detection
Elapsed time is 0.702525 seconds.
Cell-cell graph contains 10 cell types after merging tiny cell clusters
creating a total of 9 edges ...      9
Cell-cell graph contains 2 cell types after merging
Number of useful pathways is 1
```

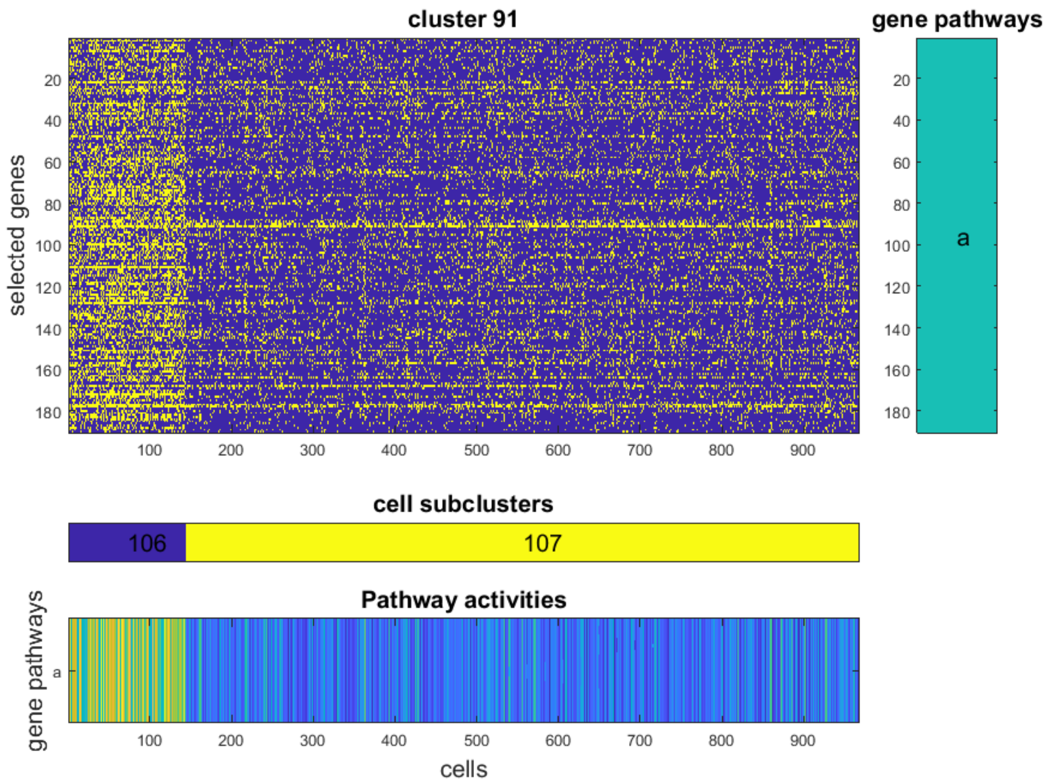


```
Remaining clusters to partition 16
Processing cluster 92 now ...
Processing data subset with 22327 genes and 69 cells:
Remove genes detected in <100 cells. Remaining 0 genes.  Elapsed time is 0.004413 seconds.

Remaining clusters to partition 15
Processing cluster 93 now ...
Processing data subset with 22327 genes and 3792 cells:
Remove genes detected in <100 cells. Remaining 10121 genes.  Elapsed time is 0.341283 seconds.
Iterate 10 random permutations for gene-gene similarity threshold ...   10 Elapsed time is 140.900300 seconds.
Compute gene-gene similarity ... Elapsed time is 10.070007 seconds.
Create gene-gene graph for clustering genes ...
Writing graph into file ... 100%Elapsed time is 0.717007 seconds.
Running ModularityOptimizer for clustering ...Elapsed time is 1.111210 seconds.
Gene-gene graph contains 9 pathways, 2622 genes in total
Elapsed time is 1.472553 seconds.
Create cell-cell graph for clustering cells ...
Writing graph into file ... 100%Elapsed time is 0.488433 seconds.
Running ModularityOptimizer for clustering ...Elapsed time is 2.970327 seconds.
Cell-cell graph contains 8 cell types by community detection
Elapsed time is 3.049553 seconds.
Cell-cell graph contains 6 cell types after merging tiny cell clusters
creating a total of 5 edges ...       5
Cell-cell graph contains 1 cell types after merging

Remaining clusters to partition 14
Processing cluster 94 now ...
Processing data subset with 22327 genes and 742 cells:
Remove genes detected in <100 cells. Remaining 9252 genes.  Elapsed time is 0.064963 seconds.
Iterate 10 random permutations for gene-gene similarity threshold ...   10 Elapsed time is 67.086382 seconds.
Compute gene-gene similarity ... Elapsed time is 5.247689 seconds.
Create gene-gene graph for clustering genes ...
Writing graph into file ... 100%Elapsed time is 23.411187 seconds.
Running ModularityOptimizer for clustering ...Elapsed time is 76.259574 seconds.
Gene-gene graph contains 4 pathways, 8855 genes in total
Elapsed time is 76.582789 seconds.
Create cell-cell graph for clustering cells ...
Writing graph into file ... 100%Elapsed time is 0.082045 seconds.
Running ModularityOptimizer for clustering ...Elapsed time is 0.488373 seconds.
Cell-cell graph contains 10 cell types by community detection
Elapsed time is 0.505726 seconds.
Cell-cell graph contains 10 cell types after merging tiny cell clusters
creating a total of 9 edges ...       9
Cell-cell graph contains 2 cell types after merging
Number of useful pathways is 1
```

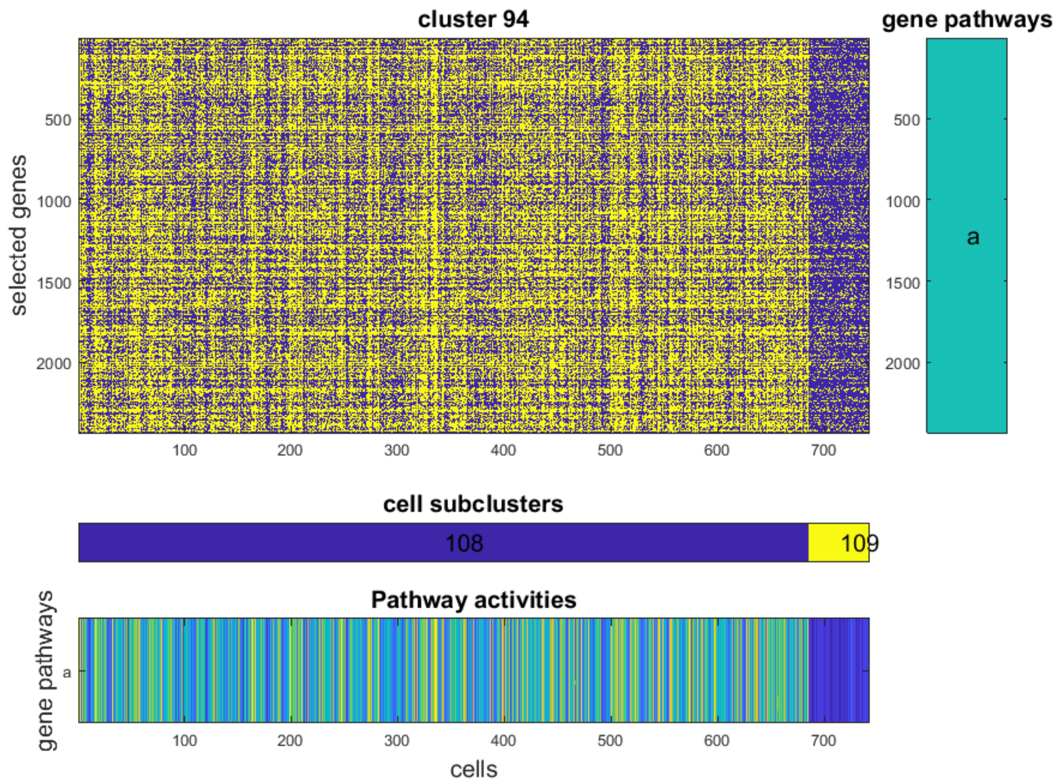


```
Remaining clusters to partition 15
Processing cluster 95 now ...
Processing data subset with 22327 genes and 29 cells:
Remove genes detected in <100 cells. Remaining 0 genes.  Elapsed time is 0.001380 seconds.

Remaining clusters to partition 14
Processing cluster 96 now ...
Processing data subset with 22327 genes and 1054 cells:
Remove genes detected in <100 cells. Remaining 10184 genes.  Elapsed time is 0.095682 seconds.
Iterate 10 random permutations for gene-gene similarity threshold ...   10 Elapsed time is 88.310891 seconds.
Compute gene-gene similarity ... Elapsed time is 6.616570 seconds.
Create gene-gene graph for clustering genes ...
Writing graph into file ... 100%Elapsed time is 4.910530 seconds.
Running ModularityOptimizer for clustering ...Elapsed time is 9.877995 seconds.
Gene-gene graph contains 4 pathways, 7445 genes in total
Elapsed time is 10.241582 seconds.
Create cell-cell graph for clustering cells ...
Writing graph into file ... 100%Elapsed time is 0.123746 seconds.
Running ModularityOptimizer for clustering ...Elapsed time is 0.587073 seconds.
Cell-cell graph contains 12 cell types by community detection
Elapsed time is 0.610783 seconds.
Cell-cell graph contains 11 cell types after merging tiny cell clusters
creating a total of 10 edges ...     10
Cell-cell graph contains 1 cell types after merging

Remaining clusters to partition 13
Processing cluster 97 now ...
Processing data subset with 22327 genes and 4946 cells:
Remove genes detected in <100 cells. Remaining 13373 genes.  Elapsed time is 0.497633 seconds.
Iterate 10 random permutations for gene-gene similarity threshold ...   10 Elapsed time is 274.335064 seconds.
Compute gene-gene similarity ... Elapsed time is 19.944548 seconds.
Create gene-gene graph for clustering genes ...
Writing graph into file ... 100%Elapsed time is 30.688461 seconds.
Running ModularityOptimizer for clustering ...Elapsed time is 96.158700 seconds.
Gene-gene graph contains 7 pathways, 13046 genes in total
Elapsed time is 96.711805 seconds.
Create cell-cell graph for clustering cells ...
Writing graph into file ... 100%Elapsed time is 0.586588 seconds.
Running ModularityOptimizer for clustering ...Elapsed time is 2.872680 seconds.
Cell-cell graph contains 17 cell types by community detection
Elapsed time is 2.981097 seconds.
Cell-cell graph contains 16 cell types after merging tiny cell clusters
Cell-cell graph contains 3 cell types after merging
Number of useful pathways is 2
```

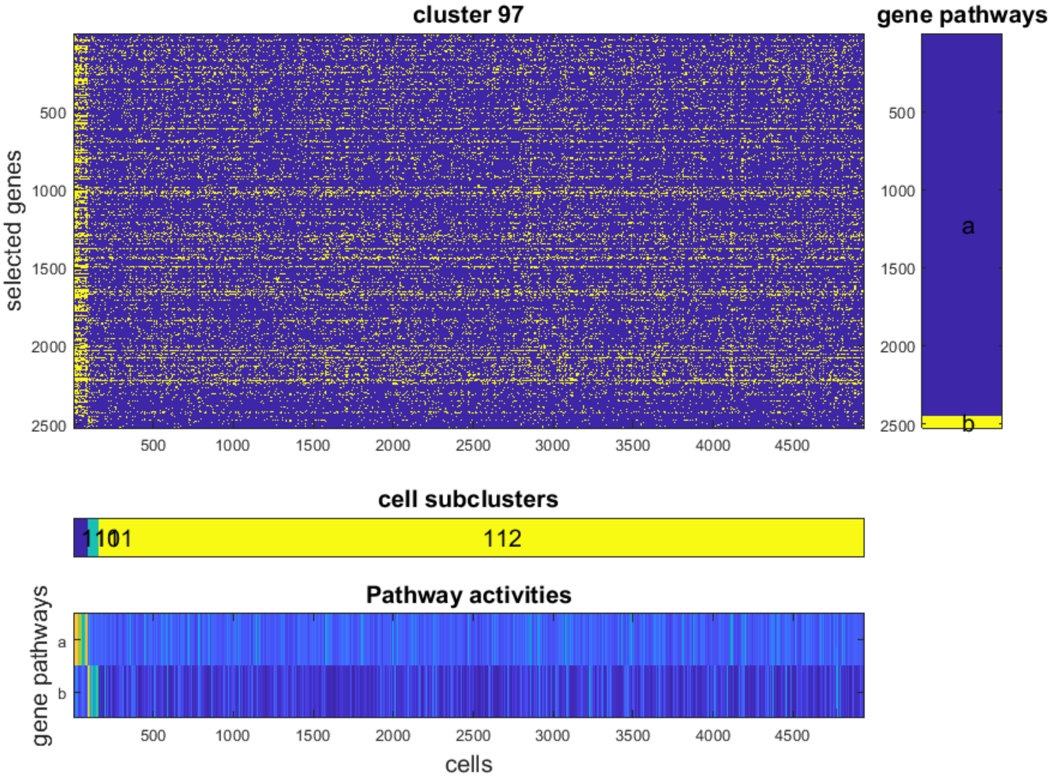


```
Remaining clusters to partition 15
Processing cluster 98 now ...
Processing data subset with 22327 genes and 561 cells:
Remove genes detected in <100 cells. Remaining 3585 genes.  Elapsed time is 0.040285 seconds.
Iterate 10 random permutations for gene-gene similarity threshold ...   10 Elapsed time is 10.421764 seconds.
Compute gene-gene similarity ... Elapsed time is 0.816818 seconds.
Create gene-gene graph for clustering genes ...
Writing graph into file ... 100%Elapsed time is 0.285513 seconds.
Running ModularityOptimizer for clustering ...Elapsed time is 0.810107 seconds.
Gene-gene graph contains 2 pathways, 1389 genes in total
Elapsed time is 0.909087 seconds.
Create cell-cell graph for clustering cells ...
Writing graph into file ... 100%Elapsed time is 0.063366 seconds.
Running ModularityOptimizer for clustering ...Elapsed time is 0.462909 seconds.
Cell-cell graph contains 10 cell types by community detection
Elapsed time is 0.477889 seconds.
Cell-cell graph contains 2 cell types after merging tiny cell clusters
creating a total of 1 edges ...      1
Cell-cell graph contains 1 cell types after merging

Remaining clusters to partition 14
Processing cluster 99 now ...
Processing data subset with 22327 genes and 53 cells:
Remove genes detected in <100 cells. Remaining 0 genes.  Elapsed time is 0.003597 seconds.

Remaining clusters to partition 13
Processing cluster 100 now ...
Processing data subset with 22327 genes and 5641 cells:
Remove genes detected in <100 cells. Remaining 11151 genes.  Elapsed time is 0.569177 seconds.
Iterate 10 random permutations for gene-gene similarity threshold ...   10 Elapsed time is 211.846259 seconds.
Compute gene-gene similarity ... Elapsed time is 15.364859 seconds.
Create gene-gene graph for clustering genes ...
Writing graph into file ... 100%Elapsed time is 6.831009 seconds.
Running ModularityOptimizer for clustering ...Elapsed time is 21.242271 seconds.
Gene-gene graph contains 4 pathways, 8494 genes in total
Elapsed time is 21.660750 seconds.
Create cell-cell graph for clustering cells ...
Writing graph into file ... 100%Elapsed time is 0.650374 seconds.
Running ModularityOptimizer for clustering ...Elapsed time is 3.079715 seconds.
Cell-cell graph contains 15 cell types by community detection
Elapsed time is 3.194240 seconds.
Cell-cell graph contains 12 cell types after merging tiny cell clusters
creating a total of 11 edges ...     11
Cell-cell graph contains 2 cell types after merging
Number of useful pathways is 1
```

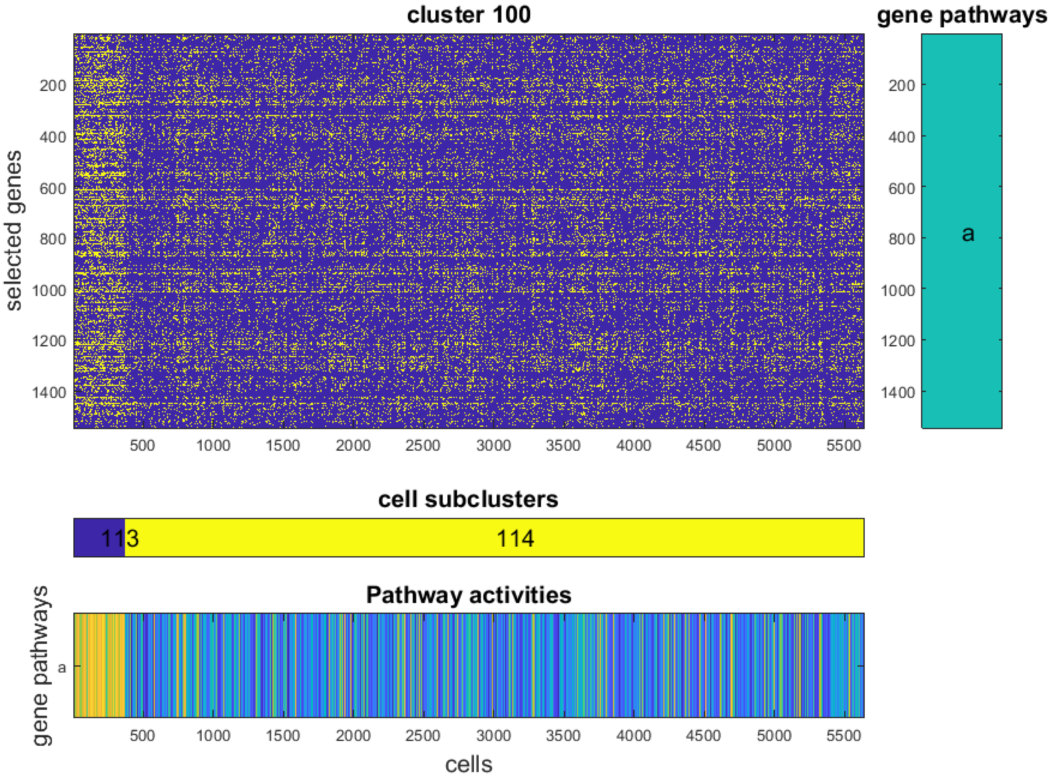


```
Remaining clusters to partition 14
Processing cluster 101 now ...
Processing data subset with 22327 genes and 1212 cells:
Remove genes detected in <100 cells. Remaining 5896 genes.  Elapsed time is 0.094135 seconds.
Iterate 10 random permutations for gene-gene similarity threshold ...   10 Elapsed time is 32.205251 seconds.
Compute gene-gene similarity ... Elapsed time is 2.368437 seconds.
Create gene-gene graph for clustering genes ...
Writing graph into file ... 100%Elapsed time is 0.854483 seconds.
Running ModularityOptimizer for clustering ...Elapsed time is 1.772021 seconds.
Gene-gene graph contains 4 pathways, 2721 genes in total
Elapsed time is 1.946156 seconds.
Create cell-cell graph for clustering cells ...
Writing graph into file ... 100%Elapsed time is 0.180826 seconds.
Running ModularityOptimizer for clustering ...Elapsed time is 0.702531 seconds.
Cell-cell graph contains 10 cell types by community detection
Elapsed time is 0.730950 seconds.
Cell-cell graph contains 10 cell types after merging tiny cell clusters
creating a total of 9 edges ...      9
Cell-cell graph contains 1 cell types after merging

Remaining clusters to partition 13
Processing cluster 102 now ...
Processing data subset with 22327 genes and 176 cells:
Remove genes detected in <100 cells. Remaining 0 genes.  Elapsed time is 0.010538 seconds.

Remaining clusters to partition 12
Processing cluster 103 now ...
Processing data subset with 22327 genes and 1643 cells:
Remove genes detected in <100 cells. Remaining 6508 genes.  Elapsed time is 0.137182 seconds.
Iterate 10 random permutations for gene-gene similarity threshold ...   10 Elapsed time is 42.798800 seconds.
Compute gene-gene similarity ... Elapsed time is 3.093257 seconds.
Create gene-gene graph for clustering genes ...
Writing graph into file ... 100%Elapsed time is 0.471356 seconds.
Running ModularityOptimizer for clustering ...Elapsed time is 1.225529 seconds.
Gene-gene graph contains 7 pathways, 2613 genes in total
Elapsed time is 1.420772 seconds.
Create cell-cell graph for clustering cells ...
Writing graph into file ... 100%Elapsed time is 0.195483 seconds.
Running ModularityOptimizer for clustering ...Elapsed time is 1.123827 seconds.
Cell-cell graph contains 9 cell types by community detection
Elapsed time is 1.161478 seconds.
Cell-cell graph contains 9 cell types after merging tiny cell clusters
creating a total of 8 edges ...      8
Cell-cell graph contains 2 cell types after merging
Number of useful pathways is 1
```

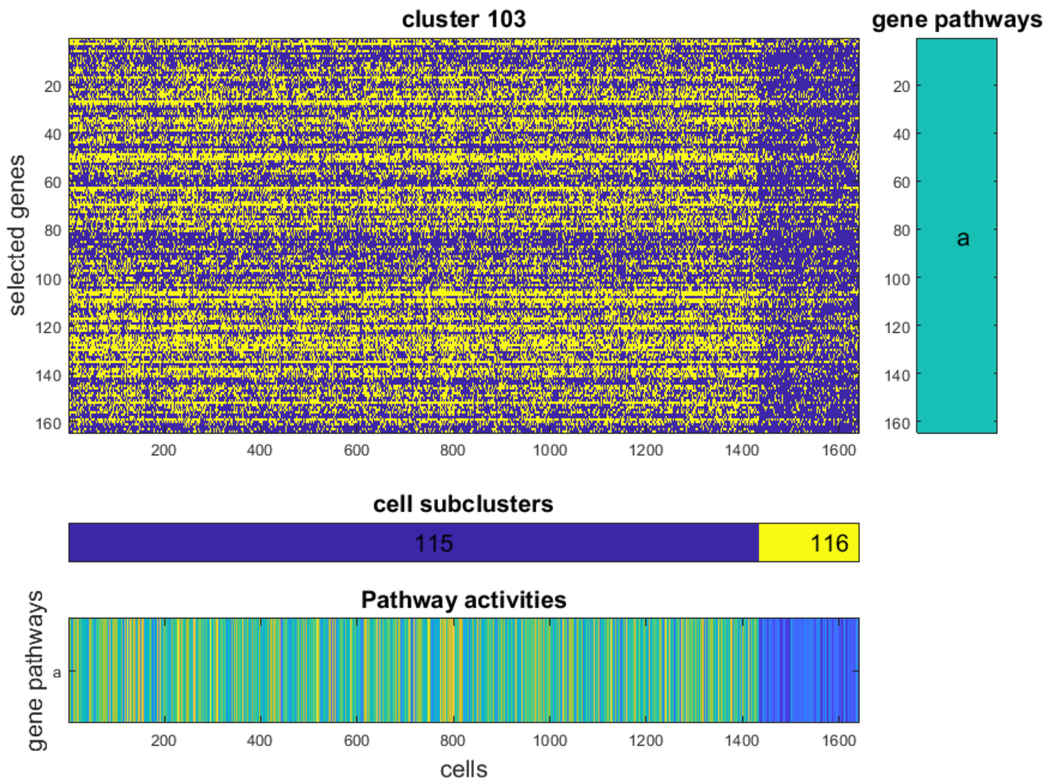


```
Remaining clusters to partition 13
Processing cluster 104 now ...
Processing data subset with 22327 genes and 926 cells:
Remove genes detected in <100 cells. Remaining 3606 genes.  Elapsed time is 0.066058 seconds.
Iterate 10 random permutations for gene-gene similarity threshold ...   10 Elapsed time is 12.103176 seconds.
Compute gene-gene similarity ... Elapsed time is 0.842468 seconds.
Create gene-gene graph for clustering genes ...
Writing graph into file ... 100%Elapsed time is 0.354370 seconds.
Running ModularityOptimizer for clustering ...Elapsed time is 0.900747 seconds.
Gene-gene graph contains 4 pathways, 1754 genes in total
Elapsed time is 0.999000 seconds.
Create cell-cell graph for clustering cells ...
Writing graph into file ... 100%Elapsed time is 0.105138 seconds.
Running ModularityOptimizer for clustering ...Elapsed time is 0.630250 seconds.
Cell-cell graph contains 11 cell types by community detection
Elapsed time is 0.651907 seconds.
Cell-cell graph contains 9 cell types after merging tiny cell clusters
Cell-cell graph contains 4 cell types after merging
Number of useful pathways is 3
```

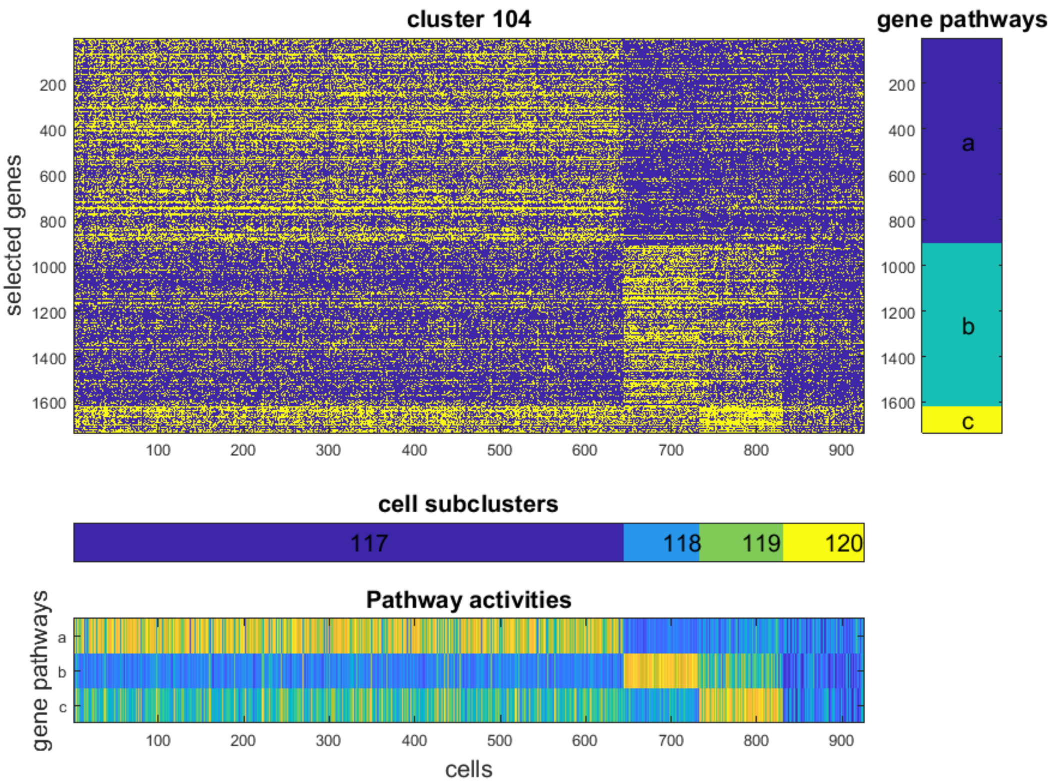


```
Remaining clusters to partition 16
Processing cluster 105 now ...
Processing data subset with 22327 genes and 329 cells:
Remove genes detected in <100 cells. Remaining 743 genes.  Elapsed time is 0.020358 seconds.
Iterate 10 random permutations for gene-gene similarity threshold ...   10 Elapsed time is 0.502497 seconds.
Compute gene-gene similarity ... Elapsed time is 0.030383 seconds.
Create gene-gene graph for clustering genes ...
Writing graph into file ... 100%Elapsed time is 0.018445 seconds.
Running ModularityOptimizer for clustering ...Elapsed time is 0.317425 seconds.
Gene-gene graph contains 5 pathways, 316 genes in total
Elapsed time is 0.337942 seconds.
Create cell-cell graph for clustering cells ...
Writing graph into file ... 100%Elapsed time is 0.037257 seconds.
Running ModularityOptimizer for clustering ...Elapsed time is 0.329074 seconds.
Cell-cell graph contains 5 cell types by community detection
Elapsed time is 0.338767 seconds.
Cell-cell graph contains 5 cell types after merging tiny cell clusters
creating a total of 4 edges ...       4
Cell-cell graph contains 1 cell types after merging

Remaining clusters to partition 15
Processing cluster 106 now ...
Processing data subset with 22327 genes and 143 cells:
Remove genes detected in <100 cells. Remaining 0 genes.  Elapsed time is 0.009242 seconds.

Remaining clusters to partition 14
Processing cluster 107 now ...
Processing data subset with 22327 genes and 825 cells:
Remove genes detected in <100 cells. Remaining 5495 genes.  Elapsed time is 0.068046 seconds.
Iterate 10 random permutations for gene-gene similarity threshold ...   10 Elapsed time is 25.325818 seconds.
Compute gene-gene similarity ... Elapsed time is 1.914710 seconds.
Create gene-gene graph for clustering genes ...
Writing graph into file ... 100%Elapsed time is 0.669678 seconds.
Running ModularityOptimizer for clustering ...Elapsed time is 1.879334 seconds.
Gene-gene graph contains 4 pathways, 2950 genes in total
Elapsed time is 2.041526 seconds.
Create cell-cell graph for clustering cells ...
Writing graph into file ... 100%Elapsed time is 0.092882 seconds.
Running ModularityOptimizer for clustering ...Elapsed time is 0.532048 seconds.
Cell-cell graph contains 9 cell types by community detection
Elapsed time is 0.551447 seconds.
Cell-cell graph contains 9 cell types after merging tiny cell clusters
creating a total of 8 edges ...       8
Cell-cell graph contains 2 cell types after merging
Number of useful pathways is 1
```

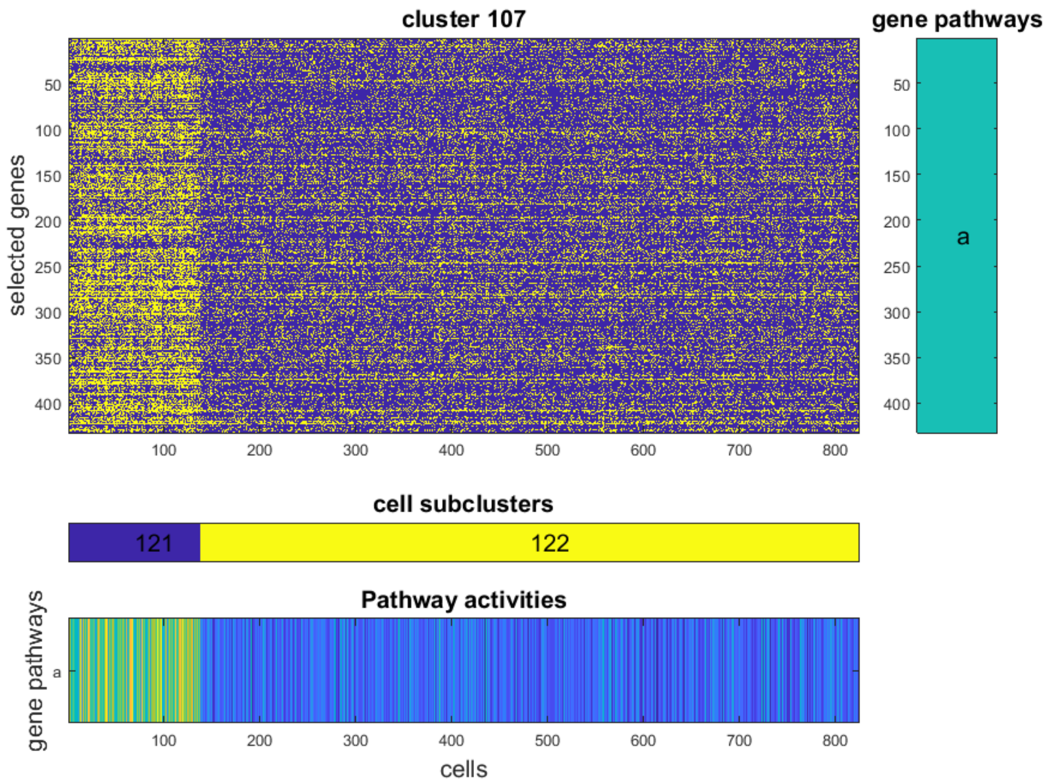


```
Remaining clusters to partition 15
Processing cluster 108 now ...
Processing data subset with 22327 genes and 685 cells:
Remove genes detected in <100 cells. Remaining 9066 genes.  Elapsed time is 0.060608 seconds.
Iterate 10 random permutations for gene-gene similarity threshold ...   10 Elapsed time is 63.952148 seconds.
Compute gene-gene similarity ... Elapsed time is 4.897681 seconds.
Create gene-gene graph for clustering genes ...
Writing graph into file ... 100%Elapsed time is 16.198604 seconds.
Running ModularityOptimizer for clustering ...Elapsed time is 55.051492 seconds.
Gene-gene graph contains 3 pathways, 8462 genes in total
Elapsed time is 55.365060 seconds.
Create cell-cell graph for clustering cells ...
Writing graph into file ... 100%Elapsed time is 0.075337 seconds.
Running ModularityOptimizer for clustering ...Elapsed time is 0.441639 seconds.
Cell-cell graph contains 10 cell types by community detection
Elapsed time is 0.458008 seconds.
Cell-cell graph contains 9 cell types after merging tiny cell clusters
Cell-cell graph contains 3 cell types after merging
Number of useful pathways is 3
```

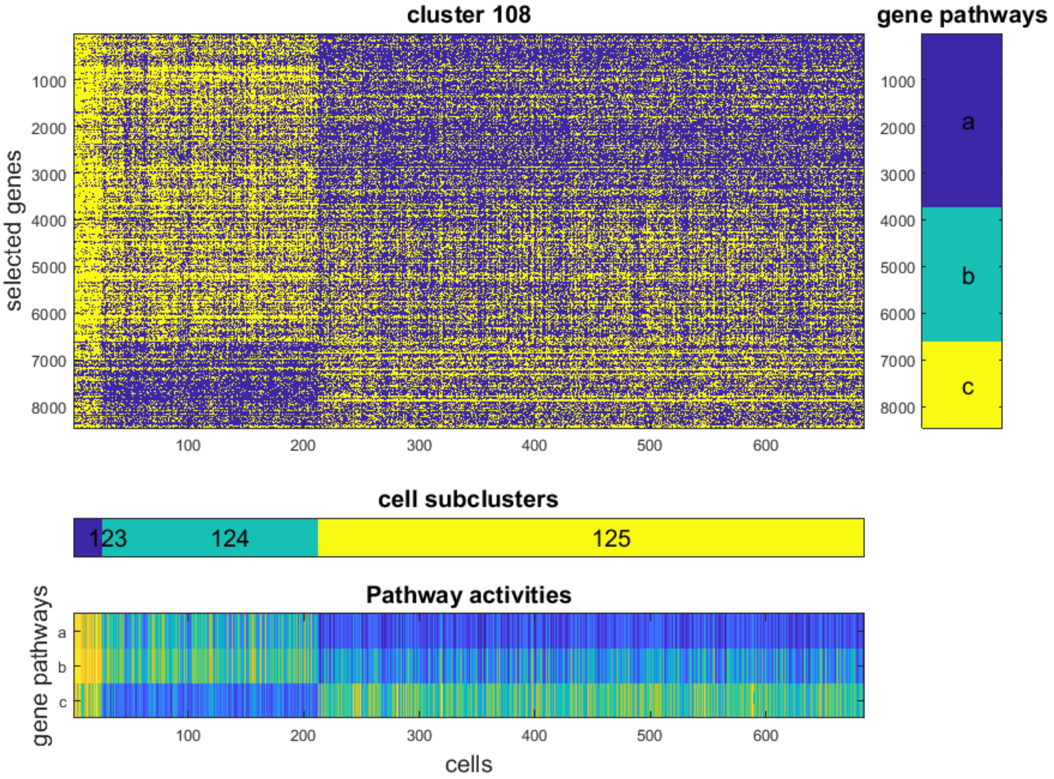


```
Remaining clusters to partition 17
Processing cluster 109 now ...
Processing data subset with 22327 genes and 57 cells:
Remove genes detected in <100 cells. Remaining 0 genes.  Elapsed time is 0.003821 seconds.

Remaining clusters to partition 16
Processing cluster 110 now ...
Processing data subset with 22327 genes and 88 cells:
Remove genes detected in <100 cells. Remaining 0 genes.  Elapsed time is 0.005178 seconds.

Remaining clusters to partition 15
Processing cluster 111 now ...
Processing data subset with 22327 genes and 74 cells:
Remove genes detected in <100 cells. Remaining 0 genes.  Elapsed time is 0.004872 seconds.

Remaining clusters to partition 14
Processing cluster 112 now ...
Processing data subset with 22327 genes and 4784 cells:
Remove genes detected in <100 cells. Remaining 13138 genes.  Elapsed time is 0.483772 seconds.
Iterate 10 random permutations for gene-gene similarity threshold ...   10 Elapsed time is 259.994385 seconds.
Compute gene-gene similarity ... Elapsed time is 18.835411 seconds.
Create gene-gene graph for clustering genes ...
Writing graph into file ... 100%Elapsed time is 26.425416 seconds.
Running ModularityOptimizer for clustering ...Elapsed time is 76.910628 seconds.
Gene-gene graph contains 6 pathways, 12622 genes in total
Elapsed time is 77.450718 seconds.
Create cell-cell graph for clustering cells ...
Writing graph into file ... 100%Elapsed time is 0.553399 seconds.
Running ModularityOptimizer for clustering ...Elapsed time is 2.654126 seconds.
Cell-cell graph contains 16 cell types by community detection
Elapsed time is 2.752308 seconds.
Cell-cell graph contains 15 cell types after merging tiny cell clusters
creating a total of 14 edges ...     14
Cell-cell graph contains 2 cell types after merging
Number of useful pathways is 1
```

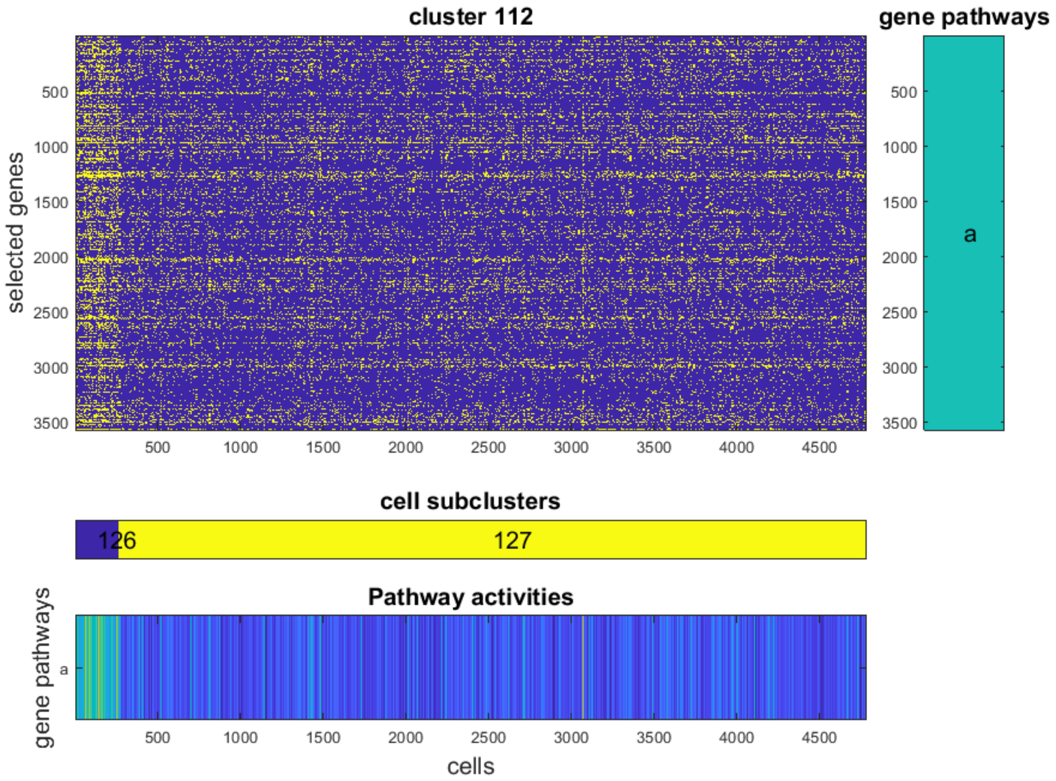


```
Remaining clusters to partition 15
Processing cluster 113 now ...
Processing data subset with 22327 genes and 372 cells:
Remove genes detected in <100 cells. Remaining 2433 genes.  Elapsed time is 0.026547 seconds.
Iterate 10 random permutations for gene-gene similarity threshold ...   10 Elapsed time is 4.556133 seconds.
Compute gene-gene similarity ... Elapsed time is 0.342495 seconds.
Create gene-gene graph for clustering genes ...
Writing graph into file ... 100%Elapsed time is 0.055347 seconds.
Running ModularityOptimizer for clustering ...Elapsed time is 0.348393 seconds.
Gene-gene graph contains 3 pathways, 394 genes in total
Elapsed time is 0.409801 seconds.
Create cell-cell graph for clustering cells ...
Writing graph into file ... 100%Elapsed time is 0.041651 seconds.
Running ModularityOptimizer for clustering ...Elapsed time is 0.331981 seconds.
Cell-cell graph contains 8 cell types by community detection
Elapsed time is 0.341952 seconds.
Cell-cell graph contains 8 cell types after merging tiny cell clusters
creating a total of 7 edges ...      7
Cell-cell graph contains 1 cell types after merging

Remaining clusters to partition 14
Processing cluster 114 now ...
Processing data subset with 22327 genes and 5269 cells:
Remove genes detected in <100 cells. Remaining 10934 genes.  Elapsed time is 0.499142 seconds.
Iterate 10 random permutations for gene-gene similarity threshold ...   10 Elapsed time is 196.630259 seconds.
Compute gene-gene similarity ... Elapsed time is 13.782633 seconds.
Create gene-gene graph for clustering genes ...
Writing graph into file ... 100%Elapsed time is 6.083325 seconds.
Running ModularityOptimizer for clustering ...Elapsed time is 16.866150 seconds.
Gene-gene graph contains 4 pathways, 8278 genes in total
Elapsed time is 17.270567 seconds.
Create cell-cell graph for clustering cells ...
Writing graph into file ... 100%Elapsed time is 0.608251 seconds.
Running ModularityOptimizer for clustering ...Elapsed time is 2.875711 seconds.
Cell-cell graph contains 14 cell types by community detection
Elapsed time is 2.986197 seconds.
Cell-cell graph contains 12 cell types after merging tiny cell clusters
creating a total of 11 edges ...     11
Cell-cell graph contains 2 cell types after merging
Number of useful pathways is 1
```

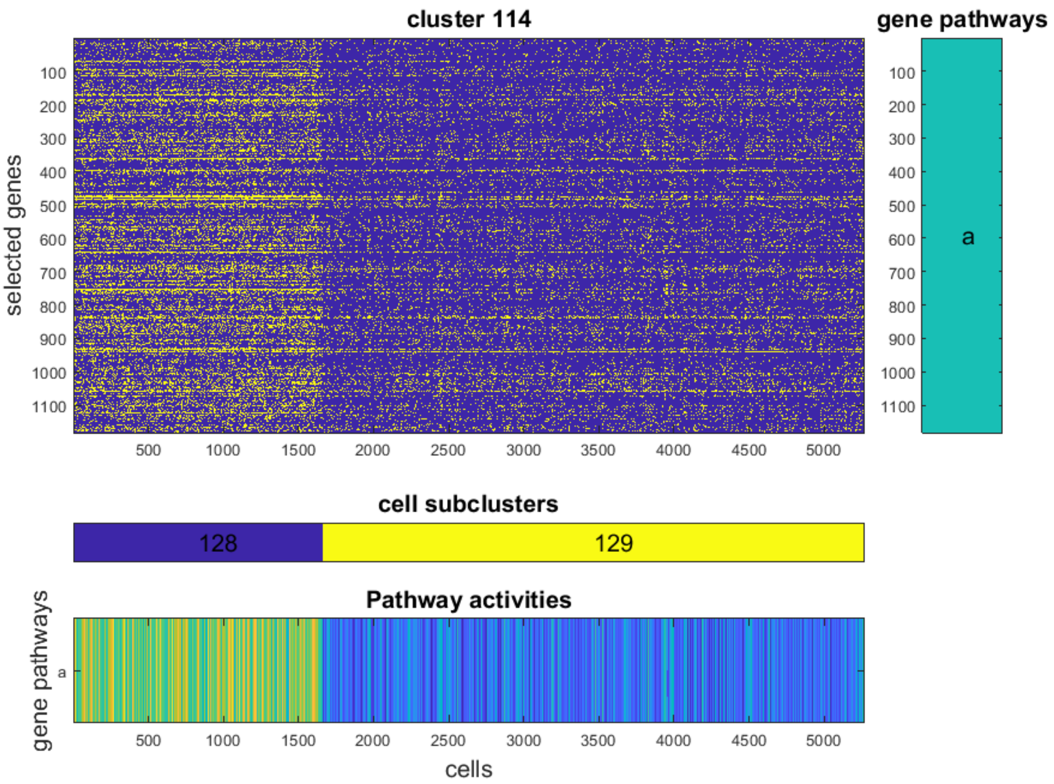


```
Remaining clusters to partition 15
Processing cluster 115 now ...
Processing data subset with 22327 genes and 1433 cells:
Remove genes detected in <100 cells. Remaining 6176 genes.  Elapsed time is 0.115478 seconds.
Iterate 10 random permutations for gene-gene similarity threshold ...   10 Elapsed time is 37.324853 seconds.
Compute gene-gene similarity ... Elapsed time is 2.677413 seconds.
Create gene-gene graph for clustering genes ...
Writing graph into file ... 100%Elapsed time is 0.383817 seconds.
Running ModularityOptimizer for clustering ...Elapsed time is 0.852214 seconds.
Gene-gene graph contains 7 pathways, 2054 genes in total
Elapsed time is 1.034576 seconds.
Create cell-cell graph for clustering cells ...
Writing graph into file ... 100%Elapsed time is 0.178345 seconds.
Running ModularityOptimizer for clustering ...Elapsed time is 0.928943 seconds.
Cell-cell graph contains 11 cell types by community detection
Elapsed time is 0.960203 seconds.
Cell-cell graph contains 10 cell types after merging tiny cell clusters
creating a total of 9 edges ...      9
Cell-cell graph contains 2 cell types after merging
Number of useful pathways is 1
```

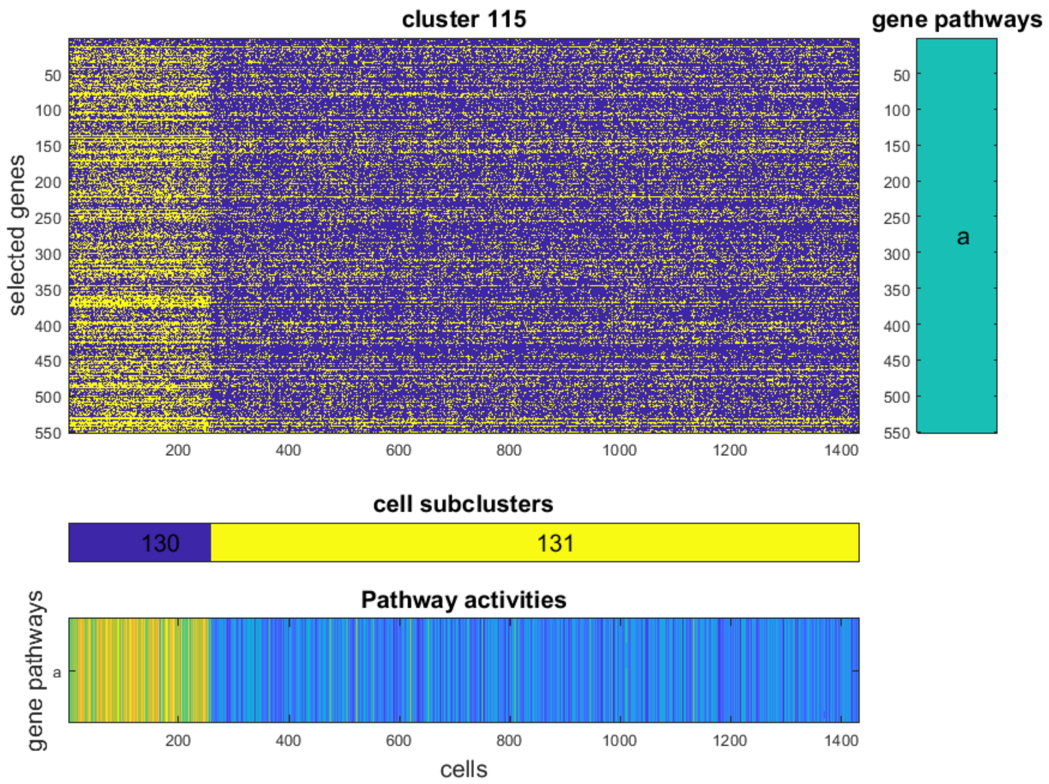


```
Remaining clusters to partition 16
Processing cluster 116 now ...
Processing data subset with 22327 genes and 210 cells:
Remove genes detected in <100 cells. Remaining 38 genes.  Elapsed time is 0.013660 seconds.
Iterate 10 random permutations for gene-gene similarity threshold ...   10 Elapsed time is 0.010402 seconds.
Compute gene-gene similarity ... Elapsed time is 0.000418 seconds.
Create gene-gene graph for clustering genes ...
Writing graph into file ... 102%Elapsed time is 0.001696 seconds.
Running ModularityOptimizer for clustering ...Elapsed time is 0.163760 seconds.
Gene-gene graph contains 0 pathways, 0 genes in total
Elapsed time is 0.167335 seconds.

Remaining clusters to partition 15
Processing cluster 117 now ...
Processing data subset with 22327 genes and 644 cells:
Remove genes detected in <100 cells. Remaining 2451 genes.  Elapsed time is 0.064091 seconds.
Iterate 10 random permutations for gene-gene similarity threshold ...   10 Elapsed time is 5.704443 seconds.
Compute gene-gene similarity ... Elapsed time is 0.405730 seconds.
Create gene-gene graph for clustering genes ...
Writing graph into file ... 100%Elapsed time is 0.047783 seconds.
Running ModularityOptimizer for clustering ...Elapsed time is 0.400335 seconds.
Gene-gene graph contains 5 pathways, 298 genes in total
Elapsed time is 0.461658 seconds.
Create cell-cell graph for clustering cells ...
Writing graph into file ... 100%Elapsed time is 0.075957 seconds.
Running ModularityOptimizer for clustering ...Elapsed time is 0.528090 seconds.
Cell-cell graph contains 6 cell types by community detection
Elapsed time is 0.543565 seconds.
Cell-cell graph contains 6 cell types after merging tiny cell clusters
Cell-cell graph contains 2 cell types after merging
Number of useful pathways is 2
```

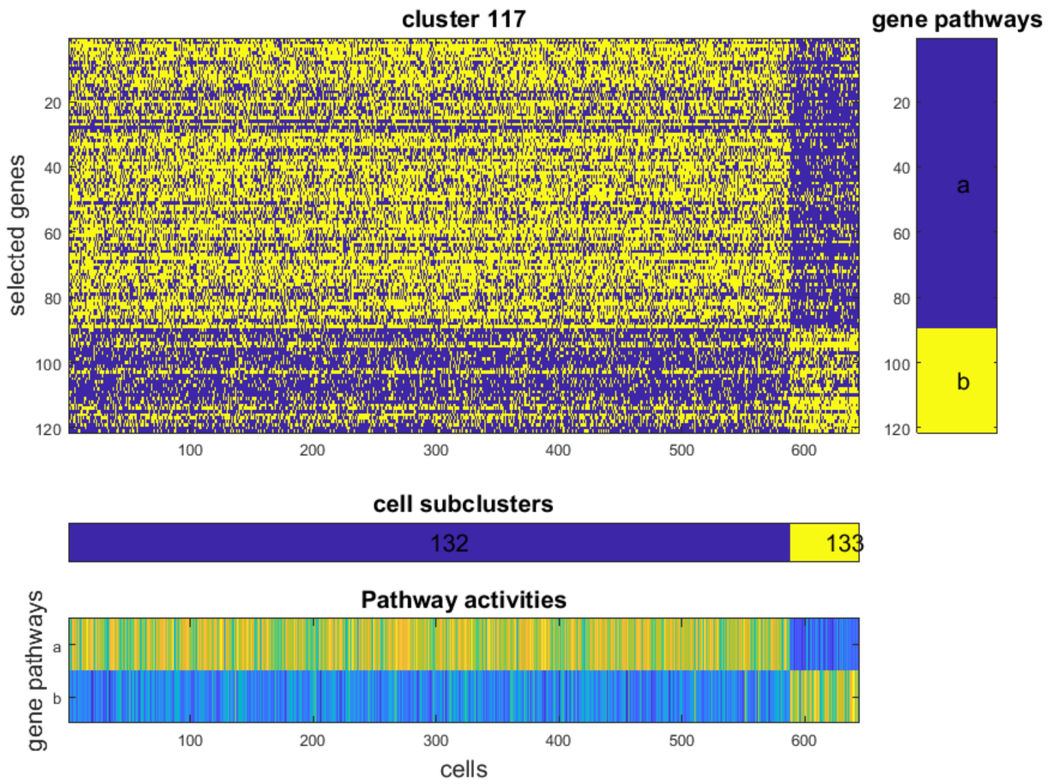


```
Remaining clusters to partition 16
Processing cluster 118 now ...
Processing data subset with 22327 genes and 89 cells:
Remove genes detected in <100 cells. Remaining 0 genes.  Elapsed time is 0.006086 seconds.

Remaining clusters to partition 15
Processing cluster 119 now ...
Processing data subset with 22327 genes and 97 cells:
Remove genes detected in <100 cells. Remaining 0 genes.  Elapsed time is 0.006608 seconds.

Remaining clusters to partition 14
Processing cluster 120 now ...
Processing data subset with 22327 genes and 96 cells:
Remove genes detected in <100 cells. Remaining 0 genes.  Elapsed time is 0.006779 seconds.

Remaining clusters to partition 13
Processing cluster 121 now ...
Processing data subset with 22327 genes and 137 cells:
Remove genes detected in <100 cells. Remaining 0 genes.  Elapsed time is 0.009076 seconds.

Remaining clusters to partition 12
Processing cluster 122 now ...
Processing data subset with 22327 genes and 688 cells:
Remove genes detected in <100 cells. Remaining 4582 genes.  Elapsed time is 0.056859 seconds.
Iterate 10 random permutations for gene-gene similarity threshold ...   10 Elapsed time is 17.482145 seconds.
Compute gene-gene similarity ... Elapsed time is 1.289440 seconds.
Create gene-gene graph for clustering genes ...
Writing graph into file ... 100%Elapsed time is 0.514181 seconds.
Running ModularityOptimizer for clustering ...Elapsed time is 1.220890 seconds.
Gene-gene graph contains 4 pathways, 2367 genes in total
Elapsed time is 1.351103 seconds.
Create cell-cell graph for clustering cells ...
Writing graph into file ... 100%Elapsed time is 0.078605 seconds.
Running ModularityOptimizer for clustering ...Elapsed time is 0.528946 seconds.
Cell-cell graph contains 7 cell types by community detection
Elapsed time is 0.545203 seconds.
Cell-cell graph contains 7 cell types after merging tiny cell clusters
Cell-cell graph contains 2 cell types after merging
Number of useful pathways is 1
```

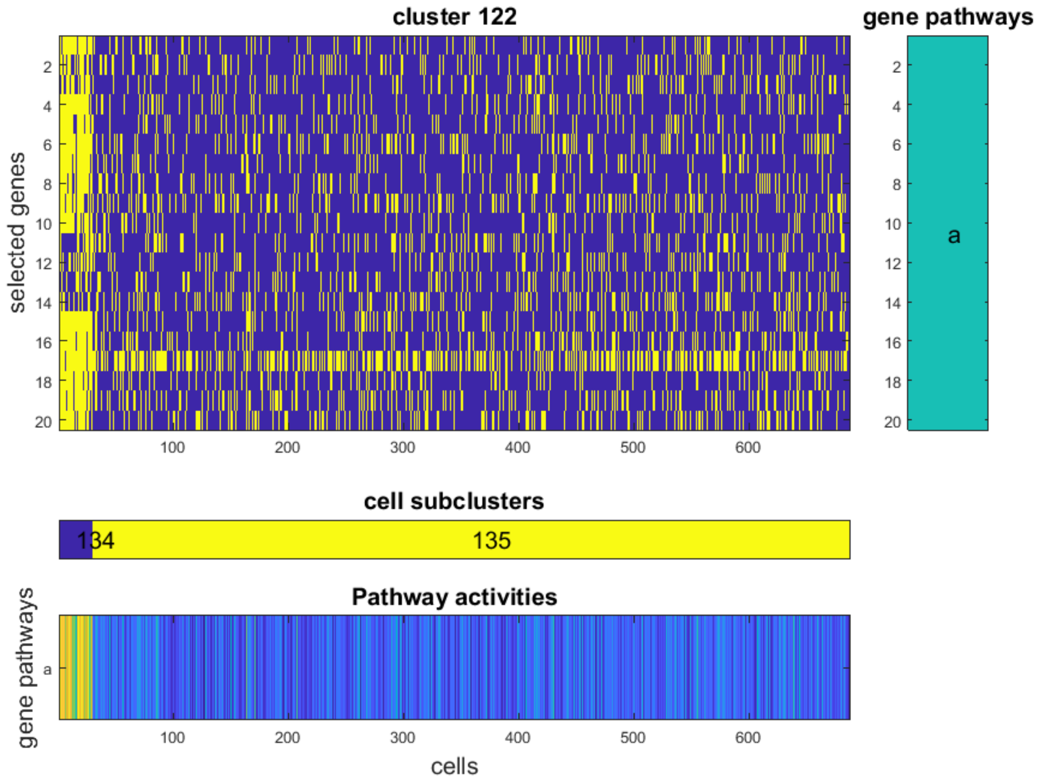


```
Remaining clusters to partition 13
Processing cluster 123 now ...
Processing data subset with 22327 genes and 25 cells:
Remove genes detected in <100 cells. Remaining 0 genes.  Elapsed time is 0.001250 seconds.

Remaining clusters to partition 12
Processing cluster 124 now ...
Processing data subset with 22327 genes and 187 cells:
Remove genes detected in <100 cells. Remaining 0 genes.  Elapsed time is 0.012218 seconds.

Remaining clusters to partition 11
Processing cluster 125 now ...
Processing data subset with 22327 genes and 473 cells:
Remove genes detected in <100 cells. Remaining 5991 genes.  Elapsed time is 0.037515 seconds.
Iterate 10 random permutations for gene-gene similarity threshold ...   10 Elapsed time is 26.926860 seconds.
Compute gene-gene similarity ... Elapsed time is 2.094708 seconds.
Create gene-gene graph for clustering genes ...
Writing graph into file ... 100%Elapsed time is 1.675288 seconds.
Running ModularityOptimizer for clustering ...Elapsed time is 3.754789 seconds.
Gene-gene graph contains 3 pathways, 3431 genes in total
Elapsed time is 3.930353 seconds.
Create cell-cell graph for clustering cells ...
Writing graph into file ... 100%Elapsed time is 0.053328 seconds.
Running ModularityOptimizer for clustering ...Elapsed time is 0.367663 seconds.
Cell-cell graph contains 8 cell types by community detection
Elapsed time is 0.379411 seconds.
Cell-cell graph contains 8 cell types after merging tiny cell clusters
Cell-cell graph contains 2 cell types after merging
Number of useful pathways is 2
```

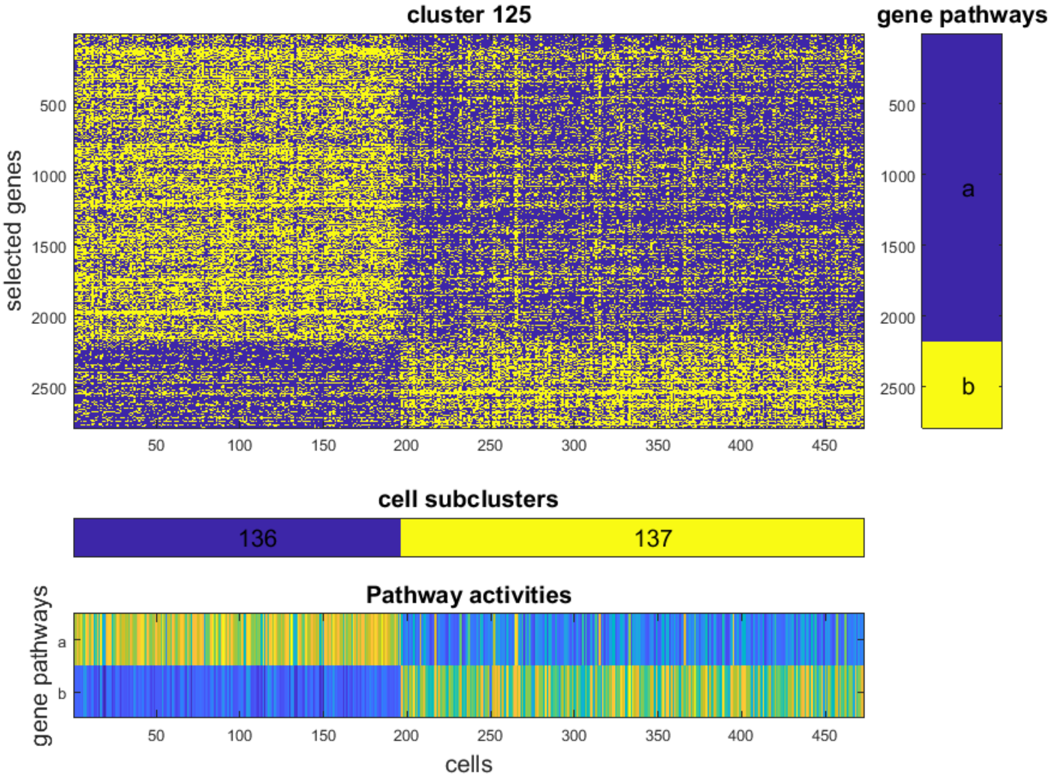


```
Remaining clusters to partition 12
Processing cluster 126 now ...
Processing data subset with 22327 genes and 263 cells:
Remove genes detected in <100 cells. Remaining 2879 genes.  Elapsed time is 0.018718 seconds.
Iterate 10 random permutations for gene-gene similarity threshold ...   10 Elapsed time is 5.853932 seconds.
Compute gene-gene similarity ... Elapsed time is 0.469233 seconds.
Create gene-gene graph for clustering genes ...
Writing graph into file ... 100%Elapsed time is 0.113013 seconds.
Running ModularityOptimizer for clustering ...Elapsed time is 0.529354 seconds.
Gene-gene graph contains 3 pathways, 780 genes in total
Elapsed time is 0.602426 seconds.
Create cell-cell graph for clustering cells ...
Writing graph into file ... 100%Elapsed time is 0.029279 seconds.
Running ModularityOptimizer for clustering ...Elapsed time is 0.304446 seconds.
Cell-cell graph contains 6 cell types by community detection
Elapsed time is 0.312129 seconds.
Cell-cell graph contains 6 cell types after merging tiny cell clusters
creating a total of 5 edges ...       5
Cell-cell graph contains 2 cell types after merging
Number of useful pathways is 1
```

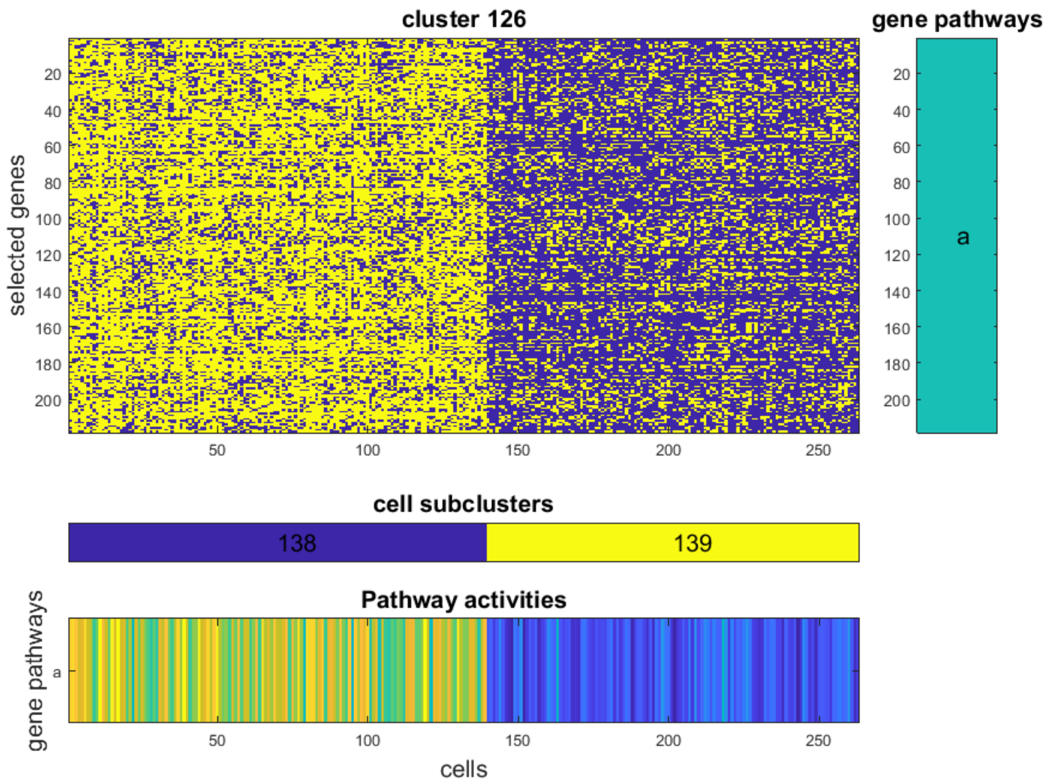


```
Remaining clusters to partition 13
Processing cluster 127 now ...
Processing data subset with 22327 genes and 4521 cells:
Remove genes detected in <100 cells. Remaining 12839 genes.  Elapsed time is 0.463863 seconds.
Iterate 10 random permutations for gene-gene similarity threshold ...   10 Elapsed time is 241.427334 seconds.
Compute gene-gene similarity ... Elapsed time is 17.277466 seconds.
Create gene-gene graph for clustering genes ...
Writing graph into file ... 100%Elapsed time is 22.205724 seconds.
Running ModularityOptimizer for clustering ...Elapsed time is 75.286921 seconds.
Gene-gene graph contains 6 pathways, 11951 genes in total
Elapsed time is 75.807069 seconds.
Create cell-cell graph for clustering cells ...
Writing graph into file ... 100%Elapsed time is 0.533523 seconds.
Running ModularityOptimizer for clustering ...Elapsed time is 2.424602 seconds.
Cell-cell graph contains 14 cell types by community detection
Elapsed time is 2.516907 seconds.
Cell-cell graph contains 11 cell types after merging tiny cell clusters
creating a total of 10 edges ...     10
Cell-cell graph contains 2 cell types after merging
Number of useful pathways is 1
```

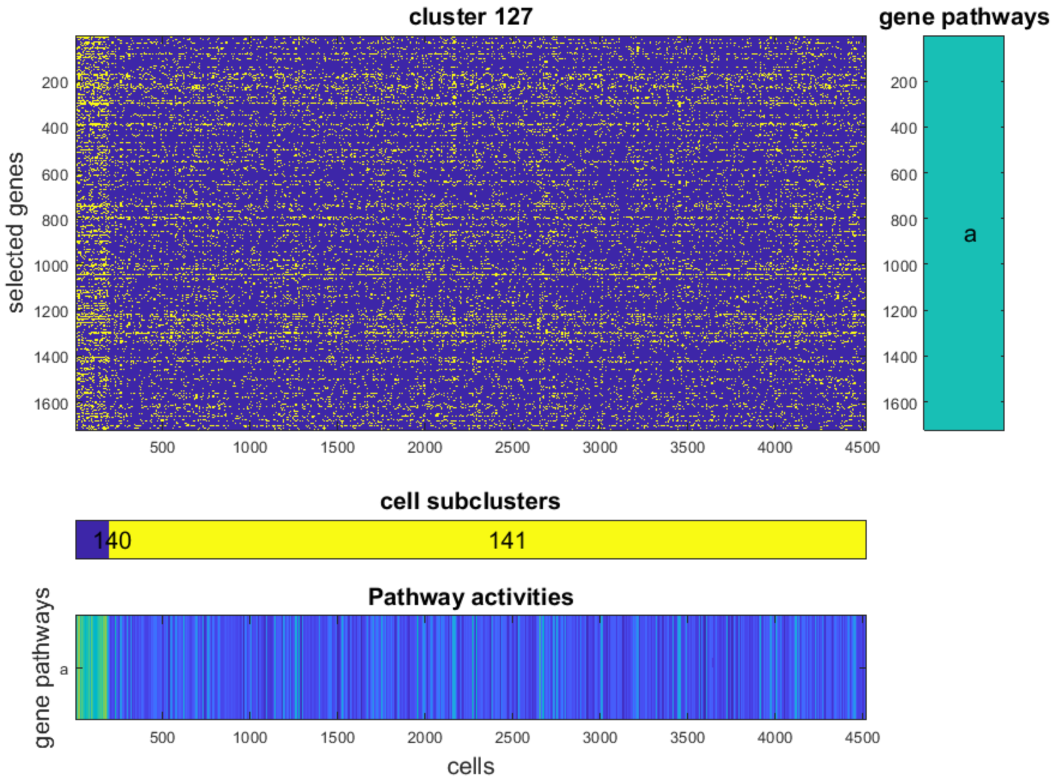


```
Remaining clusters to partition 14
Processing cluster 128 now ...
Processing data subset with 22327 genes and 1654 cells:
Remove genes detected in <100 cells. Remaining 6796 genes.  Elapsed time is 0.132976 seconds.
Iterate 10 random permutations for gene-gene similarity threshold ...   10 Elapsed time is 46.639883 seconds.
Compute gene-gene similarity ... Elapsed time is 3.390692 seconds.
Create gene-gene graph for clustering genes ...
Writing graph into file ... 100%Elapsed time is 0.460356 seconds.
Running ModularityOptimizer for clustering ...Elapsed time is 0.872534 seconds.
Gene-gene graph contains 5 pathways, 1696 genes in total
Elapsed time is 1.079582 seconds.
Create cell-cell graph for clustering cells ...
Writing graph into file ... 100%Elapsed time is 0.194101 seconds.
Running ModularityOptimizer for clustering ...Elapsed time is 1.116739 seconds.
Cell-cell graph contains 11 cell types by community detection
Elapsed time is 1.153304 seconds.
Cell-cell graph contains 11 cell types after merging tiny cell clusters
creating a total of 10 edges ...     10
Cell-cell graph contains 1 cell types after merging

Remaining clusters to partition 13
Processing cluster 129 now ...
Processing data subset with 22327 genes and 3615 cells:
Remove genes detected in <100 cells. Remaining 10040 genes.  Elapsed time is 0.344377 seconds.
Iterate 10 random permutations for gene-gene similarity threshold ...   10 Elapsed time is 134.491219 seconds.
Compute gene-gene similarity ... Elapsed time is 9.908466 seconds.
Create gene-gene graph for clustering genes ...
Writing graph into file ... 100%Elapsed time is 6.269152 seconds.
Running ModularityOptimizer for clustering ...Elapsed time is 23.750865 seconds.
Gene-gene graph contains 5 pathways, 7977 genes in total
Elapsed time is 24.112394 seconds.
Create cell-cell graph for clustering cells ...
Writing graph into file ... 100%Elapsed time is 0.421791 seconds.
Running ModularityOptimizer for clustering ...Elapsed time is 1.877499 seconds.
Cell-cell graph contains 16 cell types by community detection
Elapsed time is 1.953240 seconds.
Cell-cell graph contains 15 cell types after merging tiny cell clusters
Cell-cell graph contains 2 cell types after merging
Number of useful pathways is 1
```

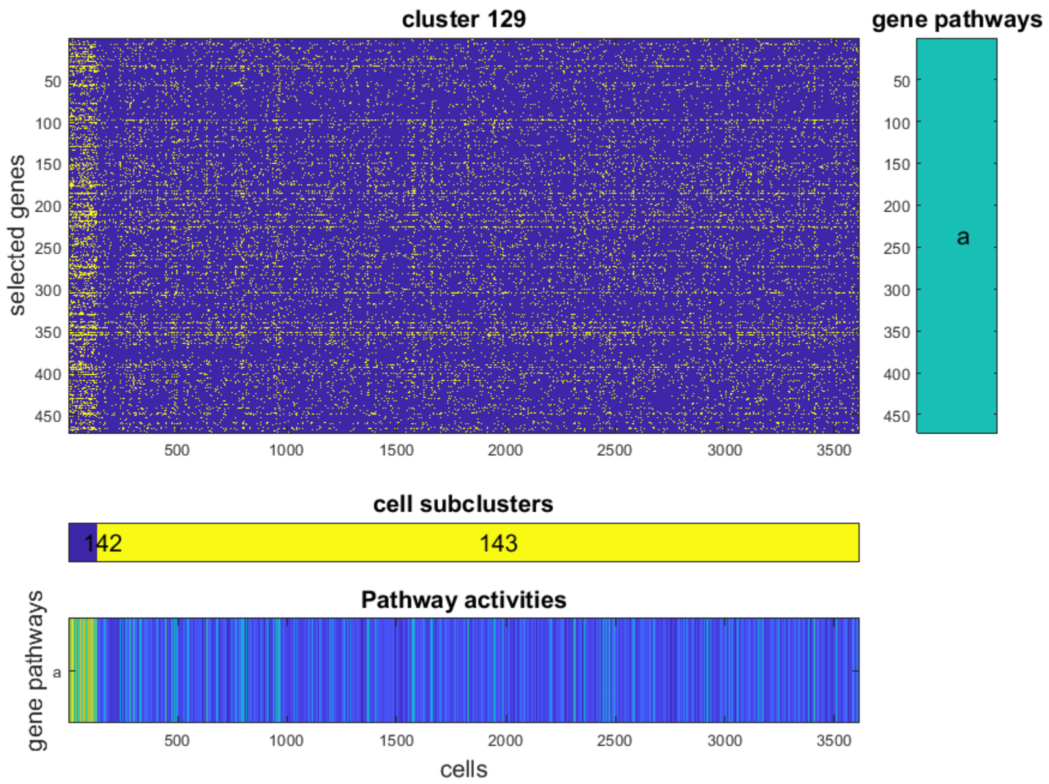


```
Remaining clusters to partition 14
Processing cluster 130 now ...
Processing data subset with 22327 genes and 259 cells:
Remove genes detected in <100 cells. Remaining 613 genes.  Elapsed time is 0.015578 seconds.
Iterate 10 random permutations for gene-gene similarity threshold ...   10 Elapsed time is 0.323188 seconds.
Compute gene-gene similarity ... Elapsed time is 0.022218 seconds.
Create gene-gene graph for clustering genes ...
Writing graph into file ... 101%Elapsed time is 0.004185 seconds.
Running ModularityOptimizer for clustering ...Elapsed time is 0.231897 seconds.
Gene-gene graph contains 0 pathways, 0 genes in total
Elapsed time is 0.248439 seconds.

Remaining clusters to partition 13
Processing cluster 131 now ...
Processing data subset with 22327 genes and 1174 cells:
Remove genes detected in <100 cells. Remaining 5291 genes.  Elapsed time is 0.089221 seconds.
Iterate 10 random permutations for gene-gene similarity threshold ...   10 Elapsed time is 26.445446 seconds.
Compute gene-gene similarity ... Elapsed time is 1.922783 seconds.
Create gene-gene graph for clustering genes ...
Writing graph into file ... 100%Elapsed time is 0.234395 seconds.
Running ModularityOptimizer for clustering ...Elapsed time is 0.682925 seconds.
Gene-gene graph contains 8 pathways, 1408 genes in total
Elapsed time is 0.842827 seconds.
Create cell-cell graph for clustering cells ...
Writing graph into file ... 100%Elapsed time is 0.144522 seconds.
Running ModularityOptimizer for clustering ...Elapsed time is 0.881806 seconds.
Cell-cell graph contains 9 cell types by community detection
Elapsed time is 0.907864 seconds.
Cell-cell graph contains 9 cell types after merging tiny cell clusters
creating a total of 8 edges ...      8
Cell-cell graph contains 1 cell types after merging

Remaining clusters to partition 12
Processing cluster 132 now ...
Processing data subset with 22327 genes and 587 cells:
Remove genes detected in <100 cells. Remaining 2197 genes.  Elapsed time is 0.039046 seconds.
Iterate 10 random permutations for gene-gene similarity threshold ...   10 Elapsed time is 4.208384 seconds.
Compute gene-gene similarity ... Elapsed time is 0.305426 seconds.
Create gene-gene graph for clustering genes ...
Writing graph into file ... 100%Elapsed time is 0.035184 seconds.
Running ModularityOptimizer for clustering ...Elapsed time is 0.342524 seconds.
Gene-gene graph contains 1 pathways, 82 genes in total
Elapsed time is 0.396611 seconds.
Create cell-cell graph for clustering cells ...
Writing graph into file ... 100%Elapsed time is 0.062764 seconds.
Running ModularityOptimizer for clustering ...Elapsed time is 0.455667 seconds.
Cell-cell graph contains 9 cell types by community detection
Elapsed time is 0.472952 seconds.
Cell-cell graph contains 2 cell types after merging tiny cell clusters
creating a total of 1 edges ...      1
Cell-cell graph contains 1 cell types after merging

Remaining clusters to partition 11
Processing cluster 133 now ...
Processing data subset with 22327 genes and 57 cells:
Remove genes detected in <100 cells. Remaining 0 genes.  Elapsed time is 0.004000 seconds.
```

```
Remaining clusters to partition 10
Processing cluster 134 now ...
Processing data subset with 22327 genes and 29 cells:
Remove genes detected in <100 cells. Remaining 0 genes.  Elapsed time is 0.001836 seconds.

Remaining clusters to partition 9
Processing cluster 135 now ...
Processing data subset with 22327 genes and 659 cells:
Remove genes detected in <100 cells. Remaining 4359 genes.  Elapsed time is 0.049132 seconds.
Iterate 10 random permutations for gene-gene similarity threshold ...   10 Elapsed time is 15.757715 seconds.
Compute gene-gene similarity ... Elapsed time is 1.181897 seconds.
Create gene-gene graph for clustering genes ...
Writing graph into file ... 100%Elapsed time is 0.462301 seconds.
Running ModularityOptimizer for clustering ...Elapsed time is 1.228418 seconds.
Gene-gene graph contains 3 pathways, 2123 genes in total
Elapsed time is 1.349596 seconds.
Create cell-cell graph for clustering cells ...
Writing graph into file ... 100%Elapsed time is 0.074703 seconds.
Running ModularityOptimizer for clustering ...Elapsed time is 0.456171 seconds.
Cell-cell graph contains 8 cell types by community detection
Elapsed time is 0.472044 seconds.
Cell-cell graph contains 8 cell types after merging tiny cell clusters
creating a total of 7 edges ...      7
Cell-cell graph contains 2 cell types after merging
Number of useful pathways is 1
```

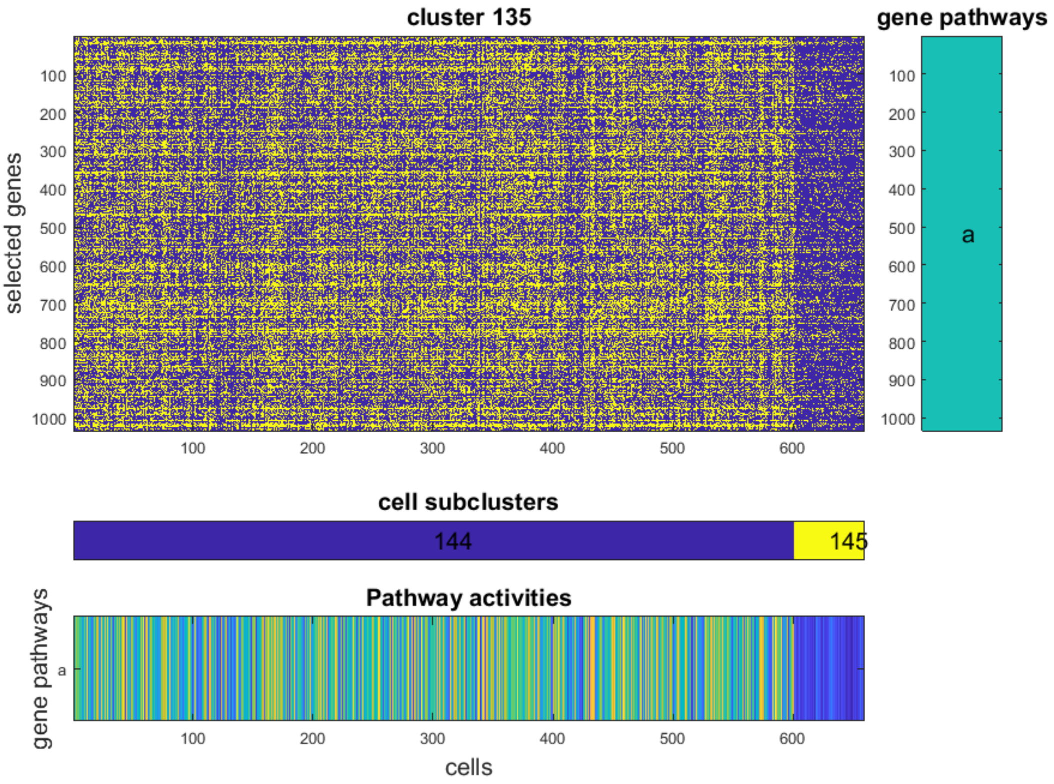


```
Remaining clusters to partition 10
Processing cluster 136 now ...
Processing data subset with 22327 genes and 196 cells:
Remove genes detected in <100 cells. Remaining 0 genes.  Elapsed time is 0.010930 seconds.

Remaining clusters to partition 9
Processing cluster 137 now ...
Processing data subset with 22327 genes and 277 cells:
Remove genes detected in <100 cells. Remaining 1979 genes.  Elapsed time is 0.020265 seconds.
Iterate 10 random permutations for gene-gene similarity threshold ...   10 Elapsed time is 2.914855 seconds.
Compute gene-gene similarity ... Elapsed time is 0.215648 seconds.
Create gene-gene graph for clustering genes ...
Writing graph into file ... 100%Elapsed time is 0.030781 seconds.
Running ModularityOptimizer for clustering ...Elapsed time is 0.361667 seconds.
Gene-gene graph contains 11 pathways, 335 genes in total
Elapsed time is 0.410407 seconds.
Create cell-cell graph for clustering cells ...
Writing graph into file ... 100%Elapsed time is 0.034489 seconds.
Running ModularityOptimizer for clustering ...Elapsed time is 0.334077 seconds.
Cell-cell graph contains 5 cell types by community detection
Elapsed time is 0.341999 seconds.
Cell-cell graph contains 4 cell types after merging tiny cell clusters
creating a total of 3 edges ...      3
Cell-cell graph contains 1 cell types after merging

Remaining clusters to partition 8
Processing cluster 138 now ...
Processing data subset with 22327 genes and 139 cells:
```

```
Remove genes detected in <100 cells. Remaining 0 genes.  Elapsed time is 0.008076 seconds.

Remaining clusters to partition 7
Processing cluster 139 now ...
Processing data subset with 22327 genes and 124 cells:
Remove genes detected in <100 cells. Remaining 0 genes.  Elapsed time is 0.007828 seconds.

Remaining clusters to partition 6
Processing cluster 140 now ...
Processing data subset with 22327 genes and 192 cells:
Remove genes detected in <100 cells. Remaining 0 genes.  Elapsed time is 0.010712 seconds.

Remaining clusters to partition 5
Processing cluster 141 now ...
Processing data subset with 22327 genes and 4329 cells:
Remove genes detected in <100 cells. Remaining 12623 genes.  Elapsed time is 0.431539 seconds.
Iterate 10 random permutations for gene-gene similarity threshold ...   10 Elapsed time is 230.223412 seconds.
Compute gene-gene similarity ... Elapsed time is 16.232589 seconds.
Create gene-gene graph for clustering genes ...
Writing graph into file ... 100%Elapsed time is 22.091612 seconds.
Running ModularityOptimizer for clustering ...Elapsed time is 64.562145 seconds.
Gene-gene graph contains 6 pathways, 11550 genes in total
Elapsed time is 65.061159 seconds.
Create cell-cell graph for clustering cells ...
Writing graph into file ... 100%Elapsed time is 0.503937 seconds.
Running ModularityOptimizer for clustering ...Elapsed time is 2.317938 seconds.
Cell-cell graph contains 16 cell types by community detection
Elapsed time is 2.408158 seconds.
Cell-cell graph contains 15 cell types after merging tiny cell clusters
creating a total of 14 edges ...     14
Cell-cell graph contains 2 cell types after merging
Number of useful pathways is 1
```

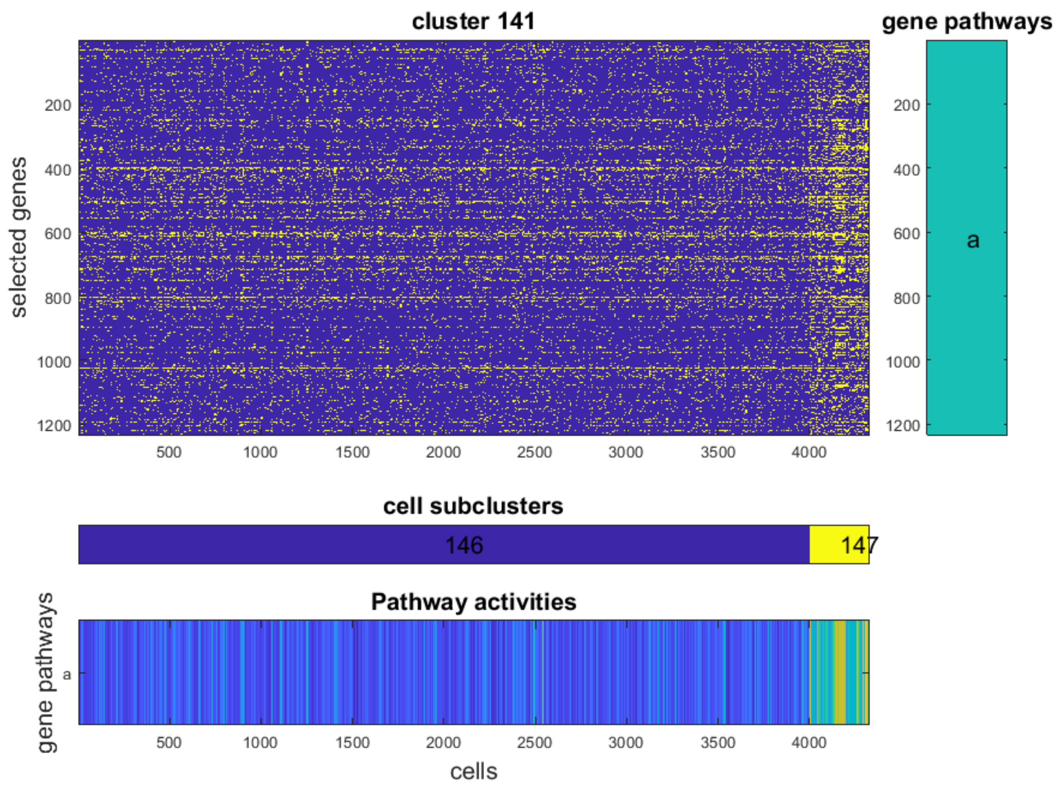


```
Remaining clusters to partition 6
Processing cluster 142 now ...
Processing data subset with 22327 genes and 131 cells:
Remove genes detected in <100 cells. Remaining 0 genes.  Elapsed time is 0.007357 seconds.

Remaining clusters to partition 5
Processing cluster 143 now ...
Processing data subset with 22327 genes and 3484 cells:
Remove genes detected in <100 cells. Remaining 9813 genes.  Elapsed time is 0.310600 seconds.
Iterate 10 random permutations for gene-gene similarity threshold ...   10 Elapsed time is 127.792298 seconds.
Compute gene-gene similarity ... Elapsed time is 9.118678 seconds.
Create gene-gene graph for clustering genes ...
Writing graph into file ... 100%Elapsed time is 5.908579 seconds.
Running ModularityOptimizer for clustering ...Elapsed time is 24.089662 seconds.
Gene-gene graph contains 5 pathways, 7627 genes in total
Elapsed time is 24.435365 seconds.
Create cell-cell graph for clustering cells ...
Writing graph into file ... 100%Elapsed time is 0.409666 seconds.
Running ModularityOptimizer for clustering ...Elapsed time is 2.099669 seconds.
Cell-cell graph contains 14 cell types by community detection
Elapsed time is 2.172431 seconds.
Cell-cell graph contains 13 cell types after merging tiny cell clusters
```

```
Cell-cell graph contains 2 cell types after merging
Number of useful pathways is 1
```

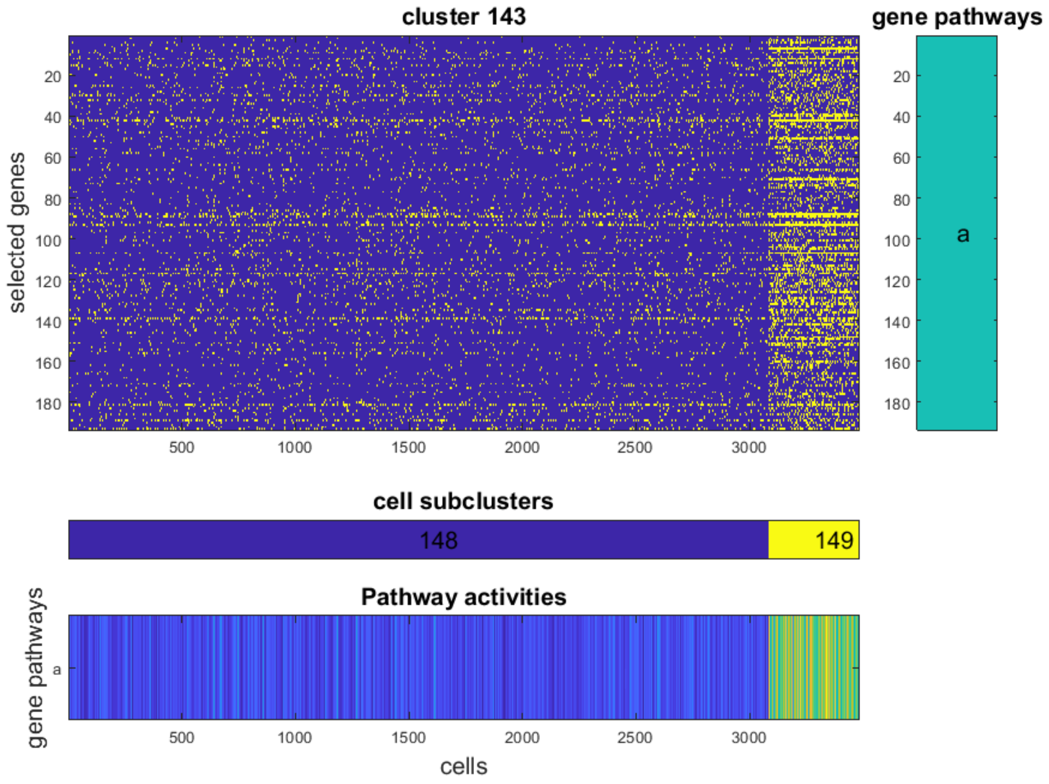


```
Remaining clusters to partition 6
Processing cluster 144 now ...
Processing data subset with 22327 genes and 600 cells:
Remove genes detected in <100 cells. Remaining 3925 genes.  Elapsed time is 0.046996 seconds.
Iterate 10 random permutations for gene-gene similarity threshold ...   10 Elapsed time is 12.517401 seconds.
Compute gene-gene similarity ... Elapsed time is 0.939217 seconds.
Create gene-gene graph for clustering genes ...
Writing graph into file ... 100%Elapsed time is 0.295916 seconds.
Running ModularityOptimizer for clustering ...Elapsed time is 0.943001 seconds.
Gene-gene graph contains 3 pathways, 1791 genes in total
Elapsed time is 1.057723 seconds.
Create cell-cell graph for clustering cells ...
Writing graph into file ... 100%Elapsed time is 0.066858 seconds.
Running ModularityOptimizer for clustering ...Elapsed time is 0.415203 seconds.
Cell-cell graph contains 8 cell types by community detection
Elapsed time is 0.429837 seconds.
Cell-cell graph contains 8 cell types after merging tiny cell clusters
creating a total of 7 edges ...      7
Cell-cell graph contains 2 cell types after merging
Number of useful pathways is 1
```

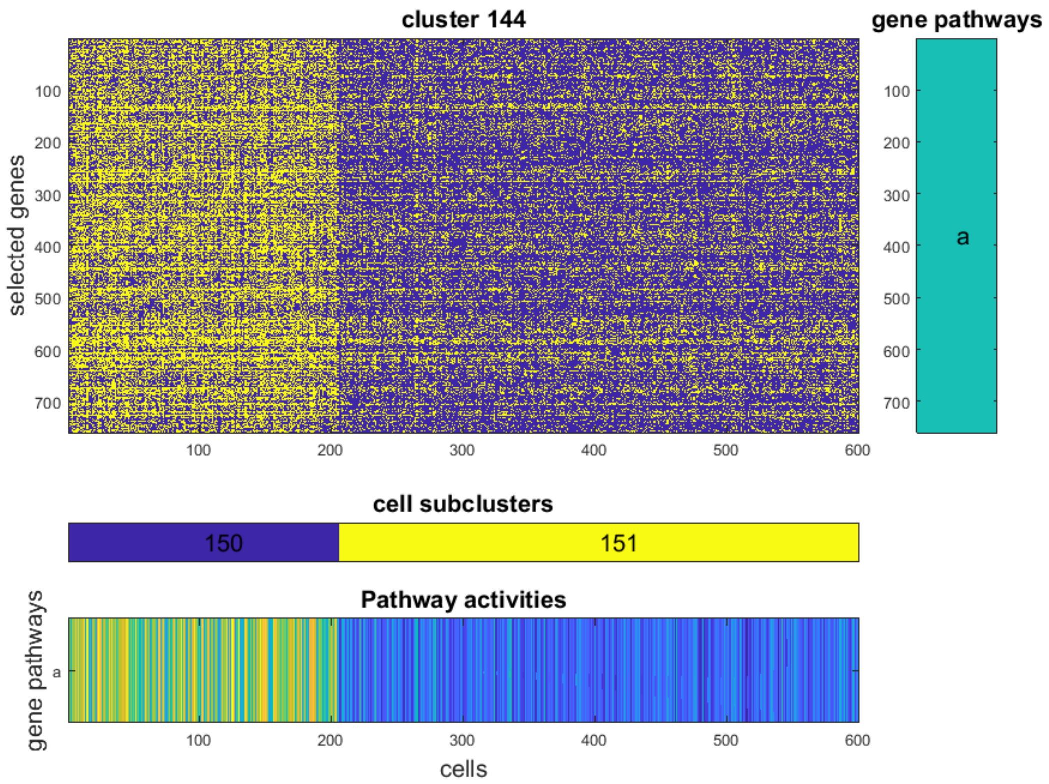


```
Remaining clusters to partition 7
Processing cluster 145 now ...
Processing data subset with 22327 genes and 59 cells:
Remove genes detected in <100 cells. Remaining 0 genes.  Elapsed time is 0.004025 seconds.

Remaining clusters to partition 6
Processing cluster 146 now ...
Processing data subset with 22327 genes and 3998 cells:
Remove genes detected in <100 cells. Remaining 12321 genes.  Elapsed time is 0.412187 seconds.
Iterate 10 random permutations for gene-gene similarity threshold ...   10 Elapsed time is 211.115370 seconds.
Compute gene-gene similarity ... Elapsed time is 15.274985 seconds.
Create gene-gene graph for clustering genes ...
Writing graph into file ... 100%Elapsed time is 20.992495 seconds.
Running ModularityOptimizer for clustering ...Elapsed time is 68.385561 seconds.
Gene-gene graph contains 4 pathways, 11095 genes in total
Elapsed time is 68.869276 seconds.
Create cell-cell graph for clustering cells ...
Writing graph into file ... 100%Elapsed time is 0.455384 seconds.
Running ModularityOptimizer for clustering ...Elapsed time is 1.989998 seconds.
Cell-cell graph contains 15 cell types by community detection
Elapsed time is 2.072306 seconds.
Cell-cell graph contains 13 cell types after merging tiny cell clusters
creating a total of 12 edges ...     12
Cell-cell graph contains 2 cell types after merging
Number of useful pathways is 1
```

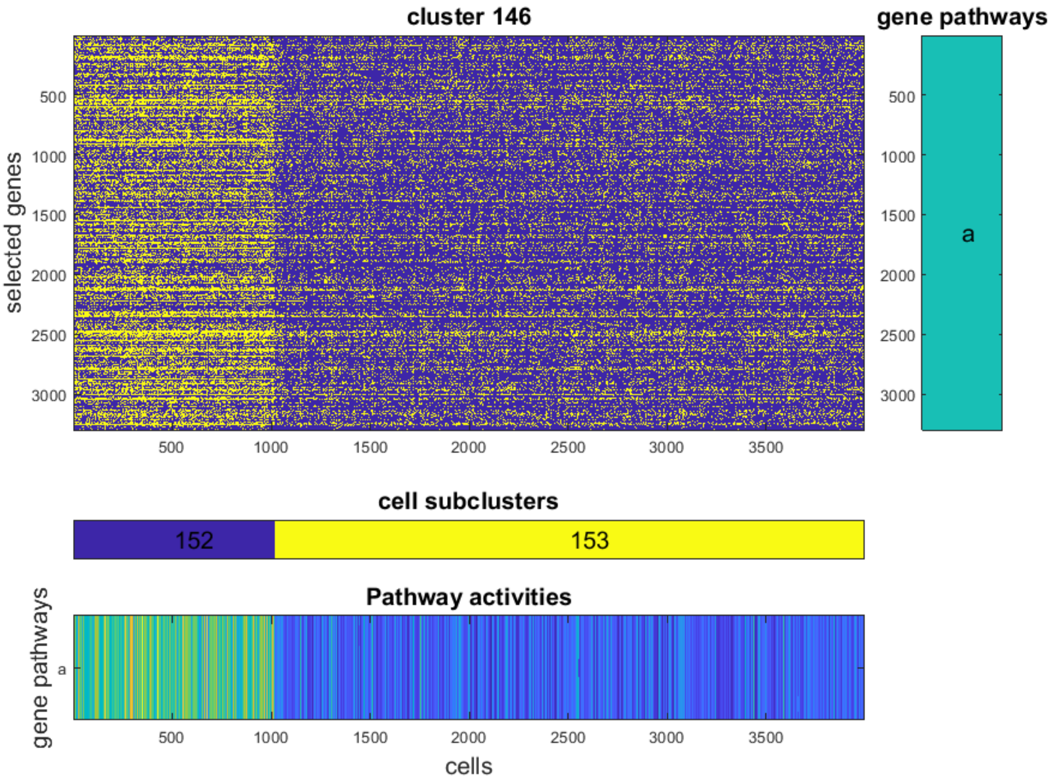


```
Remaining clusters to partition 7
Processing cluster 147 now ...
Processing data subset with 22327 genes and 331 cells:
Remove genes detected in <100 cells. Remaining 2996 genes.  Elapsed time is 0.023496 seconds.
Iterate 10 random permutations for gene-gene similarity threshold ...   10 Elapsed time is 6.527729 seconds.
Compute gene-gene similarity ... Elapsed time is 0.538852 seconds.
Create gene-gene graph for clustering genes ...
Writing graph into file ... 100%Elapsed time is 0.360364 seconds.
Running ModularityOptimizer for clustering ...Elapsed time is 0.818364 seconds.
Gene-gene graph contains 3 pathways, 1420 genes in total
Elapsed time is 0.896749 seconds.
Create cell-cell graph for clustering cells ...
Writing graph into file ... 100%Elapsed time is 0.038186 seconds.
Running ModularityOptimizer for clustering ...Elapsed time is 0.329396 seconds.
Cell-cell graph contains 8 cell types by community detection
Elapsed time is 0.338827 seconds.
Cell-cell graph contains 7 cell types after merging tiny cell clusters
creating a total of 6 edges ...       6
Cell-cell graph contains 2 cell types after merging
Number of useful pathways is 1
```

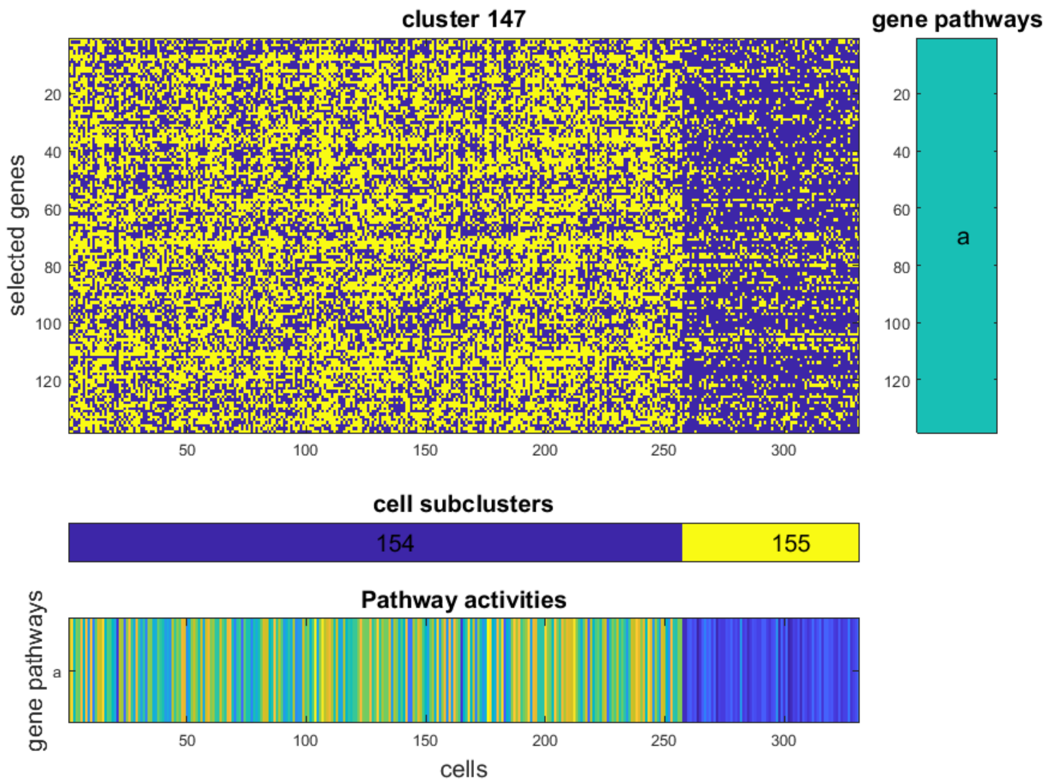


```
Remaining clusters to partition 8
Processing cluster 148 now ...
Processing data subset with 22327 genes and 3086 cells:
Remove genes detected in <100 cells. Remaining 9510 genes.  Elapsed time is 0.287671 seconds.
Iterate 10 random permutations for gene-gene similarity threshold ...   10 Elapsed time is 112.498986 seconds.
Compute gene-gene similarity ... Elapsed time is 8.113145 seconds.
Create gene-gene graph for clustering genes ...
Writing graph into file ... 100%Elapsed time is 5.968929 seconds.
Running ModularityOptimizer for clustering ...Elapsed time is 20.800394 seconds.
Gene-gene graph contains 5 pathways, 7278 genes in total
Elapsed time is 21.131313 seconds.
Create cell-cell graph for clustering cells ...
Writing graph into file ... 100%Elapsed time is 0.341761 seconds.
Running ModularityOptimizer for clustering ...Elapsed time is 1.561718 seconds.
Cell-cell graph contains 17 cell types by community detection
Elapsed time is 1.627560 seconds.
Cell-cell graph contains 16 cell types after merging tiny cell clusters
creating a total of 15 edges ...      15
Cell-cell graph contains 1 cell types after merging

Remaining clusters to partition 7
Processing cluster 149 now ...
Processing data subset with 22327 genes and 398 cells:
Remove genes detected in <100 cells. Remaining 866 genes.  Elapsed time is 0.028953 seconds.
Iterate 10 random permutations for gene-gene similarity threshold ...   10 Elapsed time is 0.715271 seconds.
Compute gene-gene similarity ... Elapsed time is 0.043988 seconds.
Create gene-gene graph for clustering genes ...
Writing graph into file ... 100%Elapsed time is 0.009380 seconds.
Running ModularityOptimizer for clustering ...Elapsed time is 0.266170 seconds.
Gene-gene graph contains 1 pathways, 32 genes in total
Elapsed time is 0.294644 seconds.
Create cell-cell graph for clustering cells ...
Writing graph into file ... 100%Elapsed time is 0.054122 seconds.
Running ModularityOptimizer for clustering ...Elapsed time is 0.334631 seconds.
Cell-cell graph contains 7 cell types by community detection
Elapsed time is 0.345488 seconds.
Cell-cell graph contains 2 cell types after merging tiny cell clusters
creating a total of 1 edges ...      1
Cell-cell graph contains 1 cell types after merging

Remaining clusters to partition 6
Processing cluster 150 now ...
Processing data subset with 22327 genes and 205 cells:
Remove genes detected in <100 cells. Remaining 111 genes.  Elapsed time is 0.013997 seconds.
Iterate 10 random permutations for gene-gene similarity threshold ...   10 Elapsed time is 0.024884 seconds.
Compute gene-gene similarity ... Elapsed time is 0.001236 seconds.
Create gene-gene graph for clustering genes ...
Writing graph into file ... 102%Elapsed time is 0.001811 seconds.
Running ModularityOptimizer for clustering ...Elapsed time is 0.181154 seconds.
Gene-gene graph contains 0 pathways, 0 genes in total
Elapsed time is 0.185932 seconds.

Remaining clusters to partition 5
Processing cluster 151 now ...
Processing data subset with 22327 genes and 395 cells:
Remove genes detected in <100 cells. Remaining 2004 genes.  Elapsed time is 0.026441 seconds.
```

```
Iterate 10 random permutations for gene-gene similarity threshold ...   10 Elapsed time is 3.204617 seconds.
Compute gene-gene similarity ... Elapsed time is 0.226627 seconds.
Create gene-gene graph for clustering genes ...
Writing graph into file ... 100%Elapsed time is 0.033980 seconds.
Running ModularityOptimizer for clustering ...Elapsed time is 0.421447 seconds.
Gene-gene graph contains 10 pathways, 504 genes in total
Elapsed time is 0.471207 seconds.
Create cell-cell graph for clustering cells ...
Writing graph into file ... 100%Elapsed time is 0.046803 seconds.
Running ModularityOptimizer for clustering ...Elapsed time is 0.389327 seconds.
Cell-cell graph contains 6 cell types by community detection
Elapsed time is 0.400413 seconds.
Cell-cell graph contains 6 cell types after merging tiny cell clusters
creating a total of 5 edges ...      5
Cell-cell graph contains 1 cell types after merging

Remaining clusters to partition 4
Processing cluster 152 now ...
Processing data subset with 22327 genes and 1016 cells:
Remove genes detected in <100 cells. Remaining 8250 genes.  Elapsed time is 0.091186 seconds.
Iterate 10 random permutations for gene-gene similarity threshold ...   10 Elapsed time is 57.920332 seconds.
Compute gene-gene similarity ... Elapsed time is 4.415058 seconds.
Create gene-gene graph for clustering genes ...
Writing graph into file ... 100%Elapsed time is 0.499553 seconds.
Running ModularityOptimizer for clustering ...Elapsed time is 1.333217 seconds.
Gene-gene graph contains 8 pathways, 3117 genes in total
Elapsed time is 1.600064 seconds.
Create cell-cell graph for clustering cells ...
Writing graph into file ... 100%Elapsed time is 0.122642 seconds.
Running ModularityOptimizer for clustering ...Elapsed time is 0.765646 seconds.
Cell-cell graph contains 8 cell types by community detection
Elapsed time is 0.788148 seconds.
Cell-cell graph contains 8 cell types after merging tiny cell clusters
Cell-cell graph contains 2 cell types after merging
Number of useful pathways is 2
```

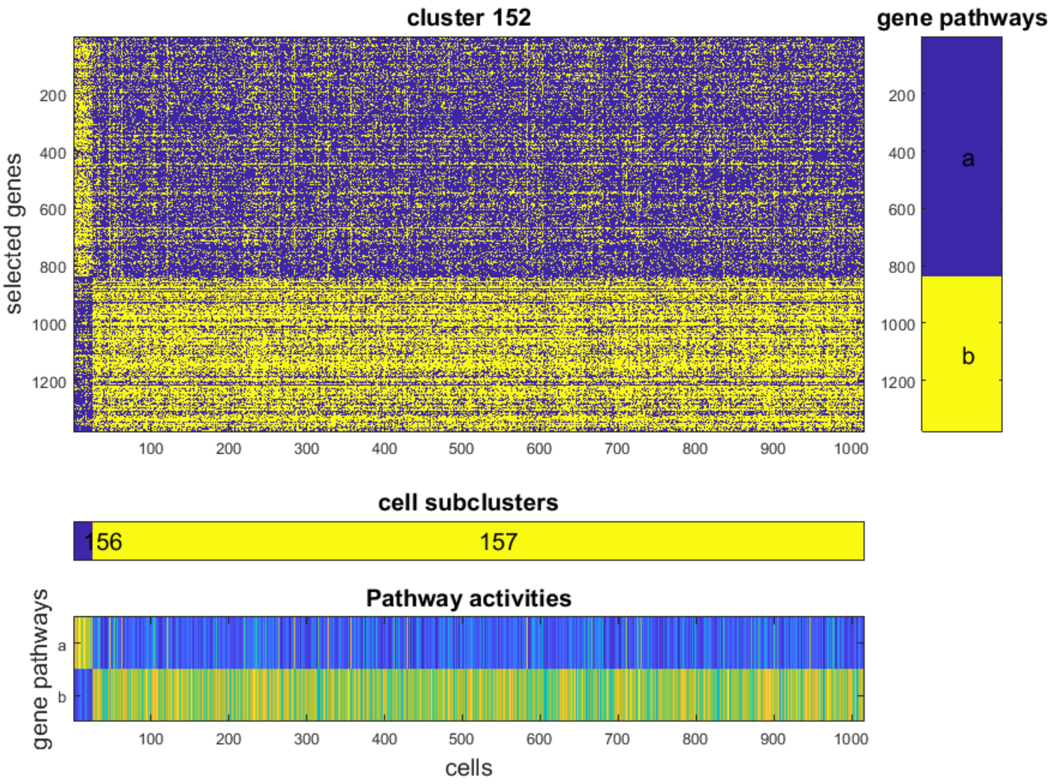


```
Remaining clusters to partition 5
Processing cluster 153 now ...
Processing data subset with 22327 genes and 2982 cells:
Remove genes detected in <100 cells. Remaining 11485 genes.  Elapsed time is 0.277855 seconds.
Iterate 10 random permutations for gene-gene similarity threshold ...   10 Elapsed time is 158.298415 seconds.
Compute gene-gene similarity ... Elapsed time is 11.649667 seconds.
Create gene-gene graph for clustering genes ...
Writing graph into file ... 100%Elapsed time is 7.029031 seconds.
Running ModularityOptimizer for clustering ...Elapsed time is 24.956755 seconds.
Gene-gene graph contains 7 pathways, 9319 genes in total
Elapsed time is 25.398322 seconds.
Create cell-cell graph for clustering cells ...
Writing graph into file ... 100%Elapsed time is 0.344486 seconds.
Running ModularityOptimizer for clustering ...Elapsed time is 1.450850 seconds.
Cell-cell graph contains 20 cell types by community detection
Elapsed time is 1.513603 seconds.
Cell-cell graph contains 19 cell types after merging tiny cell clusters
Cell-cell graph contains 2 cell types after merging
Number of useful pathways is 2
```

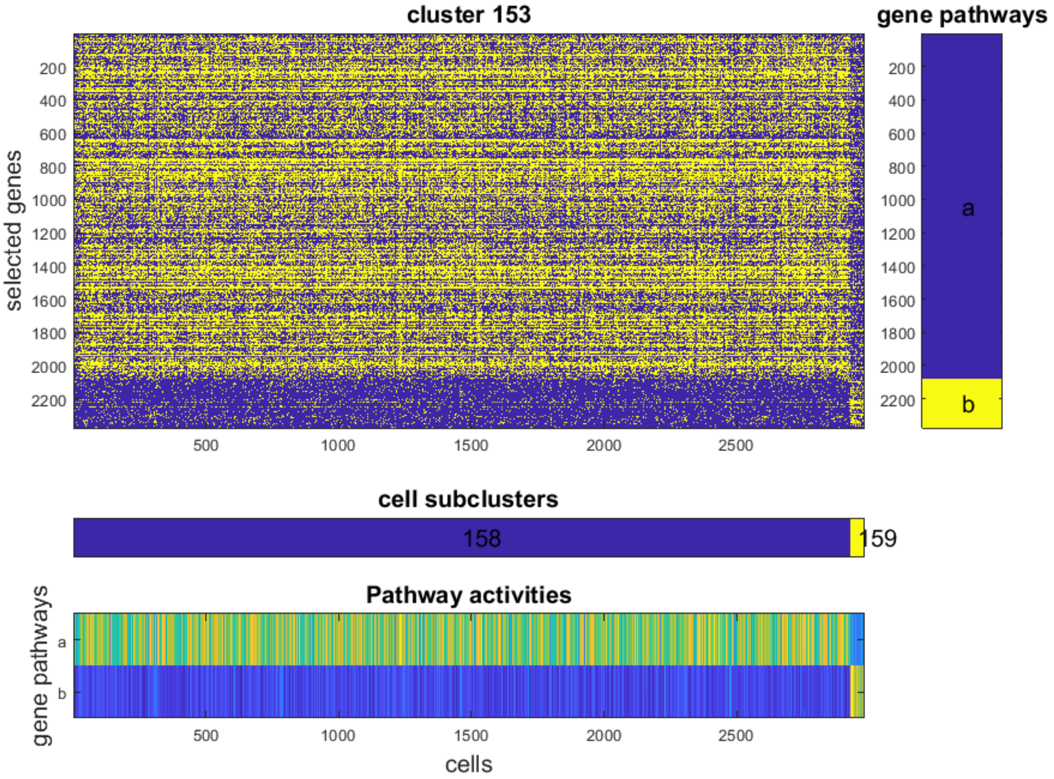


```
Remaining clusters to partition 6
Processing cluster 154 now ...
Processing data subset with 22327 genes and 257 cells:
Remove genes detected in <100 cells. Remaining 1538 genes.  Elapsed time is 0.020088 seconds.
Iterate 10 random permutations for gene-gene similarity threshold ...   10 Elapsed time is 1.801661 seconds.
Compute gene-gene similarity ... Elapsed time is 0.132044 seconds.
Create gene-gene graph for clustering genes ...
Writing graph into file ... 100%Elapsed time is 0.086389 seconds.
Running ModularityOptimizer for clustering ...Elapsed time is 0.506832 seconds.
Gene-gene graph contains 3 pathways, 571 genes in total
Elapsed time is 0.545180 seconds.
Create cell-cell graph for clustering cells ...
Writing graph into file ... 100%Elapsed time is 0.029756 seconds.
Running ModularityOptimizer for clustering ...Elapsed time is 0.303736 seconds.
Cell-cell graph contains 5 cell types by community detection
Elapsed time is 0.311524 seconds.
Cell-cell graph contains 5 cell types after merging tiny cell clusters
Cell-cell graph contains 2 cell types after merging
Number of useful pathways is 2
```

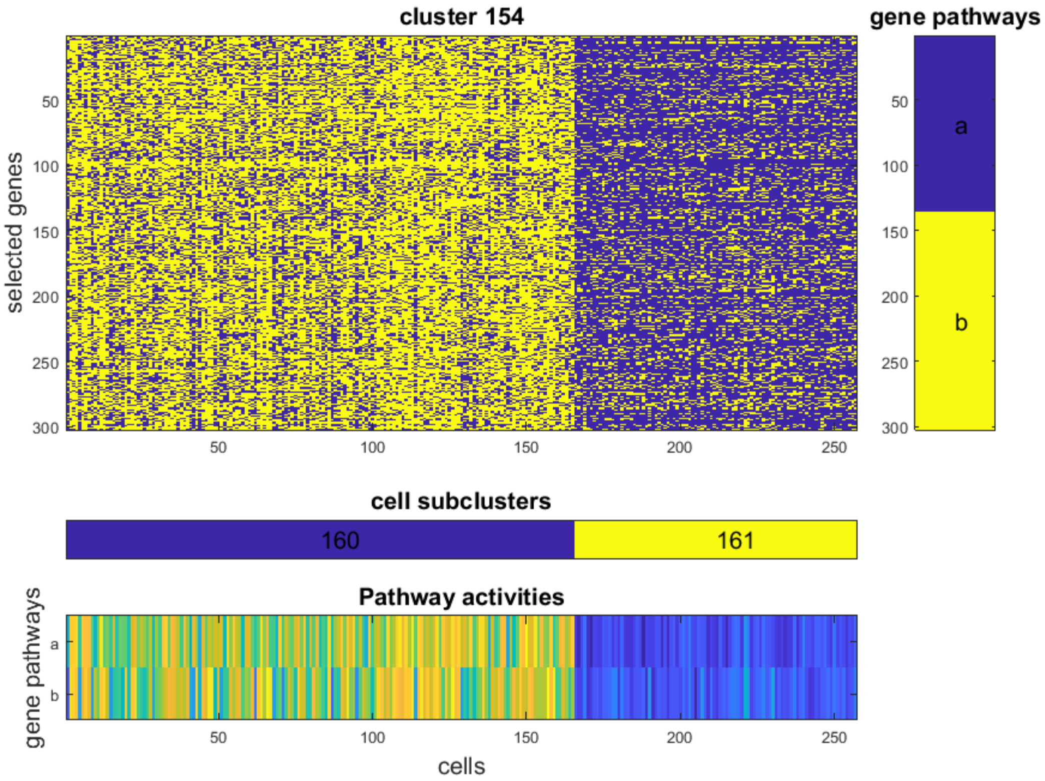


```
Remaining clusters to partition 7
Processing cluster 155 now ...
Processing data subset with 22327 genes and 74 cells:
Remove genes detected in <100 cells. Remaining 0 genes.  Elapsed time is 0.005063 seconds.

Remaining clusters to partition 6
Processing cluster 156 now ...
Processing data subset with 22327 genes and 24 cells:
Remove genes detected in <100 cells. Remaining 0 genes.  Elapsed time is 0.001748 seconds.

Remaining clusters to partition 5
Processing cluster 157 now ...
Processing data subset with 22327 genes and 992 cells:
Remove genes detected in <100 cells. Remaining 8085 genes.  Elapsed time is 0.092055 seconds.
Iterate 10 random permutations for gene-gene similarity threshold ...   10 Elapsed time is 55.719009 seconds.
Compute gene-gene similarity ... Elapsed time is 4.330982 seconds.
Create gene-gene graph for clustering genes ...
Writing graph into file ... 100%Elapsed time is 0.437400 seconds.
Running ModularityOptimizer for clustering ...Elapsed time is 1.124781 seconds.
Gene-gene graph contains 13 pathways, 2589 genes in total
Elapsed time is 1.388482 seconds.
Create cell-cell graph for clustering cells ...
Writing graph into file ... 100%Elapsed time is 0.121472 seconds.
Running ModularityOptimizer for clustering ...Elapsed time is 0.779598 seconds.
Cell-cell graph contains 7 cell types by community detection
Elapsed time is 0.802149 seconds.
Cell-cell graph contains 7 cell types after merging tiny cell clusters
Cell-cell graph contains 2 cell types after merging
Number of useful pathways is 1
```

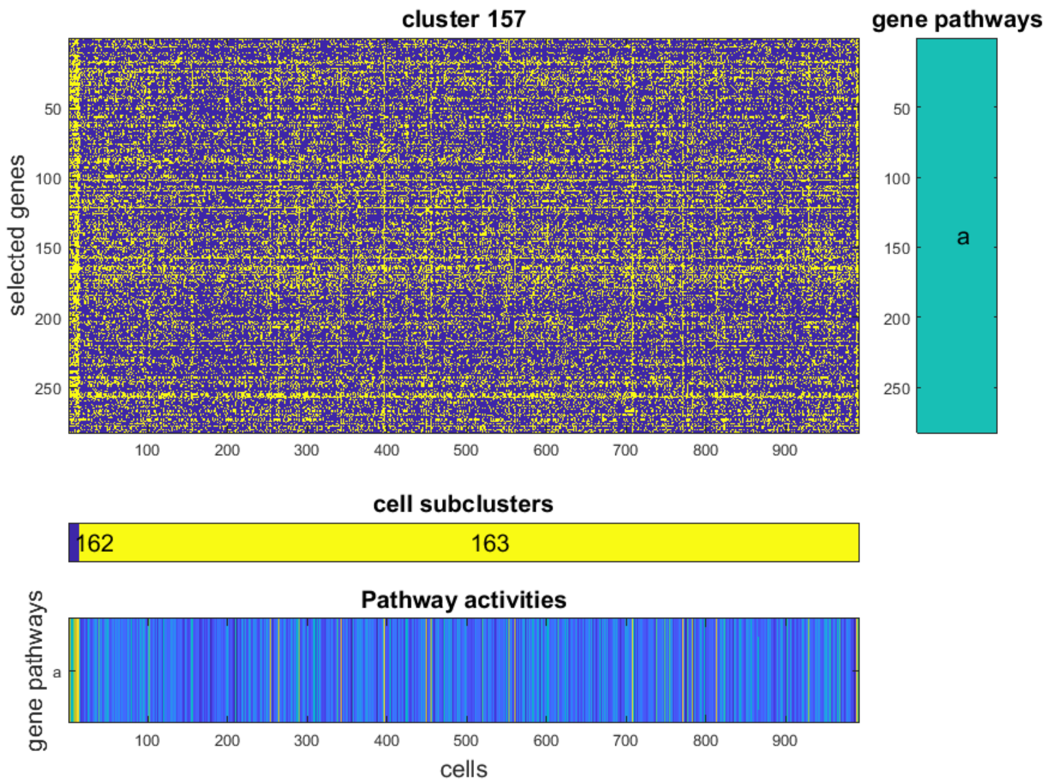


```
Remaining clusters to partition 6
Processing cluster 158 now ...
Processing data subset with 22327 genes and 2930 cells:
Remove genes detected in <100 cells. Remaining 11398 genes.  Elapsed time is 0.281843 seconds.
Iterate 10 random permutations for gene-gene similarity threshold ...   10 Elapsed time is 158.269114 seconds.
Compute gene-gene similarity ... Elapsed time is 12.272357 seconds.
Create gene-gene graph for clustering genes ...
Writing graph into file ... 100%Elapsed time is 6.037210 seconds.
Running ModularityOptimizer for clustering ...Elapsed time is 23.109389 seconds.
Gene-gene graph contains 6 pathways, 9062 genes in total
Elapsed time is 23.548855 seconds.
Create cell-cell graph for clustering cells ...
Writing graph into file ... 100%Elapsed time is 0.339587 seconds.
Running ModularityOptimizer for clustering ...Elapsed time is 1.567518 seconds.
Cell-cell graph contains 15 cell types by community detection
Elapsed time is 1.628881 seconds.
Cell-cell graph contains 13 cell types after merging tiny cell clusters
creating a total of 12 edges ...     12
Cell-cell graph contains 2 cell types after merging
Number of useful pathways is 1
```

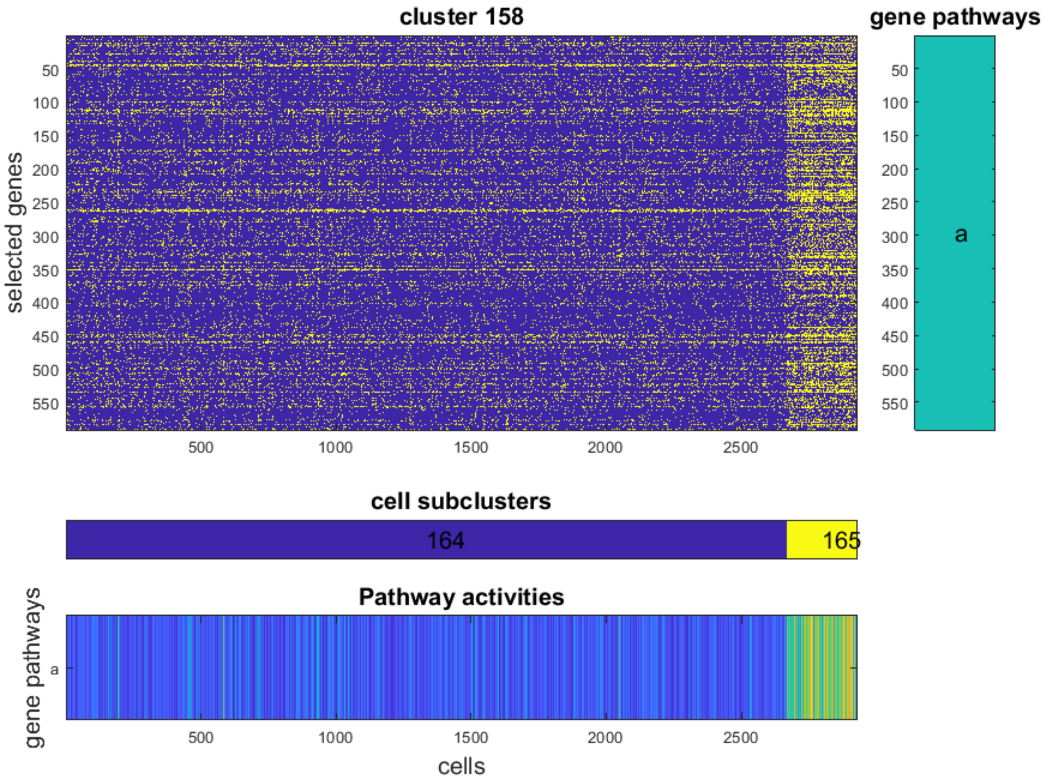


```
Remaining clusters to partition 7
Processing cluster 159 now ...
Processing data subset with 22327 genes and 52 cells:
Remove genes detected in <100 cells. Remaining 0 genes.  Elapsed time is 0.003376 seconds.

Remaining clusters to partition 6
Processing cluster 160 now ...
Processing data subset with 22327 genes and 165 cells:
Remove genes detected in <100 cells. Remaining 0 genes.  Elapsed time is 0.009256 seconds.

Remaining clusters to partition 5
Processing cluster 161 now ...
Processing data subset with 22327 genes and 92 cells:
Remove genes detected in <100 cells. Remaining 0 genes.  Elapsed time is 0.005634 seconds.

Remaining clusters to partition 4
Processing cluster 162 now ...
Processing data subset with 22327 genes and 14 cells:
Remove genes detected in <100 cells. Remaining 0 genes.  Elapsed time is 0.000848 seconds.

Remaining clusters to partition 3
Processing cluster 163 now ...
Processing data subset with 22327 genes and 978 cells:
Remove genes detected in <100 cells. Remaining 7991 genes.  Elapsed time is 0.082712 seconds.
Iterate 10 random permutations for gene-gene similarity threshold ...   10 Elapsed time is 54.972396 seconds.
Compute gene-gene similarity ... Elapsed time is 4.071777 seconds.
Create gene-gene graph for clustering genes ...
Writing graph into file ... 100%Elapsed time is 0.432026 seconds.
Running ModularityOptimizer for clustering ...Elapsed time is 1.115689 seconds.
Gene-gene graph contains 12 pathways, 2434 genes in total
Elapsed time is 1.382104 seconds.
Create cell-cell graph for clustering cells ...
Writing graph into file ... 100%Elapsed time is 0.117609 seconds.
Running ModularityOptimizer for clustering ...Elapsed time is 0.705224 seconds.
Cell-cell graph contains 8 cell types by community detection
Elapsed time is 0.731519 seconds.
Cell-cell graph contains 8 cell types after merging tiny cell clusters
creating a total of 7 edges ...      7
Cell-cell graph contains 1 cell types after merging

Remaining clusters to partition 2
Processing cluster 164 now ...
Processing data subset with 22327 genes and 2667 cells:
Remove genes detected in <100 cells. Remaining 11120 genes.  Elapsed time is 0.257411 seconds.
Iterate 10 random permutations for gene-gene similarity threshold ...   10 Elapsed time is 142.242736 seconds.
Compute gene-gene similarity ... Elapsed time is 10.298177 seconds.
Create gene-gene graph for clustering genes ...
Writing graph into file ... 100%Elapsed time is 4.648265 seconds.
Running ModularityOptimizer for clustering ...Elapsed time is 16.543210 seconds.
Gene-gene graph contains 5 pathways, 8295 genes in total
Elapsed time is 16.962331 seconds.
Create cell-cell graph for clustering cells ...
Writing graph into file ... 100%Elapsed time is 0.306020 seconds.
Running ModularityOptimizer for clustering ...Elapsed time is 1.341854 seconds.
Cell-cell graph contains 14 cell types by community detection
Elapsed time is 1.403455 seconds.
```

```
Cell-cell graph contains 12 cell types after merging tiny cell clusters
creating a total of 11 edges ...     11
Cell-cell graph contains 2 cell types after merging
Number of useful pathways is 1
```

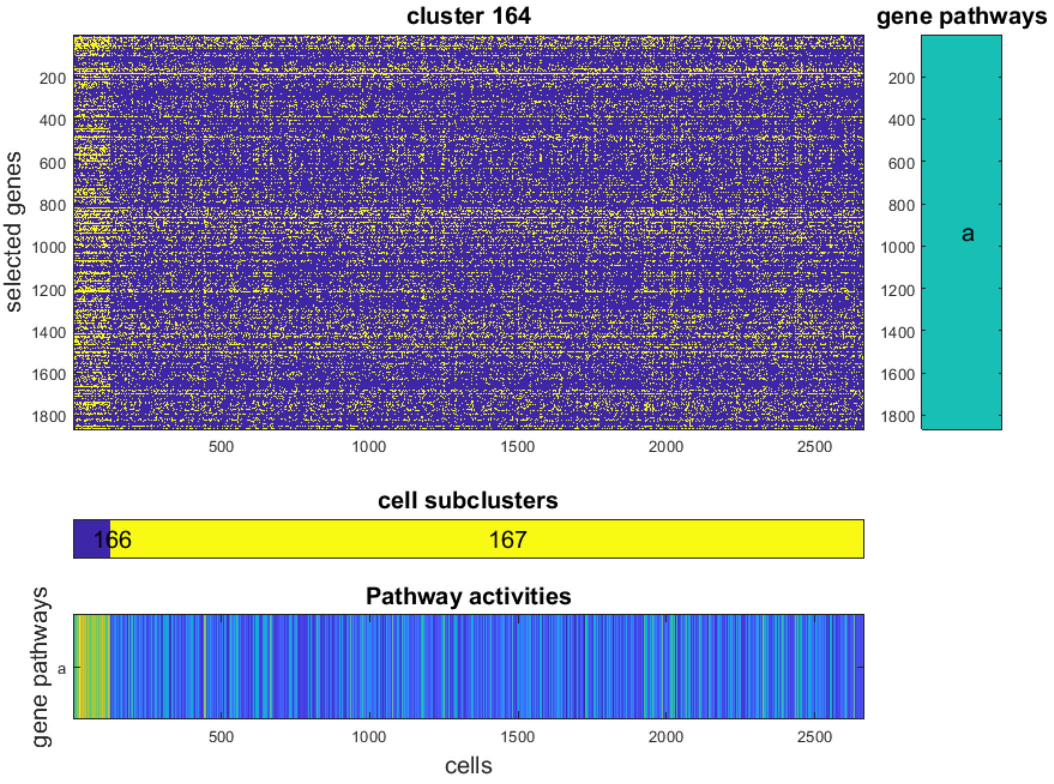


```
Remaining clusters to partition 3
Processing cluster 165 now ...
Processing data subset with 22327 genes and 263 cells:
Remove genes detected in <100 cells. Remaining 1245 genes.  Elapsed time is 0.017193 seconds.
Iterate 10 random permutations for gene-gene similarity threshold ...   10 Elapsed time is 1.196596 seconds.
Compute gene-gene similarity ... Elapsed time is 0.092114 seconds.
Create gene-gene graph for clustering genes ...
Writing graph into file ... 100%Elapsed time is 0.018492 seconds.
Running ModularityOptimizer for clustering ...Elapsed time is 0.342215 seconds.
Gene-gene graph contains 4 pathways, 229 genes in total
Elapsed time is 0.373142 seconds.
Create cell-cell graph for clustering cells ...
Writing graph into file ... 100%Elapsed time is 0.029500 seconds.
Running ModularityOptimizer for clustering ...Elapsed time is 0.328237 seconds.
Cell-cell graph contains 5 cell types by community detection
Elapsed time is 0.335926 seconds.
Cell-cell graph contains 5 cell types after merging tiny cell clusters
Cell-cell graph contains 2 cell types after merging
Number of useful pathways is 1
```

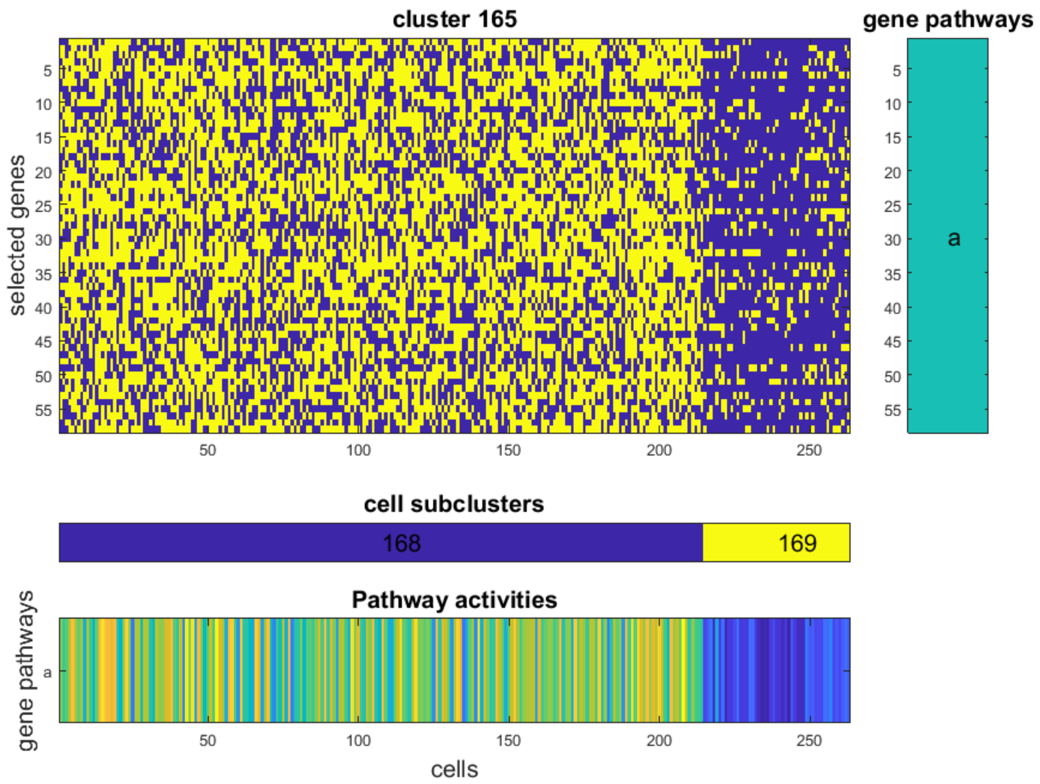


```
Remaining clusters to partition 4
Processing cluster 166 now ...
Processing data subset with 22327 genes and 126 cells:
Remove genes detected in <100 cells. Remaining 0 genes.  Elapsed time is 0.007136 seconds.

Remaining clusters to partition 3
Processing cluster 167 now ...
Processing data subset with 22327 genes and 2541 cells:
Remove genes detected in <100 cells. Remaining 10980 genes.  Elapsed time is 0.240594 seconds.
Iterate 10 random permutations for gene-gene similarity threshold ...   10 Elapsed time is 135.099660 seconds.
Compute gene-gene similarity ... Elapsed time is 10.105619 seconds.
Create gene-gene graph for clustering genes ...
Writing graph into file ... 100%Elapsed time is 3.899682 seconds.
Running ModularityOptimizer for clustering ...Elapsed time is 9.757137 seconds.
Gene-gene graph contains 7 pathways, 7815 genes in total
Elapsed time is 10.162736 seconds.
Create cell-cell graph for clustering cells ...
Writing graph into file ... 100%Elapsed time is 0.298460 seconds.
Running ModularityOptimizer for clustering ...Elapsed time is 1.334409 seconds.
Cell-cell graph contains 15 cell types by community detection
Elapsed time is 1.388032 seconds.
Cell-cell graph contains 13 cell types after merging tiny cell clusters
creating a total of 12 edges ...     12
Cell-cell graph contains 2 cell types after merging
Number of useful pathways is 1
```

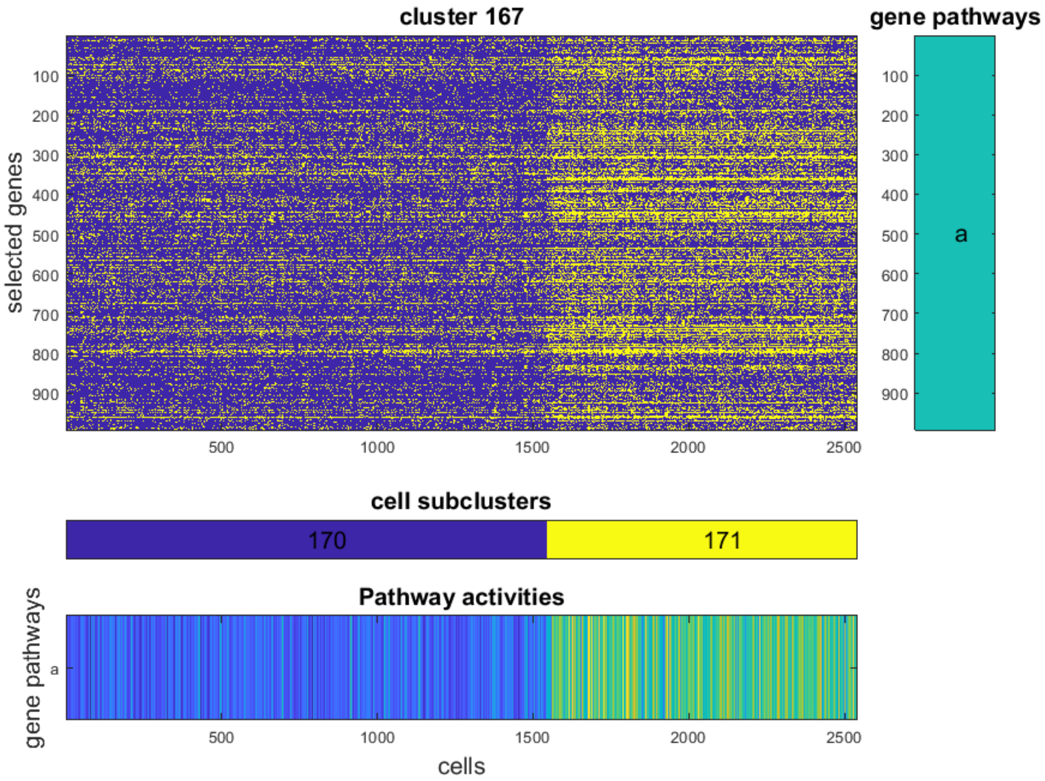


```
Remaining clusters to partition 4
Processing cluster 168 now ...
Processing data subset with 22327 genes and 214 cells:
Remove genes detected in <100 cells. Remaining 331 genes.  Elapsed time is 0.012404 seconds.
Iterate 10 random permutations for gene-gene similarity threshold ...   10 Elapsed time is 0.076025 seconds.
Compute gene-gene similarity ... Elapsed time is 0.003610 seconds.
Create gene-gene graph for clustering genes ...
Writing graph into file ... 102%Elapsed time is 0.002152 seconds.
Running ModularityOptimizer for clustering ...Elapsed time is 0.200848 seconds.
Gene-gene graph contains 0 pathways, 0 genes in total
Elapsed time is 0.210166 seconds.

Remaining clusters to partition 3
Processing cluster 169 now ...
Processing data subset with 22327 genes and 49 cells:
Remove genes detected in <100 cells. Remaining 0 genes.  Elapsed time is 0.003252 seconds.

Remaining clusters to partition 2
Processing cluster 170 now ...
Processing data subset with 22327 genes and 1546 cells:
Remove genes detected in <100 cells. Remaining 9734 genes.  Elapsed time is 0.149128 seconds.
Iterate 10 random permutations for gene-gene similarity threshold ...   10 Elapsed time is 90.927089 seconds.
Compute gene-gene similarity ... Elapsed time is 6.737741 seconds.
Create gene-gene graph for clustering genes ...
Writing graph into file ... 100%Elapsed time is 3.519497 seconds.
Running ModularityOptimizer for clustering ...Elapsed time is 9.758488 seconds.
Gene-gene graph contains 5 pathways, 6956 genes in total
Elapsed time is 10.096105 seconds.
Create cell-cell graph for clustering cells ...
Writing graph into file ... 100%Elapsed time is 0.174306 seconds.
Running ModularityOptimizer for clustering ...Elapsed time is 0.830250 seconds.
Cell-cell graph contains 13 cell types by community detection
Elapsed time is 0.863990 seconds.
Cell-cell graph contains 13 cell types after merging tiny cell clusters
creating a total of 12 edges ...     12
Cell-cell graph contains 2 cell types after merging
Number of useful pathways is 1
```

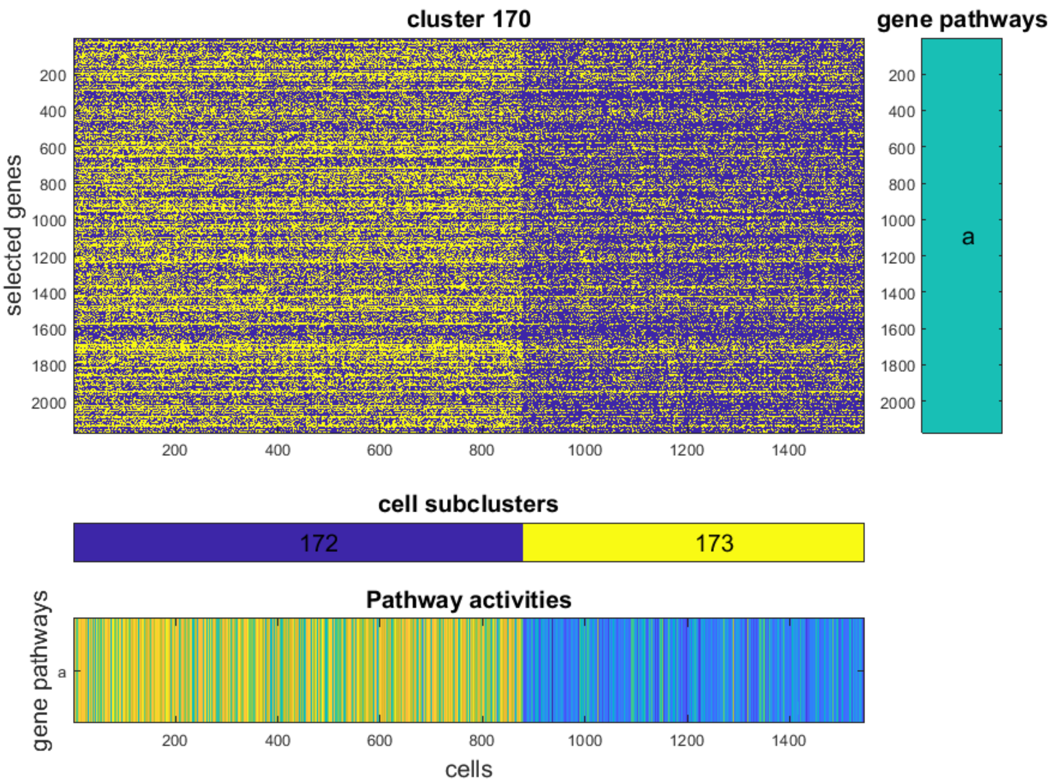


```
Remaining clusters to partition 3
Processing cluster 171 now ...
Processing data subset with 22327 genes and 995 cells:
Remove genes detected in <100 cells. Remaining 8047 genes.  Elapsed time is 0.088498 seconds.
Iterate 10 random permutations for gene-gene similarity threshold ...   10 Elapsed time is 54.509463 seconds.
Compute gene-gene similarity ... Elapsed time is 4.228664 seconds.
Create gene-gene graph for clustering genes ...
Writing graph into file ... 100%Elapsed time is 0.475360 seconds.
Running ModularityOptimizer for clustering ...Elapsed time is 0.842727 seconds.
Gene-gene graph contains 7 pathways, 1533 genes in total
Elapsed time is 1.106786 seconds.
Create cell-cell graph for clustering cells ...
Writing graph into file ... 100%Elapsed time is 0.116958 seconds.
Running ModularityOptimizer for clustering ...Elapsed time is 0.705564 seconds.
Cell-cell graph contains 9 cell types by community detection
Elapsed time is 0.728515 seconds.
Cell-cell graph contains 9 cell types after merging tiny cell clusters
creating a total of 8 edges ...      8
Cell-cell graph contains 2 cell types after merging
Number of useful pathways is 1
```

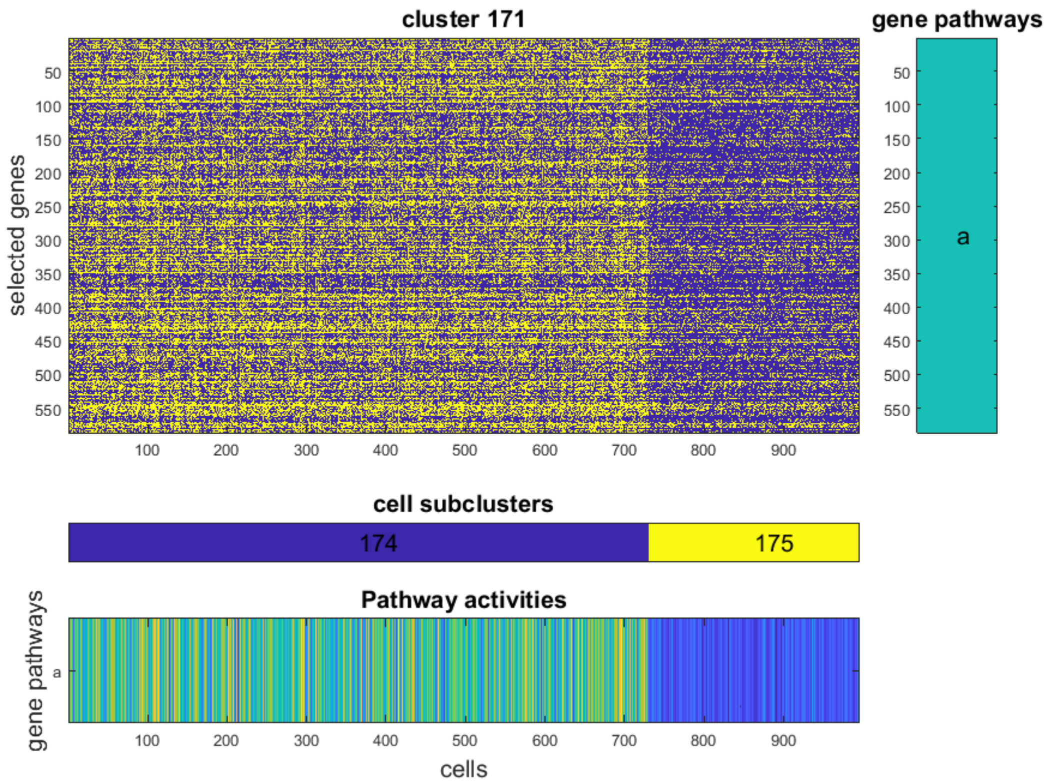


```
Remaining clusters to partition 4
Processing cluster 172 now ...
Processing data subset with 22327 genes and 879 cells:
Remove genes detected in <100 cells. Remaining 7806 genes.  Elapsed time is 0.074682 seconds.
Iterate 10 random permutations for gene-gene similarity threshold ...   10 Elapsed time is 50.618362 seconds.
Compute gene-gene similarity ... Elapsed time is 3.908691 seconds.
Create gene-gene graph for clustering genes ...
Writing graph into file ... 100%Elapsed time is 0.692269 seconds.
Running ModularityOptimizer for clustering ...Elapsed time is 1.237805 seconds.
Gene-gene graph contains 6 pathways, 2547 genes in total
Elapsed time is 1.496081 seconds.
Create cell-cell graph for clustering cells ...
Writing graph into file ... 100%Elapsed time is 0.105012 seconds.
Running ModularityOptimizer for clustering ...Elapsed time is 0.602283 seconds.
Cell-cell graph contains 10 cell types by community detection
Elapsed time is 0.623465 seconds.
Cell-cell graph contains 10 cell types after merging tiny cell clusters
creating a total of 9 edges ...      9
Cell-cell graph contains 1 cell types after merging

Remaining clusters to partition 3
Processing cluster 173 now ...
Processing data subset with 22327 genes and 667 cells:
Remove genes detected in <100 cells. Remaining 6097 genes.  Elapsed time is 0.052809 seconds.
Iterate 10 random permutations for gene-gene similarity threshold ...   10 Elapsed time is 29.642109 seconds.
Compute gene-gene similarity ... Elapsed time is 2.339522 seconds.
Create gene-gene graph for clustering genes ...
Writing graph into file ... 100%Elapsed time is 0.753785 seconds.
Running ModularityOptimizer for clustering ...Elapsed time is 1.768754 seconds.
Gene-gene graph contains 4 pathways, 3399 genes in total
Elapsed time is 1.954465 seconds.
Create cell-cell graph for clustering cells ...
Writing graph into file ... 100%Elapsed time is 0.073480 seconds.
Running ModularityOptimizer for clustering ...Elapsed time is 0.473840 seconds.
Cell-cell graph contains 8 cell types by community detection
Elapsed time is 0.489650 seconds.
Cell-cell graph contains 8 cell types after merging tiny cell clusters
creating a total of 7 edges ...      7
Cell-cell graph contains 2 cell types after merging
Number of useful pathways is 1
```

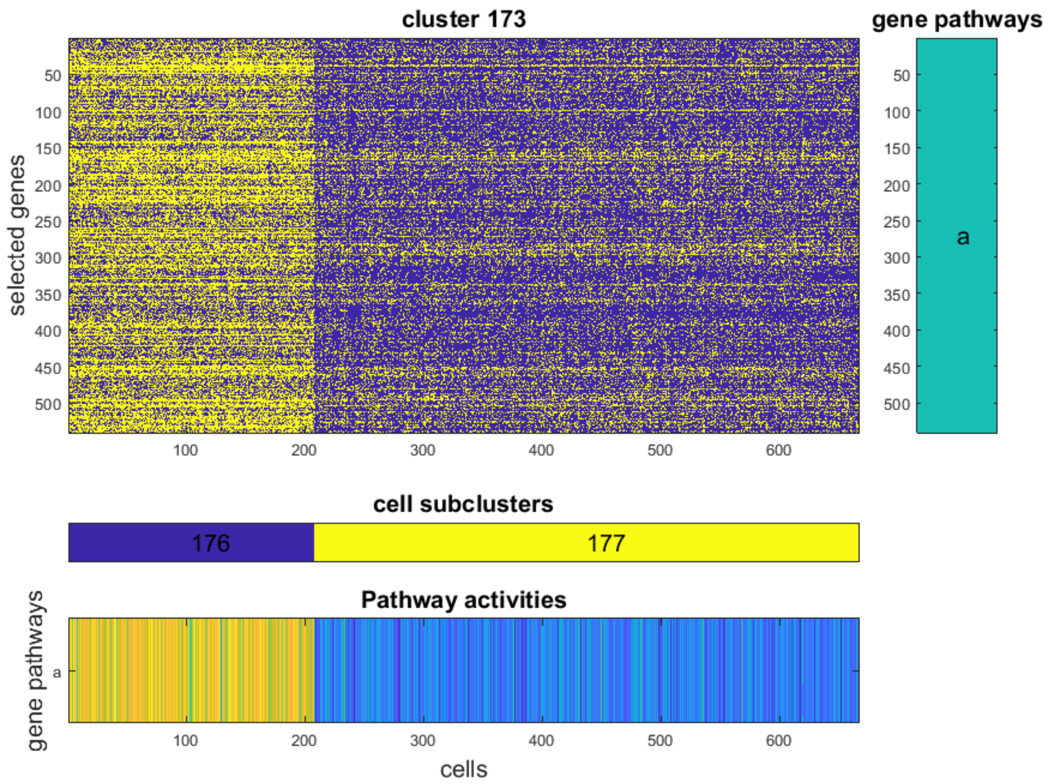


```
Remaining clusters to partition 4
Processing cluster 174 now ...
Processing data subset with 22327 genes and 730 cells:
Remove genes detected in <100 cells. Remaining 7074 genes.  Elapsed time is 0.063023 seconds.
Iterate 10 random permutations for gene-gene similarity threshold ...   10 Elapsed time is 40.023569 seconds.
Compute gene-gene similarity ... Elapsed time is 3.042358 seconds.
Create gene-gene graph for clustering genes ...
Writing graph into file ... 100%Elapsed time is 0.335482 seconds.
Running ModularityOptimizer for clustering ...Elapsed time is 0.703219 seconds.
Gene-gene graph contains 7 pathways, 1097 genes in total
Elapsed time is 0.921511 seconds.
Create cell-cell graph for clustering cells ...
Writing graph into file ... 100%Elapsed time is 0.086528 seconds.
Running ModularityOptimizer for clustering ...Elapsed time is 0.588854 seconds.
Cell-cell graph contains 7 cell types by community detection
Elapsed time is 0.605926 seconds.
Cell-cell graph contains 6 cell types after merging tiny cell clusters
creating a total of 5 edges ...       5
Cell-cell graph contains 1 cell types after merging

Remaining clusters to partition 3
Processing cluster 175 now ...
Processing data subset with 22327 genes and 265 cells:
Remove genes detected in <100 cells. Remaining 1494 genes.  Elapsed time is 0.019805 seconds.
Iterate 10 random permutations for gene-gene similarity threshold ...   10 Elapsed time is 1.703502 seconds.
Compute gene-gene similarity ... Elapsed time is 0.138133 seconds.
Create gene-gene graph for clustering genes ...
Writing graph into file ... 101%Elapsed time is 0.016729 seconds.
Running ModularityOptimizer for clustering ...Elapsed time is 0.273767 seconds.
Gene-gene graph contains 0 pathways, 0 genes in total
Elapsed time is 0.310149 seconds.

Remaining clusters to partition 2
Processing cluster 176 now ...
Processing data subset with 22327 genes and 207 cells:
Remove genes detected in <100 cells. Remaining 212 genes.  Elapsed time is 0.011875 seconds.
Iterate 10 random permutations for gene-gene similarity threshold ...   10 Elapsed time is 0.043088 seconds.
Compute gene-gene similarity ... Elapsed time is 0.002136 seconds.
Create gene-gene graph for clustering genes ...
Writing graph into file ... 106%Elapsed time is 0.001652 seconds.
Running ModularityOptimizer for clustering ...Elapsed time is 0.179594 seconds.
Gene-gene graph contains 0 pathways, 0 genes in total
Elapsed time is 0.186360 seconds.

Remaining clusters to partition 1
Processing cluster 177 now ...
Processing data subset with 22327 genes and 460 cells:
Remove genes detected in <100 cells. Remaining 4480 genes.  Elapsed time is 0.036441 seconds.
Iterate 10 random permutations for gene-gene similarity threshold ...   10 Elapsed time is 15.012456 seconds.
Compute gene-gene similarity ... Elapsed time is 1.153939 seconds.
Create gene-gene graph for clustering genes ...
Writing graph into file ... 100%Elapsed time is 0.372269 seconds.
Running ModularityOptimizer for clustering ...Elapsed time is 1.234549 seconds.
Gene-gene graph contains 4 pathways, 2231 genes in total
Elapsed time is 1.362301 seconds.
Create cell-cell graph for clustering cells ...
```

```
Writing graph into file ... 100%Elapsed time is 0.053700 seconds.
Running ModularityOptimizer for clustering ...Elapsed time is 0.404281 seconds.
Cell-cell graph contains 7 cell types by community detection
Elapsed time is 0.416050 seconds.
Cell-cell graph contains 7 cell types after merging tiny cell clusters
creating a total of 6 edges ...      6
Cell-cell graph contains 2 cell types after merging
Number of useful pathways is 2
```

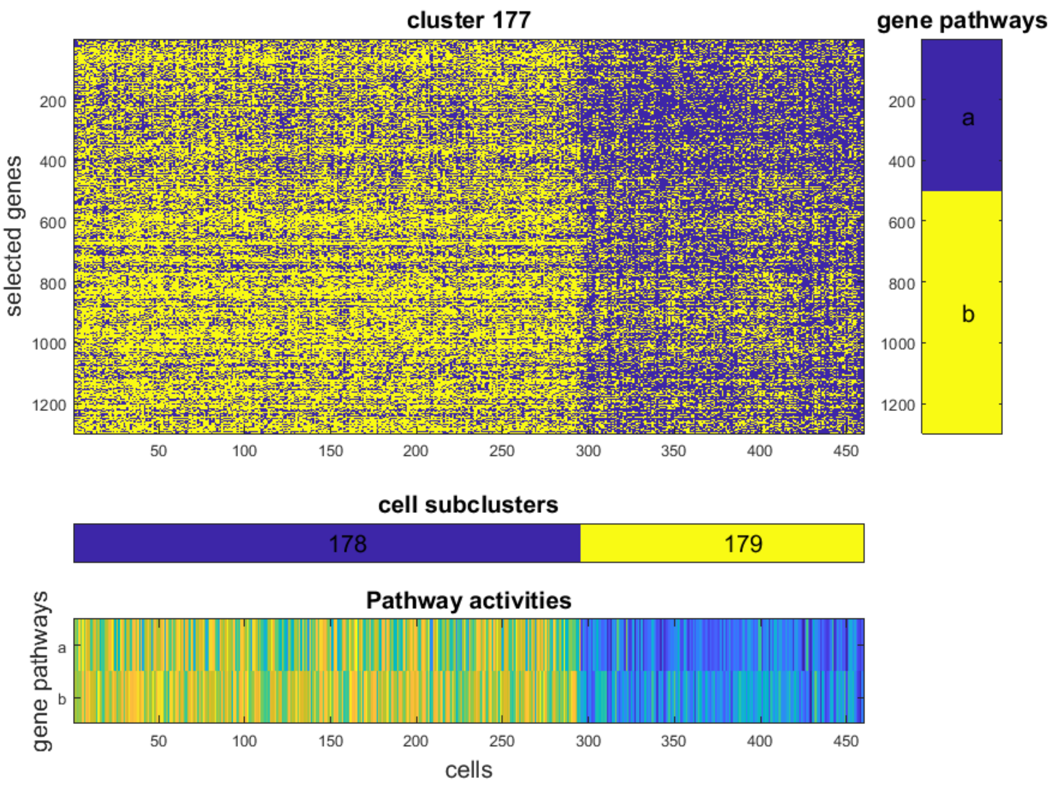


```
Remaining clusters to partition 2
Processing cluster 178 now ...
Processing data subset with 22327 genes and 295 cells:
Remove genes detected in <100 cells. Remaining 2274 genes.  Elapsed time is 0.024064 seconds.
Iterate 10 random permutations for gene-gene similarity threshold ...   10 Elapsed time is 4.038299 seconds.
Compute gene-gene similarity ... Elapsed time is 0.312180 seconds.
Create gene-gene graph for clustering genes ...
Writing graph into file ... 100%Elapsed time is 0.053911 seconds.
Running ModularityOptimizer for clustering ...Elapsed time is 0.446623 seconds.
Gene-gene graph contains 4 pathways, 532 genes in total
Elapsed time is 0.504877 seconds.
Create cell-cell graph for clustering cells ...
Writing graph into file ... 100%Elapsed time is 0.033606 seconds.
Running ModularityOptimizer for clustering ...Elapsed time is 0.314881 seconds.
Cell-cell graph contains 7 cell types by community detection
Elapsed time is 0.323430 seconds.
Cell-cell graph contains 7 cell types after merging tiny cell clusters
creating a total of 6 edges ...      6
Cell-cell graph contains 2 cell types after merging
Number of useful pathways is 1
```

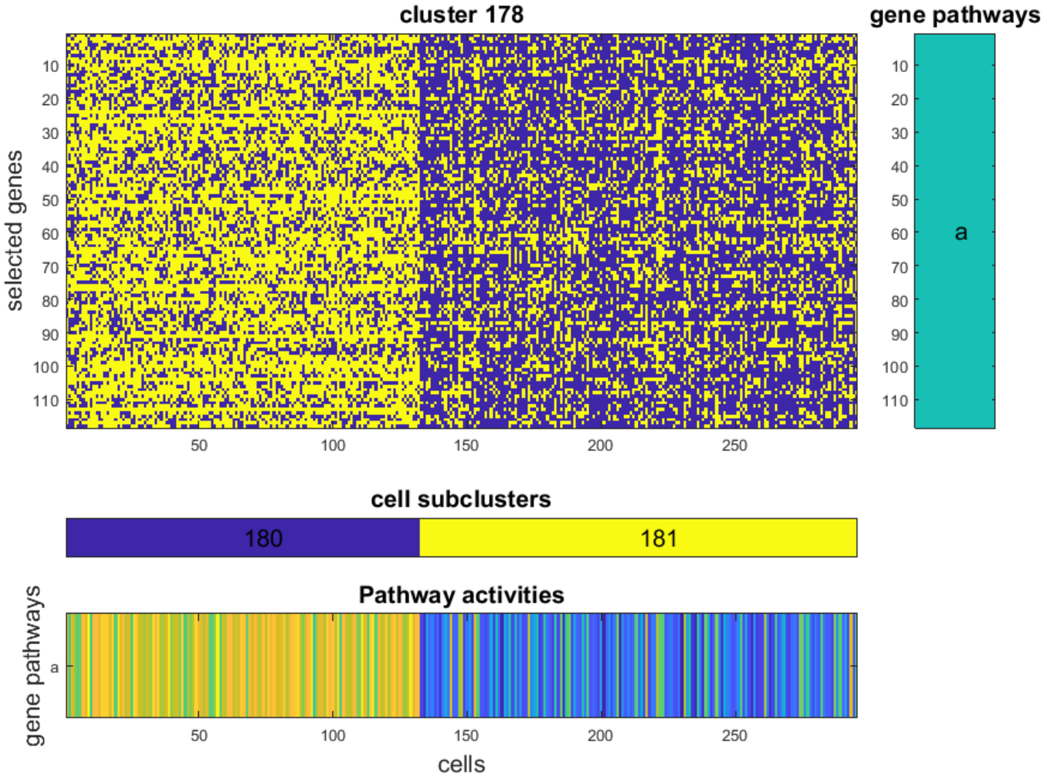


```
Remaining clusters to partition 3
Processing cluster 179 now ...
Processing data subset with 22327 genes and 165 cells:
Remove genes detected in <100 cells. Remaining 0 genes.  Elapsed time is 0.010843 seconds.

Remaining clusters to partition 2
Processing cluster 180 now ...
Processing data subset with 22327 genes and 132 cells:
Remove genes detected in <100 cells. Remaining 0 genes.  Elapsed time is 0.008518 seconds.

Remaining clusters to partition 1
Processing cluster 181 now ...
Processing data subset with 22327 genes and 163 cells:
Remove genes detected in <100 cells. Remaining 0 genes.  Elapsed time is 0.011964 seconds.
```

number of cooccurrence clusters

```
number_of_cooccurrence_clusters = length(unique(cooc.cell_labels))
```

```
number_of_cooccurrence_clusters =

    96
```

Published with MATLAB® R2017b
